# Primary α-tertiary amine synthesis *via* α-C–H functionalization†
†Electronic supplementary information (ESI) available. CCDC 1851038. For ESI and crystallographic data in CIF or other electronic format see DOI: 10.1039/c8sc05164j


**DOI:** 10.1039/c8sc05164j

**Published:** 2019-02-08

**Authors:** Dhananjayan Vasu, Angel L. Fuentes de Arriba, Jamie A. Leitch, Antoine de Gombert, Darren J. Dixon

**Affiliations:** a Department of Chemistry , Chemistry Research Laboratory , University of Oxford , 12 Mansfield Road , Oxford , UK . Email: darren.dixon@chem.ox.ac.uk

## Abstract

A quinone-mediated general synthetic platform for the construction of primary α-tertiary amines from abundant primary α-branched amine starting materials is described. This procedure pivots on the efficient *in situ* generation of reactive ketimine intermediates and subsequent reaction with carbon-centered nucleophiles such as organomagnesium and organolithium reagents, and TMSCN, creating quaternary centers. Furthermore, extension to reverse polarity photoredox catalysis enables reactivity with electrophiles, *via* a nucleophilic α-amino radical intermediate. This efficient, broadly applicable and scalable amine-to-amine synthetic platform was successfully applied to library and API synthesis and in the functionalization of drug molecules.

## Abstract 2

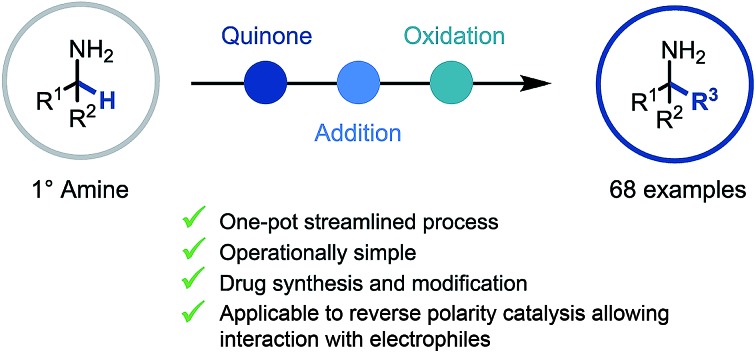
A reactive ketimine intermediate was demonstrated to be intercepted by a variety of nucleophiles including organometallics and TMSCN.

## Introduction

**Scheme 1:**
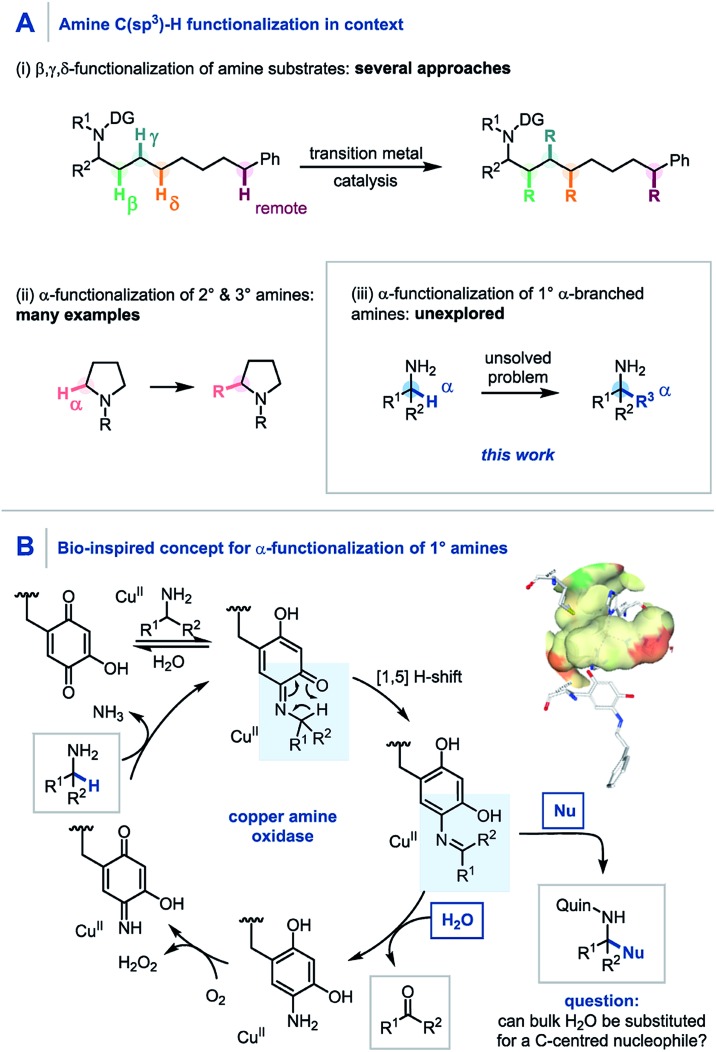
(A) C–H functionalization of amines. (B) Copper amine oxidase mechanism using a quinone co-factor.

Through direct applications in the modification of biologically relevant molecules, the construction of challenging building blocks, and the synthesis of natural products, C(sp^3^)–H functionalization has come to the forefront of modern synthetic methodology development.^1^ Along these lines, the persistent need from drug discovery programmes for elaborate amine containing architectures has led to pioneering developments in the C(sp^3^)–H functionalization of amines.^2^ Elegant use of transition metal catalysis, often in conjunction with a directing group strategy, has enabled the selective β,^3^ γ,^4^ δ,^5^ and remote^6^ C(sp^3^)–H functionalization of amines through bespoke and meticulously tailored catalyst and/or reaction systems (Scheme 1A). To date, the α-functionalization of amines has been widely achieved using primary,^7^,^8^ secondary^9^ and tertiary^10^ amines, including a quinone-mediated α-functionalization of pyrrolidine derivatives developed by Qu and co-workers.9*a* However, despite these advances, there still remains no general protocol to construct primary α-tertiary amines *via* C–H functionalization of α-branched amines. Accordingly, we believed that developing a practical method to readily access these important primary α-tertiary amine scaffolds would find widespread utility, owing to the abundance of α-branched amines as feedstock chemicals, building blocks for library synthesis, and branch-point intermediates in drug discovery programmes.

Pioneering studies by Klinman and Mure,^11^ and Sayre,^12^ among others^13^ have elucidated the role and mechanism of quinone co-factors in copper amine oxidases (CuAOs). CuAOs are a family of metalloenzymes which selectively catalyze the oxidation of primary amines into aldehydes (Scheme 1B, R^2^ = H) using molecular oxygen through the combination of a quinone-based co-factor and a Cu^II^ species.^14^ The mechanism involves the condensation of a quinone co-factor with the primary amine substrate and a subsequent formal [1,5] H-shift from the α-position of the amine to generate a reactive imine, which is then hydrolyzed to afford the aldehyde product. Despite elegant early work on quinone-mediated amine oxidation by McCoy and Day,^15^ and Corey,^16^ the elucidation of the mechanism of this biotransformation has paved the way for increasing applications in contemporary synthesis^17^ with notable contributions from Stahl,^18^ Kobayashi,^19^ and Fleury^20^ in amine oxidation, from Qu,9a,^21^ and Clift7*a* in amine functionalization, and from Lumb in heterocycle synthesis.^22^,^23^

Aligned to this enzymatic process and previous synthetic reports we hypothesized that if the reactive imine formed by quinone oxidation (Scheme 1B, R^2^ ≠ H) could be intercepted by appropriate nucleophiles for efficient carbon–carbon bond formation, a new synthetic platform for the generation of α-fully substituted primary amines could be realized. Herein we wish to report our findings.

## Results & discussion

**Scheme 2:**
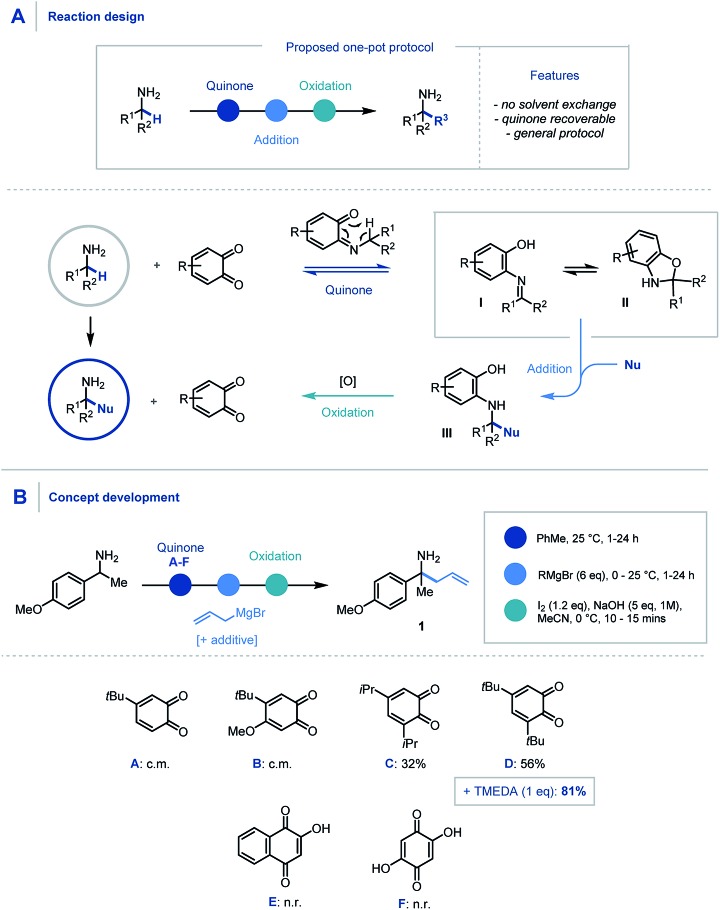
(A) Proposed reaction design using quinone mediator. (B) Discovery and optimization of allylation of α-branched primary amines.

We envisaged that the addition of a suitable quinone to an α,α-disubstituted primary amine would afford the Schiff base intermediate (Scheme 2A) which would undergo an *in situ* [1,5] H-shift creating a reactive intermediary ketimine structure **I**. It has been widely reported previously that such imines exist in equilibrium with the corresponding hemiaminal **II**.^17^,^24^ Carbon–carbon bond formation *via* nucleophilic addition and subsequent oxidation of **III** would allow hydrolytic removal of the quinone releasing the high value primary α-tertiary amine. From the outset we considered it important for the procedure to have no inter-step manipulation or solvent exchange in order to streamline this one-pot platform for primary α-C–H functionalization of amines.

In order to assess feasibility, a selection of substituted quinones was studied in the allylation of α-methyl-*p*-methoxybenzylamine as a model system (Scheme 2B). Toluene was identified as the preferred reaction solvent and following condensation of the quinone and amine, sequential addition of excess allyl Grignard reagent was required for full conversion of ketimine, which in turn aided product purification. A simple, oxidative hydrolytic work-up using iodine and NaOH (1 M)9*a* was sufficient to detach the hydroxyarene from the desired α-allylated 1°-amine product **1**. Following this sequential procedure, whereas quinones **A** & **B** led to complex product mixtures (for full optimization details see ESI†), quinones **C** & **D** did indeed provide access to the α-allylated product **1** in good yields over the three-stage, one-pot sequence. Pleasingly, the inclusion of TMEDA (1 eq.) in the nucleophilic alkylation step resulted in cleaner addition leading to a significant increase in the product yield (81%).^25^ Quinones **E** & **F** were found to be ineffective in this protocol due to their poor solubility in the reaction medium and a solvent change provided no improvement in comparison to quinone **D**.

**Scheme 3:**
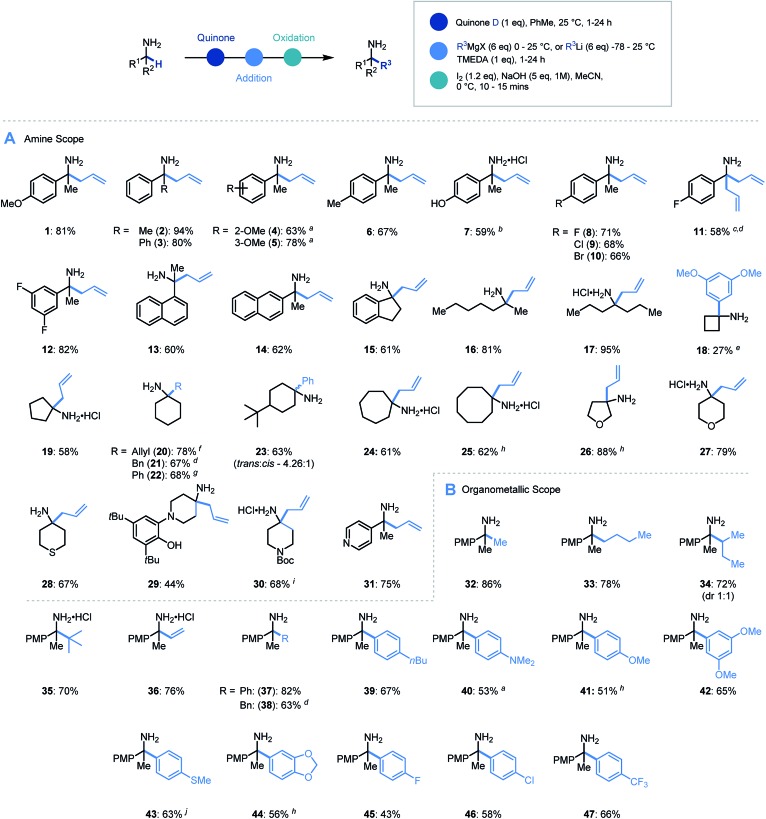
Substrate scope for primary amine α-functionalization. (A) Scope of α-branched amines. (B) Scope of organometallic reagents. ^a^ 3 eq. oxidant used. ^b^ THF : PhMe (5 : 1) used as solvent for step (i). ^c^ 2-Amino-2-(4-fluorophenyl)acetonitrile used as starting material and step (i) carried out at 80 °C. ^d^ 10 eq. organometallic reagent was used. ^e^ DCE used as solvent for step (i) and exchanged to toluene before organolithium addition. ^f^ Isolated as HCl salt. ^g^ Isolated as benzoylated product. ^h^ 2 eq. oxidant used. ^i^ Pyrrolidine (1.2 eq.) added. ^j^ 5 eq. oxidant used.

With an efficient protocol in hand, we sought to establish the scope with a variety of primary α-branched amine substrates. Investigations began using the model allylation procedure described above with a wide range of α-substituted benzylamine structures (Scheme 3A, **1–14**).^26^ Pleasingly, the use of α-methylbenzylamine (**2**) gave excellent yields of the α-allylated amine product, and benzhydrylamine (**3**) also proceeded efficiently in the reaction. The chemistry was tolerant of methoxy substituents in the *ortho* or *meta* positions (**4**, **5**). Substrates possessing alkyl (**6**), hydroxyl (**7**) and halogen (**8–12**) functionality present on the aromatic ring all performed efficiently in the reaction thereby demonstrating its tolerance to electronic variation. Interestingly, when 2-amino-2-(4-fluorophenyl)acetonitrile was employed as a substrate, it was observed that the allylated amine intermediate (**III**, with respect to Scheme 2A) underwent an *in situ* elimination of cyanide followed by a second addition of the Grignard reagent, affording diallylated structure **11** after oxidation. The biologically relevant 1-indanamine scaffold (relevant to compounds for early onset Parkinson's treatment)^27^ was shown to partake in this chemistry in good yield (**15**). The transition from benzylic to aliphatic amines was achieved with remarkable success, with excellent yields on branched aminoheptane derivatives (**16**, **17**). Cyclic systems were shown to be effective substrates for the allylation platform (**18–30**), with larger ring and heterocyclic systems achieving good to excellent yields, and even challenging cyclobutane units affording α-functionalized products (**18**). Pleasingly heteroaryl substituted amine (**31**) granted access to the α-functionalized product effectively. It should be noted that in this study the quinone can be isolated and reused with no detriment to reaction efficiency.

Having established the scope with respect to the amine component it was of interest to study the performance of diverse organometallic reagents^28^ using 1-(4-methoxyphenyl)ethan-1-amine as a model α-branched amine (Scheme 3B). Initial studies using aliphatic organomagnesium reagents were disappointing (with and without TMEDA additive). Despite this, a survey of organometallic alternatives identified simple aliphatic organolithium reagents as effective coupling partners. To this end, methyl (**32**), *n*-butyl (**33**), *s*-butyl (**34**), and *t*-butyl (**35**) alkyl groups were installed using their corresponding organolithium with good to excellent efficiency. The *tert*-butylation demonstrates that the steric profile of this methodology can easily accommodate the construction of neighbouring quaternary centres. We were pleased to find that the general protocol was effectively applied to a range of aryllithiums with varied electronic profiles (**37**, **39–47**).

**Scheme 4:**
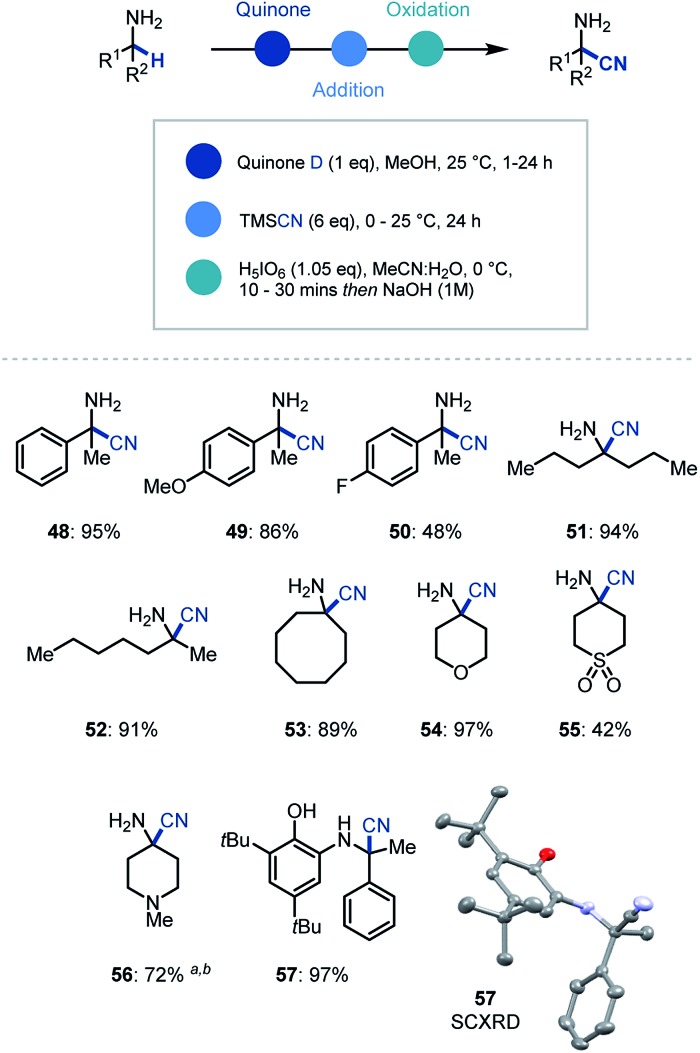
Substrate scope for primary amine α-functionalization using cyanide. ^a^ 10 eq. TMSCN was used. ^b^ Oxidation (1 h).

In addition to organometallic reagents we were keen to identify other nucleophilic species that could intercept the reactive ketimine intermediate. Due to the synthetic versatility of nitrile substituents, we decided to investigate their incorporation at the α position of primary α-branched amines.^29^ Pleasingly, we found that the use of TMSCN as a nucleophilic source of cyanide and in MeOH as solvent in the initial ketimine forming step, led to near quantitative addition to the imine (Scheme 4). Despite this, the conditions for oxidative cleavage used previously were ineffective (see ESI† for details), however orthoperiodic acid (H_5_IO_6_) was identified as an excellent replacement. This modified protocol allowed, after a basic work-up, the clean isolation of the α-aminonitrile product in 95% yield (**48**). We then expanded the scope of this methodology to electron rich (**49**) and electron deficient (**50**) arenes, as well as linear (**51**, **52**) and cyclic (**53–56**) amines with excellent yields for the one-pot multi-step process. Over the course of establishing the scope, α-aminonitrile addition intermediate was isolated and characterized *via* single crystal X-ray analysis (**57**), thus confirming our proposed reaction pathway.

**Scheme 5:**
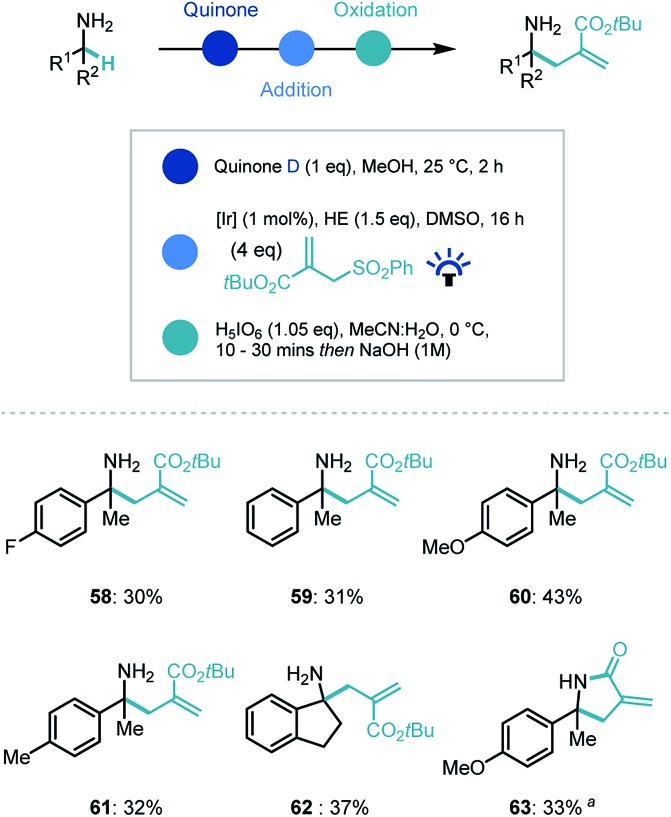
Substrate scope for photocatalytic reverse polarity primary amine α-functionalization. ^a^ Ethyl-2-((phenylsulfonyl)methyl)acrylate used as coupling partner.

Recently our group and others have demonstrated that photoredox catalysis can reverse the polarity of an imine moiety to enable new reactivity.^30^ Proton coupled electron transfer (PCET) reduction of an imine can generate a nucleophilic α-amino radical which can be intercepted by electrophiles.^31^ We recognized that applying this umpolung concept to the quinone-generated ketimine intermediate, could expand the capability of the synthetic platform (Scheme 5). Indeed, when the ketimine intermediate was treated with a photocatalyst ([Ir(dF(CF_3_)ppy)_2_(dtbpy)]PF_6_, [Ir]), the commercial Hantzsch ester (HE), an electrophilic radical acceptor (*tert*-butyl-2-((phenylsulfonyl)methyl)acrylate) in DMSO and irradiated with blue light, we were delighted to observe α-allylated product in moderate to good yields for the overall process despite a challenging photocatalytic step (**58–62**). Interestingly, when the ethyl ester derivative of the coupling partner was employed, an *in situ* lactamization took place on addition of NaOH (**63**).

**Scheme 6:**
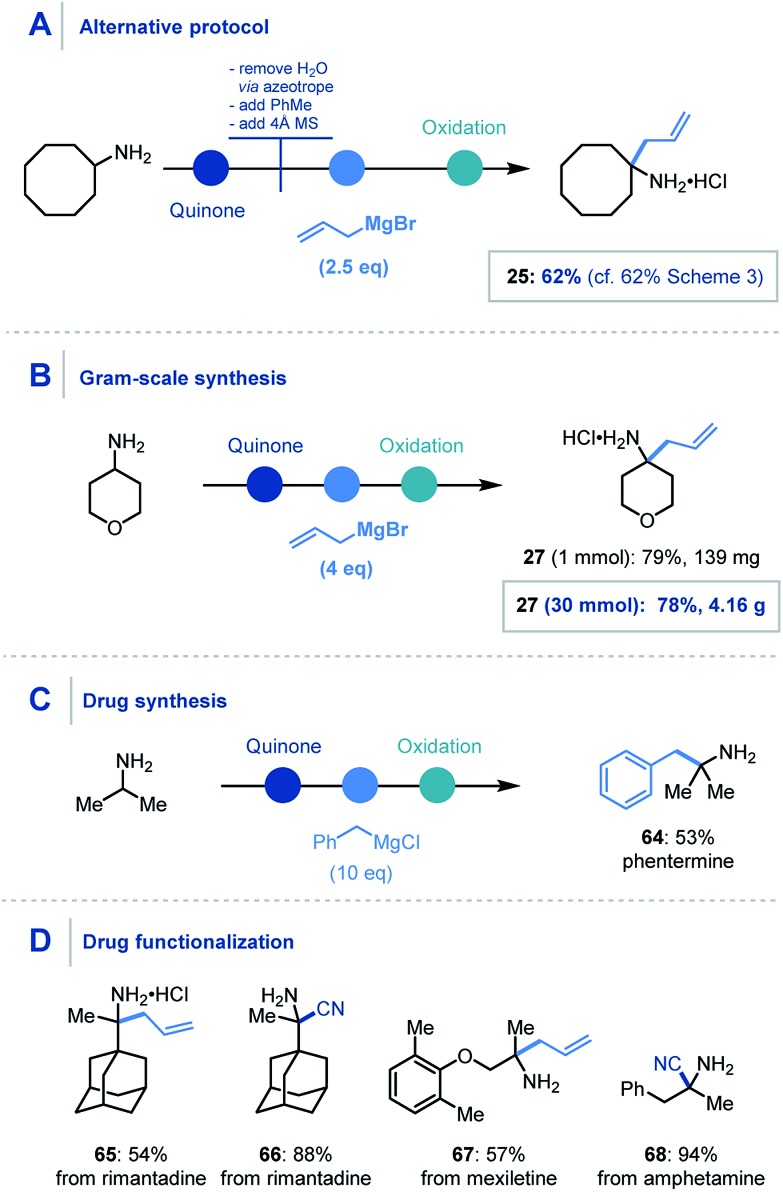
Concept application: (A) alternative protocol. (B) Gram-scale synthesis. (C) Drug synthesis. (D) API functionalization. Unspecified conditions are the same as Scheme 3 and 4 for organometallic addition and cyanide addition respectively.

For the organometallic addition stage, at least three equivalents of the organometallic reagent are required, as two equivalents are necessarily consumed in sequestering the water (produced in the first step) and then the intermediary phenol. Despite this, addition of 6 equivalents of the organometallic was found to be optimal and reliable throughout the scope. However, using a modified protocol – where azeotropic removal of water after the first step was carried out – 2.5 equivalents of the nucleophile could be employed to achieve comparable yields to the streamlined platform (Scheme 6A).

We also found that this methodology could be applied on gram-scale, where the α-allylation of 30 mmol of 4-aminopyran using four equivalents of allylmagnesium bromide (Scheme 6B) was achieved.^32^ Pleasingly, the reaction proceeded smoothly and afforded over 4 g of α-functionalized product **27**.

Furthermore, the utility of this methodology was also highlighted in the one-step synthesis of the anorectic drug phentermine (**64**), where previous reports utilize Strecker and Henry chemistry respectively and require multiple steps (Scheme 6C).^33^ A prominent advantage of this mild protocol is the potential application to derivatization of biologically relevant molecules for *inter alia* drug discovery. To demonstrate this, we were pleased to obtain α-allylated (**65**) and α-cyanated (**66**) derivatives of rimantadine, an α-allylated derivative of mexiletine (**67**) and α-cyanated amphetamine analogue (**68**) in good to excellent yields (Scheme 6D).

## Conclusions

In conclusion, a new synthetic platform for the ready construction of primary α-tertiary amines from primary α-branched amine substrates has been realized. This quinone-mediated chemistry has enabled α-allylation, α-alkylation, α-arylation, α-cyanation and photocatalytic reverse polarity α-allylation reactions to be carried out creating a fully substituted carbon center in the α-position of various amine structures. The new protocol is broad in scope, scalable and has been applied to a one-step synthesis of phentermine and in the functionalization of drug molecules. This new α-functionalization platform of α-branched primary amines should find widespread future applications ranging from complex amine diversification to drug molecule synthesis.

## Conflicts of interest

There are no conflicts to declare.

## Supplementary Material

Supplementary information

Supplementary information

Crystal structure data
