# Supplementary material for: Primary α-tertiary amine synthesis via α-C–H functionalization
Source: Chem Sci. 2019 Feb 8;10(11):3401–7. doi: 10.1039/c8sc05164j (PMC6429468; doi:10.1039/c8sc05164j)
Supplement: Supplementary file 2 [file SC-010-C8SC05164J-s002.pdf]

Electronic Supplementary Material (ESI) for Chemical Science.
This journal is © The Royal Society of Chemistry 2019

Supplementary Materials for

Primary α-Tertiary Amine Synthesis *via* α-C–H Functionalization

Dhananjayan Vasu, Ángel L. Fuentes de Arriba, Jamie A. Leitch, Antoine de Gombert,

Darren J. Dixon*

Correspondence to: *darren.dixon@chem.ox.ac.uk*

Table of Contents

1: General information: S3

2: Optimization study: S4

2.1: Optimization for organometallic addition: S4

2.2: Optimization for cyanation: S4

2.3: Optimization for oxidative cleavage of phenol unit: S5

3: General Procedures: S6

3.1: Synthesis of *o*-quinones: S6

3.1.1: Synthesis of 4-(*tert*-butyl)-*o*-benzoquinone (A): S6

3.1.2: Synthesis of 4-(*tert*-butyl)-5-methoxy-*o*-benzoquinone (B): S6

3.1.3: Synthesis of 3,5-diisopropyl-*o*-benzoquinone (C): S7

3.1.4: Synthesis of 3,5-di-*tert*-butyl-*o*-benzoquinone (D): S7

3.2: General Procedure A for α-C–H Functionalization of primary amines with Grignard reagent: S8

3.3: General Procedure B for α-C–H Functionalization of primary amines with Grignard reagent: S9

3.4 General Procedure C for α-C–H Functionalization of primary amines with organolithium reagents: S9

3.5 General Procedure D for oxidative cyanation of primary amines: S10

3.6: General Procedure E for the photocatalytic reverse polarity α-allylation: S11

3.7: Procedure for Alternative Protocol (Scheme 6A) S12

3.6.1: Photoreactor details: S13

3.6.2: Coupling partner synthesis: S14

4: Demonstration of stepwise procedure for one-pot operation: S18

5: Spectral data for *α*-functionalized products (1-68): S31

6: NMR Spectra: S57

7: X- ray structure and data for compound 57: S144

8: References: S147

1: General information:

All reagents bought from commercial sources were used as received. Organic solvents were evaporated under reduced pressure using a Büchi rotary evaporator. All solvents were commercially supplied or dried by filtration through activated alumina (powder ~150 mesh, pore size 58 Å, basic, Sigma-Aldrich) columns. Petrol ether (PE) refers to distilled light petroleum of fraction 30 - 40 °C. Toluene was distilled twice over calcium hydride.

All reactions were followed by thin-layer chromatography (TLC) when practical, using Merck Kieselgel 60 F254 fluorescent treated silica. Visualization was accomplished under UV light (λ_{max}= 254 nm) and by staining with potassium permanganate staining dip. Chromatographic purification was performed on VWR 60 silica gel 40-63 µm using HPLC grade solvents that were used as supplied. NMR spectra were recorded on Bruker spectrometers operating at 400 or 500 MHz (^{1}H resonance). Proton chemical shifts (δ) are given in parts per million (ppm) relative to tetramethylsilane (TMS) with the solvent resonance as internal standard: $CDCl_3$, δ = 7.26 ppm; CD_2Cl_2, δ = 5.32 ppm; CD_3CN, δ = 2.13 ppm; CD_3OD, δ = 3.31 ppm. The following abbreviations are used to describe spin multiplicity: s = singlet, d = doublet, t = triplet, q = quartet, dd = doublet of doublets, dt = doublet of triplets, ddt = doublet of doublet of triplets, m = multiplet, br s = broad signal. Coupling constants (*J*) are given in Hertz (Hz). ^{13}C NMR spectra were recorded with complete proton decoupling. Carbon chemical shifts are reported in ppm (δ) relative to TMS with the solvent resonance as internal standard: $CDCl_3$, δ = 77.16 ppm; CD_2Cl_2, δ = 53.84 ppm; CD_3CN, δ = 1.32 ppm. CD_3OD, δ = 49.00 ppm. Two-dimensional NMR spectroscopy experiments (COSY, HSQC and HMBC) were used where appropriate to assist in the assignment of signals in ^{1}H and ^{13}C spectra and data are not reported. High-resolution mass spectra (HRMS) were recorded on Bruker Daltonics MicroTOF mass spectrometer equipped with an ESI source. Infrared spectra (IR) were recorded on a Bruker Tensor 27 FT-IR spectrometer from a thin film on a diamond ATR module. Only selected maximum absorbances are reported. All other commercial reagents were used without further purification, unless otherwise indicated.

2: Optimization study:

2.1: Optimization for organometallic addition:

Table S1:

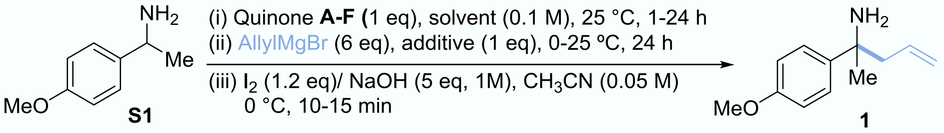

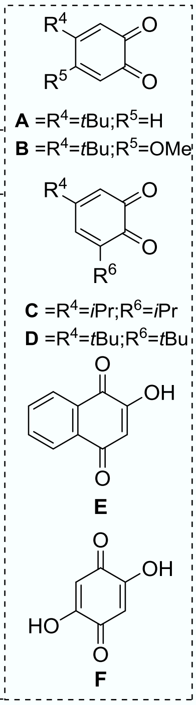

Entry	Quinone	Additive (1 equiv)	Solvent	Isolated yield (%)
1	A	---	PhMe	c.m
2	B	---	PhMe	c.m
3	C	---	PhMe	32
4	D	---	PhMe	56
**5**	**D**	**TMEDA**	**PhMe**	**81**
6	C	TMEDA	DCE	72
7	C	TMEDA	THF	52
8	C	TMEDA	Et_2O	51
9	C	TMEDA	Dioxane	24
10	E	---	DCE	n.r
11	F	---	DCE	n.r

Yields are reported after isolation from silica gel flash column chromatography. c.m = complex mixture in ketimine formation. n.r = represents no hemiaminal formation at both 25 ºC and 80 ºC in DCE. DCE = 1,2-Dichloroethane. TMEDA = N,N,N',N'-Tetramethylethylenediamine.

We began our investigation by screening various quinones against 1-(4-methoxyphenyl)ethan-1-amine with commercially available allylmagnesium bromide and the results are summarised in Table S1. Further screening in solvents reveal that toluene (entry 5) is superior to chlorinated (entry 6) or ethereal solvents (entries 7-9) under these standard conditions. DCE is superior to other solvents for *p*-quinones **E** and **F,** due to its poor solubility. No hemiaminal formation occurred with both *p*-quinones **E** and **F** (entries 10-11) with DCE at either 25 ˚C or 80 ˚C which suggest that are not suitable reagents for this transformation.

2.2: Optimization for cyanation:

After several attempts, we were delighted to found that the careful selection of solvents along with the nucleophile source (TMSCN), can play a crucial role in the incorporation of nitriles to the ketimines (Table S2). The reaction is unsuccessful with toluene alone but successful with the combination of toluene and MeOH in 2:1 ratio (0.1 M), suggesting that MeOH is crucial for the

generation of HCN from TMSCN. Pleasingly, almost comparable results were observed (entries 2-3) with MeOH (0.1 M) alone. For the sake of convenience and homogeneity, we decided to use MeOH (0.1 M) as solvent for the subsequent transformations.

Table S2:

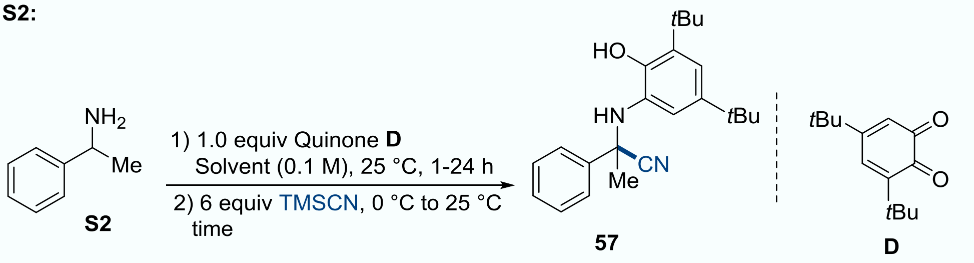

entry	solvent	time/h	products (%)
1	PhMe	4	n.r[a]
2	PhMe/MeOH (2:1)	24	**57** (96)
3	MeOH	24	**57** (97)

[a] Imine/hemiaminal was intacted after 4 h at 25 °C.

2.3: Optimization for oxidative cleavage of phenol unit:

Table S3:

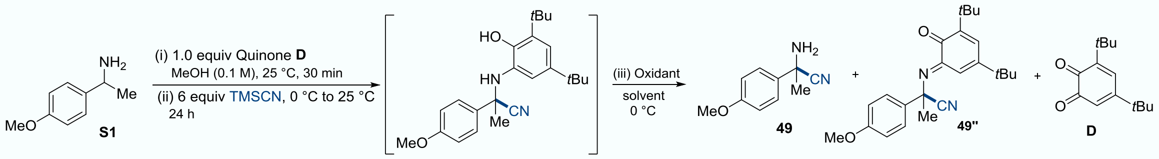

entry	oxidant	solvent	time	product yields 49	49''	D
1	I_2 (2 equiv)/ aq. NaOH (5 equiv)	CH_3CN	24 h	---	99%	---
2	CAN (4 equiv)	$CH_3CN:H_2O$ (1:1)	15 min	---	---	72%
3	$PhI(OAc)_2$ (2 equiv)	$CH_3CN:H_2O$ (2:1)	15 min	---	82%	10%
4	$PhI(OTFA)_2$ (2 equiv)	$CH_3CN:H_2O$ (2:1)	15 min	---	---	85%
5	PhI=O (2 equiv)	$CH_3CN:H_2O$ (2:1)	15 min	---	95%	---
6	O_2 ballon/ aq. NaOH (5 equiv)	CH_3CN	24 h	---	65%	12%
7	H_5IO_6 (4 equiv)/ Conc. H_2SO_4	$CH_3CN:H_2O$ (1:1)	10 min	84%	---	92%
**8**	**H_5IO_6 (1.05 equiv)**	**$CH_3CN:H_2O$ (1:1)**	**10 min**	**86%**	**---**	**96%**

The initial oxidative conditions (I_2/NaOH) were unsuccessful and gave only the undesired imine **49''** (Table S3, entry 1). Further hydrolysis of **49''** with either aqueous NaOH or aqueous HCl gave complex mixture of products. After numerous attempts by screening a variety of oxidants, we were pleased to find that orthoperiodic acid (H_5IO_6) proved to be more efficient and cleaved the phenolic unit completely from the cyanoaddition product (entry 8).

3: General Procedures:

3.1: Synthesis of *o*-quinones:

3.1.1: Synthesis of 4-(*tert*-butyl)-*o*-benzoquinone (A):

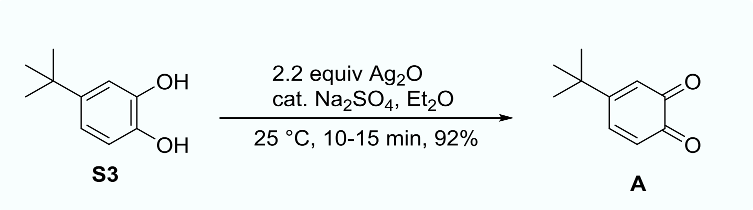

The compound **A** was synthesised using the known literature method with slight modifications. To an ether (30 mL) solution of 4-tert-butylcatechol **S3** (0.50 g, 2.57 mmol) and Na_2SO_4 (0.1 g) was added Ag_2O (3.10 g, 13.4 mmol) at 25 ˚C and stirred the resulting heterogeneous mixture for 10-15 min. After completion as indicated by TLC, the ether layer was collected by filtration. The combined ether layer was dried by Na_2SO_4 and concentrated to give 4-(*tert*-butyl)-*o*-benzoquinone **A** as a red solid (0.91 g, 5.60 mmol, 92%). **^{1}H NMR** (400 MHz, $CDCl_3$): δ 7.18 (dd, *J* = 10.4, 2.4 Hz, 1 H), 6.40 – 6.31 (m, 1 H), 6.24 (dd, *J* = 2.2, 1.2 Hz, 1 H), 1.22 – 1.20 (m, 9 H). **^{13}C NMR** (101 MHz, $CDCl_3$): δ 180.4, 180.3, 162.2, 140.2, 129.5, 123.9, 35.7, 27.9. The remaining data is consistent with the literature precedent.[1]

3.1.2: Synthesis of 4-(*tert*-butyl)-5-methoxy-*o*-benzoquinone (B):

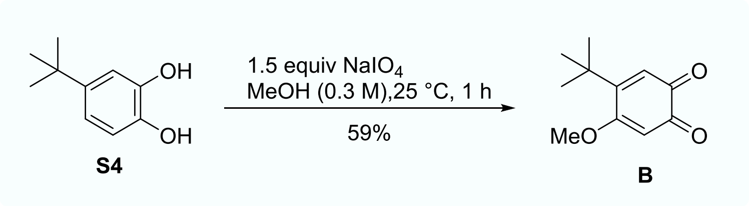

The compound **B** was synthesised using the known literature method with slight modifications. 4-(*tert*-butyl)benzene-1,2-diol **S4** (1.00 g, 6.02 mmol) was dissolved in a 100 mL round bottom flask using 20 mL of methanol. To this, $NaIO_4$ (1.93 g, 9.02 mmol) was added at 25 ˚C and stirred for 1 h. After completion as indicated by TLC, water (50 mL) was added and the resulting solution was extracted with ether (100 mL), dried (Na_2SO_4) and evaporated under rotary evaporator. The crude dark red sticky material was triturated with *n*-pentane to give a red solid of 4-(*tert*-butyl)-5-methoxy-*o*-benzoquinone **B** (0.69 g, 3.55 mmol, 59%). The data is consistent with the literature precedent.[1]

3.1.3: Synthesis of 3,5-diisopropyl-*o*-benzoquinone (C):

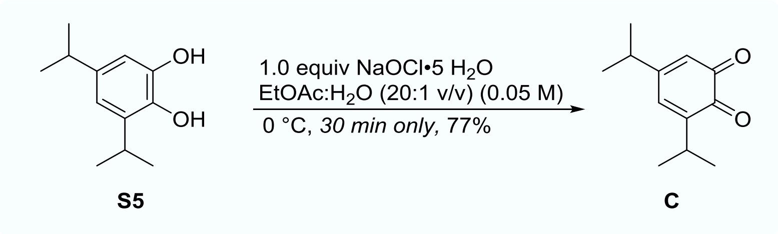

The compound **C** was synthesised using the known literature method with slight modifications. 3,5-diisopropylcatechol **S5** (0.50 g, 2.57 mmol) was dissolved in a 100 mL round bottom flask using 49 mL of EtOAc and 2.5 mL of water (20:1, 0.05 M) and the resulting biphasic solution was cooled to 0 ˚C before the addition of bleach ($NaOCl·5H_2O$) (3.02 mL, 2.57 mmol, 6-14% active Cl_2). The resulting heterogeneous green solution was stirred for exactly 30 minutes at 0 ˚C. After this time, the crude product was taken into separating funnel and organic phase was separated, washed with brine and dried (Na_2SO_4), filtered, and concentrated under reduced pressure. The crude product was recrystallized using pentane gave a deep brown solid of compound **C** (380 mg, 1.98 mmol, 77%). **^{1}H NMR** (400 MHz, $CDCl_3$): δ 6.62 (d, *J* = 0.9 Hz, 1 H), 6.11 (d, *J* = 0.9 Hz, 1 H), 2.97 (m, 1 H), 2.55 (m, 1 H), 1.19 (d, *J* = 6.8 Hz, 6 H), 1.12 (d, *J* = 6.8 Hz, 6 H). **^{13}C NMR** (101 MHz, $CDCl_3$): δ 180.8, 180.4, 161.8, 149.1, 134.6, 122.2, 34.9, 27.5, 21.7, 20.6. The remaining data was consistent with the literature.[2]

3.1.4: Synthesis of 3,5-di-*tert*-butyl-*o*-benzoquinone (D):

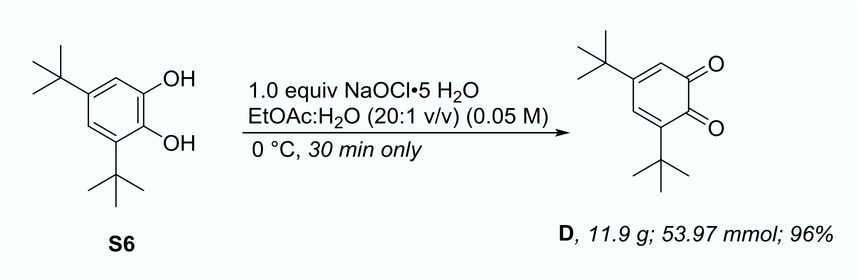

The compound **D** was synthesised using the known literature method with slight modifications. 3,5-di-*tert*-butylcatechol **S6** (12.5 g, 56.3 mmol) was dissolved in a 2 L round bottom flask using 1.07 L of EtOAc and 53.6 mL of water (20:1, 0.05 M) and the resulting biphasic solution was cooled to 0 ˚C before the addition of bleach ($NaOCl·5H_2O$) (66.0 mL, 56.2 mmol, 6-14% active Cl_2). The resulting heterogeneous green solution was stirred for exactly 30 minutes at 0 ˚C. After this time, the crude product was taken into separating funnel and organic phase was separated, washed with brine and dried (Na_2SO_4), filtered, and concentrated under reduced pressure. The crude product was recrystallized using pentane gave a deep red crystals of compound **D** (11.9 g, 53.97 mmol, 96%). **^{1}H NMR** (400 MHz, $CDCl_3$): δ 6.92 (d, *J* = 2.3 Hz, 1 H), 6.20 (d, *J* = 2.3 Hz, 1 H), 1.26 (s, 9 H), 1.21 (s, 9 H).

^{13}C NMR (101 MHz, $CDCl_3$): δ 181.3, 180.2, 163.4, 150.1, 133.6, 122.2, 36.2, 35.6, 29.3, 28.0. The remaining data was consistent with the literature.[2]

3.2: General Procedure A for α-C–H Functionalization of primary amines with Grignard reagent:

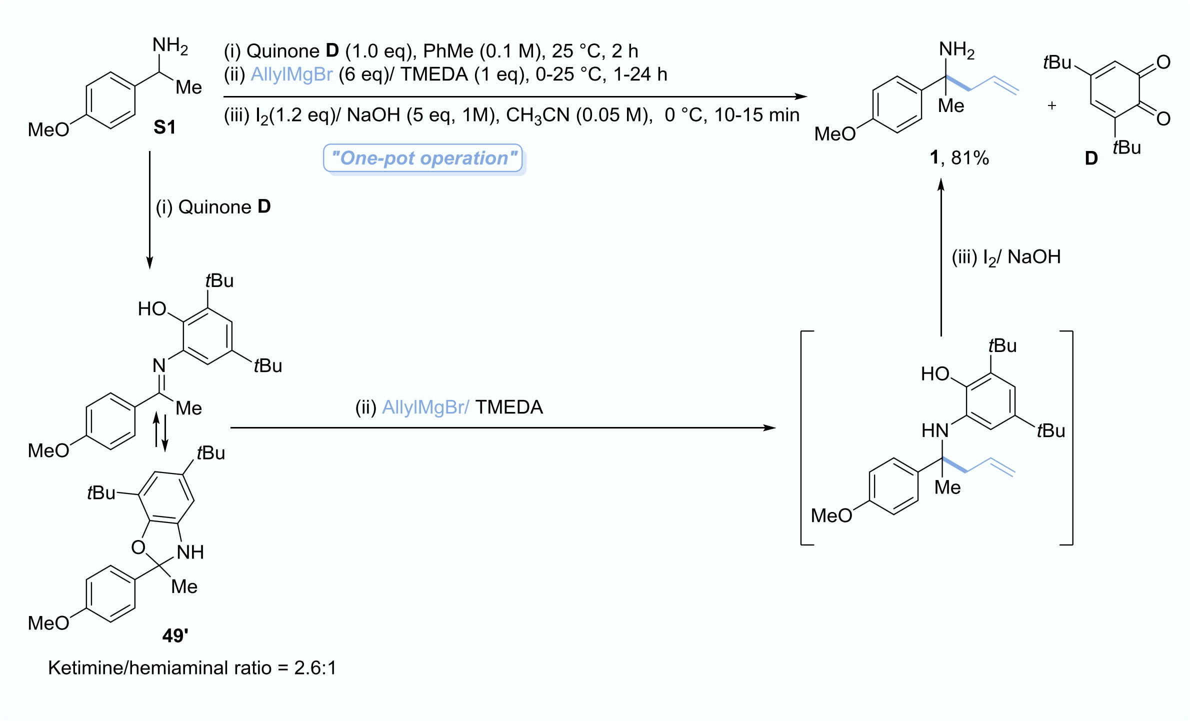

The synthesis of 2-(4-methoxyphenyl)pent-4-en-2-amine **1** is representative. To a stirred solution of 3,5-di-*tert*-butyl-*o*-benzoquinone **D** (0.29 g, 1.32 mmol, 1.0 equiv) in toluene (6.0 mL), was added a solution of 1-(4-methoxyphenyl)ethan-1-amine **S1** (0.20 g, 1.32 mmol, 1.0 equiv) in toluene (7.2 mL) dropwise over 5 min under argon atmosphere. The deep green coloured solution was stirred at room temperature for 2 h. After completion as indicated by TLC *(Note: colour changes from deep green to dark violet),* the reaction mixture was cooled to 0 ˚C, and was added TMEDA (0.19 mL, 1.32 mmol, 1.0 equiv) and allylmagnesium bromide (1.0 M in Et_2O, 7.94 mL, 7.94 mmol, 6.0 equiv) and maintained at 0˚C for 1 h. During the Grignard addition, the colour of the reaction mixture changes from dark violet to yellowish brown. The reaction temperature was gradually allowed to return to 25 ˚C and allowed to stir until TLC analysis (A small aliquot of the reaction mixture was worked up in NH_4Cl-EtOAc to check TLC (Pentane:EtOAc 9.5:0.5)) showed complete conversion. After completion of the reaction as indicated by TLC, the reaction mixture was again cooled to 0 ˚C and aqueous NaOH (1 M) (6.62 mL, 6.62 mmol, 5.0 equiv) was carefully added dropwise to quench the remaining Grignard reagent. To the resulting heterogeneous mixture were added 26 mL of CH_3CN (0.05 M) and iodine granules (0.40 g, 1.59 mmol, 1.2 equiv) and stirred vigorously for 10 min under argon

atmosphere. The *o*-quinone reappeared gradually during basic oxidation. After completion, reaction mixture was extracted with CH_3CN (3 x 20 mL). The combined organic layers were washed with aqueous saturated sodium thiosulfate (1 x 10 mL) and brine (2 x 10 mL), respectively, dried over Na_2SO_4, filtered, and concentrated *in vacuo*. The crude residue was purified by column chromatography on silica gel eluting with Pentane:EtOAc (80:20 v:v) to EtOAc:MeOH:Et_3N (70:20:10 v:v) to afford compound **1** (205 mg, 1.07 mmol, 81%) as a yellow oil.

3.3: General Procedure B for α-C–H Functionalization of primary amines with Grignard reagent:

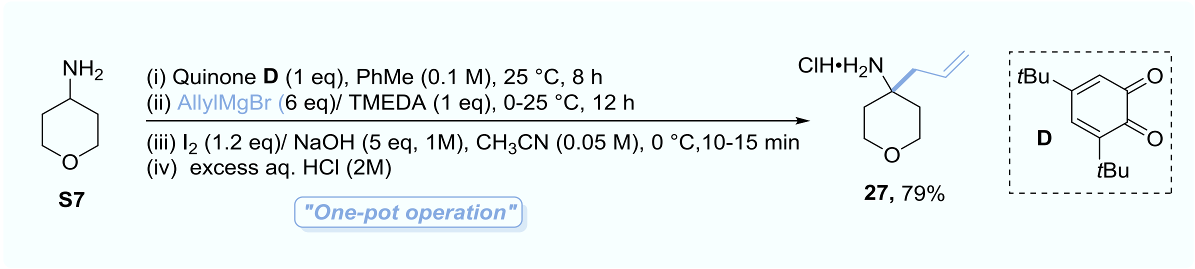

The synthesis of 4-allyltetrahydro-2*H*-pyran-4-amine hydrochloride **27** is representative. For volatile, low boiling point amines, a modified work-up was employed. After oxidative cleavage of the *o*-quinone, the reaction mixture was extracted with CH_3CN (3 x 20 mL). The combined organic layers were washed with saturated sodium thiosulfate (1 x 10 mL), brine (2 x 10 mL) respectively. The organic layer was acidified with aqueous 2 M HCl and extracted two times (2 x 20 mL) with aqueous 2 M HCl. The combined aqueous phases were washed with hexane and concentrated *in vacuo* (Bath temp: 60 ˚C, 72 mbar) to afford the crude yellow solid, which was dissolved in warm $CHCl_3$, dried over Na_2SO_4, filtered, and concentrated under reduced pressure to give 4-allyltetrahydro-2*H*-pyran-4-amine hydrochloride **27** (139 mg, 0.78 mmol, 79%) as off-white powder.

3.4 General Procedure C for α-C–H Functionalization of primary amines with organolithium reagents:

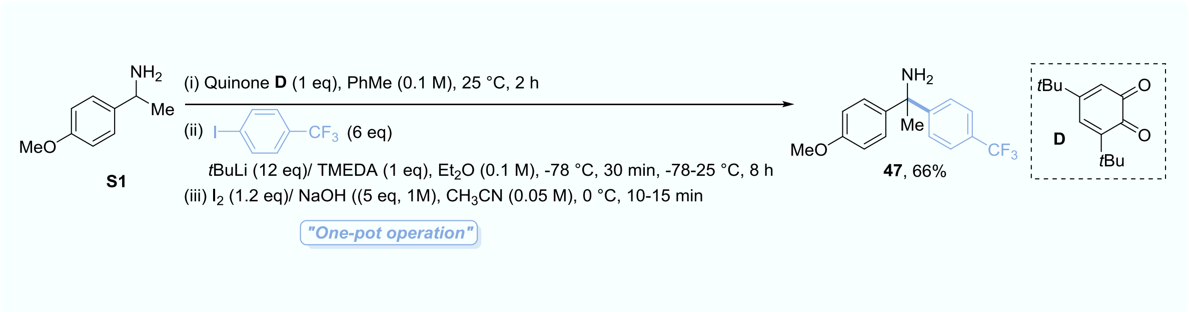

The synthesis of 1-(4-methoxyphenyl)-1-(4-(trifluoromethyl)phenyl)ethan-1-amine **47** is representative. To a stirred solution of 3,5-di-*tert*-butyl-*o*-benzoquinone **D** (0.15 g, 0.66 mmol, 1.0 equiv) in toluene (3.3 mL), was added a solution of 1-(4-methoxyphenyl)ethan-1-amine **S1** (0.10 g, 0.66 mmol, 1.0 equiv) in toluene (3.3 mL) dropwise over 5 min under argon atmosphere. The deep green coloured solution was stirred at room temperature for 2 h. In the meantime, the organolithium was prepared by treating 1-iodo-4-(trifluoromethyl)benzene (0.58 mL, 3.97 mmol, 6 equiv) with *t*BuLi (4.67 mL, 7.94 mmol, 1.7 M in pentane, 12 equiv) at -78 ˚C for 30 min in dry Et_2O (0.1 M) under argon atmosphere.[4] After completion of the ketimine/hemiaminal formation as indicated by TLC *(Note: colour changes from deep green to dark violet)*, the reaction mixture was treated with TMEDA (94 µL, 0.66 mmol, 1.0 equiv) and transferred through syringe to the flask containing (4-(trifluoromethyl)phenyl)lithium at -78 ˚C, and the resulting mixture stirred for 8 h at 25 ˚C. A small aliquot of the reaction mixture was worked up in NH_4Cl-EtOAc to check TLC (Pentane:EtOAc 9.5:0.5). After completion of the reaction as indicated by TLC, the reaction mixture was cooled to 0 ˚C and aqueous NaOH (1 M) (3.31 mL, 3.31 mmol, 5.0 equiv) was added dropwise to quench the remaining organolithium reagent. To this mixture were added 13 mL of CH_3CN and iodine granules (0.20 g, 0.79 mmol, 1.2 equiv) and the mixture stirred vigorously for 10 min under argon atmosphere. The *o*-quinone reappeared gradually during basic oxidation. After completion, the reaction mixture was extracted with CH_3CN (3 x 20 mL). The combined organic layers were washed with aqueous saturated sodium thiosulfate (1 x 10 mL) and brine (2 x 10 mL), dried over Na_2SO_4, filtered, and concentrated under reduced pressure. The crude residue was purified by column chromatography on silica gel eluting with Pentane:EtOAc (80:20 v:v) to EtOAc:MeOH:Et_3N (70:20:10 v:v) to afford compound **47** (129 mg, 0.44 mmol, 66%) as a brown oil.

3.5 General Procedure D for oxidative cyanation of primary amines:

The synthesis of 4-aminotetrahydro-2*H*-pyran-4-carbonitrile **54** is representative.[7]

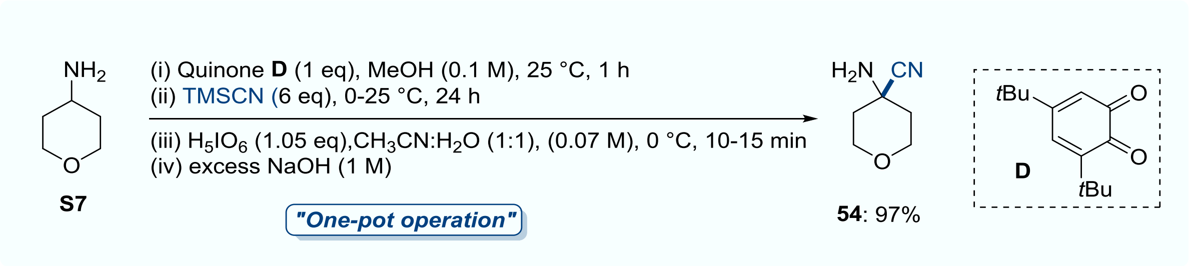

To a stirred solution of 3,5-di-*tert*-butyl-*o*-benzoquinone **D** (0.22 g, 0.99 mmol, 1.0 equiv) in MeOH (5.0 mL), was added slowly a solution of 4-aminotetrahydropyran **S7** (0.10 g, 0.99 mmol, 1.0 equiv) in MeOH (5 mL) dropwise over 5 min and the resulting mixture was stirred for 1 h at 25 ˚C. After

completion as indicated by TLC, the reaction mixture was then cooled to 0˚C and trimethylsilyl cyanide (0.74 mL, 5.93 mmol, 6.0 equiv) was added. The resulting mixture was gradually allowed to return to 25 ˚C and allowed to stir until TLC analysis showed complete conversion. After completion as indicated by TLC, solvent and excess trimethylsilyl cyanide were removed on a rotary evaporator using bath temperature below 25 ˚C (*Caution: high bath temperature causes decomposition of addition product).* The resulting grey solid was dissolved in CH_3CN:H_2O (1:1) (14.1 mL), and cooled to 0 ˚C. To the resulting mixture was added orthoperiodic acid (H_5IO_6) (0.24 g, 1.04 mmol, 1.05 equiv) and stirred vigorously for 10 min (appearance of brown colour denotes reformation of quinone). After completion, the organic solvent was removed under reduced pressure (Bath temp: 40 ˚C) and the resulting aqueous acidic phase was washed with Et_2O:Hexane (10:1) (2 x 10 mL), and the aqueous phase was then concentrated *in vacuo* (Bath temp: 60 ˚C, 72 mbar). The resulting residue was basified with 1 M NaOH (5 mL) and extracted with $CHCl_3$ (3 x 10 mL). The combined organic layers were washed with brine, dried over Na_2SO_4, filtered and concentrated *in vacuo* to afford the 4-aminotetrahydro-2*H*-pyran-4-carbonitrile **54** as a yellow oil (121 mg, 0.96 mmol, 97%). In most cases (**48, 51, 52, 53, 55, 56, 66, 68**), the obtained crude products were pure, and no further purification was performed. Compounds **49** and **50** were purified by column chromatography on Et_3N-treated silica gel eluting with (pentane/EtOAc = 80:20 v:v) to (EtOAc:MeOH:Et_3N = 70:20:10 v:v).

3.6: General Procedure E for the photocatalytic reverse polarity α-allylation:

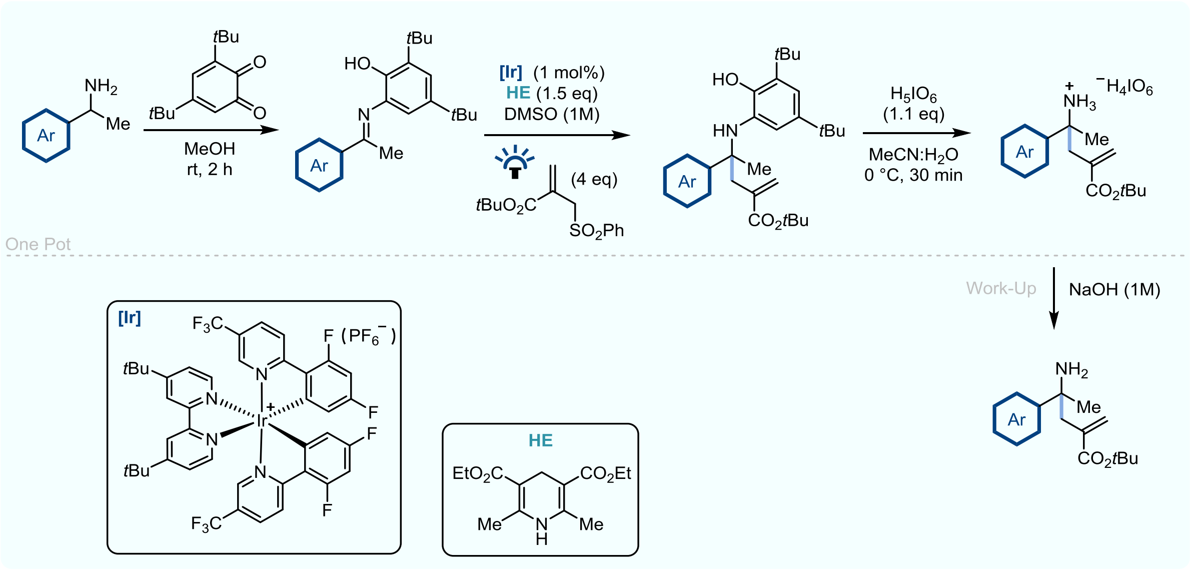

To a mass spec vial under a stream of N_2 was equipped a micro-stirrer charged 3,5-di-*tert*-butyl-*o*-benzoquinone **D** (55.0 mg, 0.25 mmol), anhydrous MeOH (0.50 mL), and relevant benzylamine structure (0.25 mmol). The nitrogen line was removed and the reaction mixture allowed to stir for 2

h. The solvent was then removed under a nitrogen stream. To the residue was added anhydrous DMSO (0.25 mL), followed by (Ir[(dFCF$_3$ppy)$_2$(dtbbpy)]PF$_6$ ([Ir], 2.8 mg, 0.0025 mmol), diethyl 1,4-dihydro-2,6-dimethyl-3,5-pyridinedicarboxylate (HE, 95.0 mg, 0.375 mmol) and *tert*-butyl 2-((phenylsulfonyl)methyl)acrylate (282 mg, 1.00 mmol). The reaction mixture was then degassed with N_2 for 10 min. The flask was sealed and allowed to stir for 20 h under blue light irradiation. After this time the reaction mixture was cooled to 0 °C, and MeCN (0.50 mL), water (0.25 mL) and periodic acid (63.0 mg, 0.28 mmol) were added. The resulting mixture went instantly deep brown to signal reformation of 3,5-di-*tert*-butyl-*o*-benzoquinone and was then allowed to stir at 0 °C for 30 min. The reaction mixture was poured into a separating funnel containing water (5 mL) and Et_2O (10 mL). The aqueous phase was extracted, and the organic phase re-extracted with water (4 x 5 mL). To the combined aqueous phases was added NaOH (1M, 10 mL). The aqueous phase was then extracted with EtOAc:MeCN (1:1, 5 x 10 mL). The combined organics were then washed with NaOH (1M, 5 x 5 mL) and the organics were dried over $MgSO_4$ and concentrated *in vacuo*. The crude residue was then purified *via* silica gel column chromatography to give the desired primary amine product.

3.7: Procedure for Alternative Protocol (Scheme 6A)

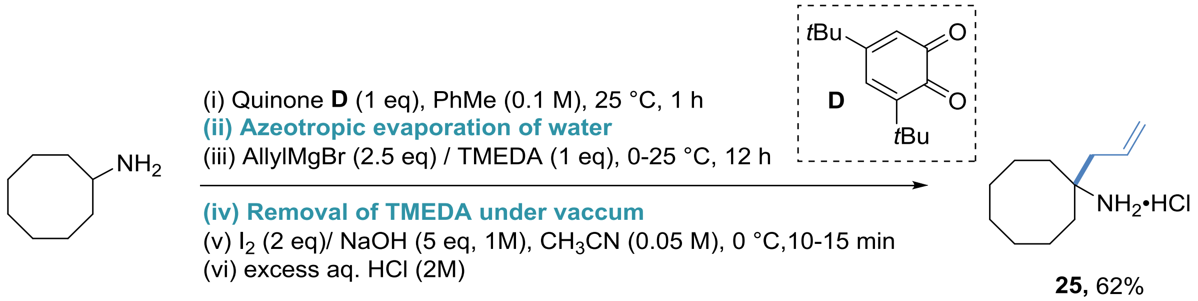

25, 62%

To a stirred solution of 3,5-di-*tert*-butyl-*o*-benzoquinone **D** (1.75 g, 7.94 mmol, 1.01 equiv) in toluene (40 mL), was added a solution of 1-(4-methoxyphenyl)ethan-1-amine **S1** (1.00 g, 7.86 mmol, 1.0 equiv) in toluene (40 mL) dropwise over 5 min under argon atmosphere. After completion as indicated by TLC *(Note: colour changes from deep green to dark violet),* the reaction mixture was concentrated *in vacuo* (150 – 5 mbar, 40 °C) to remove water. To the crude residue was added toluene (80 mL) and 4Å molecular sieves (and the reaction mixture was cooled to 0 ˚C. To the cooled solution was added TMEDA (1.18 mL, 7.86 mmol, 1.0 equiv) and allylmagnesium bromide (1.0 M in Et_2O, 19.63 mL, 19.63 mmol, 2.5 equiv) and maintained at 0˚C for 30 mins. The reaction temperature was gradually allowed to return to 25 ˚C and allowed to stir until TLC analysis showed complete conversion. concentrated *in vacuo* (80 - 40 mbar, 60 °C, then high vacuum) to remove the TMEDA. (to assist product isolation post-oxidation). The crude residue was again cooled to 0 ˚C and aqueous NaOH (1 M) (6.62 mL, 6.62 mmol, 5.0 equiv) was carefully added dropwise to quench the remaining

Grignard reagent. To the resulting heterogeneous mixture were added 40 mL of CH_3CN (0.05 M) and iodine granules (3.99 g, 39.3 mmol, 2 equiv) and stirred vigorously for 10 min under argon atmosphere. The *o*-quinone reappeared gradually during basic oxidation. After completion, the reaction mixture was extracted with CH_3CN (3 x 80 mL). The combined organic layers were washed with saturated sodium thiosulfate (1 x 80 mL), brine (2 x 80 mL) respectively. The organic layer was acidified with aqueous 2 M HCl and extracted two times (2 x 80 mL) with aqueous 2 M HCl. The combined aqueous phases were washed with hexane and concentrated *in vacuo* (Bath temp: 60 ˚C, 72 mbar) to afford **25** as the hydrochloride salt, (993 mg, 4.87 mmol, 62%).

3.6.1: Photoreactor details:

Hepatochem PhotoRedOx Box, equipped with an EvoluChem LED 18 W light source supplied by Hepatochem. A cardboard cover was also placed over the reactor during reactions. Capable of carrying out up to 8 reactions at one time. Accurate and reproducible results, high throughput.

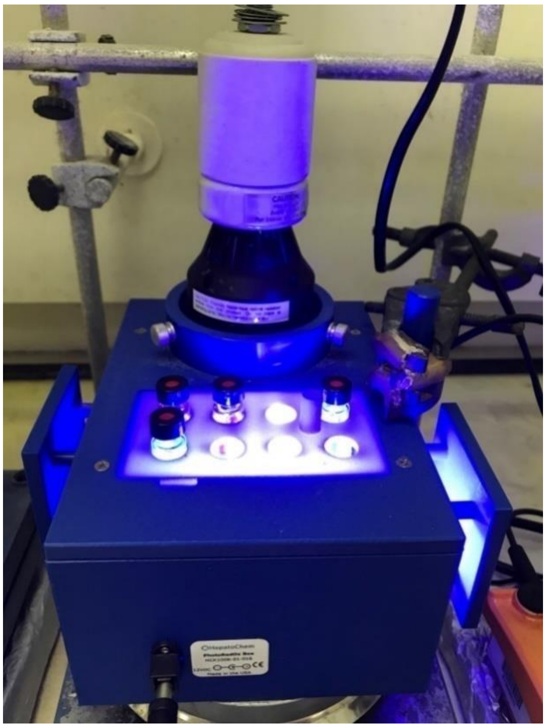

3.6.2: Coupling partner synthesis:

Synthesis of *tert*-butyl 2-((phenylsulfonyl)methyl)acrylate:

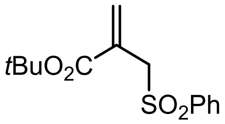

To a 1 L round-bottomed flask containing *tert*-butyl acrylate (11.7 mL, 80.0 mmol) and formaldehyde (3.60 g, 120 mmol) in 1,4-dioxane (80 mL) and water (80 mL) was added 1,4-diazabicyclo[2.2.2]octane (11.6 g, 104 mmol) portionwise. The reaction mixture was allowed to stir at room temperature for 16 h. The resulting mixture was partitioned between EtOAc (200 mL) and water (200 mL). The organic layer was extracted and then washed with brine (300 mL). The organics were then dried over $MgSO_4$ and concentrated *in vacuo*. The crude residue was then dispersed in anhydrous Et_2O (300 mL) and the flask cooled to -10 °C. To the resulting mixture was added phosphorus tribromide (4.05 mL, 43.0 mmol) dropwise. The flask was allowed to warm to room temperature and stirred for 4 h. The flask was quenched by dropwise addition of water (200 mL). The resulting suspension was poured into a separating funnel and organic layer extracted. The aqueous phase was then re-extracted with pentane (2 x 200 mL). The combined organics were washed with brine, dried over $MgSO_4$ and concentrated *in vacuo*. The crude residue was dissolved in MeOH (300 mL) and was added sodium benzenesulfinate (5.50 g, 33.5 mmol) and the reaction mixture was heated to reflux for 16 h. The flask was allowed to cool for rt and then the MeOH was removed *in vacuo*. The crude residue was partitioned between EtOAc (200 mL) and water (200 mL). The organic layer was extracted and then washed with brine (300 mL), dried over $MgSO_4$ and concentrated *in vacuo*. The crude residue was purified *via* silica gel column chromatography (EtOAc:Pentane 3:7 v:v) to give the above compound as a white crystalline solid (4.90 g, 17.4 mmol, 22%). **^{1}H NMR** (400 MHz, $CDCl_3$): δ 7.88 – 7.78 (m, 2 H), 7.68 – 7.57 (m, 1 H), 7.57 – 7.43 (m, 2 H), 6.43 (d, *J* = 0.9 Hz, 1 H), 5.89 (d, *J* = 0.9 Hz, 1 H), 4.13 (d, *J* = 0.8 Hz, 2 H), 1.31 (s, 9 H). **^{13}C NMR** (101 MHz, $CDCl_3$): δ 163.8, 138.6, 133.9, 132.9, 130.4, 129.2, 129.0, 81.9, 57.5, 27.9. Data is in line with literature precedent.[8]

Synthesis of ethyl 2-((phenylsulfonyl)methyl)acrylate:

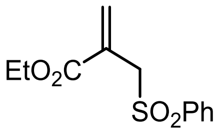

To a 1 L round-bottomed flask containing ethyl acrylate (21.8 mL, 200 mmol) and formaldehyde (9.00 g, 300 mmol) in 1,4-dioxane (200 mL) and water (200 mL) was added 1,4-diazabicyclo[2.2.2]octane (29.1 g, 260 mmol) portionwise. The reaction mixture was allowed to stir at room temperature for 16 h. The resulting mixture was partitioned between EtOAc (300 mL) and water (300 mL). The organic layer was extracted and then washed with brine (300 mL). The organics were then dried over $MgSO_4$ and concentrated *in vacuo*. The crude residue was then dispersed in anhydrous Et_2O (300 mL) and the flask cooled to -10 °C. To the resulting mixture was added phosphorus tribromide (13.8 mL, 134 mmol) dropwise. The flask was allowed to warm to room temperature and stirred for 4 h. The flask was quenched by dropwise addition of water (300 mL). The resulting suspension was poured into a separating funnel and organic layer extracted. The aqueous phase was then re-extracted with pentane (2 x 300 mL). The combined organics were washed with brine, dried over $MgSO_4$ and concentrated *in vacuo*. The crude residue was dissolved in MeOH (300 mL) and was added sodium benzenesulfinate (19.4 g, 119 mmol) and the reaction mixture was heated to reflux for 16 h. The flask was allowed to cool for rt and then the MeOH was removed *in vacuo*. The crude residue was partitioned between EtOAc (300 mL) and water (300 mL). The organic layer was extracted and then washed with brine (300 mL), dried over $MgSO_4$ and concentrated *in vacuo*. The crude residue was purified *via* silica gel column chromatography (Et_2O:Pentane 3:7 v:v) to give the above compound as a thick colourless oil (19.1 g, 75.1 mmol, 38%). **^{1}H NMR** (400 MHz, $CDCl_3$): δ 7.84 (dd, *J* = 8.3, 1.3 Hz, 2 H), 7.70 – 7.59 (m, 1 H), 7.59 – 7.45 (m, 2 H), 6.49 (s, 1 H), 5.90 (d, *J* = 1.0 Hz, 1 H), 4.15 (d, *J* = 0.8 Hz, 2 H), 3.99 (q, *J* = 7.1 Hz, 2 H), 1.15 (t, *J* = 7.1 Hz, 3 H). **^{13}C NMR** (101 MHz, $CDCl_3$): δ 164.8, 138.5, 133.9, 133.4, 129.2, 129.1, 128.8, 61.5, 57.6, 14.1. Data is in line with literature precedent.[9]

3.6.3: Catalyst Synthesis:

Synthesis of 2-(2,4-difluorophenyl)-5-(trifluoromethyl)pyridine:

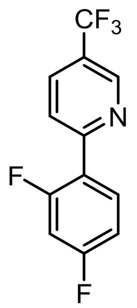

To a three-necked 250 mL round bottomed flask was charged with 2,4-difluorophenylboronic acid (5.68 g, 36.0 mmol), 2-bromo-5-trifluoromethylpyridine (6.78 g, 30.0 mmol), potassium carbonate (12.4 g, 90.0 mmol), palladium acetate (202 mg, 0.90 mmol) and triphenylphosphine (472 mg, 1.80

mmol). The flask was equipped with a condenser then evacuated and refilled with N_2 three times. Following this, toluene (40 mL), water (40 mL) and ethanol (8 mL) were added *via* septum. The flask was heated to reflux for 16 h. After this time, the flask was cooled to room temperature and quenched with water (100 mL). The organic phase was separated and then the aqueous phase re-extracted with Et_2O (3 x 200 mL). The combined organics were washed with brine (3 x 200 mL) and then dried over $MgSO_4$ and concentrated *in vacuo*. The crude residue was the then purified *via* silica gel column chromatography (EtOAc:Pentane 1:99 – 4:96 v:v) to give the title compound as a white solid (6.62 g, 25.5 mmol, 85%). **^{1}H NMR** (400 MHz, $CDCl_3$): δ 8.85 (dd, *J* = 2.4, 1.2 Hz, 1 H), 7.99 (td, *J* = 8.9, 6.6 Hz, 1 H), 7.87 (dd, *J* = 8.3, 2.4 Hz, 1 H), 7.82 – 7.76 (m, 1 H), 6.98 – 6.88 (m, 1 H), 6.82 (ddd, *J* = 11.3, 8.7, 2.5 Hz, 1 H). **^{19}F NMR** (377 MHz, $CDCl_3$): δ -62.49, -107.28 (t, *J* = 8.0 Hz), -112.04 (d, *J* = 10.0 Hz). **^{13}C NMR** (101 MHz, $CDCl_3$): δ 164.0 (dd, *J* = 252.8, 12.5 Hz), 161.1 (dd, *J* = 253.6, 12.0 Hz), 155.9, 146.7 (q, *J* = 4.2 Hz), 133.8 (q, *J* = 3.5 Hz), 132.6 (dd, *J* = 10.0, 4.2 Hz), 125.3 (q, *J* = 33.2 Hz), 123.7 (q, *J* = 272.3 Hz), 123.7 (d, *J* = 11.1 Hz), 122.5 (dd, *J* = 11.3, 3.8 Hz), 112.4 (dd, *J* = 21.1, 3.6 Hz), 106.5 – 101.0 (app t). Data was consistent with literature precedent.[10]

Synthesis of $[Ir(dF(CF_3)ppy)_2Cl]_2$:

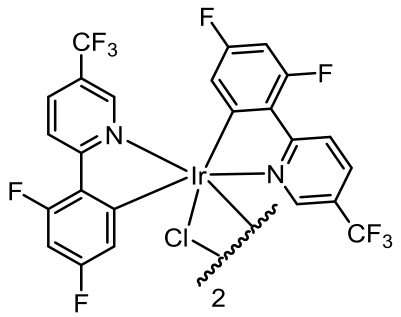

To a three-necked 100 mL round bottomed flask was charged iridium(III) chloride hydrate (448 mg, 1.50 mmol) and 2-(2,4-difluorophenyl)-5-(trifluoromethyl)pyridine (856 mg, 3.30 mmol). The flask was equipped with a condenser, then evacuated and refilled with nitrogen three times. Rigorously degassed 2-ethoxyehtanol (18 mL) and water (6 mL) were added *via* syringe. The reaction mixture was heated 150 °C for 16 h. After this time the reaction mixture was allowed to return to room temperature and the bright yellow precipitate formed was filtered under a blanket of N_2, washing with water (150 mL) and then hexane (60 mL), to give title compound after further removal of water *via* high vacuum (960 mg, 0.65 mmol, 86%). **^{1}H NMR** (400 MHz, $CDCl_3$): δ 9.51 (d, *J* = 2.1 Hz, 1 H), 8.46 (dd, *J* = 8.7, 3.0 Hz, 1 H), 8.05 (dd, *J* = 8.7, 2.3 Hz, 1 H), 6.43 (ddd, *J* = 12.5, 8.8, 2.3 Hz, 1 H), 5.07 (dd, *J* = 8.8, 2.3 Hz, 1 H). **^{19}F NMR** (377 MHz, $CDCl_3$): δ -62.36 (12 F), -103.41 – -103.72 (m, 4 F), -106.97 – -107.91 (m, 4 F). Data was consistent with literature precedent.[10]

Synthesis of (Ir[dF(CF_3)ppy)$_2$](dtbpy))PF_6 – [Ir]:

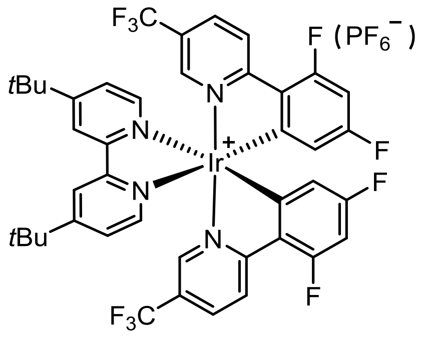

To a three-necked 250 mL round bottomed flask was charged [Ir(dF(CF_3)ppy)$_2$Cl]$_2$ (960 mg, 0.65 mmol) and 4,4'-di-*tert*-butyl-2,2'-dipyridyl (429 mg, 1.60 mmol). The flask was equipped with a reflux condenser, then evacuated and refilled three times with nitrogen. Rigorously degassed ethylene glycol (44 mL) was then added *via* syringe. The reaction mixture was then heated to 150 °C for 16 h. After this time the flask was allowed to return to room temperature. The mixture was diluted in water (300 mL) and hexane (300 mL). The aqueous phase was then separated and then re-extracted with hexane (2 x 300 mL). The aqueous phase was then decanted into a 500 mL conical flask and equipped with a stirrer bar. The flask was heated at 80 °C for 1 hour to remove residual hexane. The flask was allowed to return to room temperature, and an aqueous solution of potassium hexafluorophosphate (7 g in 70 mL water) was added with stirring, and a vibrant yellow precipitate was formed. The mixture was then allowed to stand at 5 °C for 1 hour, before the precipitate was collected *via* vacuum filtration washing with water (150 mL) and hexane (100 mL), The collected powdery solid was then subjected to further water removal *via* high vacuum, to give the title compound, (915 mg, 0.82 mmol, 63%). **^{1}H NMR** (400 MHz, $CDCl_3$): δ 8.88 (d, *J* = 2.1 Hz, 2 H), 8.47 (dd, *J* = 8.7, 3.1 Hz, 2 H), 8.04 (dd, *J* = 8.8, 2.1 Hz, 2 H), 7.86 (d, *J* = 5.9 Hz, 2 H), 7.59 (dd, *J* = 5.9, 1.9 Hz, 2 H), 7.41 (s, 2 H), 6.64 (ddd, *J* = 11.6, 8.9, 2.3 Hz, 2 H), 5.63 (dd, *J* = 8.0, 2.4 Hz, 2 H), 1.50 (s, 18 H). **^{19}F NMR** (377 MHz, $CDCl_3$): δ -62.99 (6 F), -72.24 (3 F), -74.13 (3 F), -101.81 (dt, *J* = 12.4, 8.4 Hz, 2 F), -105.92 (td, *J* = 12.4, 3.4 Hz, 2 F). NMR Spectra matched those from commercial sources.

4: Demonstration of stepwise procedure for one-pot operation:

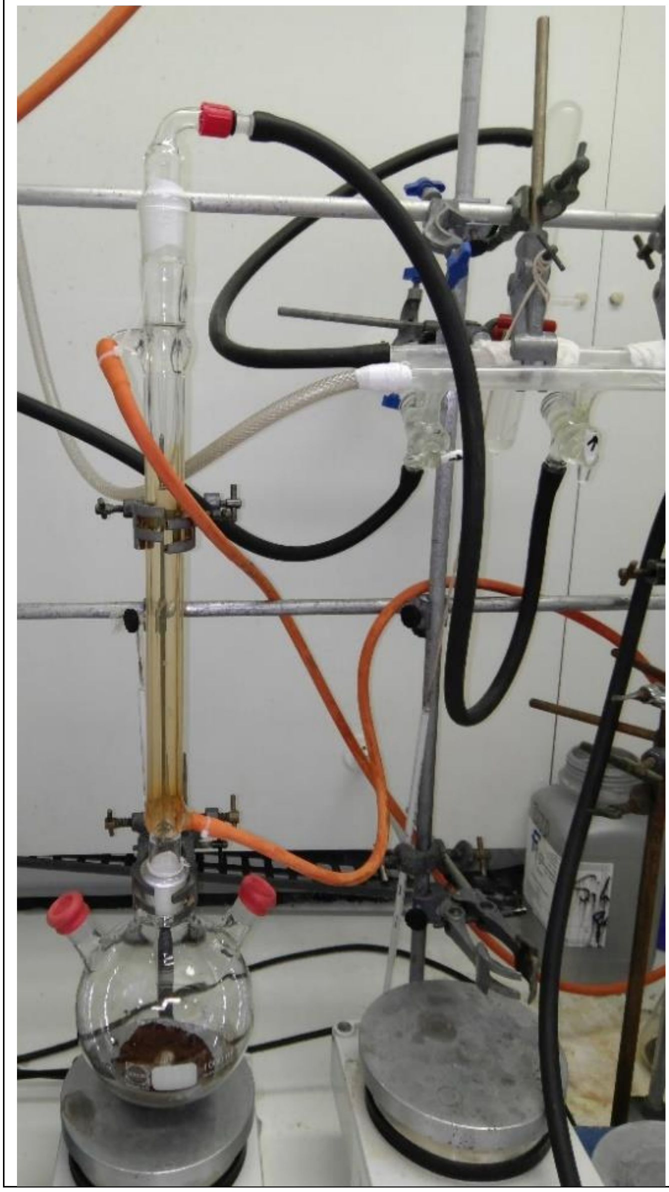	**Reaction setup:** ***Step 1: (ketimine/hemiaminal formation):*** *o*-quinone (6.60 g, 30.0 mmol) was placed in a 3 neck round bottom flask (1000 ml), which was connected to a reflux condenser and argon. The evacuate/refill cycle was repeated 2-3 times before the addition of dry toluene and the whole setup was maintained under argon.

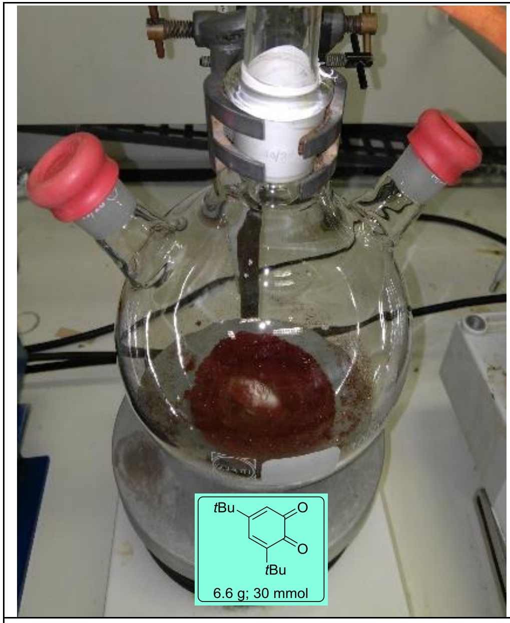	Close view of *o*-quinone.
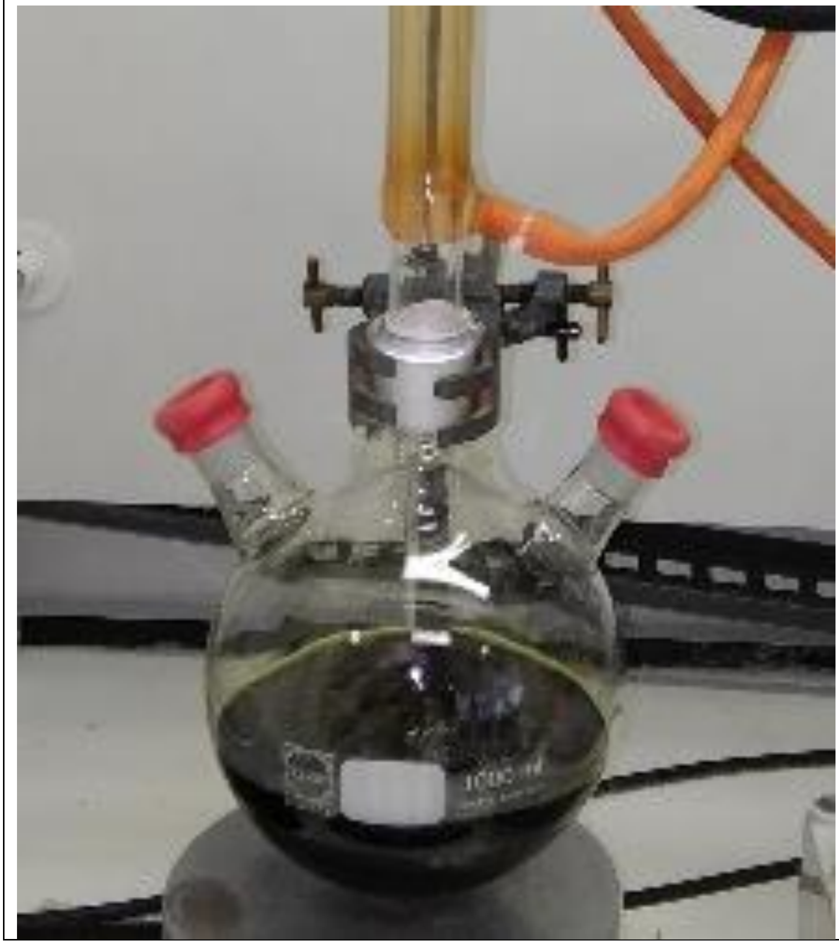	Addition of toluene

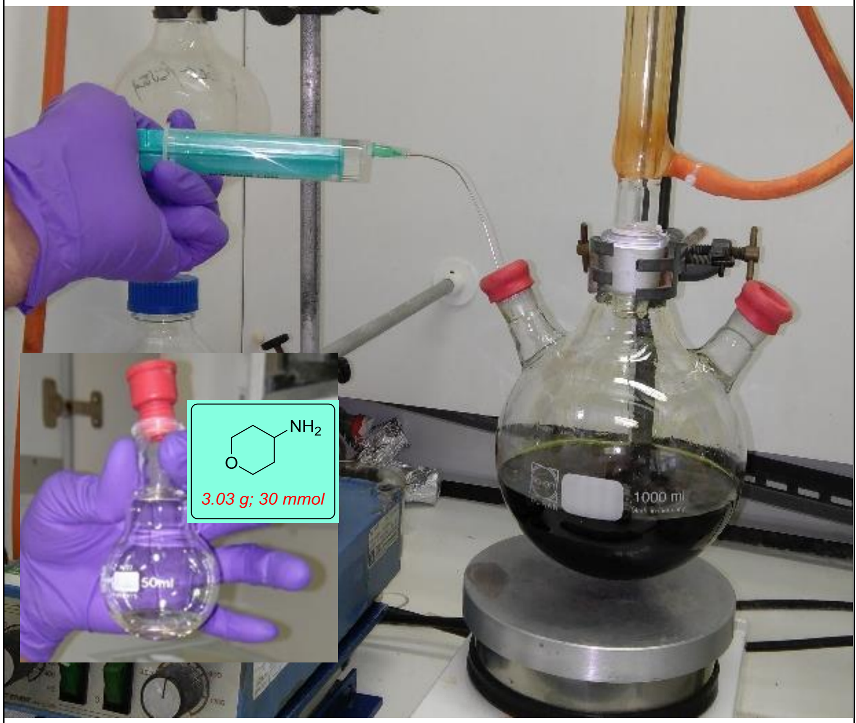	Addition of 4-aminotetrahydropyran in toluene at 25 ˚C. (Inside Picture: 4-aminotetrahydropyran, 3.03 g; 30.0 mmol).

<table>
<tr>
<td>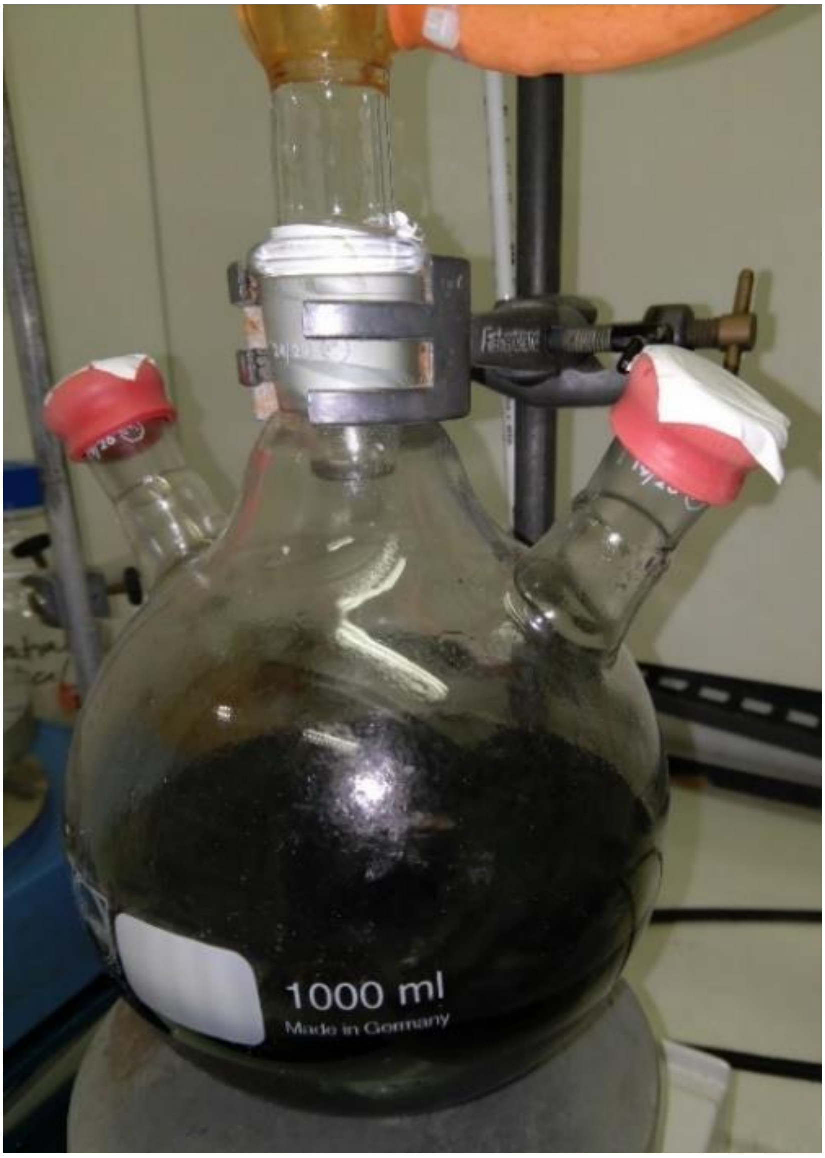
</td>
<td>Completion of ketimine/hemiaminal formation. (Note: the colour changes from deep green (o-quinone) to violet (ketimine/hemiaminal) during the course of reaction).</td>
</tr>
<tr>
<td colspan="2">Ketimine/hemiaminal formation:
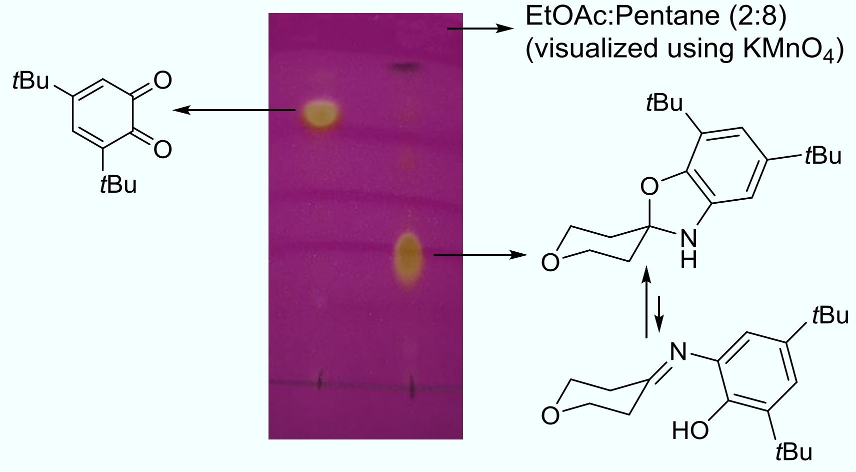
</td>
</tr>
</table>

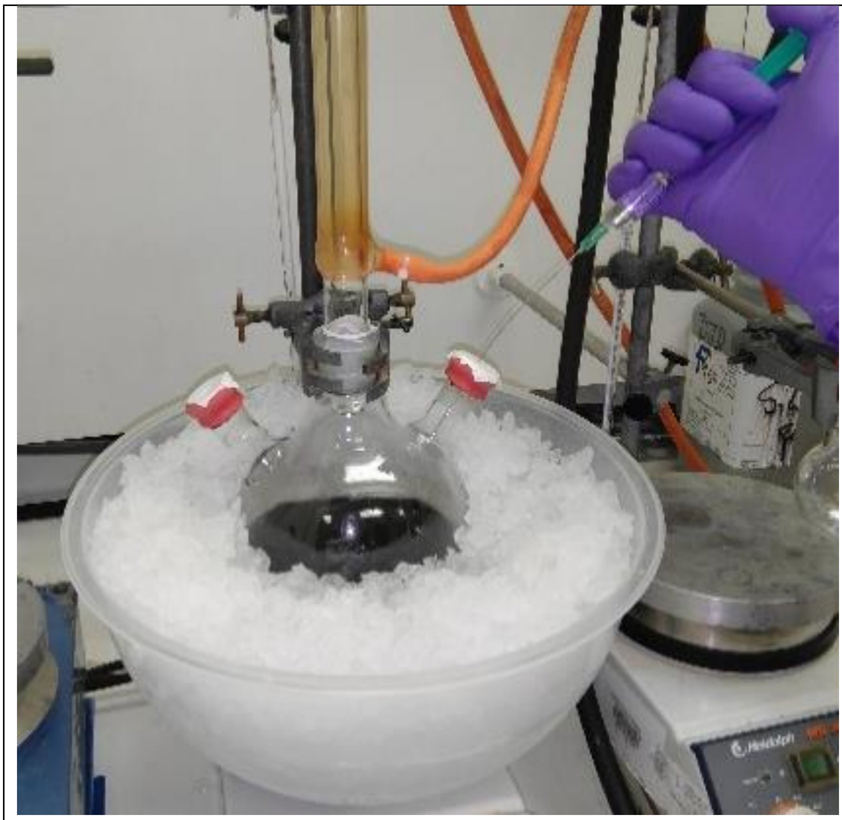	***Step 2: (Allylation):*** Addition of TMEDA at 0 ˚C in neat form.
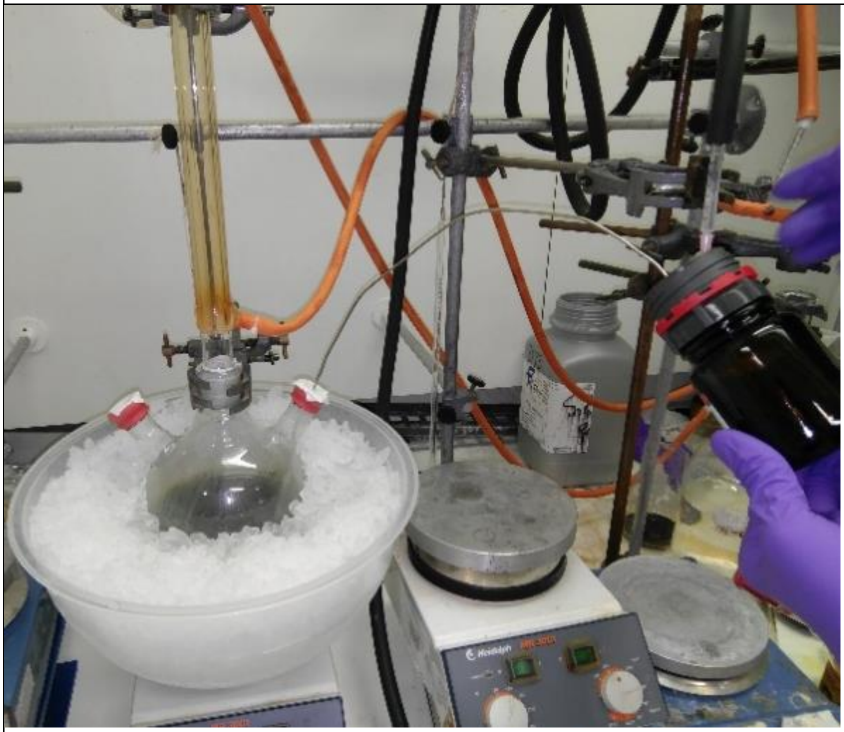	Addition of allylmagnesium bromide (1.0 M in Et_2O) *via* cannula.

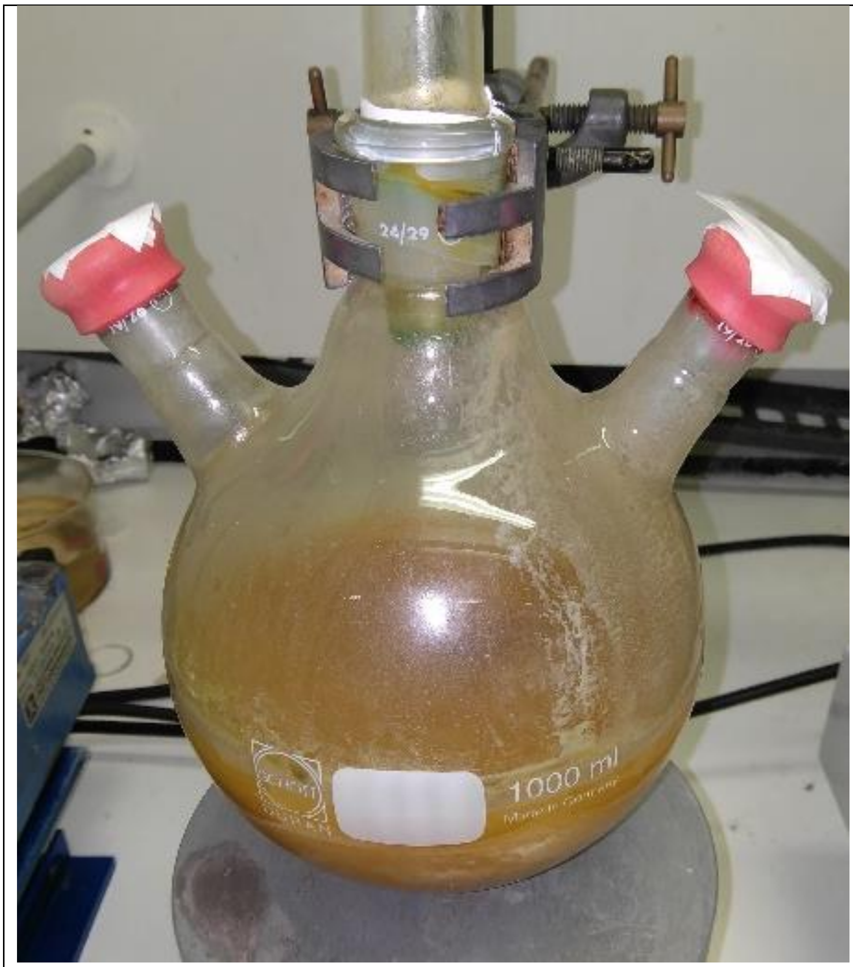

Completion of allylation.

Note: *(violet to yellow colouration).*

Allylation: (A small aliquot was worked up in NH_4Cl-EtOAc for TLC)

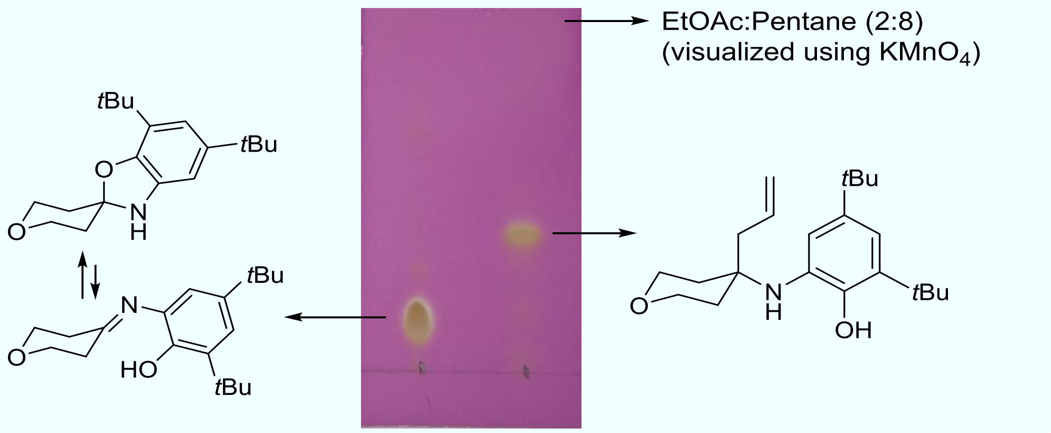

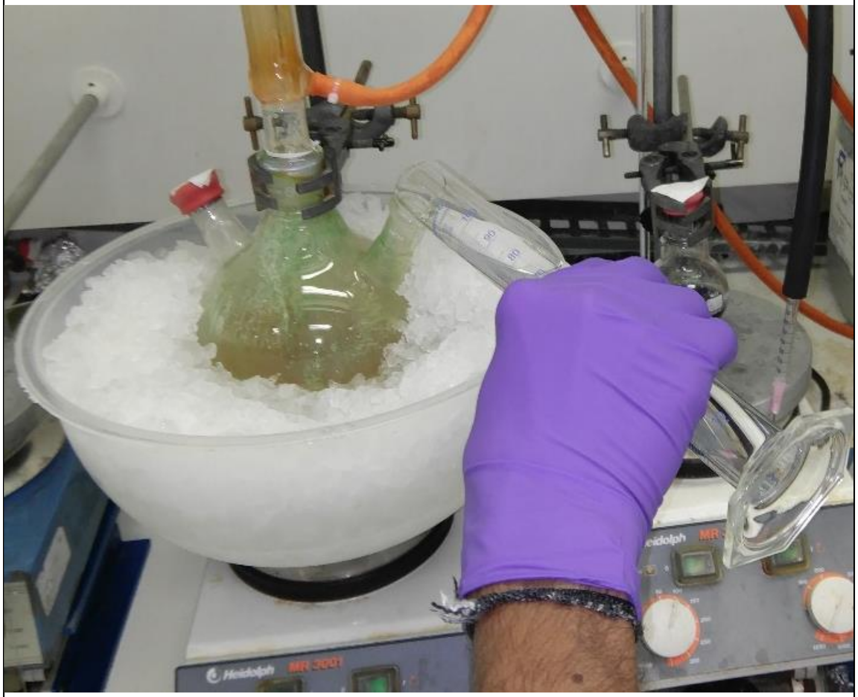	***Step 3: Oxidative cleavage:*** Quenching the remaining RMgX by the addition of 1M NaOH (150 mL) at 0 ˚C under argon atmosphere. ***Note****: Care must be taken while addition due to vigorous reaction.*
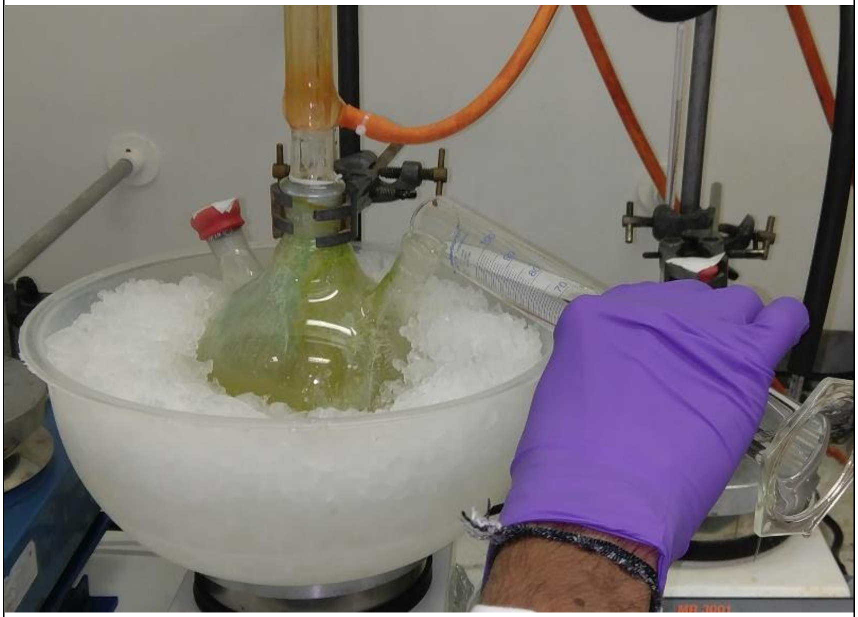	Addition of CH_3CN (300 mL).

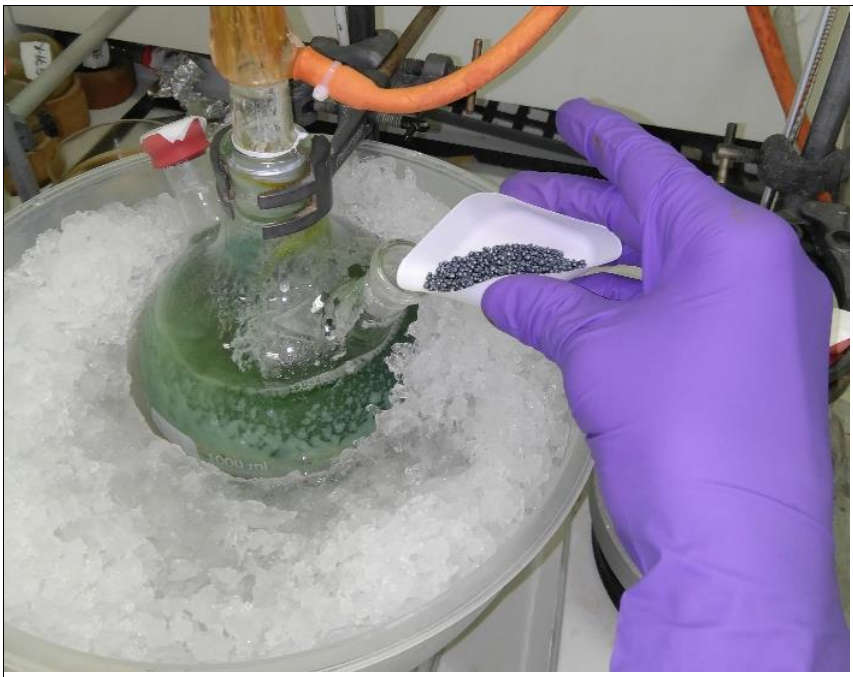	Addition of Iodine (7.62 g, 30.0 mmol)
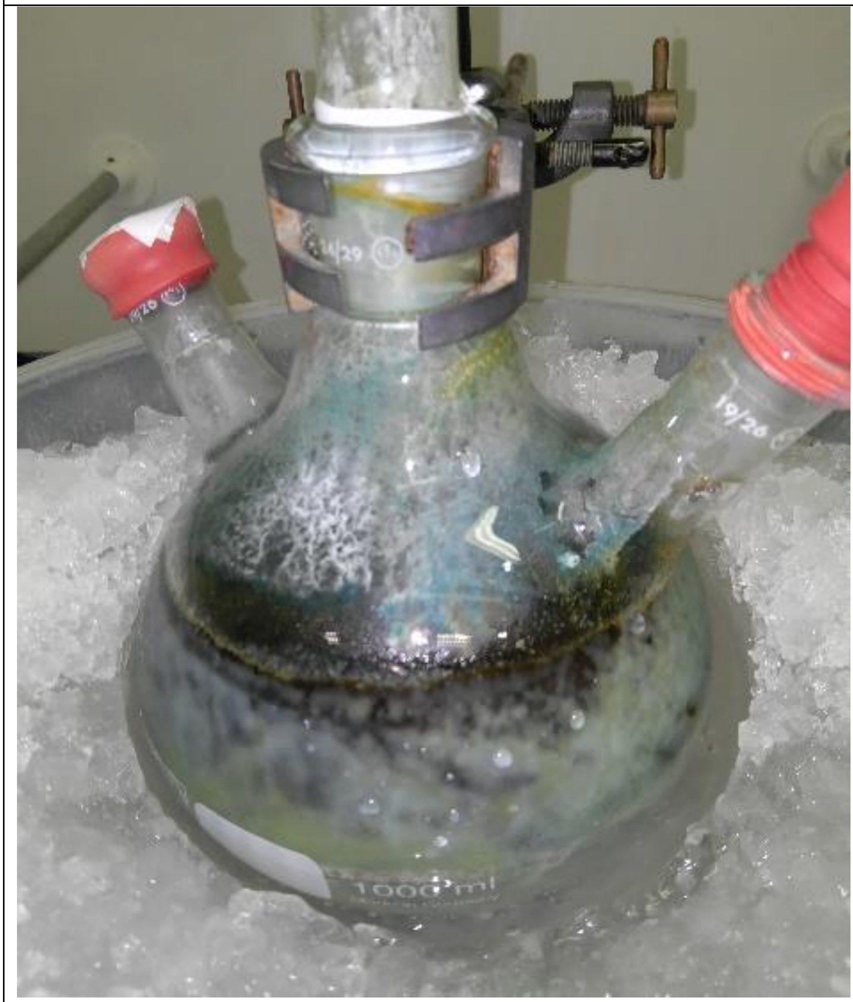	Completion of oxidation ***Note:*** *(green to orange)*

Oxidative Cleavage:

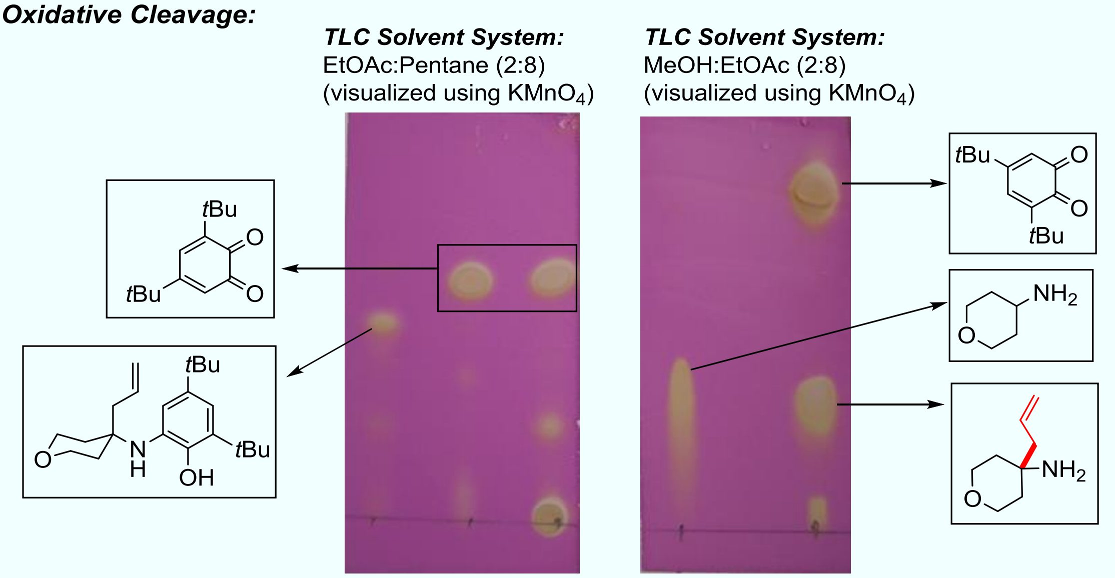

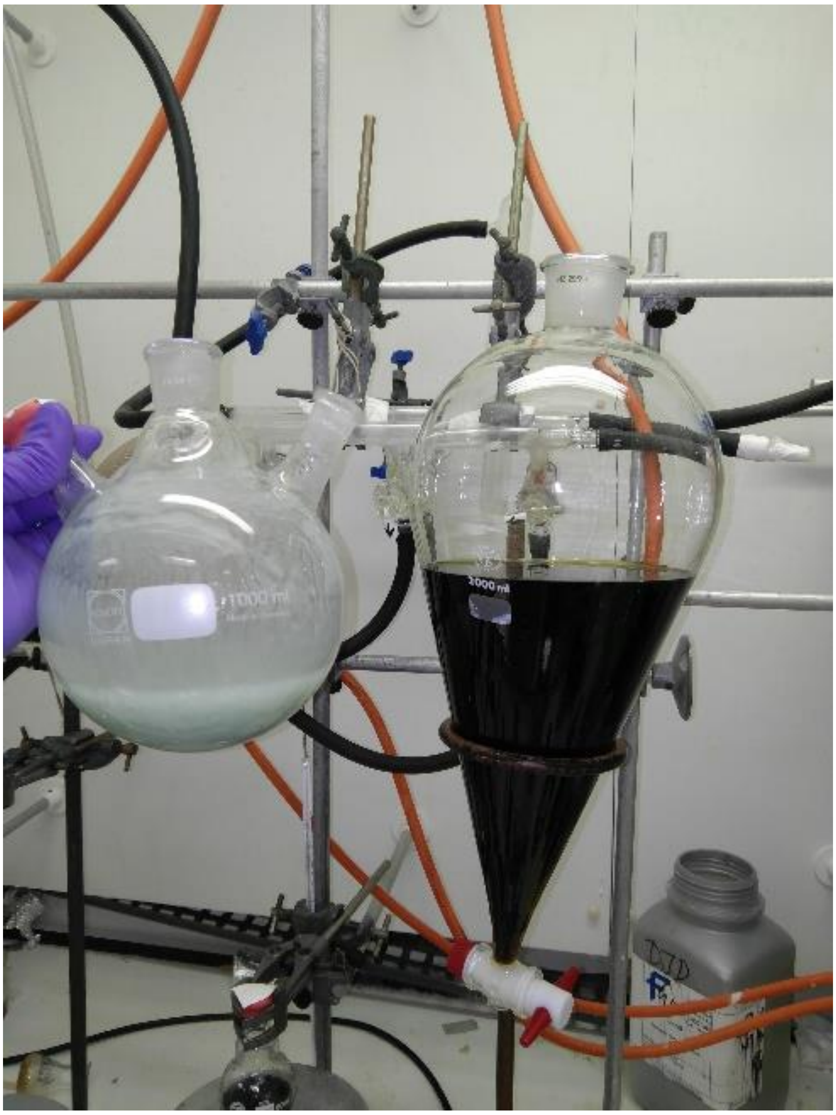

Work up:

Separation of Organic layer from the heterogeneous mixture using CH_3CN.

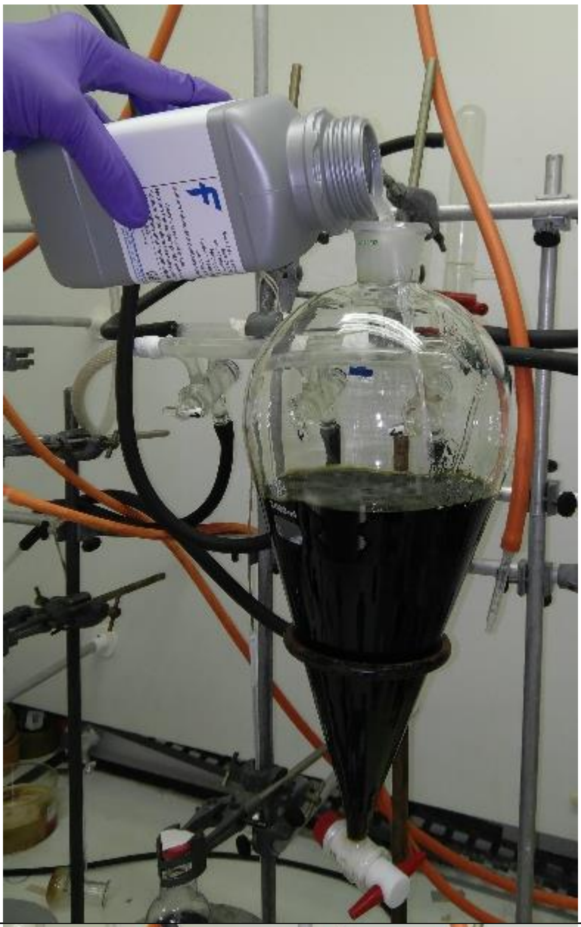	Organic layer was washed with saturated sodium thiosulphate (1 x 50 mL) to remove Iodine.
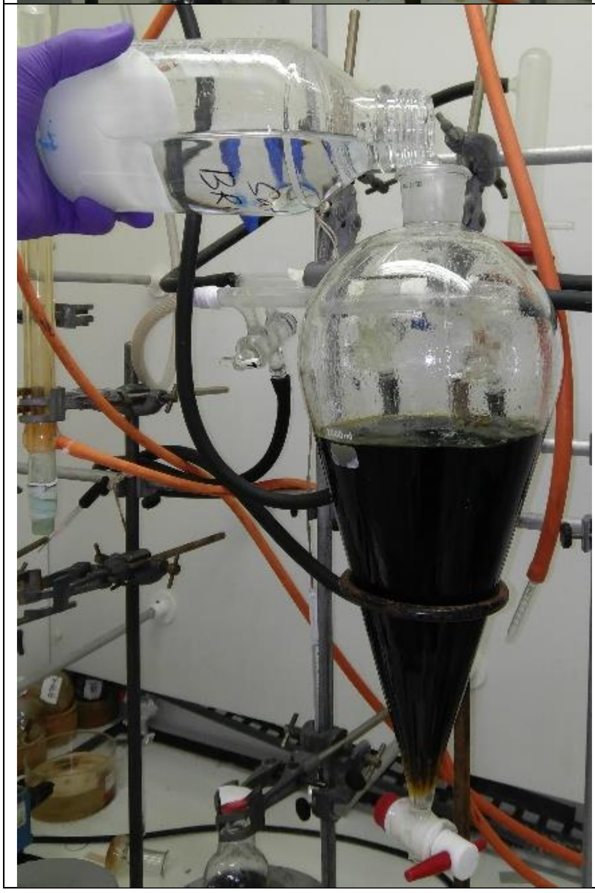	Washed with brine twice (2 x 50 mL) to remove TMEDA and any residual iodinated species.

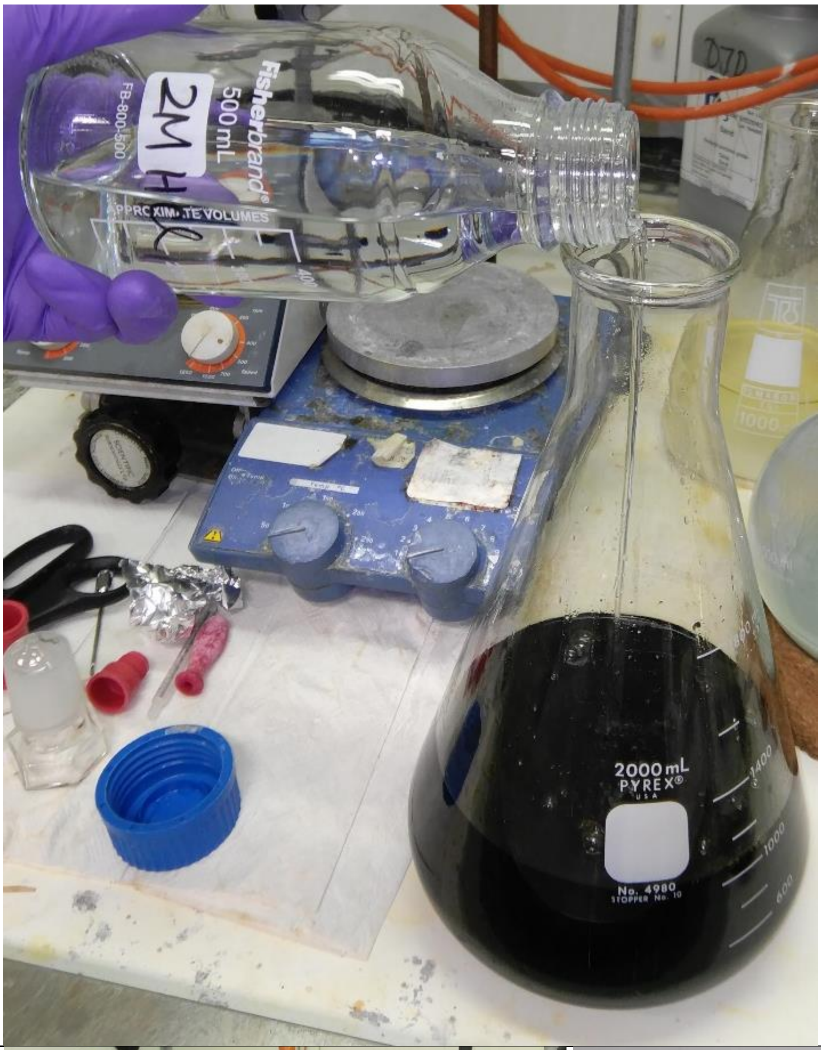	Organic layer was acidified directly with 2 M HCl (***General Procedure B***). For ***General Procedure A and C***; the resulting organic layer was dried and condensed *via* vacuo, followed by column chromatography.
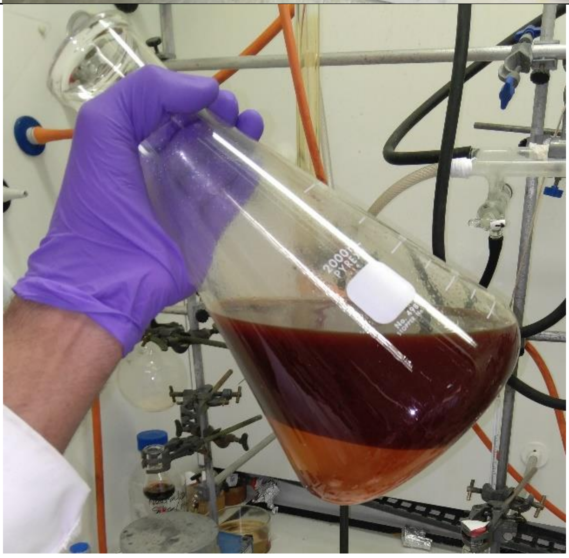 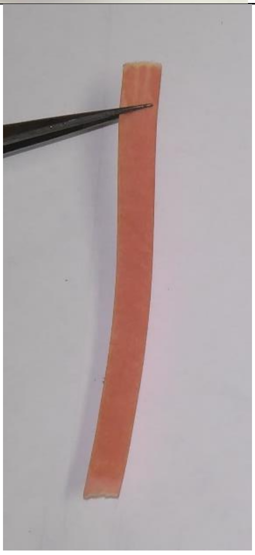	Dispersion of aqueous acidic phase from organic phase; and conformation of its acidity using pH paper.

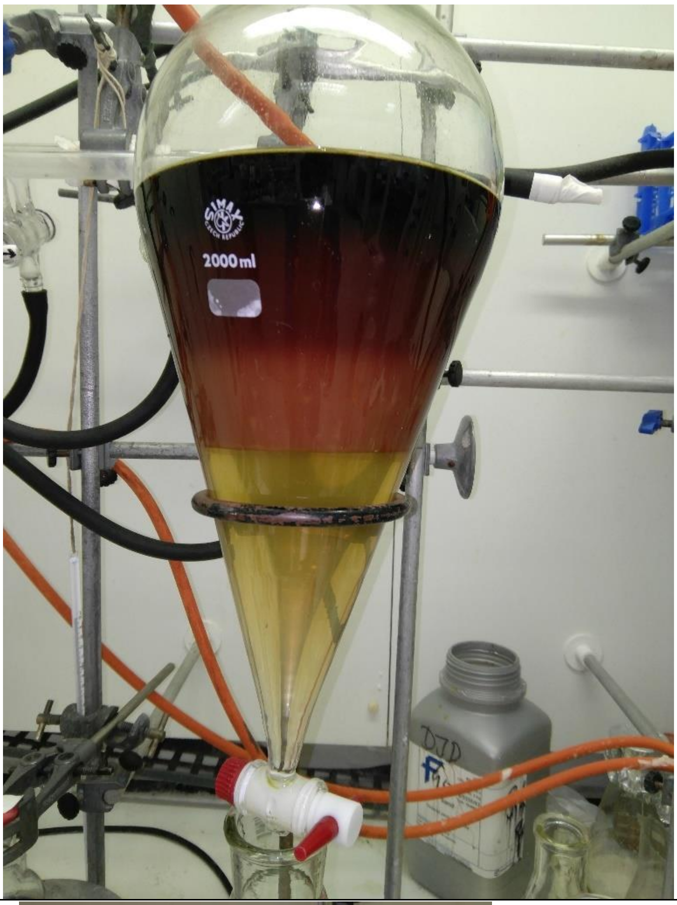	Partitioned between aqueous and organic layers
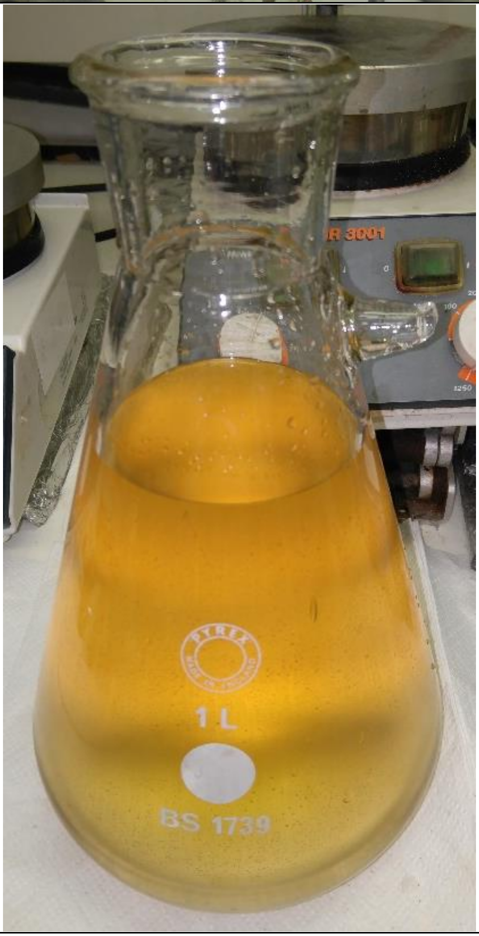	Separation of aqueous acidic phase from organic phase and followed by further extraction twice with 2M aqueous HCl (2 x 250 mL).

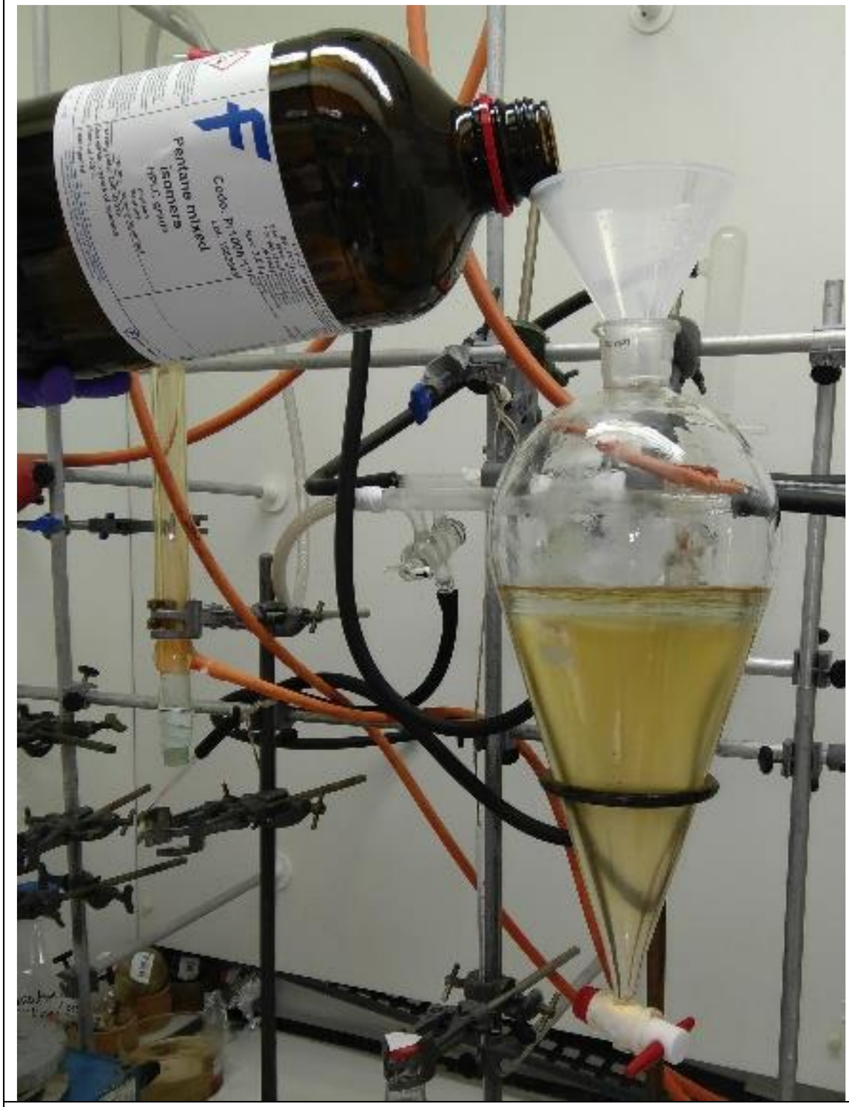	The aqueous acidic phase was washed with pentane to remove any I_2 impurity.
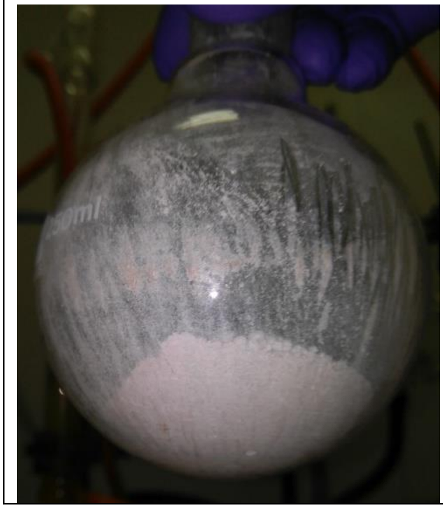 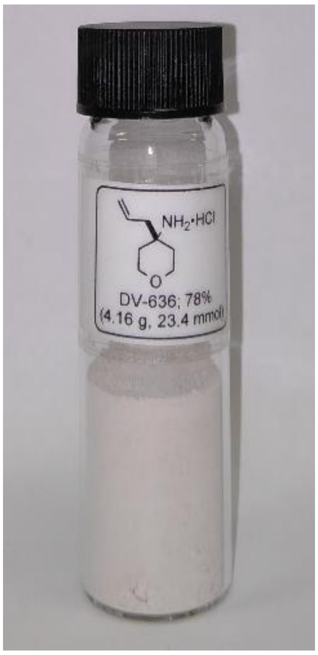	Evaporation of aqueous acidic phase by rotary evaporator gave ammonium salt **27** as off-white powder (4.16 g, 23.4 mmol, 78%).

5: Spectral data for *α*-functionalized products (1-68):

Synthesis of 2-(4-methoxyphenyl)pent-4-en-2-amine (1)

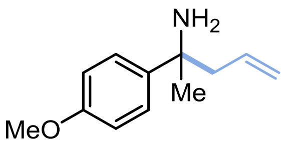

The title compound was prepared using **General Procedure A**. The cleavage of phenol unit was performed by using 3 equiv of I_2 and 5 equiv NaOH (1 M). Yellow oil (205 mg, 1.07 mmol, 81%). **FT-IR** (thin film) ν_{max} (cm^{-1}): 3073, 2962, 1610, 1511, 1247, 1034, 830. **^{1}H NMR** (400 MHz, $CDCl_3$): δ 7.37 (d, *J* = 8.8 Hz, 2 H), 6.86 (d, *J* = 8.8 Hz, 2 H), 5.57 (dddd, *J* = 17.0, 10.2, 7.9, 6.8 Hz, 1 H), 5.09 – 5.03 (m, 2 H), 3.80 (s, 3 H), 2.53 (ddt, *J* = 13.5, 6.8, 1.3 Hz, 1 H), 2.39 (ddt, *J* = 13.5, 7.8, 1.1 Hz, 1 H), 1.57 (br s, 2 H, NH_2), 1.45 (s, 3 H). **^{13}C NMR** (101 MHz, $CDCl_3$): δ 158.0, 140.7, 134.5, 126.4, 118.5, 113.5, 55.3, 54.3, 49.8, 31.0. **HRMS** (ESI) Calcd for $C_{12}H_{15}O$ [M-NH_2]: 175.1117, found: 175.1118.

Synthesis of 2-phenylpent-4-en-2-amine (2)

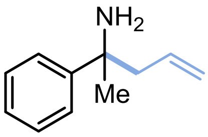

The title compound was prepared using **General Procedure A**. Brown oil (125 mg, 0.77 mmol, 94%). **FT-IR** (thin film) ν_{max} (cm^{-1}): 3060, 2925, 1639, 1445, 1197, 917, 766, 699. **^{1}H NMR** (400 MHz, $CDCl_3$): δ 7.49 – 7.45 (m, 2 H), 7.36 – 7.31 (m, 2 H), 7.25 – 7.20 (m, 1 H), 5.56 (dddd, *J* = 17.0, 10.1, 7.9, 6.8 Hz, 1 H), 5.11 – 5.04 (m, 2 H), 2.62 – 2.44 (m, 4 H, CH_2, NH_2), 1.50 (s, 3 H). **^{13}C NMR** (101 MHz, $CDCl_3$): δ 147.9, 134.1, 128.3, 126.5, 125.3, 118.9, 55.2, 49.4, 30.4. **HRMS** (ESI) Calcd for $C_{11}H_{16}N$ [$(M+H)^+$]: 162.1277, found: 162.1278.

Synthesis of 1,1-diphenylbut-3-en-1-amine (3)

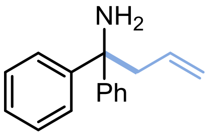

The title compound was prepared using **General Procedure A**. Yellow semi-solid (211 mg, 0.95 mmol, 80%). **FT-IR** (thin film) ν_{max} (cm^{-1}): 3059, 2980, 1598, 1492, 1445, 1156, 917, 755, 698. **^{1}H NMR** (400 MHz, $CDCl_3$): δ 7.43 – 7.40 (m, 4 H), 7.34 – 7.30 (m, 4 H), 7.25 – 7.20 (m, 2 H), 5.55 (dddd, *J* = 16.6, 10.2, 7.6, 6.8 Hz, 1 H), 5.22 – 5.10 (m, 2 H), 3.05 (dt, *J* = 7.1, 1.2 Hz, 2 H), 1.87 (br s, 2 H, NH_2).

^{13}C NMR (101 MHz, $CDCl_3$): δ 148.2, 134.2, 128.2, 126.7, 126.5, 119.3, 60.3, 47.6. **HRMS** (ESI) Calcd for $C_{16}H_{15}$ [M-NH_2]: 207.1168, found: 207.1170.

Synthesis of 2-(2-methoxyphenyl)pent-4-en-2-amine (4)

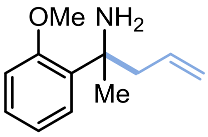

The title compound was prepared using **General Procedure A**. The cleavage of phenol unit was performed by using 3 equiv of I_2 and 5 equiv NaOH (1 M). Viscous yellow oil (160 mg, 0.84 mmol, 63%). **FT-IR** (thin film) ν_{max} (cm^{-1}): 3375, 3073, 2934, 1582, 1488, 1234, 1027, 753. **^{1}H NMR** (400 MHz, $CDCl_3$): δ 7.31 (dd, *J* = 7.7, 1.8 Hz, 1 H), 7.26 – 7.20 (m, 1 H), 6.94 – 6.89 (m, 2 H), 5.57 – 5.48 (m, 1 H), 5.04 – 4.94 (m, 2 H), 3.87 (s, 3 H), 2.75 – 2.62 (m, 2 H), 2.26 (br s, 2 H, NH_2), 1.50 (s, 3 H). **^{13}C NMR** (101 MHz, $CDCl_3$): δ 157.6, 135.7, 135.5, 128.0, 126.9, 120.6, 117.5, 111.5, 55.2, 55.0, 46.6, 28.0. **HRMS** (ESI) Calcd for $C_{12}H_{18}NO$ [$(M+H)^+$]: 192.1383, found: 192.1382.

Synthesis of 2-(3-methoxyphenyl)pent-4-en-2-amine (5)

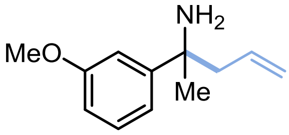

The title compound was prepared using **General Procedure A**. The cleavage of phenol unit was performed by using 3 equiv of I_2 and 5 equiv NaOH (1 M). Yellow oil (197 mg, 1.03 mmol, 78%). **FT-IR** (thin film) ν_{max} (cm^{-1}): 3074, 2924, 1582, 1431, 1244, 1045, 781, 702. **^{1}H NMR** (400 MHz, $CDCl_3$): δ 7.27 – 7.23 (m, 1 H), 7.04 – 7.01 (m, 2 H), 6.77 – 6.75 (m, 1 H), 5.56 (dddd, *J* = 17.0, 10.1, 8.0, 6.7 Hz, 1 H), 5.10 – 5.03 (m, 2 H), 3.80 (s, 3 H), 2.57 (ddt, *J* = 13.6, 6.7, 1.3 Hz, 1 H), 2.42 (ddt, *J* = 13.6, 8.1, 1.1 Hz, 1 H), 2.06 (br s, 2 H, NH_2), 1.46 (s, 3 H). **^{13}C NMR** (101 MHz, $CDCl_3$): δ 159.6, 150.2, 134.2, 129.2, 118.7, 117.8, 111.7, 111.2, 55.3, 54.9, 49.5, 30.7. **HRMS** (ESI) Calcd for $C_{12}H_{18}NO$ [$(M+H)^+$]: 192.1383, found: 192.1385.

Synthesis of 2-(*p*-tolyl)pent-4-en-2-amine (6)

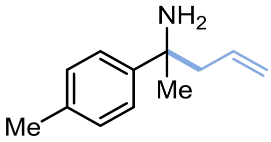

The title compound was prepared using **General Procedure A**. Yellow oil (174 mg, 0.99 mmol, 67%). **FT-IR** (thin film) ν_{max} (cm^{-1}): 2980, 2923, 1639, 1513, 914, 816. **^{1}H NMR** (400 MHz, $CDCl_3$): δ 7.36 (d, *J*

= 8.3 Hz, 2 H), 7.15 (d, J = 8.3 Hz, 2 H), 5.59 (dddd, J = 17.0, 10.1, 7.9, 6.7 Hz, 1 H), 5.11 – 5.04 (m, 2 H), 2.56 (ddt, J = 13.5, 6.7, 1.2 Hz, 1 H), 2.41 (ddt, J = 13.5, 7.8, 1.1 Hz, 1 H), 2.34 (s, 3 H), 1.53 (br s, 2 H, NH_2), 1.46 (s, 3 H). ^{13}**C NMR** (101 MHz, $CDCl_3$): δ 145.9, 135.7, 134.6, 128.9, 125.2, 118.4, 54.4, 49.8, 31.1, 21.0. **HRMS** (ESI) Calcd for $C_{12}H_{18}N$ [$(M+H)^+$]: 176.1434, found: 176.1435.

Synthesis of 4-(2-aminopent 4-en-2-yl)phenol hydrochloride (7)

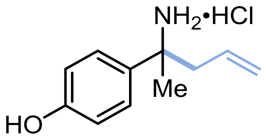

The title compound was prepared using **General Procedure B**. A mixture of Toluene:THF (5:1)(0.1 M) was used to improve the solubility of starting material for ketimine formation. Yellow solid (184 mg, 0.86 mmol, 59%). **FT-IR** (thin film) ν_{max} (cm^{-1}): 3401, 3025, 2922, 1614, 1519, 1224, 1188, 833. ^{1}**H NMR** (400 MHz, CD_3OD): δ 7.31 (d, J = 8.8 Hz, 2 H), 6.86 (d, J = 8.8 Hz, 2 H), 5.54 (ddt, J = 17.3, 10.1, 7.2 Hz, 1 H), 5.23 – 5.14 (m, 2 H), 2.81 – 2.69 (m, 2 H), 1.69 (s, 3 H). ^{13}**C NMR** (101 MHz, CD_3OD): δ 158.8, 132.3, 131.8, 127.6, 121.3, 116.6, 59.2, 46.5, 25.1. **HRMS** (ESI) Calcd for $C_{11}H_{13}O$ [$M-NH_3Cl$]: 161.0961, found: 161.0961.

Synthesis of 2-(4-fluorophenyl)pent-4-en-2-amine (8)

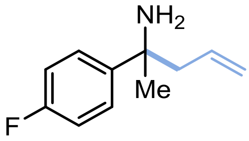

The title compound was prepared using **General Procedure A**. Brown oil (183 mg, 1.02 mmol, 71%). **FT-IR** (thin film) ν_{max} (cm^{-1}): 2980, 1601, 1509, 1225, 919, 834. ^{1}**H NMR** (400 MHz, $CDCl_3$): δ 7.42 (dd, J = 8.9, 5.3 Hz, 2 H), 6.99 (t, J = 8.7 Hz, 2 H), 5.53 (dddd, J = 16.6, 10.5, 7.8, 6.8 Hz, 1 H), 5.08 – 5.03 (m, 2 H), 2.54 (ddt, J = 13.6, 6.9, 1.2 Hz, 1 H), 2.41 (ddt, J = 13.6, 7.9, 1.0 Hz, 1 H), 2.28 (br s, 2 H, NH_2), 1.46 (s, 3 H). ^{19}**F NMR** (377 MHz, $CDCl_3$): δ -117.2 (d, J = 11.6 Hz). ^{13}**C NMR** (101 MHz, $CDCl_3$): δ 161.5 (d, J_{C-F} = 245.6 Hz), 143.7 (d, J_{C-F} = 3.2 Hz), 133.8, 127.0 (d, J_{C-F} = 7.9 Hz), 119.0, 114.9 (d, J_{C-F} = 21.1 Hz), 54.8, 49.6, 30.7. **HRMS** (ESI) Calcd for $C_{11}H_{15}NF$ [$(M+H)^+$]: 180.1183, found: 180.1182.

Synthesis of 2-(4-chlorophenyl)pent-4-en-2-amine (9)

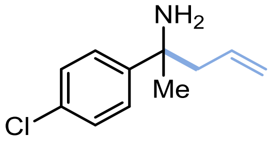

The title compound was prepared using **General Procedure A**. Yellow oil (171 mg, 0.87 mmol, 68%). **FT-IR** (thin film) $ν_{max}$ (cm^{-1}): 3075, 2923, 2849, 1639, 1490, 1195, 918, 826. **^{1}H NMR** (400 MHz, $CDCl_3$): δ 7.32 (d, *J* = 8.7 Hz, 2 H), 7.21 (d, *J* = 8.7 Hz, 2 H), 5.45 (dddd, *J* = 16.5, 10.8, 7.9, 6.8 Hz, 1 H), 5.02 – 4.96 (m, 2 H), 2.47 (ddt, *J* = 13.6, 6.8, 1.2 Hz, 1 H), 2.33 (ddt, *J* = 13.6, 7.9, 1.0 Hz, 1 H), 2.03 (br s, 2 H, NH_2), 1.39 (s, 3 H). **^{13}C NMR** (101 MHz, $CDCl_3$): δ 146.7, 133.7, 132.2, 128.3, 126.9, 119.2, 54.8, 49.5, 30.7. **HRMS** (ESI) Calcd for $C_{11}H_{15}N_{35}Cl$ [$(M+H)^+$]: 196.0888, found: 196.0888.

Synthesis of 2-(4-bromophenyl)pent-4-en-2-amine (10)

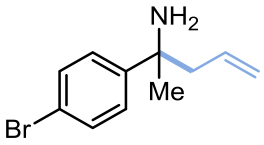

The title compound was prepared using **General Procedure A**. Pale brown oil (159 mg, 0.66 mmol, 66%). **FT-IR** (thin film) $ν_{max}$ (cm^{-1}): 3075, 2965, 2924, 1639, 1484, 1007, 918, 822. **^{1}H NMR** (400 MHz, $CDCl_3$): δ 7.43 (d, *J* = 8.6 Hz, 2 H), 7.33 (d, *J* = 8.6 Hz, 2 H), 5.58 – 5.48 (m, 1 H), 5.08 – 5.02 (m, 2 H), 2.52 (ddt, *J* = 13.6, 6.9, 1.2 Hz, 1 H), 2.37 (ddt, *J* = 13.6, 7.9, 1.1 Hz, 1 H), 1.64 (br s, 2 H, NH_2), 1.43 (s, 3 H). **^{13}C NMR** (101 MHz, $CDCl_3$): δ 147.7, 133.9, 131.2, 127.3, 120.2, 119.0, 54.6, 49.7, 30.9. **HRMS** (ESI) Calcd for $C_{11}H_{15}N_{79}Br$ [$(M+H)^+$]: 240.0382, found: 240.0381.

Synthesis of 4-(4-fluorophenyl)hepta-1,6-dien-4-amine (11)

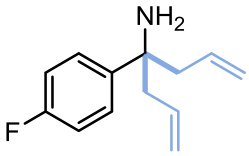

The title compound was prepared using 2-amino-2-(4-fluorophenyl)acetonitrile as amine precursor. ketimine formation was done at 80 ˚C for 8 h. 10 equiv of allylmagnesium bromide (1 M in Et_2O) was used and the remaining procedure is same as **General Procedure A**. Yellow oil (79.0 mg, 0.38 mmol, 58%). **FT-IR** (thin film) $ν_{max}$ (cm^{-1}): 3076, 2920, 1601, 1508, 1224, 916, 833, 815. **^{1}H NMR** (400 MHz, $CDCl_3$): δ 7.38 (dd, *J* = 8.9, 5.3 Hz, 2 H), 6.99 (t, *J* = 8.7 Hz, 2 H), 5.50 (dddd, *J* = 16.7, 10.1, 8.3, 6.4 Hz, 2 H), 5.08 – 5.02 (m, 4 H), 2.60 (ddt, *J* = 13.7, 6.3, 1.4 Hz, 2 H), 2.38 (ddt, *J* = 13.7, 7.9, 1.0 Hz, 2 H), 1.55 (br s, 2 H, NH_2). **^{19}F NMR** (377 MHz, $CDCl_3$): δ -117.4 (d, *J* = 6.1 Hz). **^{13}C NMR** (101 MHz, $CDCl_3$): δ 161.4 (d, $J_{C\text{-}F}$ = 245.6 Hz), 142.4 (d, $J_{C\text{-}F}$ = 3.2 Hz), 133.7, 127.6 (d, $J_{C\text{-}F}$ = 8.0 Hz), 118.9, 114.8 (d, $J_{C\text{-}F}$ = 20.7 Hz), 56.7, 48.2. **HRMS** (ESI) Calcd for $C_{13}H_{17}NF$ [$(M+H)^+$]: 206.1340, found: 206.1339.

Synthesis of 2-(3,5-difluorophenyl)pent-4-en-2-amine (12)

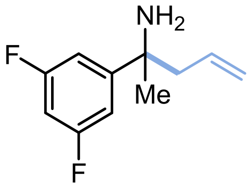

The title compound was prepared using **General Procedure A**. Brown oil (207 mg, 1.05 mmol, 82%). **FT-IR** (thin film) ν_{max} (cm^{-1}): 3079, 2969, 2926, 1623, 1597, 1432, 1115, 984, 856. **^{1}H NMR** (400 MHz, $CDCl_3$): δ 6.97 (dd, *J* = 9.4, 2.3 Hz, 2 H), 6.62 (tt, *J* = 8.8, 2.3 Hz, 1 H), 5.56 – 5.46 (m, 1 H), 5.08 – 5.02 (m, 2 H), 2.49 (ddt, *J* = 13.6, 6.7, 1.2 Hz, 1 H), 2.34 (ddt, *J* = 13.7, 8.0, 1.0 Hz, 1 H), 1.59 (br s, 2 H, NH_2), 1.41 (s, 3 H). **^{19}F NMR** (377 MHz, $CDCl_3$): δ -110.1. **^{13}C NMR** (101 MHz, $CDCl_3$): δ 163.0 (dd, $J_{C\text{-}F}$ = 248.0, 12.7 Hz), 153.3 (t, $J_{C\text{-}F}$ = 7.9 Hz), 133.5, 119.3, 108.6 (dd, $J_{C\text{-}F}$ = 19.0, 7.0, Hz), 101.6 (t, $J_{C\text{-}F}$ = 25.8 Hz), 54.8 (t, $J_{C\text{-}F}$ = 2.1 Hz), 49.5, 30.8. **HRMS** (ESI) Calcd for $C_{11}H_{14}F_2N$ [$(M+H)^+$]: 198.1089, found: 198.1087.

Synthesis of 2-(naphthalen-1-yl)pent-4-en-2-amine (13)

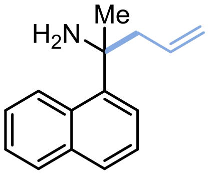

The title compound was prepared using **General Procedure A**. Yellow oil (148 mg, 0.70 mmol, 60%). **FT-IR** (thin film) ν_{max} (cm^{-1}): 3047, 2925, 1638, 1509, 1177, 914, 803, 776. **^{1}H NMR** (400 MHz, $CDCl_3$): δ 8.89 (d, *J* = 9.0 Hz, 1 H), 7.89 (dd, *J* = 8.0, 1.8 Hz, 1 H), 7.77 (d, *J* = 8.0 Hz, 1 H), 7.61 (dd, *J* = 7.4, 1.3 Hz, 1 H), 7.54 – 7.40 (m, 3 H), 5.59 (ddt, *J* = 17.3, 10.2, 7.4 Hz, 1 H), 5.12 – 5.03 (m, 2 H), 3.01 (ddt, *J* = 13.7, 7.1, 1.2 Hz, 1 H), 2.84 (ddt, *J* = 13.8, 7.6, 1.2 Hz, 1 H), 1.77 (s, 5 H, CH_3, NH_2). **^{13}C NMR** (101 MHz, $CDCl_3$): δ 143.4, 135.1, 134.7, 131.3, 129.5, 128.4, 127.4, 125.1, 125.0, 124.9, 124.1, 118.4, 56.2, 48.2, 31.4. **HRMS** (ESI) Calcd for $C_{15}H_{18}N$ [$(M+H)^+$]: 212.1434, found: 212.1437.

Synthesis of 2-(naphthalen-2-yl)pent-4-en-2-amine (14)

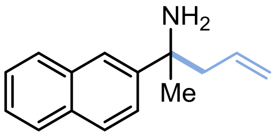

The title compound was prepared using **General Procedure A**. Pale yellow oil (180 mg, 0.73 mmol, 62%). **FT-IR** (thin film) ν_{max} (cm^{-1}): 3364, 3057, 2924, 2856, 1638, 1600, 916, 818, 748. **^{1}H NMR** (400 MHz, $CDCl_3$): δ 7.91 (s, 1 H), 7.85 – 7.81 (m, 3 H), 7.60 (dd, *J* = 8.7, 2.0 Hz, 1 H), 7.50 – 7.43 (m, 2 H),

5.57 (ddt, *J* = 17.1, 9.8, 7.2 Hz, 1 H), 5.12 – 5.03 (m, 2 H), 2.69 (dd, *J* = 13.6, 6.6 Hz, 1 H), 2.51 (dd, *J* = 13.6, 8.0 Hz, 1 H), 1.66 (br s, 2 H, NH_2), 1.57 (s, 3 H). **^{13}C NMR** (101 MHz, $CDCl_3$): δ 146.2, 134.4, 133.3, 132.1, 128.2, 127.9, 127.5, 126.1, 125.7, 124.4, 123.5, 118.7, 54.9, 49.6, 31.1. **HRMS** (ESI) Calcd for $C_{15}H_{18}N$ [$(M+H)^+$]: 212.1434, found: 212.1435.

Synthesis of 1-allyl-2,3-dihydro-1*H*-inden-1-amine (15)

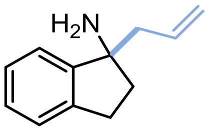

The title compound was prepared using **General Procedure A**. Yellow oil (159 mg, 0.92 mmol, 61%). **FT-IR** (thin film) ν_{max} (cm^{-1}): 3354, 3071, 2980, 2930, 1638, 1457, 1157, 998, 915, 761. **^{1}H NMR** (400 MHz, $CDCl_3$): δ 7.27 – 7.20 (m, 4 H), 5.78 (ddt, *J* = 17.4, 10.2, 7.4 Hz, 1 H), 5.17 – 5.10 (m, 2 H), 2.97 – 2.80 (m, 2 H), 2.49 – 2.37 (m, 2 H), 2.28 (ddd, *J* = 12.9, 7.9, 4.0 Hz, 1 H), 2.03 (br s, 2 H, NH_2), 1.93 (dt, *J* = 12.8, 8.3 Hz, 1 H). **^{13}C NMR** (101 MHz, $CDCl_3$): δ 149.4, 142.8, 134.2, 127.5, 126.6, 125.0, 122.8, 118.8, 64.1, 45.6, 40.8, 29.6. **HRMS** (ESI) Calcd for $C_{12}H_{16}N$ [$(M+H)^+$]: 174.1277, found: 174.1277.

Synthesis of 4-methylnon-1-en-4-amine (16)

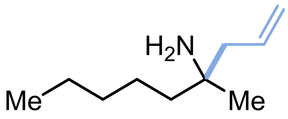

The title compound was prepared using **General Procedure B**. Yellow oil (270 mg, 1.41 mmol, 81%). **FT-IR** (thin film) ν_{max} (cm^{-1}): 3017, 2930, 2874, 1617, 1518, 1298, 994, 921. **^{1}H NMR** (400 MHz, $CDCl_3$): **^{1}H NMR** (400 MHz, $CDCl_3$): δ 5.83 (ddt, *J* = 16.8, 10.3, 7.5 Hz, 1 H), 5.15 – 5.04 (m, 2 H), 3.02 – 2.31 (br s, 2 H, NH_2), 2.15 (dt, *J* = 7.4, 1.1 Hz, 2 H), 1.38 – 1.26 (m, 8 H), 1.09 (s, 3 H), 0.88 (t, *J* = 6.9 Hz, 3 H). **^{13}C NMR** (101 MHz, $CDCl_3$): δ 134.3, 118.6, 52.5, 46.7, 42.2, 32.6, 27.3, 23.6, 22.8, 14.2. **HRMS** (ESI) Calcd for $C_{10}H_{22}N$ [$(M+H)^+$]: 156.1747, found: 156.1745.

Synthesis of 4-propylhept-1-en-4-amine hydrochloride (17)

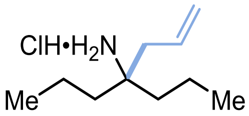

The title compound was prepared using **General Procedure B**. Yellow solid (323 mg, 1.68 mmol, 95%). **FT-IR** (thin film) ν_{max} (cm^{-1}): 2964, 2876, 1643, 1518, 1252. **^{1}H NMR** (400 MHz, $CDCl_3$): δ 8.32 (br s, 3 H, NH_3^+), 5.90 (ddt, *J* = 17.5, 10.2, 7.3 Hz, 1 H), 5.24 – 5.19 (m, 2 H), 2.45 (d, *J* = 7.3 Hz, 2 H), 1.66 – 1.62 (m, 4 H), 1.49 – 1.43 (m, 4 H), 0.93 (t, *J* = 7.2 Hz, 6 H). **^{13}C NMR** (101 MHz, $CDCl_3$): δ 131.1,

120.7, 59.8, 41.1, 38.7, 16.6, 14.5. **HRMS** (ESI) Calcd for $C_{10}H_{22}N$ $[(M-Cl)^+]$: 156.1747, found: 156.1746.

Synthesis of 1-(3,5-dimethoxyphenyl)cyclobutan-1-amine (18)

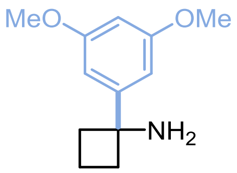

The title compound was prepared using **General Procedure C** with slight modification. The ketimine was generated in DCE from the combination of cyclobutylamine (0.10 g, 1.41 mmol) and 3,5-di-*tert*-butyl-*o*-benzoquinone **D** (0.31 g, 1.41 mmol) at 0 ˚C for 1 h. The solvent DCE was exchanged with toluene before the addition of (3,5-dimethoxyphenyl)lithium[3] which was generated from 1-bromo-3,5-dimethoxybenzene (1.83 g, 8.44 mmol, 6 equiv) and *t*BuLi (9.92 mL, 16.9 mmol, 1.7 M in pentane, 12 equiv) at -78 ˚C for 1 h in dry Et_2O (0.1 M) under argon atmosphere. The remaining procedure is same as **General Procedure C**. Yellow oil (80.0 mg, 0.39 mmol, 27% yield). **FT-IR** (thin film) ν_{max} (cm^{-1}): 2981, 1596, 1252, 1204, 1154, 840. **^{1}H NMR** (500 MHz, $CDCl_3$): δ 6.56 (d, *J* = 2.3 Hz, 2 H), 6.35 (t, *J* = 2.3 Hz, 1 H), 3.80 (s, 6 H), 3.40 – 3.02 (br s, 2 H, NH_2), 2.56 – 2.50 (m, 2 H), 2.26 – 2.08 (m, 3 H), 1.80 – 1.74 (m, 1 H). **^{13}C NMR** (126 MHz, $CDCl_3$): δ 161.1, 151.1, 103.5, 98.8, 59.6, 55.5, 36.0, 14.4. **HRMS** (ESI) Calcd for $C_{12}H_{18}NO_2$ $[(M+H)^+]$: 208.1332, found: 208.1334.

Synthesis of 1-allylcyclopentan-1-amine hydrochloride (19)

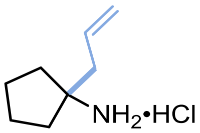

The title compound was prepared using **General Procedure B**. Yellow oil (110 mg, 0.68 mmol, 58%). **FT-IR** (thin film) ν_{max} (cm^{-1}): 3393, 2958, 2874, 1609, 1519, 1186, 994, 923. **^{1}H NMR** (400 MHz, $CDCl_3$): δ 8.31 (br s, 3 H, NH_3^+), 5.93 – 5.85 (m, 1 H), 5.25 (dd, *J* = 13.4, 9.3 Hz, 2 H), 2.55 (d, *J* = 7.0 Hz, 2 H), 2.07 – 1.86 (m, 4 H), 1.85 – 1.73 (m, 2 H), 1.72 – 1.53 (m, 2 H). **^{13}C NMR** (101 MHz, $CDCl_3$): δ 131.5, 121.2, 64.8, 42.6, 36.3, 24.1. **HRMS** (ESI) Calcd for $C_8H_{16}N$ $[(M-Cl)^+]$: 126.1277, found: 126.1277.

Synthesis of 1-allylcyclohexan-1-amine hydrochloride (20)

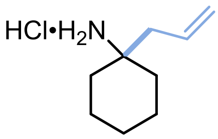

The title compound was prepared using **General Procedure B**. Pale yellow solid (76.5 mg, 0.44 mmol, 78%). **FT-IR** (thin film) ν_{max} (cm^{-1}): 3031, 2925, 2861, 1616, 1516, 1197, 999. **^{1}H NMR** (500 MHz, $CDCl_3$): δ 8.22 (br s, 3 H, NH_3^+), 5.97 – 5.90 (m, 1 H), 5.27 – 5.24 (m, 2 H), 2.54 (d, *J* = 7.1 Hz, 2 H), 1.90 – 1.66 (m, 6 H), 1.58 – 1.34 (m, 4 H). **^{13}C NMR** (126 MHz, $CDCl_3$): δ 130.8, 121.4, 57.5, 41.4, 33.8, 24.9, 21.3. **HRMS** (ESI) Calcd for $C_9H_{18}N$ [(M-Cl)$^+$]: 140.1434, found: 140.1434.

Synthesis of 1-benzylcyclohexan-1-amine (21)

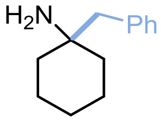

The title compound was prepared using **General Procedure A**. 10 equiv of BnMgCl (2 M in THF) was used instead of allylmagnesium bromide for the addition. Pale yellow solid (64.0 mg, 0.34 mmol, 67%). **FT-IR** (thin film) ν_{max} (cm^{-1}): 3428, 2980, 2934, 2859, 1574, 1487, 1463, 1148, 764, 704. **^{1}H NMR** (500 MHz, CD_3OD): δ 7.40 – 7.36 (m, 2 H), 7.34 – 7.30 (m, 1 H), 7.28 – 7.25 (m, 2 H), 3.00 (s, 2 H), 1.79 – 1.63 (m, 8 H), 1.59 – 1.45 (m, 2 H). **^{13}C NMR** (126 MHz, CD_3OD): δ 135.6, 131.8, 129.8, 128.6, 57.5, 43.6, 35.0, 25.9, 22.3. **HRMS** (ESI) Calcd for $C_{13}H_{20}N$ [(M+H)$^+$]: 190.1590, found: 190.1592.

Synthesis of *N*-(1-phenylcyclohexyl)benzamide (22)

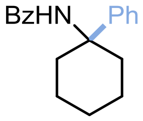

The title compound was prepared using **General Procedure C**. Commercial Phenyl lithium was used as arylating agent. The free amine was protected as benzoyl derivative using benzoyl chloride (5 equiv) with aqueous NaOH (1 M) (5 equiv). White solid (60.0 mg, 0.23 mmol, 68%). **FT-IR** (thin film) ν_{max} (cm^{-1}): 3416, 2980, 1621, 1462, 1383, 1252, 1073, 954. **^{1}H NMR** (500 MHz, $CDCl_3$): δ 7.79 (d, *J* = 7.3 Hz, 2 H), 7.52 – 7.49 (m, 1 H), 7.47 – 7.43 (m, 4 H), 7.33 (t, *J* = 7.8 Hz, 2 H), 7.22 (t, *J* = 7.3 Hz, 1 H), 6.29 (s, 1 H), 2.56 – 2.50 (m, 2 H), 1.94 – 1.88 (m, 2 H), 1.78 – 1.71 (m, 3 H), 1.68 – 1.58 (m, 2 H), 1.42 – 1.32 (m, 1 H). **^{13}C NMR** (126 MHz, $CDCl_3$): δ 166.3, 146.7, 135.8, 131.5, 128.8, 128.5, 126.9, 126.7, 125.2, 58.6, 36.4, 25.6, 22.6. **HRMS** (ESI) Calcd for $C_{19}H_{22}NO$ [(M+H)$^+$]: 280.1696, found: 280.1695.

Synthesis of 4-(*tert*-butyl)-1-phenylcyclohexan-1-amine (23)

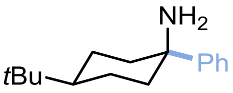

23 (dr=**4.26**:1)
(trans:cis)

The title compounds (**23** and **23'**) were prepared using **General Procedure C**. Commercial Phenyl lithium was used as arylating agent. dr=4.26:1 (determined using crude ^{1}H NMR). White solid (756 mg, 3.27 mmol, 51%). **FT-IR** (thin film) ν_{max} (cm^{-1}): 2968, 2867, 1600, 1445, 1239, 757, 698. **^{1}H NMR** (400 MHz, $CDCl_3$): δ 7.59 – 7.52 (m, 2 H), 7.40 – 7.30 (m, 2 H), 7.28 – 7.18 (m, 1 H), 1.89 – 1.68 (m, 8 H), 1.51 – 1.39 (m, 2 H), 1.08 (tt, *J* = 12.2, 3.2 Hz, 1 H), 0.93 (s, 9 H). **^{13}C NMR** (101 MHz, $CDCl_3$): δ: 150.9, 128.2, 126.3, 124.9, 53.3, 47.8, 39.7, 32.5, 27.7, 23.0. **HRMS** (ESI) Calcd for $C_{16}H_{26}N$ [$(M+H)^+$]: 232.2060, found: 232.2063.

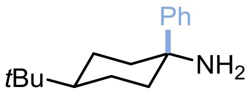

23' (dr=4.26:**1**)
(trans:cis)

Yellow oil (180 mg, 0.78 mmol, 12%). **FT-IR** (thin film) ν_{max} (cm^{-1}): 2939, 2864, 1601, 1496, 1206, 768, 699. **^{1}H NMR** (400 MHz, $CDCl_3$): δ 7.50 – 7.42 (m, 2 H), 7.34 (dd, *J* = 8.5, 7.0 Hz, 2 H), 7.25 – 7.17 (m, 1 H), 2.57 – 2.44 (m, 2 H), 2.15 (br s, 2 H, NH_2), 1.68 – 1.54 (m, 4 H), 1.11 (tt, *J* = 11.9, 3.1 Hz, 1 H), 1.04 – 0.90 (m, 2 H), 0.74 (s, 9 H). **^{13}C NMR** (101 MHz, $CDCl_3$): δ 145.7, 128.7, 126.5, 126.2, 54.5, 48.1, 39.5, 32.3, 27.6, 24.5. **HRMS** (ESI) Calcd for $C_{16}H_{26}N$ [$(M+H)^+$]: 232.2060, found: 232.2061.

Synthesis of 1-allylcycloheptan-1-amine hydrochloride (24)

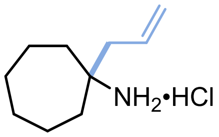

The title compound was prepared using **General Procedure B**. Off-white solid (356 mg, 1.88 mmol, 61%). **FT-IR** (thin film) ν_{max} (cm^{-1}): 2981, 2929, 1614, 1514, 1252, 955. **^{1}H NMR** (400 MHz, $CDCl_3$): δ 8.30 (br s, 3 H, NH_3^+), 5.96 (ddt, *J* = 16.4, 10.3, 7.4 Hz, 1 H), 5.28 – 5.23 (m, 2 H), 2.49 (d, *J* = 7.4 Hz, 2 H), 2.00 – 1.89 (m, 2 H), 1.89 – 1.76 (m, 4 H), 1.71 – 1.59 (m, 2 H), 1.56 – 1.39 (m, 4 H). **^{13}C NMR** (101 MHz, $CDCl_3$): δ 131.2, 121.4, 61.0, 44.1, 37.7, 29.9, 22.3. **HRMS** (ESI) Calcd for $C_{10}H_{20}N$ [$(M-Cl)^+$]: 154.1590, found: 154.1590.

Synthesis of 1-allylcyclooctan-1-amine hydrochloride (25)

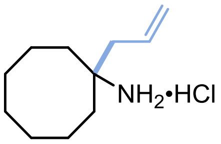

The title compound was prepared using **General Procedure A**. The free amine was protected as HCl salt using 4 M HCl in dioxane. The cleavage of phenol unit was performed by using 2 equiv of I_2 and 5

equiv NaOH (1 M). Pale yellow solid (993 mg, 4.87 mmol, 62%). **FT-IR** (thin film) ν_{max} (cm^{-1}): 2980, 2916, 1641, 1517, 1253, 920, 731. **^{1}H NMR** (400 MHz, $CDCl_3$): δ 8.25 (br s, 3 H, NH_3^+), 6.07 – 5.97 (m, 1 H), 5.29 – 5.22 (m, 2 H), 2.47 (d, *J* = 7.4 Hz, 2 H), 2.08 – 2.02 (m, 2 H), 1.83 – 1.62 (m, 6 H), 1.61 – 1.49 (m, 4 H), 1.48 – 1.35 (m, 2 H). **^{13}C NMR** (101 MHz, $CDCl_3$): δ 131.2, 121.1, 60.8, 43.0, 33.2, 28.1, 24.9, 22.1. **HRMS** (ESI) Calcd for $C_{11}H_{22}N$ [$(M-Cl)^+$]: 168.1747, found: 168.1745.

Synthesis of 3-allyltetrahydrofuran-3-amine (26)

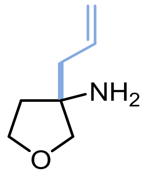

The title compound was prepared using **General Procedure A**. The cleavage of phenol unit was performed by using 2 equiv of I_2 and 5 equiv NaOH (1 M). Yellow oil (141 mg, 1.11 mmol, 88%). **FT-IR** (thin film) ν_{max} (cm^{-1}): 3349, 2928, 2866, 1640, 1440, 1220, 1054, 913. **^{1}H NMR** (400 MHz, $CDCl_3$): δ 5.80 (ddt, *J* = 19.9, 9.2, 7.4 Hz, 1 H), 5.15 – 5.11 (m, 2 H), 3.97 (dd, *J* = 16.0, 8.0 Hz, 1 H), 3.85 (td, *J* = 8.7, 4.7 Hz, 1 H), 3.56 (d, *J* = 8.7 Hz, 1 H), 3.48 (d, *J* = 8.7 Hz, 1 H), 2.29 (d, *J* = 7.4 Hz, 2 H), 1.91 (dt, *J* = 12.4, 8.3 Hz, 1 H), 1.82 (br s, 2 H, NH_2), 1.72 (ddd, *J* = 12.5, 7.6, 4.7 Hz, 1 H). **^{13}C NMR** (101 MHz, $CDCl_3$): δ 133.8, 119.1, 79.2, 67.5, 60.5, 44.0, 40.0. **HRMS** (ESI) Calcd for $C_7H_{14}NO$ [$(M+H)^+$]: 128.1070, found: 128.1069.

Synthesis of 4-allyltetrahydro-2*H*-pyran-4-amine hydrochloride (27)

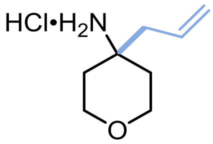

The title compound was prepared using **General Procedure B**. Off-white powder (139 mg, 0.78 mmol, 79%). **FT-IR** (thin film) ν_{max} (cm^{-1}): 3390, 2980, 1640, 1524, 1237, 1157, 936, 839. **^{1}H NMR** (400 MHz, $CDCl_3$): δ 8.56 (br s, 3 H, NH_3^+), 5.94 (ddt, *J* = 17.5, 10.3, 7.4 Hz, 1 H), 5.44 – 5.14 (m, 2 H), 3.96 (ddd, *J* = 11.9, 8.0, 3.6 Hz, 2 H), 3.70 (ddd, *J* = 12.4, 6.2, 4.1 Hz, 2 H), 2.61 (d, *J* = 7.4 Hz, 2 H), 2.00 – 1.81 (m, 4 H). **^{13}C NMR** (101 MHz, $CDCl_3$): δ 129.8, 122.2, 62.8, 55.0, 41.8, 33.8. **HRMS** (ESI) Calcd for $C_8H_{16}NO$ [$(M-Cl)^+$]: 142.1226, found: 142.1225.

Synthesis of 4-allyltetrahydro-2*H*-thiopyran-4-amine (28)

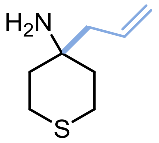

The title compound was prepared using **General Procedure B**. The HCl salt of compound **28** was basified with 1 M NaOH (1 mL), extracted with $CHCl_3$ (3 x 20 mL) and dried by Na_2SO_4. After removal of solvents gave the desired 4-allyltetrahydro-2*H*-thiopyran-4-amine **28** as yellow oil (99.0 mg, 0.63 mmol, 67%). **FT-IR** (thin film) ν_{max} (cm^{-1}): 3336, 3073, 2925, 1638, 1437, 1273, 998, 916. **^{1}H NMR** (400 MHz, $CDCl_3$): δ 5.81 (ddt, *J* = 16.9, 10.2, 7.5 Hz, 1 H), 5.18 – 5.05 (m, 2 H), 2.89 – 2.77 (m, 2 H), 2.55 – 2.41 (m, 2 H), 2.13 (dt, *J* = 7.5, 1.1 Hz, 2 H), 2.01 – 1.90 (br s, 2 H, NH_2), 1.81 – 1.67 (m, 4 H). **^{13}C NMR** (101 MHz, $CDCl_3$): δ 133.0, 119.1, 49.7, 48.0, 39.2, 24.2. **HRMS** (ESI) Calcd for $C_8H_{16}NS$ [$(M+H)^+$]: 158.0998, found: 158.0999.

Synthesis of 2-(4-allyl-4-aminopiperidin-1-yl)-4,6-di-*tert*-butylphenol (29)

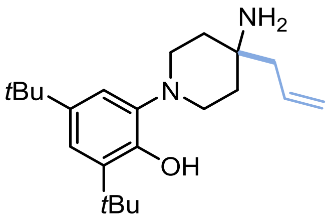

The title compound was prepared using **General Procedure A** starting from *tert*-butyl 4-aminopiperidine-1-carboxylate. The Boc protection was cleaved during oxidation and the free amine 4-allylpiperidin-4-amine was reactive enough to undergo a self-condensation with the formed *o*-quinone, which ends up with the product **29**[5] as a yellow oil (76.0 mg, 0.22 mmol, 44%). **FT-IR** (thin film) ν_{max} (cm^{-1}): 3389, 2958, 1640, 1480, 1248, 967. **^{1}H NMR** (400 MHz, CD_3OD): δ 7.01 (d, *J* = 2.3 Hz, 1 H), 6.93 (d, *J* = 2.3 Hz, 1 H), 5.96 (ddt, *J* = 17.5, 10.3, 7.3 Hz, 1 H), 5.22 – 5.15 (m, 2 H), 3.35 – 3.29 (m, 2 H), 3.16 (ddd, *J* = 12.9, 6.9, 4.0 Hz, 2 H), 2.50 (d, *J* = 7.3 Hz, 2 H), 2.01 – 1.93 (m, 2 H), 1.83 (ddd, *J* = 14.1, 9.0, 3.9 Hz, 2 H), 1.39 (s, 9 H), 1.28 (s, 9 H). **^{13}C NMR** (101 MHz, CD_3OD): δ 148.9, 142.7, 137.2, 134.1, 132.5, 120.6, 119.7, 119.5, 54.6, 43.3, 41.6, 35.8, 35.1, 32.9, 32.1, 30.2. **HRMS** (ESI) Calcd for $C_{22}H_{37}N_2O$ [$(M+H)^+$]: 345.2900, found: 345.2898.

Synthesis of *tert*-butyl 4-allyl-4-aminopiperidine-1-carboxylate hydrochloride (30)

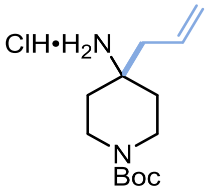

The title compound was prepared using **General Procedure B**. The cleavage of phenol unit was performed by adding 1.2 equiv of pyrrolidine along with I_2 (1.2 equiv) and NaOH (1 M) (5 equiv) to suppress the self-condensation of 4-allylpiperidin-4-amine with the liberated *o*-quinone. The HCl salt of compound **30** was selectively Boc protected on the secondary amine using Boc_2O (2 equiv) and K_2CO_3 (3 equiv) in THF:H_2O (2:1 v:v)(0.4 M) at 25 ˚C for 24 h. The crude product was purified by column chromatography on silica gel using pentane/EtOAc (80:20 v:v) and followed by EtOAc:MeOH:Et_3N (70:20:10 v:v) as solvent system to afford the Boc protected free amine **30** (82.0 mg, 0.38 mmol, 68%) as a brown oil and the free amine was protected as HCl salt using 4 M HCl (excess) in dioxane to give a yellow solid. **FT-IR** (thin film) ν_{max} (cm^{-1}): 3398, 2928, 1693, 1526 1479, 1249, 1164. **^{1}H NMR** (500 MHz, $CDCl_3$): δ 8.53 (br s, 3 H, NH_3^+), 5.97 – 5.87 (m, 1 H), 5.34 – 5.24 (m, 2 H), 3.70 – 3.49 (m, 4 H), 2.57 (d, *J* = 7.0 Hz , 2 H), 1.95 – 1.71 (m, 4 H), 1.45 (s, 9 H). **^{13}C NMR** (126 MHz, $CDCl_3$): δ 154.5, 129.7, 122.3, 80.3, 56.0, 41.6, 39.0, 33.2, 28.5. **HRMS** (ESI) Calcd for $C_{13}H_{25}N_2O_2$ [$(M-Cl)^+$]: 241.1911, found: 241.1913.

Synthesis of 2-(pyridin-4-yl)pent-4-en-2-amine (31)

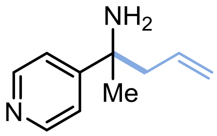

The title compound was prepared using **General Procedure B**. The HCl salt of compound **31** was basified with 1 M NaOH (1 mL), extracted with $CHCl_3$ (3 x 20 mL) and dried with Na_2SO_4. After removal of solvents gave the desired 2-(pyridin-4-yl)pent-4-en-2-amine **31** as a pale-yellow oil (100 mg, 0.62 mmol, 75%). **FT-IR** (thin film) ν_{max} (cm^{-1}): 3349, 3077, 2921, 2850, 1640, 1439, 1279, 1000, 922, 825. **^{1}H NMR** (400 MHz, $CDCl_3$): δ 8.55 (d, *J* = 6.3 Hz, 2 H), 7.37 (d, *J* = 6.3 Hz, 2 H), 5.57 – 5.46 (m, 1 H), 5.10 – 5.05 (m, 2 H), 2.54 (ddt, *J* = 13.6, 6.7, 1.3 Hz, 1 H), 2.39 (ddt, *J* = 13.6, 8.0, 1.1 Hz, 1 H), 1.91 (br s, 2 H, NH_2), 1.45 (s, 3 H). **^{13}C NMR** (101 MHz, $CDCl_3$): δ 157.7, 149.8, 133.3, 120.8, 119.6, 54.6, 49.2, 30.5. **HRMS** (ESI) Calcd for $C_{10}H_{15}N_2$ [$(M+H)^+$]: 163.1230, found: 163.1230.

Synthesis of 2-(4-methoxyphenyl)propan-2-amine (32)

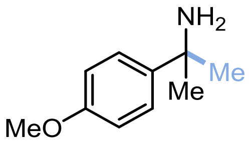

The title compound was prepared using **General Procedure C**. Commercial methyllithium solution (1.6 M in diethyl ether) was used. Yellow oil (94.0 mg, 0.57 mmol, 86%). **FT-IR** (thin film) ν_{max} (cm^{-1}): 3353, 2962, 2836, 1610, 1512, 1245, 1180, 1032, 830. **^{1}H NMR** (400 MHz, $CDCl_3$): δ 7.42 (d, *J* = 8.9 Hz, 2 H), 6.85 (d, *J* = 8.9 Hz, 2 H), 3.78 (s, 3 H), 1.96 (br s, 2 H, NH_2), 1.48 (s, 6 H). **^{13}C NMR** (101 MHz, $CDCl_3$): δ 158.0, 142.3, 125.9, 113.5, 55.2, 52.1, 32.8. **HRMS** (ESI) Calcd for $C_{10}H_{13}O$ [M-NH_2]: 149.0961, found: 149. 0960.

Synthesis of 2-(4-methoxyphenyl)hexan-2-amine (33)

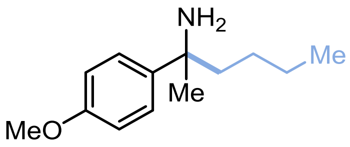

The title compound was prepared using **General Procedure C**. Commercial *n*-butyllithium solution (2.5 M in hexanes) was used. Yellow oil (107 mg, 0.52 mmol, 78%). **FT-IR** (thin film) ν_{max} (cm^{-1}): 3396, 2956, 2930, 2860, 1611, 1510, 1246, 1180, 1135, 829. **^{1}H NMR** (400 MHz, $CDCl_3$): δ 7.35 (d, *J* = 8.9 Hz, 2 H), 6.85 (d, *J* = 8.9 Hz, 2 H), 3.79 (s, 3 H), 1.89 (br s, 2 H, NH_2), 1.77 – 1.63 (m, 2 H), 1.44 (s, 3 H), 1.29 – 1.02 (m, 4 H), 0.83 (t, *J* = 7.3 Hz, 3 H). **^{13}C NMR** (101 MHz, $CDCl_3$): δ 157.8, 141.0, 126.4, 113.4, 55.3, 54.7, 45.1, 31.1, 26.7, 23.2, 14.1. **HRMS** (ESI) Calcd for $C_{13}H_{19}O$ [M-NH_2]: 191.1430, found: 191.1431.

Synthesis of 2-(4-methoxyphenyl)-3-methylpentan-2-amine (34)

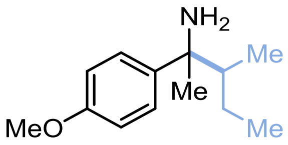

The title compound was prepared using **General Procedure C**. Commercial *sec*-butyllithium solution (1.4 M in cyclohexane) was used. dr = 1:1, yellow oil (99.0 mg, 0.48 mmol, 72%). **FT-IR** (thin film) ν_{max} (cm^{-1}): 3390, 2965, 2931, 1610, 1510, 1247, 1180, 1136, 831. **^{1}H NMR** (400 MHz, $CDCl_3$): δ 7.35 (d, *J* = 8.9 Hz, 4 H), 6.85 (d, *J* = 8.9 Hz, 4 H), 3.79 (s, 6 H), 1.66 – 1.56 (m, 2 H), 1.55 – 1.41 (m, 6 H), 1.40 (s, 3 H), 1.37 (s, 3 H), 0.93 – 0.80 (m, 11 H), 0.78 (d, *J* = 6.7 Hz, 3 H). **^{13}C NMR** (101 MHz, $CDCl_3$): δ 157.8 (2

x C), 141.8, 141.6, 126.8, 126.7, 113.3, 113.2, 57.6, 57.4, 55.3 (2 x OCH_3), 46.4, 46.3, 28.1, 27.5, 24.3, 24.2, 13.7, 13.6, 13.0 (2 x CH_3). **HRMS** (ESI) Calcd for $C_{13}H_{19}O$[M-NH_2]: 191.1430, found: 191.1430.

Synthesis of 2-(4-methoxyphenyl)-3,3-dimethylbutan-2-amine hydrochloride (35)

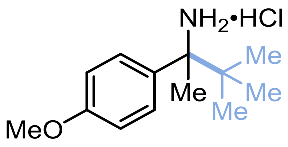

The title compound was prepared using **General Procedure C**. Commercial *tert*-butyllithium solution (1.7 M in pentane) was used. The free amine was protected as HCl salt using 4 M HCl (excess) in dioxane. Yellow solid (96.0 mg, 0.46 mmol, 70%). **FT-IR** (thin film) ν_{max} (cm^{-1}): 2980, 2889, 1610, 1515, 1256, 831, 729. **^{1}H NMR** (400 MHz, $CDCl_3$): δ 7.37 (d, *J* = 8.9 Hz, 2 H), 6.78 (d, *J* = 8.9 Hz, 2 H), 3.74 (s, 3 H), 1.80 (s, 3 H), 1.01 (s, 9 H). **^{13}C NMR** (101 MHz, $CDCl_3$): δ 158.7, 131.0, 128.5, 113.0, 65.4, 55.2, 37.4, 26.1, 21.4. **HRMS** (ESI) Calcd for $C_{13}H_{22}NO[(M-Cl)^+]$: 208.1696, found: 208.1697.

Synthesis of 2-(4-methoxyphenyl)but-3-en-2-amine hydrochloride (36)

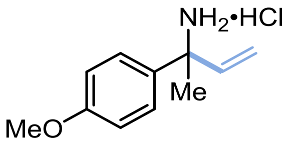

The title compound was prepared using **General Procedure C**. Vinyl lithium was generated using the known literature method[6] in brief, in a Schlenk tube under an argon atmosphere, *n*-butyllithium (3.17, 7.94 mmol. 2.5 M in hexanes) was added dropwise to the neat tetravinyl stannane (0.72 mL, 3.97 mmol) over the course of 5 min at 25 ˚C. A white precipitate has formed during the addition of *n*-butyllithium. After letting it settle, the supernatant solution was collected through syringe and added to the ketimine/hemiaminal and TMEDA mixture at 0 ˚C. The reaction was gradually warmed to 25 ˚C and continued the stirring for 24 h. The remaining procedure is same as **General Procedure C**. 10 equiv of NaOH (1.0 M) was used during oxidation. The free amine was protected as HCl salt using 4 M HCl (0.5 mL) in dioxane. Yellow solid (107 mg, 0.50 mmol, 76%). **FT-IR** (thin film) ν_{max} (cm^{-1}): 3387, 2980, 1609, 1514, 1255, 1188, 1029, 830. **^{1}H NMR** (400 MHz, $CDCl_3$): δ 9.01 (br s, 3 H, NH_3^+), 7.45 (d, *J* = 8.9 Hz, 2 H), 6.84 (d, *J* = 8.9 Hz, 2 H), 6.02 (dd, *J* = 17.4, 10.9 Hz, 1 H), 5.40 – 5.23 (m, 2 H), 3.77 (s, 3 H), 1.82 (s, 3 H). **^{13}C NMR** (101 MHz, $CDCl_3$): δ 159.5, 139.0, 131.3, 127.8, 116.2, 114.0, 60.1, 55.3, 25.3. **HRMS** (ESI) Calcd for $C_{11}H_{13}O$ [M-NH_3Cl]: 161.0961, found: 161.0961.

Synthesis of 1-(4-methoxyphenyl)-1-phenylethan-1-amine (37)

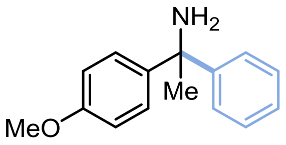

The title compound was prepared using **General Procedure C**. Commercial PhLi (1.9 M in dibutyl ether) was used. Yellow oil (123 mg, 0.54 mmol, 82%). **FT-IR** (thin film) ν_{max} (cm^{-1}): 2967, 2835, 1609, 1509, 1248, 1180, 1030, 832, 701. **^{1}H NMR** (400 MHz, $CDCl_3$): δ 7.39 – 7.36 (m, 2 H), 7.32 – 7.27 (m, 4 H), 7.23 – 7.19 (m, 1 H), 6.83 (d, *J* = 8.9 Hz, 2 H), 3.79 (s, 3 H), 2.31 (br s, 2 H, NH_2), 1.85 (s, 3 H). **^{13}C NMR** (101 MHz, $CDCl_3$): δ 158.2, 149.9, 141.9, 128.2, 127.5, 126.5, 126.2, 113.5, 58.3, 55.4, 32.0. **HRMS** (ESI) Calcd for $C_{15}H_{15}O$ [M-NH_2]: 211.1117, found: 211.1116.

Synthesis of 2-(4-methoxyphenyl)-1-phenylpropan-2-amine (38)

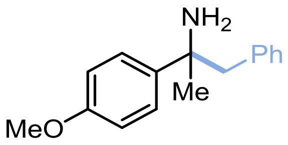

The title compound was prepared using **General Procedure A**. 10 equiv of BnMgCl (2 M in THF) was used instead of allylmagnesium bromide for the addition. Yellow oil (101 mg, 0.42 mmol, 63%). **FT-IR** (thin film) ν_{max} (cm^{-1}): 3028, 2930, 2835, 1610, 1511, 1247, 1181, 1033, 831, 703. **^{1}H NMR** (400 MHz, $CDCl_3$): δ 7.32 (d, *J* = 8.9 Hz, 2 H), 7.21 – 7.17 (m, 3 H), 6.94 – 6.90 (m, 2 H), 6.86 (d, *J* = 8.9 Hz, 2 H), 3.81 (s, 3 H), 3.00 (d, *J* = 13.0 Hz, 1 H), 2.94 (d, *J* = 13.0 Hz, 1 H), 1.51 (br s, 2 H, NH_2), 1.48 (s, 3 H). **^{13}C NMR** (101 MHz, $CDCl_3$): δ 158.1, 140.8, 137.8, 130.7, 127.9, 126.7, 126.4, 113.4, 55.4, 55.2, 51.9, 30.8. **HRMS** (ESI) Calcd for $C_{16}H_{17}O$ [M-NH_2]: 225.1274, found: 225.1273.

Synthesis of 1-(4-butylphenyl)-1-(4-methoxyphenyl)ethan-1-amine (39)

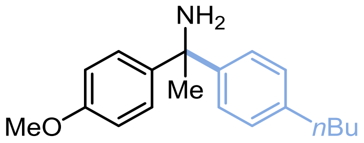

The title compound was prepared using **General Procedure C**. 1-bromo-4-butylbenzene was used for lithium-halogen exchange[3] in THF at -78 ˚C for 1 h. Yellow oil (125 mg, 0.44 mmol, 67%). **FT-IR** (thin film) ν_{max} (cm^{-1}): 3023, 2956, 2929, 2857, 1609, 1510, 1249, 1181, 1034, 831. **^{1}H NMR** (400 MHz, $CDCl_3$): δ 7.31 – 7.25 (m, 4 H), 7.10 (d, J = 8.90 Hz, 2 H), 6.83 (d, J = 8.9 Hz, 2 H), 3.79 (s, 3 H), 2.58 (t, *J* = 7.8 Hz, 2 H), 2.01 (br s, 2 H, NH_2), 1.82 (s, 3 H), 1.61 – 1.54 (m, 2 H), 1.38 – 1.33 (m, 2 H), 0.92 (t, J = 7.3 Hz, 3 H). **^{13}C NMR** (101 MHz, $CDCl_3$): δ 158.1, 147.2, 142.1, 141.0, 128.2, 127.4, 126.1, 113.5,

58.0, 55.3, 35.3, 33.7, 32.1, 22.6, 14.1. **HRMS** (ESI) Calcd for $C_{19}H_{23}O$ [M-NH_2]: 267.1743, found: 267.1743.

Synthesis of 4-(1-amino-1-(4-methoxyphenyl)ethyl)-*N*,*N*-dimethylaniline (40)

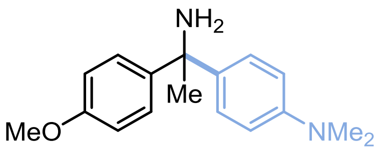

The title compound was prepared using **General Procedure C**. 4-bromo-*N*,*N*-dimethylaniline was used for lithium-halogen exchange[3] in THF at -78 ˚C for 1 h. 3 equiv of iodine was used as oxidant. Brown oil (95.0 mg, 0.35 mmol, 53%). **FT-IR** (thin film) ν_{max} (cm^{-1}): 3310, 2929, 2910, 2863, 1609, 1509, 1245, 1178, 1032, 815. **^{1}H NMR** (400 MHz, CD_2Cl_2): δ 7.30 (d, *J* = 8.9 Hz, 2 H), 7.21 (d, *J* = 8.8 Hz, 2 H), 6.81 (d, *J* = 8.9 Hz, 2 H), 6.66 (d, *J* = 8.8 Hz, 2 H), 3.77 (s, 3 H), 2.91 (s, 6 H), 1.92 (br s, 2 H, NH_2), 1.77 (s, 3 H). **^{13}C NMR** (101 MHz, CD_2Cl_2): δ 158.2, 149.5, 143.3, 138.7, 127.6, 127.1, 113.5, 112.5, 57.6, 55.5, 40.8, 32.5. **HRMS** (ESI) Calcd for $C_{17}H_{20}NO$ [M-NH_2]: 254.1539, found: 254.1541.

Synthesis of 1,1-bis(4-methoxyphenyl)ethan-1-amine (41)

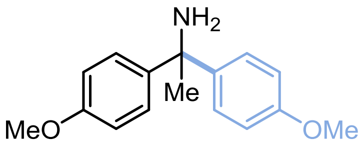

The title compound was prepared using **General Procedure C**. 1-bromo-4-methoxybenzene was used for lithium-halogen exchange[3] in THF at -78 ˚C for 1 h. 2 equiv of iodine was used as oxidant. Yellow oil (86.0 mg, 0.33 mmol, 51%). **FT-IR** (thin film) ν_{max} (cm^{-1}): 3035, 2934, 2836, 1609, 1510, 1246, 1181, 1029, 1008, 830, 758. **^{1}H NMR** (400 MHz, $CDCl_3$): δ 7.29 (d, *J* = 8.9 Hz, 4 H), 6.83 (d, *J* = 8.9 Hz, 4 H), 3.79 (s, 6 H), 2.73 (br s, 2 H, NH_2, 1.83 (s, 3 H). **^{13}C NMR** (101 MHz, $CDCl_3$): δ 158.1, 141.7, 127.4, 113.5, 58.0, 55.3, 32.0. **HRMS** (ESI) Calcd for $C_{16}H_{17}O_2$ [M-NH_2]: 241.1223, found: 241.1221.

Synthesis of 1-(3,5-dimethoxyphenyl)-1-(4-methoxyphenyl)ethan-1-amine (42)

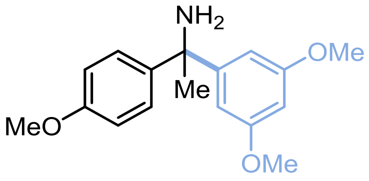

The title compound was prepared using **General Procedure C**. 1-bromo-3,5-dimethoxybenzene was used for lithium-halogen exchange[3] in THF at -78 ˚C for 1 h. Yellow oil (123 mg, 0.43 mmol, 65%). **FT-**

IR (thin film) ν_{max} (cm^{-1}): 3315, 2935, 2836, 1595, 1456, 1247, 1181, 1034, 832. ^{1}**H NMR** (400 MHz, $CDCl_3$): δ 7.29 (d, *J* = 8.9 Hz, 2 H), 6.82 (d, *J* = 8.9 Hz, 2 H), 6.53 (d, *J* = 2.3 Hz, 2 H), 6.32 (t, *J* = 2.3 Hz, 1 H), 3.79 (s, 3 H), 3.75 (s, 6 H), 1.98 (br s, 2 H, NH_2), 1.80 (s, 3 H). ^{13}**C NMR** (101 MHz, $CDCl_3$): δ 160.6, 158.1, 152.9, 141.8, 127.3, 113.5, 104.9, 97.9, 58.3, 55.4, 55.3, 32.1. **HRMS** (ESI) Calcd for $C_{17}H_{19}O_3$ [M-NH_2]: 271.1329, found: 271.1329.

Synthesis of 1-(4-methoxyphenyl)-1-(4-(methylthio)phenyl)ethan-1-amine (43)

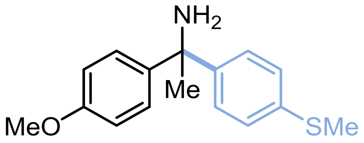

The title compound was prepared using **General Procedure C**. (4-bromophenyl)(methyl)sulfane was used for lithium-halogen exchange[3] in THF at -78 ˚C for 1.5 h. 5 equiv of iodine was used as oxidant. Yellow oil (113 mg, 0.41 mmol, 63%). **FT-IR** (thin film) ν_{max} (cm^{-1}): 3300, 2965, 2837, 1608, 1510, 1247, 1181, 1031, 814, 782. ^{1}**H NMR** (400 MHz, $CDCl_3$): δ 7.30 (dd, *J* = 8.7, 5.5 Hz, 4 H), 7.19 (d, *J* = 8.9 Hz, 2 H), 6.83 (d, *J* = 8.9 Hz, 2 H), 3.79 (s, 3 H), 3.49 (br s, 2 H, NH_2), 2.47 (s, 3 H), 1.85 (s, 3 H). ^{13}**C NMR** (101 MHz, $CDCl_3$): δ 158.3, 146.1, 140.8, 136.5, 127.4, 126.8, 126.5, 113.5, 58.4, 55.3, 31.5, 15.9. **HRMS** (ESI) Calcd for $C_{16}H_{17}OS$ [M-NH_2]: 257.0995, found: 257.0995.

Synthesis of 1-(benzo[*d*][1,3]dioxol-5-yl)-1-(4-methoxyphenyl)ethan-1-amine (44)

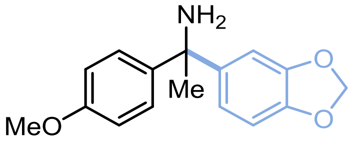

The title compound was prepared using **General Procedure C**. 5-bromobenzo[*d*][1,3]dioxole was used for lithium-halogen exchange[3] in THF at -78 ˚C for 1 h. 2 equiv of iodine was used as oxidant. Yellow oil (101 mg, 0.37 mmol, 56%). **FT-IR** (thin film) ν_{max} (cm^{-1}): 3356, 2980, 2890, 1609, 1485, 1237, 1180, 1035, 864, 833. ^{1}**H NMR** (500 MHz, $CDCl_3$): δ 7.29 (d, *J* = 8.9 Hz, 2 H), 6.87 (dd, *J* = 8.1, 2.0 Hz, 1 H), 6.84 – 6.82 (m, 3 H), 6.72 (d, *J* = 8.1 Hz, 1 H), 5.91 (s, 2 H), 3.79 (s, 3 H), 2.02 (br s, 2 H, NH_2), 1.80 (s, 3 H). ^{13}**C NMR** (126 MHz, $CDCl_3$): δ 158.2, 147.6, 146.0, 144.3, 142.0, 127.3, 119.1, 113.5, 107.7, 107.5, 101.1, 58.1, 55.4, 32.3. **HRMS** (ESI) Calcd for $C_{16}H_{15}O_3$ [M-NH_2]: 255.1016, found: 255.1016.

Synthesis of 1-(4-fluorophenyl)-1-(4-methoxyphenyl)ethan-1-amine (45)

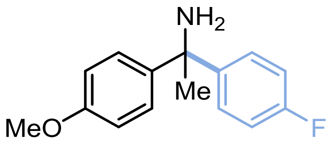

The title compound was prepared using **General Procedure C**. 1-bromo-4-fluorobenzene was used for lithium-halogen exchange[4] in Et_2O at -78 ˚C for 30 min. Yellow oil (70.0 mg, 0.29 mmol, 43%). **FT-IR** (thin film) ν_{max} (cm^{-1}): 3062, 2927, 2836, 1603, 1509, 1249, 1033, 833. **^{1}H NMR** (400 MHz, $CDCl_3$): δ 7.26 (dd, *J* = 8.9, 5.3 Hz, 2 H), 7.19 (d, *J* = 8.9 Hz, 2 H), 6.88 (t, *J* = 8.7 Hz, 2 H), 6.76 (d, *J* = 8.9 Hz, 2 H), 3.72 (s, 3 H), 1.80 (br s, 2 H, NH_2), 1.74 (s, 3 H). **^{19}F NMR** (377 MHz, $CDCl_3$): δ -117.2. **^{13}C NMR** (101 MHz, $CDCl_3$): δ 161.5 (d, $J_{C\text{-}F}$ = 245.7 Hz), 158.2, 146.0 (d, $J_{C\text{-}F}$ = 3.2 Hz), 142.0, 127.9 (d, $J_{C\text{-}F}$ = 8.0 Hz), 127.3, 114.8 (d, $J_{C\text{-}F}$ = 20.8 Hz), 113.6, 57.8, 55.4, 32.4. **HRMS** (ESI) Calcd for $C_{15}H_{14}OF$[M-NH_2]: 229.1023, found: 229.1026.

Synthesis of 1-(4-chlorophenyl)-1-(4-methoxyphenyl)ethan-1-amine (46)

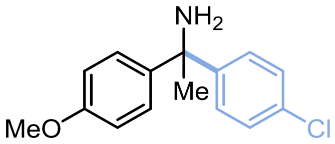

The title compound was prepared using **General Procedure C**. 1-chloro-4-iodobenzene was used for lithium-halogen exchange[4] in Et_2O at -78 ˚C for 1 h. Yellow oil (100 mg, 0.38 mmol, 58%). **FT-IR** (thin film) ν_{max} (cm^{-1}): 3310, 2962, 2835, 1608, 1509, 1247, 1180, 1033, 1012, 829. **^{1}H NMR** (400 MHz, $CDCl_3$): δ 7.30 (d, *J* = 8.9 Hz, 2 H), 7.25 – 7.21 (m, 4 H), 6.81 (d, *J* = 8.9 Hz, 2 H), 3.77 (s, 3 H), 1.84 (br s, 2 H, NH_2), 1.79 (s, 3 H). **^{13}C NMR** (101 MHz, $CDCl_3$): δ 158.3, 148.8, 141.7, 132.2, 128.3, 127.8, 127.3, 113.6, 57.8, 55.4, 32.2. **HRMS** (ESI) Calcd for $C_{15}H_{14}O^{35}Cl$ [M-NH_2]: 245.0728, found: 245.0729.

Synthesis of 1-(4-methoxyphenyl)-1-(4-(trifluoromethyl)phenyl)ethan-1-amine (47)

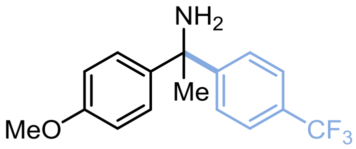

The title compound was prepared using **General Procedure C**. 1-iodo-4-(trifluoromethyl)benzene was used for lithium-halogen exchange[4] in Et_2O at -78 ˚C for 1 h. Brown oil (129 mg, 0.44 mmol, 66%). **FT-IR** (thin film) ν_{max} (cm^{-1}): 2967, 2840, 2812, 1615, 1510, 1325, 1250, 1117, 832. **^{1}H NMR** (400 MHz, $CDCl_3$): δ 7.55 – 7.49 (m, 4 H), 7.27 (d, *J* = 8.9 Hz, 2 H), 6.84 (d, *J* = 8.9 Hz, 2 H), 3.79 (s, 3 H), 1.92 (br s, 2 H, NH_2), 1.84 (s, 3 H). **^{19}F NMR** (377 MHz, $CDCl_3$): δ -62.4. **^{13}C NMR** (101 MHz, $CDCl_3$): δ 158.4,

154.2, 141.3, 128.6 (q, $J_{C\text{-}F}$ = 32.5 Hz), 127.4, 126.6, 125.2 (q, $J_{C\text{-}F}$ = 3.7 Hz), 124.4 (q, $J_{C\text{-}F}$ = 272.9 Hz), 113.7, 58.1, 55.4, 32.1. **HRMS** (ESI) Calcd for $C_{16}H_{14}F_3O$ [M-NH_2]: 279.0991, found: 279.0991.

Synthesis of 2-amino-2-phenylpropanenitrile (48)

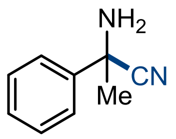

The title compound was prepared using **general method D**. Colourless oil (45.0 mg, 0.31 mmol, 95%). **FT-IR** (thin film) ν_{max} (cm^{-1}): 3376, 2980, 2905, 2225, 1682, 1448, 1202, 881, 764, 699. **^{1}H NMR** (400 MHz, $CDCl_3$): δ 7.75 – 7.59 (m, 2 H), 7.45 – 7.32 (m, 3 H), 2.10 (br s, 2 H, NH_2), 1.77 (s, 3 H). **^{13}C NMR** (101 MHz, $CDCl_3$): δ 141.3, 129.0, 128.8, 125.0, 124.3, 53.8, 31.9. **HRMS** (ESI) Calcd for $C_8H_{10}N$ [M-CN]: 120.0808, found: 120.0808.

Synthesis of 2-amino-2-(4-methoxyphenyl)propanenitrile (49)

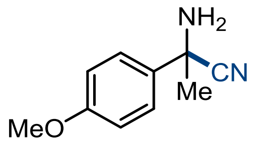

The title compound was prepared using **general method D**. The crude product was purified by column chromatography on neutralized silica gel (treated with Et_3N prior to silica column chromatography) eluting with (pentane/EtOAc = 80:20 v:v) to EtOAc:MeOH:Et_3N (70:20:10 v:v) to afford compound **49** as yellow oil (100 mg, 0.57 mmol, 86%). **FT-IR** (thin film) ν_{max} (cm^{-1}): 3376, 2933, 2839, 2235, 1606, 1510, 1248, 1178, 1029, 831. **^{1}H NMR** (400 MHz, CD_3CN): δ 7.55 (d, J = 8.9 Hz, 2 H), 6.95 (d, J = 8.9 Hz, 2 H), 3.79 (s, 3 H), 2.18 (br s, 2 H, NH_2), 1.67 (s, 3 H). **^{13}C NMR** (101 MHz, $CDCl_3$): δ 159.9, 133.4, 126.4, 124.5, 114.2, 55.5, 53.3, 32.0. **HRMS** (ESI) Calcd for $C_9H_{12}NO$ [M-CN]: 150.0913, found: 150.0913.

Synthesis of ketimine and hemiaminal 49’

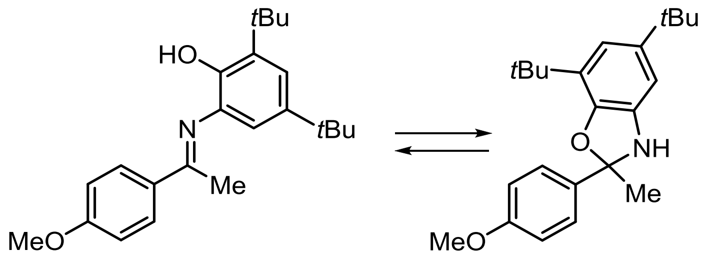

ketimine/hemiaminal ratio = 2.6:1

The title compound was prepared using **general method A**. Deep green viscous oil (Hemiaminal) and white solid (ketimine) (229 mg, 0.65 mmol, 98%). **FT-IR** (thin film) ν_{max} (cm^{-1}): 3396, 2958, 2905, 2870, 2300, 1675, 1599, 1250, 1118, 999, 832, 773. **Ketimine: ^{1}H NMR** (400 MHz, $CDCl_3$): δ 8.06 – 7.97 (m, 2 H), 7.13 (d, *J* = 2.3 Hz, 1 H), 6.99 – 6.94 (m, 2 H), 6.70 (d, *J* = 2.3 Hz, 1 H), 6.31 (s, 1 H, OH), 3.89 (s, 3 H), 2.47 (s, 3 H), 1.46 (s, 9 H), 1.32 (s, 9 H). **^{13}C NMR** (101 MHz, $CDCl_3$): δ 166.4, 161.9, 146.1, 140.9, 135.9, 134.6, 132.3, 129.0, 120.3, 115.3, 113.8, 55.5, 35.0, 34.5, 31.8, 29.7, 17.5. **Hemiaminal: ^{1}H NMR** (400 MHz, $CDCl_3$): δ 7.60 – 7.53 (m, 2 H), 6.92 – 6.87 (m, 2 H), 6.84 (d, *J* = 2.3 Hz, 1 H), 6.77 (d, *J* = 2.3 Hz, 1 H), 3.81 (s, 3 H), 1.86 (s, 3 H), 1.41 (s, 9 H), 1.28 (s, 9 H). **^{13}C NMR** (101 MHz, $CDCl_3$): 159.4, 146.7, 143.4, 137.0, 136.6, 131.2, 126.5, 116.8, 113.7, 109.2, 100.2, 55.4, 34.7, 34.3, 31.9, 29.7, 28.9. **HRMS** (ESI) Calcd for $C_{23}H_{32}NO_2$ [$(M+H)^+$]: 354.2428 found: 354.2433.

Synthesis of (*E*)-2-((3,5-di-*tert*-butyl-6-oxocyclohexa-2,4-dien-1-ylidene)amino)-2-(4-methoxyphenyl) propanenitrile (49'')

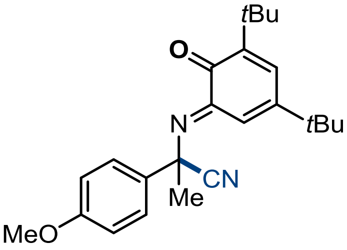

The title compound was prepared using **general method D**. The cleavage of phenol unit was performed by using 1.2 equiv of I_2 and 5 equiv NaOH (1 M) instead of H_5IO_6. The crude product was purified by column chromatography on silica gel using (pentane/ EtOAc = 80:20 v:v) and followed by EtOAc:MeOH:Et_3N (70:20:10 v:v) as solvent system. Brown oil (249 mg, 0.66 mmol, 99%). **FT-IR** (thin film) ν_{max} (cm^{-1}): 3310, 2963, 2865, 2361, 1677, 1605, 1511, 1253, 1176, 1031, 957, 909, 833, 731**. ^{1}H NMR** (400 MHz, $CDCl_3$): δ 7.41 (d, *J* = 8.6 Hz, 2 H), 6.92 – 6.84 (m, 3 H), 6.47 (d, *J* = 2.1 Hz, 1 H), 3.80 (s, 3 H), 2.17 (s, 3 H), 1.27 (s, 9 H), 1.06 (s, 9 H). **^{13}C NMR** (101 MHz, $CDCl_3$): δ 183.9, 159.8, 159.1, 154.6, 149.5, 134.2, 133.0, 126.2, 120.8, 114.9, 114.2, 59.5, 55.2, 35.6, 35.2, 34.0, 29.2, 28.0. **HRMS** (ESI) Calcd for $C_{24}H_{31}N_2O_2$ [$(M+H)^+$]: 379.2380, found: 379.2373.

Synthesis of 2-amino-2-(4-fluorophenyl)propanenitrile (50)

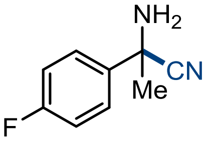

The title compound was prepared using **general method D**. The crude product was purified by column chromatography on neutralized silica gel (treated with Et_3N prior to column) using (pentane/

EtOAc = 80:20 v:v) and followed by EtOAc:MeOH:Et_3N (70:20:10 v:v) as solvent system. Yellow oil (57.0 mg, 0.35 mmol, 48%). **FT-IR** (thin film) ν_{max} (cm^{-1}): 3379, 2933, 2240, 1602, 1509, 1225, 1160, 838. **^{1}H NMR** (400 MHz, $CDCl_3$): δ 7.65 (dd, *J* = 8.9, 5.1 Hz, 2 H), 7.09 (t, *J* = 8.6 Hz, 2 H), 2.09 (br s, 2 H, NH_2), 1.75 (s, 3 H). **^{19}F NMR** (377 MHz, CD_3CN): δ -110.8. **^{13}C NMR** (126 MHz, $CDCl_3$): δ 162.8 (d, $J_{C\text{-}F}$ = 248.1 Hz), 137.2 (d, $J_{C\text{-}F}$ = 4.0 Hz), 127.0 (d, $J_{C\text{-}F}$ = 8.4 Hz), 124.1, 115.9 (d, $J_{C\text{-}F}$ = 21.7 Hz), 53.3, 32.1. **HRMS** (ESI) Calcd for C_8H_9FN [M-CN]: 138.0714, found: 138.0713.

Synthesis of 2-amino-2-propylpentanenitrile (51)

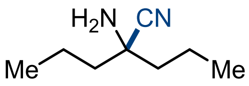

The title compound was prepared using **general method D**. Yellow oil (115 mg, 0.82 mmol, 94%). **FT-IR** (thin film) ν_{max} (cm^{-1}): 3376, 2962, 2935, 2876, 2221, 1465, 1270, 1164, 836. **^{1}H NMR** (400 MHz, $CDCl_3$): δ 1.75 (br s, 2 H, NH_2), 1.68 – 1.47 (m, 8 H), 0.99 (t, *J* = 7.1 Hz, 6 H). **^{13}C NMR** (101 MHz, $CDCl_3$): δ 124.1, 53.9, 42.5, 17.7, 14.2. **HRMS** (ESI) Calcd for $C_8H_{17}N_2$ [$(M+H)^+$]: 141.1386, found: 141.1385.

Synthesis of 2-amino-2-methylheptanenitrile (52)

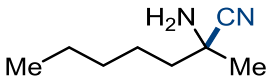

The title compound was prepared using **general method D**. Pale-yellow oil (111 mg, 0.79 mmol, 91%). **FT-IR** (thin film) ν_{max} (cm^{-1}): 3374, 2931, 2860, 2223, 1610, 1460, 1220, 1185, 863. **^{1}H NMR** (400 MHz, $CDCl_3$): δ 1.79 (br s, 2 H, NH_2), 1.65 – 1.56 (m, 2 H), 1.52 – 1.37 (m, 5 H), 1.36 – 1.23 (m, 4 H), 0.90 – 0.85 (m, 3 H). **^{13}C NMR** (101 MHz, $CDCl_3$): δ 124.6, 49.9, 41.8, 31.6, 27.7, 24.3, 22.5, 14.0. **HRMS** (ESI) Calcd for $C_8H_{17}N_2$ [$(M+H)^+$]: 141.1386, found: 141.1387.

Synthesis of 1-aminocyclooctane-1-carbonitrile (53)

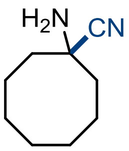

The title compound was prepared using **general method D**. Pale-yellow oil (212 mg, 1.39 mmol, 89%). **FT-IR** (thin film) ν_{max} (cm^{-1}): 3370, 3303, 2932, 2854, 2227, 1280, 1134, 883, 849. **^{1}H NMR** (400 MHz, CD_3CN): δ 1.91 – 1.77 (m, 4 H), 1.76 – 1.43 (m, 10 H). **^{13}C NMR** (101 MHz, CD_3CN): δ 126.5, 54.8, 36.4, 28.5, 25.0, 22.5. **HRMS** (ESI) Calcd for $C_9H_{17}N_2$ [$(M+H)^+$]: 153.1386, found: 153.1386.

Synthesis of 4-aminotetrahydro-2*H*-pyran-4-carbonitrile (54)[7]

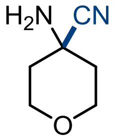

The title compound was prepared using **general method D**. Yellow oil (121 mg, 0.96 mmol, 97%). The spectroscopic data of compound **54** were identical to those reported. **^{1}H NMR** (400 MHz, CD_3CN): δ 4.03 – 3.93 (m, 2 H), 3.66 (ddd, *J* = 12.5, 10.3, 2.5 Hz, 2 H), 1.99 (ddt, *J* = 13.5, 4.3, 2.1 Hz, 2 H), 1.84 (br s, 2 H, NH_2), 1.75 (ddd, *J* = 13.9, 10.3, 4.2 Hz, 2 H). **^{13}C NMR** (101 MHz, CD_3CN): δ 122.4, 63.1, 48.1, 36.7. **HRMS** (ESI) Calcd for $C_6H_{11}N_2O$ [$(M+H)^+$]: 127.0866, found: 127.0866.

Synthesis of 4-aminotetrahydro-2*H*-thiopyran-4-carbonitrile 1,1-dioxide (55)

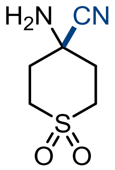

The title compound was prepared using **general method D**. White solid (49.0 mg, 0.28 mmol, 42%). **FT-IR** (thin film) ν_{max} (cm^{-1}): 3370, 3303, 2975, 2935, 2227, 1332, 1279, 1124, 882, 848. **^{1}H NMR** (400 MHz, CD_3CN): δ 3.31- 3.24 (m, 2 H), 2.99 – 2.92 (m, 2 H), 2.45 – 2.36 (m, 2 H), 2.34 – 2.22 (m, 2 H), 2.16 (br s, 2 H, NH_2). **^{13}C NMR** (126 MHz, CD_3CN): δ 123.9, 48.7, 47.2, 35.5. **HRMS** (ESI) Calcd for $C_5H_{10}NO_2S$ [M-CN]: 148.0427, found: 148.0427.

Synthesis of 4-amino-1-methylpiperidine-4-carbonitrile (56)[7]

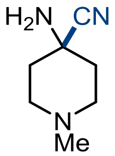

The title compound was prepared using **general method D**. 10 eq of coupling partner and carrying out the oxidation for 1 hour. Yellow oil (883 mg, 6.34 mmol, 72%). The spectroscopic data of compound **56** were identical to those reported. **^{1}H NMR** (400 MHz, $CDCl_3$): δ 4.58 (br s, 2 H), 2.81 (dt, *J* = 12.8, 4.2 Hz, 2 H), 2.38 – 2.26 (m, 5 H), 2.22 – 2.12 (m, 2 H), 1.83 (ddd, *J* = 14.1, 10.9, 3.9 Hz, 2 H). **^{13}C NMR** (101 MHz, $CDCl_3$): δ 119.8, 60.2, 51.5, 45.7, 34.2. **HRMS** (ESI) Calcd for $C_7H_{14}N_3$ [$(M+H)^+$]: 140.1182, found: 140.1182.

Synthesis of 2-((3,5-di-*tert*-butyl-2-hydroxyphenyl)amino)-2-phenylpropanenitrile (57)

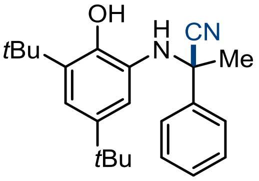

The title compound was prepared using **general method D** without oxidation. White crystals (1.40 g, 3.99 mmol, 97%). The recrystallization was performed using pentane: EtOAc (10:1) solvent system. mp = 141-148 ˚C. **FT-IR** (thin film) ν_{max} (cm^{-1}): 3390, 2959, 2906, 2870, 2230, 1598, 1485, 1227, 761, 699. -**^{1}H NMR** (400 MHz, CD_3CN): δ 7.68 – 7.60 (m, 2 H), 7.47 – 7.39 (m, 2 H), 7.39 – 7.31 (m, 1 H), 6.88 (d, *J* = 2.3 Hz, 1 H), 6.63 (d, *J* = 2.3 Hz, 1 H), 6.10 (s, 1 H, OH), 4.67 (s, 1 H, NH), 1.93 (s, 3 H), 1.36 (s, 9 H), 1.07 (s, 9 H). **^{13}C NMR** (101 MHz, CD_3CN): δ 145.0, 142.9, 141.3, 137.3, 133.2, 129.9, 129.4, 126.1, 122.5, 117.7, 116.3, 59.2, 35.5, 35.0, 31.7, 31.6, 30.1. **HRMS** (ESI) Calcd for $C_{23}H_{31}N_2O$ $[(M+H)^+]$: 351.2431, found: 351.2431.

Synthesis of *tert*-butyl 4-amino-4-(4-fluorophenyl)-2-methylenepentanoate (58)

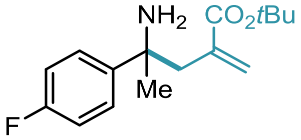

General Procedure E was carried out using 4-fluoro-α-methylbenzylamine (33.0 µL, 0.25 mmol). Silica gel column chromatography (EtOAc:Pentane 10:90 – 50:50 v:v) gave a colourless oil, (21.0 mg, 0.08, 30%). **FT-IR** (thin film): ν_{max} (cm^{-1}) = 2980, 1709, 1626, 1602, 15039. **^{1}H NMR** (400 MHz, CD_3CN): δ 7.53 – 7.43 (m, 2 H), 7.14 – 6.88 (m, 2 H), 5.97 (d, *J* = 1.9 Hz, 1 H), 5.33 – 5.23 (m, 1 H), 2.66 (dd, *J* = 1.9, 0.9 Hz, 2 H), 1.38 (s, 3 H), 1.37 (s, 9 H). **^{19}F NMR** (377 MHz, DMSO-d_6): δ -118.12 (ddd, *J* = 14.2, 9.1, 5.5 Hz). **^{13}C NMR** (101 MHz, CD_3CN): δ 167.8, 162.2 (d, *J* = 242.1 Hz), 146.0 (d, *J* = 3.1 Hz), 140.4, 128.6 (d, *J* = 7.9 Hz), 127.3, 115.1 (d, *J* = 21.3 Hz), 81.1, 55.9, 46.6, 31.0, 28.1. **HRMS** (ESI) Calcd for $C_{16}H_{22}O_2N_1F_1$ $[(M+H)^+]$: 280.1707, found: 280.1705.

Synthesis of *tert*-butyl 4-amino-4-(4-chlorophenyl)-2-methylenepentanoate (58')

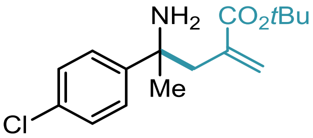

General Procedure E was carried out using 4-chloro-α-methylbenzylamine (36.0 µL, 0.25 mmol). Silica gel column chromatography (EtOAc:Pentane 10:90 – 50:50 v:v) gave a colourless oil, (16.2 mg,

0.05 mmol, 22%). **FT-IR** (thin film): ν_{max} (cm^{-1}) = 2976, 2931, 1707. **^{1}H NMR** (400 MHz, CD_3CN): δ 7.38 (d, *J* = 8.7 Hz, 2 H), 7.21 (d, *J* = 8.8 Hz, 2 H), 5.89 (d, *J* = 1.9 Hz, 1 H), 5.21 (dt, *J* = 1.9, 0.9 Hz, 1 H), 2.58 (d, *J* = 0.9 Hz, 2 H), 1.71 (s, 2 H), 1.30 (s, 3 H), 1.28 (s, 9 H). **^{13}C NMR** (101 MHz, CD_3CN) δ 167.8, 148.8, 140.3, 132.3, 128.6, 127.3, 81.1, 55.9, 46.5, 30.9, 28.1, 28.1. **HRMS** (ESI) Calcd for $C_{16}H_{23}O_2NCl$ [$(M+H)^+$]: 264.1412, found 296.1409.

Synthesis of *tert*-butyl 4-amino-4-phenyl-2-methylenepentanoate (59)

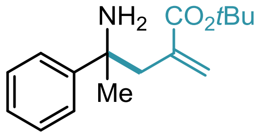

General Procedure E was carried out using α-methylbenzylamine (32.0 µL, 0.25 mmol). Silica gel column chromatography (EtOAc:Pentane 10:90 – 50:50 v:v) gave a colourless oil, (20.2 mg, 0.08 mmol, 31%). **FT-IR** (thin film): ν_{max} (cm^{-1}) = 2976, 2930, 1709, 1626. **^{1}H NMR** (400 MHz, CD_3CN): δ 7.55 – 7.42 (m, 2 H), 7.30 (dd, *J* = 8.4, 7.0 Hz, 2 H), 7.21 – 7.15 (m, 1 H), 5.97 (d, *J* = 2.0 Hz, 1 H), 5.28 (dt, *J* = 2.0, 1.0 Hz, 1 H), 2.79 – 2.50 (m, 2 H), 2.02 (s, 7 H), 1.39 (s, 9 H), 1.38 (s, 3 H). **^{13}C NMR** (101 MHz, CD_3CN): δ 167.9, 150.1, 140.5, 128.8, 127.1, 127.0, 126.6, 81.1, 56.1, 46.5, 30.8, 28.1. **HRMS** (ESI) Calcd for $C_{16}H_{24}O_2N_1$ [$(M+H)^+$]: 262.1802, found: 262.1800.

Synthesis of *tert*-butyl 4-amino-4-(4-methoxyphenyl)-2-methylenepentanoate (60)

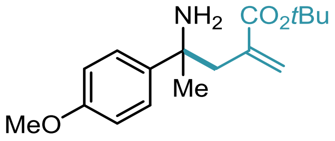

General Procedure E was carried out using 4-methoxy-α-methylbenzylamine (37.0 µL, 0.25 mmol). Silica gel column chromatography (EtOAc:Pentane 10:90 – 50:50 v:v) gave a colourless oil, (31.3 mg, 0.11 mmol, 43%). **FT-IR** (thin film): ν_{max} (cm^{-1}) = 2977, 1708, 1611, 1512, 1458. **^{1}H NMR** (400 MHz, CD_3CN): δ 7.48 (d, *J* = 8.8 Hz, 2 H), 6.94 (d, *J* = 8.8 Hz, 2 H), 6.07 (d, *J* = 2.1 Hz, 1 H), 5.40 – 5.35 (m, 1 H), 3.86 (s, 3 H), 2.75 (dd, *J* = 2.6, 0.9 Hz, 2 H), 1.48 (s, 9 H), 1.46 (s, 3 H). **^{13}C NMR** (101 MHz, CD_3CN): δ 167.9, 159.0, 142.1, 140.7, 127.7, 127.0, 114.0, 81.0, 55.8, 55.6, 46.7, 30.9, 28.1. **HRMS** (ESI) Calcd for $C_{17}H_{26}O_3N_1$ [$(M+H)^+$]: 292.1907, found 292.1910.

Synthesis of *tert*-butyl 4-amino-4-(4-methylphenyl)-2-methylenepentanoate (61)

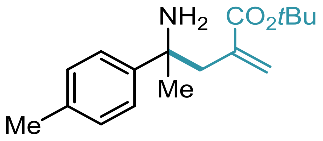

General Procedure E was carried out using 4-methyl-α-methylbenzylamine (37.0 µL, 0.25 mmol). Silica gel column chromatography (EtOAc:Pentane 10:90 – 50:50 v:v) gave a colourless oil, (22.0 mg, 0.08 mmol, 32%). **FT-IR** (thin film): ν_{max} (cm^{-1}) = 2980, 1710. **^{1}H NMR** (400 MHz, CD_3CN): δ 7.34 (d, *J* = 8.3 Hz, 2 H), 7.11 (d, *J* = 7.7 Hz, 2 H), 5.96 (d, *J* = 2.0 Hz, 1 H), 5.28 (dd, *J* = 2.0, 1.1 Hz, 1 H), 2.72 – 2.58 (m, 2 H), 2.28 (s, 3H), 1.38 (s, 9 H), 1.36 (s, 3 H). **^{13}C NMR** (101 MHz, CD_3CN): δ 167.9, 147.0, 140.6, 136.5, 129.4, 127.0, 126.5, 81.0, 55.9, 46.6, 30.7, 28.1, 20.9. **HRMS** (ESI) Calcd for $C_{17}H_{26}O_2N$ [(M+H)$^+$]: 276.1958, found 276.1958.

Synthesis of *tert*-butyl 2-((1-amino-2,3-dihydro-1H-inden-1-yl)methyl)acrylate (62)

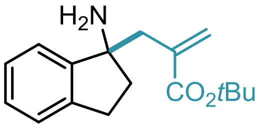

General Procedure E was carried out using 1-aminoindane (32.0 µL, 0.25 mmol). Silica gel column chromatography (EtOAc:Pentane 10:90 – 50:50 v:v) gave an amorphous solid, (25.3 mg, 0.09 mmol, 37%). **FT-IR** (thin film): ν_{max} (cm^{-1}) = 2980, 1707, 1626. **^{1}H NMR** (400 MHz, CD_3CN): δ 7.23 – 7.12 (m, 4 H), 6.03 (d, *J* = 2.0 Hz, 1 H), 5.41 (dd, *J* = 2.0, 1.0 Hz, 1 H), 2.82 (ddd, *J* = 9.1, 5.2, 4.0 Hz, 2 H), 2.72 (d, *J* = 13.1 Hz, 1 H), 2.48 (dd, *J* = 13.2, 1.0 Hz, 1 H), 2.21 (ddd, *J* = 12.7, 6.7, 3.9 Hz, 1 H), 1.95 (br s, 2 H), 1.79 (dt, *J* = 12.7, 8.8 Hz, 1 H), 1.38 (s, 9 H). **^{13}C NMR** (101 MHz, CD_3CN): δ 167.9, 151.0, 143.6, 140.6, 128.1, 127.3, 127.2, 125.5, 123.8, 81.1, 65.5, 42.4, 41.6, 30.0, 28.1. **HRMS** (ESI) Calcd for $C_{17}H_{24}O_2N$ [(M+H)$^+$]: 274.1802, found 274.1802.

Synthesis of 5-(4-methoxyphenyl)-5-methyl-3-methylenepyrrolidin-2-one (63)

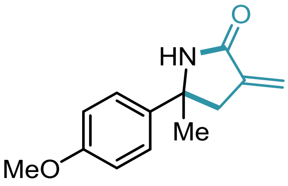

General Procedure E was followed using 4-methoxy-α-methylbenzylamine (37.0 µL, 0.25 mmol) and ethyl 2-((phenylsulfonyl)methyl)acrylate (254 mg, 1.00 mmol) as coupling partner. Work up with NaOH (1M) initiated an *in situ* lactamization, and purification *via* silica gel column chromatography (EtOAc:Pentane 10:90-40:60 v:v) gave an amorphous solid, (17.9 mg, 0.08 mmol, 33%). mp = 98-100 ˚C. **FT-IR** (thin film): ν_{max} (cm^{-1}) = 3212, 2968, 1695, 1658, 1613, 1583, 1514. **^{1}H NMR** (400 MHz, CD_3CN): δ 7.29 (d, *J* = 8.9 Hz, 2 H), 7.11 (s, 1 H), 6.90 (d, *J* = 8.8 Hz, 2 H), 5.81 (td, *J* = 2.7, 1.1 Hz, 1 H), 5.28 (tt, *J* = 2.4, 1.1 Hz, 1 H), 3.77 (s, 3 H), 2.93 (qt, *J* = 16.8, 2.5 Hz, 2 H), 1.59 (s, 3 H). **^{13}C NMR** (101

MHz, CD_3CN): δ 169.9, 159.6, 141.8, 140.3, 127.0, 115.8, 114.7, 59.0, 55.9, 45.0, 30.1 **HRMS** (ESI) Calcd for $C_{13}H_{16}O_2N$ [$(M+H)^+$]: 218.1176, found 218.1177.

Synthesis of 2-methyl-1-phenylpropan-2-amine (*Phentermine)* (64)[11]

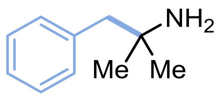

(*Phentermine*)

The title compound was prepared using **general method A**. 10 equiv of BnMgCl (2 M in THF) was used instead of allylmagnesium bromide for the addition. Pale yellow oil (134 mg, 0.90 mmol, 53%). The spectroscopic data of compound **64** were identical to those reported. **^{1}H NMR** (400 MHz, $CDCl_3$): δ 7.33 – 7.27 (m, 2 H), 7.27 – 7.21 (m, 1 H), 7.21 – 7.16 (m, 2 H), 2.66 (s, 2 H), 1.30 – 1.19 (br s, 2 H), 1.12 (s, 6 H). **^{13}C NMR** (101 MHz, $CDCl_3$): δ 138.6, 130.6, 128.1, 126.4, 51.3, 50.1, 30.6. **HRMS** (ESI) Calcd for $C_{10}H_{16}N$ [$(M+H)^+$]: 150.1277, found: 150.1277.

Synthesis of 2-(adamantan-1-yl)pent-4-en-2-amine hydrochloride *(from Rimantadine or Flumadine)* (65)

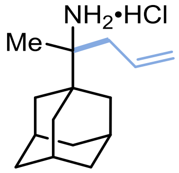

(from Rimantadine)

The title compound was prepared using **general method B**. Yellow solid (198 mg, 0.77 mmol, 54%). **FT-IR** (thin film) ν_{max} (cm^{-1}): 3032, 2904, 2851, 1641, 1516, 1449, 1197, 919, 731. **^{1}H NMR** (400 MHz, $CDCl_3$): δ 8.20 (br s, 3 H, NH_3^+), 6.21 – 6.08 (m, 1 H), 5.21 – 5.12 (m, 2 H), 2.63 (dd, *J* = 14.5, 5.9 Hz, 1 H), 2.34 (dd, *J* = 14.5, 8.3 Hz, 1 H), 2.03 (s, 3 H), 1.86 – 1.70 (m, 6 H), 1.64 (s, 6 H), 1.26 (s, 3 H). **^{13}C NMR** (101 MHz, $CDCl_3$): δ 132.9, 120.0, 62.5, 39.0, 38.2, 36.6, 36.1, 28.4, 18.5. **HRMS** (ESI) Calcd for $C_{15}H_{26}N$ [$(M-Cl)^+$]: 220.2060, found: 220.2059.

Synthesis of 2-(adamantan-1-yl)-2-aminopropanenitrile*(from Rimantadine or Flumadine)* (66)

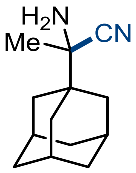

(from Rimantadine)

The title compound was prepared using **general method D**. White powder (100 mg, 0.49 mmol, 88%). **FT-IR** (thin film) $ν_{max}$ (cm^{-1}): 3376, 2904, 2850, 2218, 1630, 1450, 1266, 1102, 851, 815. **^{1}H NMR** (400 MHz, CD_3CN): δ 2.03 (m, 3 H), 1.76 – 1.62 (m, 12 H), 1.32 (s, 3 H). **^{13}C NMR** (101 MHz, CD_3CN): δ 125.3, 58.4, 38.6, 37.4, 36.8, 29.3, 21.9. **HRMS** (ESI) Calcd for $C_{13}H_{21}N_2$ [$(M+H)^+$]: 205.1699, found: 205.1700.

Synthesis of 1-(2,6-dimethylphenoxy)-2-methylpent-4-en-2-amine (*Mexiletine*) (67)

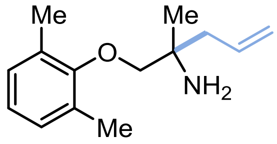

(from Mexiletine)

The title compound was prepared using **general method B**. The HCl salt of compound **67** was basified with 1 M NaOH (1 mL), extracted with $CHCl_3$ (3 x 20 mL) and dried by Na_2SO_4. After removal of solvents gave the desired 1-(2,6-dimethylphenoxy)-2-methylpent-4-en-2-amine **67** as a pale-yellow oil (150 mg, 0.68 mmol, 57%). **FT-IR** (thin film) $ν_{max}$ (cm^{-1}): 3380, 2968, 2922, 2840, 1639, 1476, 1263, 1202, 1020, 767. **^{1}H NMR** (400 MHz, $CDCl_3$): δ 7.01 (d, *J* = 7.3 Hz, 2 H), 6.92 (dd, *J* = 8.3, 6.6 Hz, 1 H), 5.98 – 5.88 (m, 1 H), 5.19 – 5.13 (m, 2 H), 3.54 (dd, *J* = 10.6, 8.6 Hz, 2 H), 2.38 – 2.34 (m, 2 H), 2.29 (s, 6 H), 1.68 (br s, 2 H, NH_2), 1.25 (s, 3 H). **^{13}C NMR** (101 MHz, $CDCl_3$): δ 155.2, 133.9, 131.0, 129.1, 124.0, 118.8, 79.6, 52.8, 44.5, 25.1, 16.5. **HRMS** (ESI) Calcd for $C_{14}H_{22}NO$ [$(M+H)^+$]: 220.1696, found: 220.1697.

Synthesis of 2-amino-2-methyl-3-phenylpropanenitrile (68)

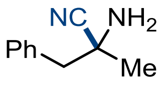

(from Amphetamine)

The title compound was prepared using **general method D**. Pale yellow oil (87.0 mg, 0.54 mmol, 94%). **FT-IR** (thin film) $ν_{max}$ (cm^{-1}): 3374, 3031, 2925, 2361, 1603, 1454, 846, 760, 702. **^{1}H NMR** (400 MHz, CD_3CN): δ 7.44 – 7.26 (m, 5 H), 2.90 (q, *J* = 13.3 Hz, 2 H), 1.97 (br s, 2 H, NH_2), 1.43 (s, 3 H). **^{13}C NMR** (101 MHz, CD_3CN): δ 136.6, 131.5, 129.2, 128.3, 125.3, 51.7, 48.0, 27.8. **HRMS** (ESI) Calcd for $C_{10}H_{13}N_2$ [$(M+H)^+$]: 161.1073, found: 161.1073.

6: NMR Spectra:

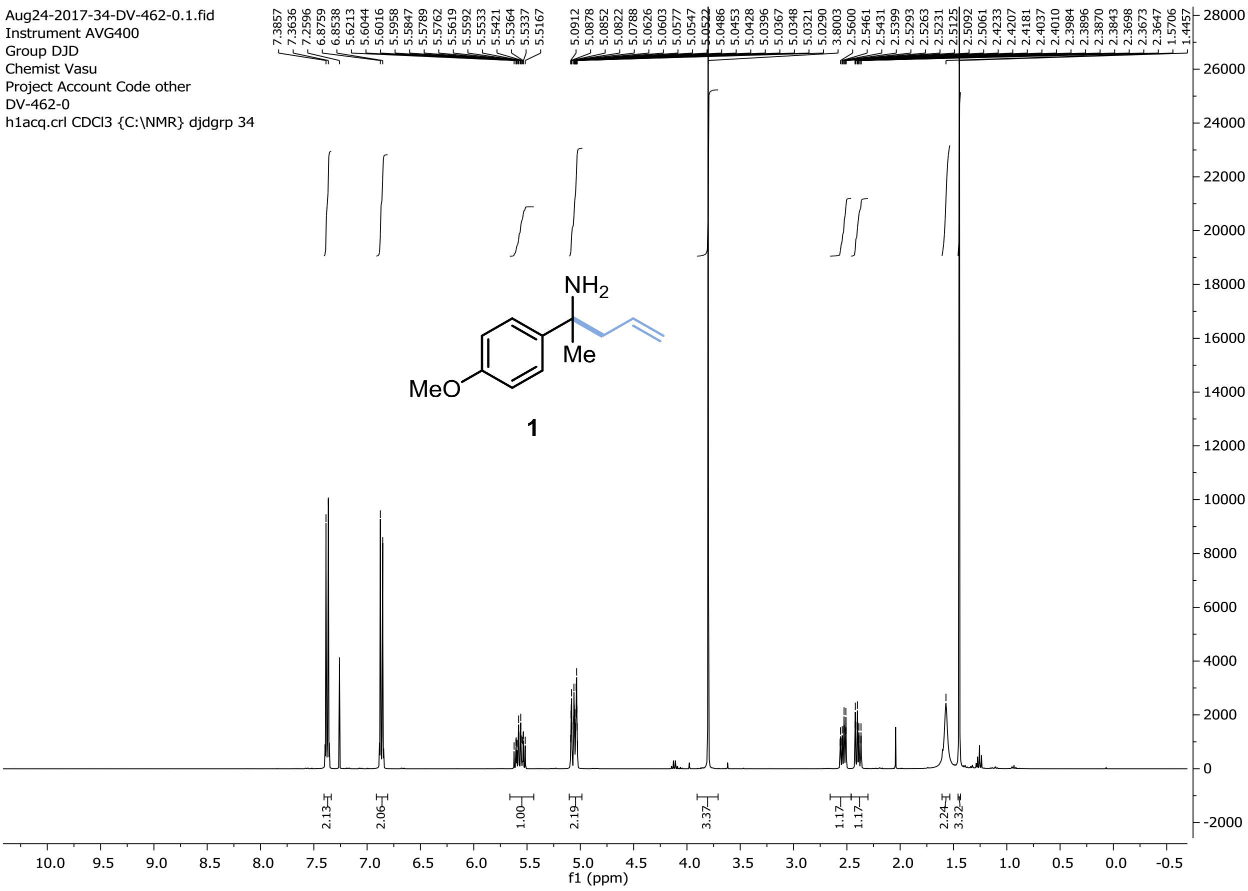
Aug24-2017-34-DV-462-0.1.fid
Instrument AVG400
Group DJD
Chemist Vasu
Project Account Code other
DV-462-0
h1acq.crl CDCl3 {C:\NMR} djdgrp 34
7.3857 7.3636 7.2596 6.8759 6.8538 5.6213 5.6044 5.6016 5.5958 5.5847 5.5789 5.5762 5.5619 5.5592 5.5533 5.5421 5.5364 5.5337 5.5167
5.0912 5.0878 5.0852 5.0822 5.0788 5.0626 5.0603 5.0577 5.0547 5.0522 5.0486 5.0453 5.0428 5.0396 5.0367 5.0348 5.0321 5.0290 3.8003
2.5600 2.5461 2.5431 2.5399 2.5293 2.5263 2.5231 2.5125 2.5092 2.5061 2.4233 2.4207 2.4181 2.4037 2.4010 2.3984 2.3896 2.3870 2.3843 2.3698 2.3673 2.3647 1.5706 1.4457
NH2
Me
MeO
1
2.13 2.06 1.00 2.19 3.37 1.17 1.17 2.24 3.32
f1 (ppm)

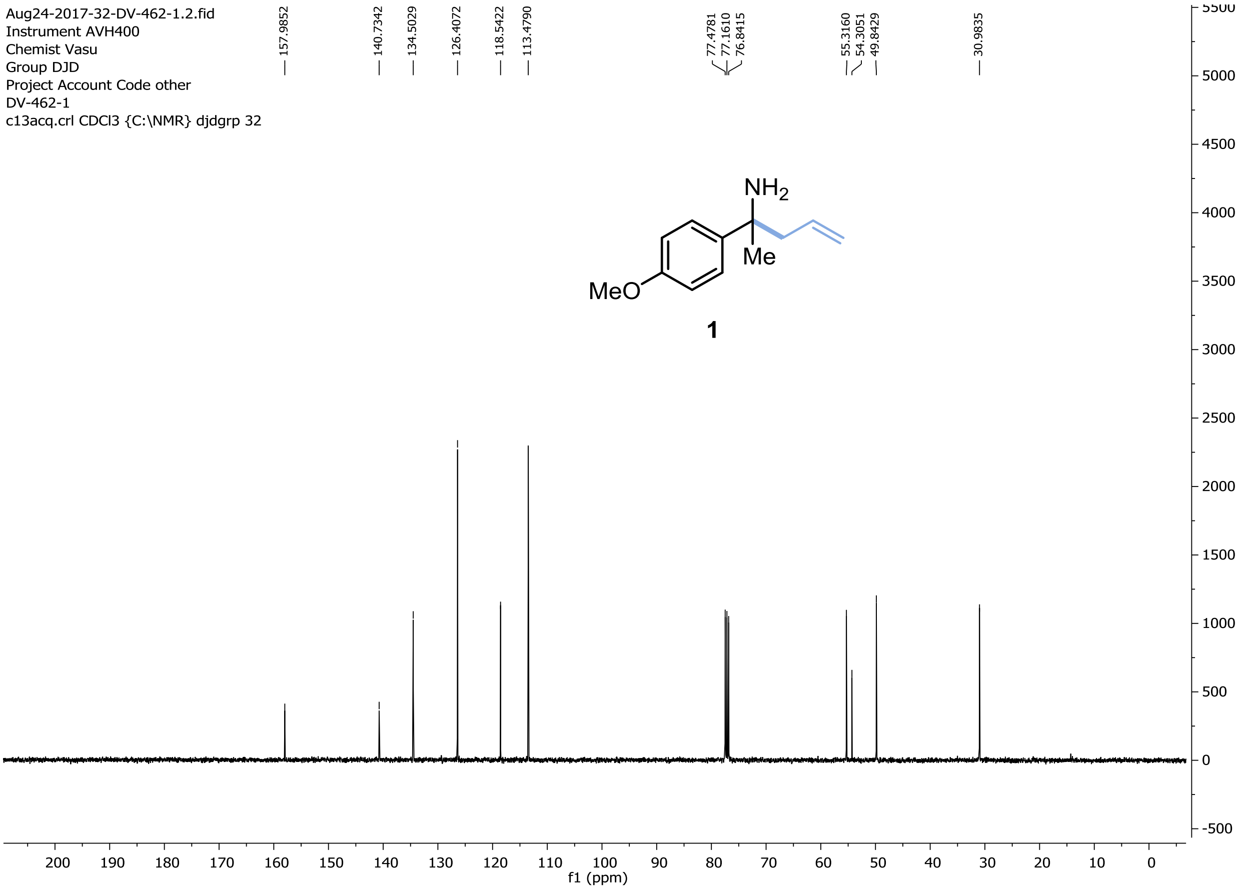
Aug24-2017-32-DV-462-1.2.fid
Instrument AVH400
Chemist Vasu
Group DJD
Project Account Code other
DV-462-1
c13acq.crl CDCl3 {C:\NMR} djdgrp 32
157.9852 140.7342 134.5029 126.4072 118.5422 113.4790
77.4781 77.1610 76.8415
55.3160 54.3051 49.8429
30.9835
NH2
Me
MeO
1
f1 (ppm)

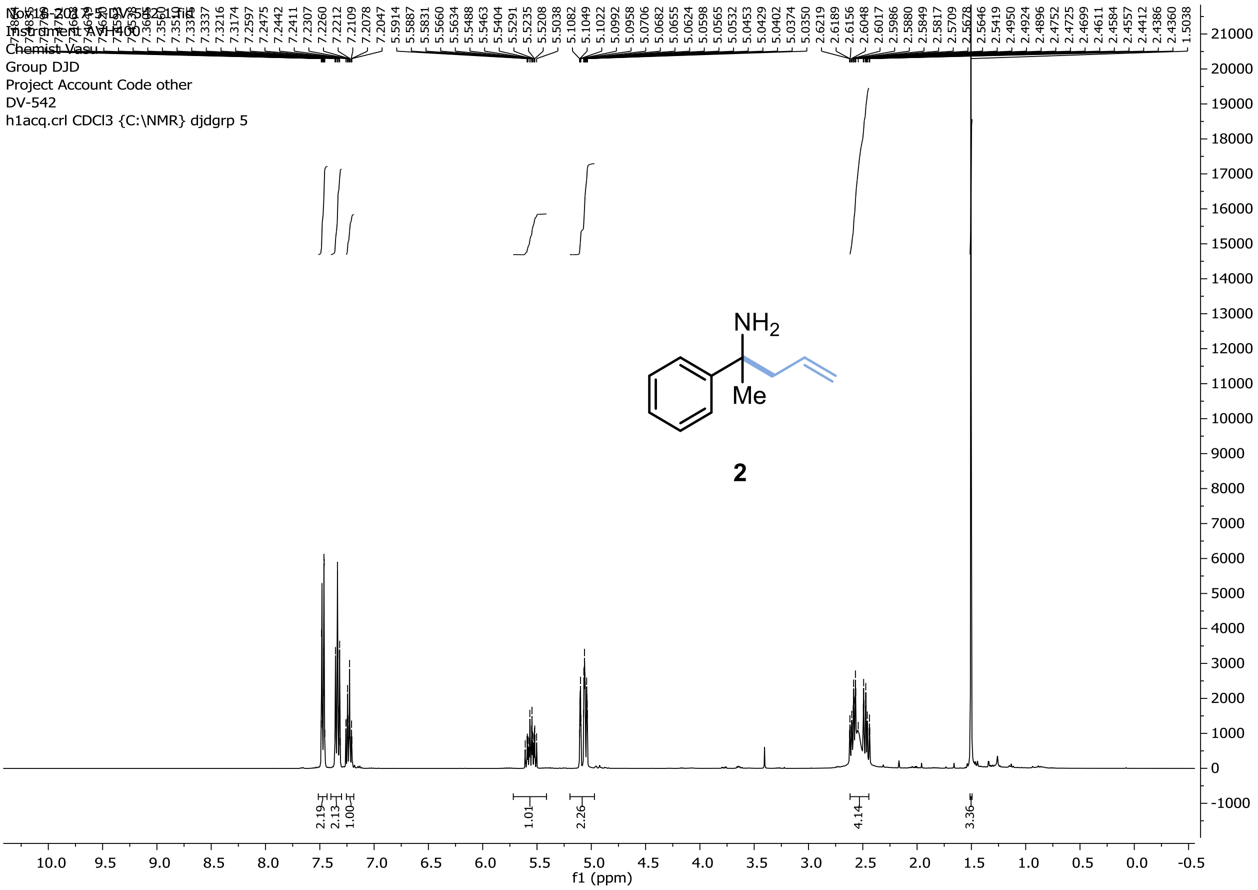

Instrument AVH400
Chemist Vasu
Group DJD
Project Account Code other
DV-542
h1acq.crl CDCl3 {C:\NMR} djdgrp 5
7.3562 7.3516 7.3381 7.3337 7.3216 7.3174 7.2597 7.2475 7.2442 7.2411 7.2307 7.2260 7.2212 7.2109 7.2078 7.2047
5.5914 5.5887 5.5831 5.5660 5.5634 5.5488 5.5463 5.5404 5.5291 5.5235 5.5208 5.5038
5.1082 5.1049 5.1022 5.0992 5.0958 5.0706 5.0682 5.0655 5.0624 5.0598 5.0565 5.0532 5.0453 5.0429 5.0402 5.0374 5.0350
2.6219 2.6189 2.6156 2.6048 2.6017 2.5986 2.5880 2.5849 2.5817 2.5709 2.5678 2.5646 2.5419 2.4950 2.4924 2.4896 2.4752 2.4725 2.4699 2.4611 2.4584 2.4557 2.4412 2.4386 2.4360 1.5038
NH2
Me
2
2.19 2.13 1.00 1.01 2.26 4.14 3.36
f1 (ppm)

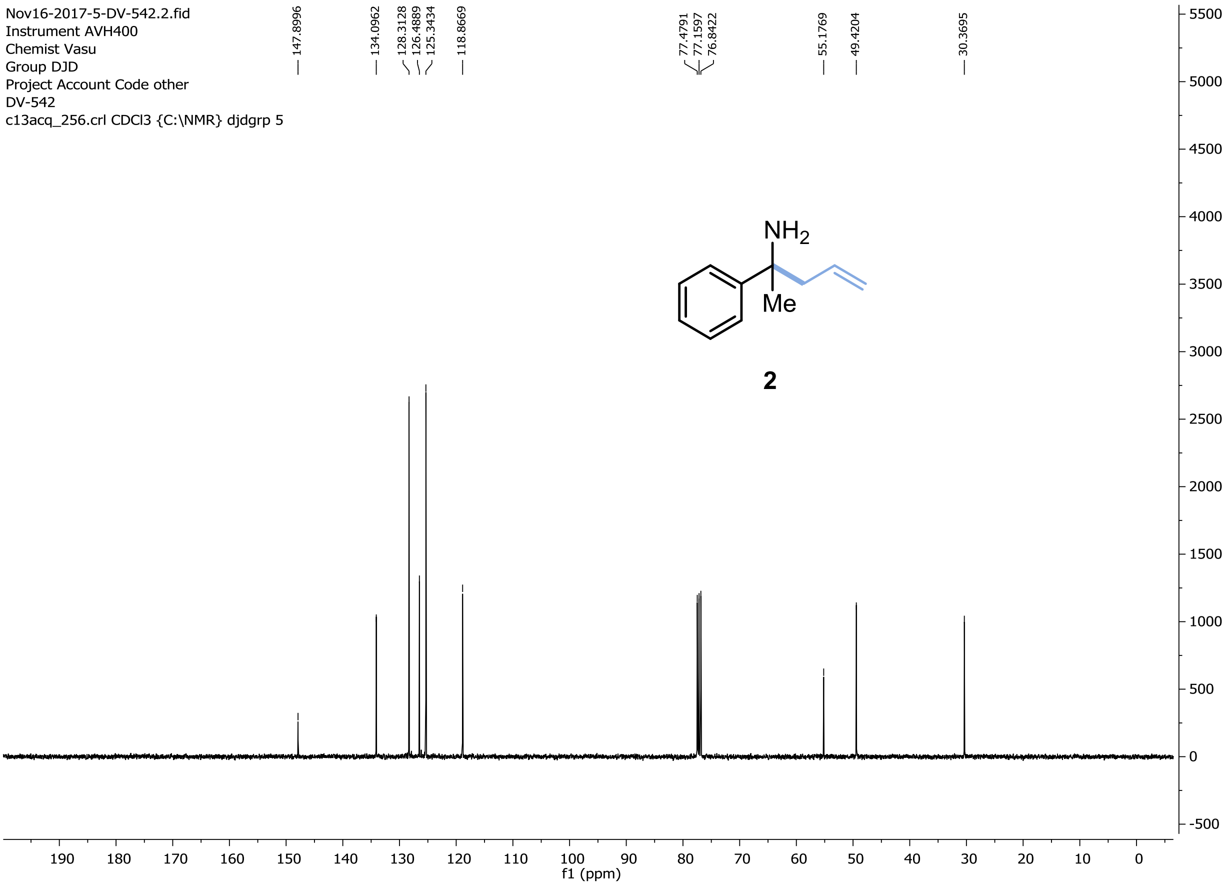

Nov16-2017-5-DV-542.2.fid
Instrument AVH400
Chemist Vasu
Group DJD
Project Account Code other
DV-542
c13acq_256.crl CDCl3 {C:\NMR} djdgrp 5
147.8996
134.0962 128.3128 126.4889 125.3434
118.8669
77.4791 77.1597 76.8422
55.1769
49.4204
30.3695
NH2
Me
2
f1 (ppm)

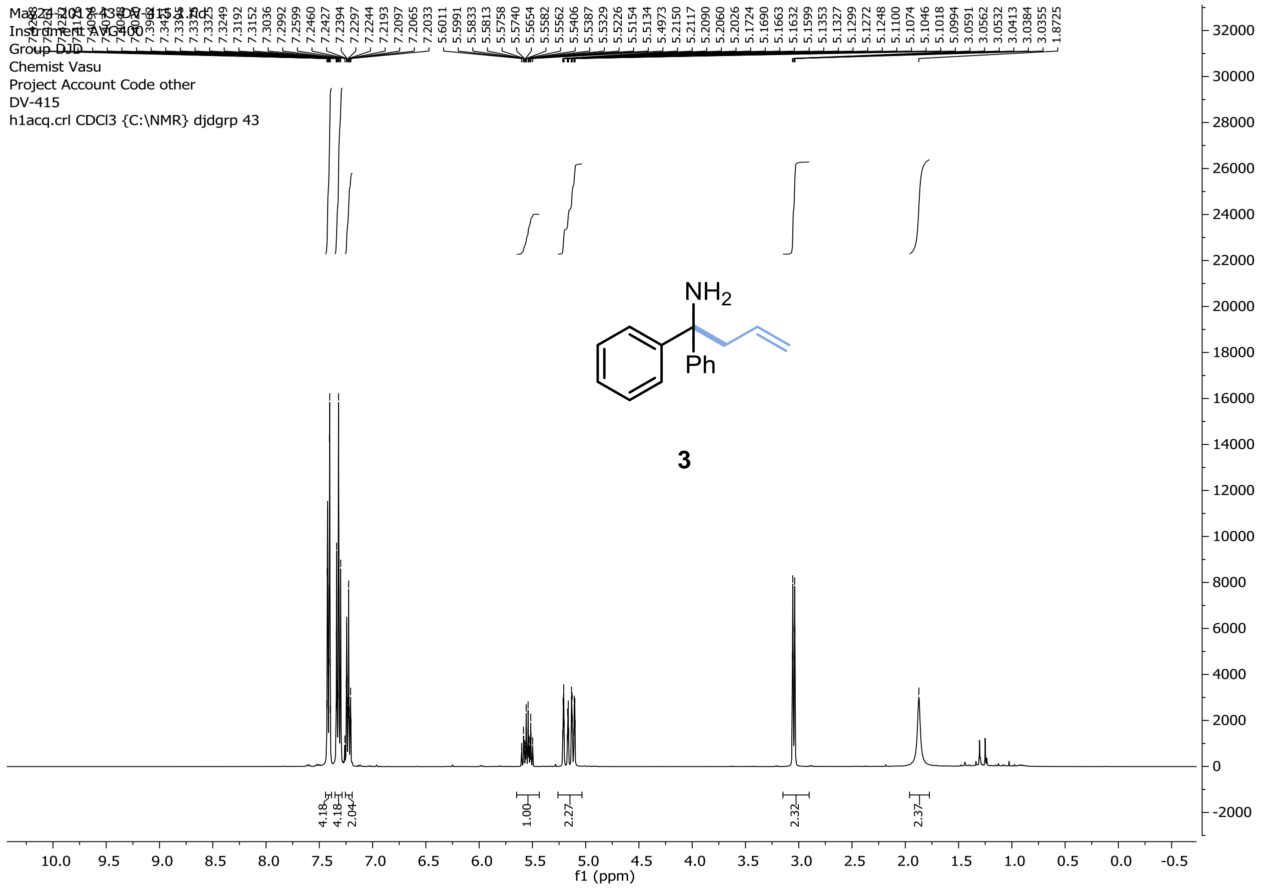
Instrument AVG400
Group DJD
Chemist Vasu
Project Account Code other
DV-415
h1acq.crl CDCl3 {C:\NMR} djdgrp 43
NH2
Ph
3
f1 (ppm)

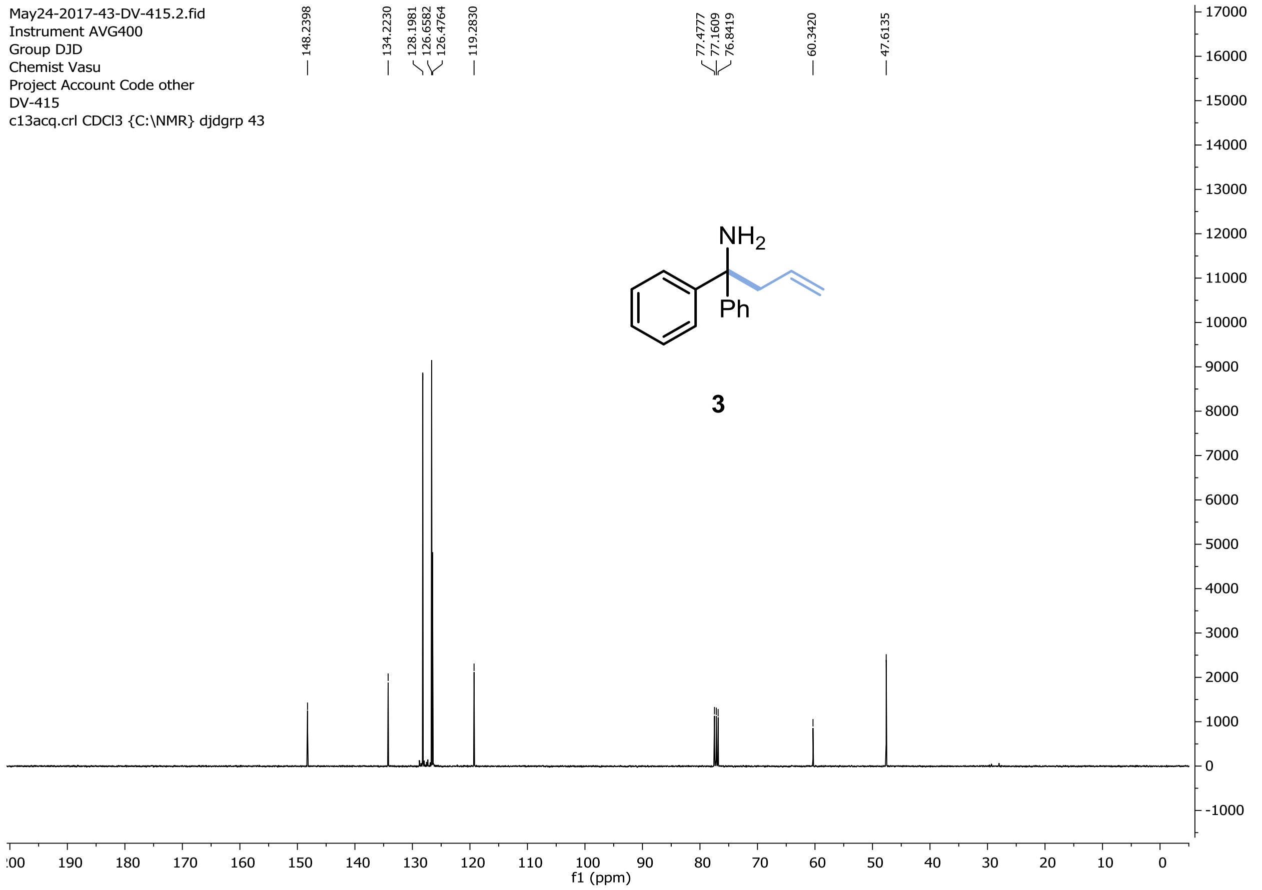
May24-2017-43-DV-415.2.fid
Instrument AVG400
Group DJD
Chemist Vasu
Project Account Code other
DV-415
c13acq.crl CDCl3 {C:\NMR} djdgrp 43
148.2398
134.2230
128.1981
126.6582
126.4764
119.2830
77.4777
77.1609
76.8419
60.3420
47.6135
NH2
Ph
3
f1 (ppm)

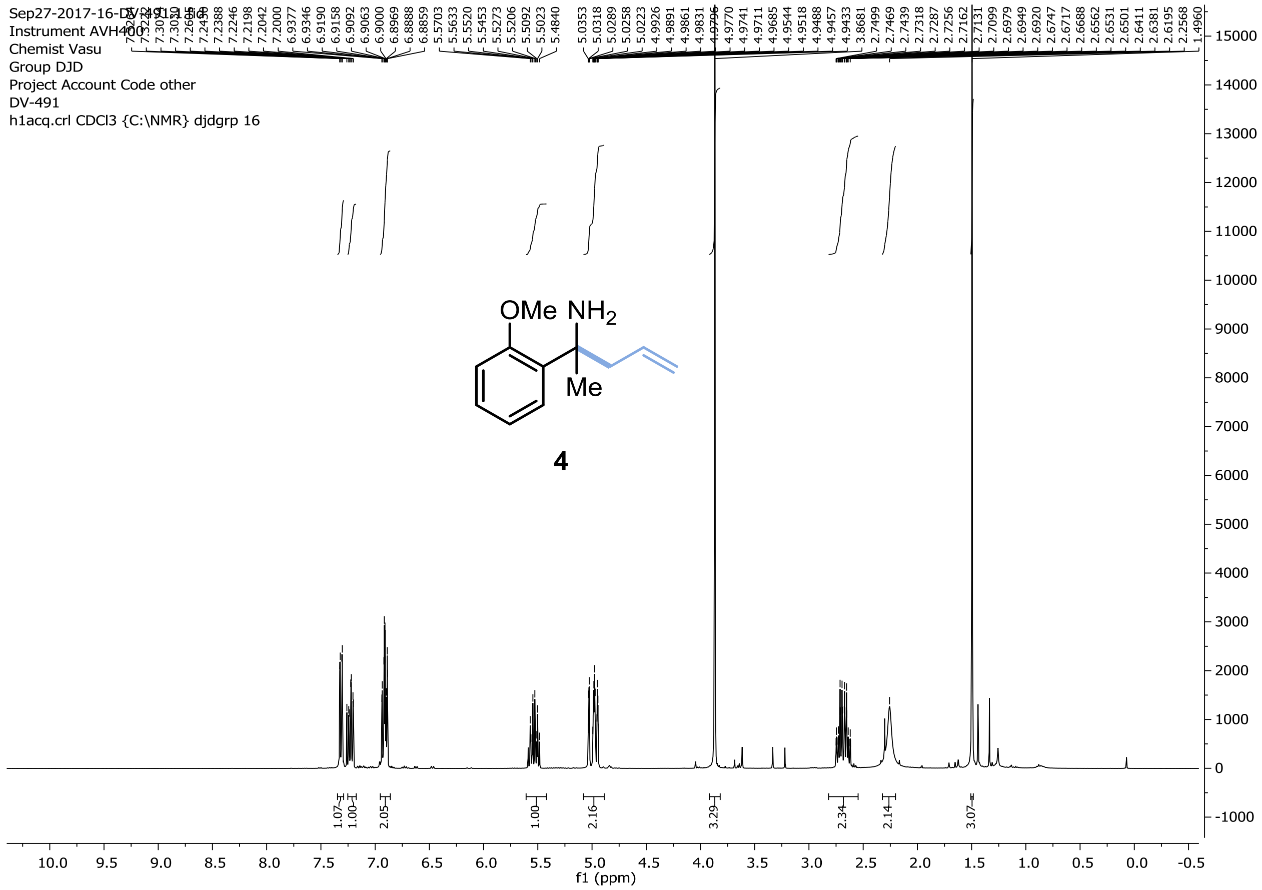
Sep27-2017-16-DV-491.1.fid
Instrument AVH400
Chemist Vasu
Group DJD
Project Account Code other
DV-491
h1acq.crl CDCl3 {C:\NMR} djdgrp 16
7.2388
7.2246
7.2198
7.2042
7.2000
6.9377
6.9346
6.9190
6.9158
6.9092
6.9063
6.9000
6.8969
6.8888
6.8859
5.5703
5.5633
5.5520
5.5453
5.5273
5.5206
5.5092
5.5023
5.4840
5.0353
5.0318
5.0289
5.0258
5.0223
4.9926
4.9891
4.9861
4.9831
4.9770
4.9741
4.9711
4.9685
4.9544
4.9518
4.9488
4.9457
4.9433
3.8681
2.7499
2.7469
2.7439
2.7318
2.7287
2.7256
2.7162
2.7131
2.7099
2.6979
2.6949
2.6920
2.6747
2.6717
2.6688
2.6562
2.6531
2.6501
2.6411
2.6381
2.6195
2.2568
1.4960
OMe NH2
Me
4
1.07
1.00
2.05
1.00
2.16
3.29
2.34
2.14
3.07
f1 (ppm)

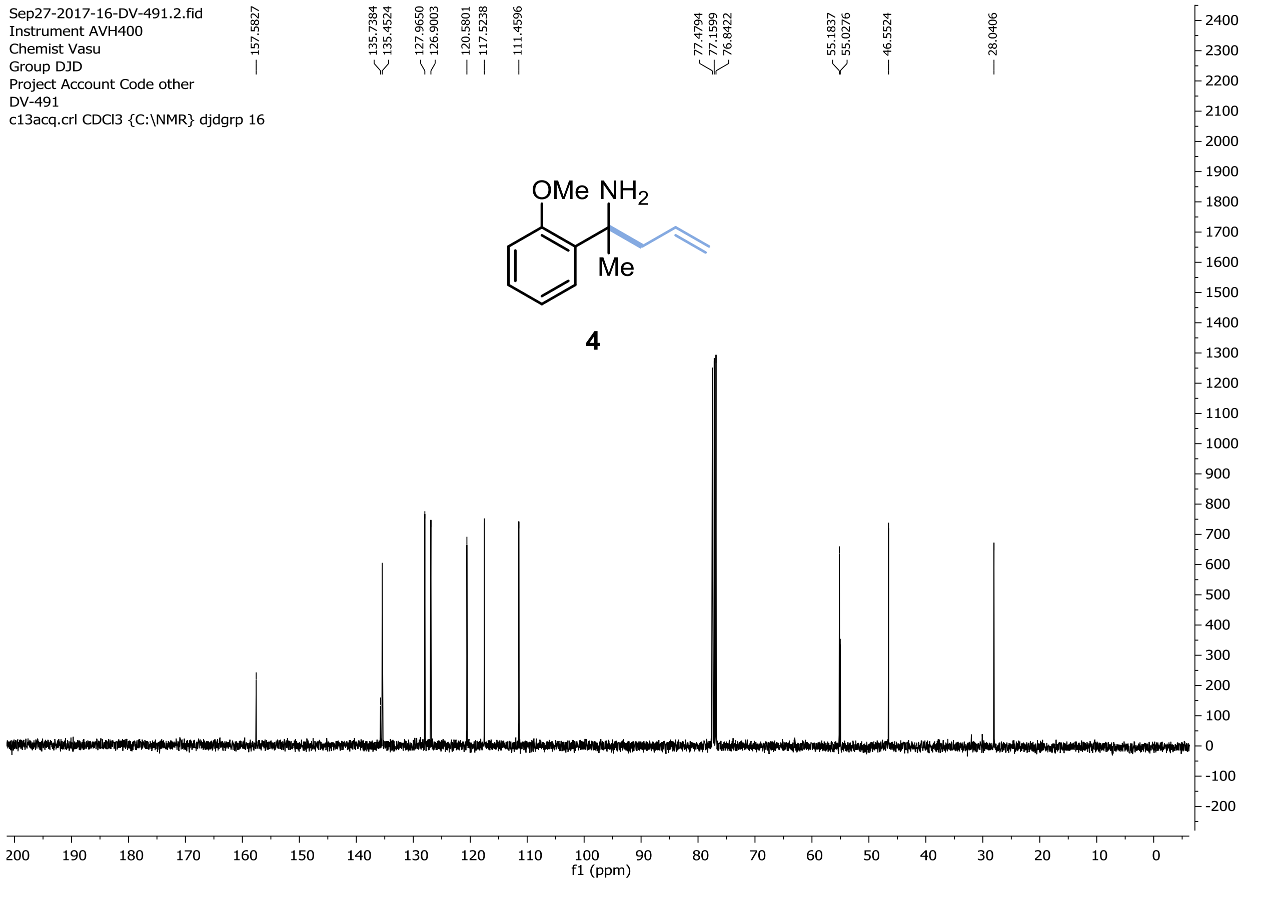
Sep27-2017-16-DV-491.2.fid
Instrument AVH400
Chemist Vasu
Group DJD
Project Account Code other
DV-491
c13acq.crl CDCl3 {C:\NMR} djdgrp 16
157.5827
135.7384
135.4524
127.9650
126.9003
120.5801
117.5238
111.4596
77.4794
77.1599
76.8422
55.1837
55.0276
46.5524
28.0406
OMe NH2
Me
4
f1 (ppm)

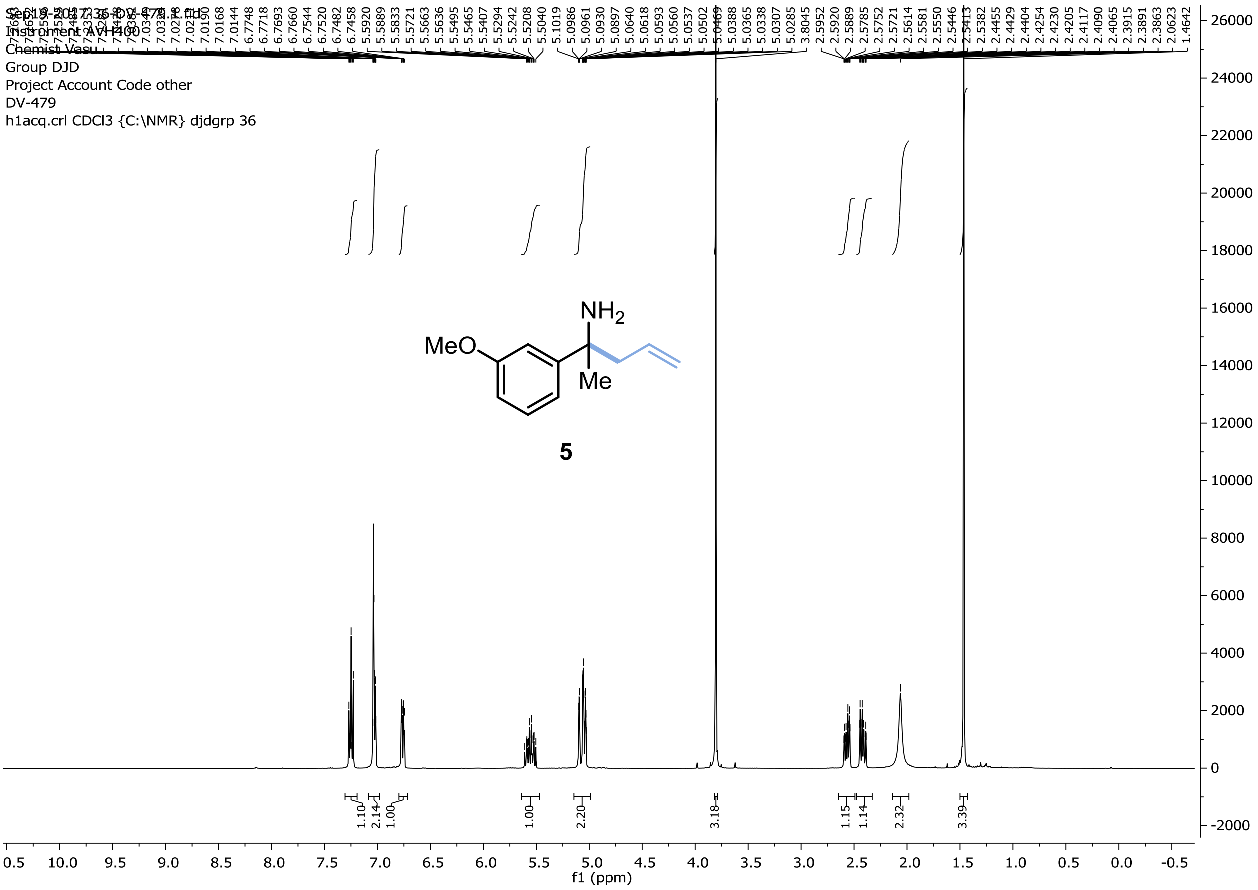
Sep19-2017-36-DV-479.1.fid
Instrument AVH400
Chemist Vasu
Group DJD
Project Account Code other
DV-479
h1acq.crl CDCl3 {C:\NMR} djdgrp 36
7.0190 7.0168 7.0144 6.7748 6.7718 6.7693 6.7660 6.7544 6.7520 6.7482 6.7458 5.5920 5.5889 5.5833 5.5721 5.5663 5.5636 5.5495 5.5465 5.5407 5.5294 5.5242 5.5208 5.5040 5.1019 5.0986 5.0961 5.0930 5.0897 5.0640 5.0618 5.0593 5.0560 5.0537 5.0502 5.0469 5.0388 5.0365 5.0338 5.0307 5.0285 3.8045 2.5952 2.5920 2.5889 2.5785 2.5752 2.5721 2.5614 2.5581 2.5550 2.5446 2.5413 2.5382 2.4455 2.4429 2.4404 2.4254 2.4230 2.4205 2.4117 2.4090 2.4065 2.3915 2.3891 2.3863 2.0623 1.4642
MeO
NH2
Me
5
1.10 2.14 1.00 1.00 2.20 3.18 1.15 1.14 2.32 3.39
f1 (ppm)

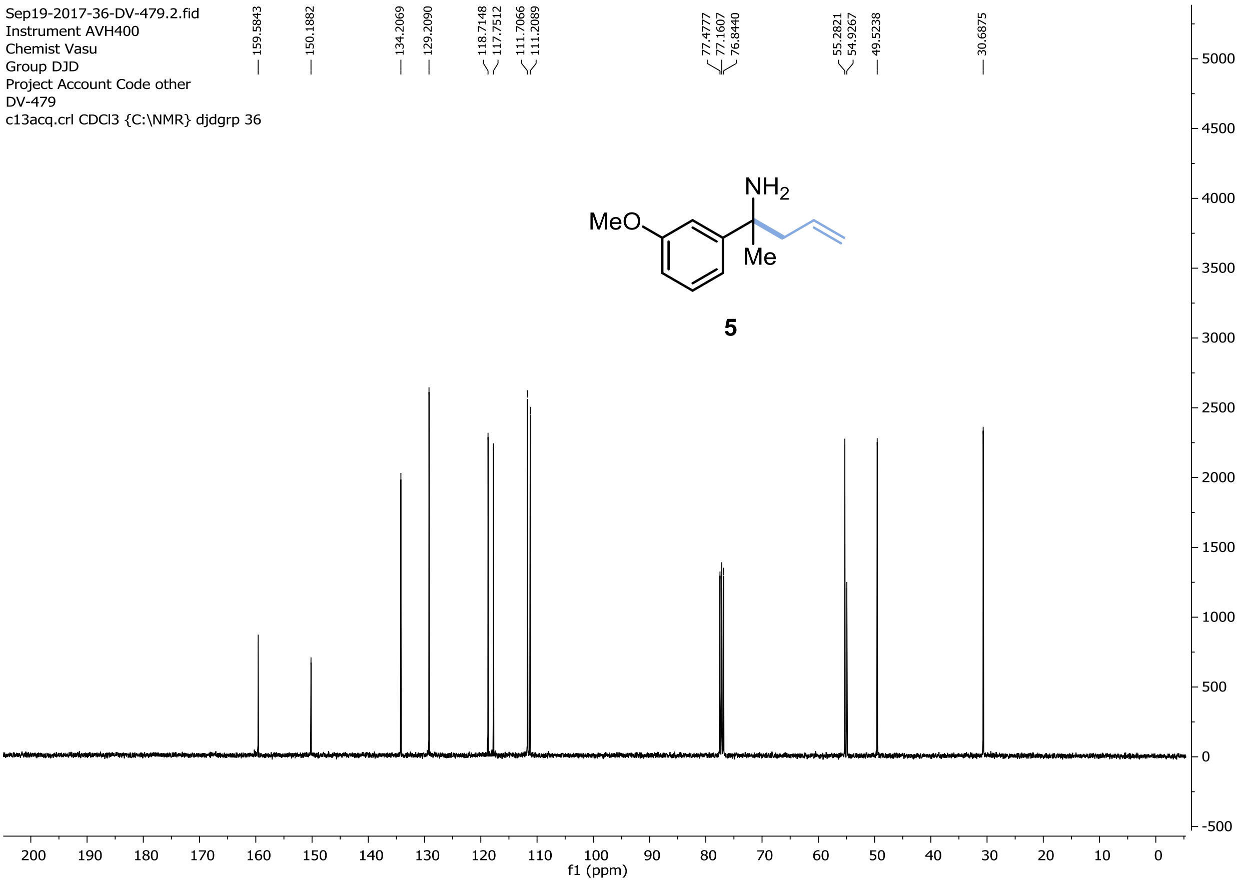
Sep19-2017-36-DV-479.2.fid
Instrument AVH400
Chemist Vasu
Group DJD
Project Account Code other
DV-479
c13acq.crl CDCl3 {C:\NMR} djdgrp 36
159.5843 150.1882 134.2069 129.2090 118.7148 117.7512 111.7066 111.2089 77.4777 77.1607 76.8440 55.2821 54.9267 49.5238 30.6875
MeO
NH2
Me
5
f1 (ppm)

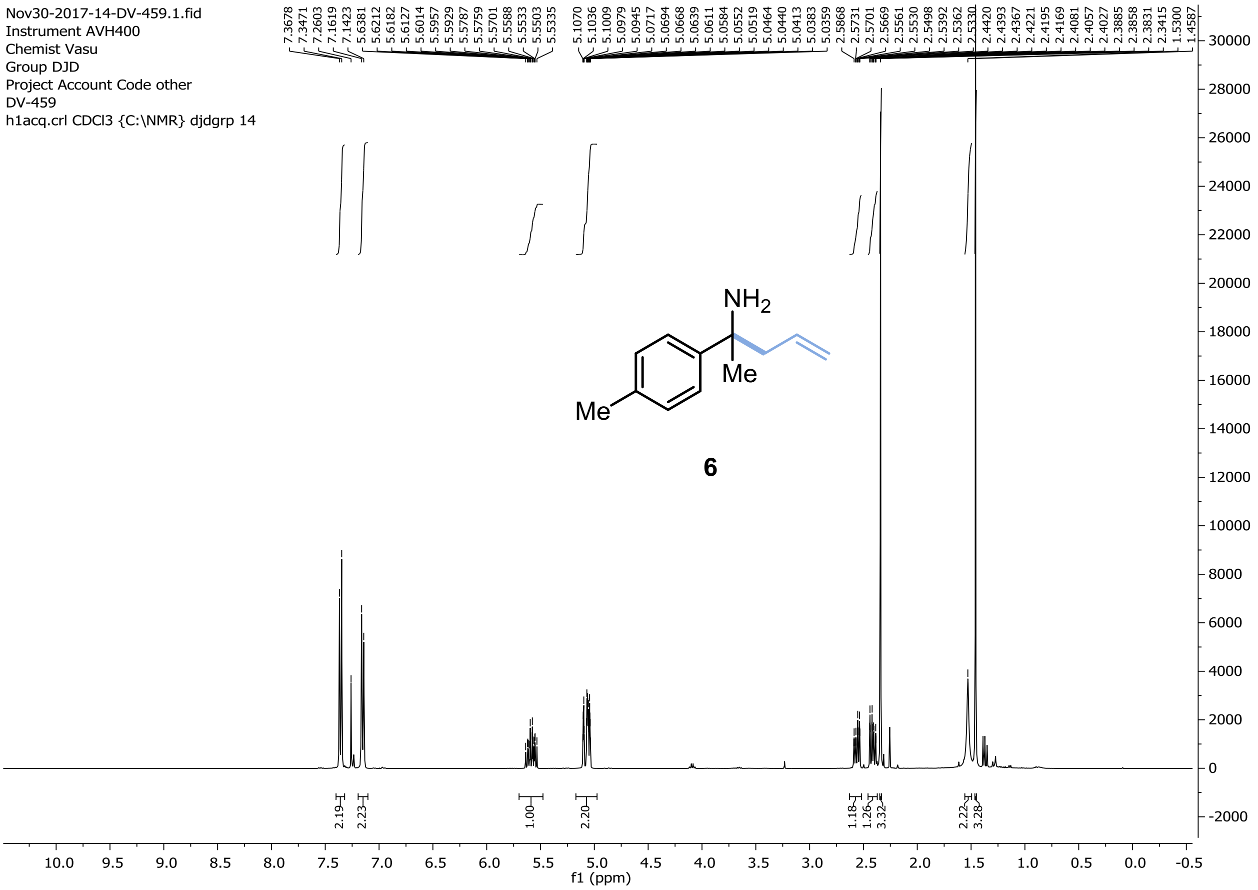
Nov30-2017-14-DV-459.1.fid
Instrument AVH400
Chemist Vasu
Group DJD
Project Account Code other
DV-459
h1acq.crl CDCl3 {C:\NMR} djdgrp 14
7.3678 7.3471 7.2603 7.1619 7.1423 5.6381 5.6212 5.6182 5.6127 5.6014 5.5957 5.5929 5.5787 5.5759 5.5701 5.5588 5.5533 5.5503 5.5335 5.1070 5.1036 5.1009 5.0979 5.0945 5.0717 5.0694 5.0668 5.0639 5.0611 5.0584 5.0552 5.0519 5.0464 5.0440 5.0413 5.0383 5.0359 2.5868 2.5731 2.5701 2.5669 2.5561 2.5530 2.5498 2.5392 2.5362 2.5330 2.4420 2.4393 2.4367 2.4221 2.4195 2.4169 2.4081 2.4057 2.4027 2.3885 2.3858 2.3831 2.3415 1.5300 1.4587
NH2
Me
Me
6
2.19 2.23 1.00 2.20 1.18 1.26 3.32 2.22 3.28
f1 (ppm)

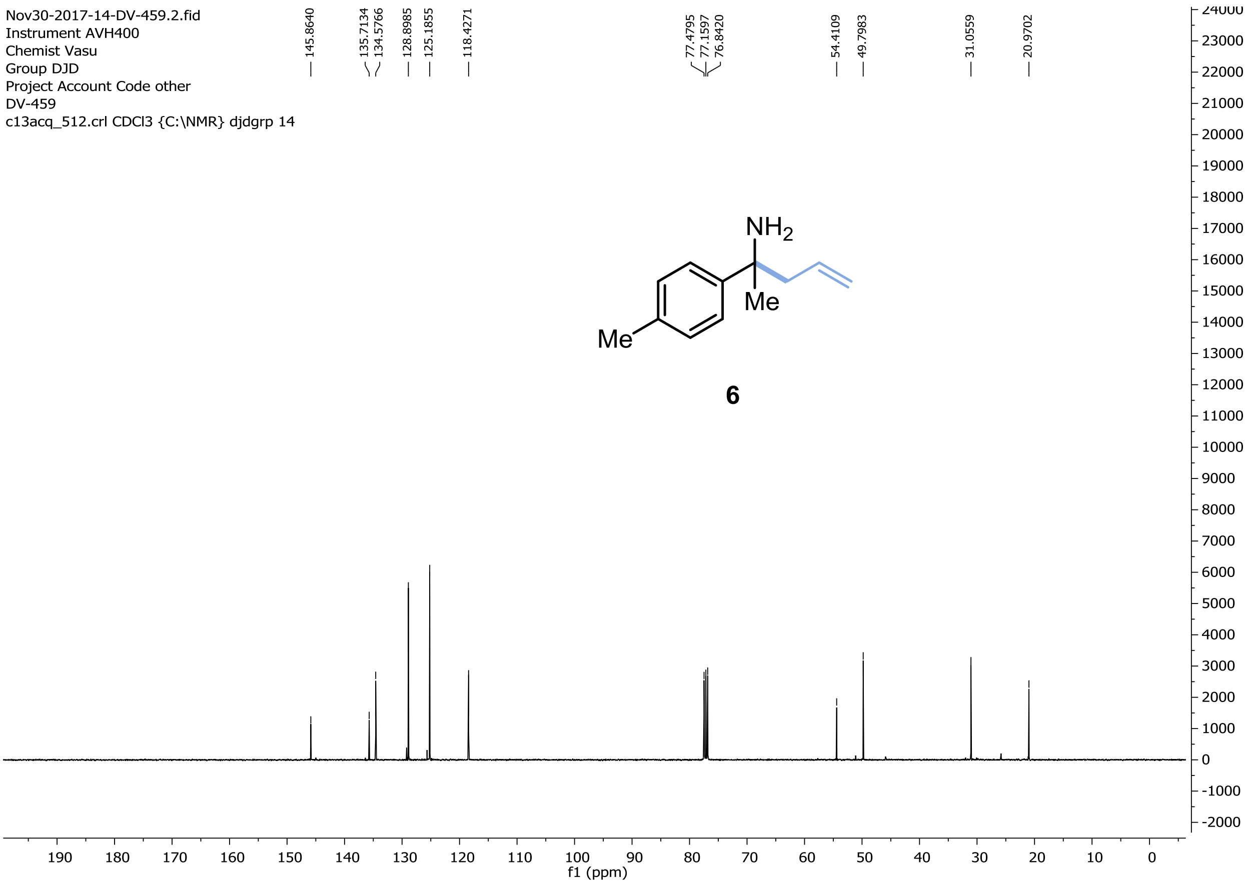
Nov30-2017-14-DV-459.2.fid
Instrument AVH400
Chemist Vasu
Group DJD
Project Account Code other
DV-459
c13acq_512.crl CDCl3 {C:\NMR} djdgrp 14
145.8640 135.7134 134.5766 128.8985 125.1855 118.4271 77.4795 77.1597 76.8420 54.4109 49.7983 31.0559 20.9702
NH2
Me
Me
6
f1 (ppm)

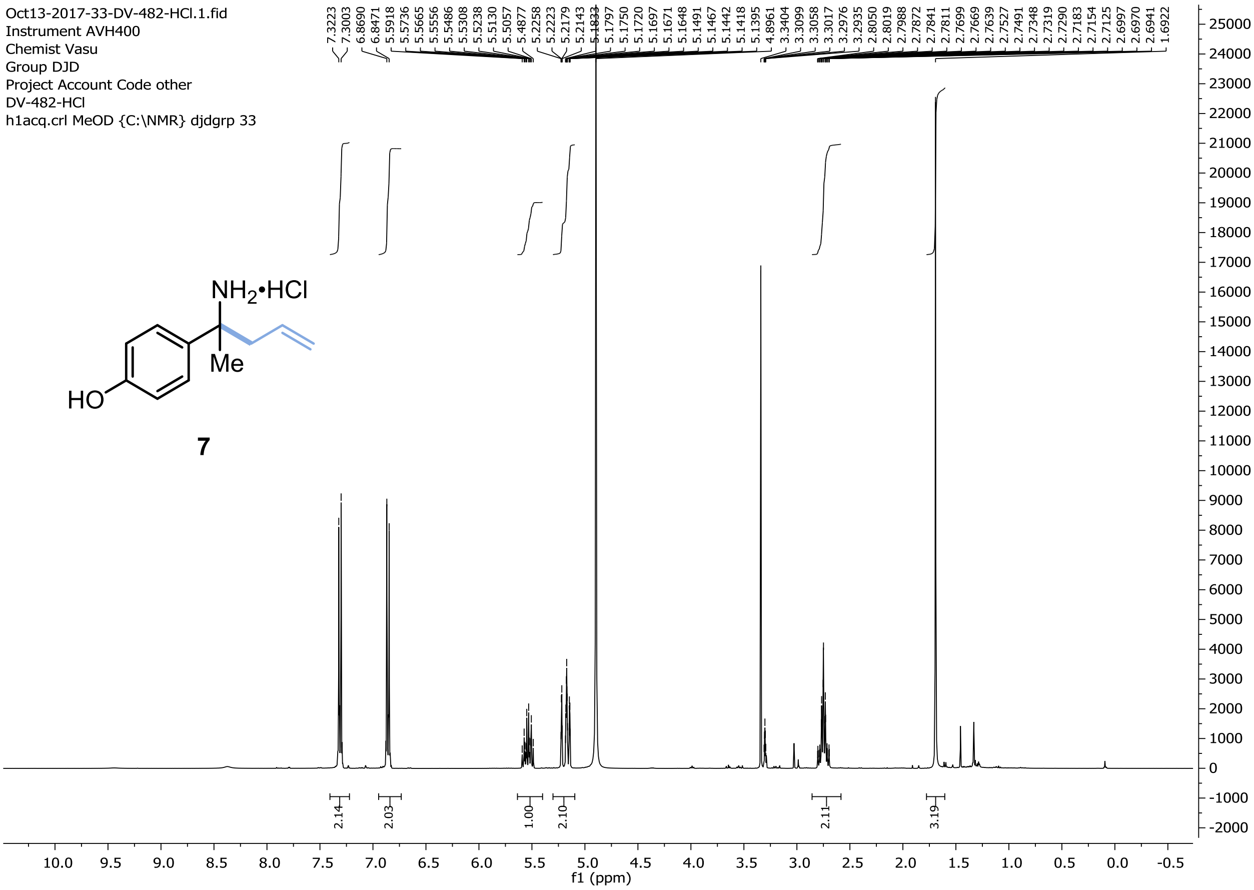
Oct13-2017-33-DV-482-HCl.1.fid
Instrument AVH400
Chemist Vasu
Group DJD
Project Account Code other
DV-482-HCl
h1acq.crl MeOD {C:\NMR} djdgrp 33
7.3223
7.3003
6.8690
6.8471
5.5918
5.5736
5.5665
5.5556
5.5486
5.5308
5.5238
5.5130
5.5057
5.4877
5.2258
5.2223
5.2179
5.2143
5.1833
5.1797
5.1750
5.1720
5.1697
5.1671
5.1648
5.1491
5.1467
5.1442
5.1418
5.1395
4.8961
3.3404
3.3099
3.3058
3.3017
3.2976
3.2935
2.8050
2.8019
2.7988
2.7872
2.7841
2.7811
2.7699
2.7669
2.7639
2.7527
2.7491
2.7348
2.7319
2.7290
2.7183
2.7154
2.7125
2.6997
2.6970
2.6941
1.6922
NH2•HCl
Me
HO
7
2.14
2.03
1.00
2.10
2.11
3.19
f1 (ppm)

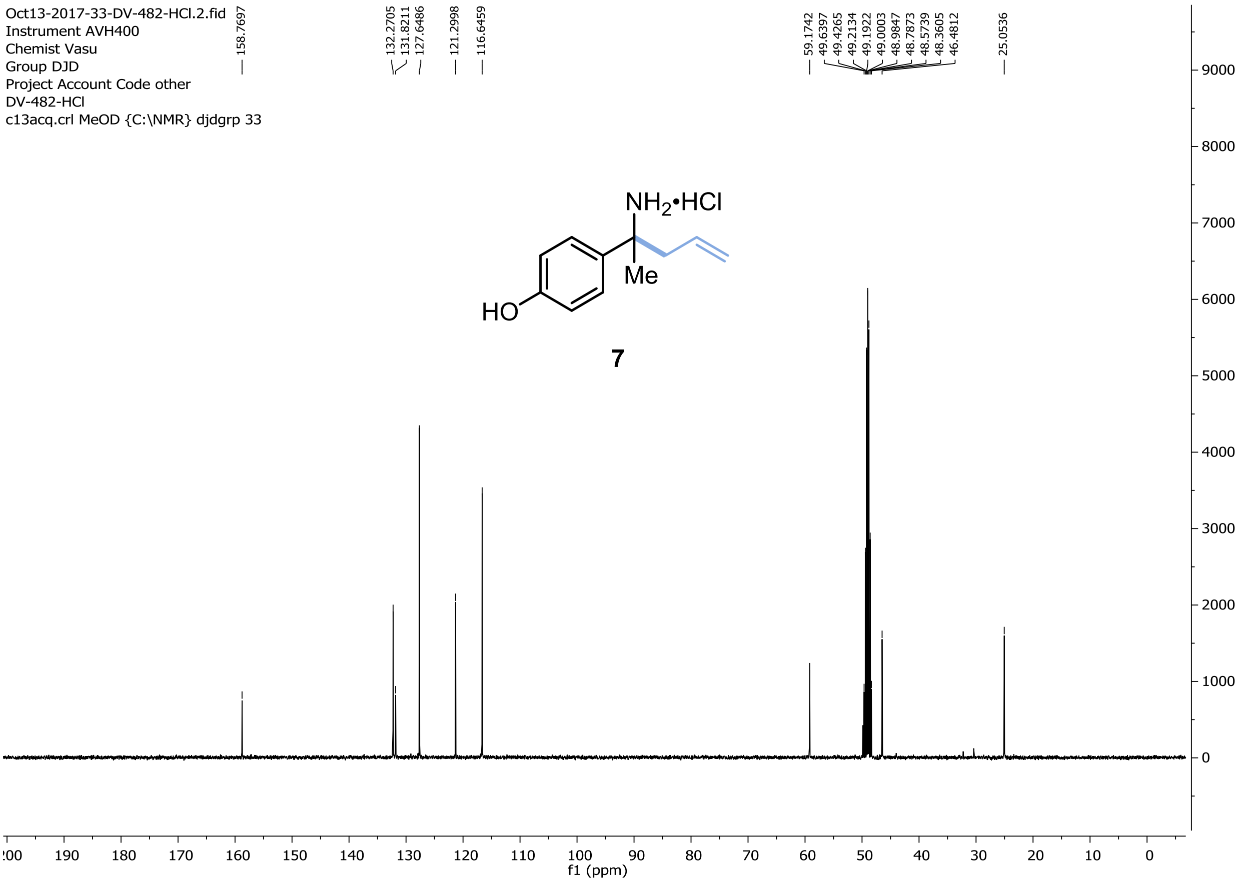
Oct13-2017-33-DV-482-HCl.2.fid
Instrument AVH400
Chemist Vasu
Group DJD
Project Account Code other
DV-482-HCl
c13acq.crl MeOD {C:\NMR} djdgrp 33
158.7697
132.2705
131.8211
127.6486
121.2998
116.6459
59.1742
49.6397
49.4265
49.2134
49.1922
49.0003
48.9847
48.7873
48.5739
48.3605
46.4812
25.0536
NH2•HCl
Me
HO
7
f1 (ppm)

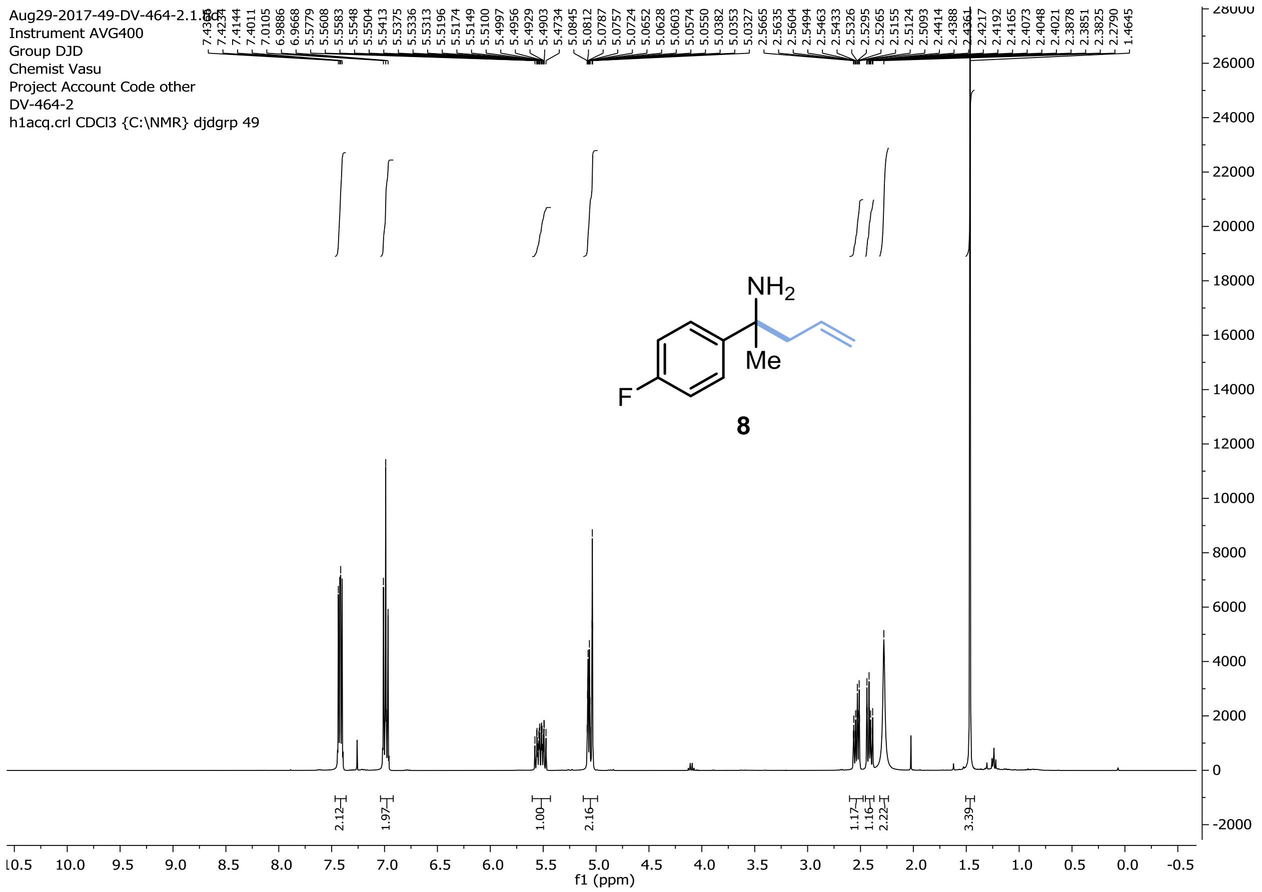
Aug29-2017-49-DV-464-2.1.fid
Instrument AVG400
Group DJD
Chemist Vasu
Project Account Code other
DV-464-2
h1acq.crl CDCl3 {C:\NMR} djdgrp 49
NH2
Me
F
8
f1 (ppm)

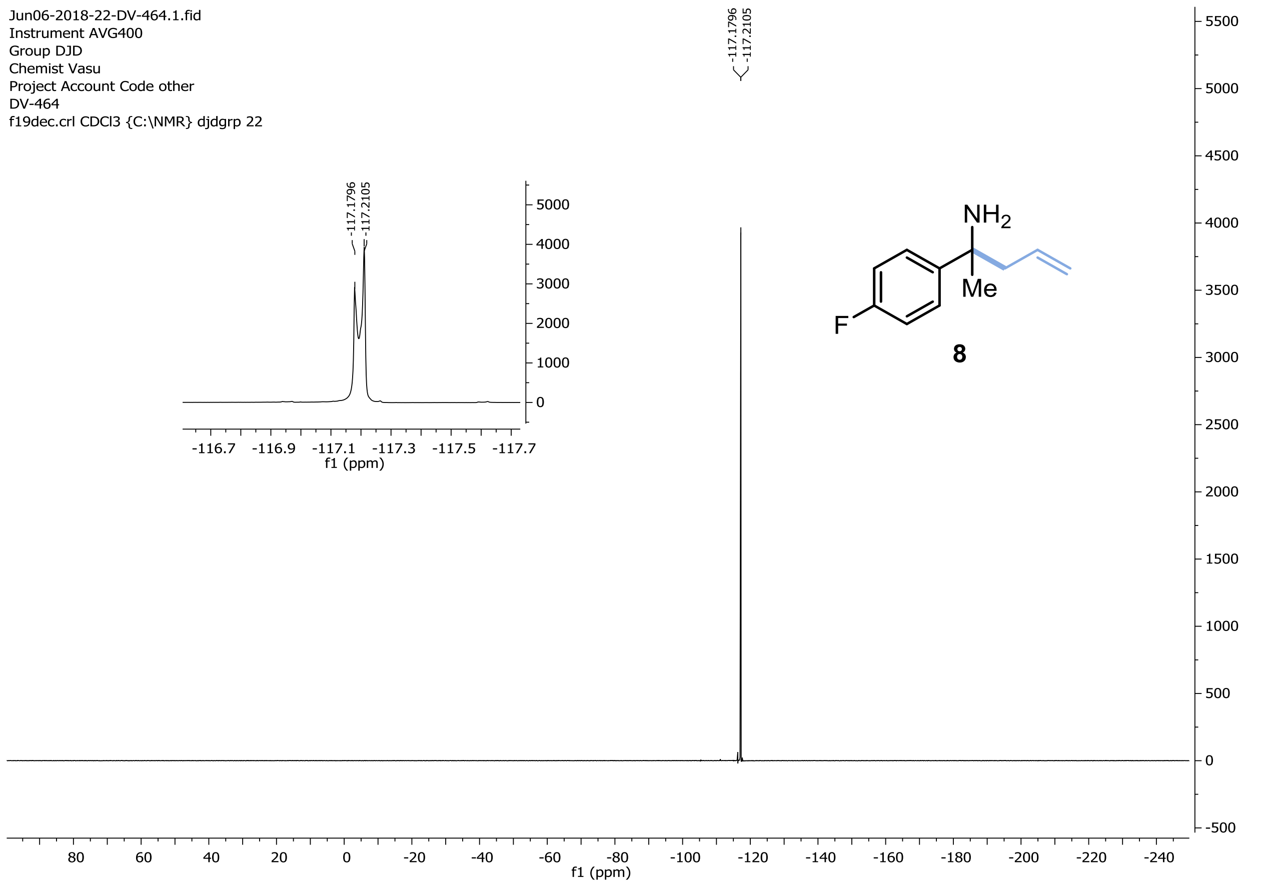
Jun06-2018-22-DV-464.1.fid
Instrument AVG400
Group DJD
Chemist Vasu
Project Account Code other
DV-464
f19dec.crl CDCl3 {C:\NMR} djdgrp 22
-117.1796
-117.2105
NH2
Me
F
8
f1 (ppm)

Aug29-2017-49-DV-464-2.2.fid
Instrument AVG400
Group DJD
Chemist Vasu
Project Account Code other
DV-464-2
c13acq.crl CDCl3 {C:\NMR} djdgrp 49

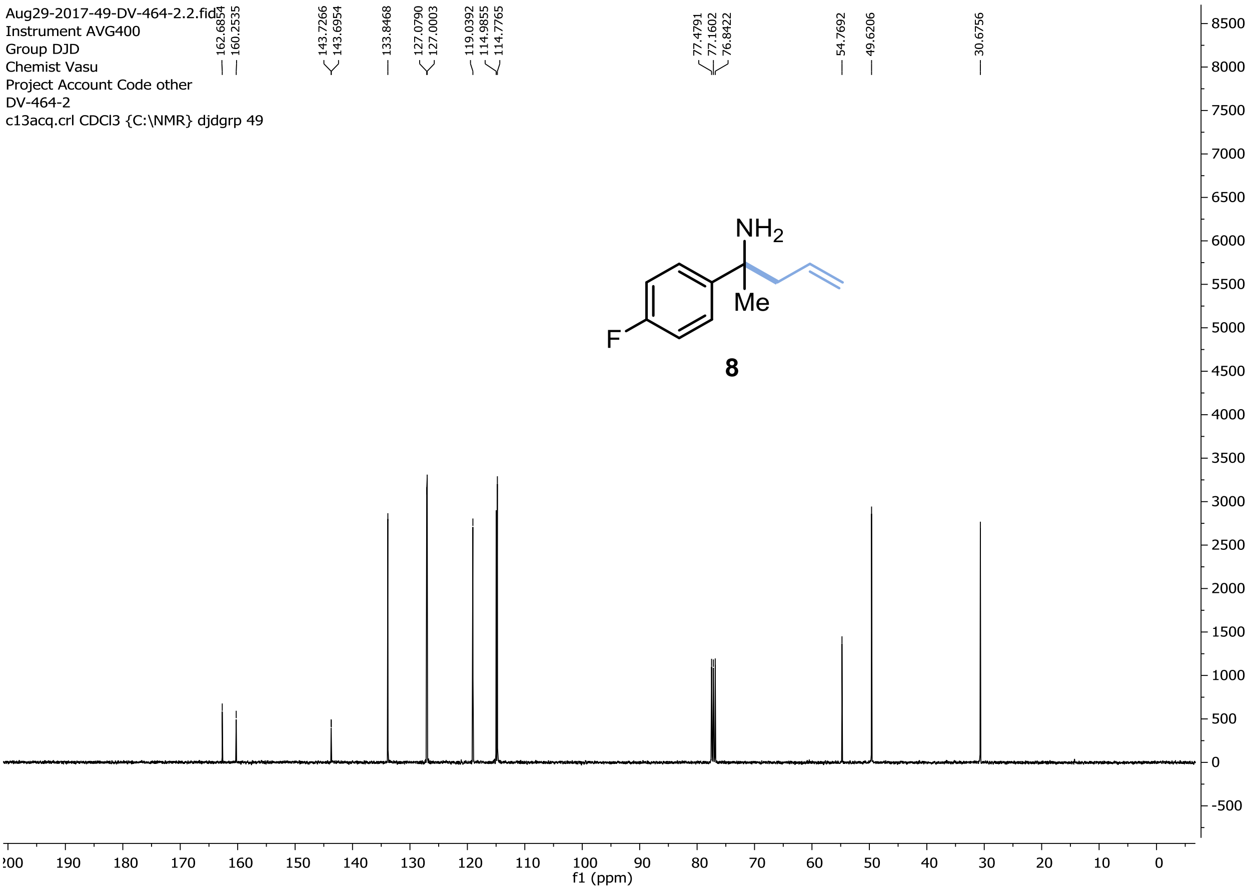

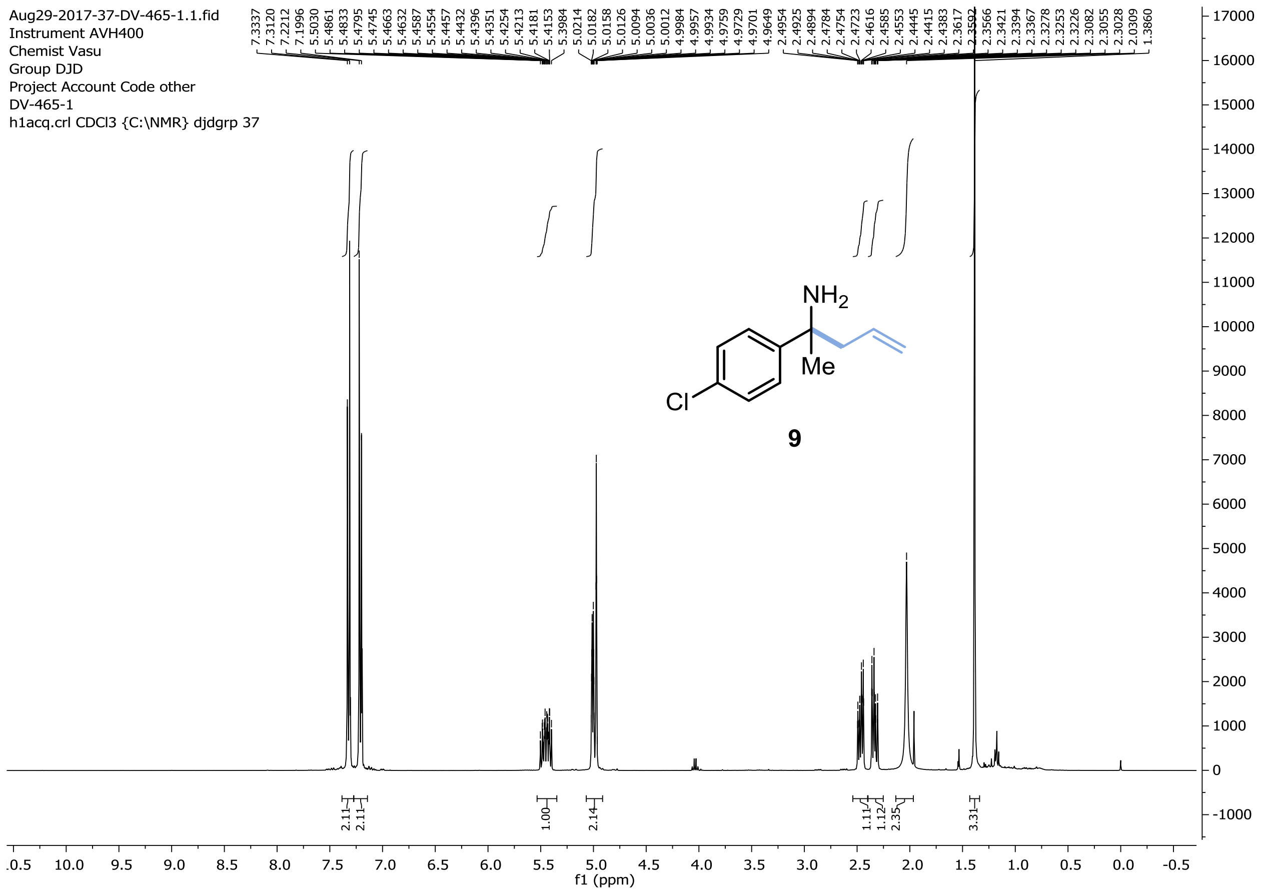
Aug29-2017-37-DV-465-1.1.fid
Instrument AVH400
Chemist Vasu
Group DJD
Project Account Code other
DV-465-1
h1acq.crl CDCl3 {C:\NMR} djdgrp 37
7.3337 7.3120 7.2212 7.1996 5.5030 5.4861 5.4833 5.4795 5.4745 5.4663 5.4632 5.4587 5.4554 5.4457 5.4432 5.4396 5.4351 5.4254 5.4213 5.4181 5.4153 5.3984 5.0214 5.0182 5.0158 5.0126 5.0094 5.0036 5.0012 4.9984 4.9957 4.9934 4.9759 4.9729 4.9701 4.9649 2.4954 2.4925 2.4894 2.4784 2.4754 2.4723 2.4616 2.4585 2.4553 2.4445 2.4415 2.4383 2.3617 2.3592 2.3566 2.3421 2.3394 2.3367 2.3278 2.3253 2.3226 2.3082 2.3055 2.3028 2.0309 1.3860
NH2
Me
Cl
9
2.11 2.11 1.00 2.14 1.11 1.12 2.35 3.31
f1 (ppm)

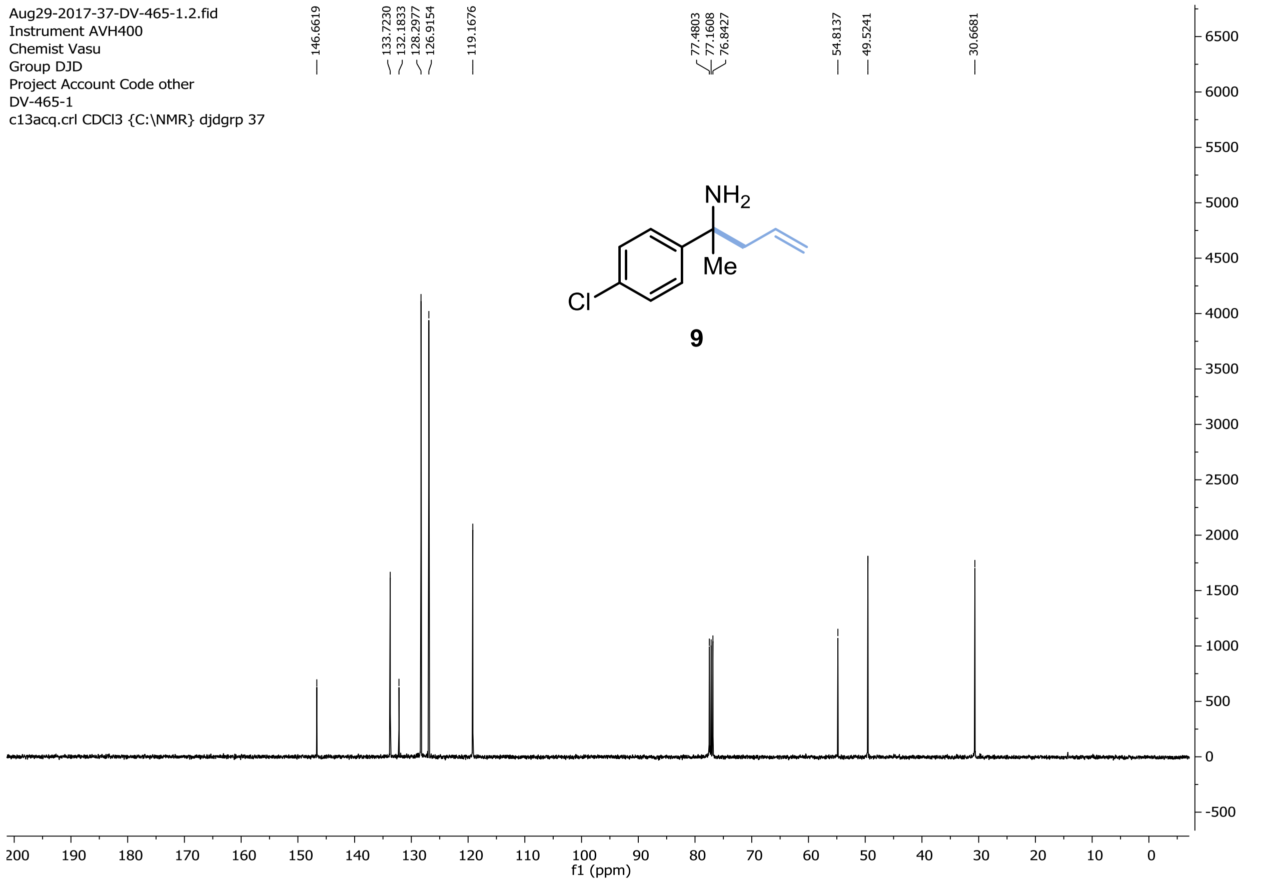
Aug29-2017-37-DV-465-1.2.fid
Instrument AVH400
Chemist Vasu
Group DJD
Project Account Code other
DV-465-1
c13acq.crl CDCl3 {C:\NMR} djdgrp 37
146.6619
133.7230 132.1833 128.2977 126.9154
119.1676
77.4803 77.1608 76.8427
54.8137
49.5241
30.6681
NH2
Me
Cl
9
f1 (ppm)

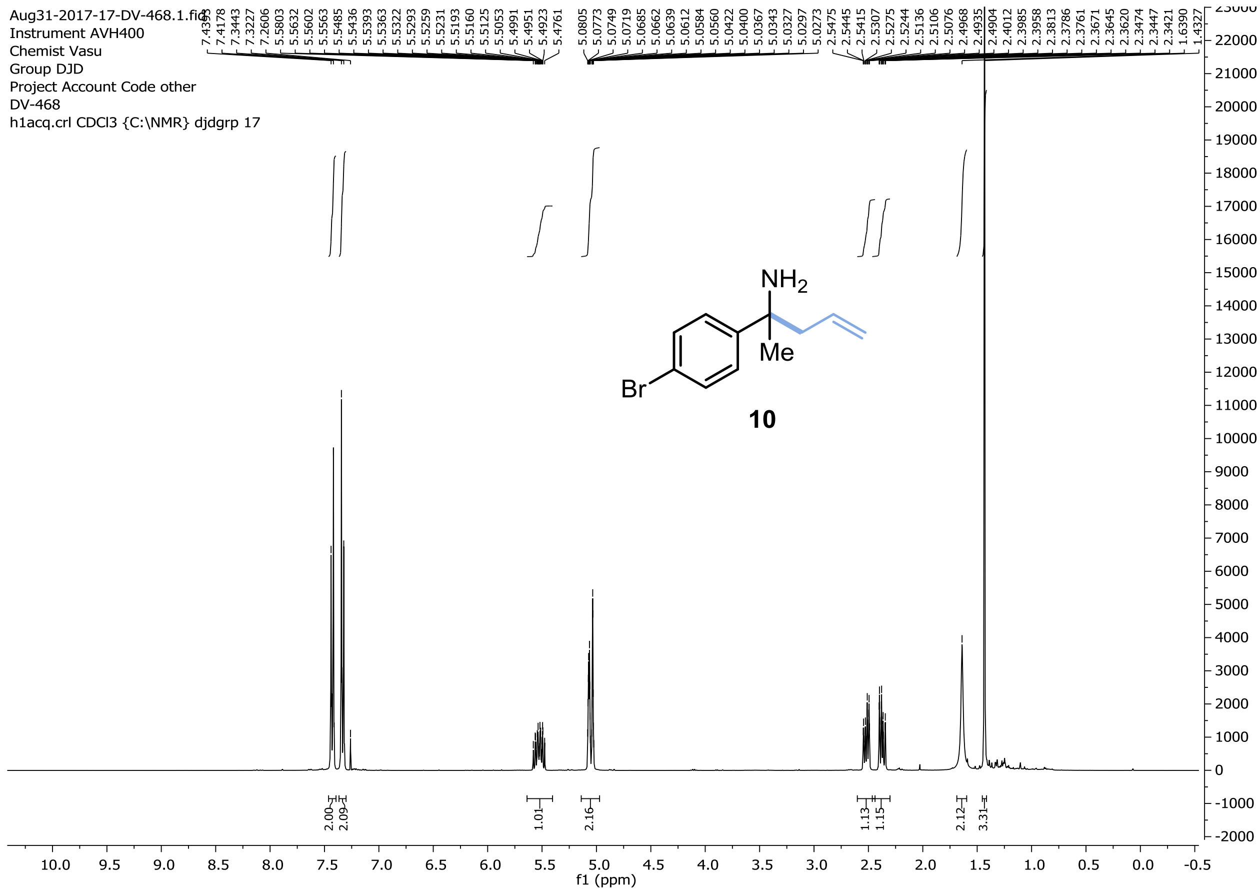
Aug31-2017-17-DV-468.1.fid
Instrument AVH400
Chemist Vasu
Group DJD
Project Account Code other
DV-468
h1acq.crl CDCl3 {C:\NMR} djdgrp 17
NH2
Me
Br
10
f1 (ppm)

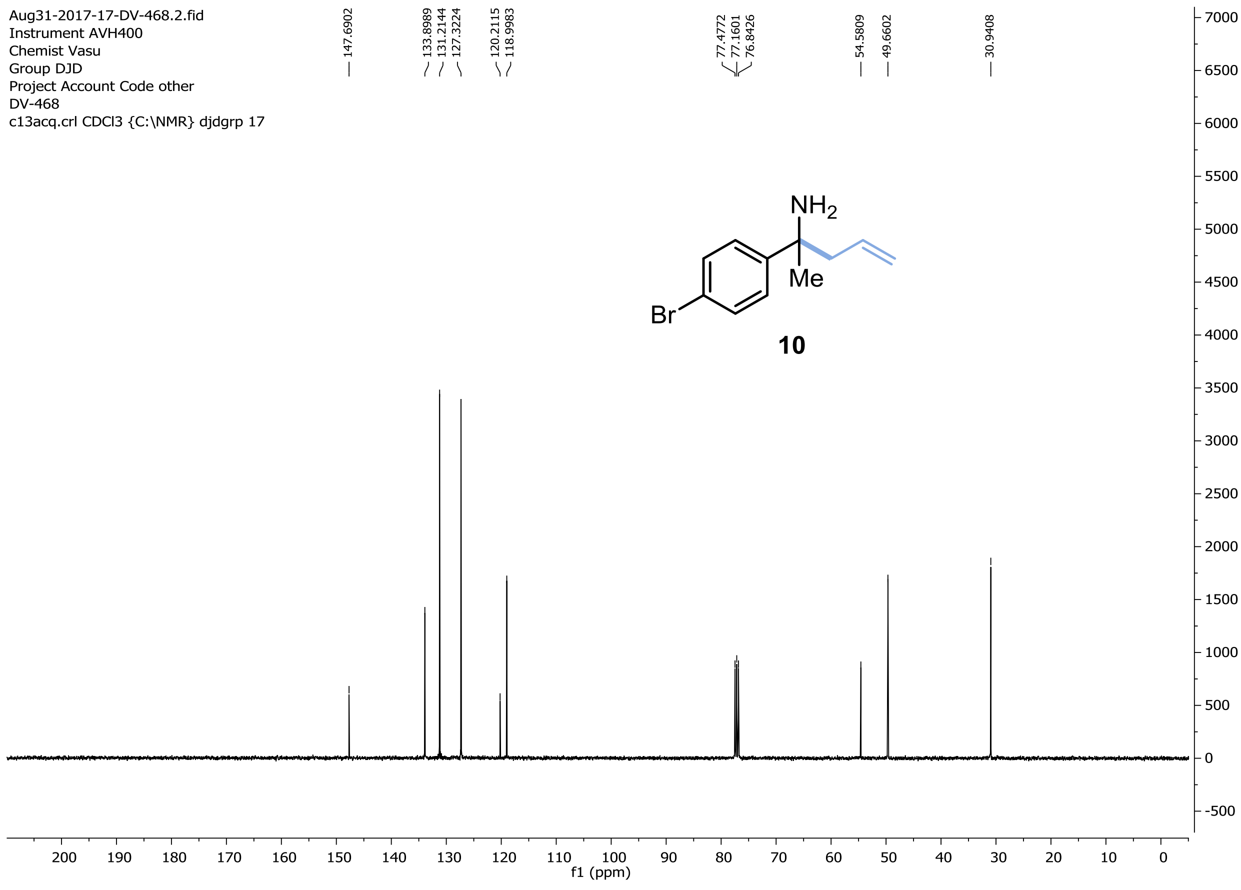
Aug31-2017-17-DV-468.2.fid
Instrument AVH400
Chemist Vasu
Group DJD
Project Account Code other
DV-468
c13acq.crl CDCl3 {C:\NMR} djdgrp 17
147.6902
133.8989
131.2144
127.3224
120.2115
118.9983
77.4772
77.1601
76.8426
54.5809
49.6602
30.9408
NH2
Me
Br
10
f1 (ppm)

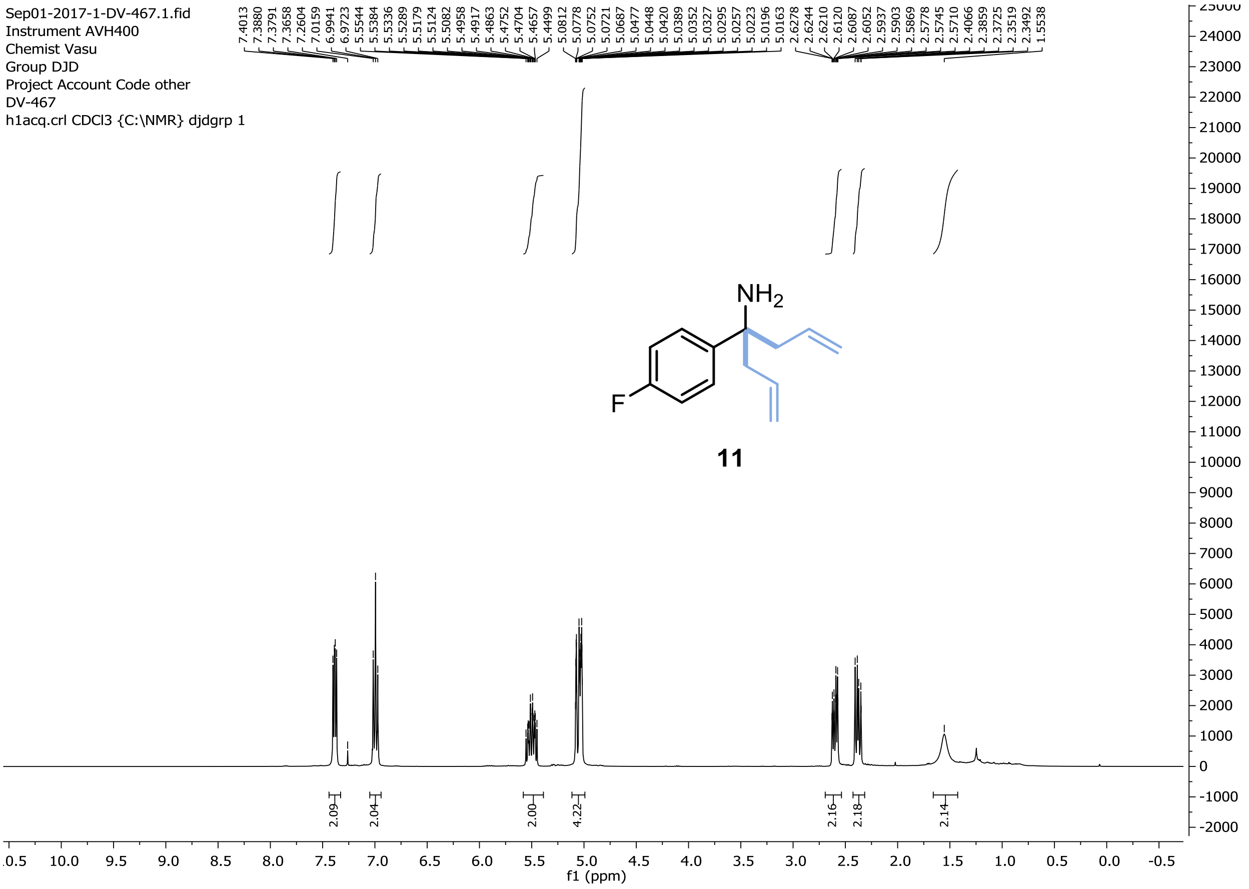
Sep01-2017-1-DV-467.1.fid
Instrument AVH400
Chemist Vasu
Group DJD
Project Account Code other
DV-467
h1acq.crl CDCl3 {C:\NMR} djdgrp 1
NH2
F
11
f1 (ppm)

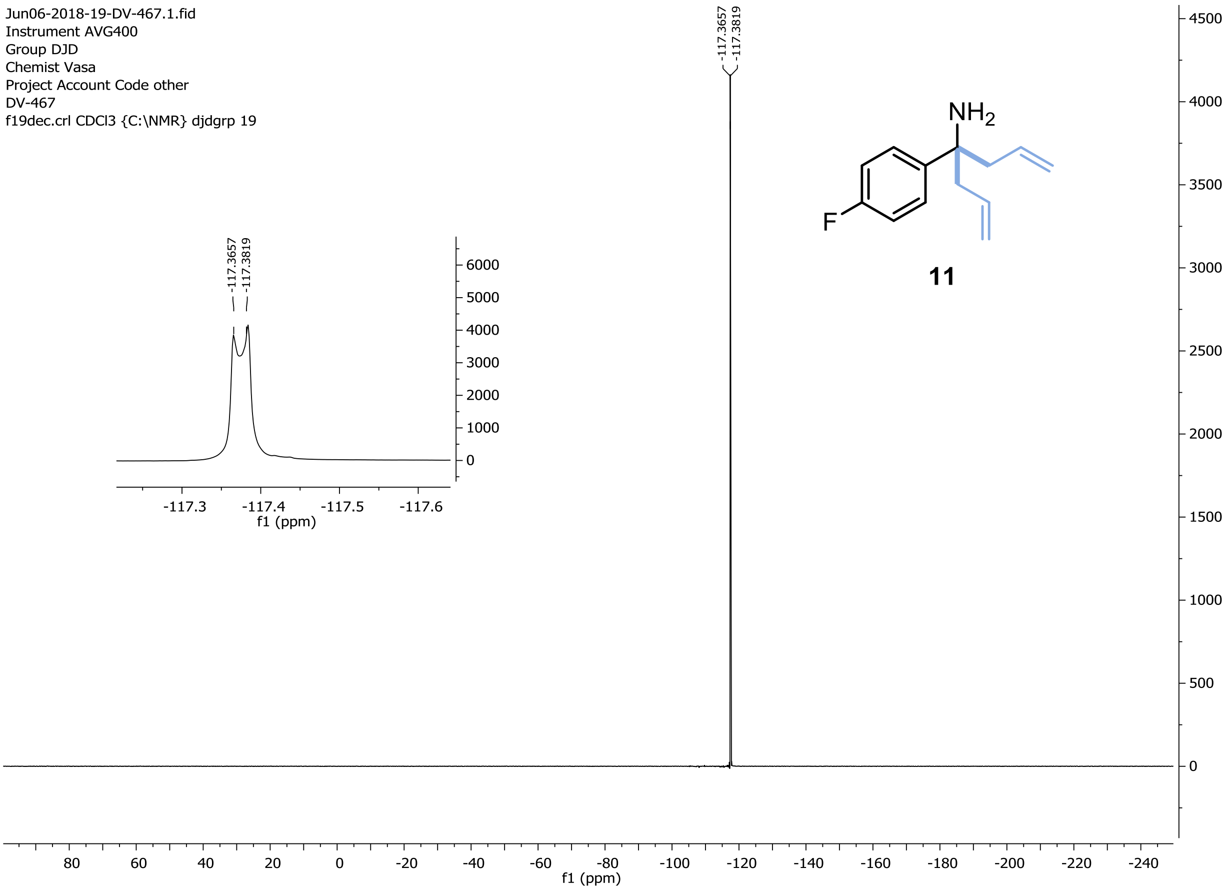
Jun06-2018-19-DV-467.1.fid
Instrument AVG400
Group DJD
Chemist Vasa
Project Account Code other
DV-467
f19dec.crl CDCl3 {C:\NMR} djdgrp 19
-117.3657
-117.3819
NH2
F
11
f1 (ppm)

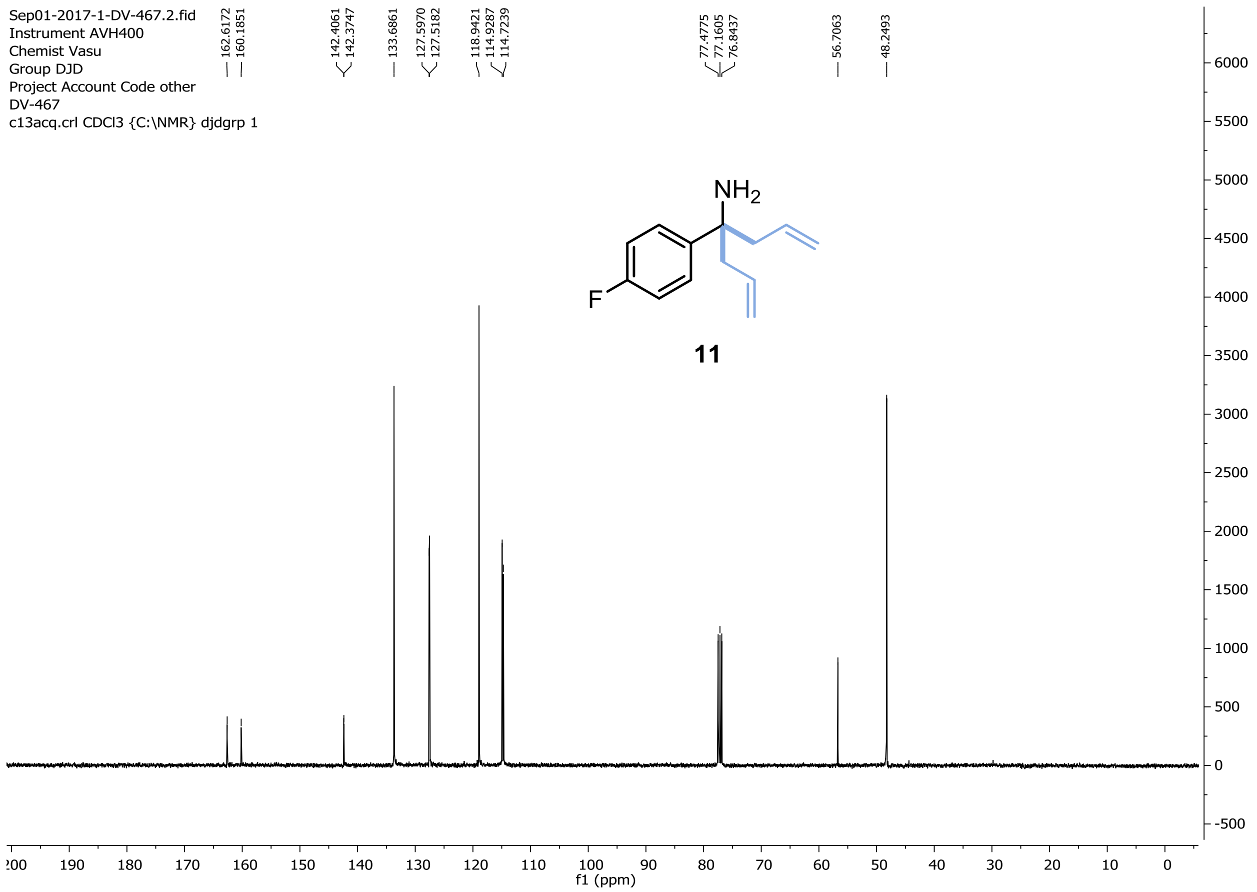
Sep01-2017-1-DV-467.2.fid
Instrument AVH400
Chemist Vasu
Group DJD
Project Account Code other
DV-467
c13acq.crl CDCl3 {C:\NMR} djdgrp 1
162.6172
160.1851
142.4061
142.3747
133.6861
127.5970
127.5182
118.9421
114.9287
114.7239
77.4775
77.1605
76.8437
56.7063
48.2493
NH2
F
11
f1 (ppm)

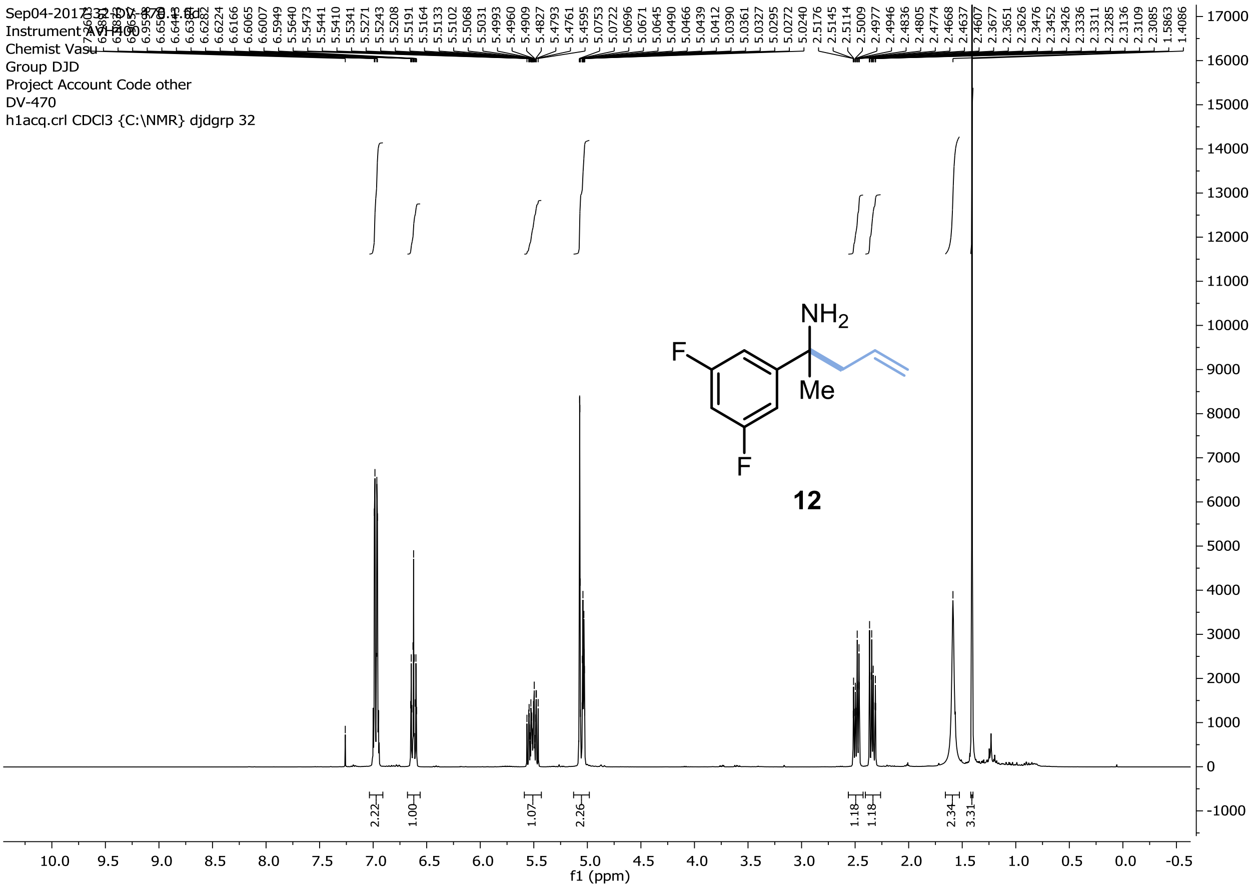
Sep04-2017-32-DV-470.1.fid
Instrument AVH400
Chemist Vasu
Group DJD
Project Account Code other
DV-470
h1acq.crl CDCl3 {C:\NMR} djdgrp 32
NH2
F
Me
F
12
2.22
1.00
1.07
2.26
1.18
1.18
2.34
3.31
f1 (ppm)

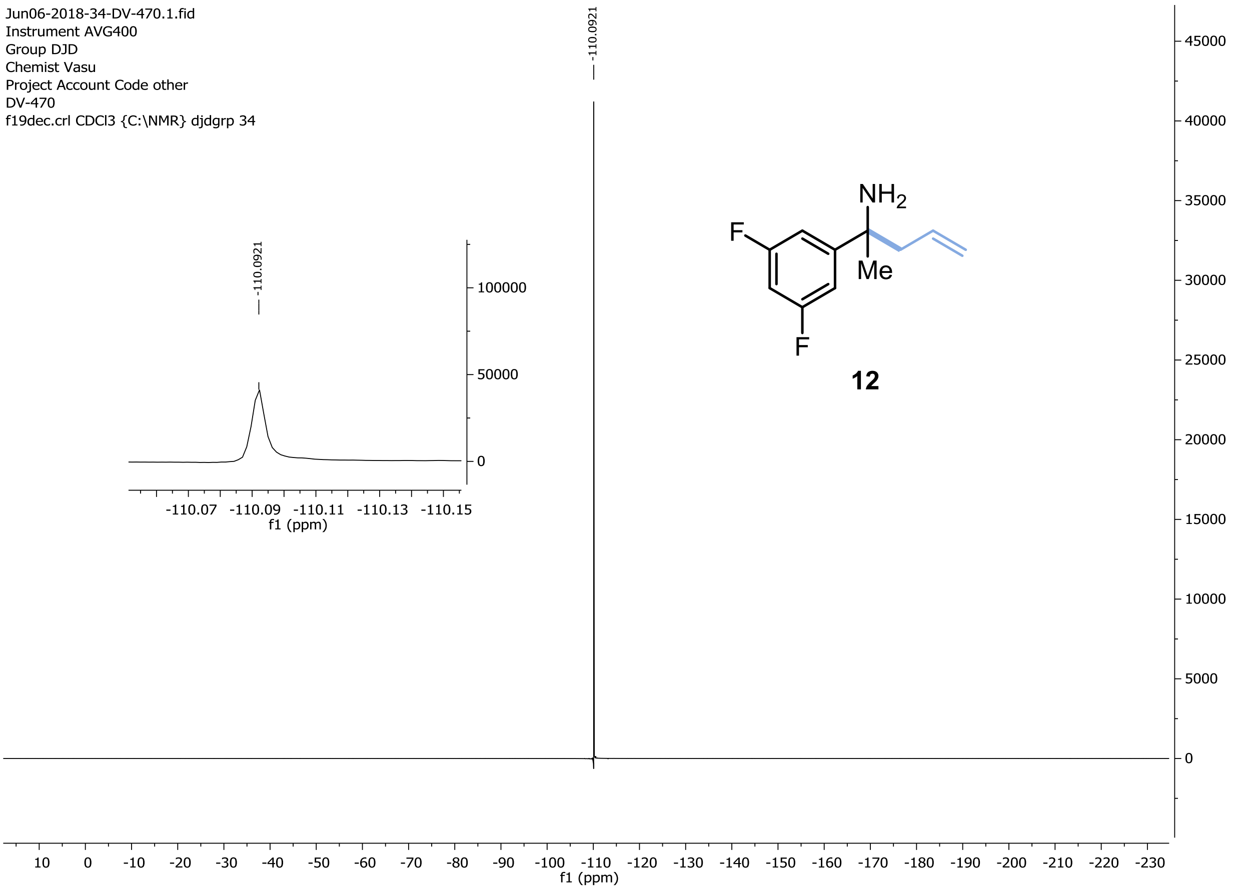
Jun06-2018-34-DV-470.1.fid
Instrument AVG400
Group DJD
Chemist Vasu
Project Account Code other
DV-470
f19dec.crl CDCl3 {C:\NMR} djdgrp 34
-110.0921
NH2
F
Me
F
12
f1 (ppm)

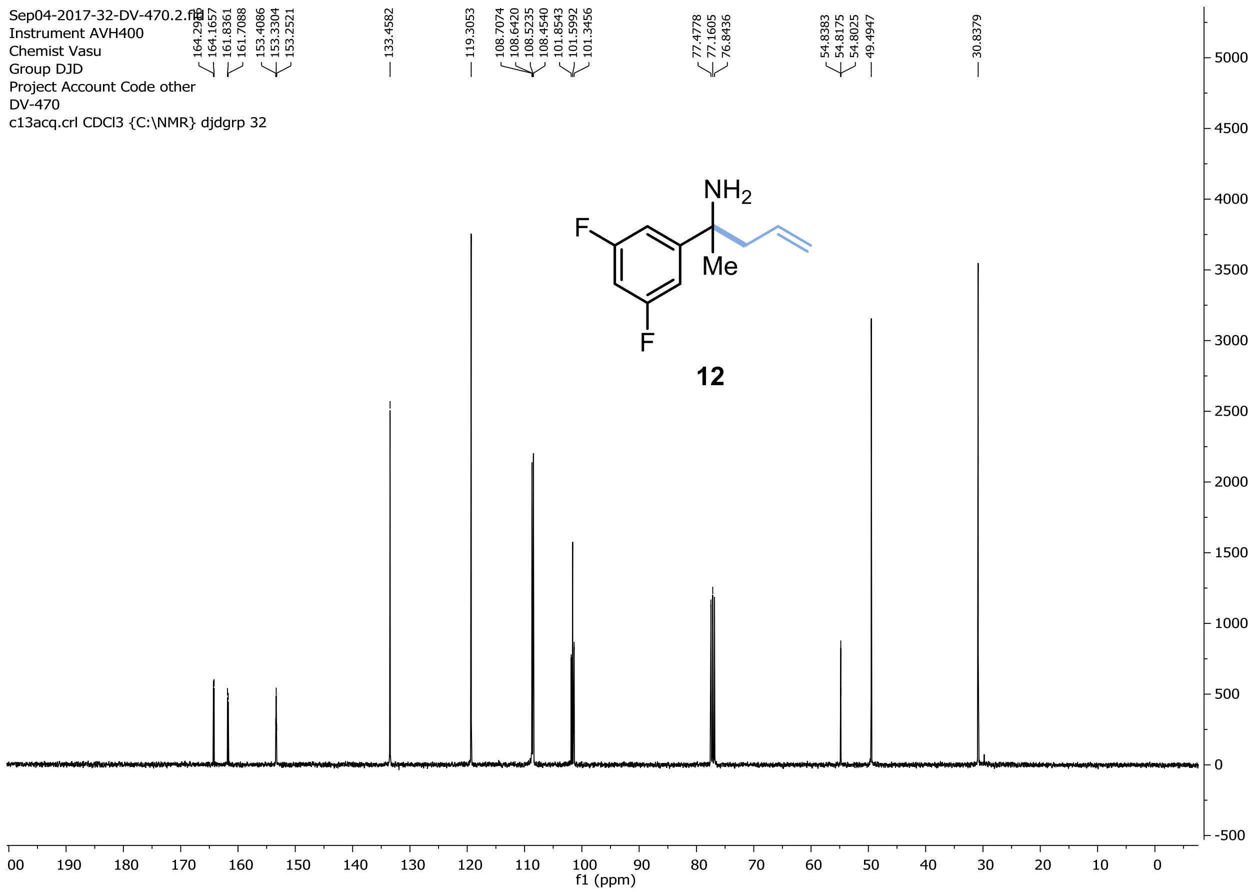
Sep04-2017-32-DV-470.2.fid
Instrument AVH400
Chemist Vasu
Group DJD
Project Account Code other
DV-470
c13acq.crl CDCl3 {C:\NMR} djdgrp 32
164.2993
164.1657
161.8361
161.7088
153.4086
153.3304
153.2521
133.4582
119.3053
108.7074
108.6420
108.5235
108.4540
101.8543
101.5992
101.3456
77.4778
77.1605
76.8436
54.8383
54.8175
54.8025
49.4947
30.8379
NH2
F
Me
F
12
f1 (ppm)

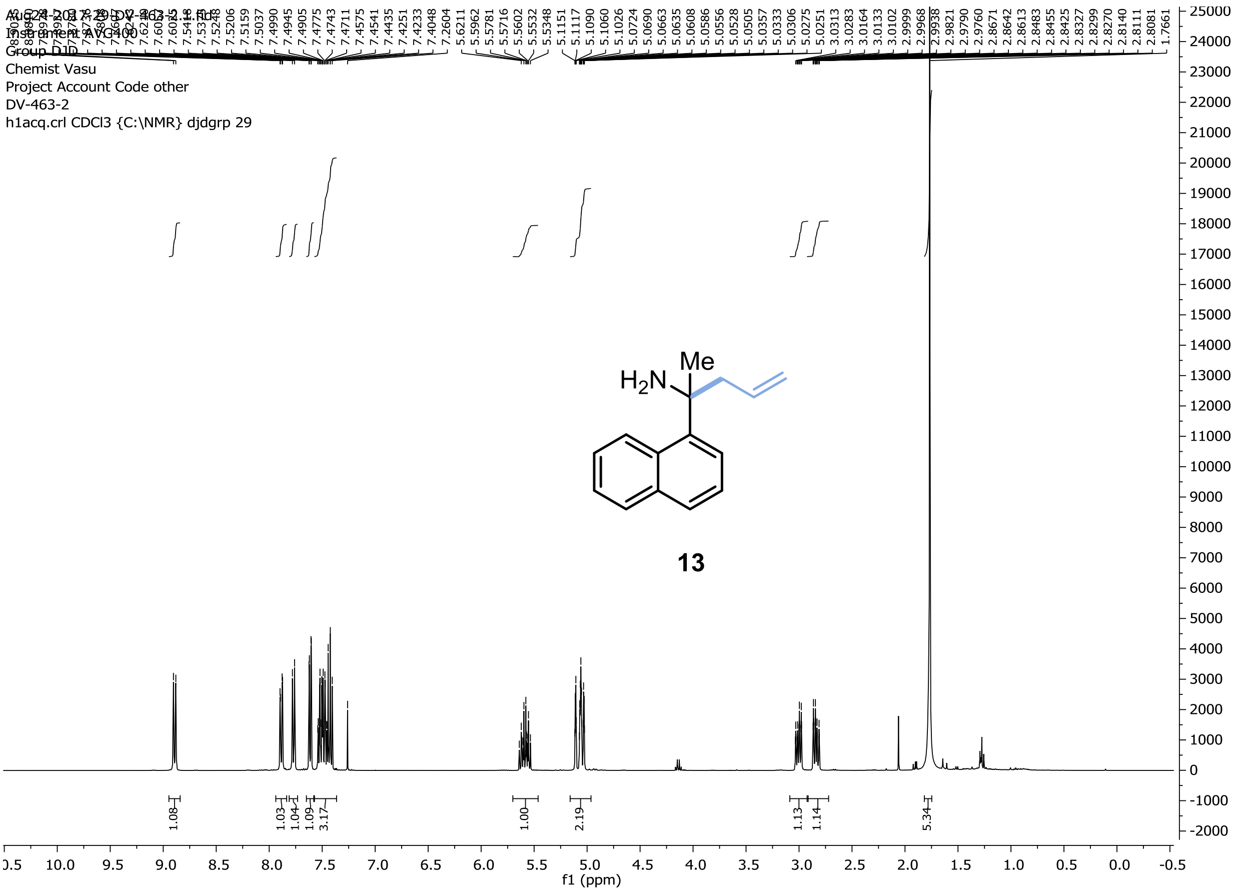
Instrument AVG400
Group DJD
Chemist Vasu
Project Account Code other
DV-463-2
h1acq.crl CDCl3 {C:\NMR} djdgrp 29
Me
H2N
13
1.08
1.03
1.04
1.09
3.17
1.00
2.19
1.13
1.14
5.34
f1 (ppm)

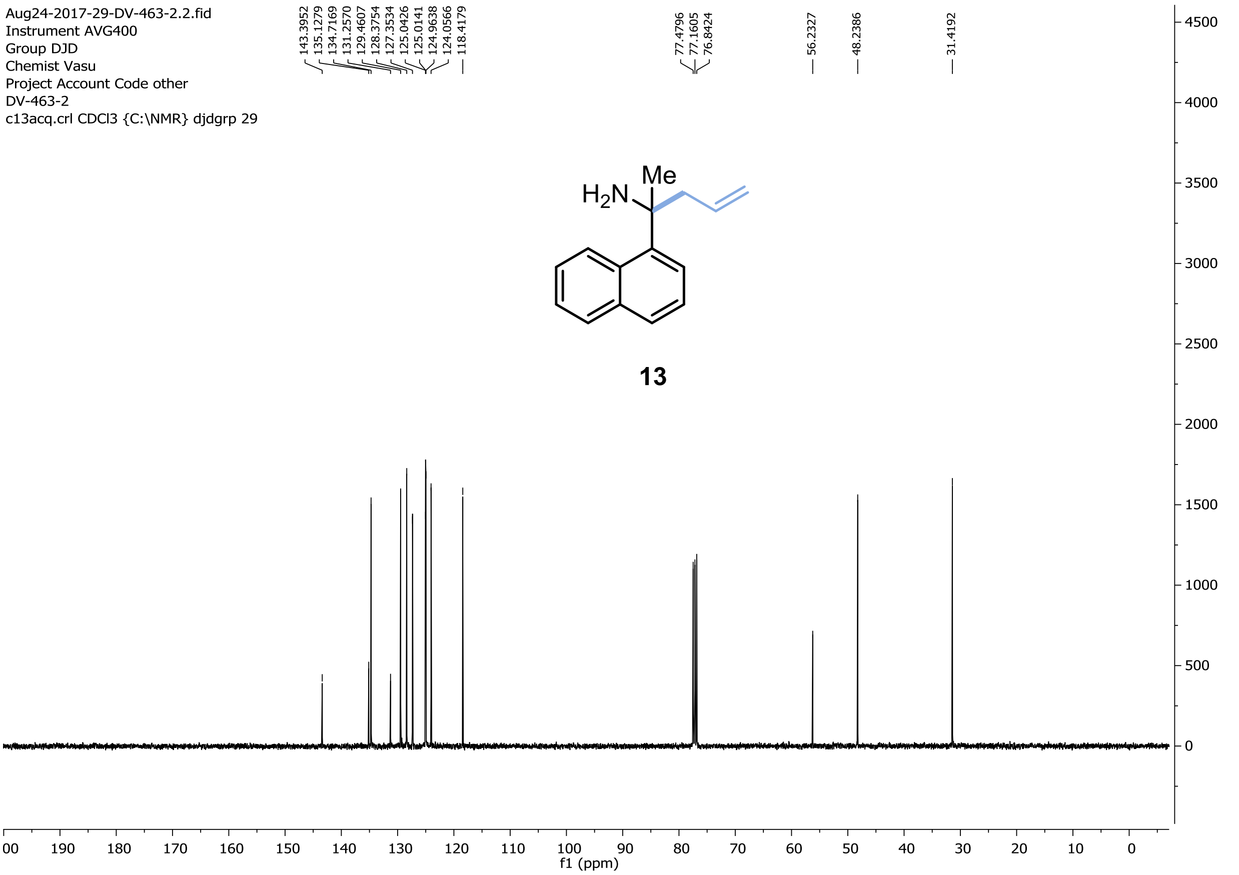
Aug24-2017-29-DV-463-2.2.fid
Instrument AVG400
Group DJD
Chemist Vasu
Project Account Code other
DV-463-2
c13acq.crl CDCl3 {C:\NMR} djdgrp 29
143.3952
135.1279
134.7169
131.2570
129.4607
128.3754
127.3534
125.0426
125.0141
124.9638
124.0566
118.4179
77.4796
77.1605
76.8424
56.2327
48.2386
31.4192
Me
H2N
13
f1 (ppm)

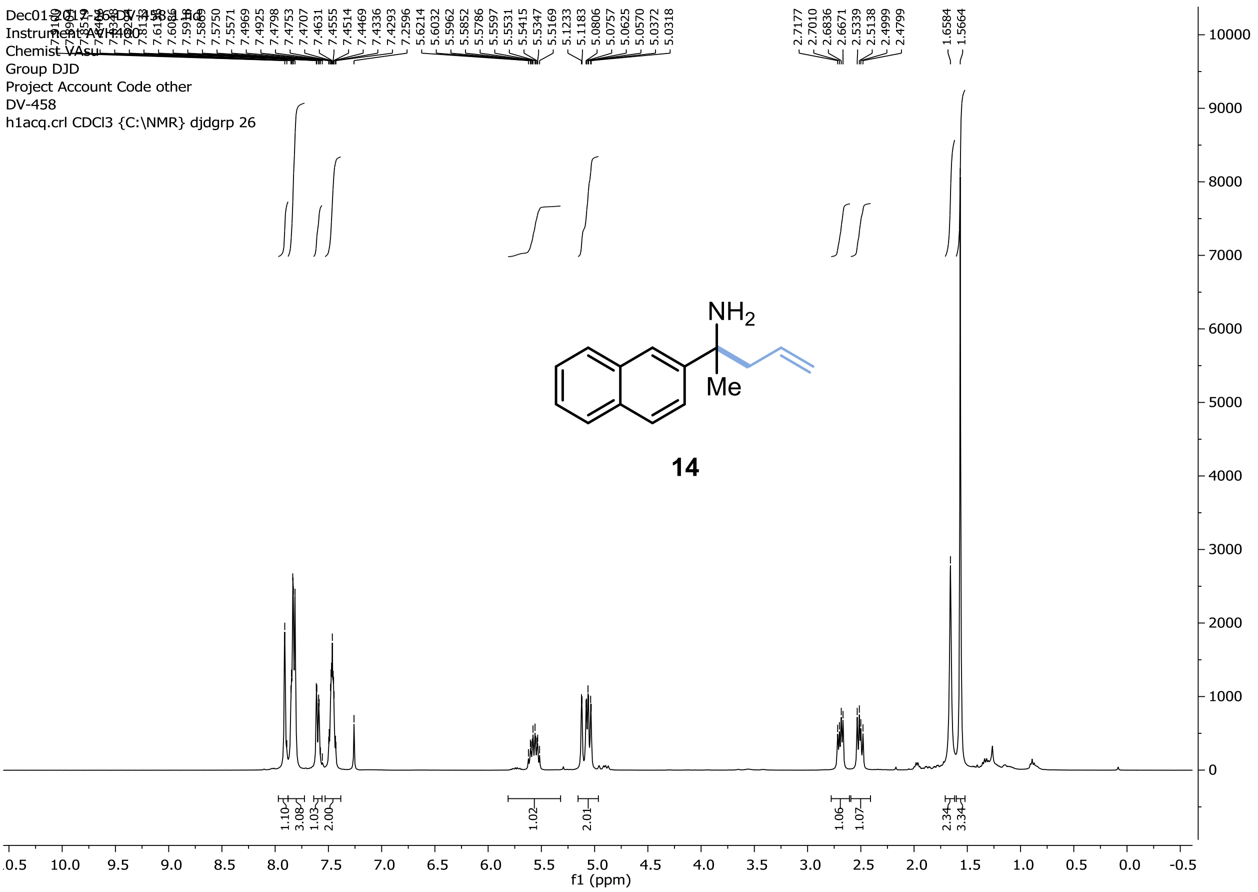
Instrument AVH400
Chemist VAsu
Group DJD
Project Account Code other
DV-458
h1acq.crl CDCl3 {C:\NMR} djdgrp 26
2.7177
2.7010
2.6836
2.6671
2.5339
2.5138
2.4999
2.4799
1.6584
1.5664
NH2
Me
14
1.10
3.08
1.03
2.00
1.02
2.01
1.06
1.07
2.34
3.34
f1 (ppm)

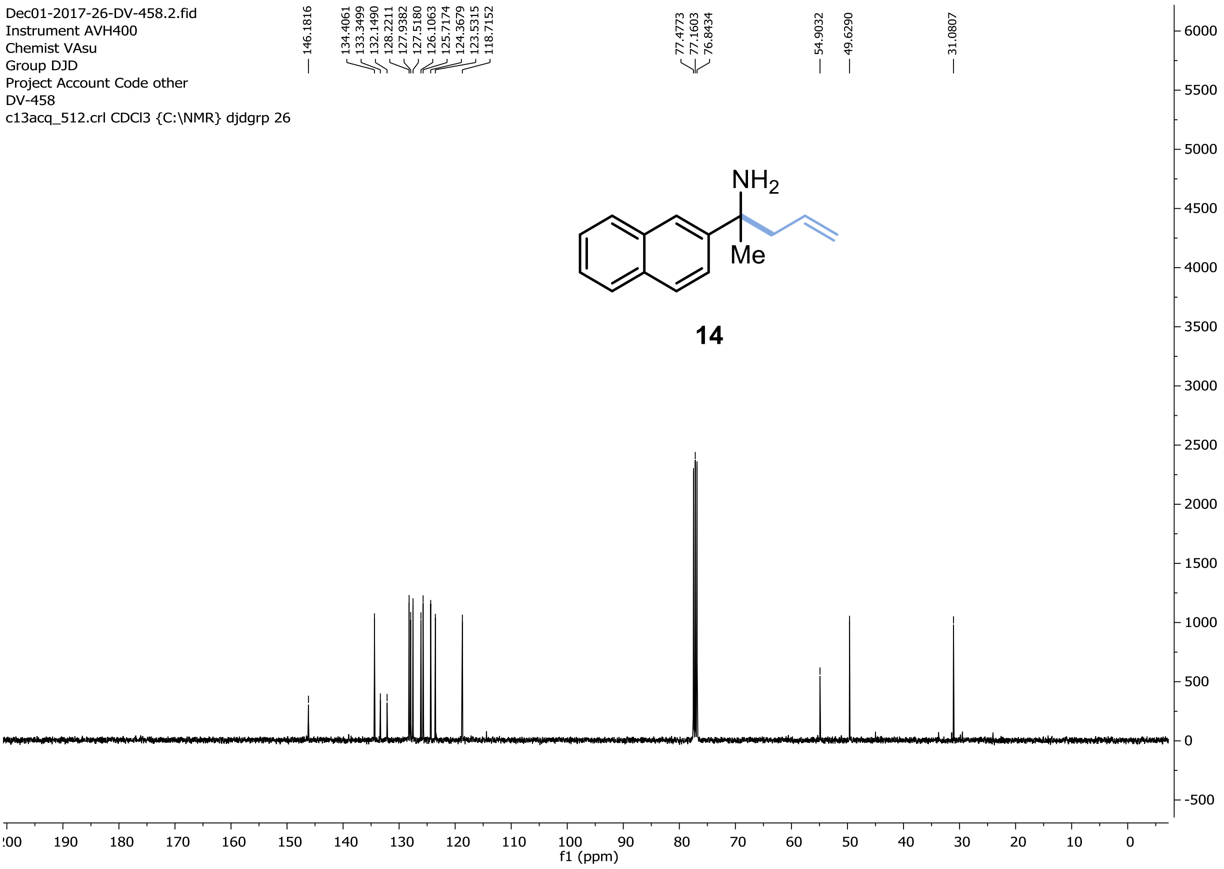
Dec01-2017-26-DV-458.2.fid
Instrument AVH400
Chemist VAsu
Group DJD
Project Account Code other
DV-458
c13acq_512.crl CDCl3 {C:\NMR} djdgrp 26
146.1816
134.4061
133.3499
132.1490
128.2211
127.9382
127.5180
126.1063
125.7174
124.3679
123.5315
118.7152
77.4773
77.1603
76.8434
54.9032
49.6290
31.0807
NH2
Me
14
f1 (ppm)

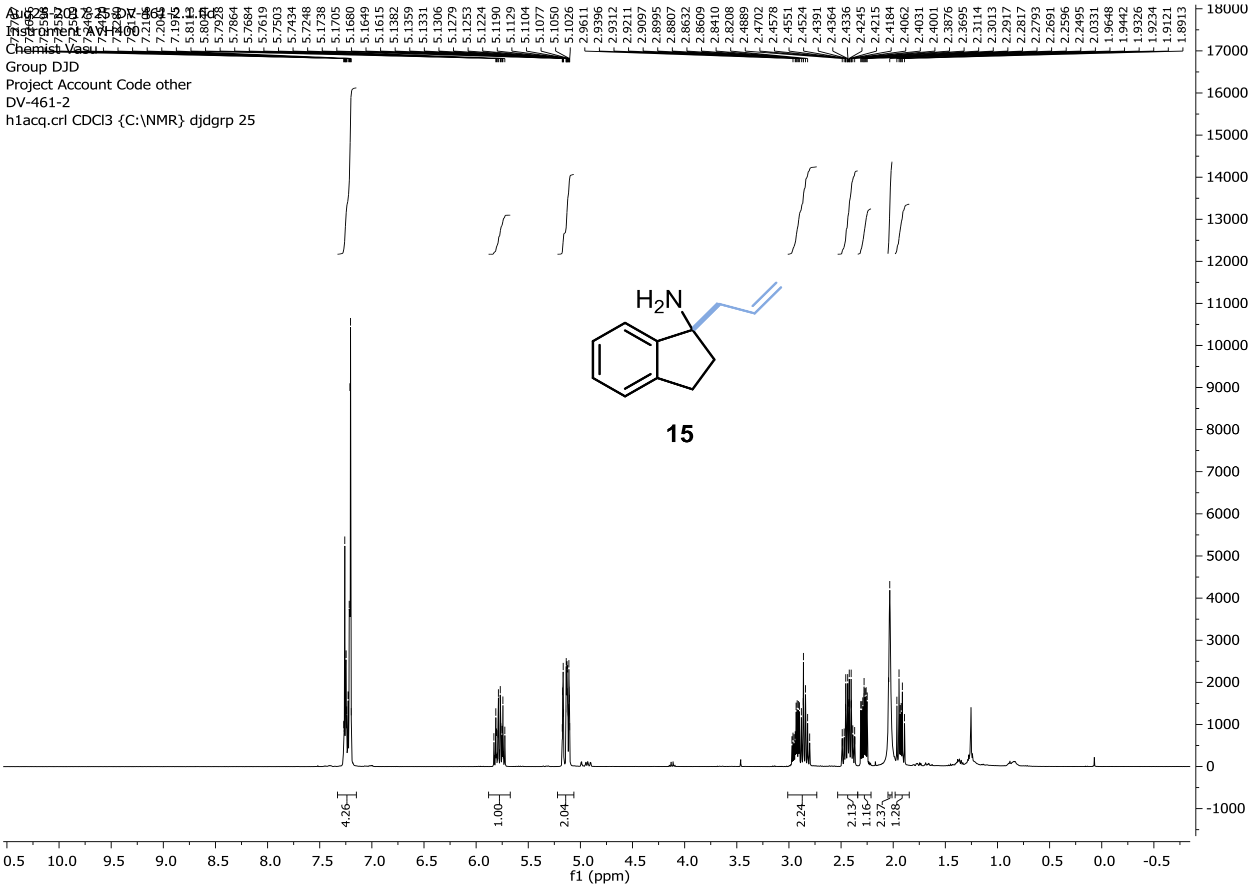
Instrument AVH400
Chemist Vasu
Group DJD
Project Account Code other
DV-461-2
h1acq.crl CDCl3 {C:\NMR} djdgrp 25
H2N
15
4.26
1.00
2.04
2.24
2.13
1.16
2.37
1.28
f1 (ppm)

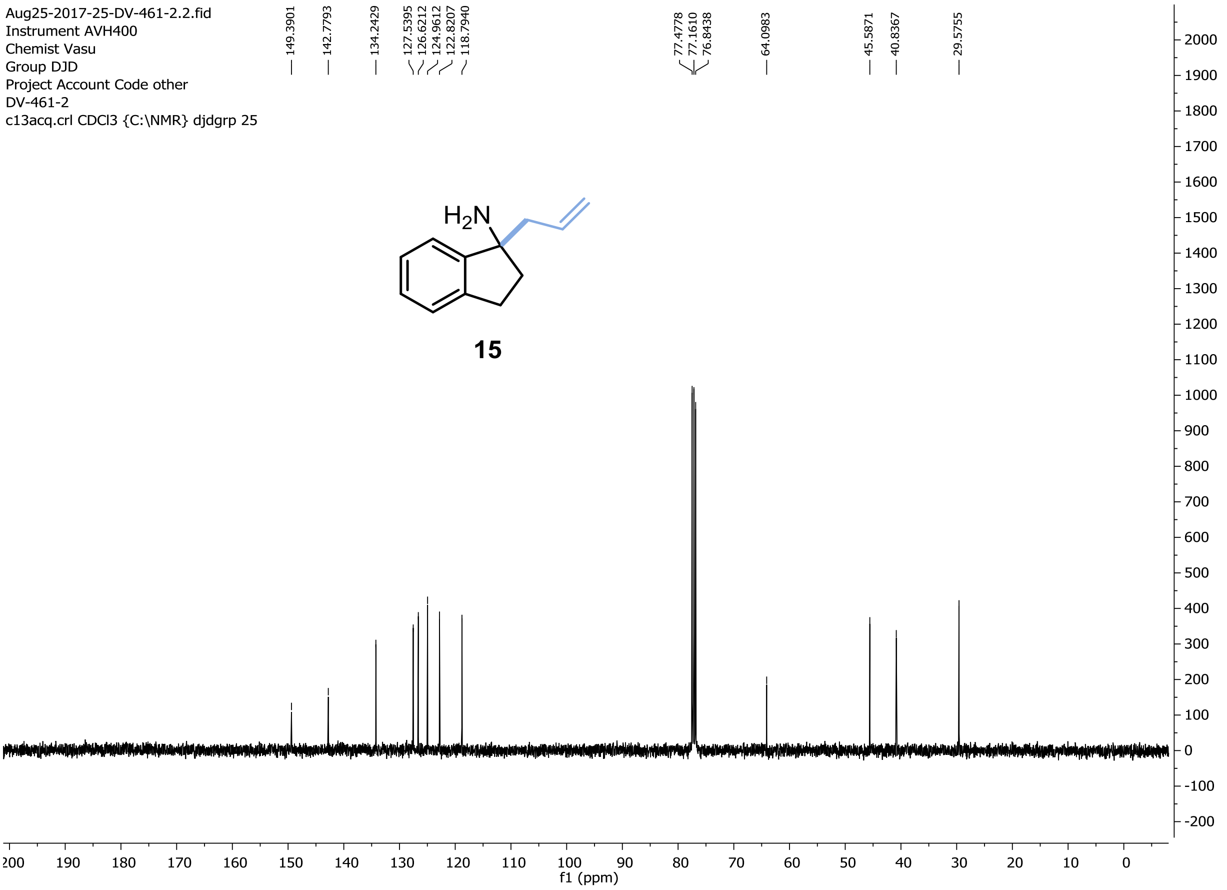
Aug25-2017-25-DV-461-2.2.fid
Instrument AVH400
Chemist Vasu
Group DJD
Project Account Code other
DV-461-2
c13acq.crl CDCl3 {C:\NMR} djdgrp 25
149.3901
142.7793
134.2429
127.5395
126.6212
124.9612
122.8207
118.7940
77.4778
77.1610
76.8438
64.0983
45.5871
40.8367
29.5755
H2N
15
f1 (ppm)

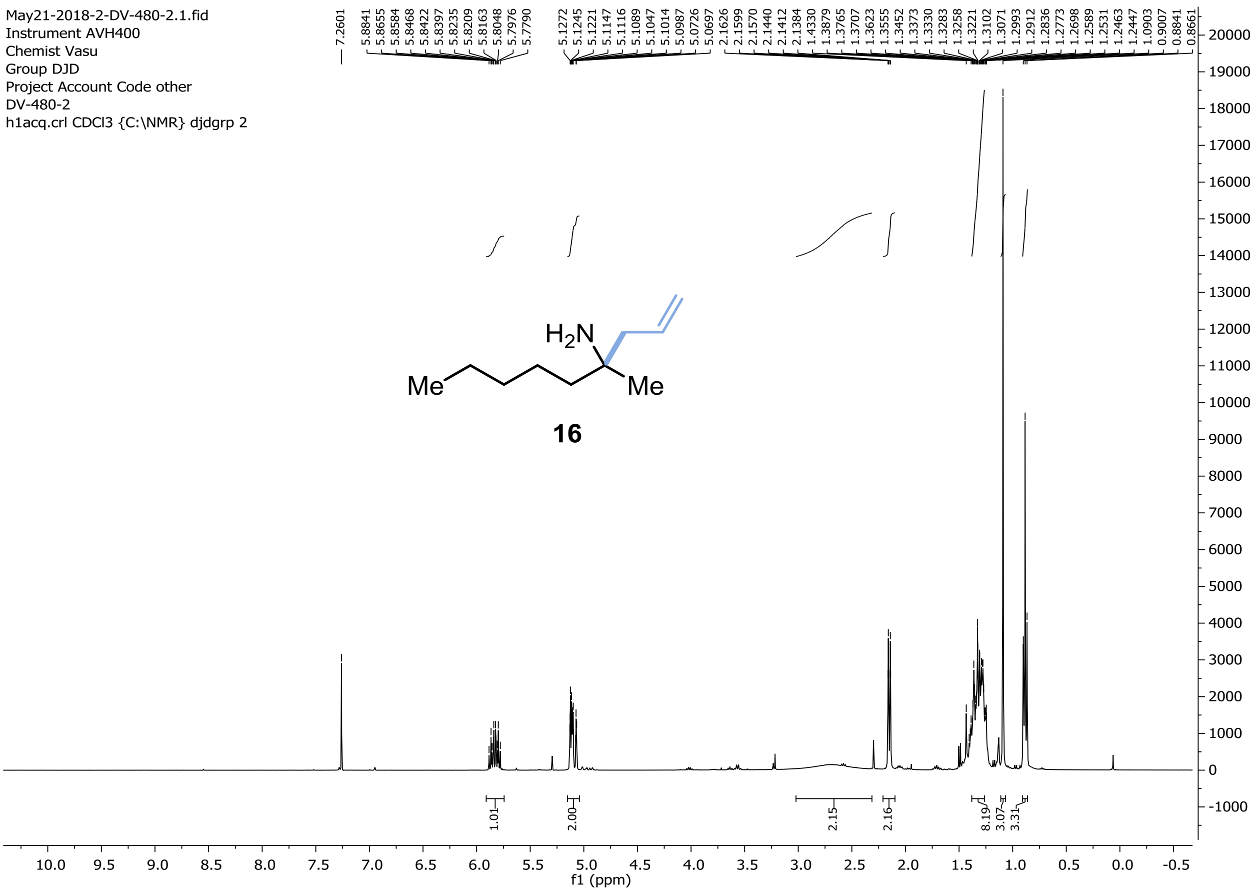
May21-2018-2-DV-480-2.1.fid
Instrument AVH400
Chemist Vasu
Group DJD
Project Account Code other
DV-480-2
h1acq.crl CDCl3 {C:\NMR} djdgrp 2
7.2601
5.8841 5.8655 5.8584 5.8468 5.8422 5.8397 5.8235 5.8209 5.8163 5.8048 5.7976 5.7790
5.1272 5.1245 5.1221 5.1147 5.1116 5.1089 5.1047 5.1014 5.0987 5.0726 5.0697
2.1626 2.1599 2.1570 2.1440 2.1412 2.1384
1.4330 1.3879 1.3765 1.3707 1.3623 1.3555 1.3452 1.3373 1.3330 1.3283 1.3258 1.3221 1.3102 1.3071 1.2993 1.2912 1.2836 1.2773 1.2698 1.2589 1.2531 1.2463 1.2447 1.0903 0.9007 0.8841 0.8661
H2N
Me
Me
16
1.01
2.00
2.15
2.16
8.19
3.07
3.31
f1 (ppm)

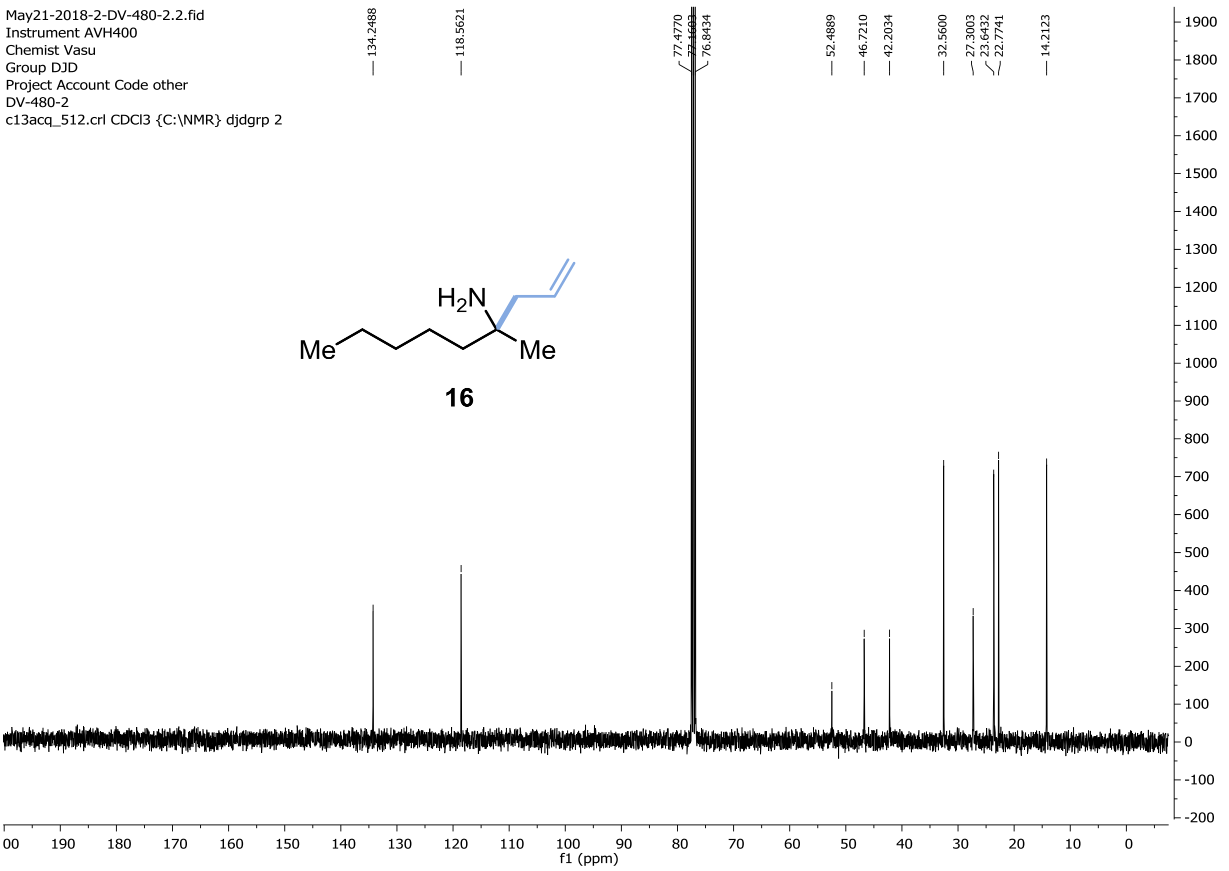
May21-2018-2-DV-480-2.2.fid
Instrument AVH400
Chemist Vasu
Group DJD
Project Account Code other
DV-480-2
c13acq_512.crl CDCl3 {C:\NMR} djdgrp 2
134.2488
118.5621
77.4770
77.1603
76.8434
52.4889
46.7210
42.2034
32.5600
27.3003
23.6432
22.7741
14.2123
H2N
Me
Me
16
f1 (ppm)

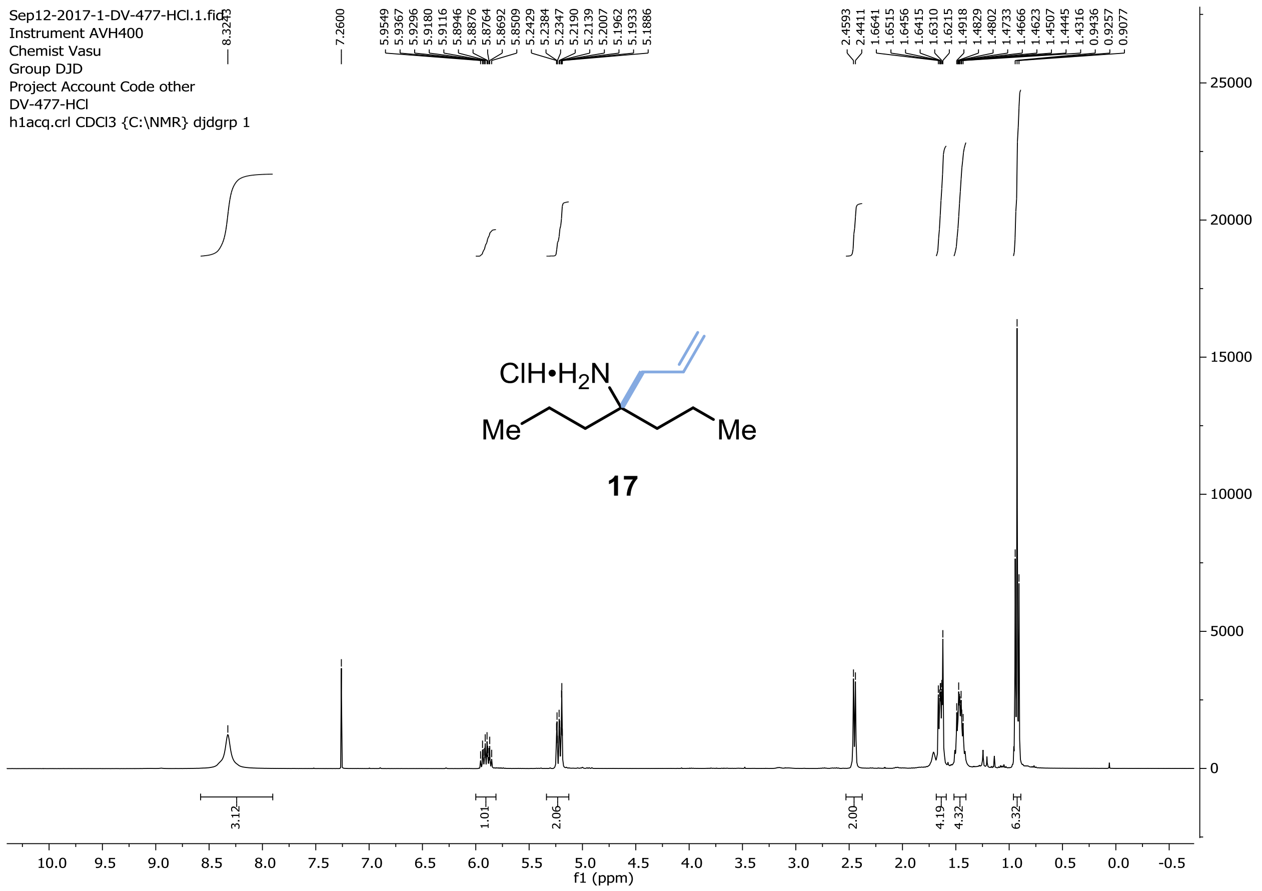
Sep12-2017-1-DV-477-HCl.1.fid
Instrument AVH400
Chemist Vasu
Group DJD
Project Account Code other
DV-477-HCl
h1acq.crl CDCl3 {C:\NMR} djdgrp 1
ClH•H2N
Me
Me
17
3.12
1.01
2.06
2.00
4.19
4.32
6.32
f1 (ppm)

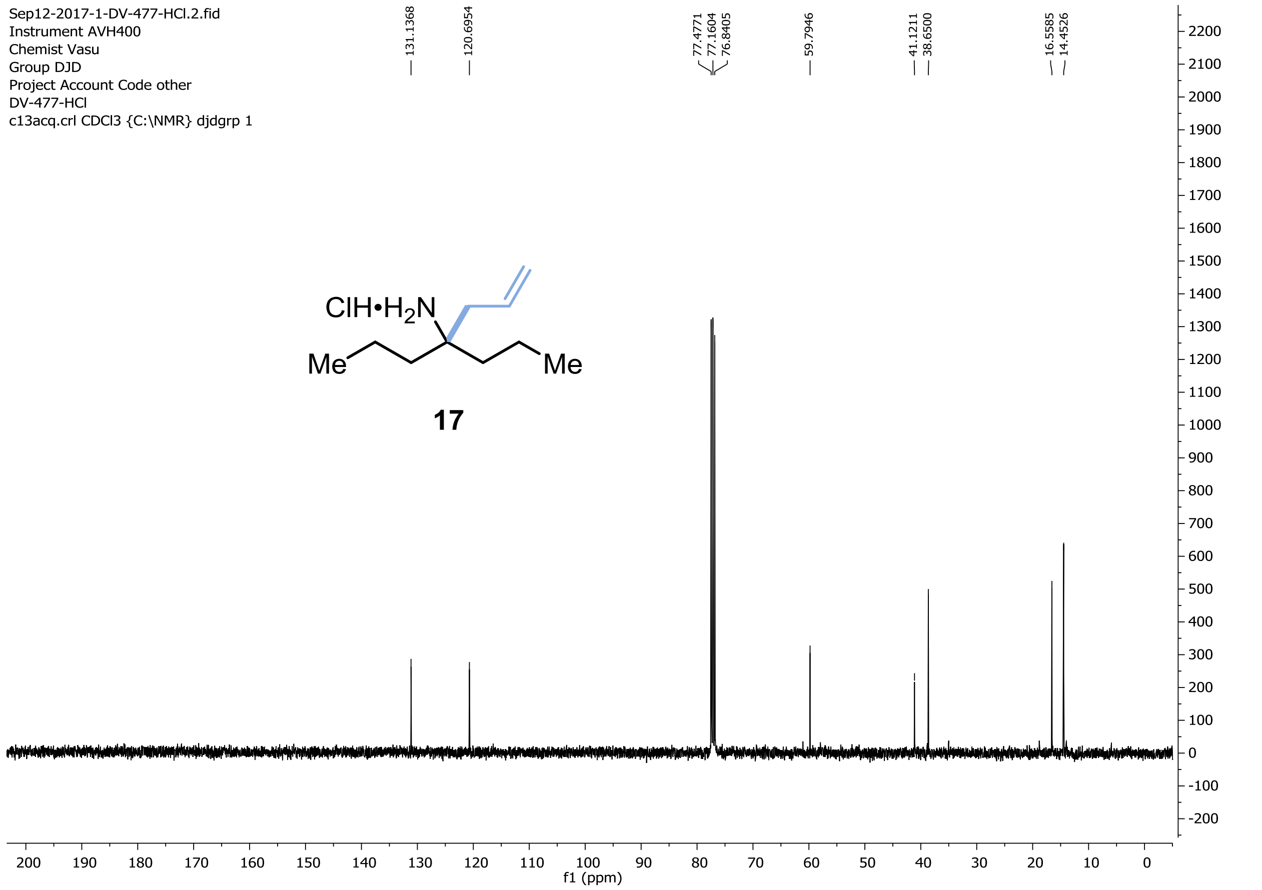
Sep12-2017-1-DV-477-HCl.2.fid
Instrument AVH400
Chemist Vasu
Group DJD
Project Account Code other
DV-477-HCl
c13acq.crl CDCl3 {C:\NMR} djdgrp 1
131.1368
120.6954
77.4771
77.1604
76.8405
59.7946
41.1211
38.6500
16.5585
14.4526
ClH•H2N
Me
Me
17
f1 (ppm)

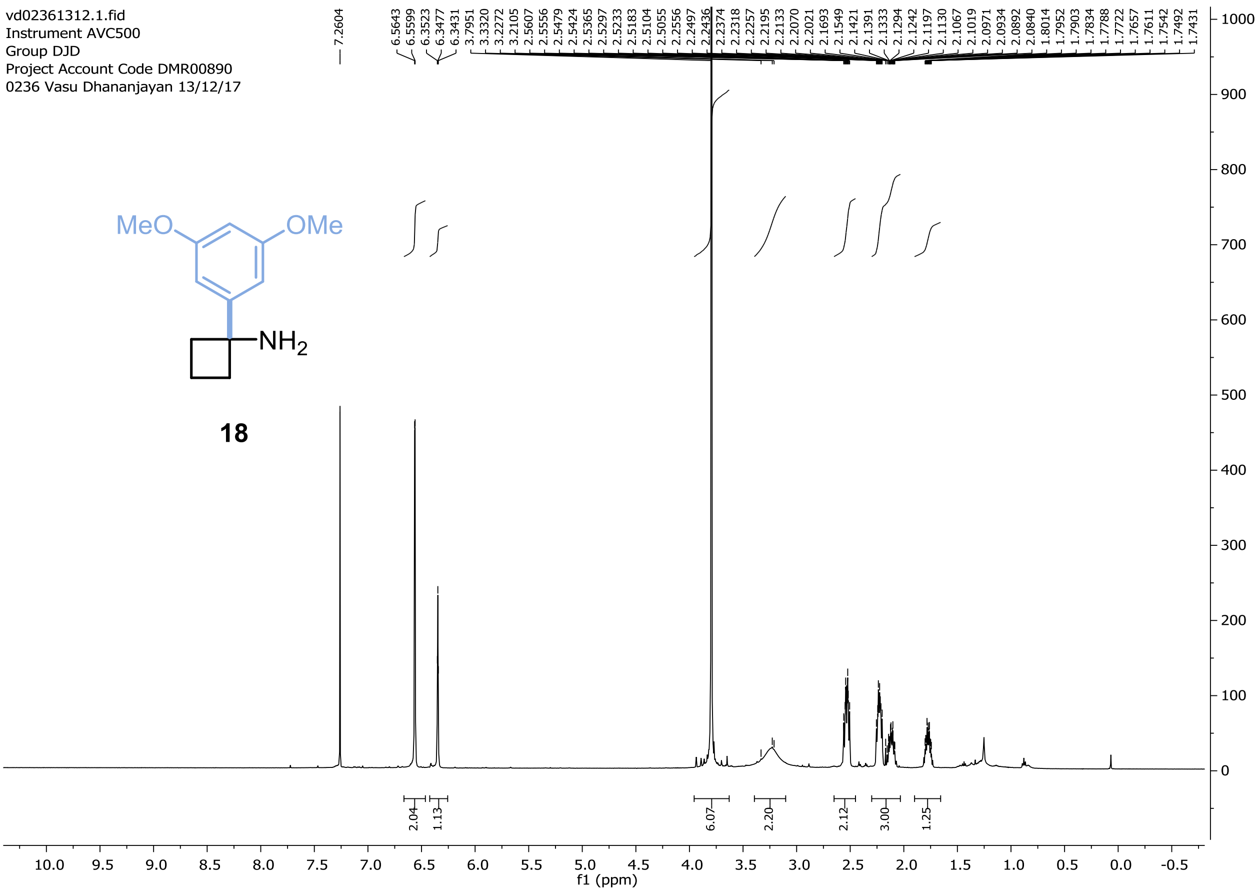
vd02361312.1.fid
Instrument AVC500
Group DJD
Project Account Code DMR00890
0236 Vasu Dhananjayan 13/12/17
7.2604
6.5643
6.5599
6.3523
6.3477
6.3431
3.7951
3.3320
3.2272
3.2105
2.5607
2.5556
2.5479
2.5424
2.5365
2.5297
2.5233
2.5183
2.5104
2.5055
2.2556
2.2497
2.2436
2.2374
2.2318
2.2257
2.2195
2.2133
2.2070
2.2021
2.1693
2.1549
2.1421
2.1391
2.1333
2.1294
2.1242
2.1197
2.1130
2.1067
2.1019
2.0971
2.0934
2.0892
2.0840
1.8014
1.7952
1.7903
1.7834
1.7788
1.7722
1.7657
1.7611
1.7542
1.7492
1.7431
MeO
OMe
NH2
18
2.04
1.13
6.07
2.20
2.12
3.00
1.25
f1 (ppm)

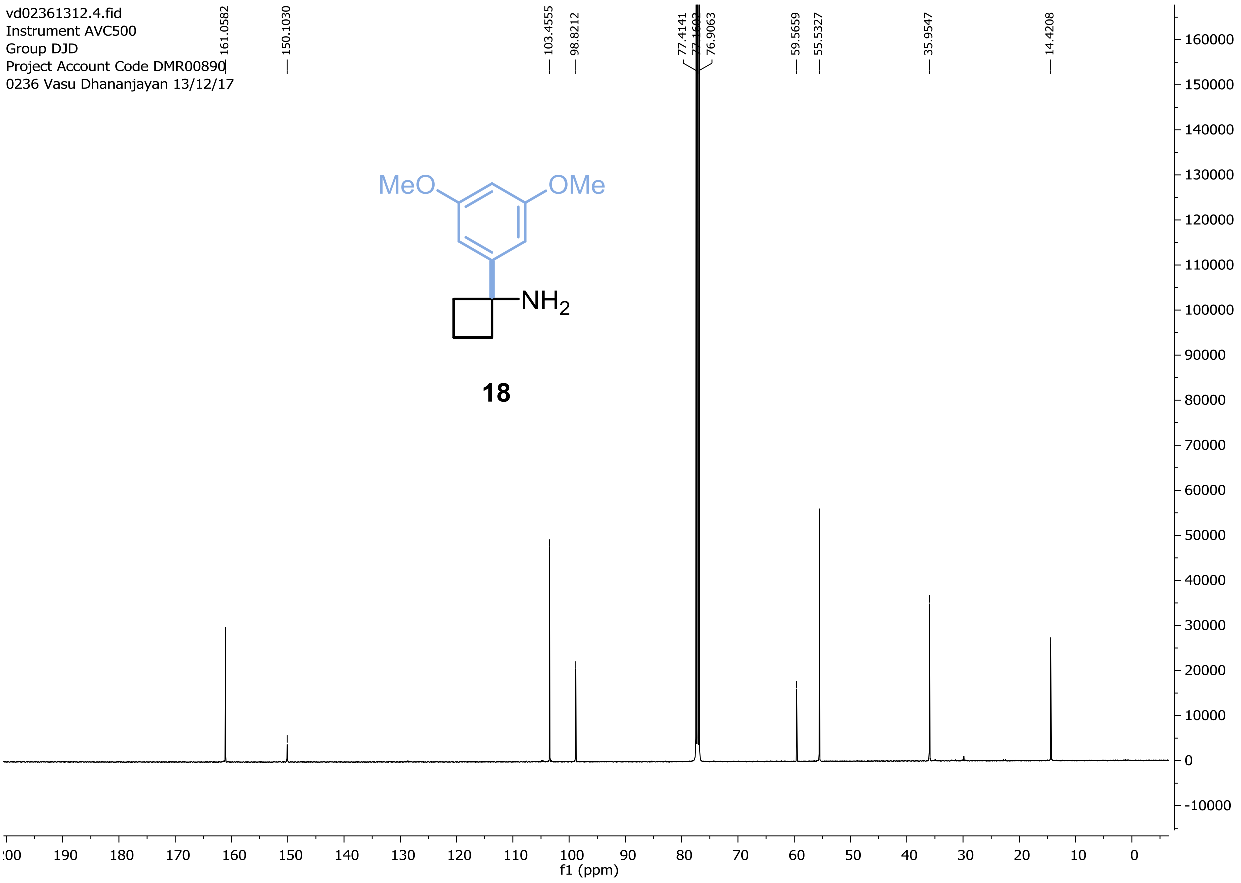
vd02361312.4.fid
Instrument AVC500
Group DJD
Project Account Code DMR00890
0236 Vasu Dhananjayan 13/12/17
161.0582
150.1030
103.4555
98.8212
77.4141
76.9063
59.5659
55.5327
35.9547
14.4208
MeO
OMe
NH2
18
f1 (ppm)

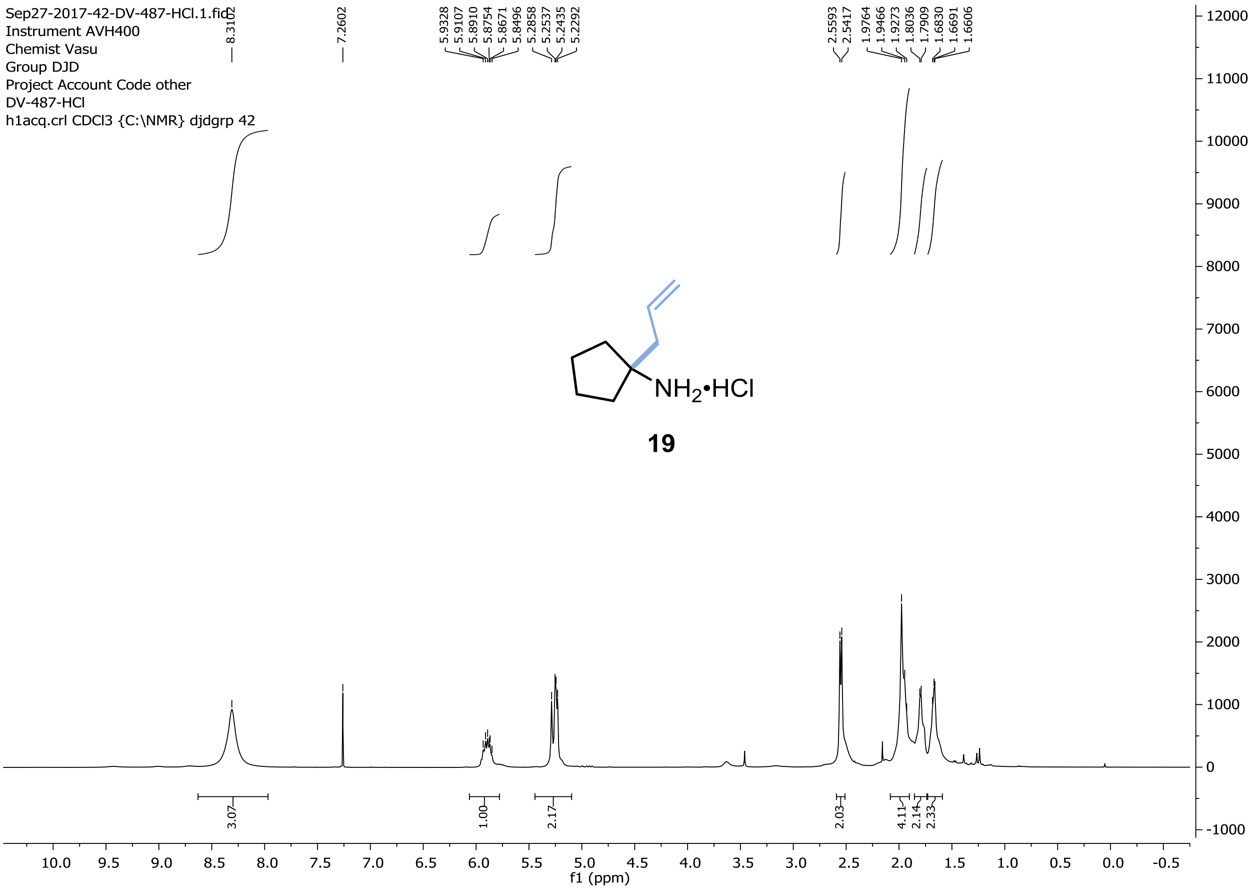
Sep27-2017-42-DV-487-HCl.1.fid
Instrument AVH400
Chemist Vasu
Group DJD
Project Account Code other
DV-487-HCl
h1acq.crl CDCl3 {C:\NMR} djdgrp 42
8.3102
7.2602
5.9328
5.9107
5.8910
5.8754
5.8671
5.8496
5.2858
5.2537
5.2435
5.2292
2.5593
2.5417
1.9764
1.9466
1.9273
1.8036
1.7909
1.6830
1.6691
1.6606
NH2•HCl
19
3.07
1.00
2.17
2.03
4.11
2.14
2.33
f1 (ppm)

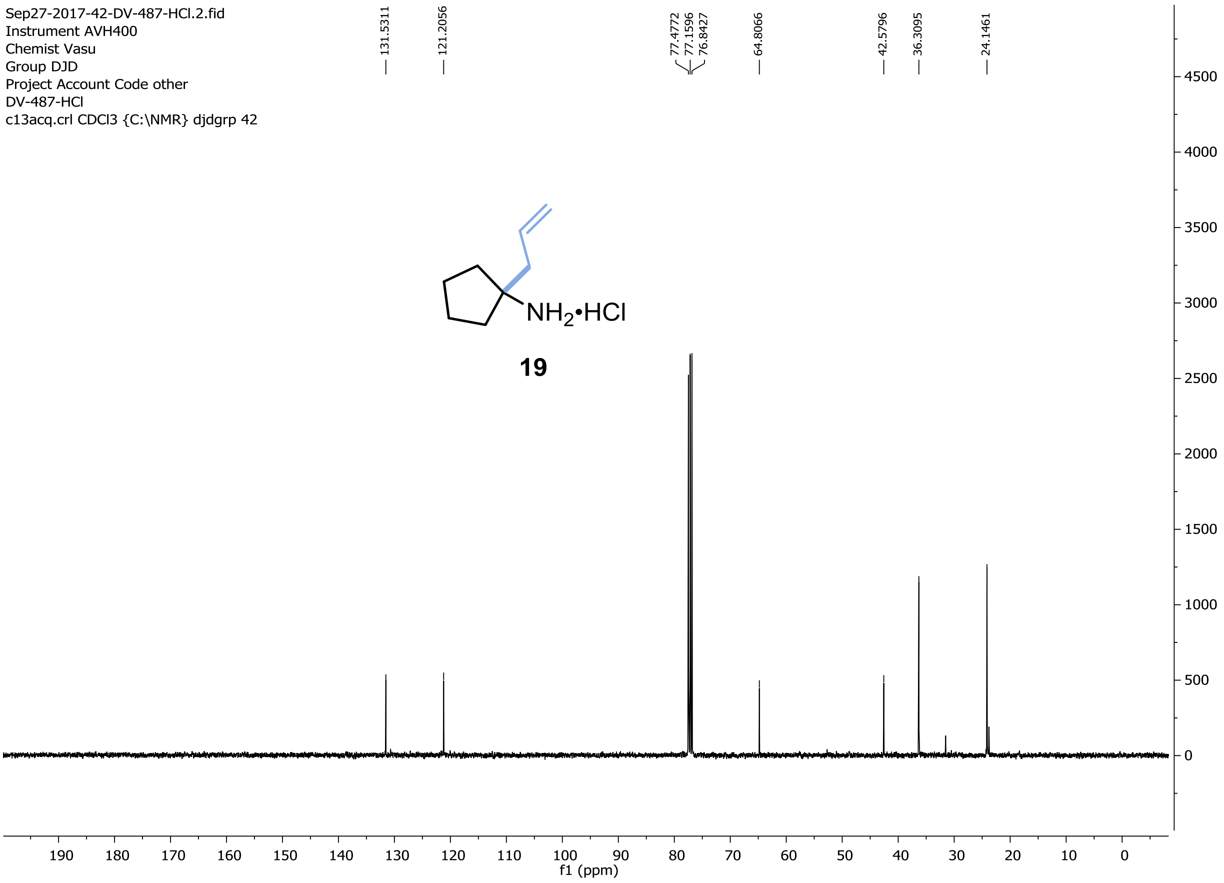
Sep27-2017-42-DV-487-HCl.2.fid
Instrument AVH400
Chemist Vasu
Group DJD
Project Account Code other
DV-487-HCl
c13acq.crl CDCl3 {C:\NMR} djdgrp 42
131.5311
121.2056
77.4772
77.1596
76.8427
64.8066
42.5796
36.3095
24.1461
NH2•HCl
19
f1 (ppm)

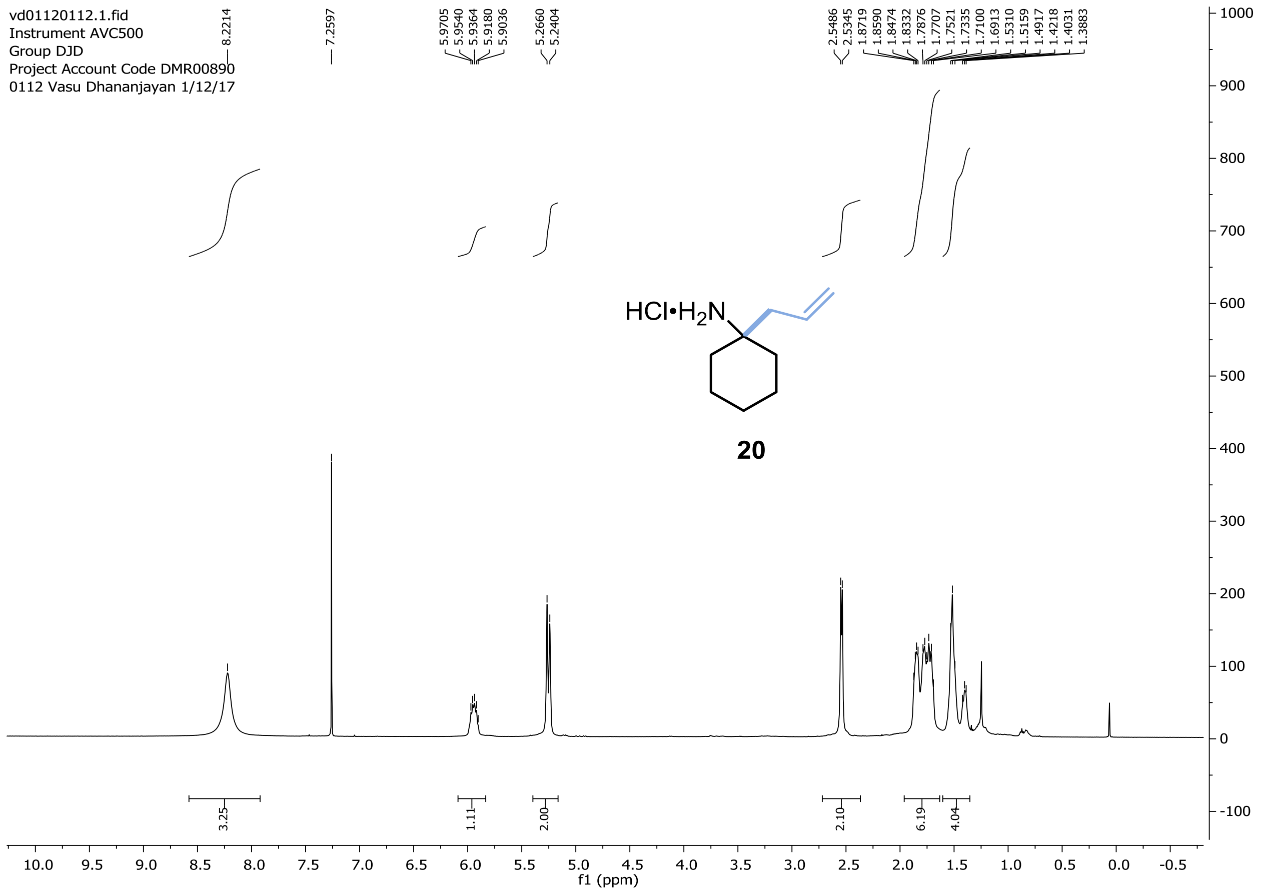

vd01120112.1.fid
Instrument AVC500
Group DJD
Project Account Code DMR00890
0112 Vasu Dhananjayan 1/12/17
8.2214
7.2597
5.9705
5.9540
5.9364
5.9180
5.9036
5.2660
5.2404
2.5486
2.5345
1.8719
1.8590
1.8474
1.8332
1.7876
1.7707
1.7521
1.7335
1.7100
1.6913
1.5310
1.5159
1.4917
1.4218
1.4031
1.3883
HCl•H2N
20
3.25
1.11
2.00
2.10
6.19
4.04
f1 (ppm)

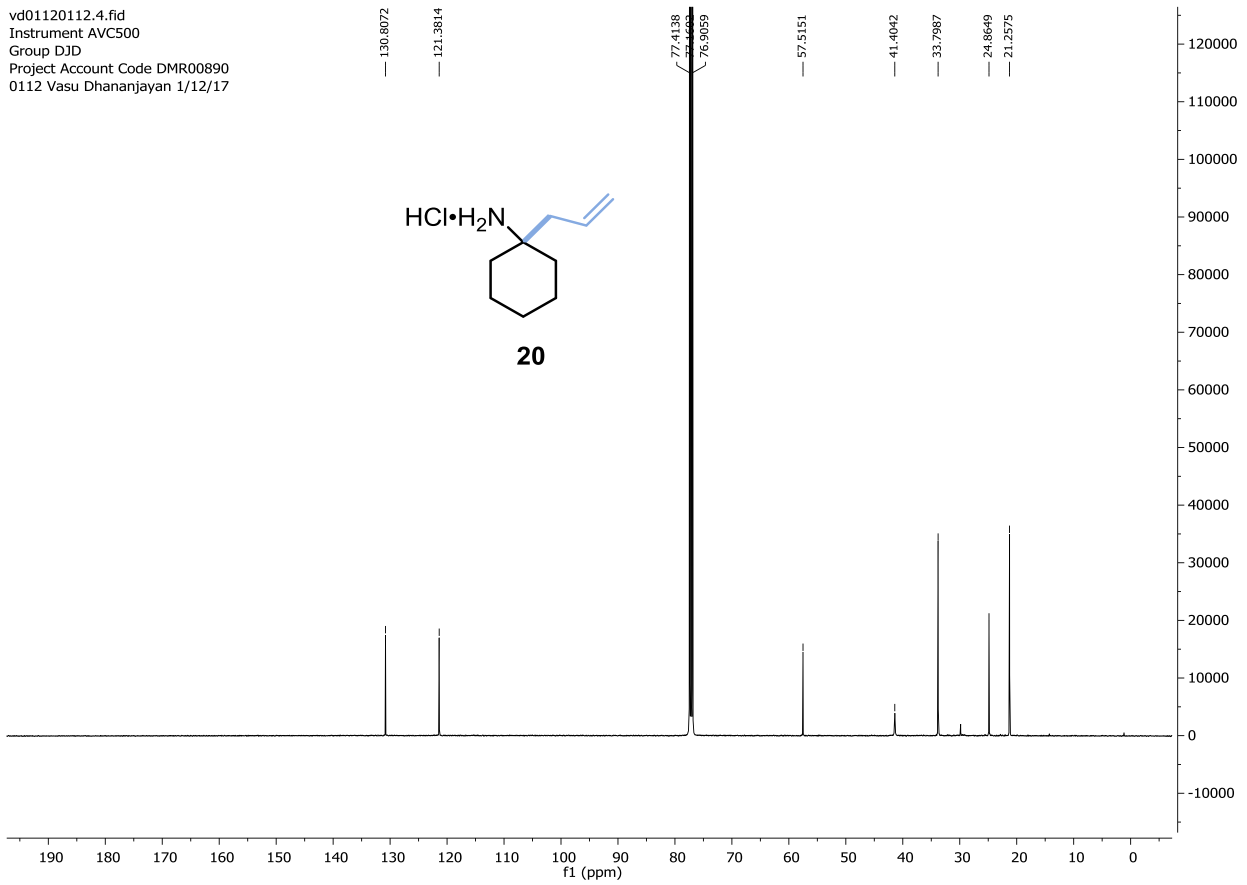

vd01120112.4.fid
Instrument AVC500
Group DJD
Project Account Code DMR00890
0112 Vasu Dhananjayan 1/12/17
130.8072
121.3814
77.4138
76.9059
57.5151
41.4042
33.7987
24.8649
21.2575
HCl•H2N
20
f1 (ppm)

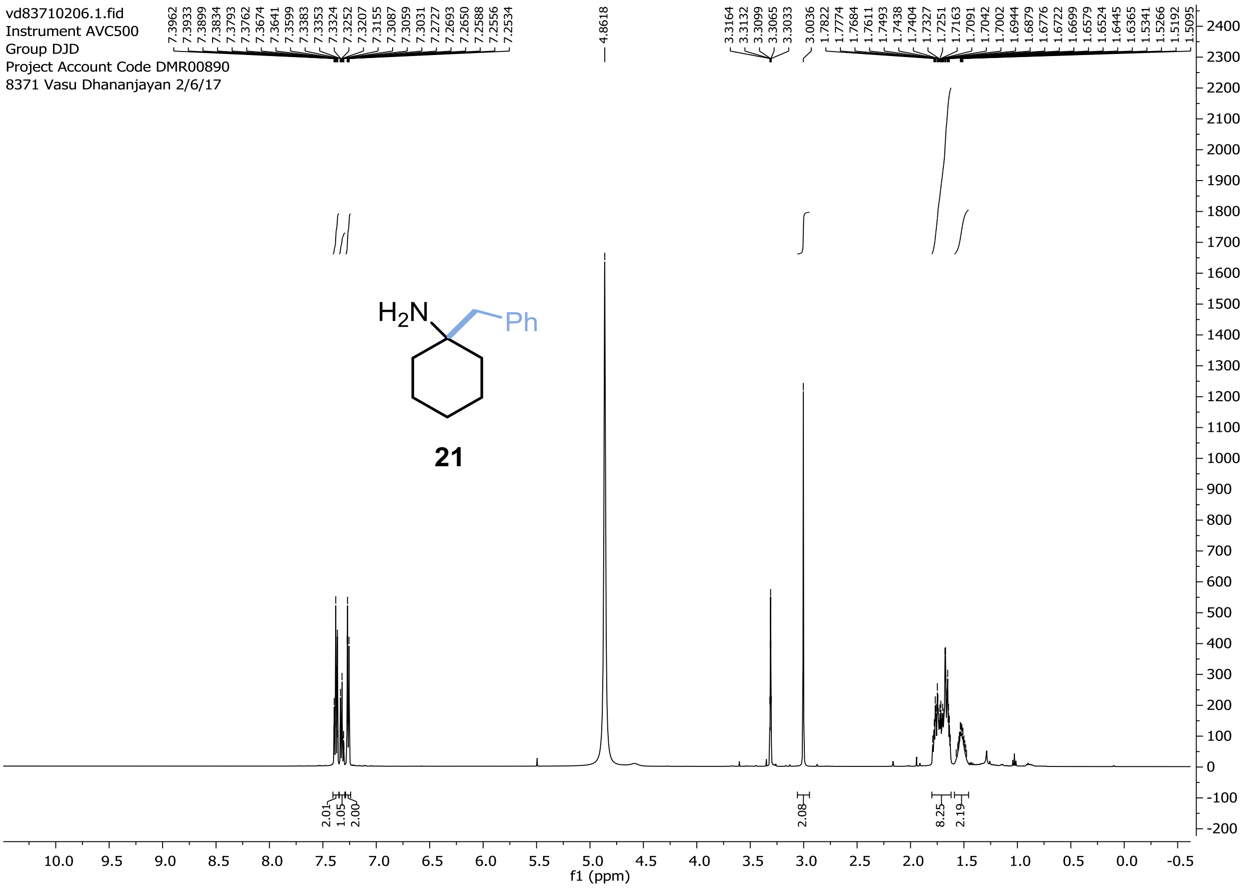
vd83710206.1.fid
Instrument AVC500
Group DJD
Project Account Code DMR00890
8371 Vasu Dhananjayan 2/6/17
7.3962 7.3933 7.3899 7.3834 7.3793 7.3762 7.3674 7.3641 7.3599 7.3383 7.3353 7.3324 7.3252 7.3207 7.3155 7.3087 7.3059 7.3031 7.2727 7.2693 7.2650 7.2588 7.2556 7.2534
4.8618
3.3164 3.3132 3.3099 3.3065 3.3033
3.0036
1.7822 1.7774 1.7684 1.7611 1.7493 1.7438 1.7404 1.7327 1.7251 1.7163 1.7091 1.7042 1.7002 1.6944 1.6879 1.6776 1.6722 1.6699 1.6579 1.6524 1.6445 1.6365 1.5341 1.5266 1.5192 1.5095
H2N
Ph
21
2.01 1.05 2.00
2.08
8.25 2.19
f1 (ppm)

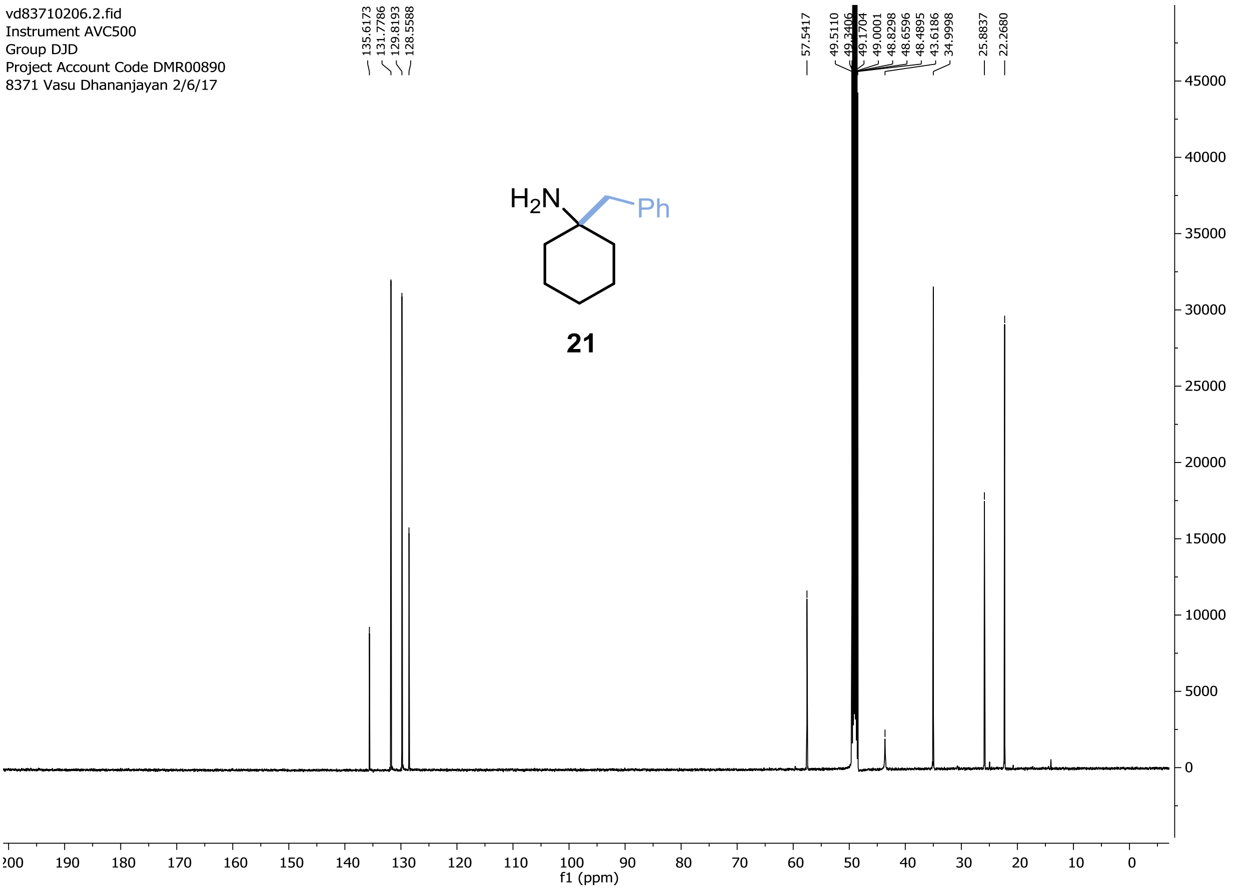
vd83710206.2.fid
Instrument AVC500
Group DJD
Project Account Code DMR00890
8371 Vasu Dhananjayan 2/6/17
135.6173 131.7786 129.8193 128.5588
57.5417
49.5110 49.3406 49.1704 49.0001 48.8298 48.6596 48.4895 43.6186 34.9998
25.8837 22.2680
H2N
Ph
21
f1 (ppm)

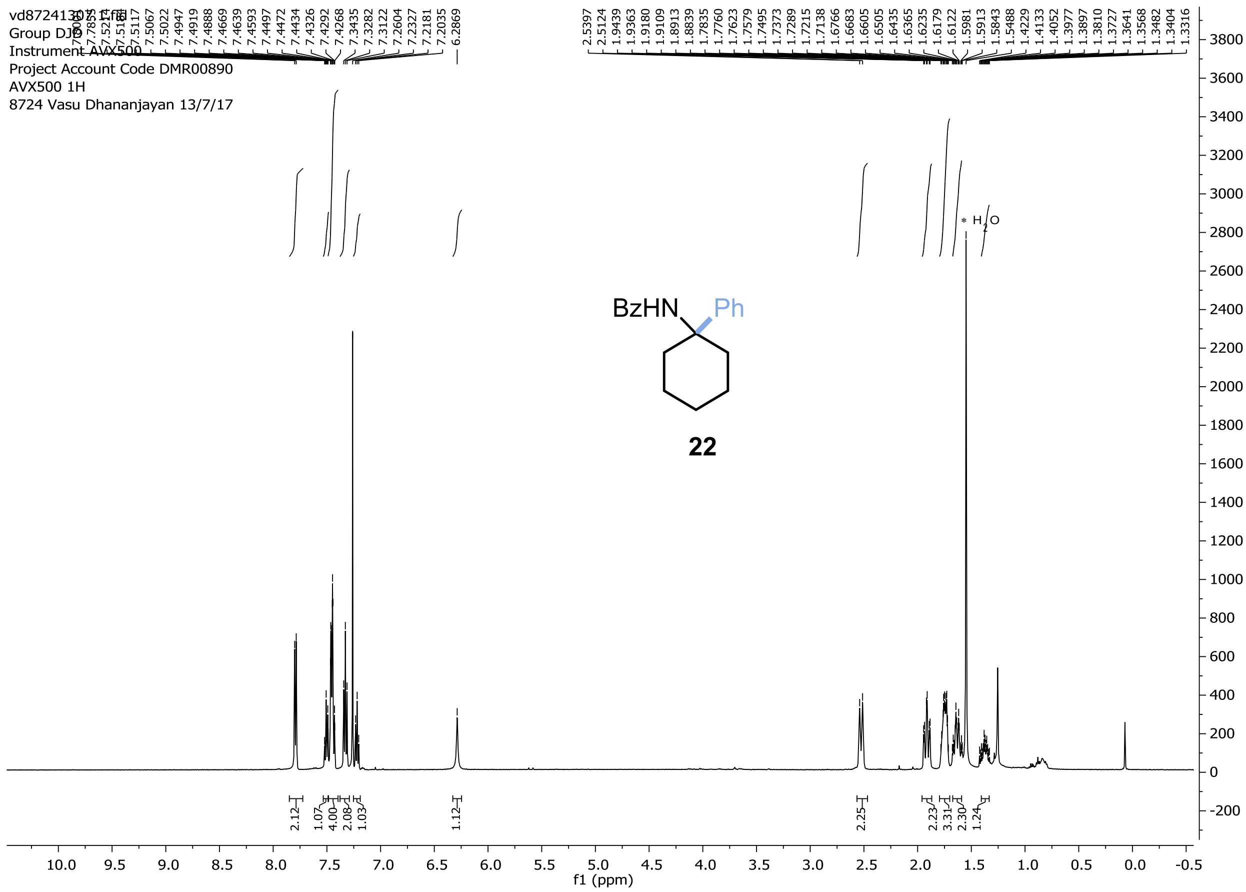
vd87241307.1.fid
Group DJD
Instrument AVX500
Project Account Code DMR00890
AVX500 1H
8724 Vasu Dhananjayan 13/7/17
7.5117 7.5067 7.5022 7.4947 7.4919 7.4888 7.4669 7.4639 7.4593 7.4497 7.4472 7.4434 7.4326 7.4292 7.4268 7.3435 7.3282 7.3122 7.2604 7.2327 7.2181 7.2035 6.2869
2.5397 2.5124 1.9439 1.9363 1.9180 1.9109 1.8913 1.8839 1.7835 1.7760 1.7623 1.7579 1.7495 1.7373 1.7289 1.7215 1.7138 1.6766 1.6683 1.6605 1.6505 1.6435 1.6365 1.6235 1.6179 1.6122 1.5981 1.5913 1.5843 1.5488 1.4229 1.4133 1.4052 1.3977 1.3897 1.3810 1.3727 1.3641 1.3568 1.3482 1.3404 1.3316
* H2O
BzHN
Ph
22
2.12 1.07 4.00 2.08 1.03 1.12 2.25 2.23 3.31 2.30 1.24
f1 (ppm)

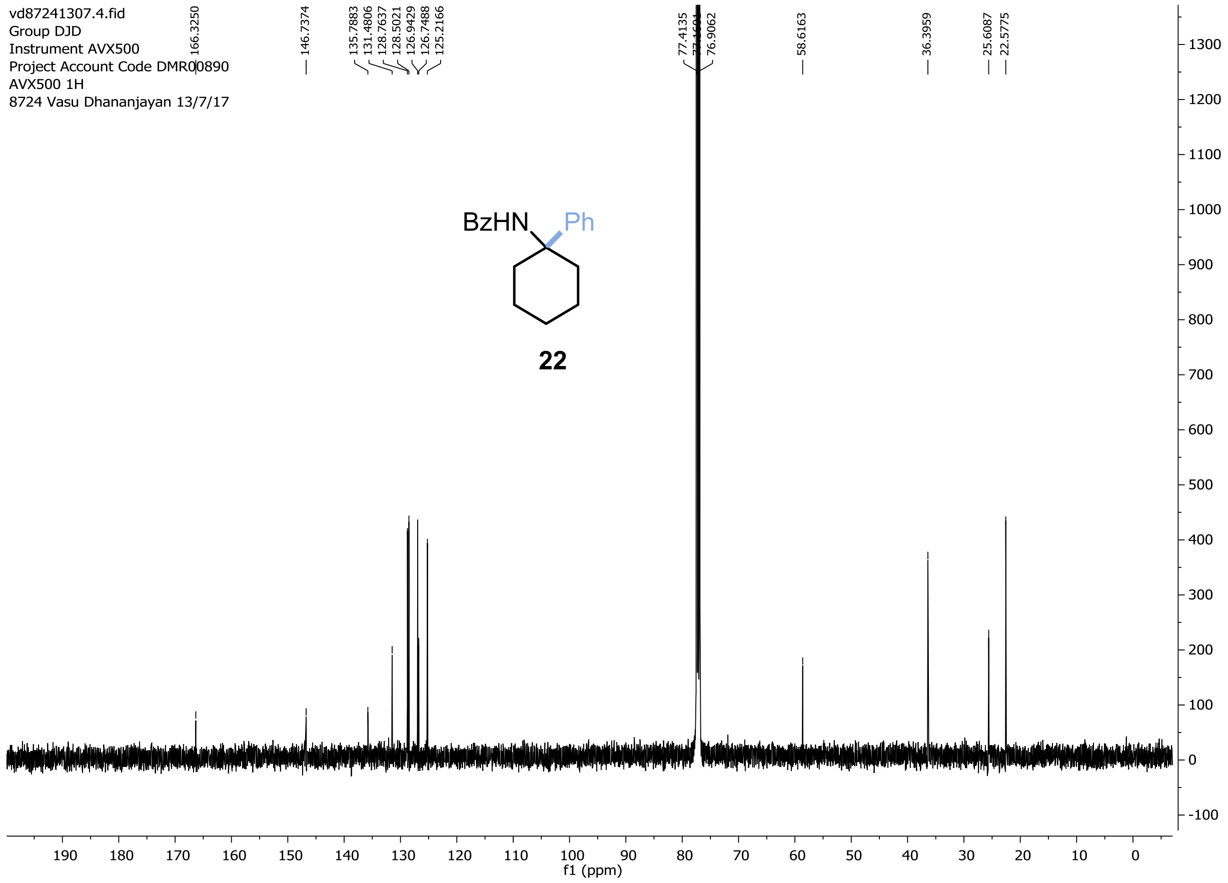
vd87241307.4.fid
Group DJD
Instrument AVX500
Project Account Code DMR00890
AVX500 1H
8724 Vasu Dhananjayan 13/7/17
166.3250
146.7374
135.7883 131.4806 128.7637 128.5021 126.9429 126.7488 125.2166
77.4135 76.9062
58.6163
36.3959
25.6087 22.5775
BzHN
Ph
22
f1 (ppm)

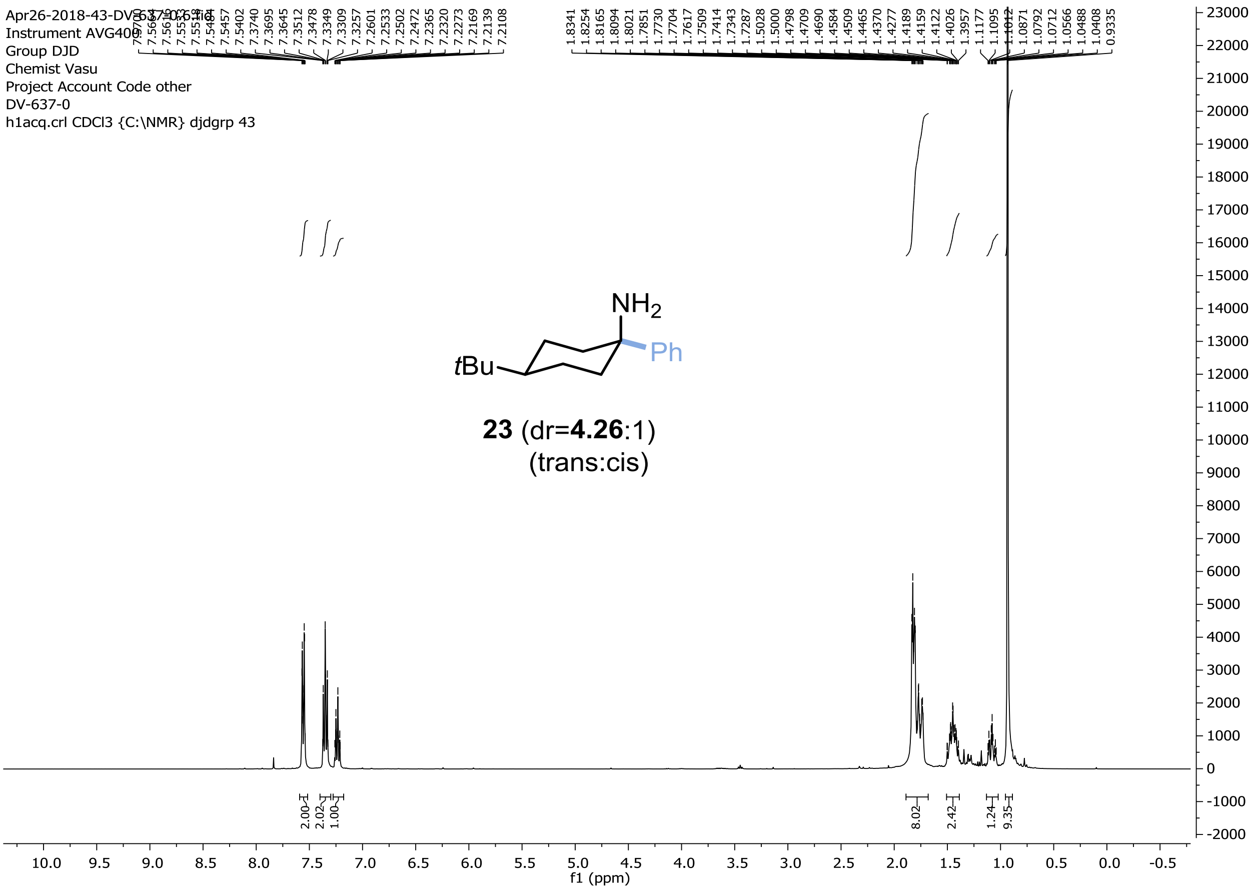
Group DJD
Chemist Vasu
Project Account Code other
DV-637-0
h1acq.crl CDCl3 {C:\NMR} djdgrp 43
NH2
tBu
Ph
23 (dr=4.26:1)
(trans:cis)
f1 (ppm)

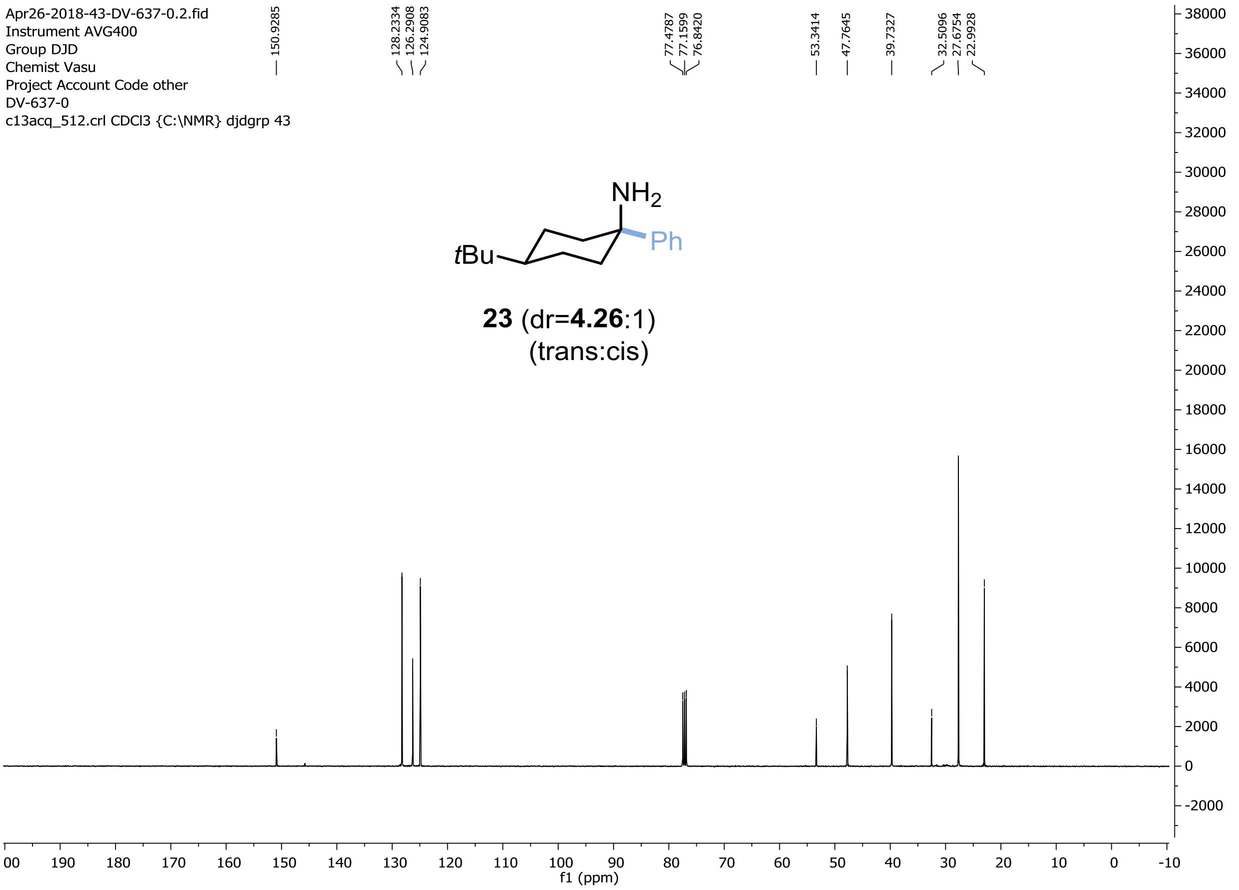
Apr26-2018-43-DV-637-0.2.fid
Instrument AVG400
Group DJD
Chemist Vasu
Project Account Code other
DV-637-0
c13acq_512.crl CDCl3 {C:\NMR} djdgrp 43
150.9285
128.2334
126.2908
124.9083
77.4787
77.1599
76.8420
53.3414
47.7645
39.7327
32.5096
27.6754
22.9928
NH2
tBu
Ph
23 (dr=4.26:1)
(trans:cis)
f1 (ppm)

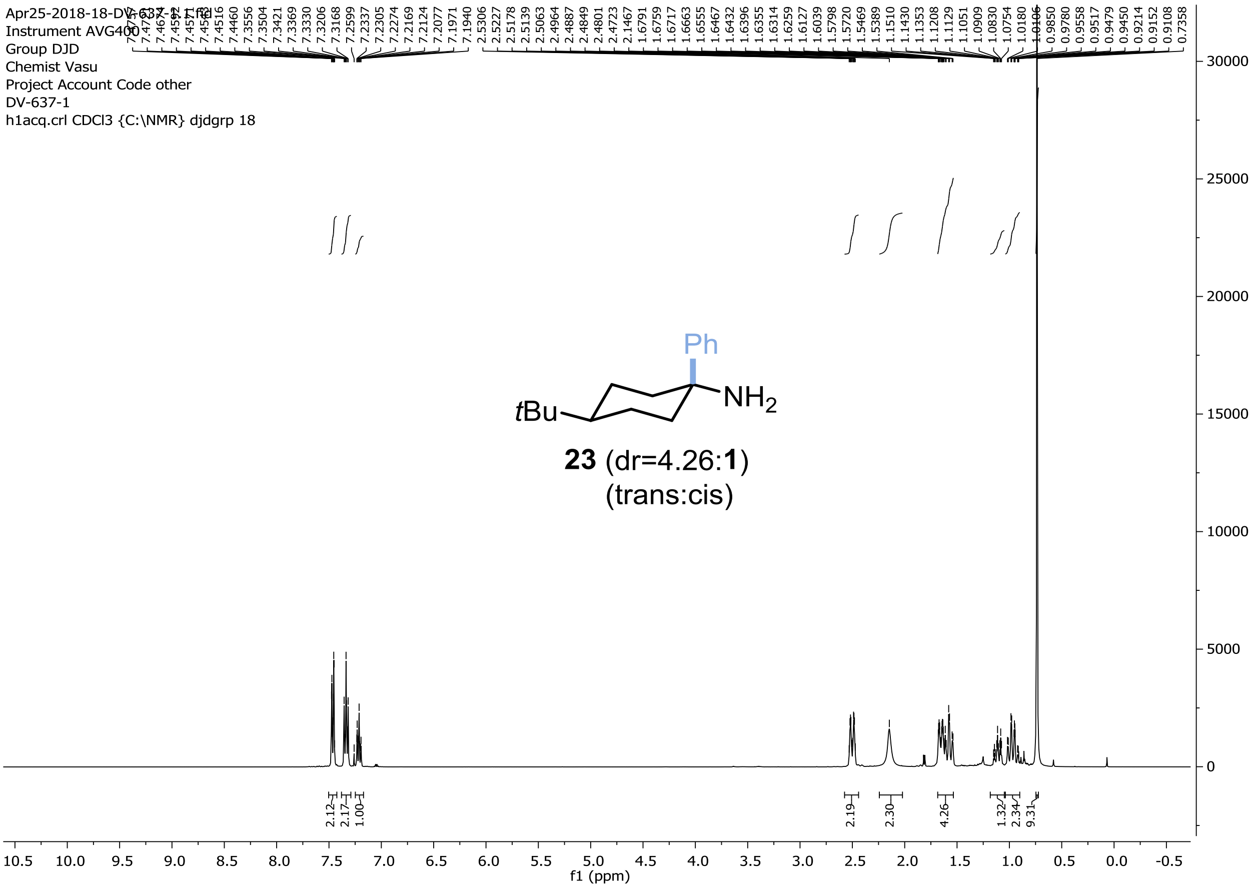
Apr25-2018-18-DV-637-1.1.fid
Instrument AVG400
Group DJD
Chemist Vasu
Project Account Code other
DV-637-1
h1acq.crl CDCl3 {C:\NMR} djdgrp 18
Ph
tBu
NH2
23 (dr=4.26:1)
(trans:cis)
2.12
2.17
1.00
2.19
2.30
4.26
1.32
2.34
9.31
f1 (ppm)

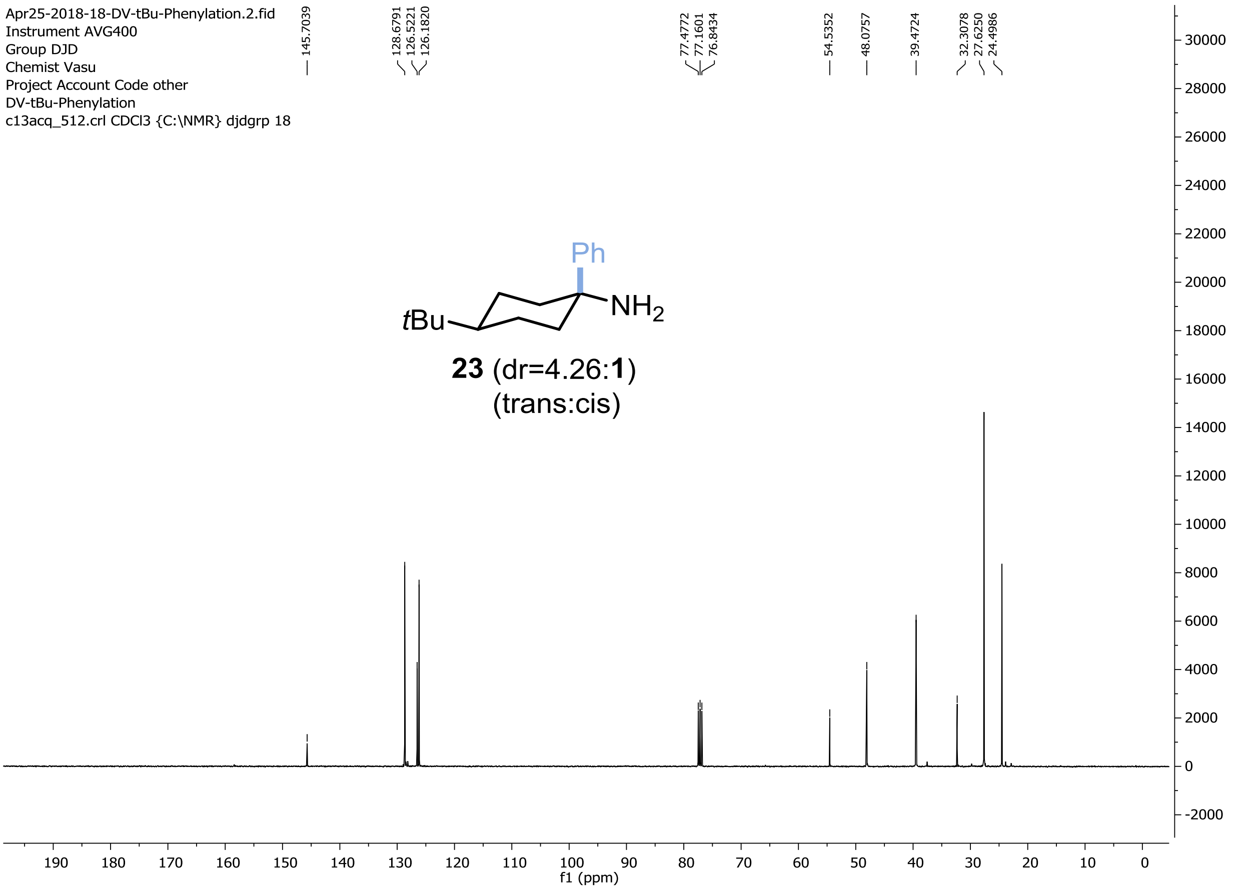
Apr25-2018-18-DV-tBu-Phenylation.2.fid
Instrument AVG400
Group DJD
Chemist Vasu
Project Account Code other
DV-tBu-Phenylation
c13acq_512.crl CDCl3 {C:\NMR} djdgrp 18
145.7039
128.6791
126.5221
126.1820
77.4772
77.1601
76.8434
54.5352
48.0757
39.4724
32.3078
27.6250
24.4986
Ph
tBu
NH2
23 (dr=4.26:1)
(trans:cis)
f1 (ppm)

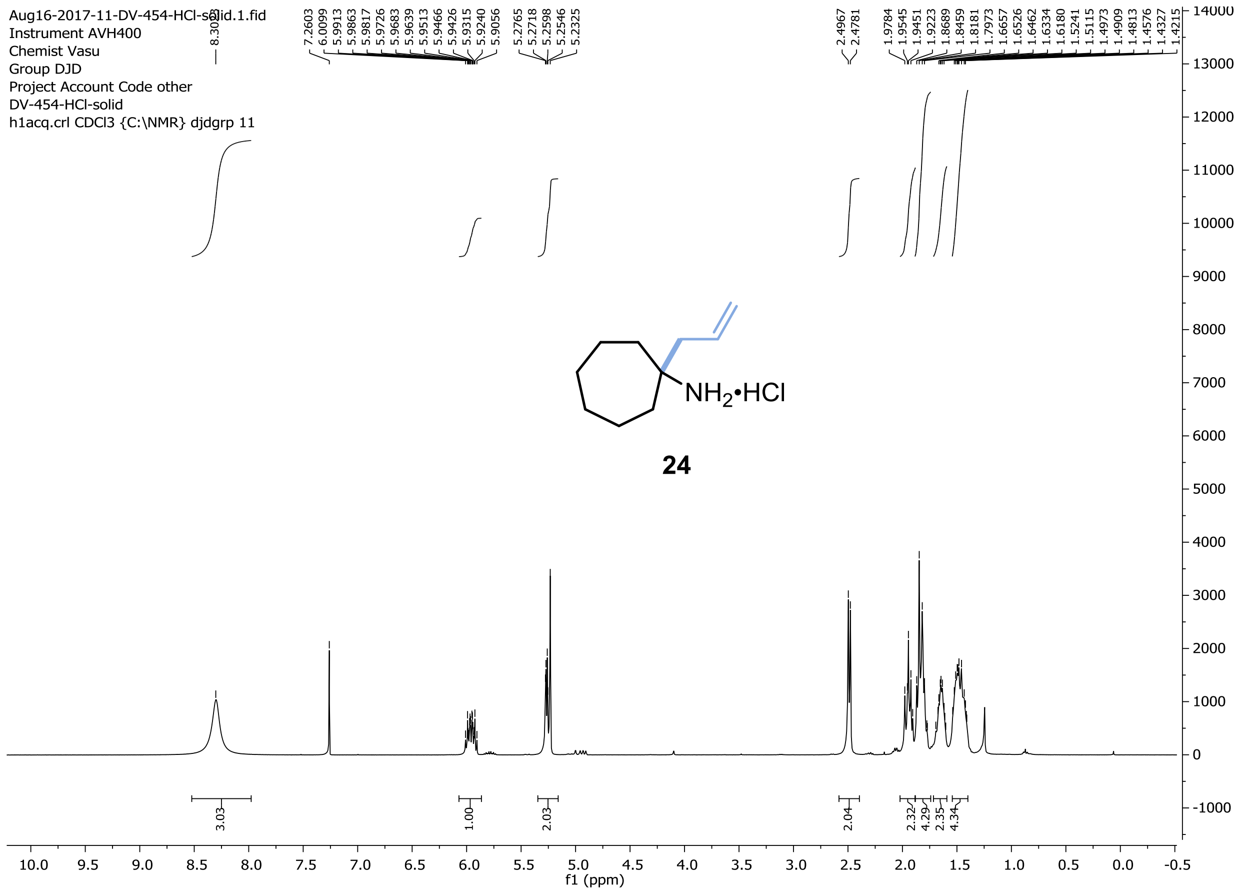
Aug16-2017-11-DV-454-HCl-solid.1.fid
Instrument AVH400
Chemist Vasu
Group DJD
Project Account Code other
DV-454-HCl-solid
h1acq.crl CDCl3 {C:\NMR} djdgrp 11
NH2•HCl
24
f1 (ppm)

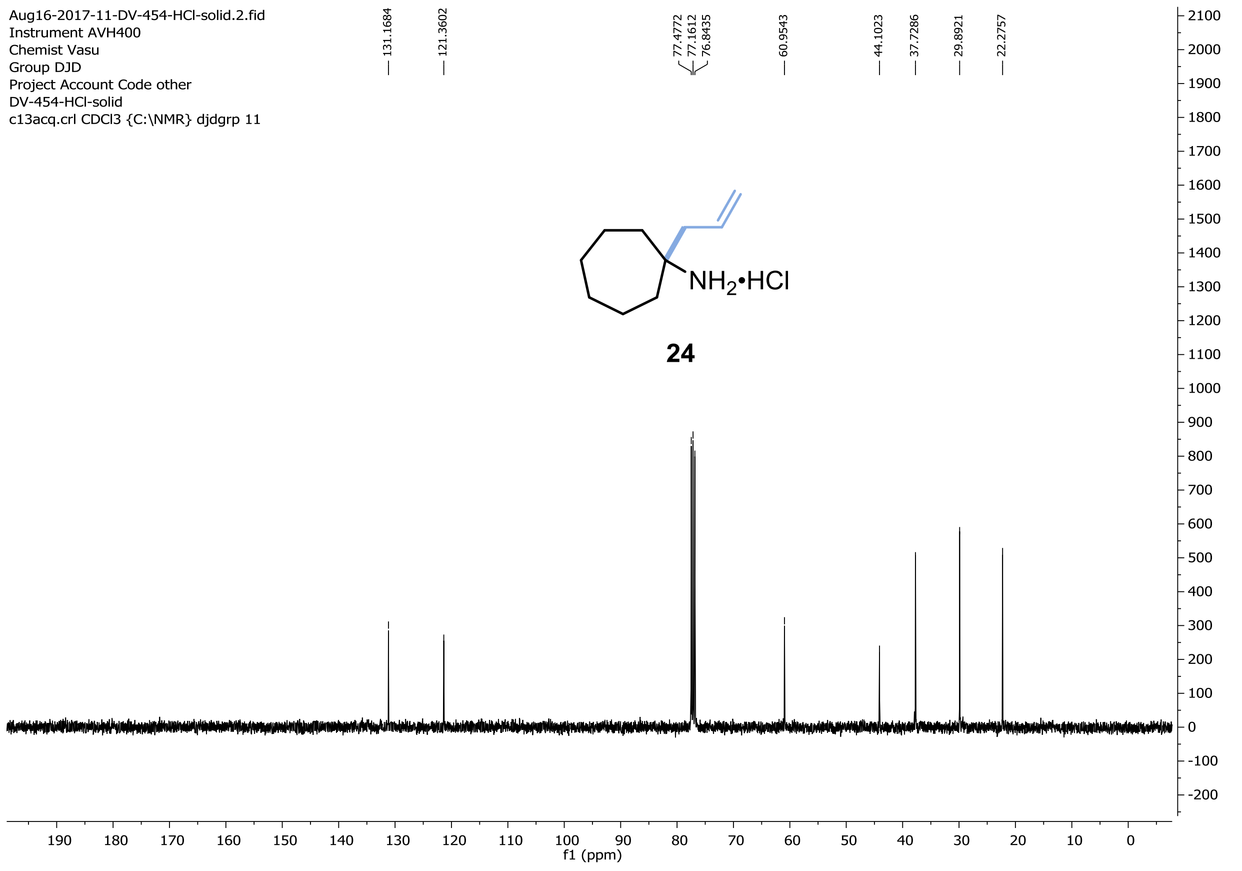
Aug16-2017-11-DV-454-HCl-solid.2.fid
Instrument AVH400
Chemist Vasu
Group DJD
Project Account Code other
DV-454-HCl-solid
c13acq.crl CDCl3 {C:\NMR} djdgrp 11
131.1684
121.3602
77.4772
77.1612
76.8435
60.9543
44.1023
37.7286
29.8921
22.2757
NH2•HCl
24
f1 (ppm)

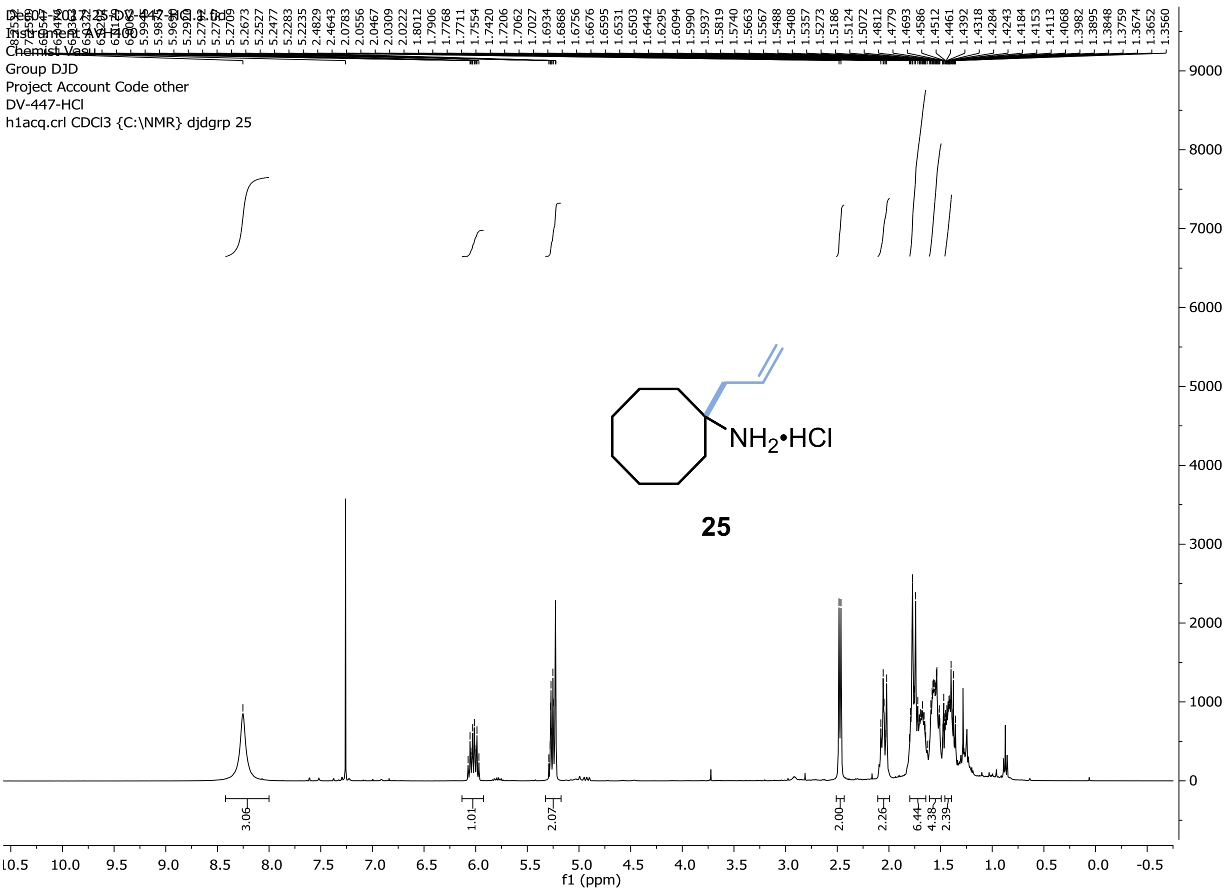
Instrument AVH400
Chemist Vasu
Group DJD
Project Account Code other
DV-447-HCl
h1acq.crl CDCl3 {C:\NMR} djdgrp 25
5.2709 5.2673 5.2527 5.2477 5.2283 5.2235 2.4829 2.4643 2.0783 2.0556 2.0467 2.0309 2.0222 1.8012 1.7906 1.7768 1.7711 1.7554 1.7420 1.7206 1.7062 1.7027 1.6934 1.6868 1.6756 1.6676 1.6595 1.6531 1.6503 1.6442 1.6295 1.6094 1.5990 1.5937 1.5819 1.5740 1.5663 1.5567 1.5488 1.5408 1.5357 1.5273 1.5186 1.5124 1.5072 1.4812 1.4779 1.4693 1.4586 1.4512 1.4461 1.4392 1.4318 1.4284 1.4243 1.4184 1.4153 1.4113 1.4068 1.3982 1.3895 1.3848 1.3759 1.3674 1.3652 1.3560
NH2•HCl
25
3.06
1.01
2.07
2.00
2.26
6.44
4.38
2.39
f1 (ppm)

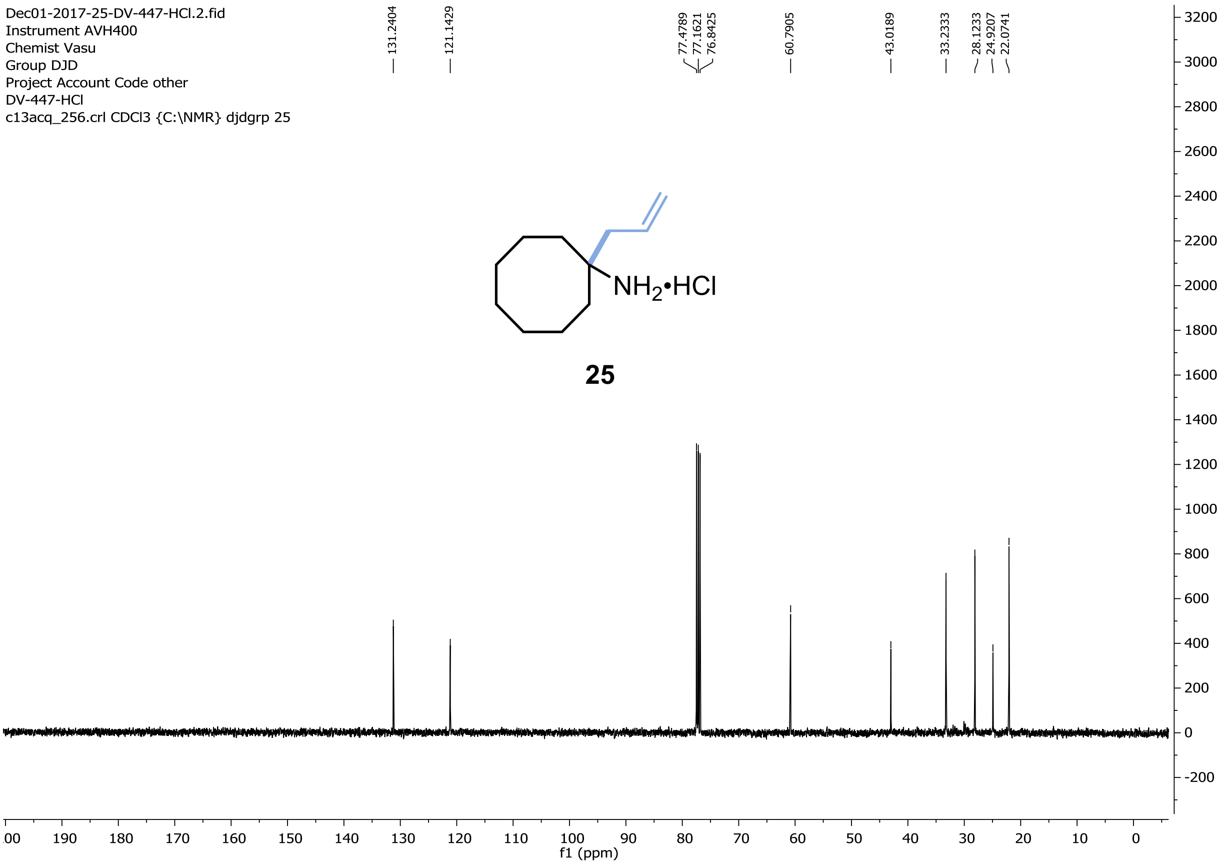
Dec01-2017-25-DV-447-HCl.2.fid
Instrument AVH400
Chemist Vasu
Group DJD
Project Account Code other
DV-447-HCl
c13acq_256.crl CDCl3 {C:\NMR} djdgrp 25
131.2404
121.1429
77.4789
77.1621
76.8425
60.7905
43.0189
33.2333
28.1233
24.9207
22.0741
NH2•HCl
25
f1 (ppm)

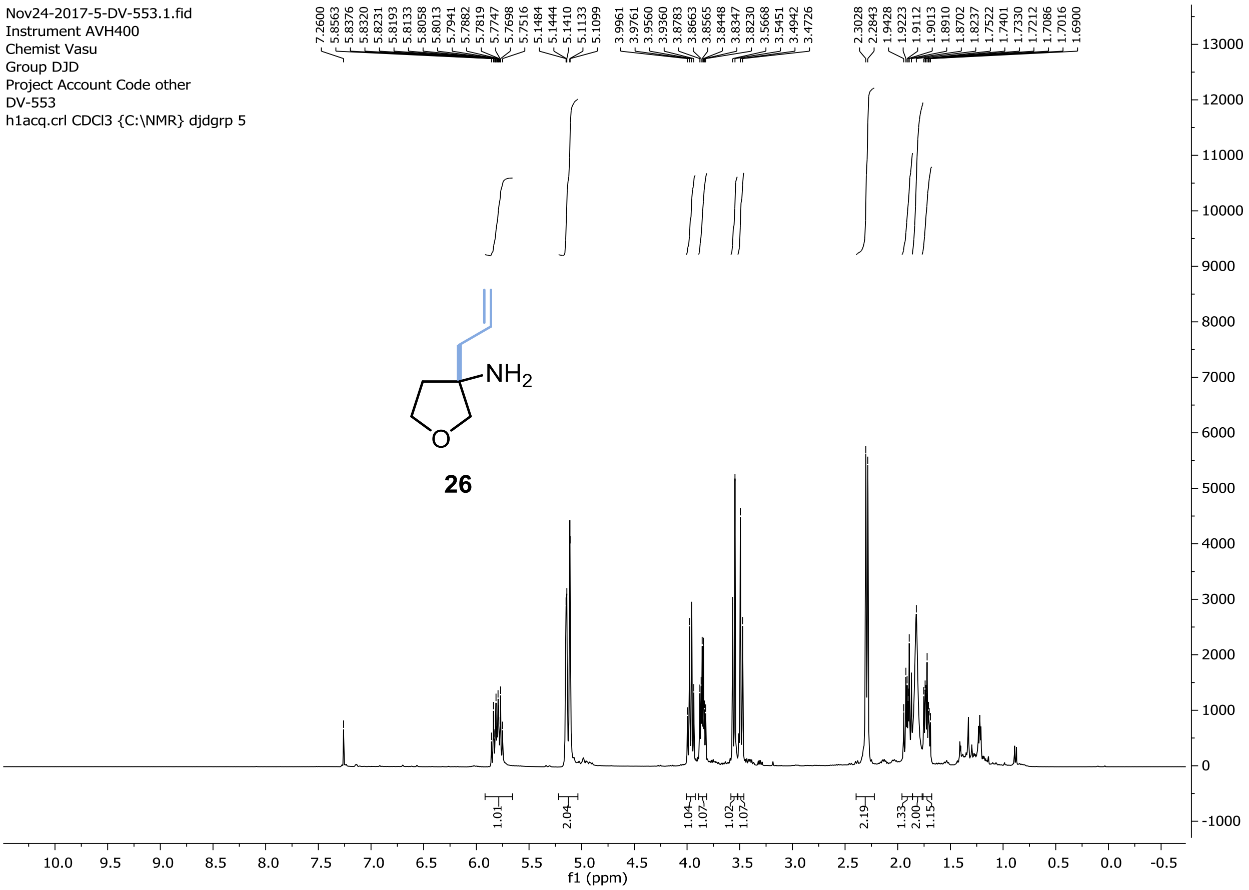
Nov24-2017-5-DV-553.1.fid
Instrument AVH400
Chemist Vasu
Group DJD
Project Account Code other
DV-553
h1acq.crl CDCl3 {C:\NMR} djdgrp 5
NH2
O
26
f1 (ppm)

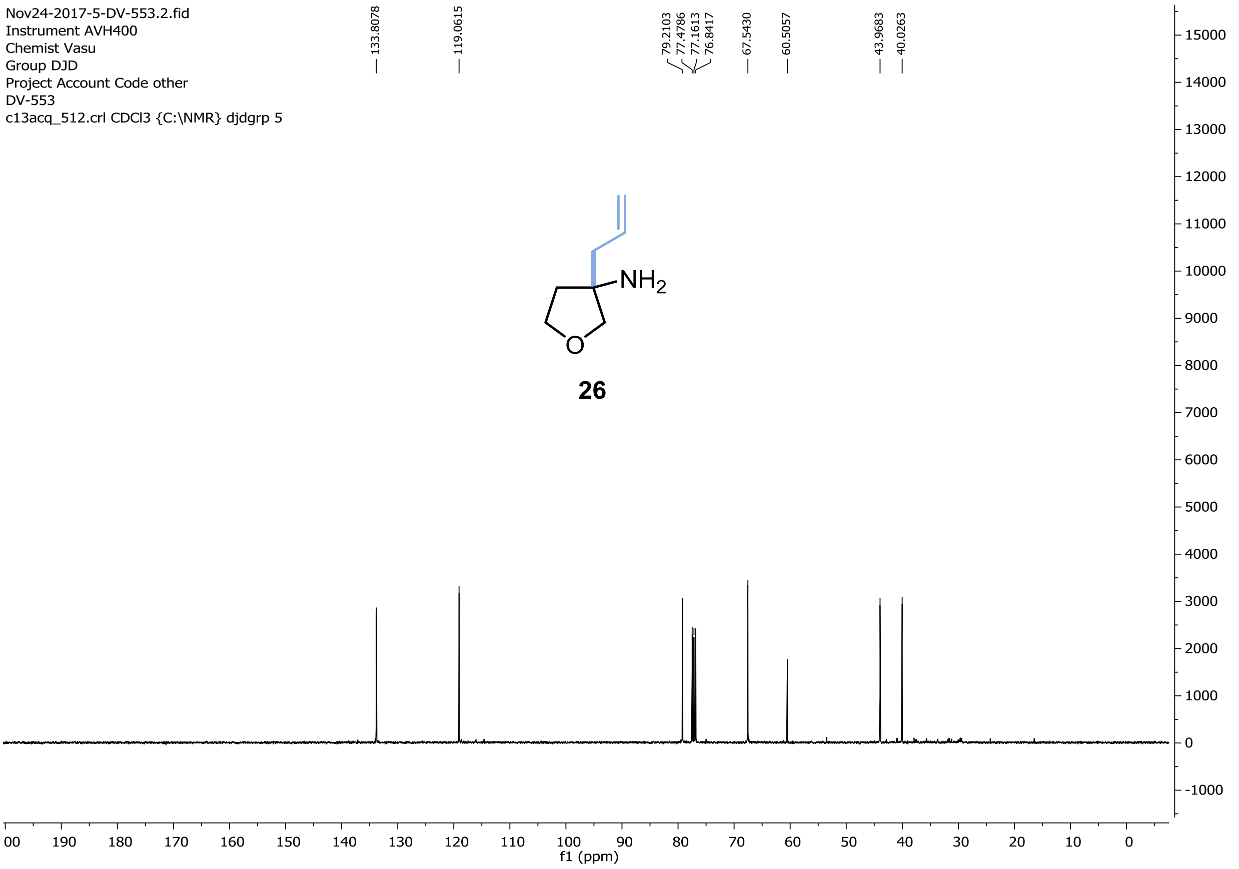
Nov24-2017-5-DV-553.2.fid
Instrument AVH400
Chemist Vasu
Group DJD
Project Account Code other
DV-553
c13acq_512.crl CDCl3 {C:\NMR} djdgrp 5
133.8078
119.0615
79.2103
77.4786
77.1613
76.8417
67.5430
60.5057
43.9683
40.0263
NH2
O
26
f1 (ppm)

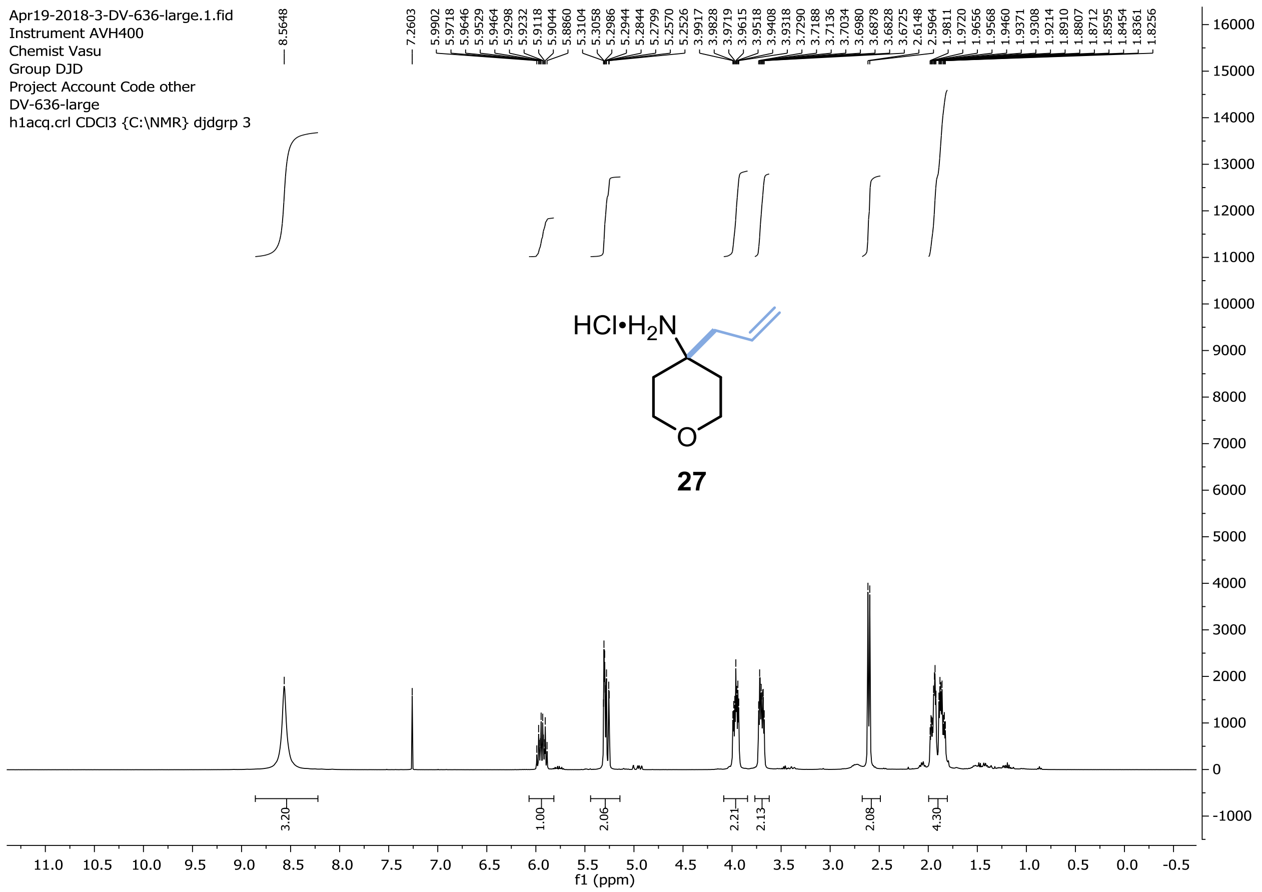
Apr19-2018-3-DV-636-large.1.fid
Instrument AVH400
Chemist Vasu
Group DJD
Project Account Code other
DV-636-large
h1acq.crl CDCl3 {C:\NMR} djdgrp 3
8.5648
7.2603
5.9902 5.9718 5.9646 5.9529 5.9464 5.9298 5.9232 5.9118 5.9044 5.8860 5.3104 5.3058 5.2986 5.2944 5.2844 5.2799 5.2570 5.2526 3.9917 3.9828 3.9719 3.9615 3.9518 3.9408 3.9318 3.7290 3.7188 3.7136 3.7034 3.6980 3.6878 3.6828 3.6725 2.6148 2.5964 1.9811 1.9720 1.9656 1.9568 1.9460 1.9371 1.9308 1.9214 1.8910 1.8807 1.8712 1.8595 1.8454 1.8361 1.8256
HCl•H2N
O
27
3.20
1.00
2.06
2.21
2.13
2.08
4.30
f1 (ppm)

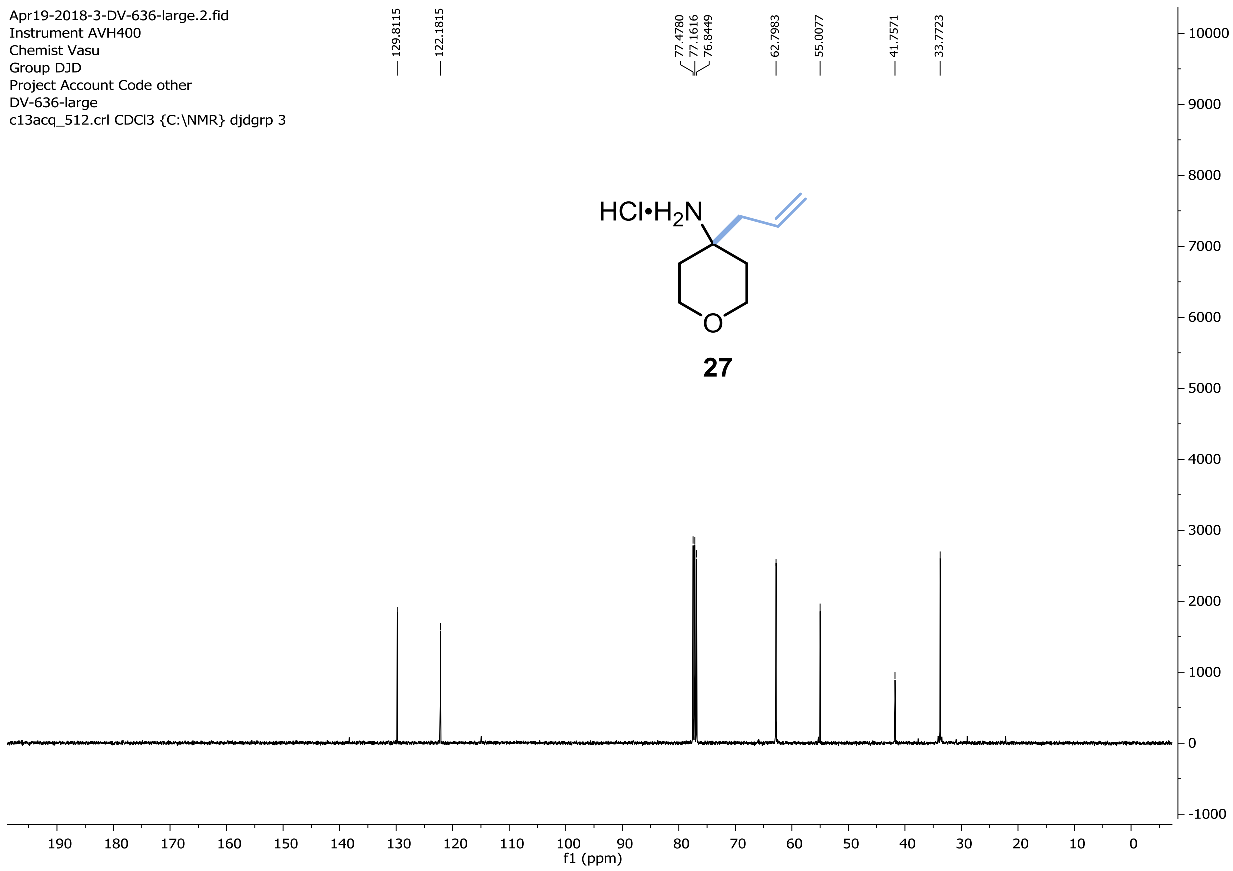
Apr19-2018-3-DV-636-large.2.fid
Instrument AVH400
Chemist Vasu
Group DJD
Project Account Code other
DV-636-large
c13acq_512.crl CDCl3 {C:\NMR} djdgrp 3
129.8115
122.1815
77.4780
77.1616
76.8449
62.7983
55.0077
41.7571
33.7723
HCl•H2N
O
27
f1 (ppm)

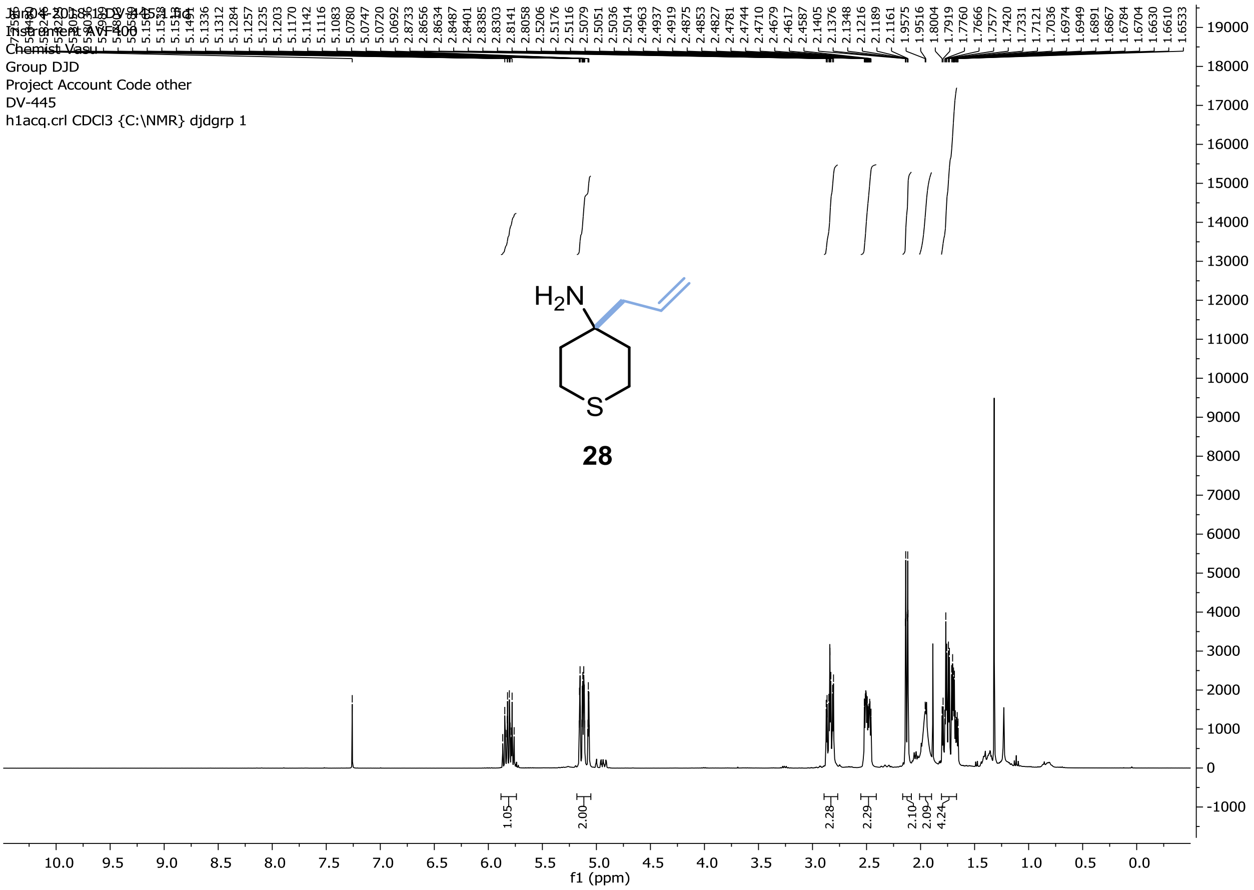
Group DJD
Project Account Code other
DV-445
h1acq.crl CDCl3 {C:\NMR} djdgrp 1
5.1336 5.1312 5.1284 5.1257 5.1235 5.1203 5.1170 5.1142 5.1116 5.1083 5.0780 5.0747 5.0720 5.0692 2.8733 2.8656 2.8634 2.8487 2.8401 2.8385 2.8303 2.8141 2.8058 2.5206 2.5176 2.5116 2.5079 2.5051 2.5036 2.5014 2.4963 2.4937 2.4919 2.4875 2.4853 2.4827 2.4781 2.4744 2.4710 2.4679 2.4617 2.4587 2.1405 2.1376 2.1348 2.1216 2.1189 2.1161 1.9575 1.9516 1.8004 1.7919 1.7760 1.7666 1.7577 1.7420 1.7331 1.7121 1.7036 1.6974 1.6949 1.6891 1.6867 1.6784 1.6704 1.6630 1.6610 1.6533
H2N
S
28
1.05 2.00 2.28 2.29 2.10 2.09 4.24
f1 (ppm)

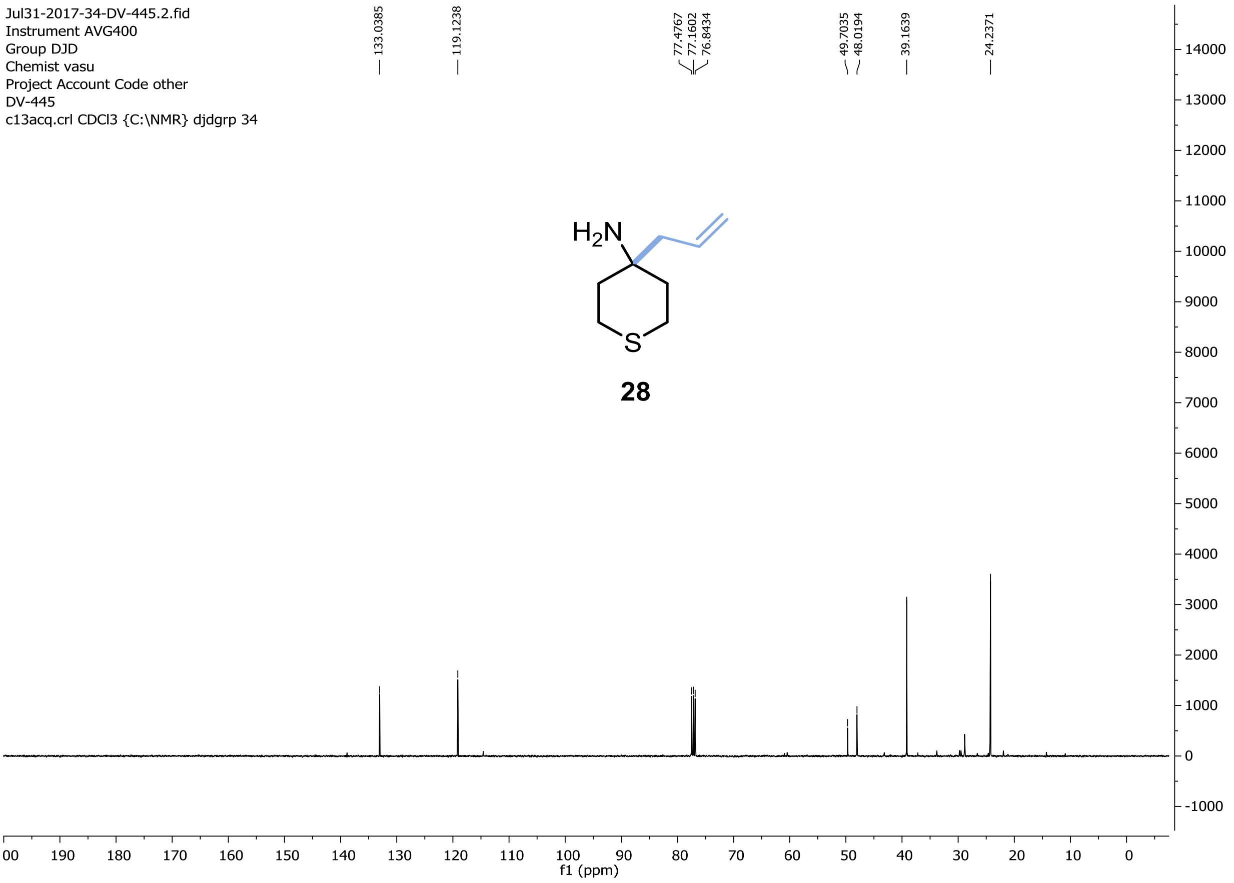
Jul31-2017-34-DV-445.2.fid
Instrument AVG400
Group DJD
Chemist vasu
Project Account Code other
DV-445
c13acq.crl CDCl3 {C:\NMR} djdgrp 34
133.0385
119.1238
77.4767
77.1602
76.8434
49.7035
48.0194
39.1639
24.2371
H2N
S
28
f1 (ppm)

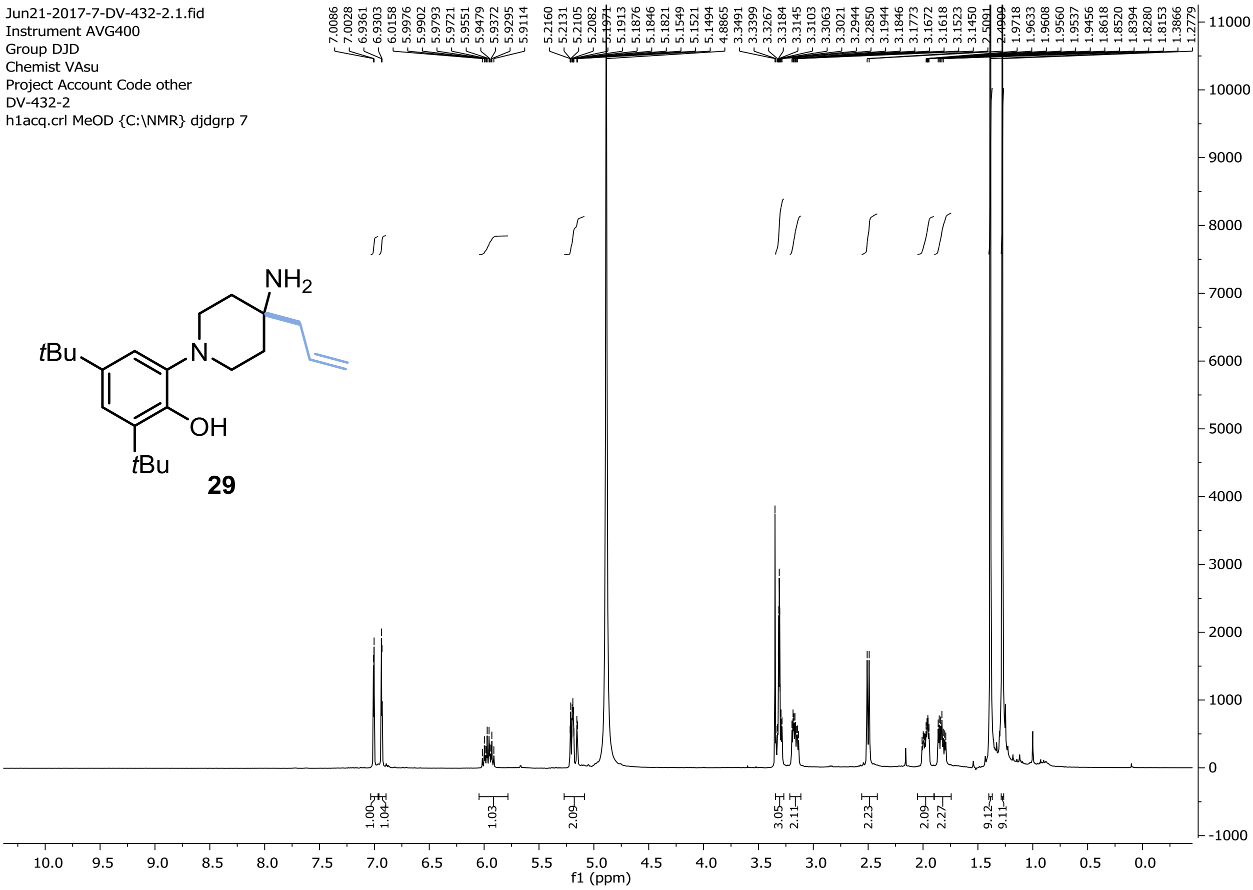

Jun21-2017-7-DV-432-2.1.fid
Instrument AVG400
Group DJD
Chemist VAsu
Project Account Code other
DV-432-2
h1acq.crl MeOD {C:\NMR} djdgrp 7
NH2
tBu
N
OH
tBu
29
f1 (ppm)

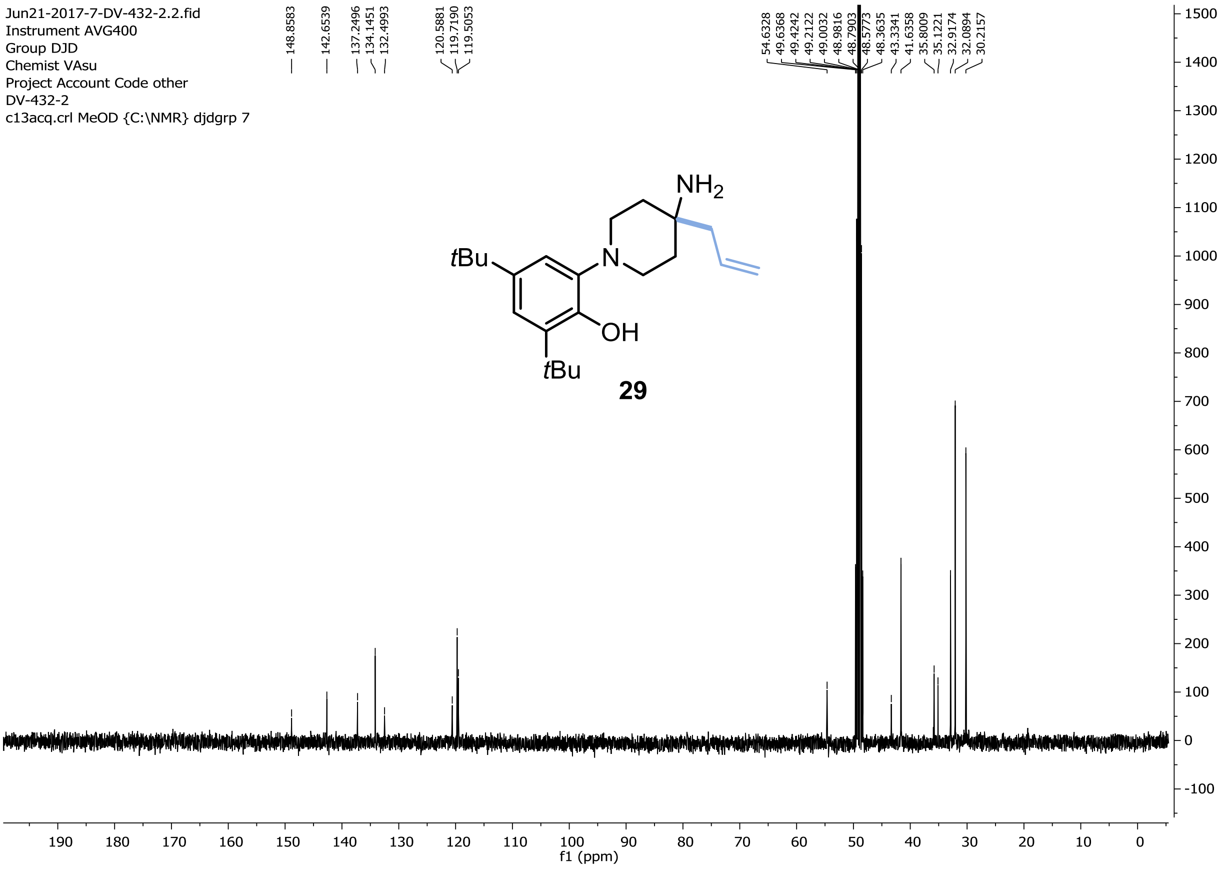

Jun21-2017-7-DV-432-2.2.fid
Instrument AVG400
Group DJD
Chemist VAsu
Project Account Code other
DV-432-2
c13acq.crl MeOD {C:\NMR} djdgrp 7
NH2
tBu
N
OH
tBu
29
f1 (ppm)

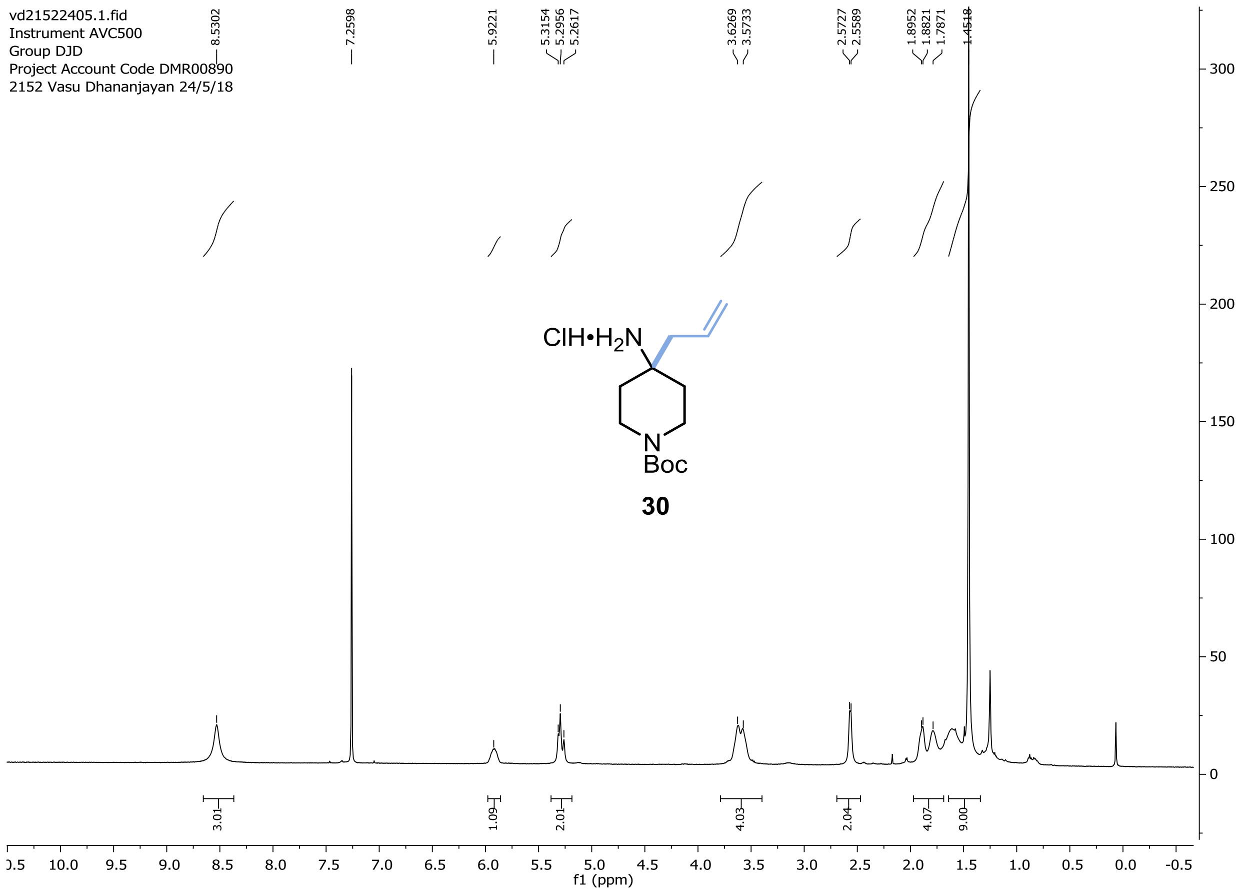
vd21522405.1.fid
Instrument AVC500
Group DJD
Project Account Code DMR00890
2152 Vasu Dhananjayan 24/5/18
8.5302
7.2598
5.9221
5.3154
5.2956
5.2617
3.6269
3.5733
2.5727
2.5589
1.8952
1.8821
1.7871
1.4518
ClH•H2N
N
Boc
30
3.01
1.09
2.01
4.03
2.04
4.07
9.00
f1 (ppm)

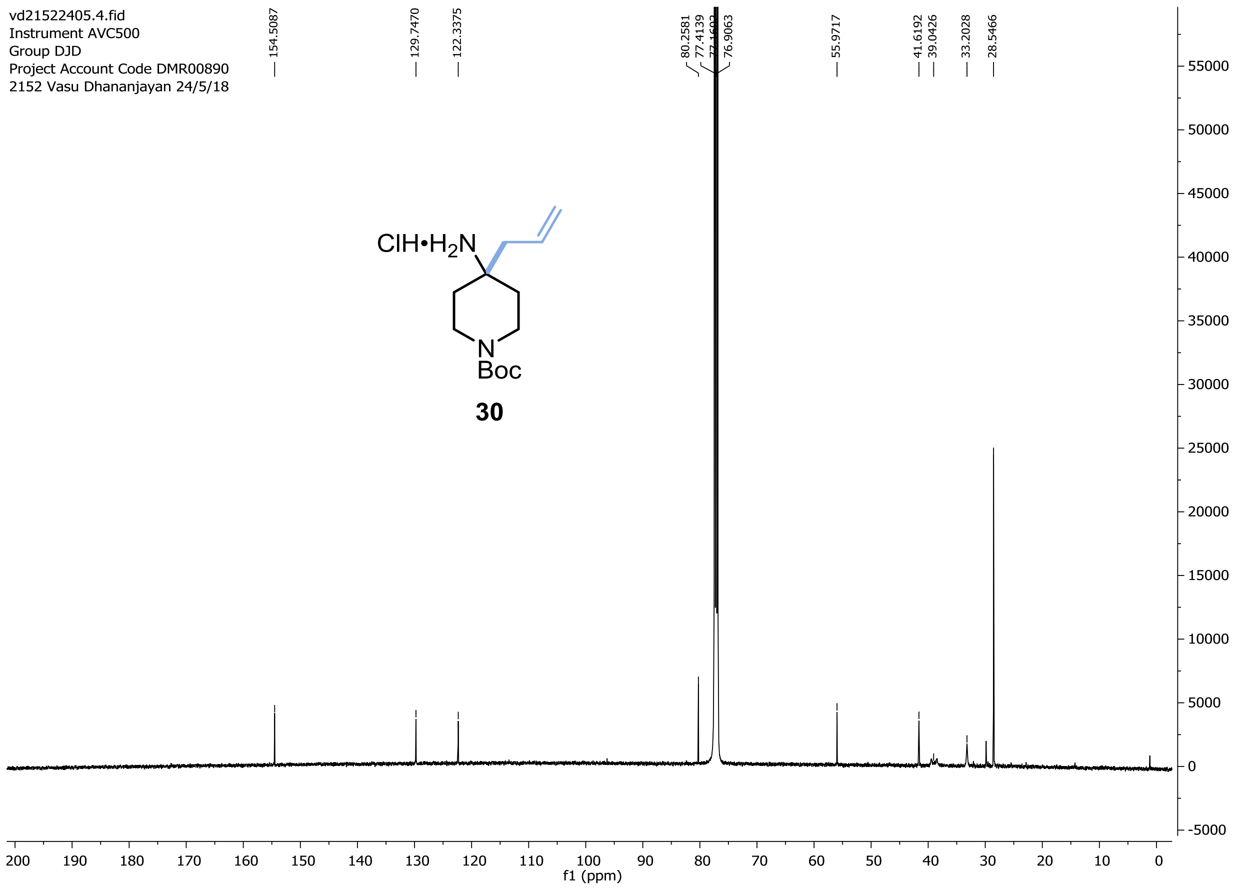
vd21522405.4.fid
Instrument AVC500
Group DJD
Project Account Code DMR00890
2152 Vasu Dhananjayan 24/5/18
154.5087
129.7470
122.3375
80.2581
77.4139
76.9063
55.9717
41.6192
39.0426
33.2028
28.5466
ClH•H2N
N
Boc
30
f1 (ppm)

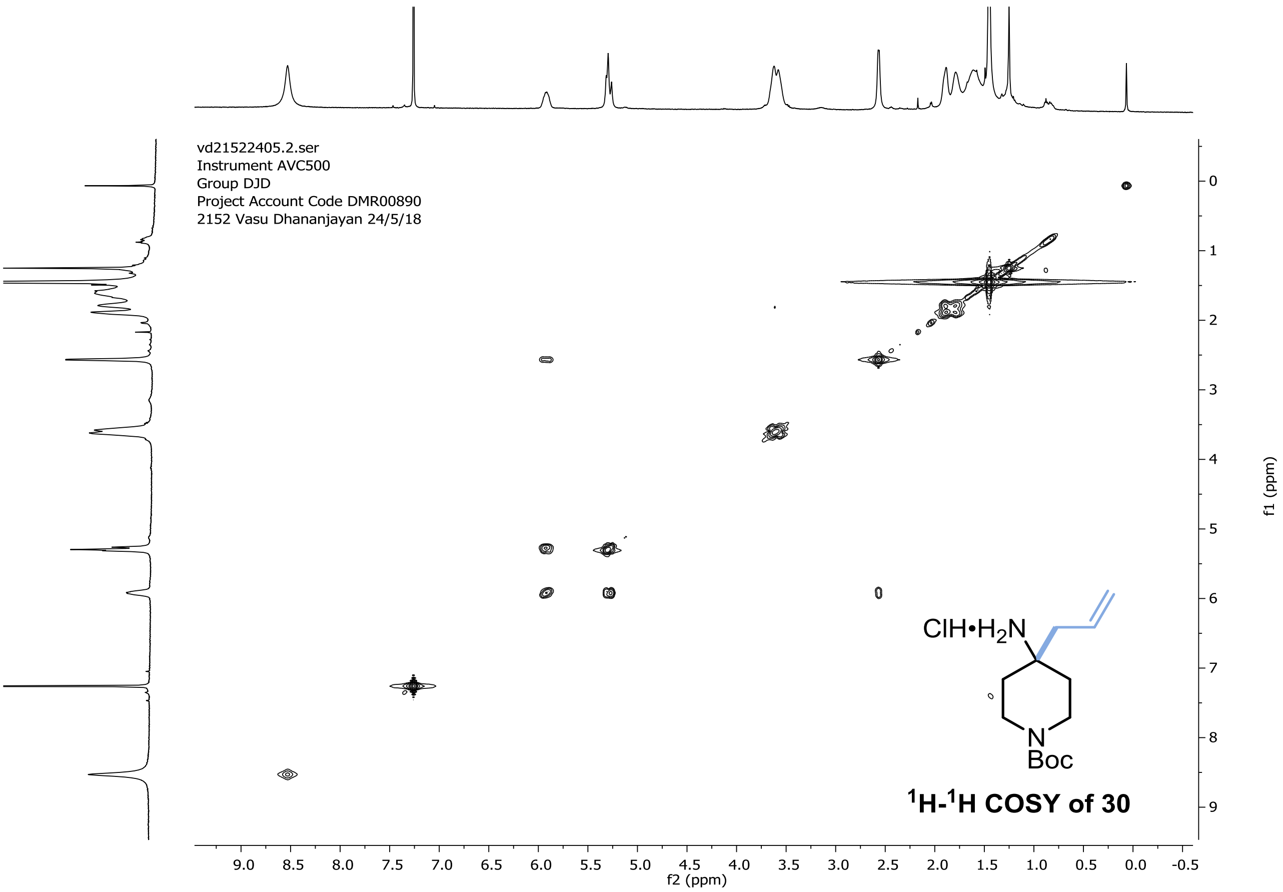

^{1}H-^{1}H COSY of 30

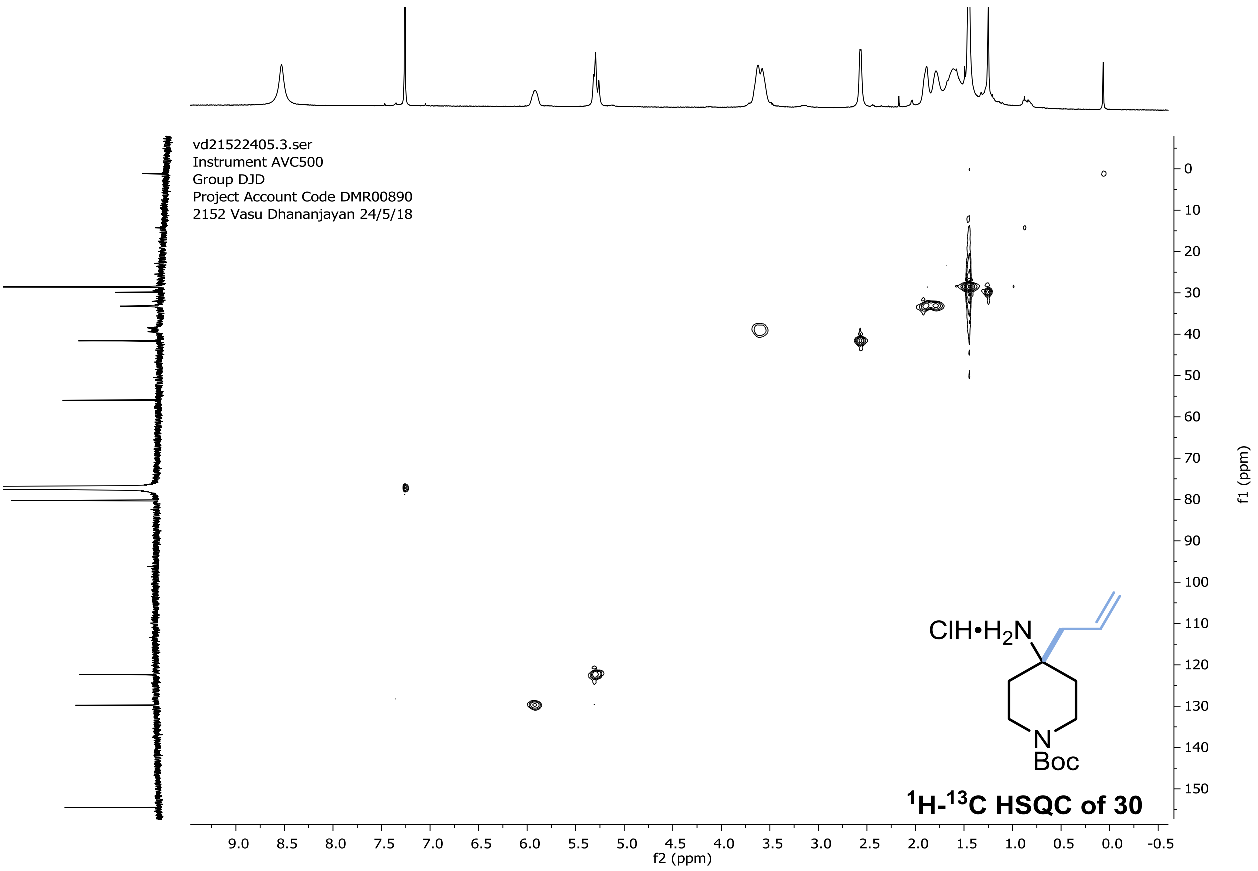

^{1}H-^{13}C HSQC of 30

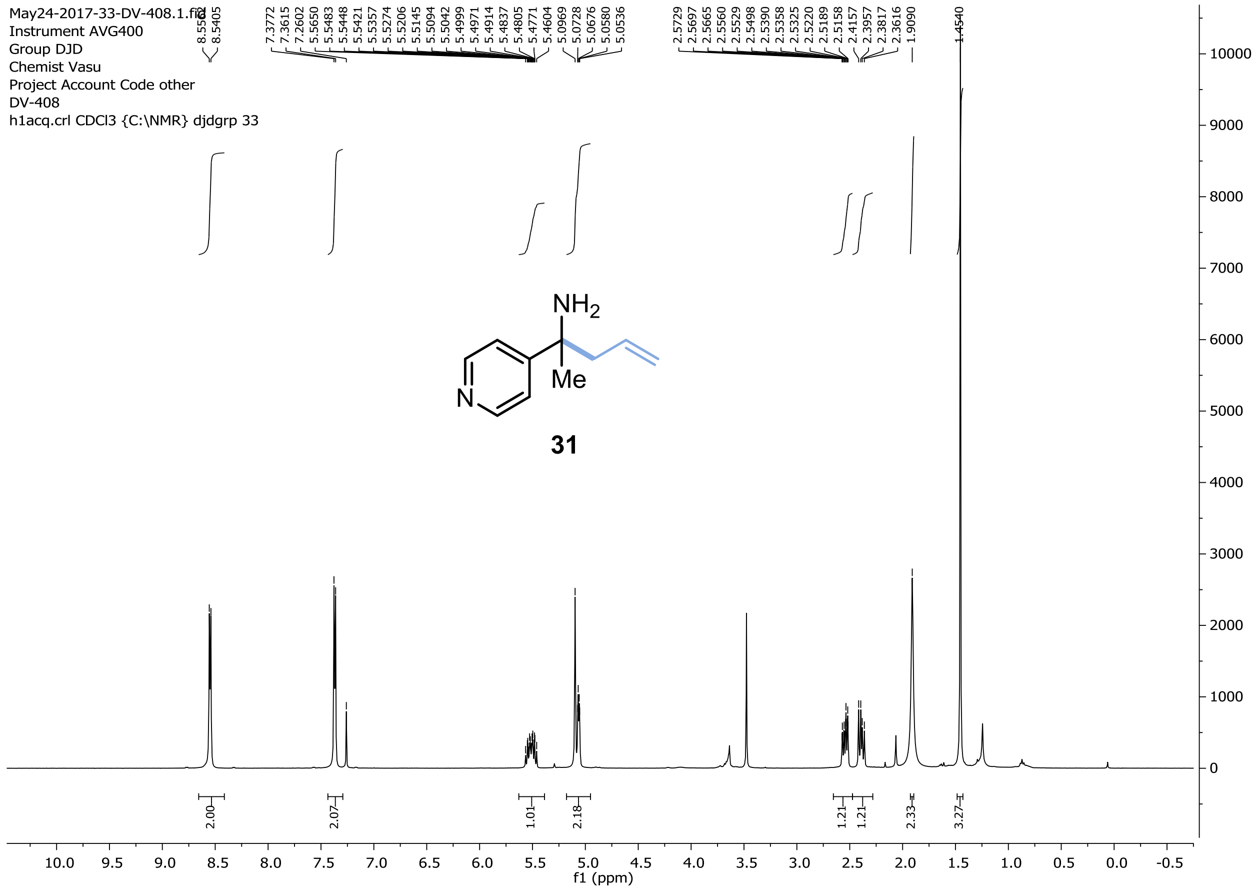
May24-2017-33-DV-408.1.fid
Instrument AVG400
Group DJD
Chemist Vasu
Project Account Code other
DV-408
h1acq.crl CDCl3 {C:\NMR} djdgrp 33
8.5405
7.3772
7.3615
7.2602
5.5650
5.5483
5.5448
5.5421
5.5357
5.5274
5.5206
5.5145
5.5094
5.5042
5.4999
5.4971
5.4914
5.4837
5.4805
5.4771
5.4604
5.0969
5.0728
5.0676
5.0580
5.0536
2.5729
2.5697
2.5665
2.5560
2.5529
2.5498
2.5390
2.5358
2.5325
2.5220
2.5189
2.5158
2.4157
2.3957
2.3817
2.3616
1.9090
1.4540
NH2
Me
N
31
2.00
2.07
1.01
2.18
1.21
1.21
2.33
3.27
f1 (ppm)

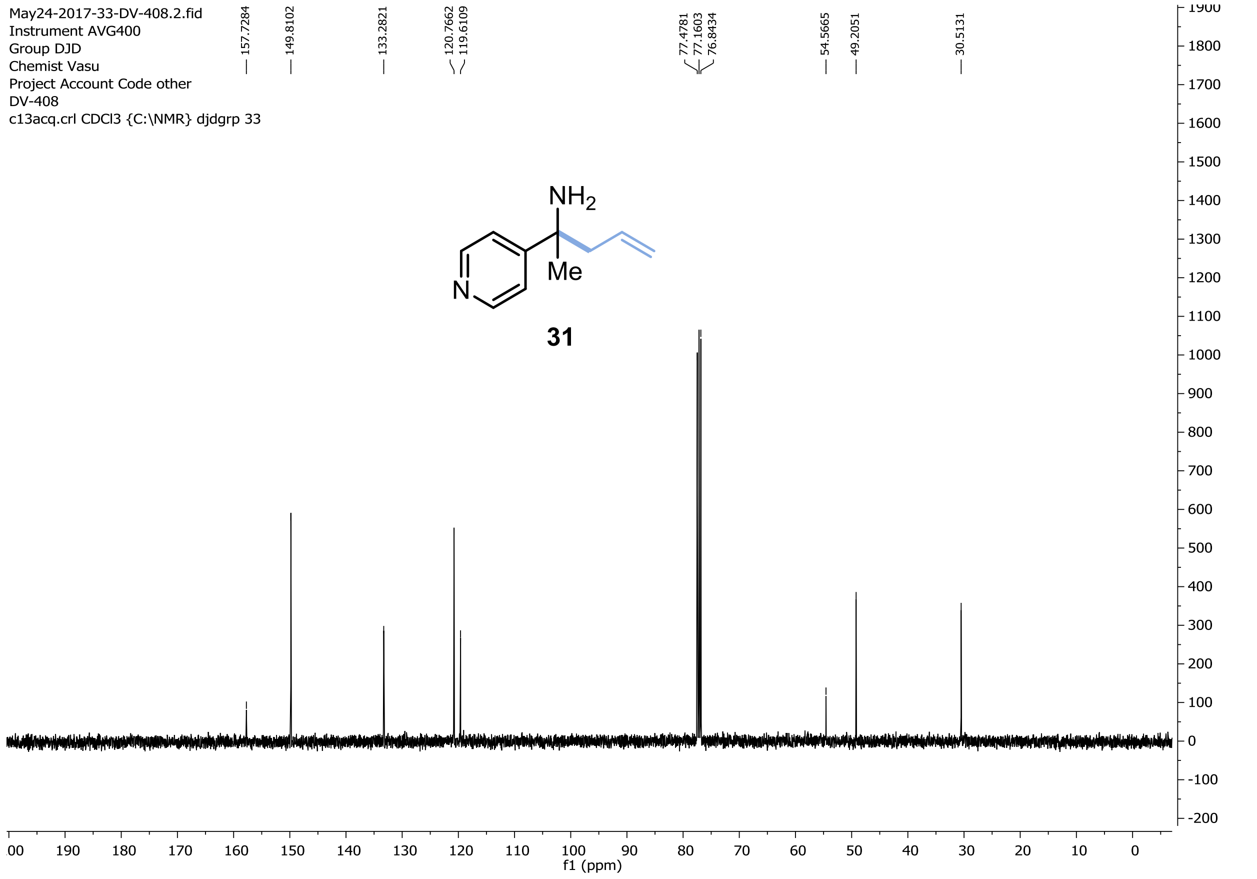
May24-2017-33-DV-408.2.fid
Instrument AVG400
Group DJD
Chemist Vasu
Project Account Code other
DV-408
c13acq.crl CDCl3 {C:\NMR} djdgrp 33
157.7284
149.8102
133.2821
120.7662
119.6109
77.4781
77.1603
76.8434
54.5665
49.2051
30.5131
NH2
Me
N
31
f1 (ppm)

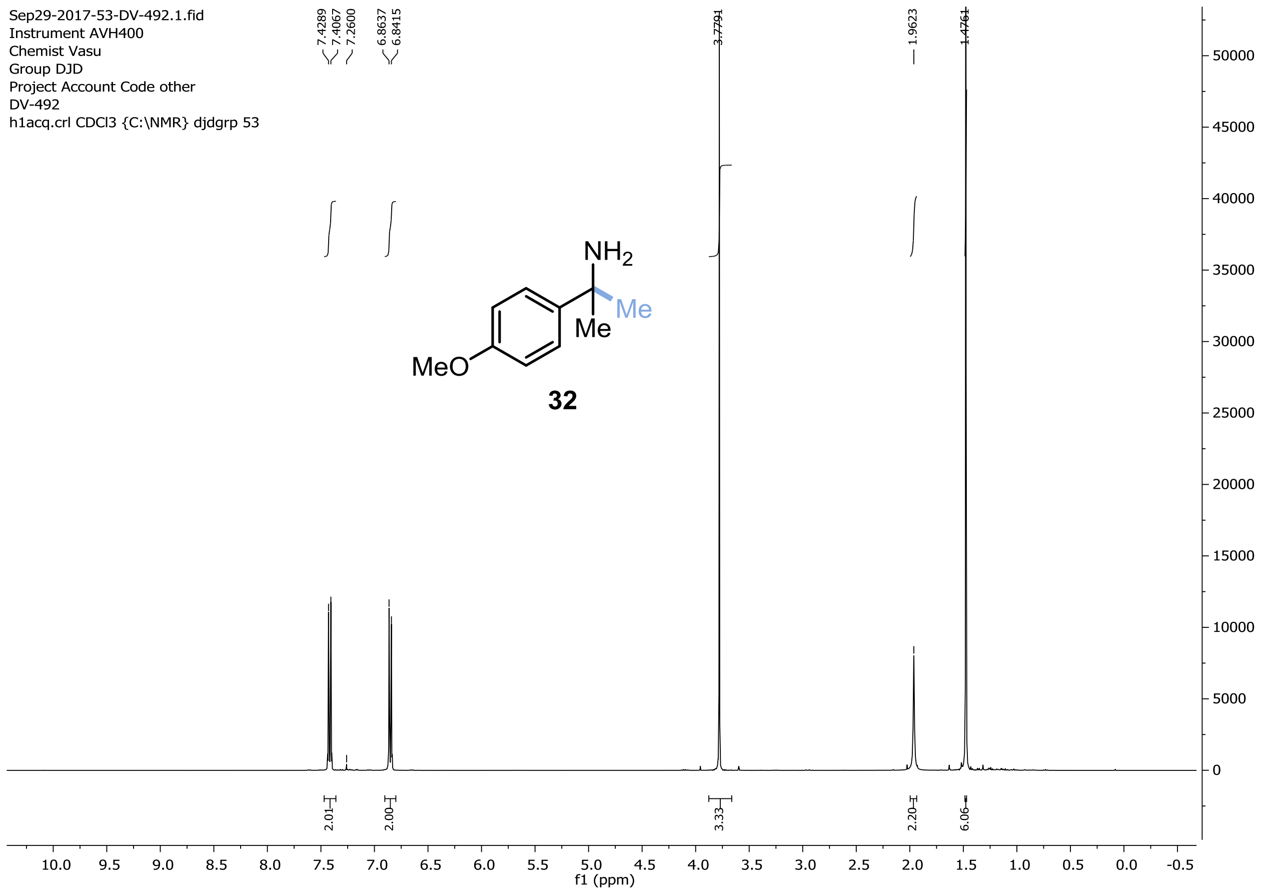
Sep29-2017-53-DV-492.1.fid
Instrument AVH400
Chemist Vasu
Group DJD
Project Account Code other
DV-492
h1acq.crl CDCl3 {C:\NMR} djdgrp 53
7.4289
7.4067
7.2600
6.8637
6.8415
3.7791
1.9623
1.4761
NH2
Me
Me
MeO
32
2.01
2.00
3.33
2.20
6.06
f1 (ppm)

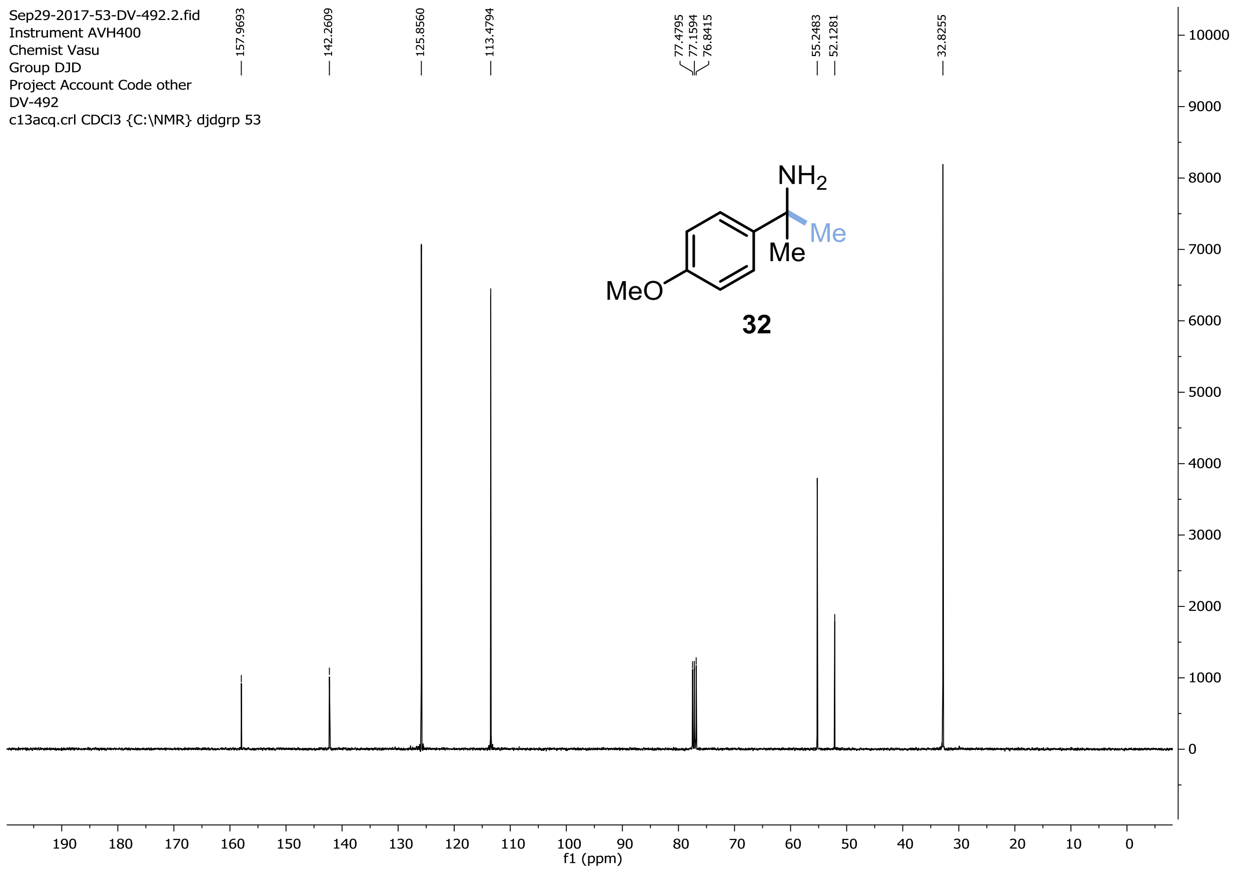
Sep29-2017-53-DV-492.2.fid
Instrument AVH400
Chemist Vasu
Group DJD
Project Account Code other
DV-492
c13acq.crl CDCl3 {C:\NMR} djdgrp 53
157.9693
142.2609
125.8560
113.4794
77.4795
77.1594
76.8415
55.2483
52.1281
32.8255
NH2
Me
Me
MeO
32
f1 (ppm)

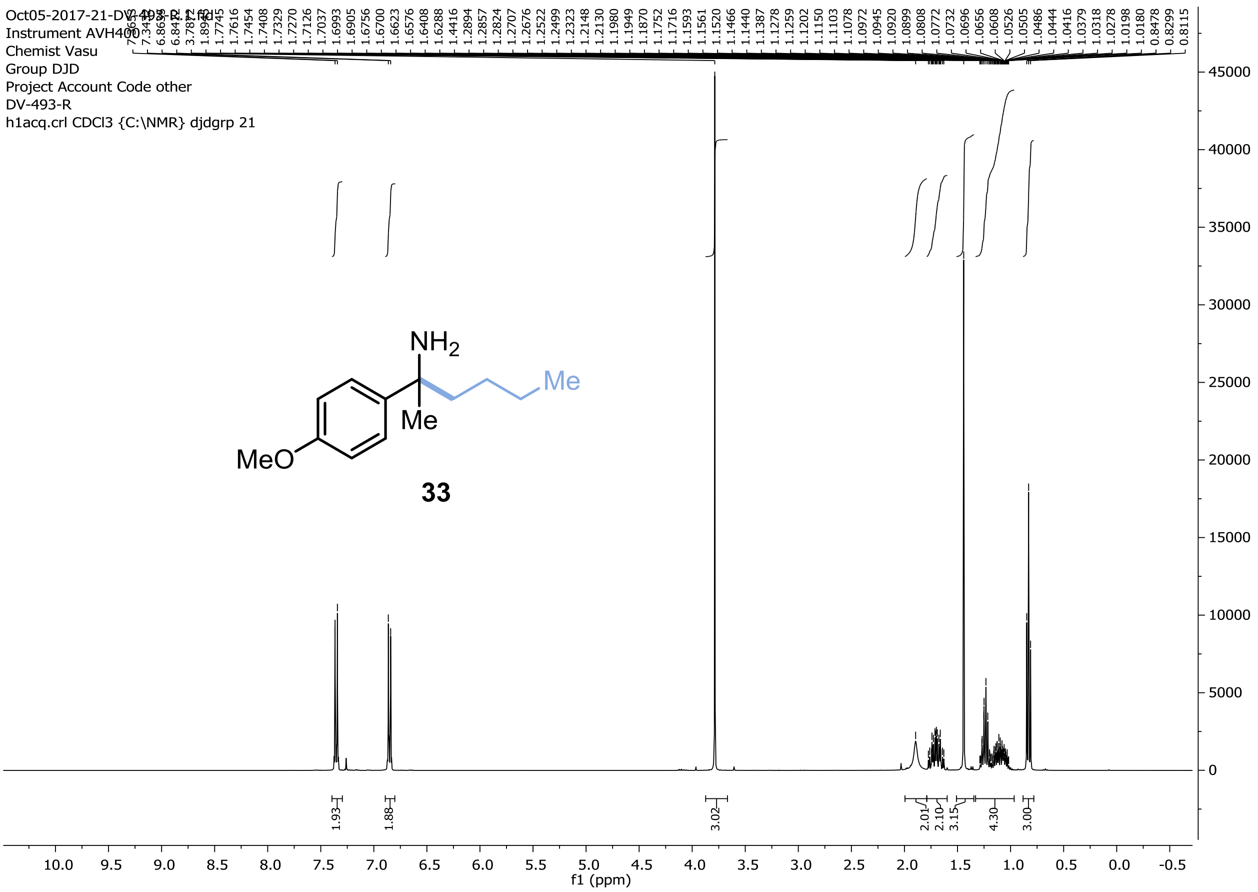
Oct05-2017-21-DV-493-R.1.fid
Instrument AVH400
Chemist Vasu
Group DJD
Project Account Code other
DV-493-R
h1acq.crl CDCl3 {C:\NMR} djdgrp 21
NH2
Me
Me
MeO
33
f1 (ppm)

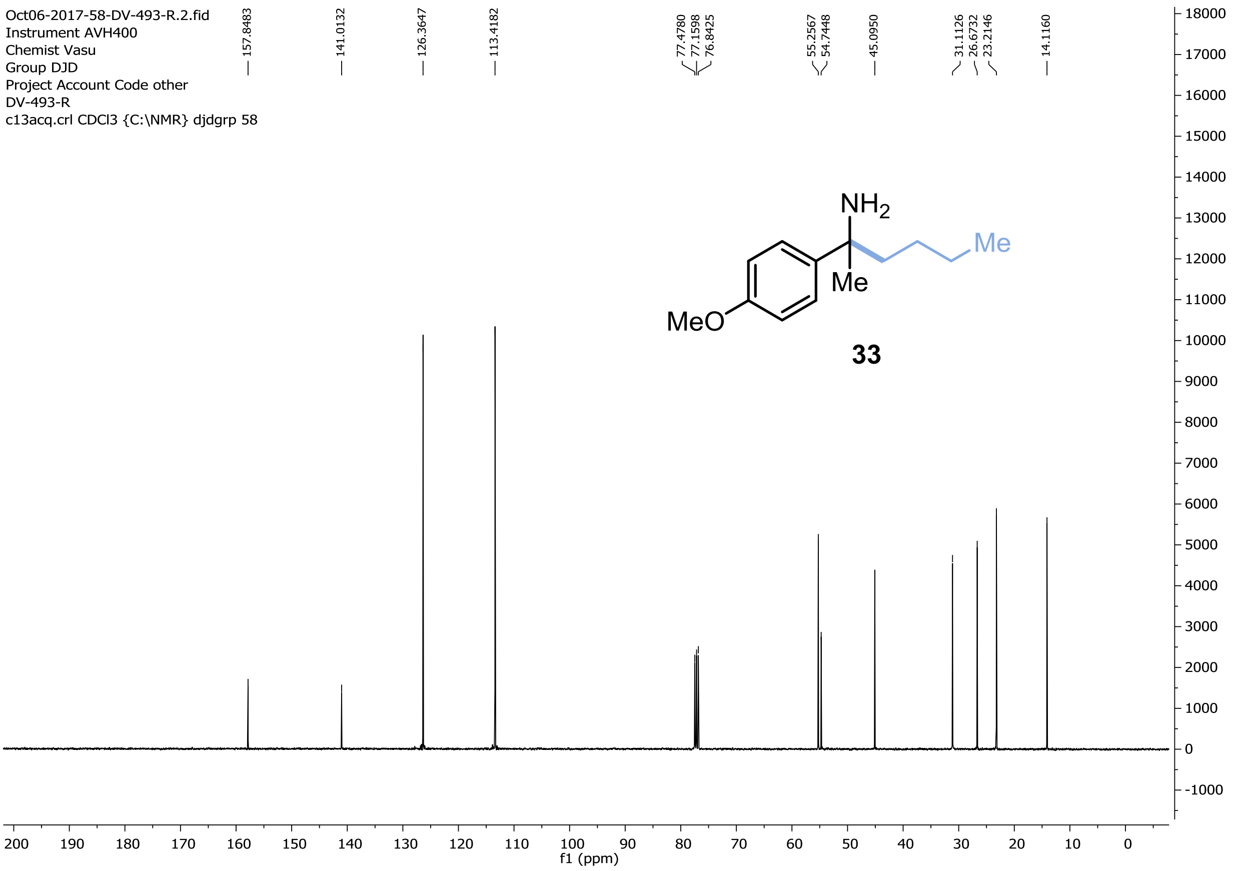
Oct06-2017-58-DV-493-R.2.fid
Instrument AVH400
Chemist Vasu
Group DJD
Project Account Code other
DV-493-R
c13acq.crl CDCl3 {C:\NMR} djdgrp 58
157.8483
141.0132
126.3647
113.4182
77.4780
77.1598
76.8425
55.2567
54.7448
45.0950
31.1126
26.6732
23.2146
14.1160
NH2
Me
Me
MeO
33
f1 (ppm)

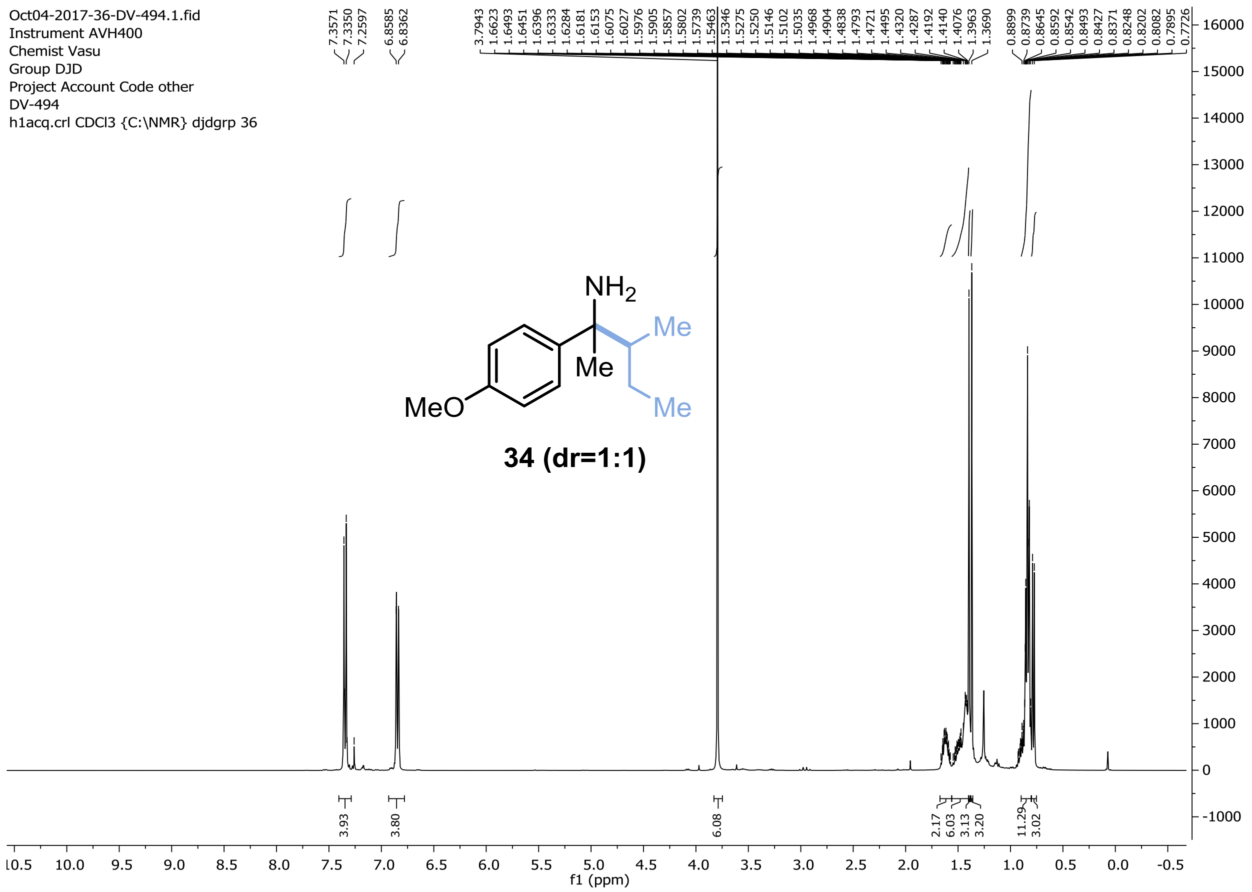
Oct04-2017-36-DV-494.1.fid
Instrument AVH400
Chemist Vasu
Group DJD
Project Account Code other
DV-494
h1acq.crl CDCl3 {C:\NMR} djdgrp 36
NH2
Me
Me
MeO
Me
34 (dr=1:1)
f1 (ppm)

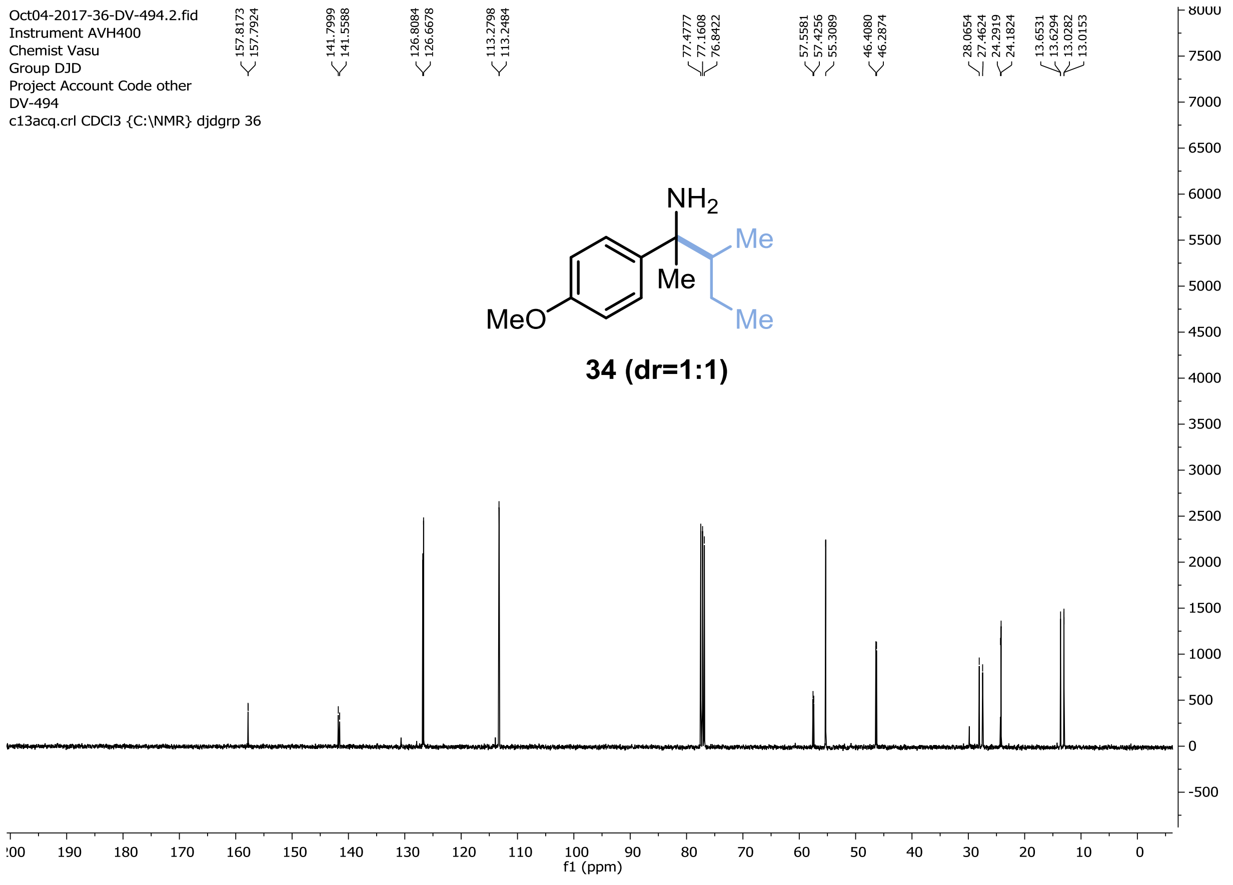
Oct04-2017-36-DV-494.2.fid
Instrument AVH400
Chemist Vasu
Group DJD
Project Account Code other
DV-494
c13acq.crl CDCl3 {C:\NMR} djdgrp 36
NH2
Me
Me
MeO
Me
34 (dr=1:1)
f1 (ppm)

Oct13-2017-34-DV-499-HCl.1.fid
Instrument AVH400
Chemist Vasu
Group DJD
Project Account Code other
DV-499-HCl
h1acq.crl CDCl3 {C:\NMR} djdgrp 34

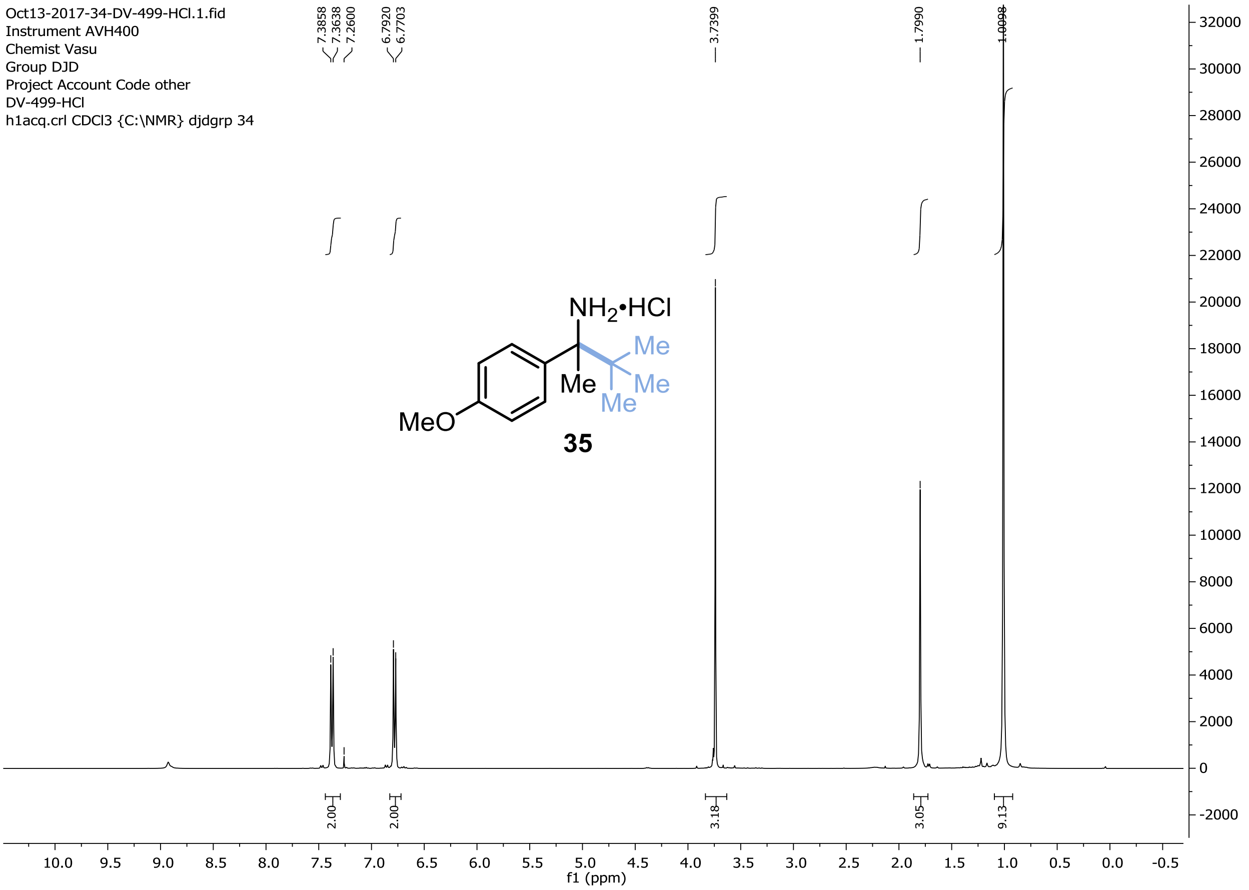

Oct13-2017-34-DV-499-HCl.2.fid
Instrument AVH400
Chemist Vasu
Group DJD
Project Account Code other
DV-499-HCl
c13acq.crl CDCl3 {C:\NMR} djdgrp 34

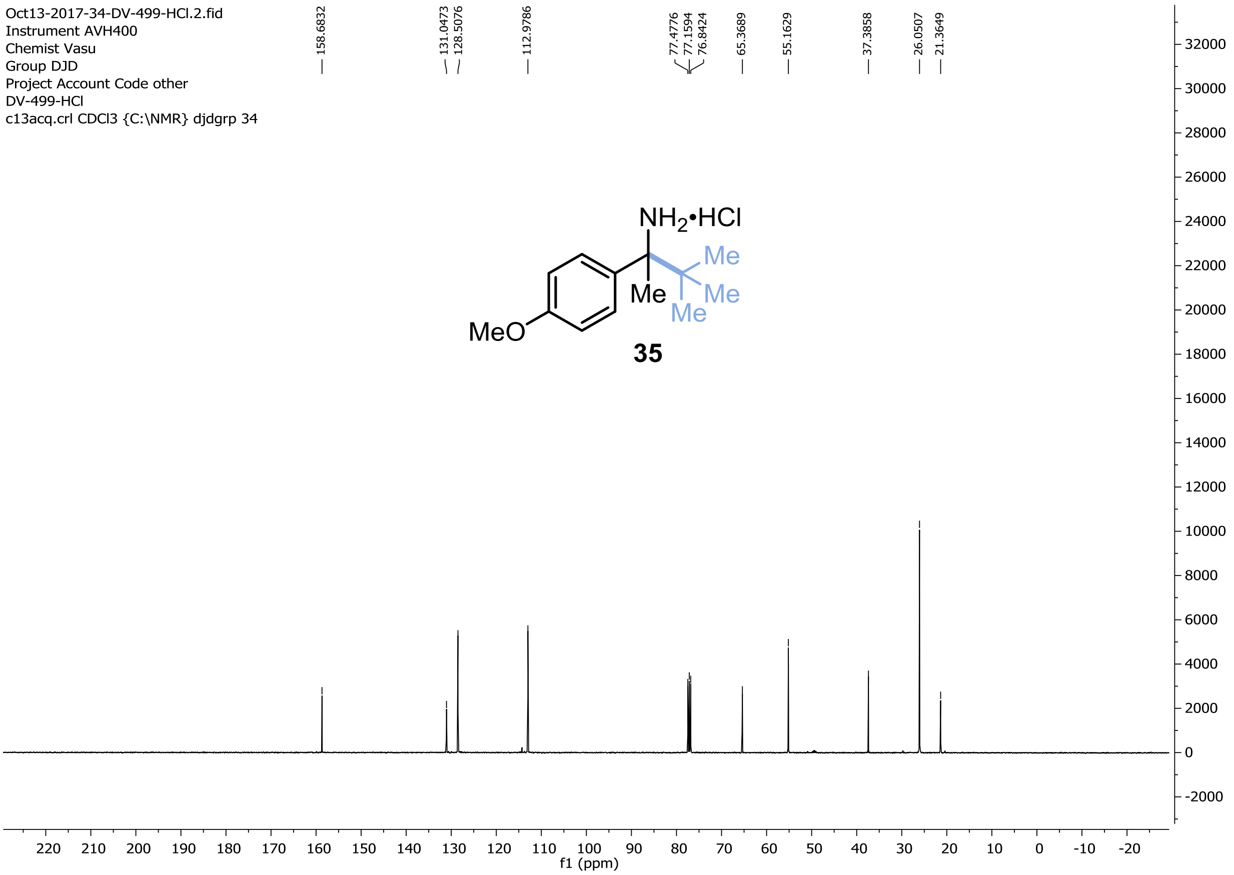

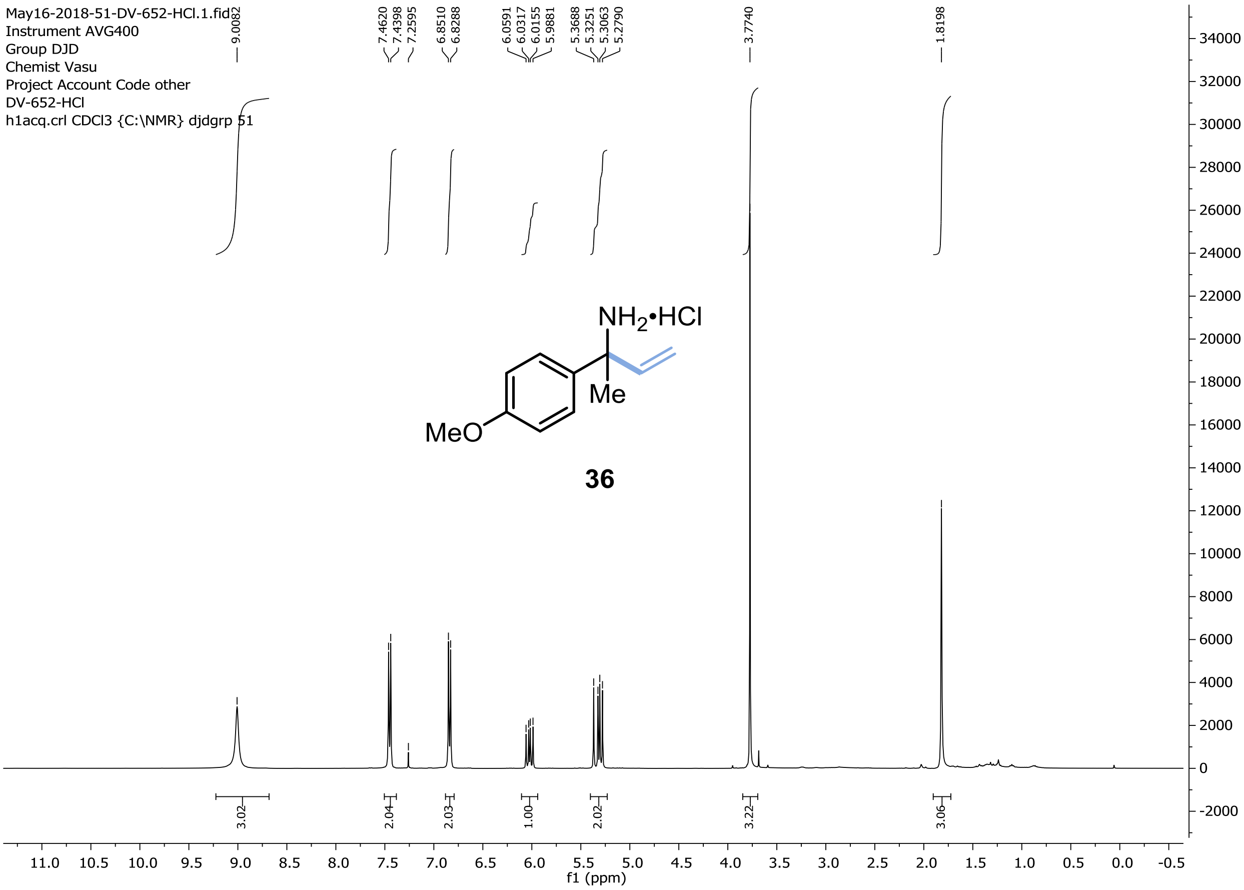
May16-2018-51-DV-652-HCl.1.fid
Instrument AVG400
Group DJD
Chemist Vasu
Project Account Code other
DV-652-HCl
h1acq.crl CDCl3 {C:\NMR} djdgrp 51
9.0082
7.4620
7.4398
7.2595
6.8510
6.8288
6.0591
6.0317
6.0155
5.9881
5.3688
5.3251
5.3063
5.2790
3.7740
1.8198
NH2•HCl
Me
MeO
36
3.02
2.04
2.03
1.00
2.02
3.22
3.06
f1 (ppm)

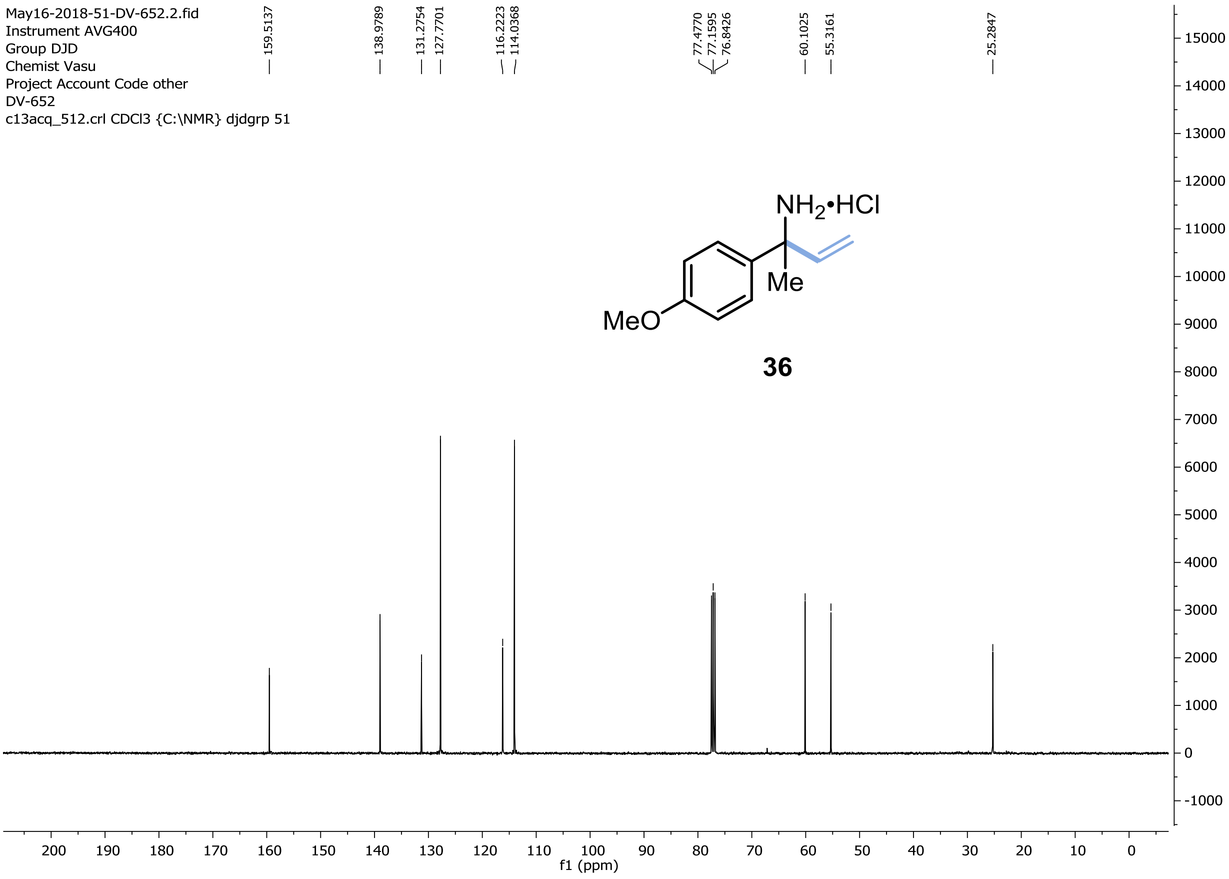
May16-2018-51-DV-652.2.fid
Instrument AVG400
Group DJD
Chemist Vasu
Project Account Code other
DV-652
c13acq_512.crl CDCl3 {C:\NMR} djdgrp 51
159.5137
138.9789
131.2754
127.7701
116.2223
114.0368
77.4770
77.1595
76.8426
60.1025
55.3161
25.2847
NH2•HCl
Me
MeO
36
f1 (ppm)

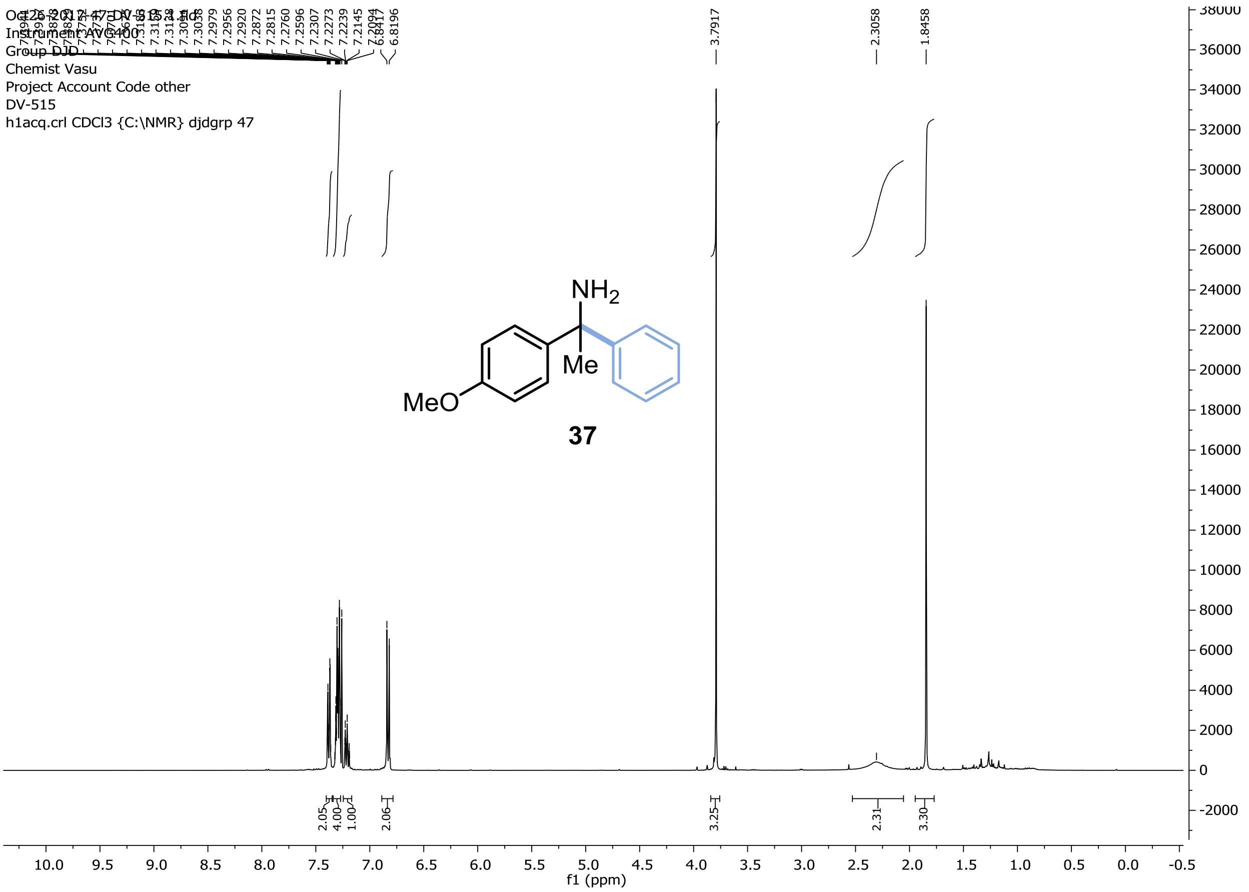
Instrument AVG400
Group DJD
Chemist Vasu
Project Account Code other
DV-515
h1acq.crl CDCl3 {C:\NMR} djdgrp 47
37
f1 (ppm)

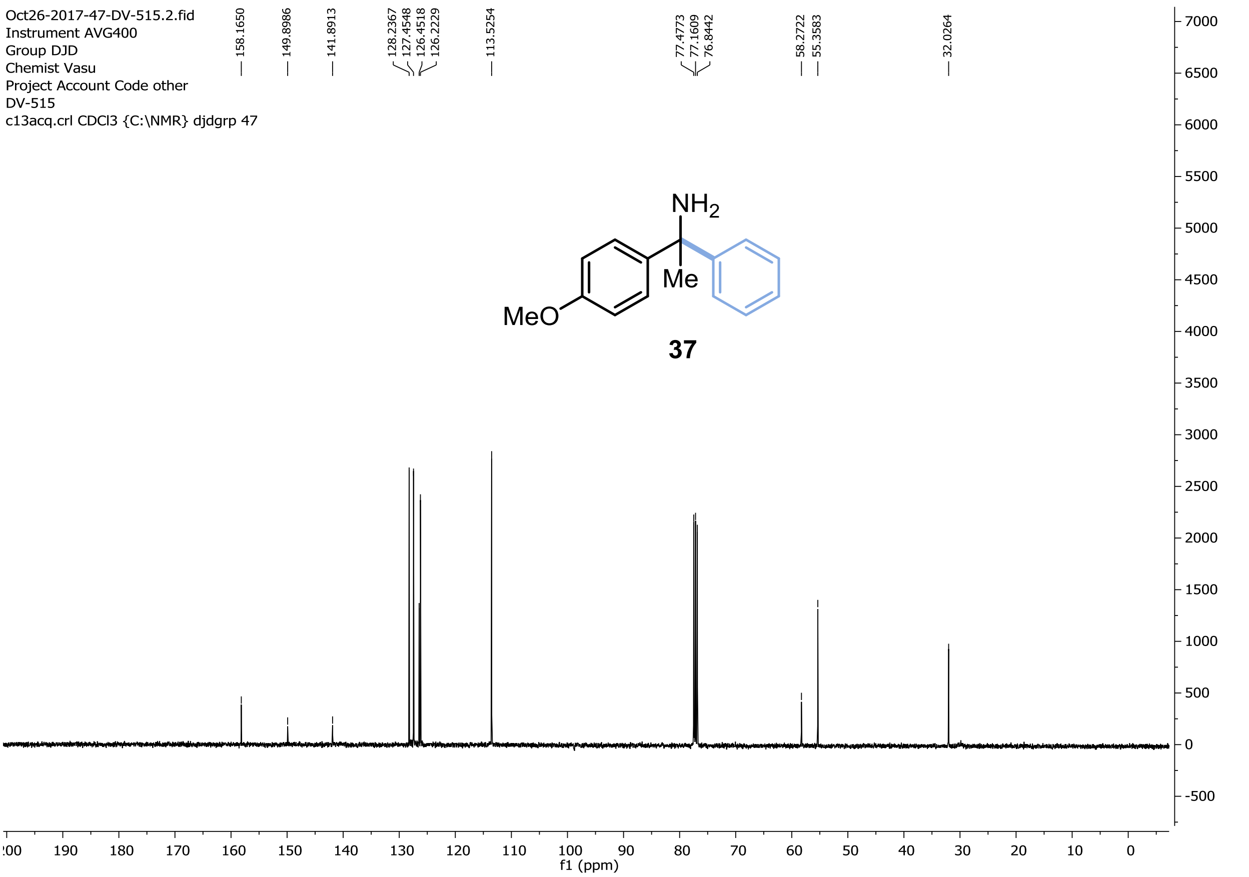
Oct26-2017-47-DV-515.2.fid
Instrument AVG400
Group DJD
Chemist Vasu
Project Account Code other
DV-515
c13acq.crl CDCl3 {C:\NMR} djdgrp 47
37
f1 (ppm)

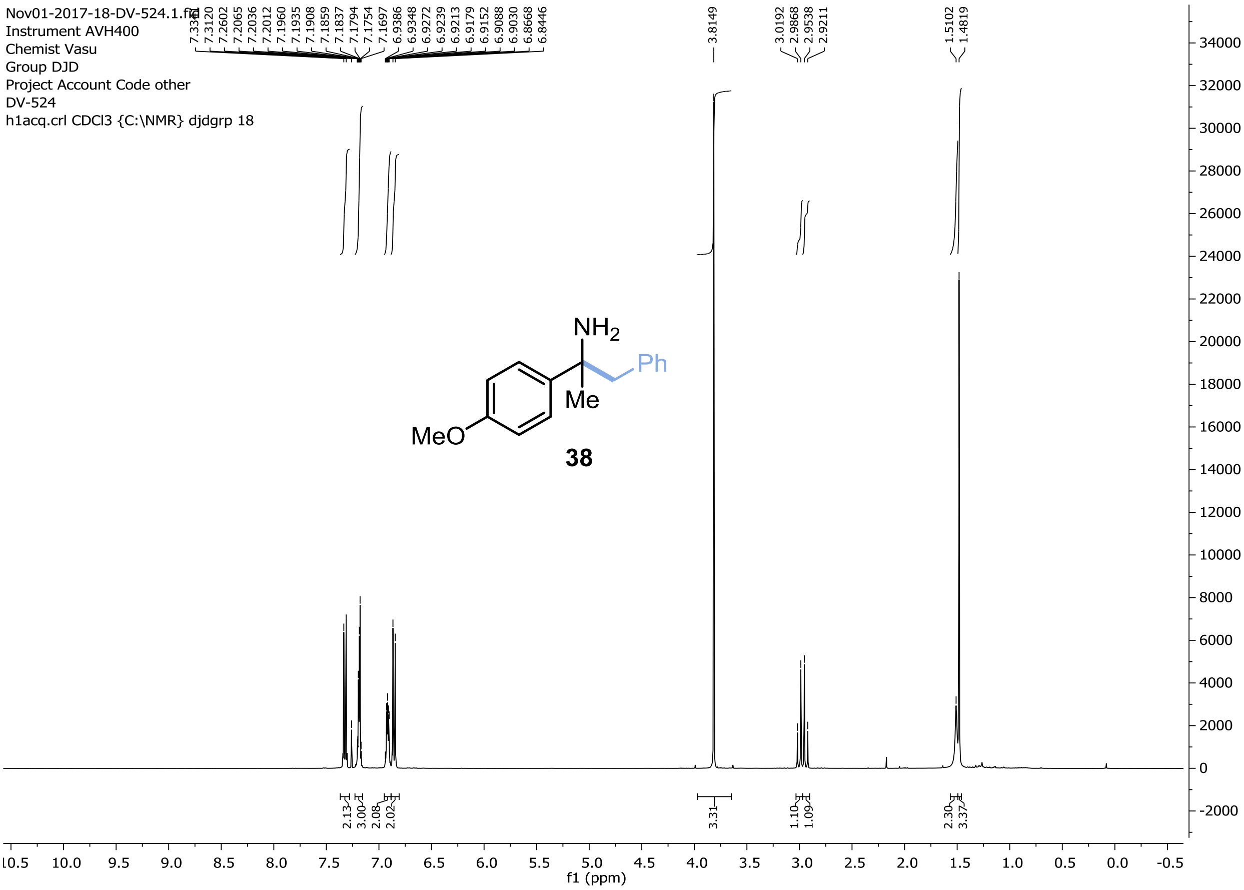
Nov01-2017-18-DV-524.1.fid
Instrument AVH400
Chemist Vasu
Group DJD
Project Account Code other
DV-524
h1acq.crl CDCl3 {C:\NMR} djdgrp 18
7.3120
7.2602
7.2065
7.2036
7.2012
7.1960
7.1935
7.1908
7.1859
7.1837
7.1794
7.1754
7.1697
6.9386
6.9348
6.9272
6.9239
6.9213
6.9179
6.9152
6.9088
6.9030
6.8668
6.8446
3.8149
3.0192
2.9868
2.9538
2.9211
1.5102
1.4819
NH2
Ph
Me
MeO
38
2.13
3.00
2.08
2.02
3.31
1.10
1.09
2.30
3.37
f1 (ppm)

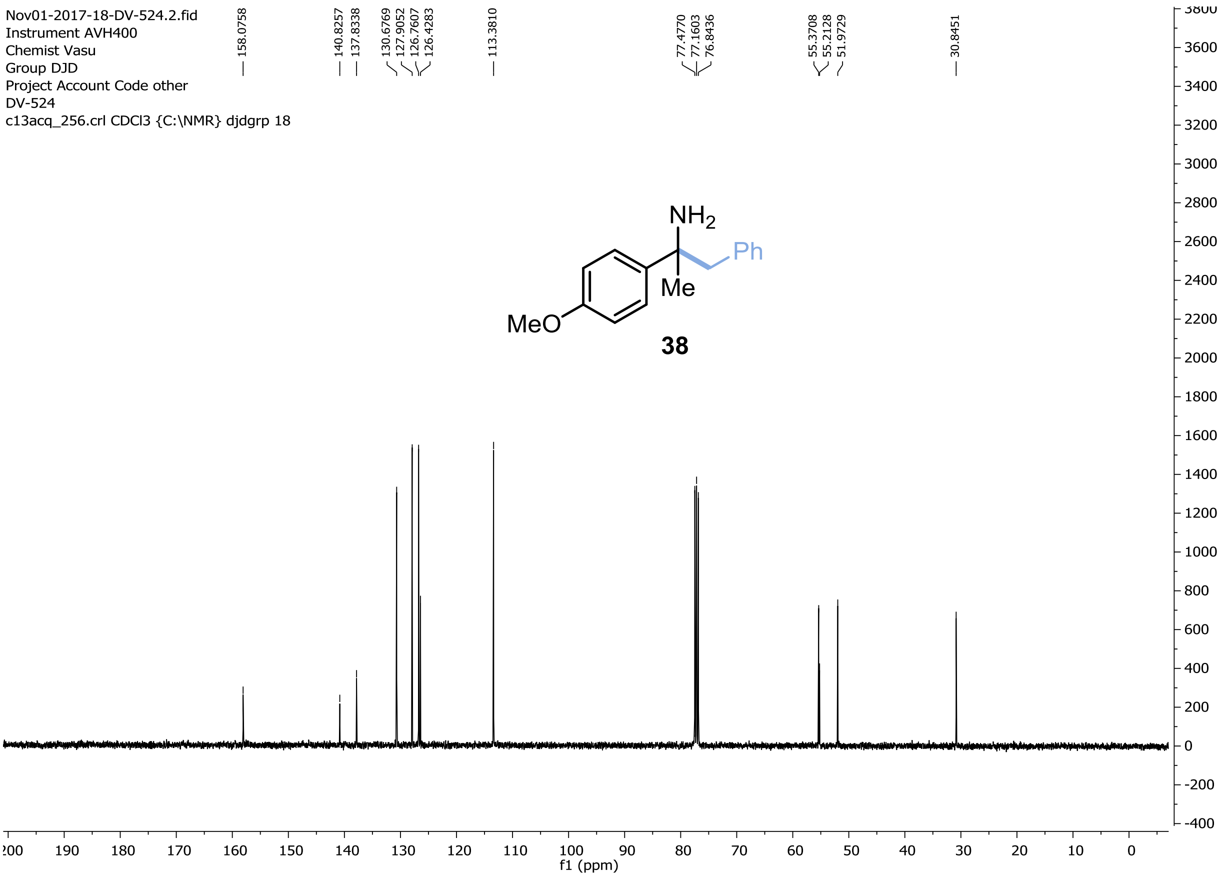
Nov01-2017-18-DV-524.2.fid
Instrument AVH400
Chemist Vasu
Group DJD
Project Account Code other
DV-524
c13acq_256.crl CDCl3 {C:\NMR} djdgrp 18
158.0758
140.8257
137.8338
130.6769
127.9052
126.7607
126.4283
113.3810
77.4770
77.1603
76.8436
55.3708
55.2128
51.9729
30.8451
NH2
Ph
Me
MeO
38
f1 (ppm)

Nov08-2017-60-DV-529.1.fid
Instrument AVG400
Group DJD
Chemist Vasu
Project Account Code other
DV-529
h1acq.crl CDCl3 {C:\NMR} djdgrp 60

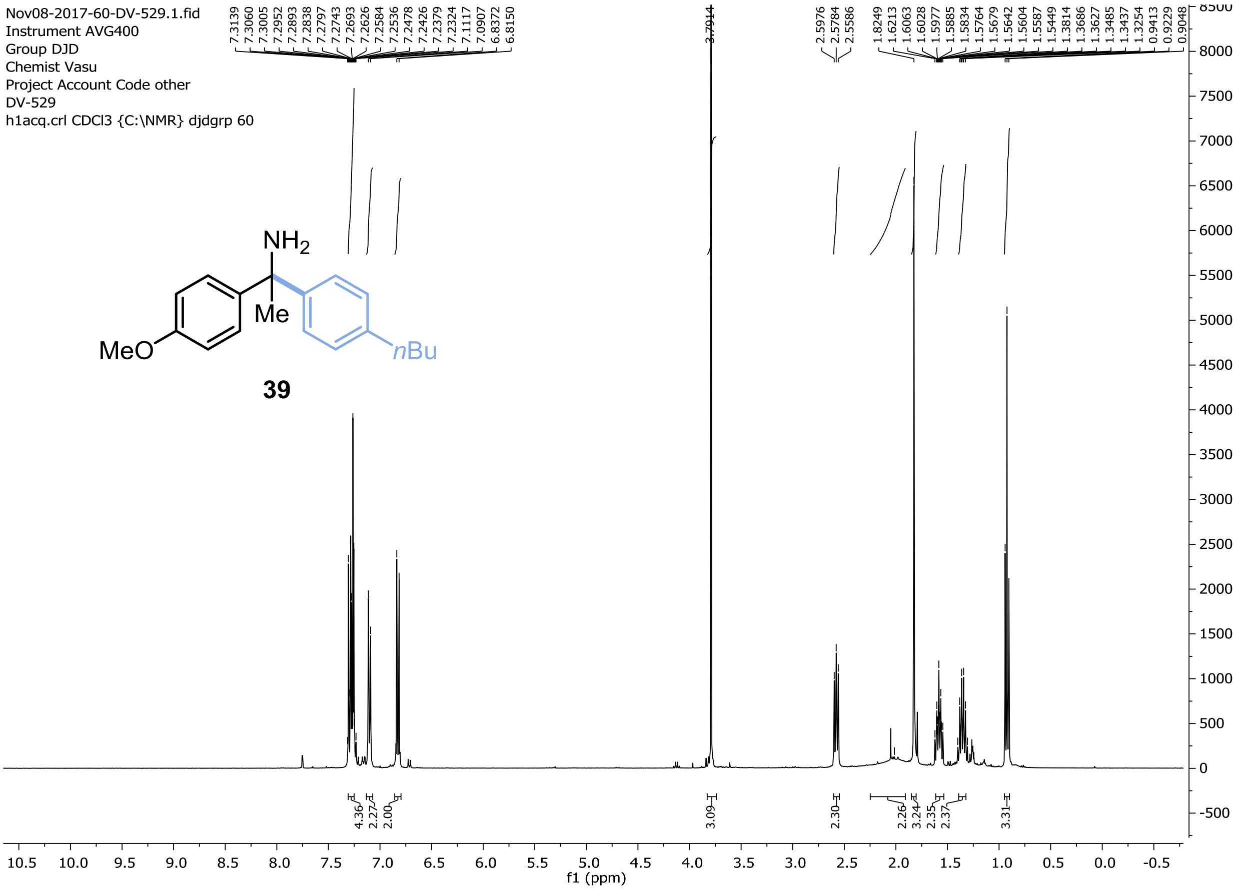

Nov06-2017-37-DV-529.2.fid
Instrument AVH400
Chemist Vasu
Group DJD
Project Account Code other
DV-529
c13acq_256.crl CDCl3 {C:\NMR} djdgrp 37

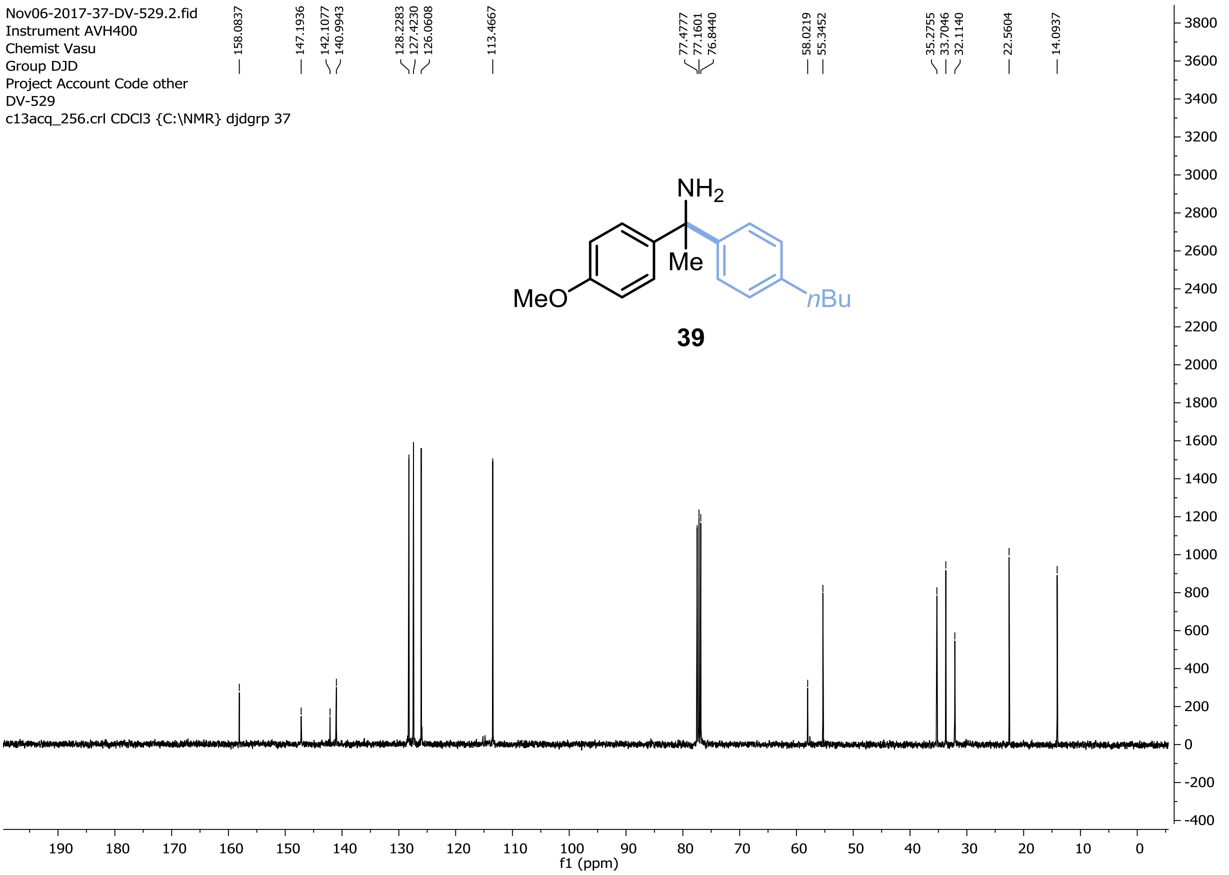

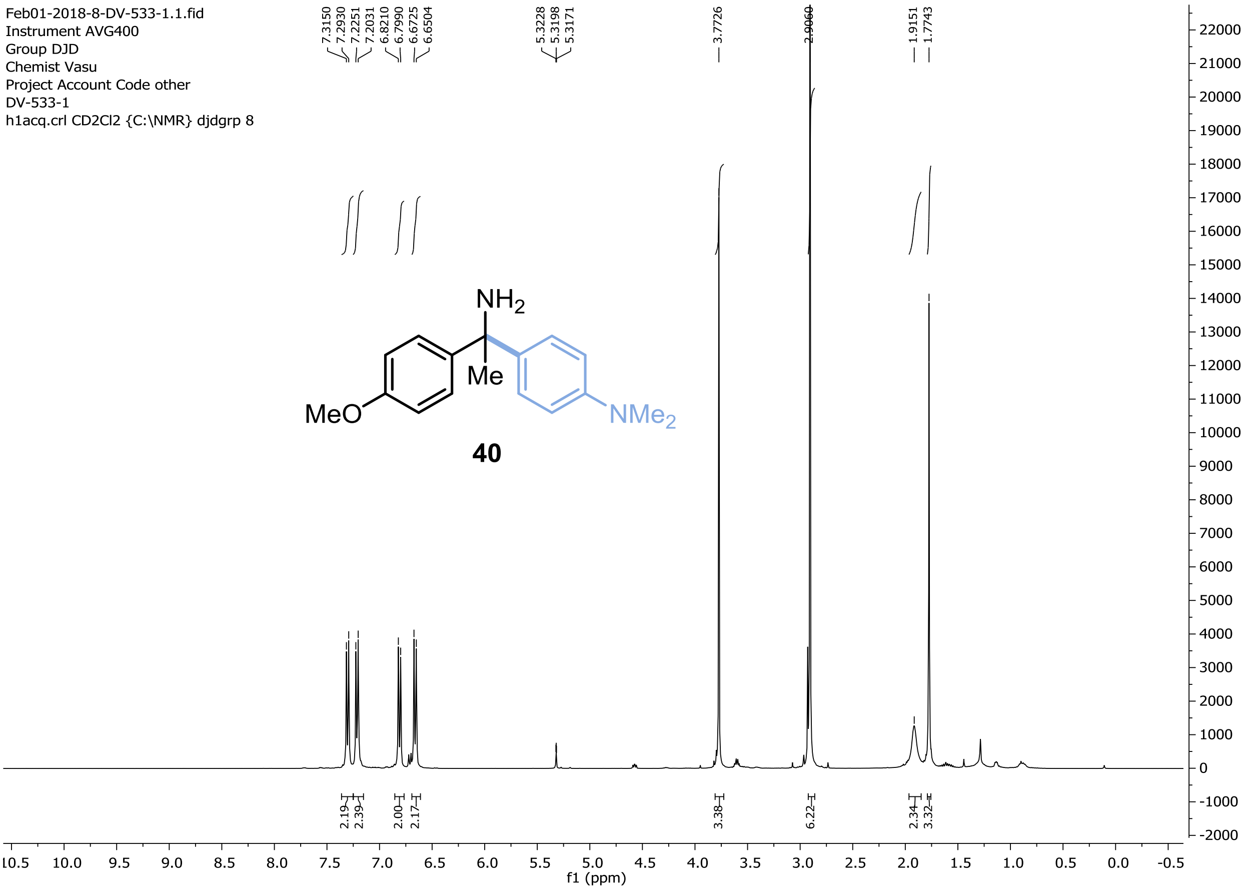
Feb01-2018-8-DV-533-1.1.fid
Instrument AVG400
Group DJD
Chemist Vasu
Project Account Code other
DV-533-1
h1acq.crl CD2Cl2 {C:\NMR} djdgrp 8
7.3150
7.2930
7.2251
7.2031
6.8210
6.7990
6.6725
6.6504
5.3228
5.3198
5.3171
3.7726
2.9060
1.9151
1.7743
NH2
Me
MeO
NMe2
40
2.19
2.39
2.00
2.17
3.38
6.22
2.34
3.32
f1 (ppm)

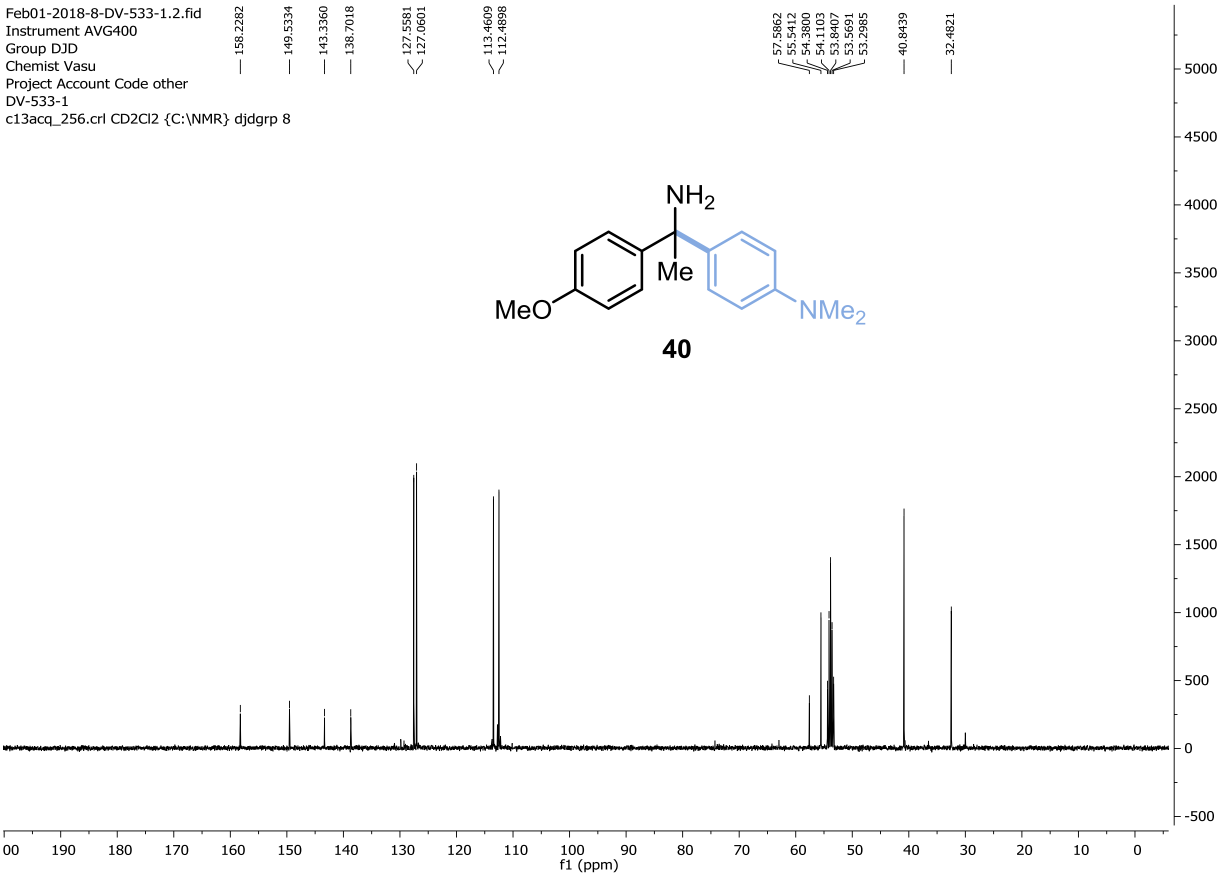
Feb01-2018-8-DV-533-1.2.fid
Instrument AVG400
Group DJD
Chemist Vasu
Project Account Code other
DV-533-1
c13acq_256.crl CD2Cl2 {C:\NMR} djdgrp 8
158.2282
149.5334
143.3360
138.7018
127.5581
127.0601
113.4609
112.4898
57.5862
55.5412
54.3800
54.1103
53.8407
53.5691
53.2985
40.8439
32.4821
NH2
Me
MeO
NMe2
40
f1 (ppm)

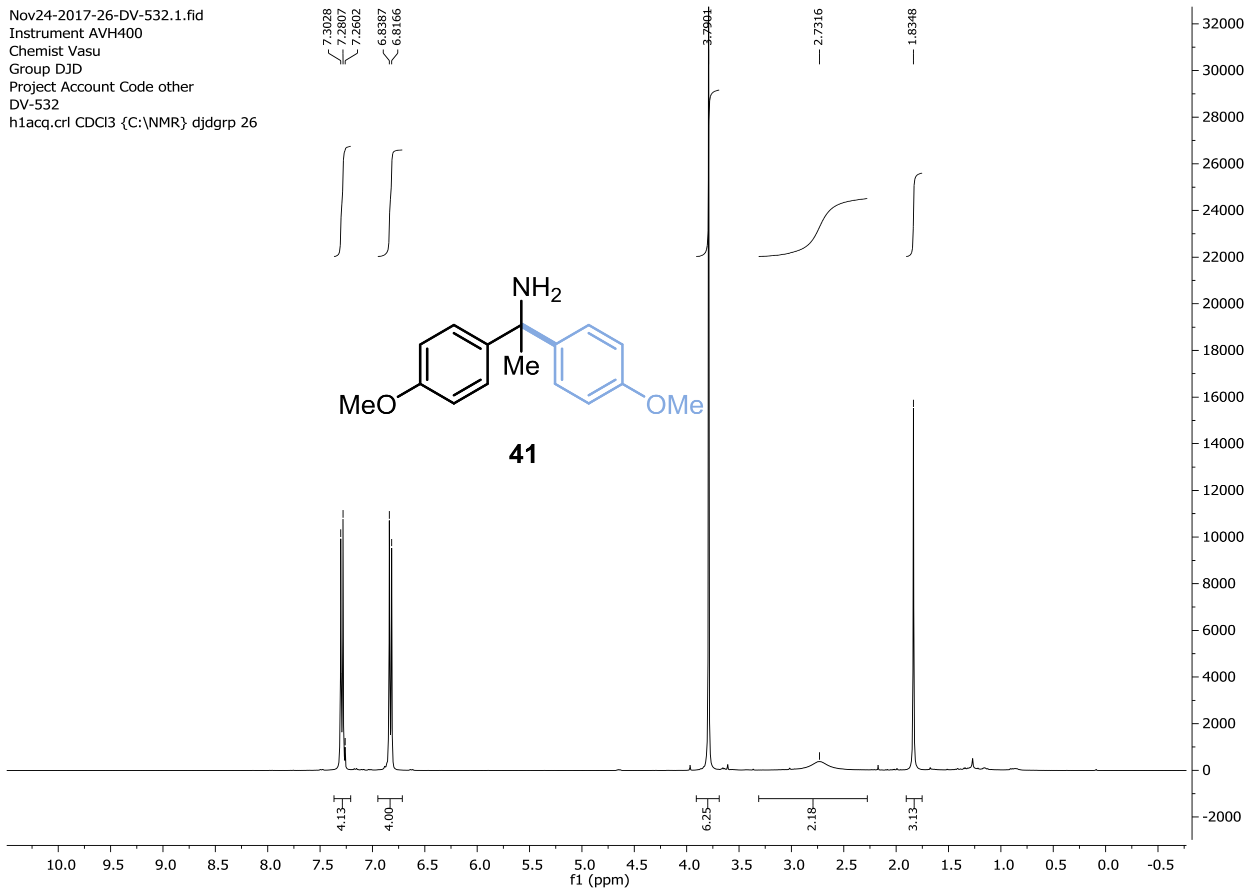

Nov24-2017-26-DV-532.1.fid
Instrument AVH400
Chemist Vasu
Group DJD
Project Account Code other
DV-532
h1acq.crl CDCl3 {C:\NMR} djdgrp 26
7.3028
7.2807
7.2602
6.8387
6.8166
3.7901
2.7316
1.8348
NH2
Me
MeO
OMe
41
4.13
4.00
6.25
2.18
3.13
f1 (ppm)

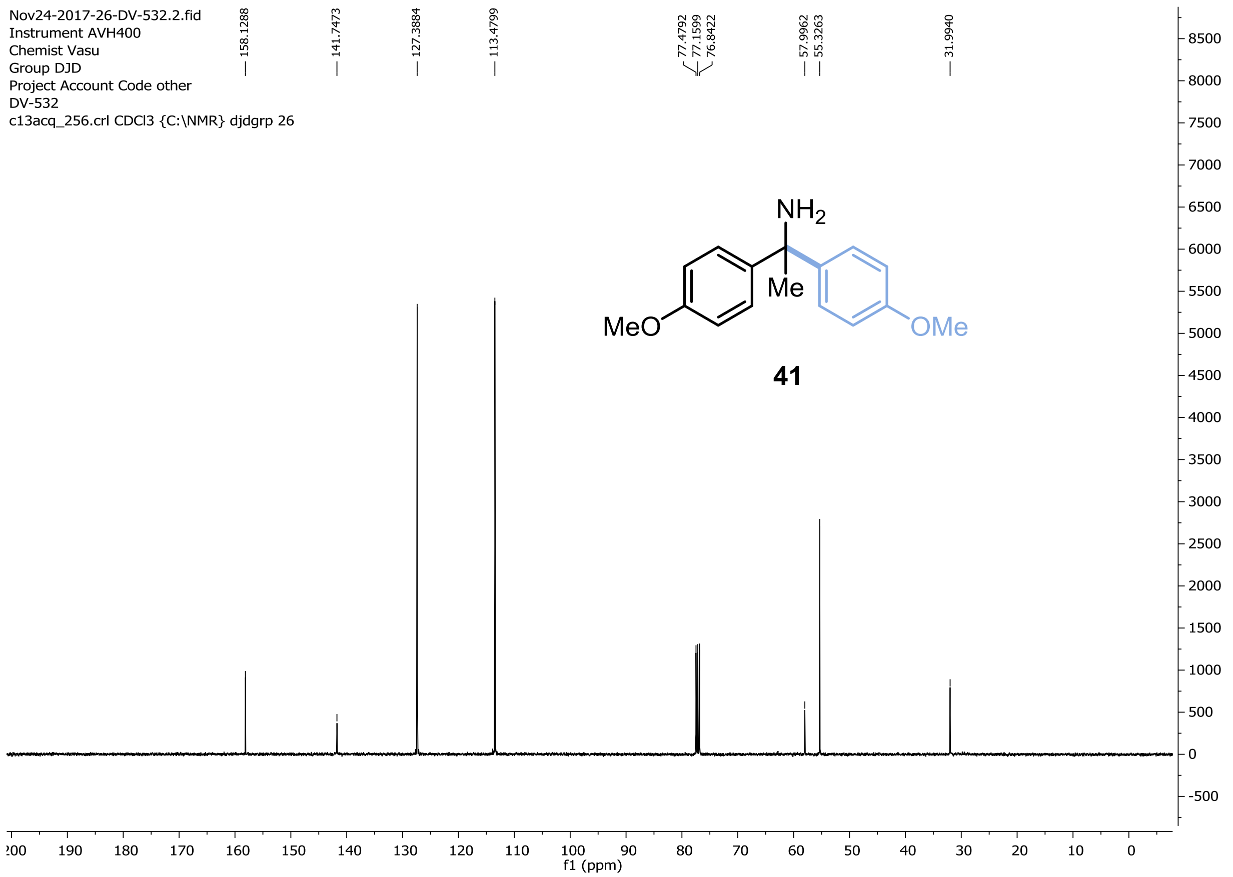

Nov24-2017-26-DV-532.2.fid
Instrument AVH400
Chemist Vasu
Group DJD
Project Account Code other
DV-532
c13acq_256.crl CDCl3 {C:\NMR} djdgrp 26
158.1288
141.7473
127.3884
113.4799
77.4792
77.1599
76.8422
57.9962
55.3263
31.9940
NH2
Me
MeO
OMe
41
f1 (ppm)

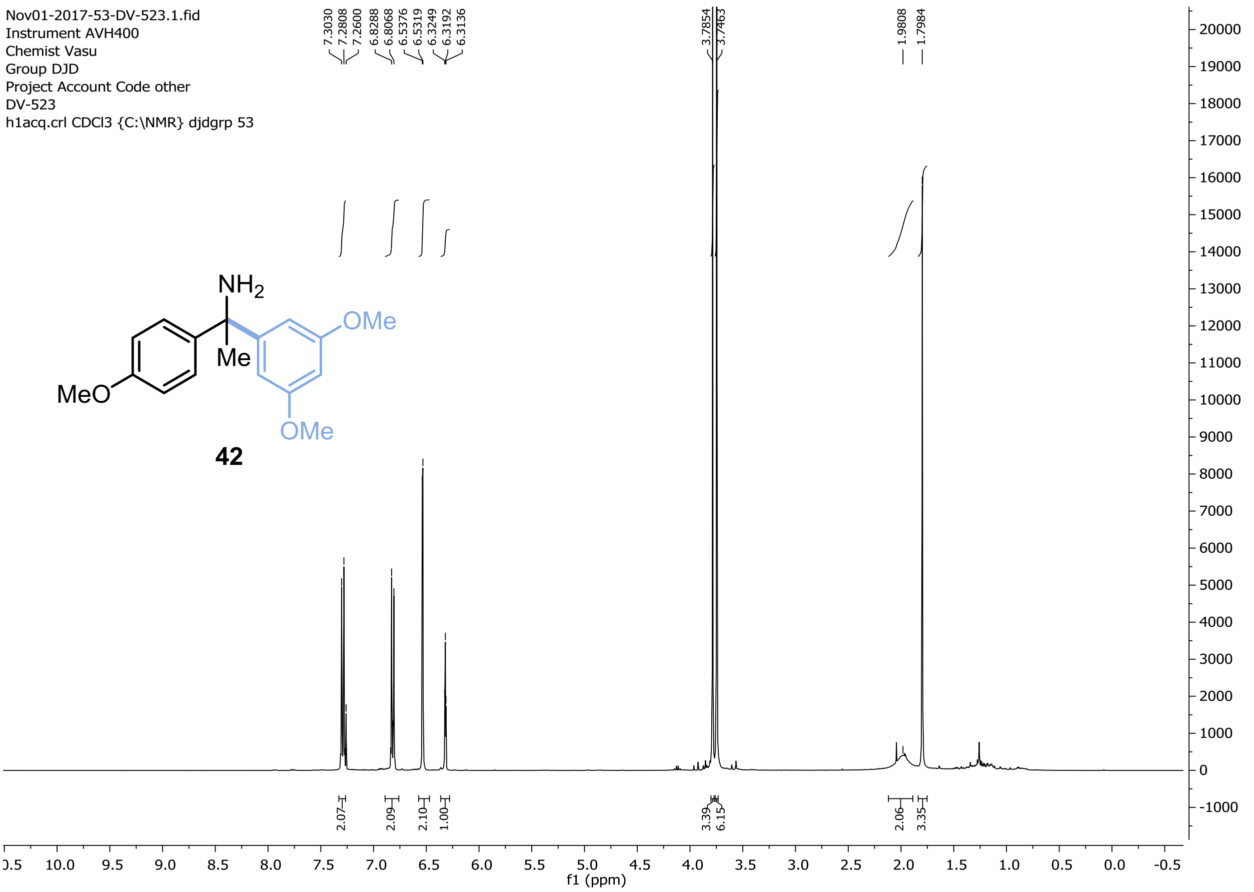
Nov01-2017-53-DV-523.1.fid
Instrument AVH400
Chemist Vasu
Group DJD
Project Account Code other
DV-523
h1acq.crl CDCl3 {C:\NMR} djdgrp 53
7.3030
7.2808
7.2600
6.8288
6.8068
6.5376
6.5319
6.3249
6.3192
6.3136
3.7854
3.7463
1.9808
1.7984
NH2
OMe
Me
MeO
OMe
42
2.07
2.09
2.10
1.00
3.39
6.15
2.06
3.35
f1 (ppm)

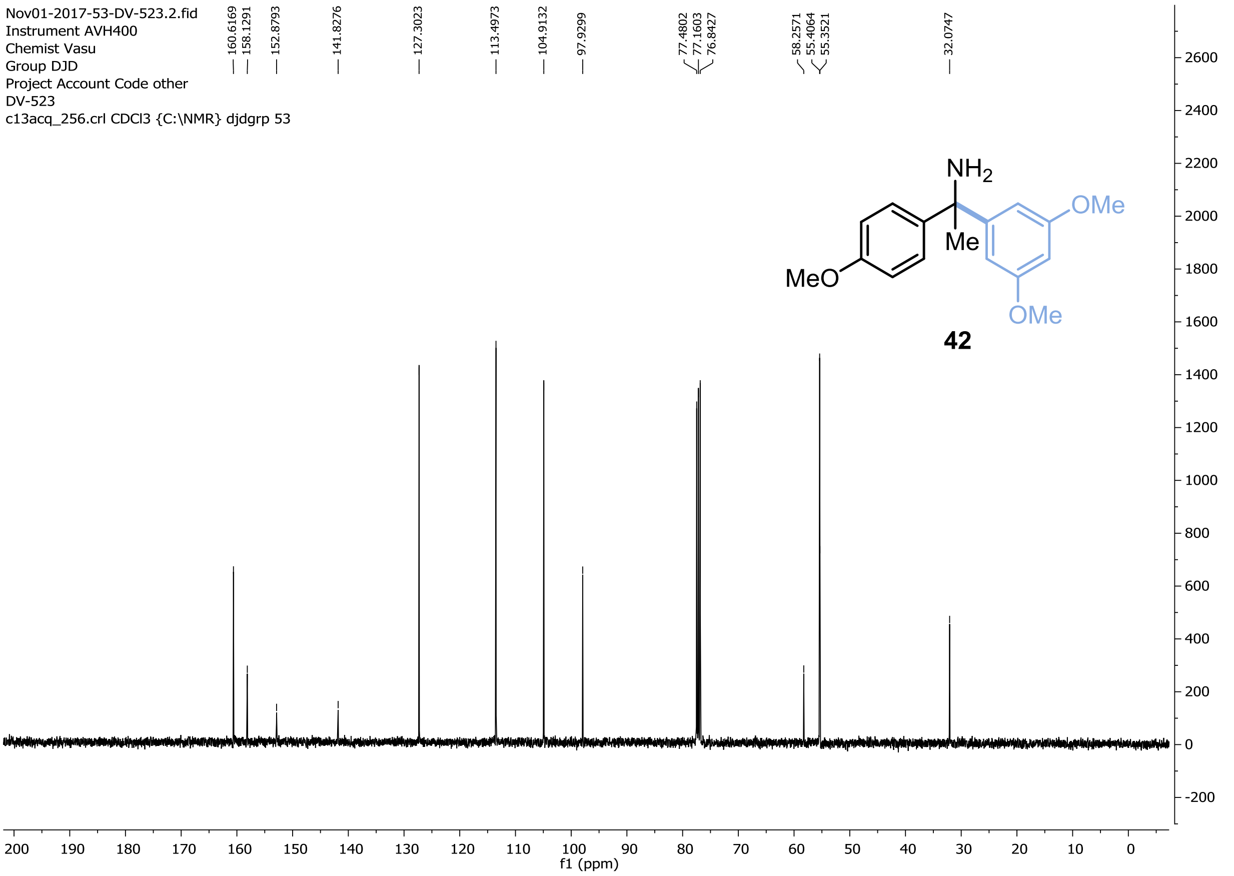
Nov01-2017-53-DV-523.2.fid
Instrument AVH400
Chemist Vasu
Group DJD
Project Account Code other
DV-523
c13acq_256.crl CDCl3 {C:\NMR} djdgrp 53
160.6169
158.1291
152.8793
141.8276
127.3023
113.4973
104.9132
97.9299
77.4802
77.1603
76.8427
58.2571
55.4064
55.3521
32.0747
NH2
OMe
Me
MeO
OMe
42
f1 (ppm)

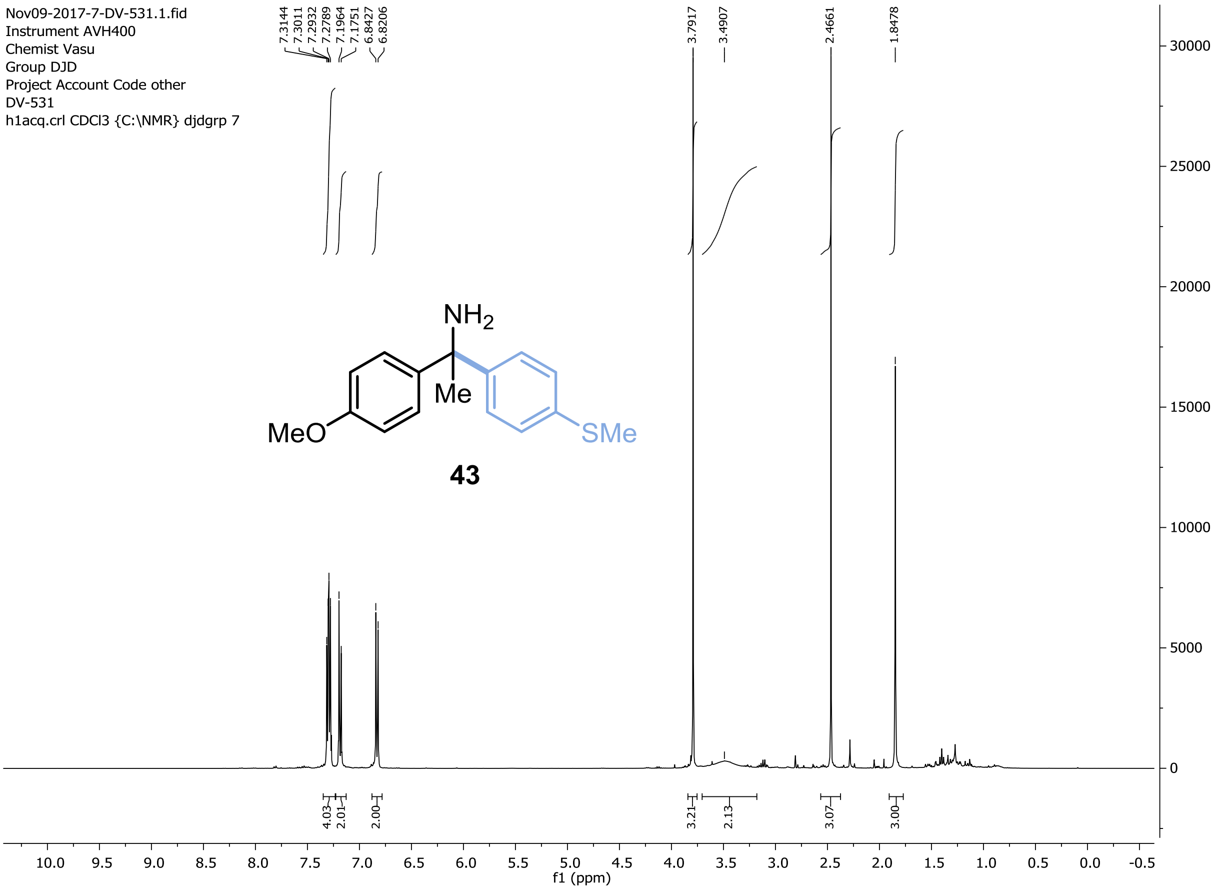
Nov09-2017-7-DV-531.1.fid
Instrument AVH400
Chemist Vasu
Group DJD
Project Account Code other
DV-531
h1acq.crl CDCl3 {C:\NMR} djdgrp 7
7.3144
7.3011
7.2932
7.2789
7.1964
7.1751
6.8427
6.8206
3.7917
3.4907
2.4661
1.8478
NH2
Me
MeO
SMe
43
4.03
2.01
2.00
3.21
2.13
3.07
3.00
f1 (ppm)

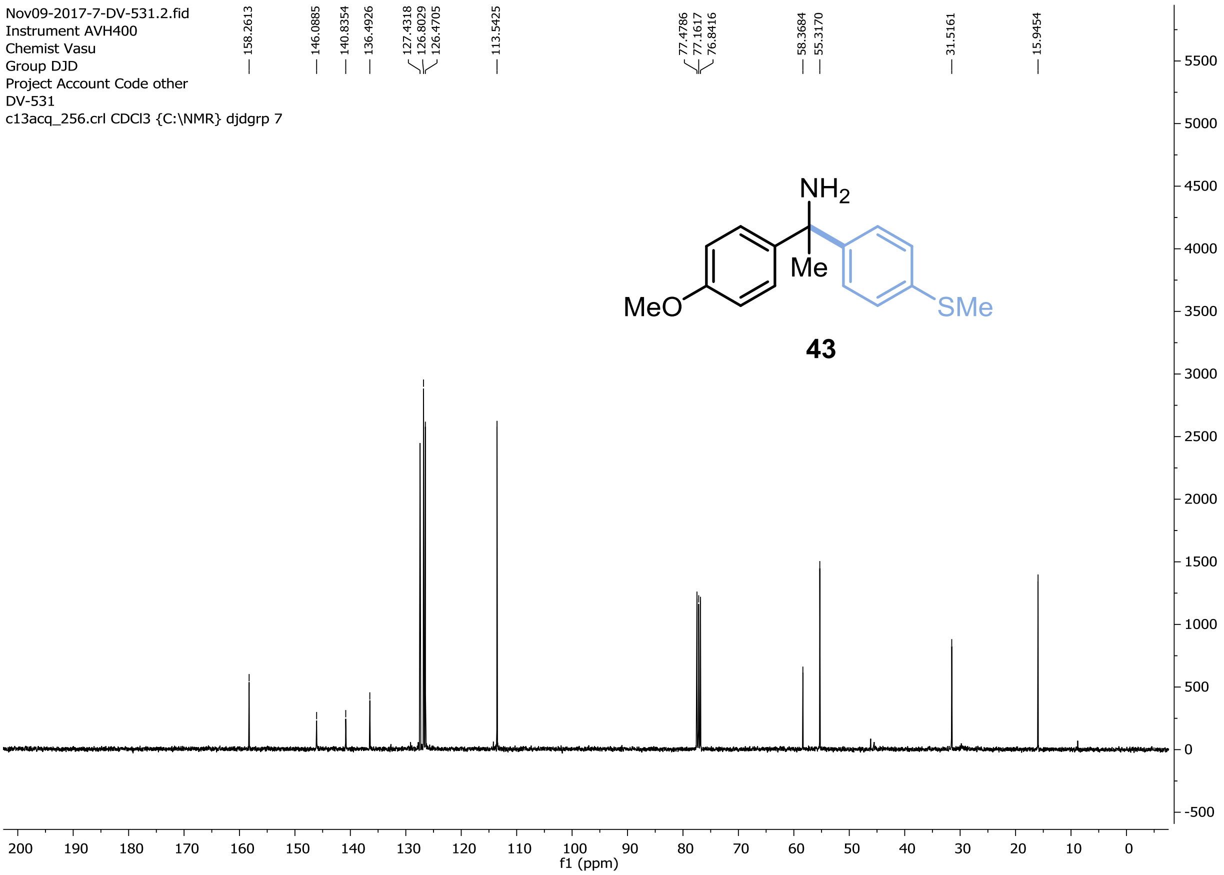
Nov09-2017-7-DV-531.2.fid
Instrument AVH400
Chemist Vasu
Group DJD
Project Account Code other
DV-531
c13acq_256.crl CDCl3 {C:\NMR} djdgrp 7
158.2613
146.0885
140.8354
136.4926
127.4318
126.8029
126.4705
113.5425
77.4786
77.1617
76.8416
58.3684
55.3170
31.5161
15.9454
NH2
Me
MeO
SMe
43
f1 (ppm)

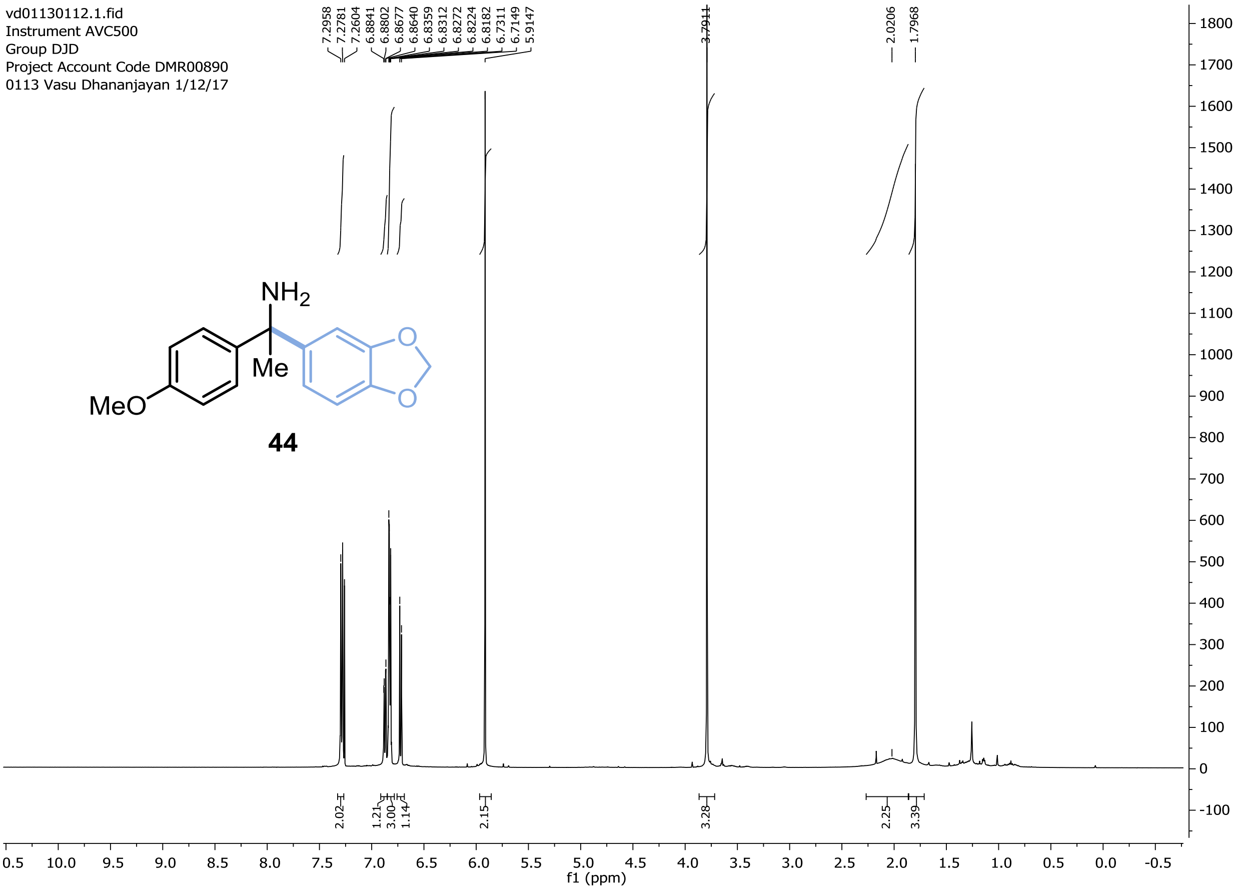
vd01130112.1.fid
Instrument AVC500
Group DJD
Project Account Code DMR00890
0113 Vasu Dhananjayan 1/12/17
NH2
Me
MeO
44
f1 (ppm)

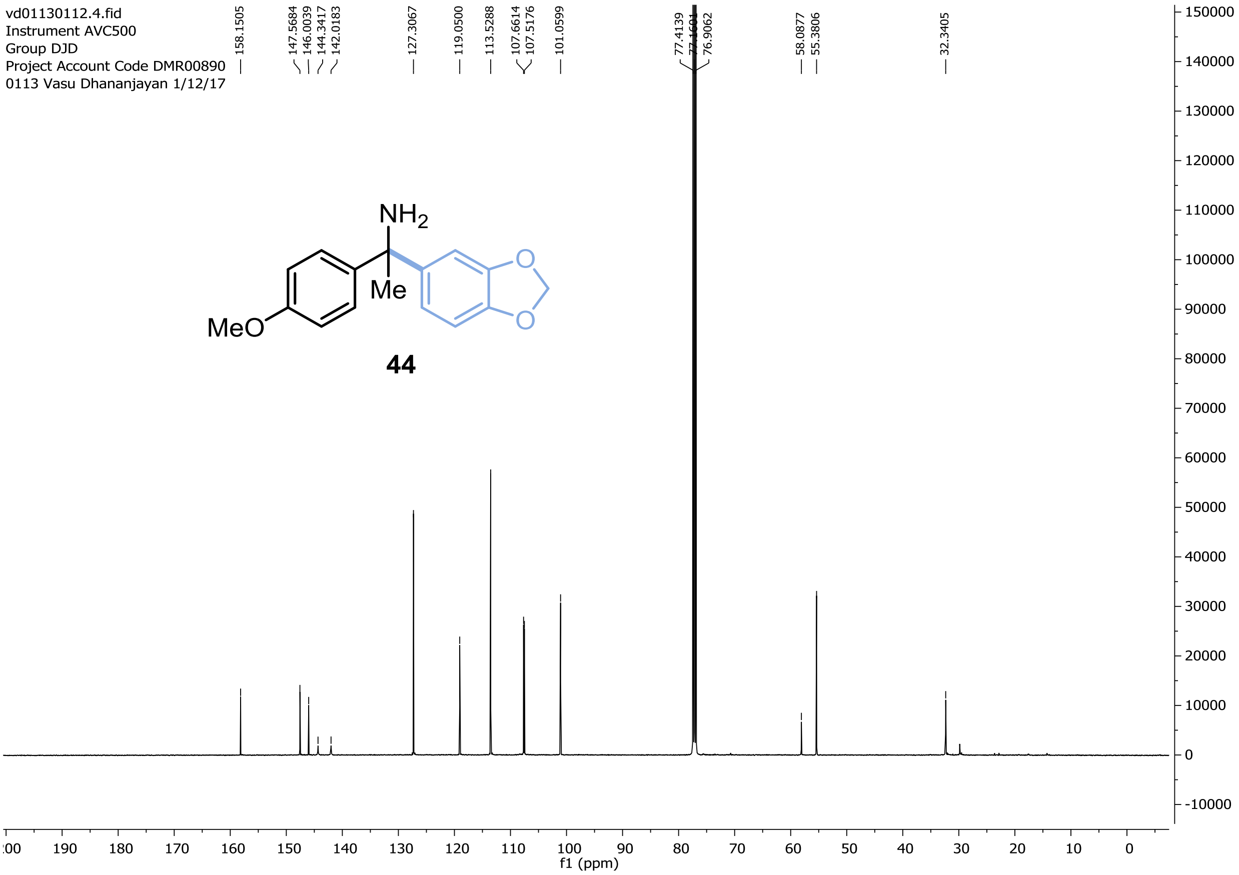
vd01130112.4.fid
Instrument AVC500
Group DJD
Project Account Code DMR00890
0113 Vasu Dhananjayan 1/12/17
NH2
Me
MeO
44
f1 (ppm)

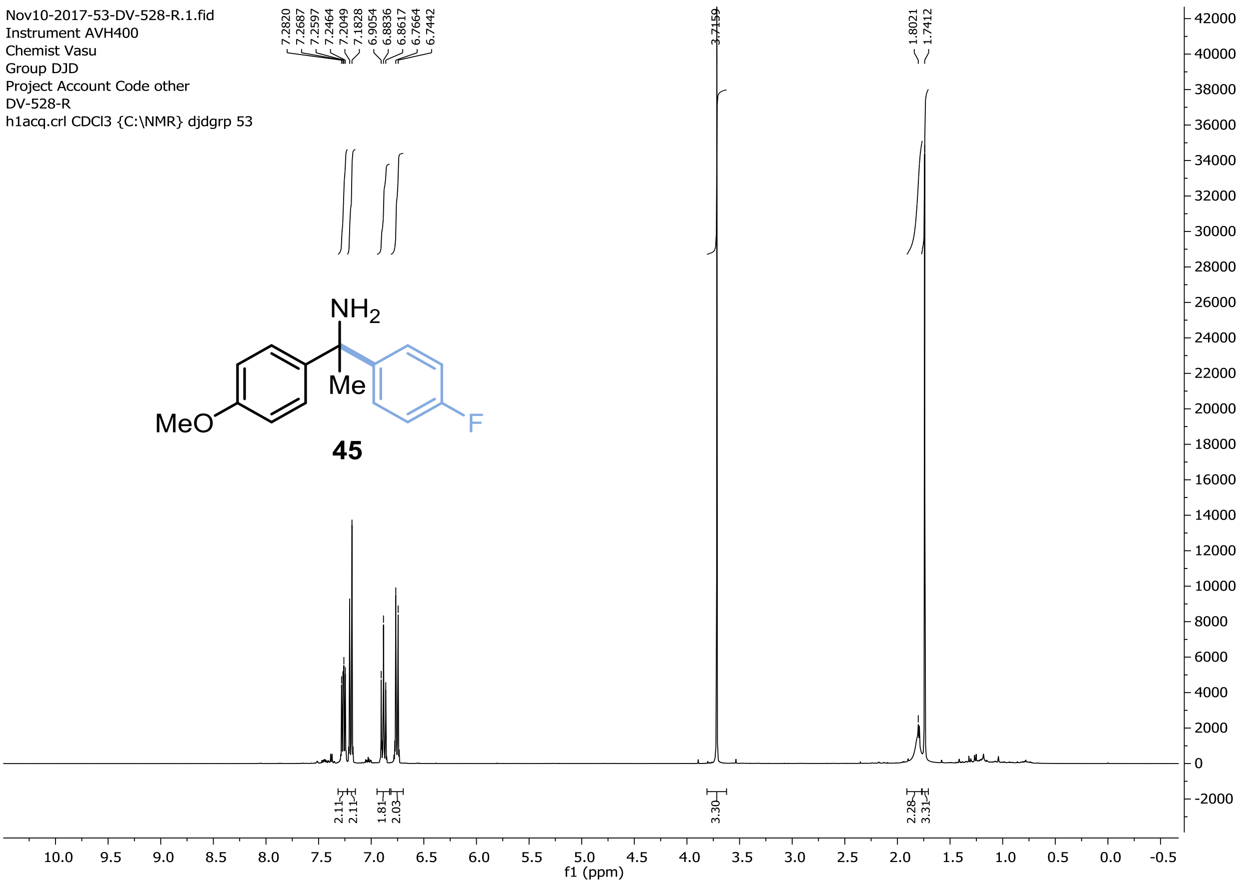
Nov10-2017-53-DV-528-R.1.fid
Instrument AVH400
Chemist Vasu
Group DJD
Project Account Code other
DV-528-R
h1acq.crl CDCl3 {C:\NMR} djdgrp 53
7.2820
7.2687
7.2597
7.2464
7.2049
7.1828
6.9054
6.8836
6.8617
6.7664
6.7442
3.7159
1.8021
1.7412
NH2
Me
MeO
F
45
2.11
2.11
1.81
2.03
3.30
2.28
3.31
f1 (ppm)

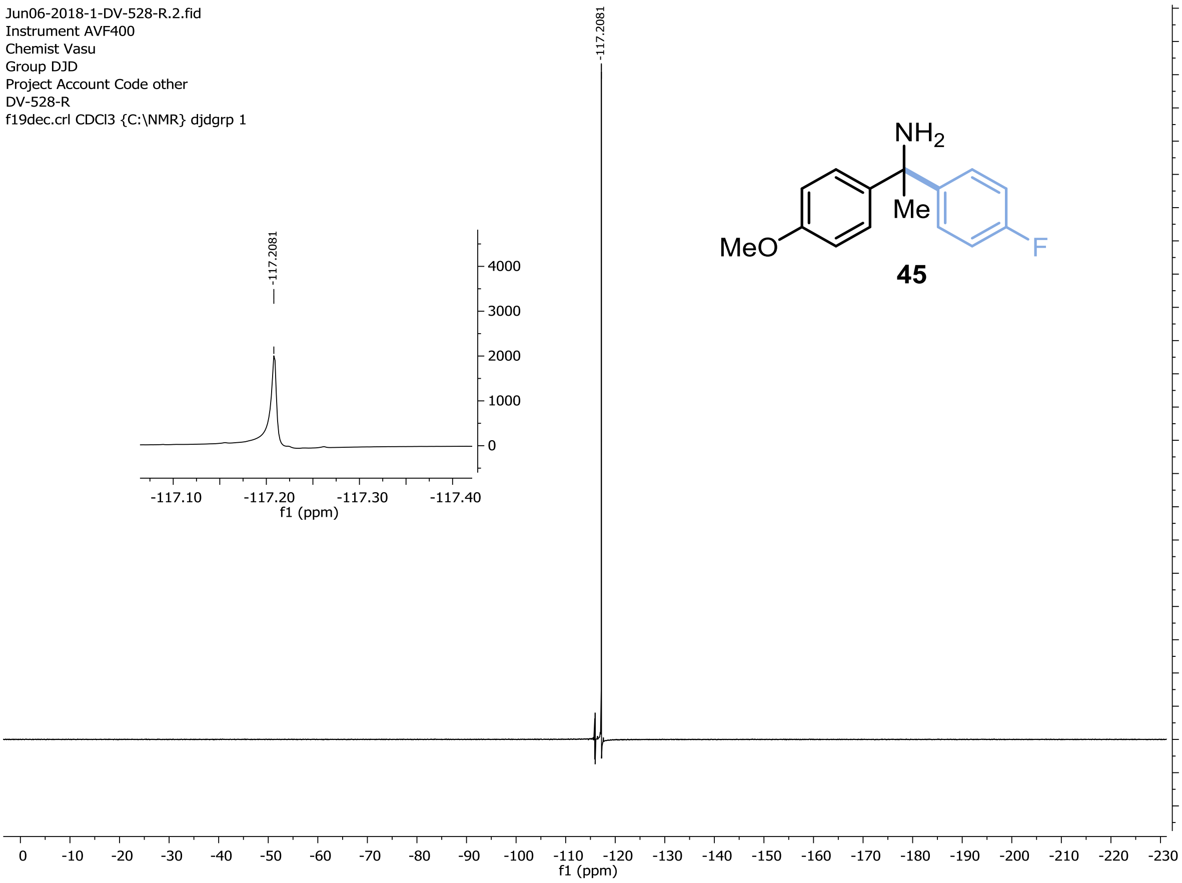
Jun06-2018-1-DV-528-R.2.fid
Instrument AVF400
Chemist Vasu
Group DJD
Project Account Code other
DV-528-R
f19dec.crl CDCl3 {C:\NMR} djdgrp 1
-117.2081
-117.2081
NH2
Me
MeO
F
45
f1 (ppm)
f1 (ppm)

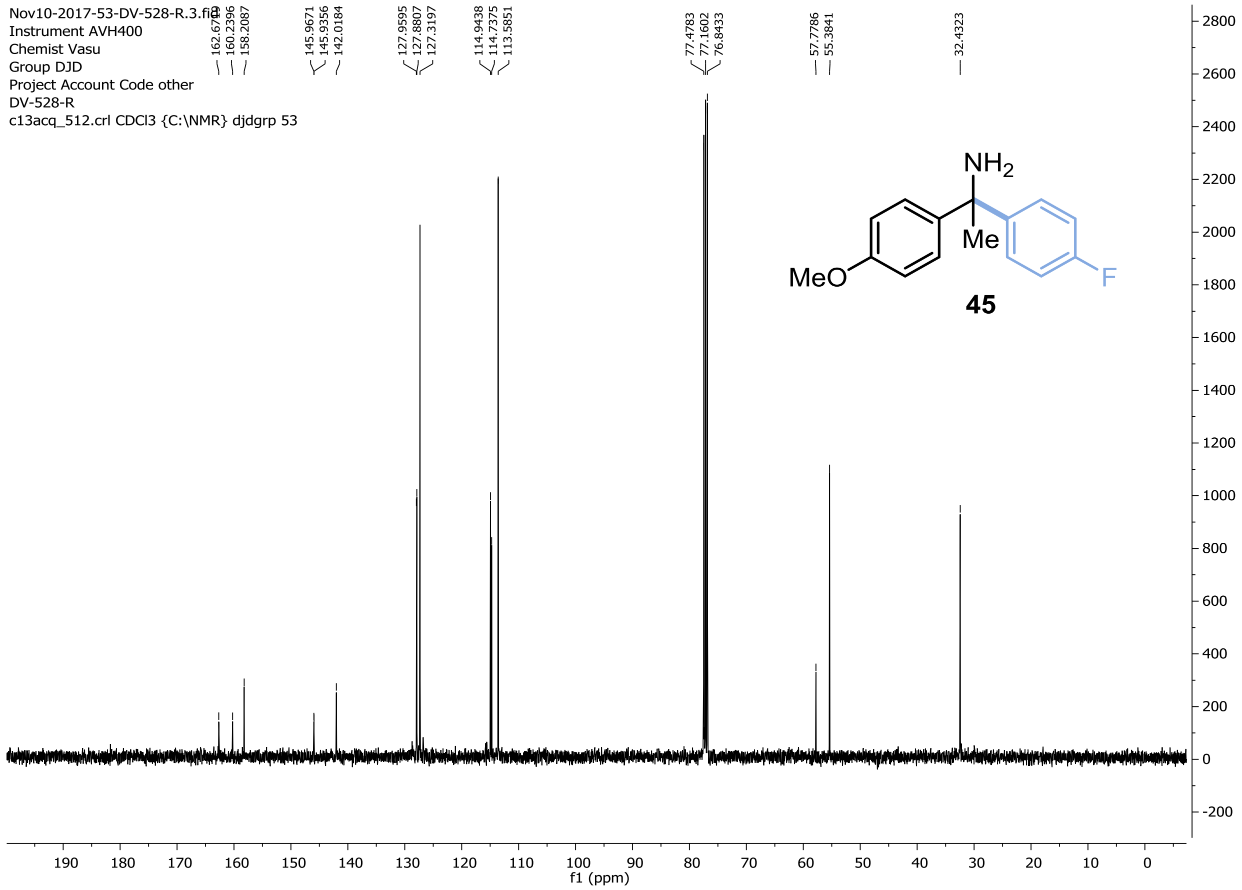
Nov10-2017-53-DV-528-R.3.fid
Instrument AVH400
Chemist Vasu
Group DJD
Project Account Code other
DV-528-R
c13acq_512.crl CDCl3 {C:\NMR} djdgrp 53
162.6719
160.2396
158.2087
145.9671
145.9356
142.0184
127.9595
127.8807
127.3197
114.9438
114.7375
113.5851
77.4783
77.1602
76.8433
57.7786
55.3841
32.4323
NH2
Me
MeO
F
f1 (ppm)

45

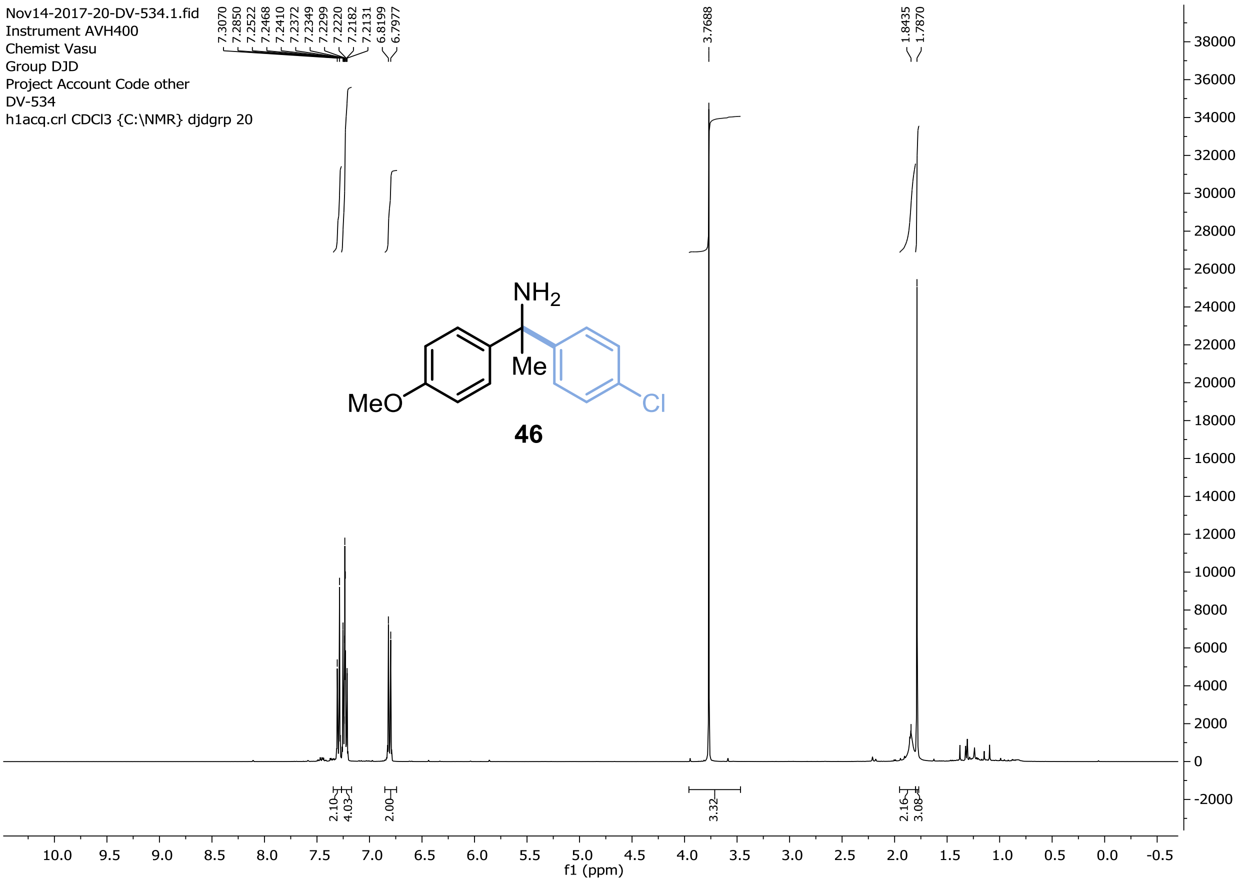
Nov14-2017-20-DV-534.1.fid
Instrument AVH400
Chemist Vasu
Group DJD
Project Account Code other
DV-534
h1acq.crl CDCl3 {C:\NMR} djdgrp 20
7.3070
7.2850
7.2522
7.2468
7.2410
7.2372
7.2349
7.2299
7.2220
7.2182
7.2131
6.8199
6.7977
3.7688
1.8435
1.7870
NH2
Me
MeO
Cl
46
2.10
4.03
2.00
3.32
2.16
3.08
f1 (ppm)

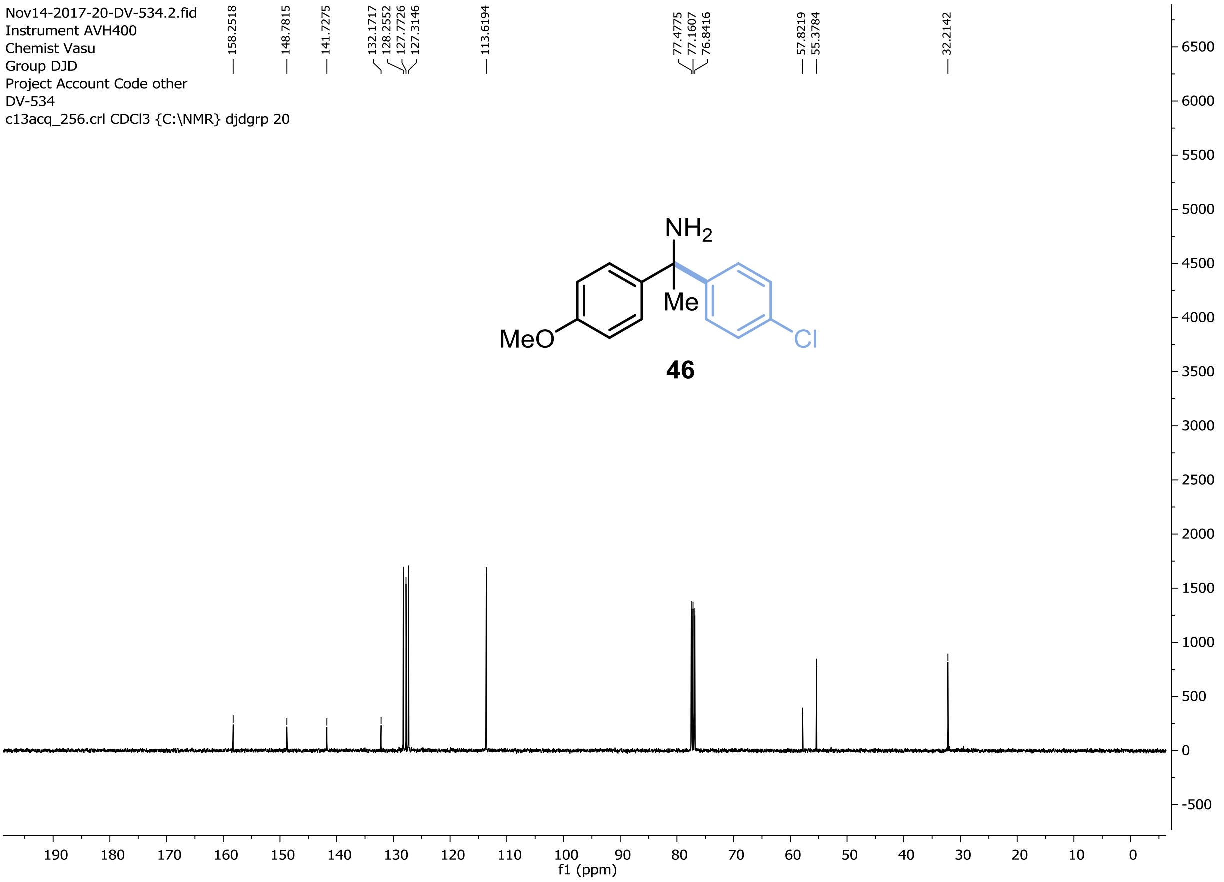
Nov14-2017-20-DV-534.2.fid
Instrument AVH400
Chemist Vasu
Group DJD
Project Account Code other
DV-534
c13acq_256.crl CDCl3 {C:\NMR} djdgrp 20
158.2518
148.7815
141.7275
132.1717
128.2552
127.7726
127.3146
113.6194
77.4775
77.1607
76.8416
57.8219
55.3784
32.2142
NH2
Me
MeO
Cl
46
f1 (ppm)

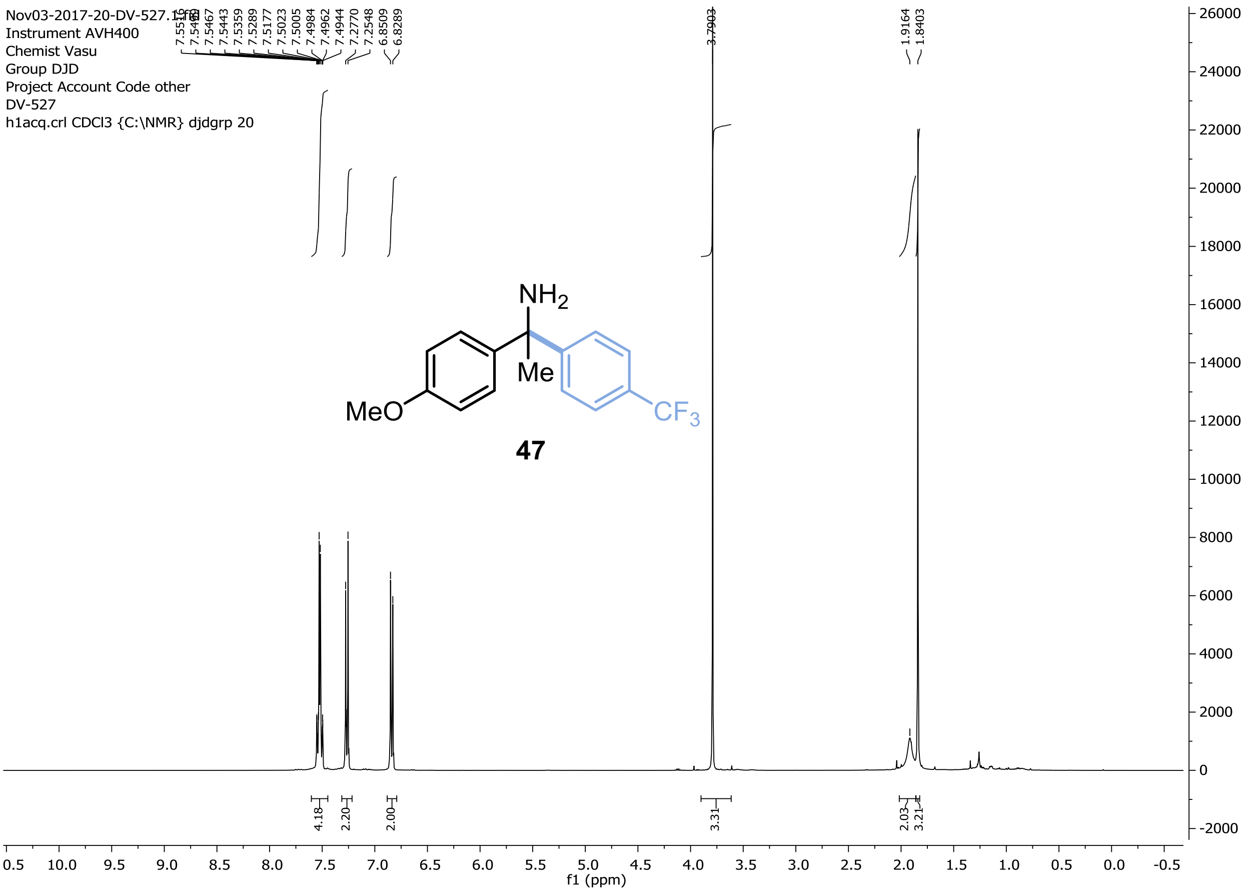
Nov03-2017-20-DV-527.1.fid
Instrument AVH400
Chemist Vasu
Group DJD
Project Account Code other
DV-527
h1acq.crl CDCl3 {C:\NMR} djdgrp 20
3.7903
1.9164
1.8403
4.18
2.20
2.00
3.31
2.03
3.21
NH2
Me
MeO
CF3
47
f1 (ppm)

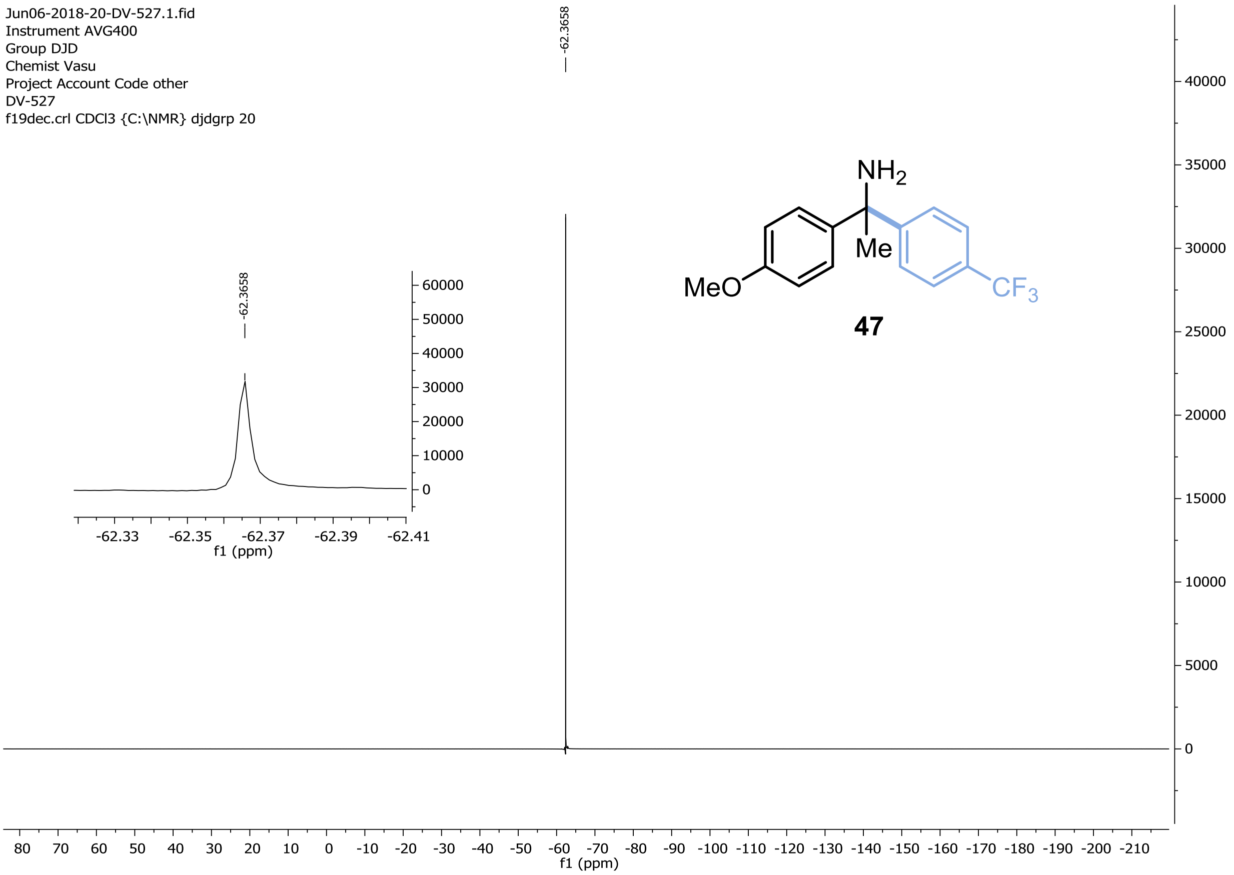
Jun06-2018-20-DV-527.1.fid
Instrument AVG400
Group DJD
Chemist Vasu
Project Account Code other
DV-527
f19dec.crl CDCl3 {C:\NMR} djdgrp 20
-62.3658
NH2
Me
MeO
CF3
47
f1 (ppm)

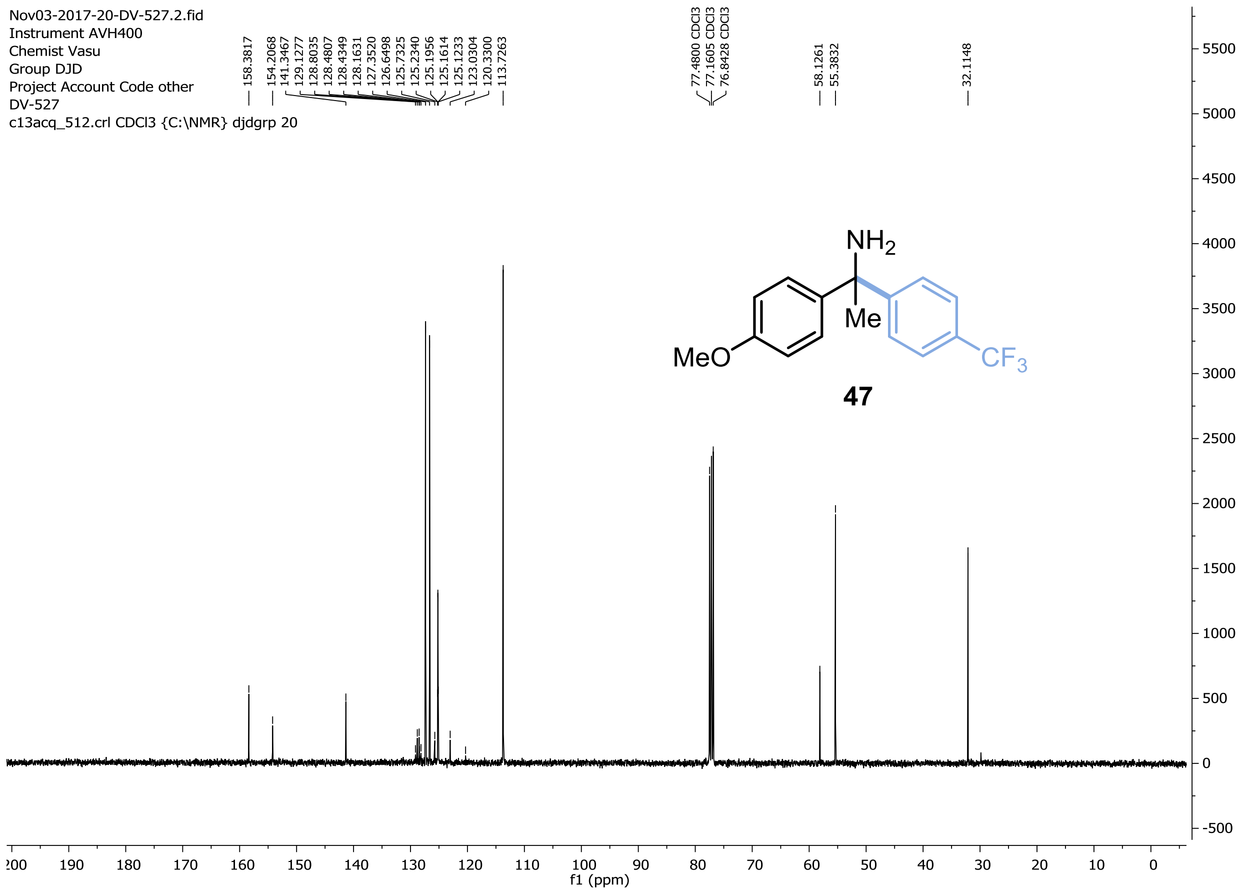

Nov03-2017-20-DV-527.2.fid
Instrument AVH400
Chemist Vasu
Group DJD
Project Account Code other
DV-527
c13acq_512.crl CDCl3 {C:\NMR} djdgrp 20
158.3817
154.2068
141.3467
129.1277
128.8035
128.4807
128.4349
128.1631
127.3520
126.6498
125.7325
125.2340
125.1956
125.1614
125.1233
123.0304
120.3300
113.7263
77.4800 CDCl3
77.1605 CDCl3
76.8428 CDCl3
58.1261
55.3832
32.1148
NH2
Me
MeO
CF3
47
f1 (ppm)

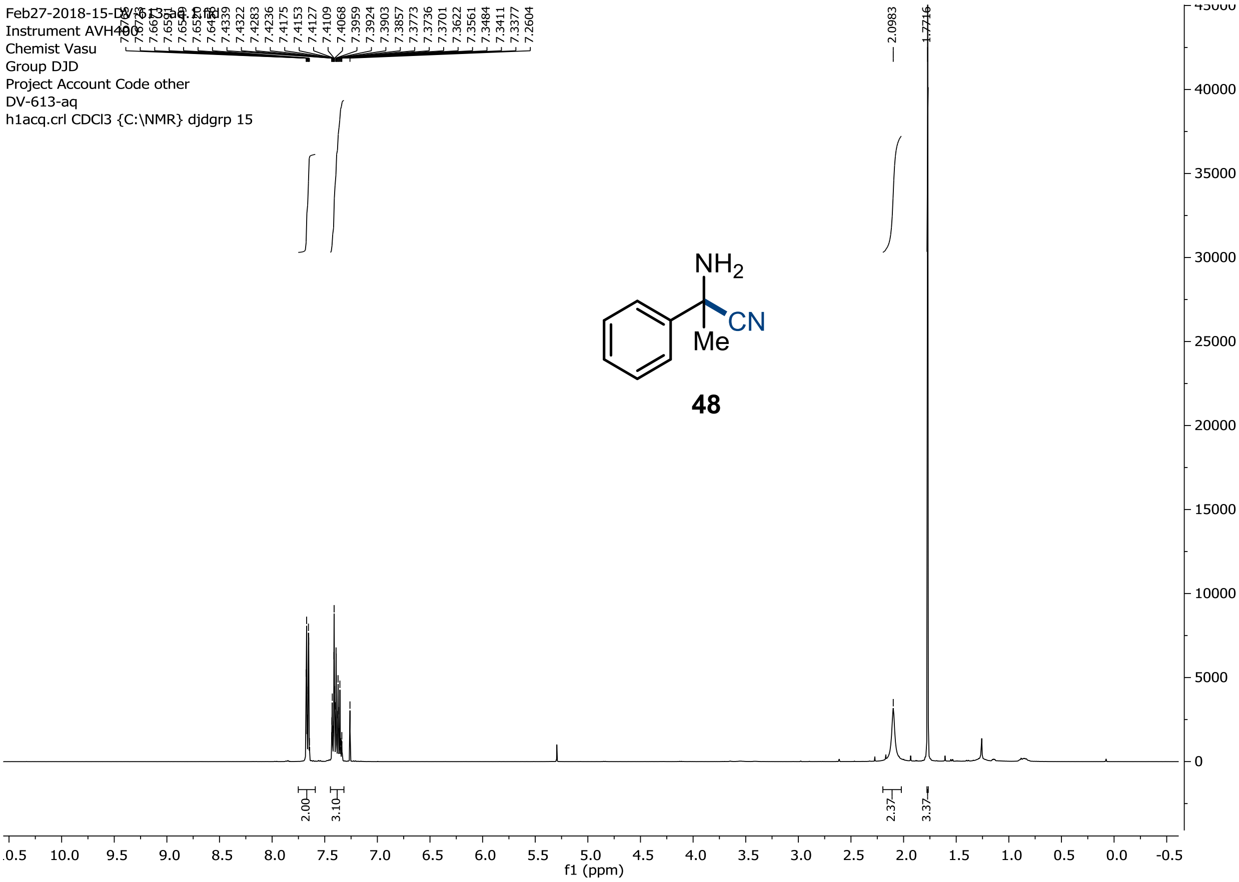

Instrument AVH400
Chemist Vasu
Group DJD
Project Account Code other
DV-613-aq
h1acq.crl CDCl3 {C:\NMR} djdgrp 15
7.4339
7.4322
7.4283
7.4236
7.4175
7.4153
7.4127
7.4109
7.4068
7.3959
7.3924
7.3903
7.3857
7.3773
7.3736
7.3701
7.3622
7.3561
7.3484
7.3411
7.3377
7.2604
2.0983
1.7716
NH2
CN
Me
48
2.00
3.10
2.37
3.37
f1 (ppm)

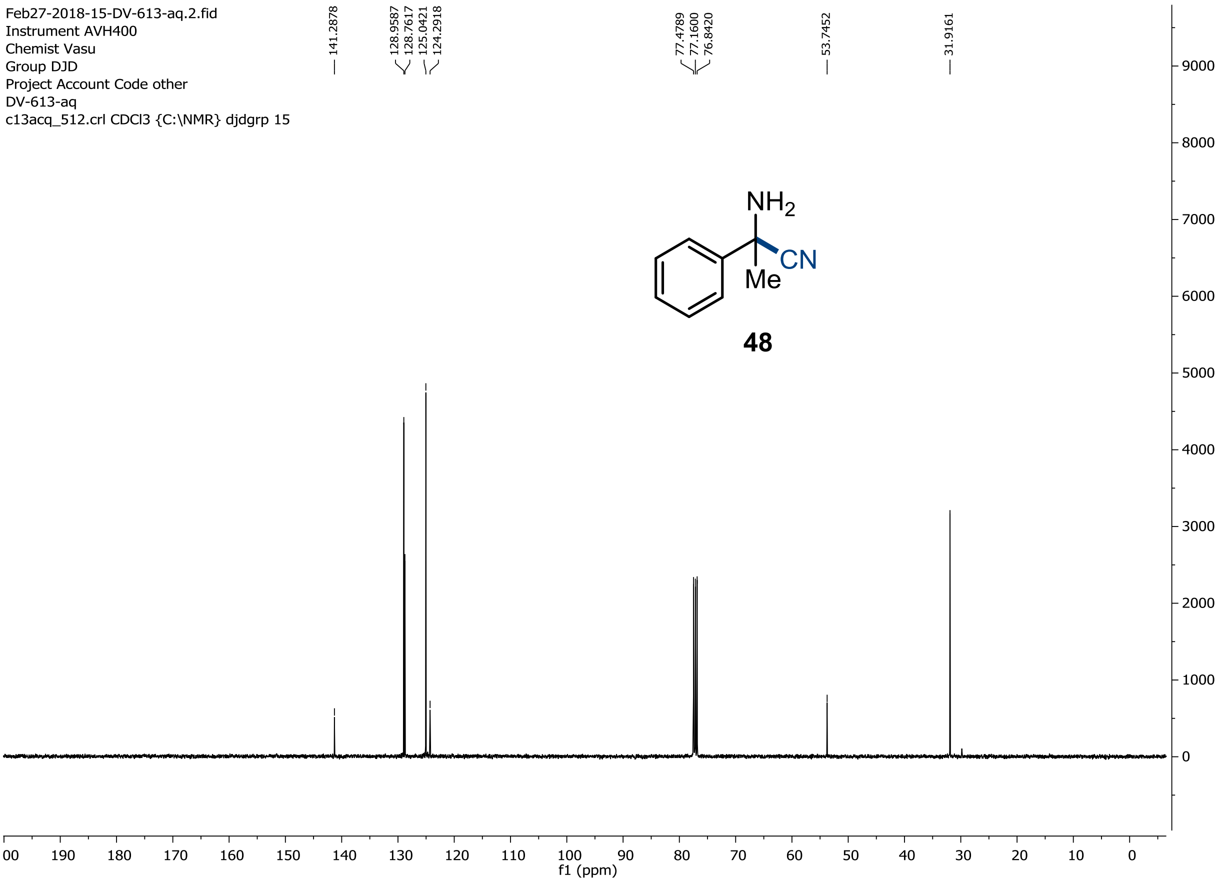

Feb27-2018-15-DV-613-aq.2.fid
Instrument AVH400
Chemist Vasu
Group DJD
Project Account Code other
DV-613-aq
c13acq_512.crl CDCl3 {C:\NMR} djdgrp 15
141.2878
128.9587
128.7617
125.0421
124.2918
77.4789
77.1600
76.8420
53.7452
31.9161
NH2
CN
Me
48
f1 (ppm)

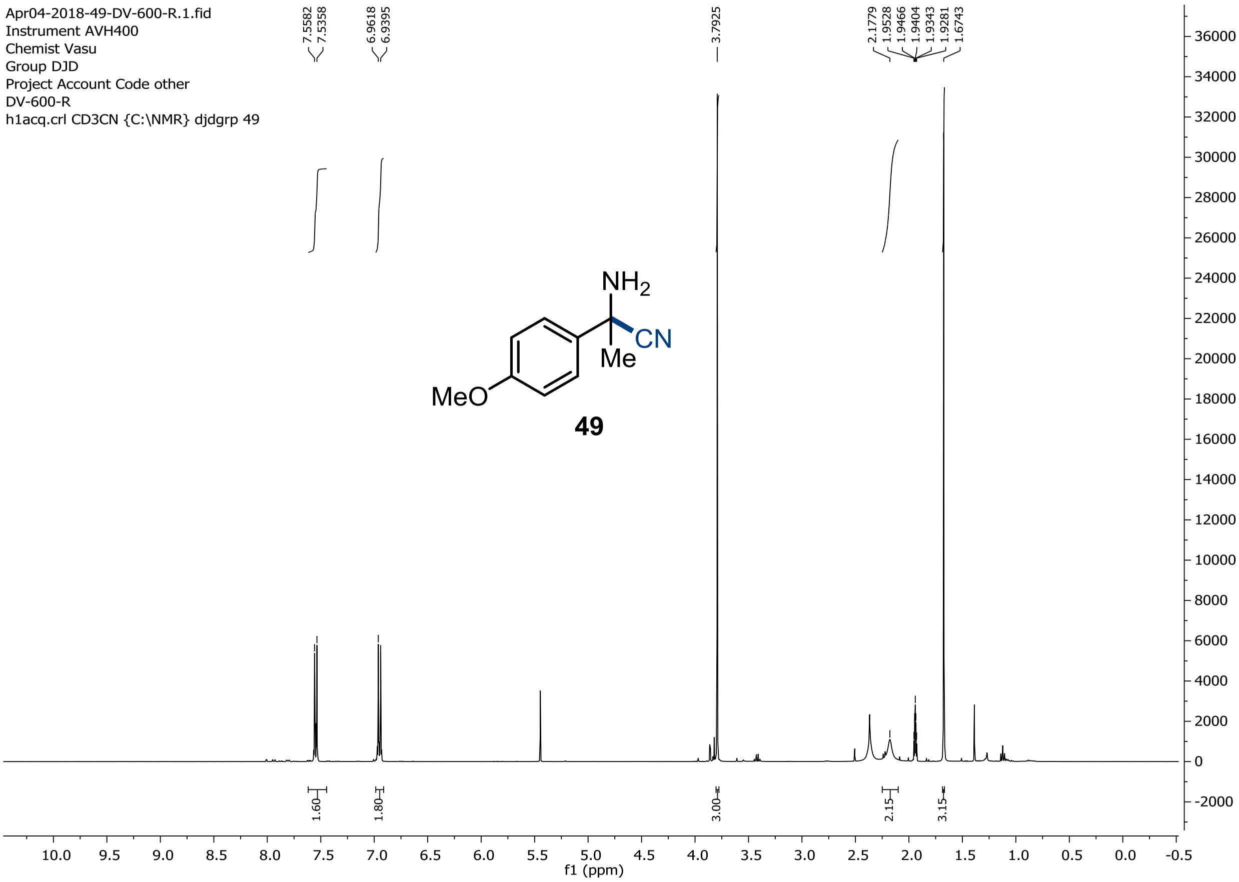
Apr04-2018-49-DV-600-R.1.fid
Instrument AVH400
Chemist Vasu
Group DJD
Project Account Code other
DV-600-R
h1acq.crl CD3CN {C:\NMR} djdgrp 49
7.5582
7.5358
6.9618
6.9395
3.7925
2.1779
1.9528
1.9466
1.9404
1.9343
1.9281
1.6743
NH2
CN
Me
MeO
49
1.60
1.80
3.00
2.15
3.15
f1 (ppm)

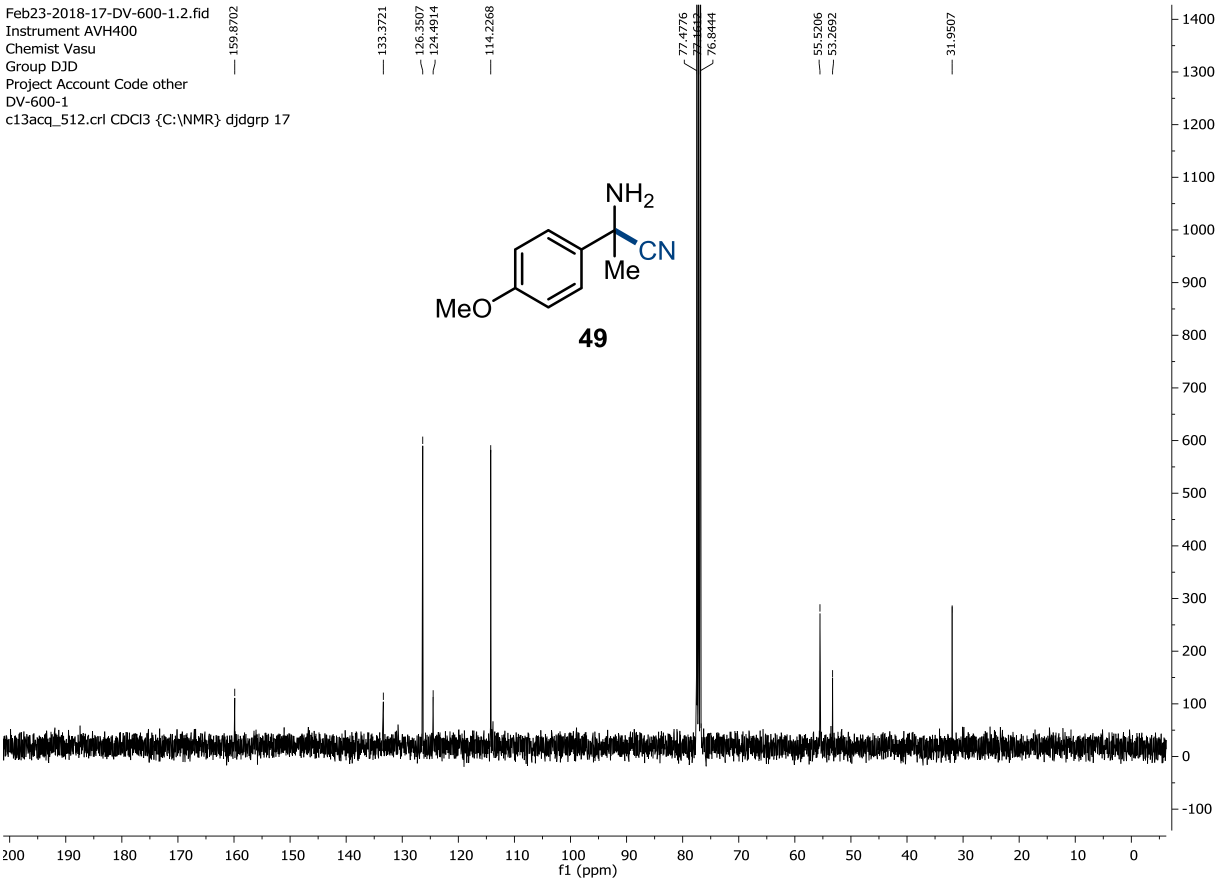
Feb23-2018-17-DV-600-1.2.fid
Instrument AVH400
Chemist Vasu
Group DJD
Project Account Code other
DV-600-1
c13acq_512.crl CDCl3 {C:\NMR} djdgrp 17
159.8702
133.3721
126.3507
124.4914
114.2268
77.4776
76.8444
55.5206
53.2692
31.9507
NH2
CN
Me
MeO
49
f1 (ppm)

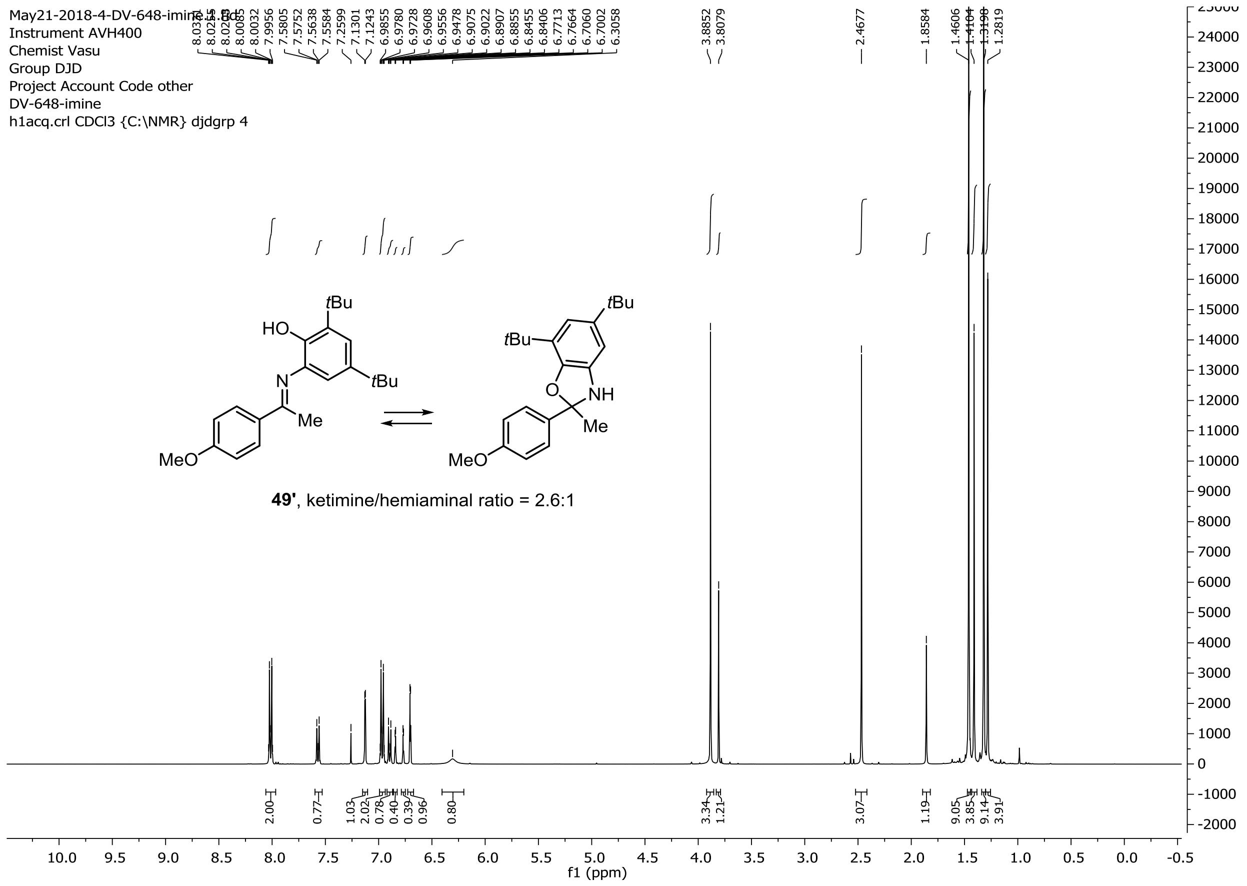
May21-2018-4-DV-648-imine
Instrument AVH400
Chemist Vasu
Group DJD
Project Account Code other
DV-648-imine
h1acq.crl CDCl3 {C:\NMR} djdgrp 4
49', ketimine/hemiaminal ratio = 2.6:1
f1 (ppm)

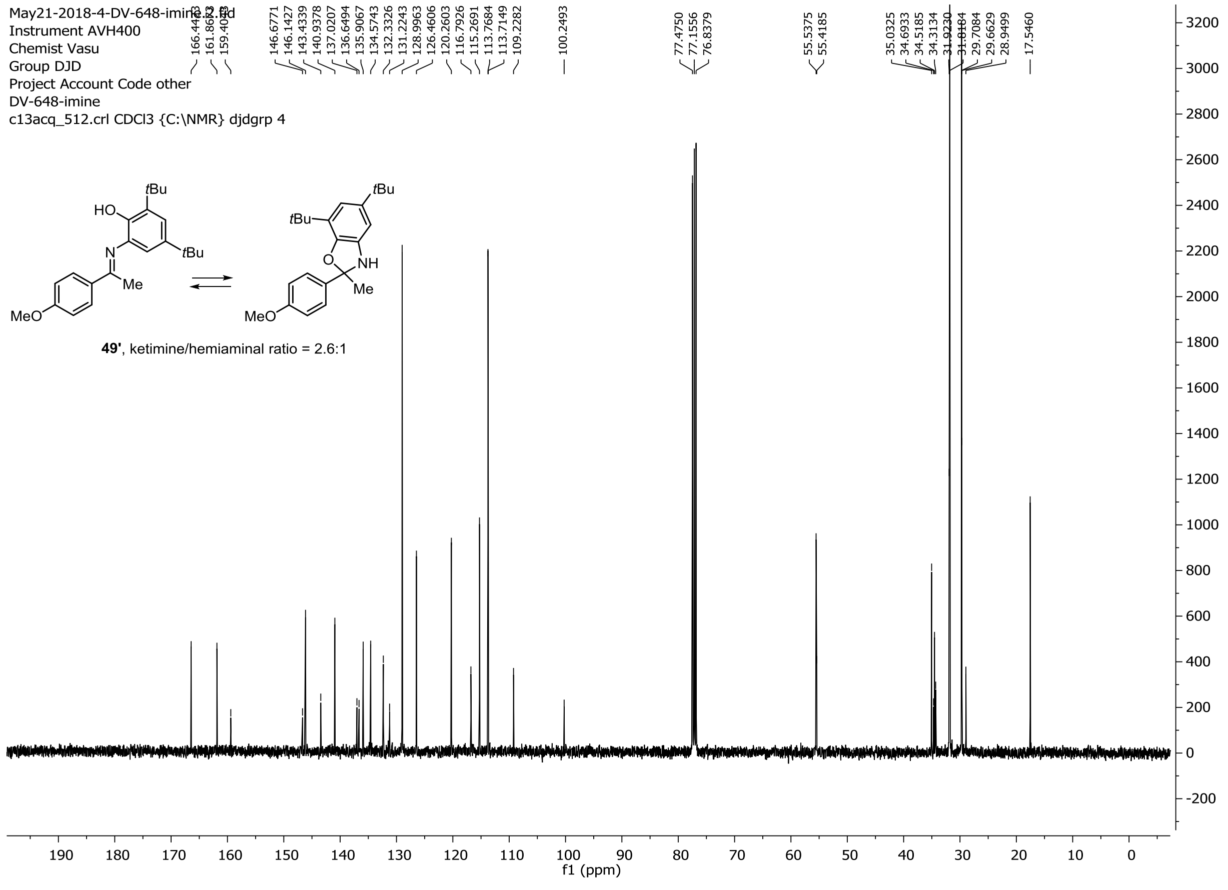
May21-2018-4-DV-648-imine
Instrument AVH400
Chemist Vasu
Group DJD
Project Account Code other
DV-648-imine
c13acq_512.crl CDCl3 {C:\NMR} djdgrp 4
49', ketimine/hemiaminal ratio = 2.6:1
f1 (ppm)

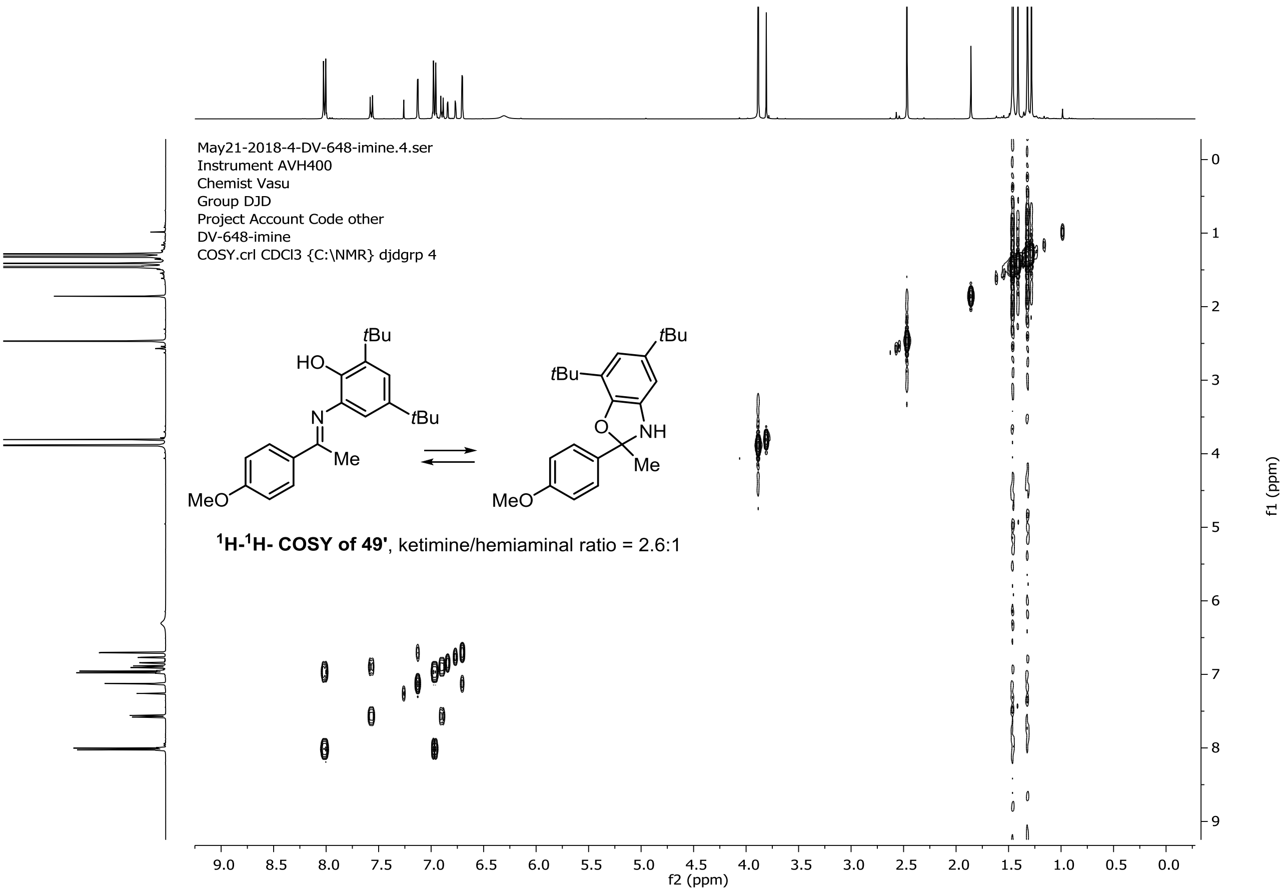
May21-2018-4-DV-648-imine.4.ser
Instrument AVH400
Chemist Vasu
Group DJD
Project Account Code other
DV-648-imine
COSY.crl CDCl3 {C:\NMR} djdgrp 4
^{1}H-^{1}H- COSY of 49', ketimine/hemiaminal ratio = 2.6:1
f2 (ppm)
f1 (ppm)

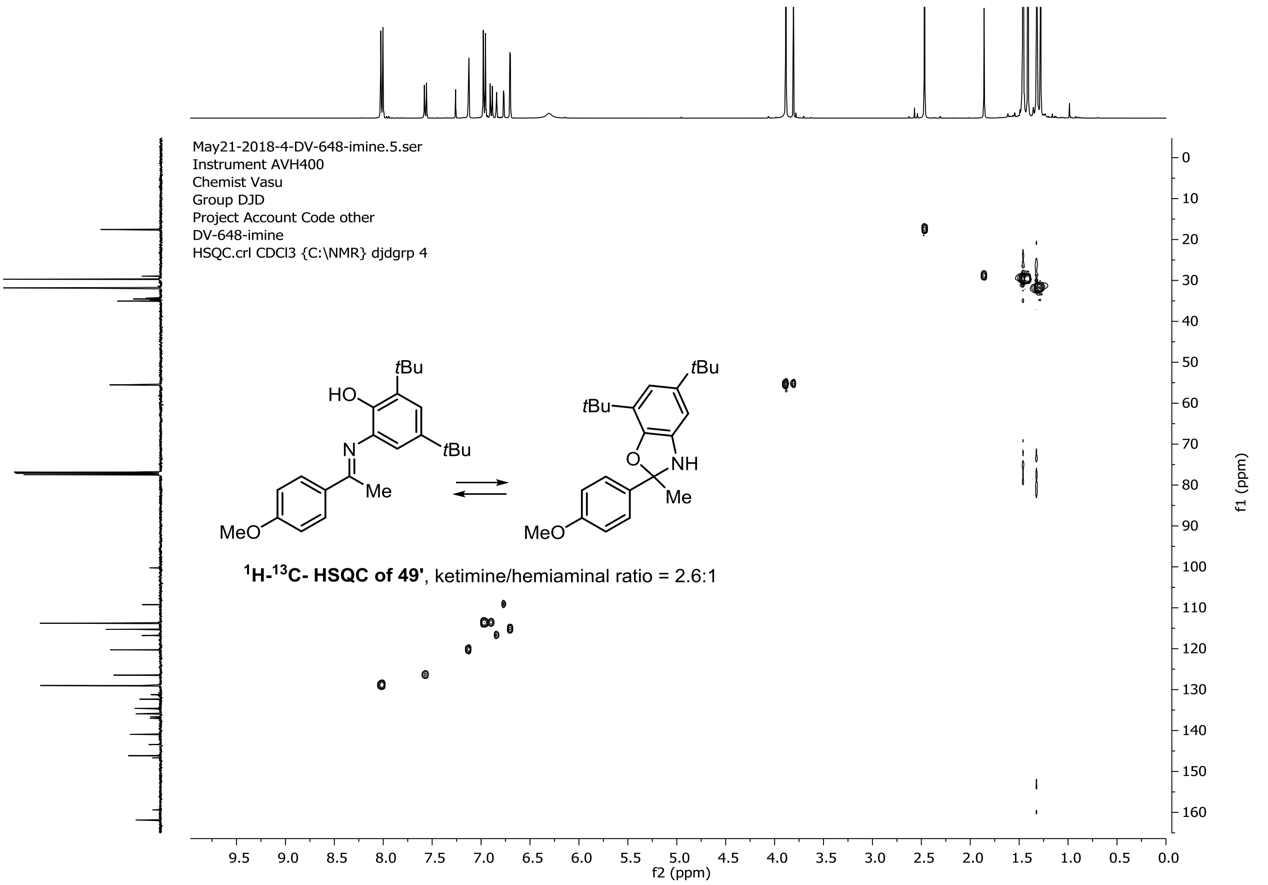
May21-2018-4-DV-648-imine.5.ser
Instrument AVH400
Chemist Vasu
Group DJD
Project Account Code other
DV-648-imine
HSQC.crl CDCl3 {C:\NMR} djdgrp 4
^{1}H-^{13}C- HSQC of 49', ketimine/hemiaminal ratio = 2.6:1
f2 (ppm)
f1 (ppm)

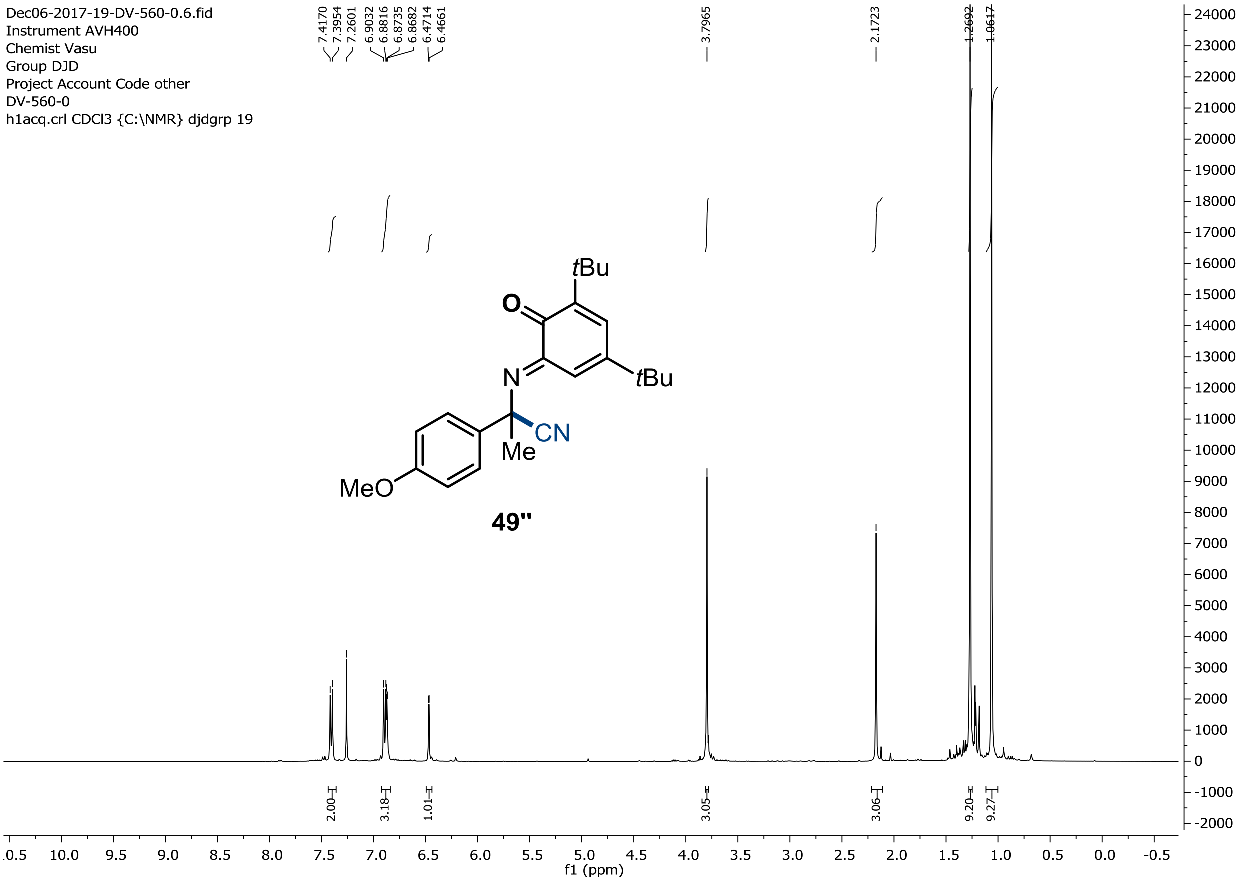
Dec06-2017-19-DV-560-0.6.fid
Instrument AVH400
Chemist Vasu
Group DJD
Project Account Code other
DV-560-0
h1acq.crl CDCl3 {C:\NMR} djdgrp 19
7.4170
7.3954
7.2601
6.9032
6.8816
6.8735
6.8682
6.4714
6.4661
3.7965
2.1723
1.0617
tBu
O
N
tBu
CN
Me
MeO
49''
2.00
3.18
1.01
3.05
3.06
9.20
9.27
f1 (ppm)

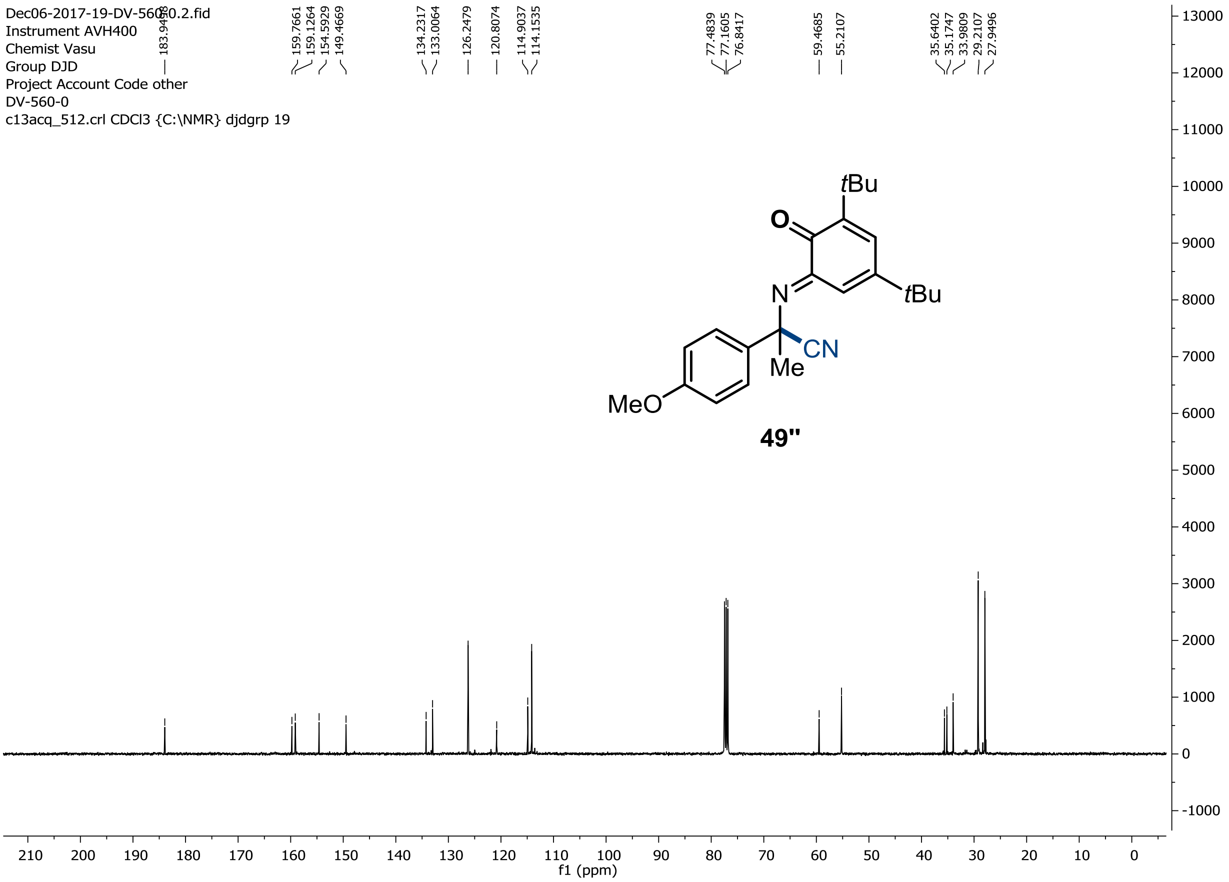
Instrument AVH400
Chemist Vasu
Group DJD
Project Account Code other
DV-560-0
c13acq_512.crl CDCl3 {C:\NMR} djdgrp 19
159.7661
159.1264
154.5929
149.4669
134.2317
133.0064
126.2479
120.8074
114.9037
114.1535
77.4839
77.1605
76.8417
59.4685
55.2107
35.6402
35.1747
33.9809
29.2107
27.9496
tBu
O
N
tBu
CN
Me
MeO
49''
f1 (ppm)

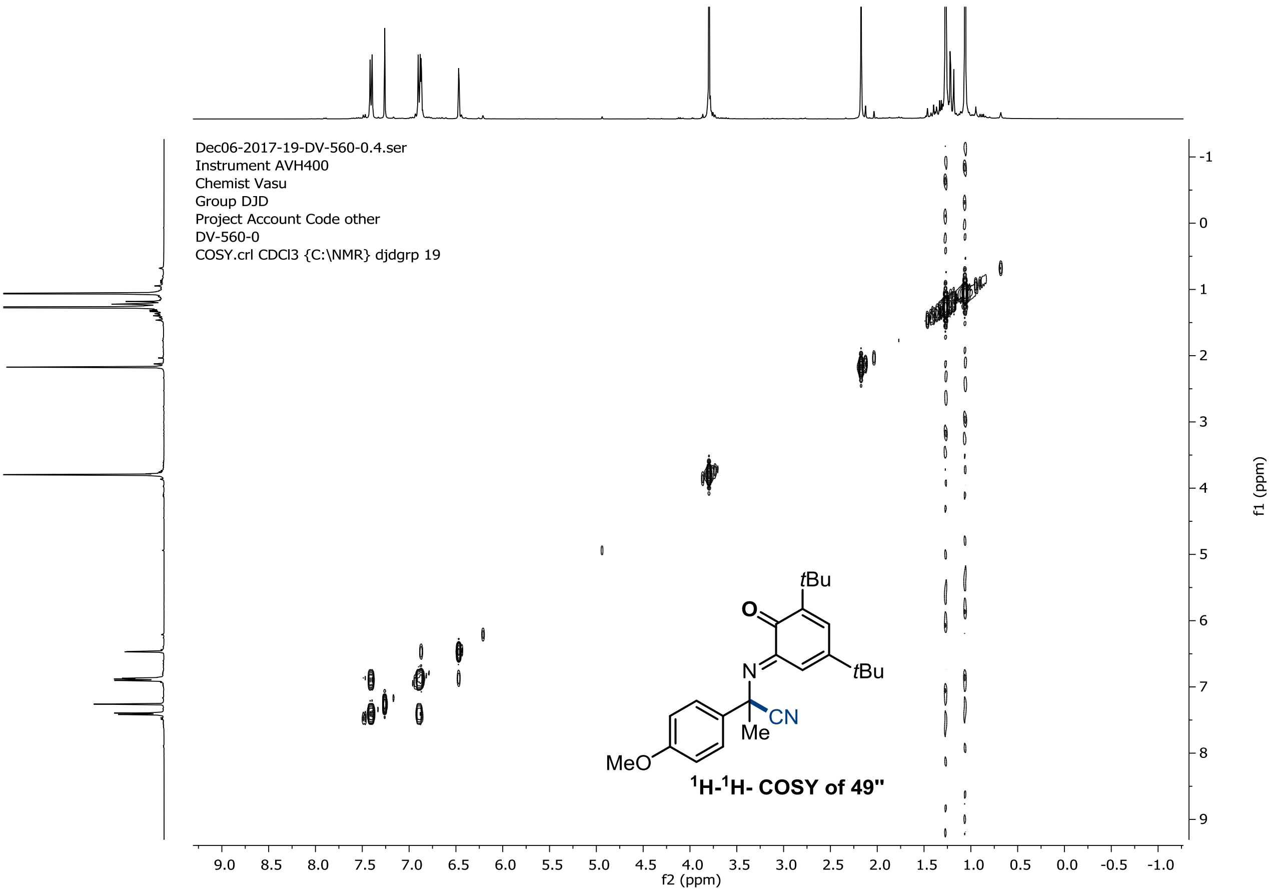
Dec06-2017-19-DV-560-0.4.ser
Instrument AVH400
Chemist Vasu
Group DJD
Project Account Code other
DV-560-0
COSY.crl CDCl3 {C:\NMR} djdgrp 19
tBu
O
N
tBu
CN
Me
MeO
1H-1H- COSY of 49''
f2 (ppm)
f1 (ppm)

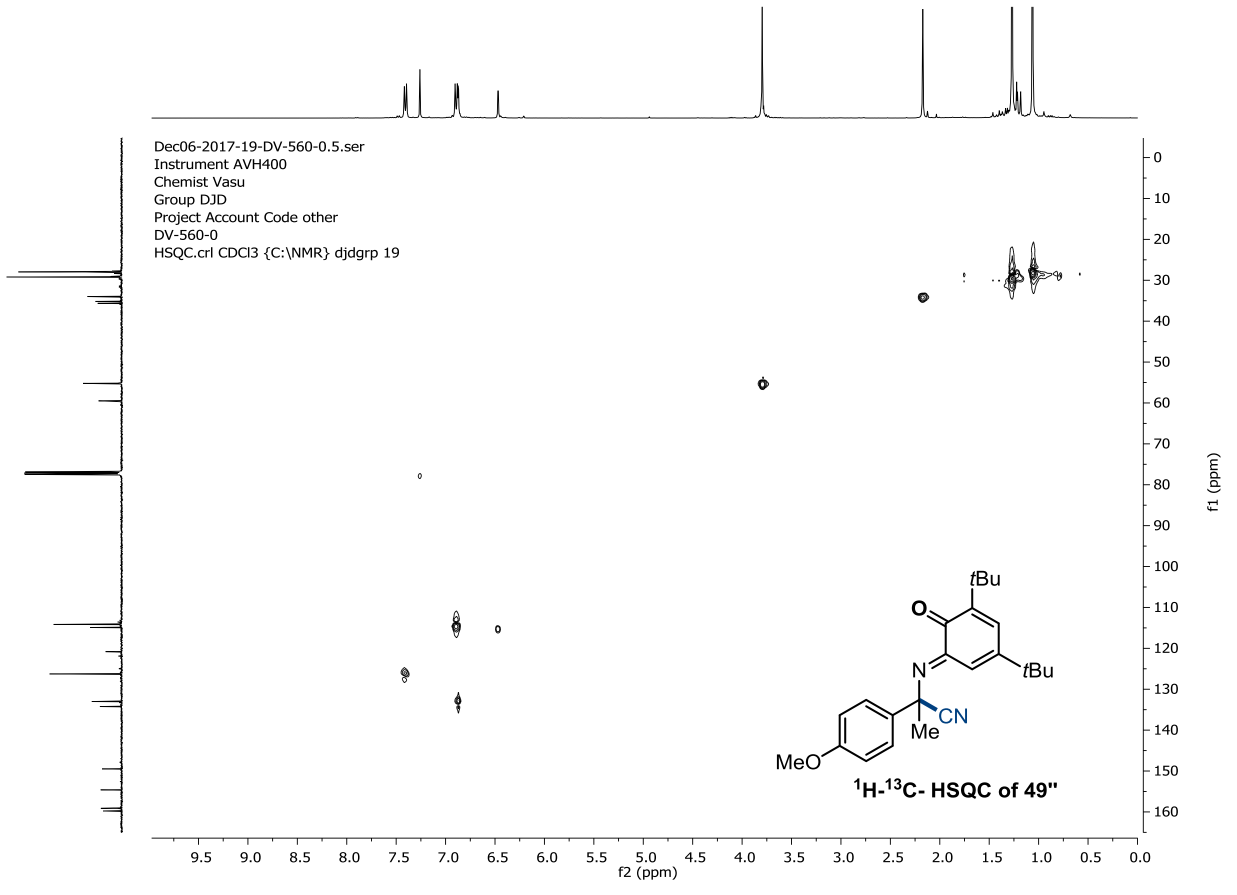
Dec06-2017-19-DV-560-0.5.ser
Instrument AVH400
Chemist Vasu
Group DJD
Project Account Code other
DV-560-0
HSQC.crl CDCl3 {C:\NMR} djdgrp 19
tBu
O
N
tBu
CN
Me
MeO
1H-13C- HSQC of 49''
f2 (ppm)
f1 (ppm)

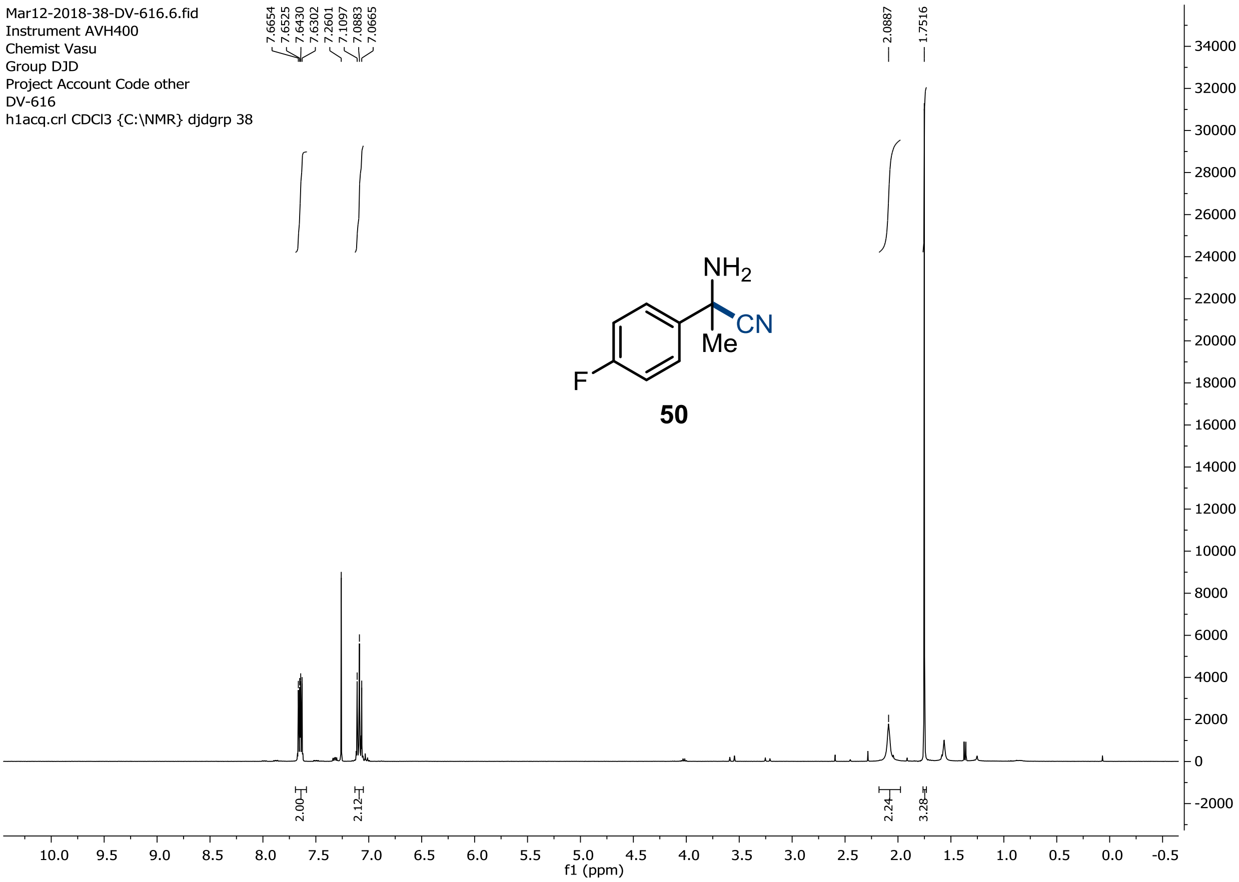

Mar12-2018-38-DV-616.6.fid
Instrument AVH400
Chemist Vasu
Group DJD
Project Account Code other
DV-616
h1acq.crl CDCl3 {C:\NMR} djdgrp 38
7.6654
7.6525
7.6430
7.6302
7.2601
7.1097
7.0883
7.0665
2.0887
1.7516
NH2
CN
Me
F
50
2.00
2.12
2.24
3.28
f1 (ppm)

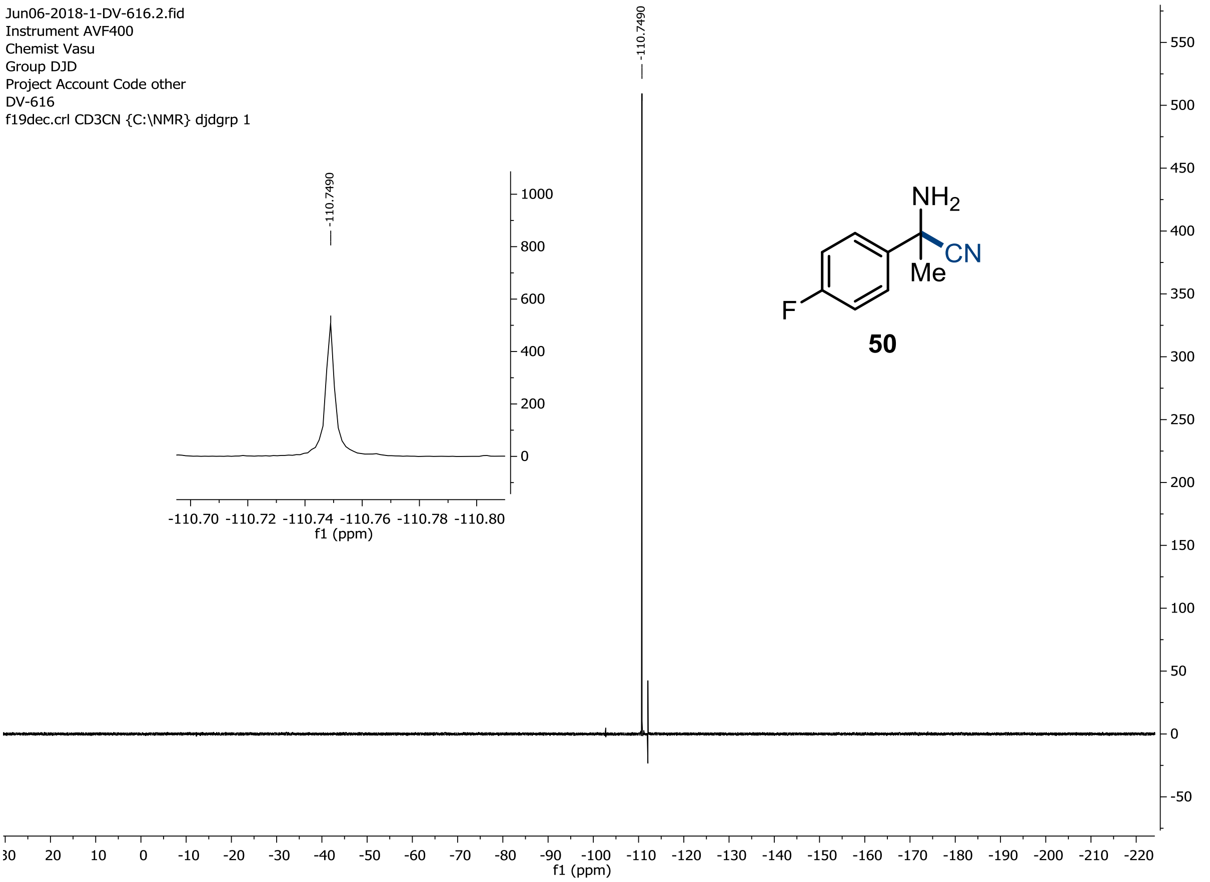

Jun06-2018-1-DV-616.2.fid
Instrument AVF400
Chemist Vasu
Group DJD
Project Account Code other
DV-616
f19dec.crl CD3CN {C:\NMR} djdgrp 1
-110.7490
-110.7490
NH2
CN
Me
F
50
f1 (ppm)

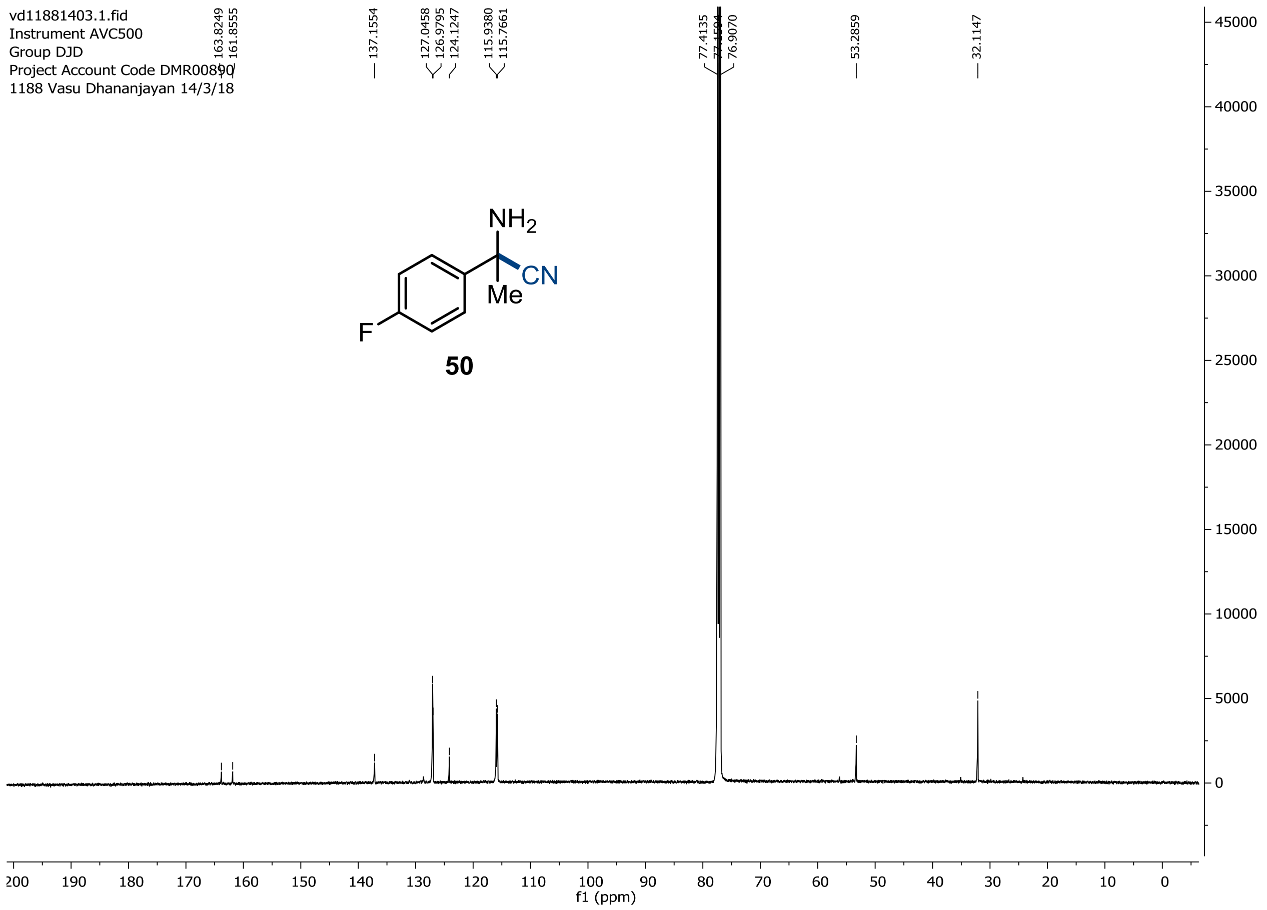
vd11881403.1.fid
Instrument AVC500
Group DJD
Project Account Code DMR00890
1188 Vasu Dhananjayan 14/3/18
163.8249
161.8555
137.1554
127.0458
126.9795
124.1247
115.9380
115.7661
77.4135
76.9070
53.2859
32.1147
NH2
CN
Me
F
50
f1 (ppm)

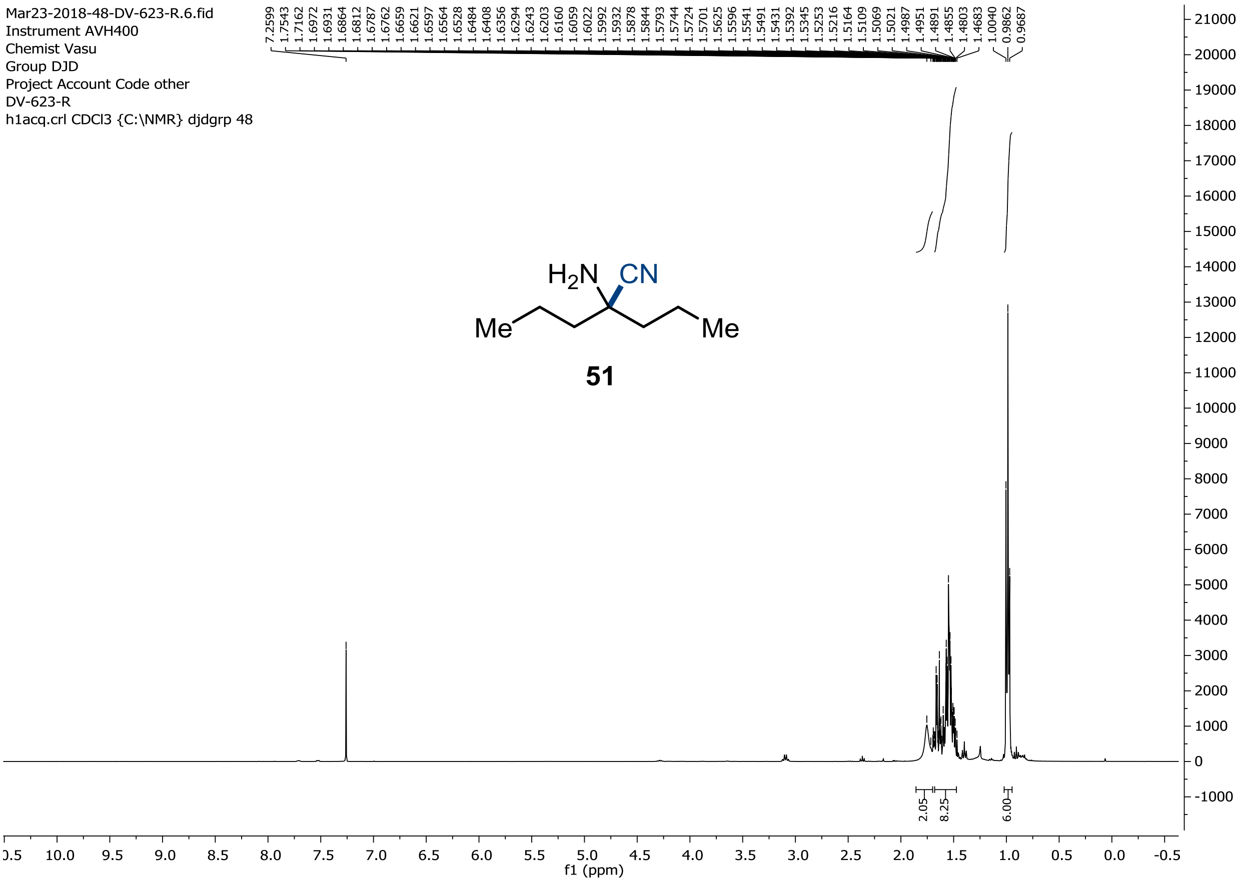
Mar23-2018-48-DV-623-R.6.fid
Instrument AVH400
Chemist Vasu
Group DJD
Project Account Code other
DV-623-R
h1acq.crl CDCl3 {C:\NMR} djdgrp 48
H2N CN
Me Me
51
f1 (ppm)

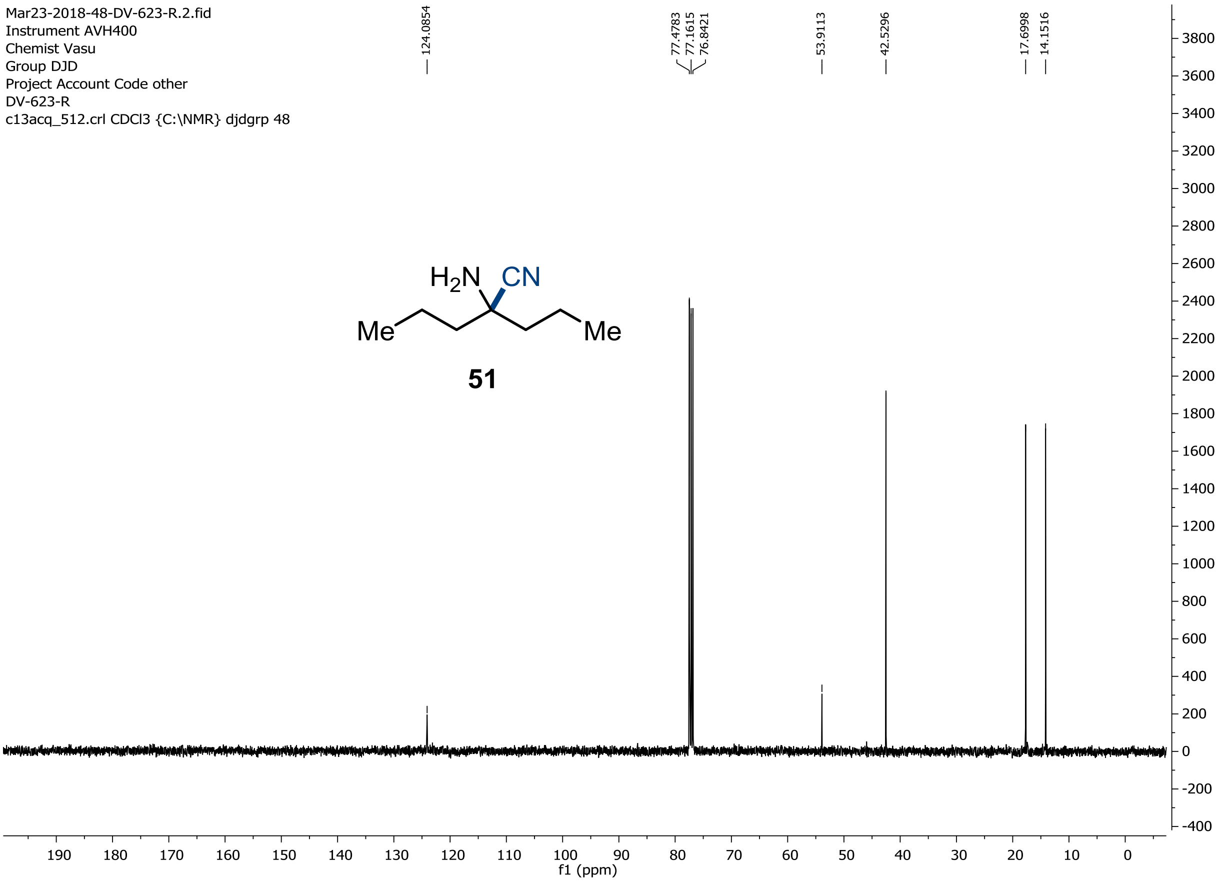
Mar23-2018-48-DV-623-R.2.fid
Instrument AVH400
Chemist Vasu
Group DJD
Project Account Code other
DV-623-R
c13acq_512.crl CDCl3 {C:\NMR} djdgrp 48
124.0854
77.4783
77.1615
76.8421
53.9113
42.5296
17.6998
14.1516
H2N CN
Me Me
51
f1 (ppm)

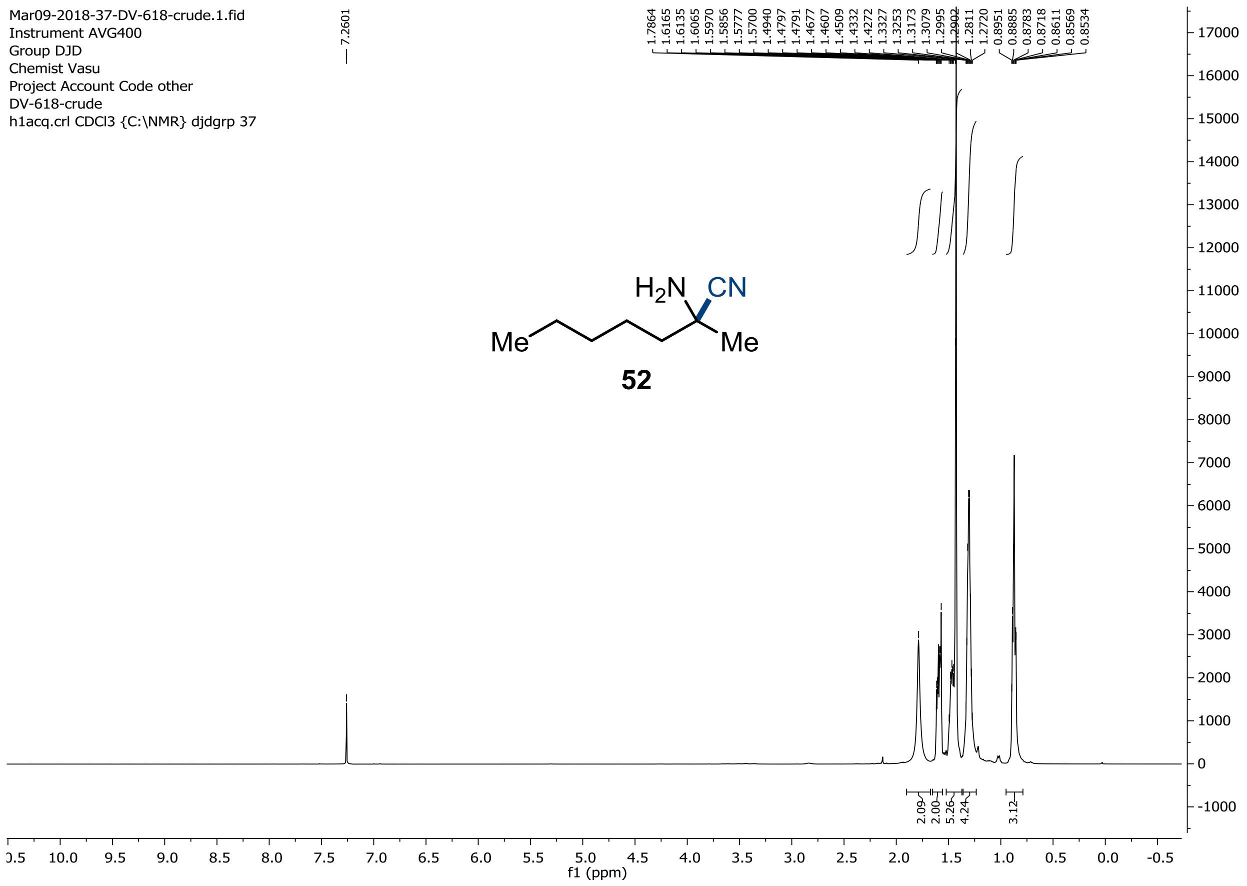

Mar09-2018-37-DV-618-crude.1.fid
Instrument AVG400
Group DJD
Chemist Vasu
Project Account Code other
DV-618-crude
h1acq.crl CDCl3 {C:\NMR} djdgrp 37
52
f1 (ppm)

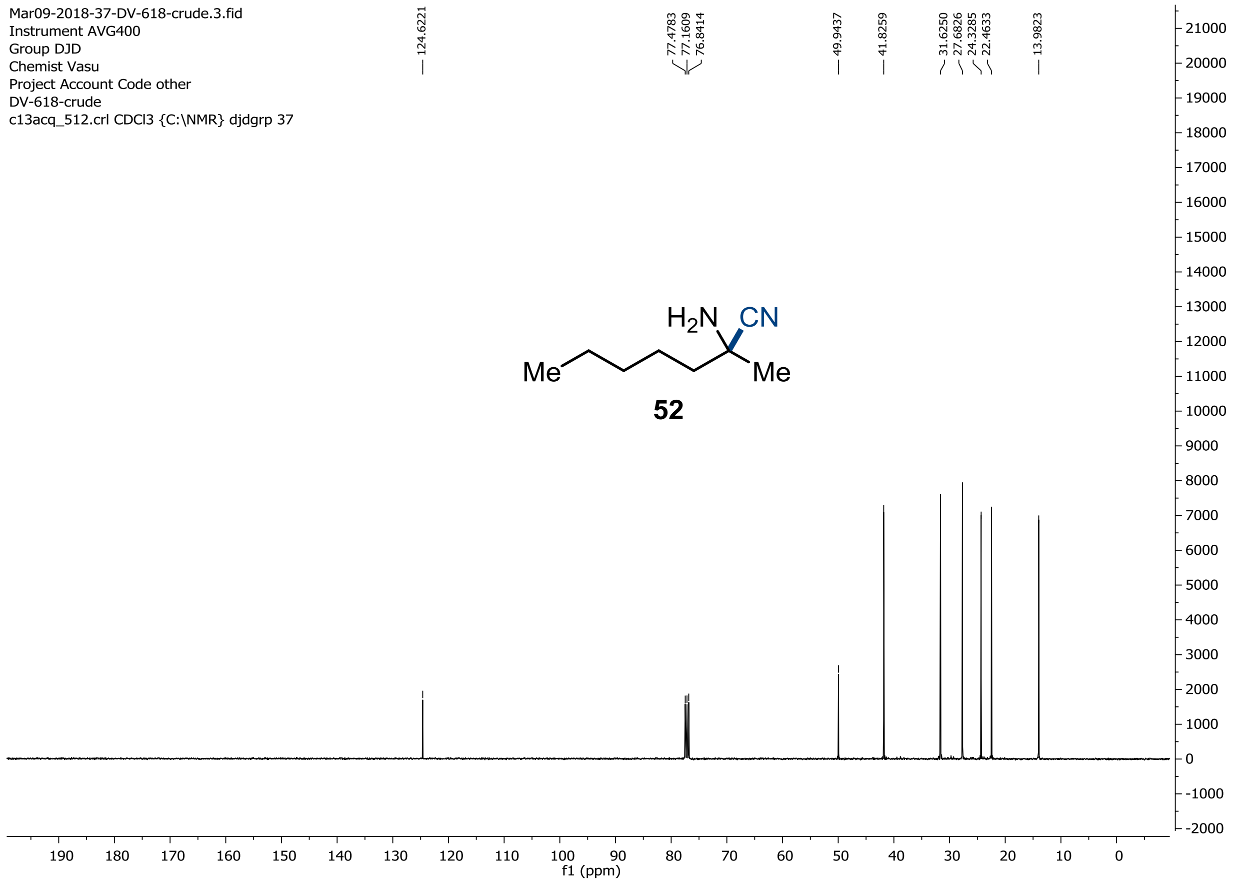

Mar09-2018-37-DV-618-crude.3.fid
Instrument AVG400
Group DJD
Chemist Vasu
Project Account Code other
DV-618-crude
c13acq_512.crl CDCl3 {C:\NMR} djdgrp 37
124.6221
77.4783
77.1609
76.8414
49.9437
41.8259
31.6250
27.6826
24.3285
22.4633
13.9823
52
f1 (ppm)

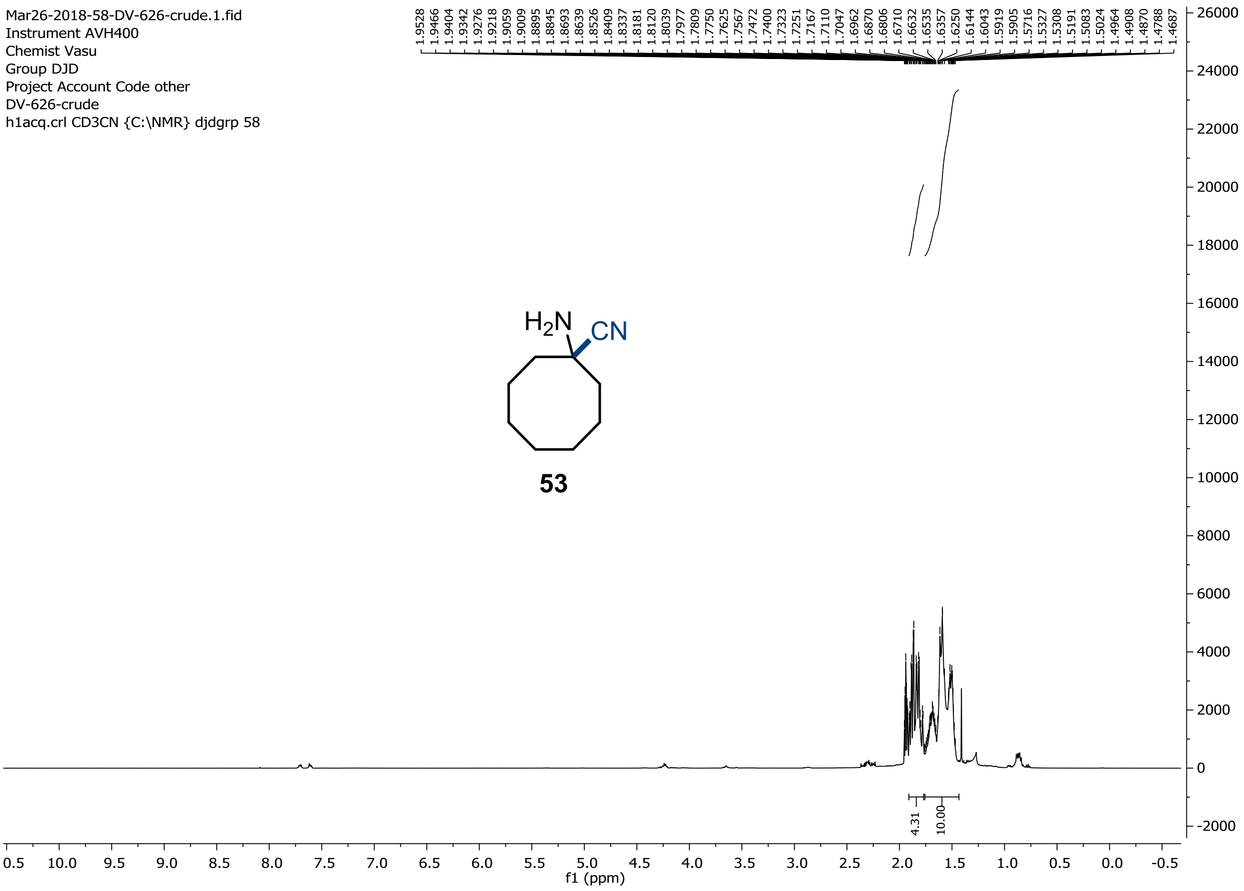
Mar26-2018-58-DV-626-crude.1.fid
Instrument AVH400
Chemist Vasu
Group DJD
Project Account Code other
DV-626-crude
h1acq.crl CD3CN {C:\NMR} djdgrp 58
1.9528 1.9466 1.9404 1.9342 1.9276 1.9218 1.9059 1.9009 1.8895 1.8845 1.8693 1.8639 1.8526 1.8409 1.8337 1.8181 1.8120 1.8039 1.7977 1.7809 1.7750 1.7625 1.7567 1.7472 1.7400 1.7323 1.7251 1.7167 1.7110 1.7047 1.6962 1.6870 1.6806 1.6710 1.6632 1.6535 1.6357 1.6250 1.6144 1.6043 1.5919 1.5905 1.5716 1.5327 1.5308 1.5191 1.5083 1.5024 1.4964 1.4908 1.4870 1.4788 1.4687
H2N
CN
53
4.31
10.00
f1 (ppm)

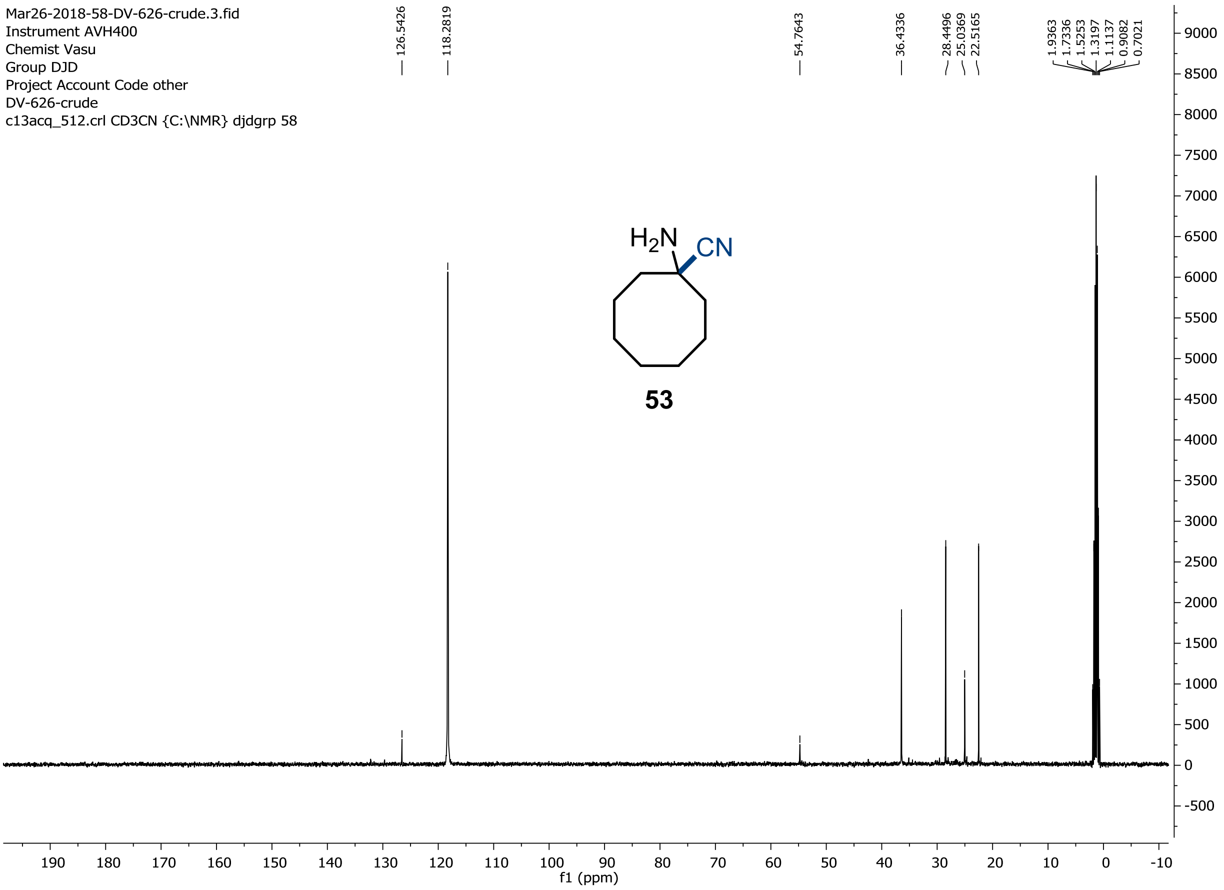
Mar26-2018-58-DV-626-crude.3.fid
Instrument AVH400
Chemist Vasu
Group DJD
Project Account Code other
DV-626-crude
c13acq_512.crl CD3CN {C:\NMR} djdgrp 58
126.5426
118.2819
54.7643
36.4336
28.4496 25.0369 22.5165
1.9363 1.7336 1.5253 1.3197 1.1137 0.9082 0.7021
H2N
CN
53
f1 (ppm)

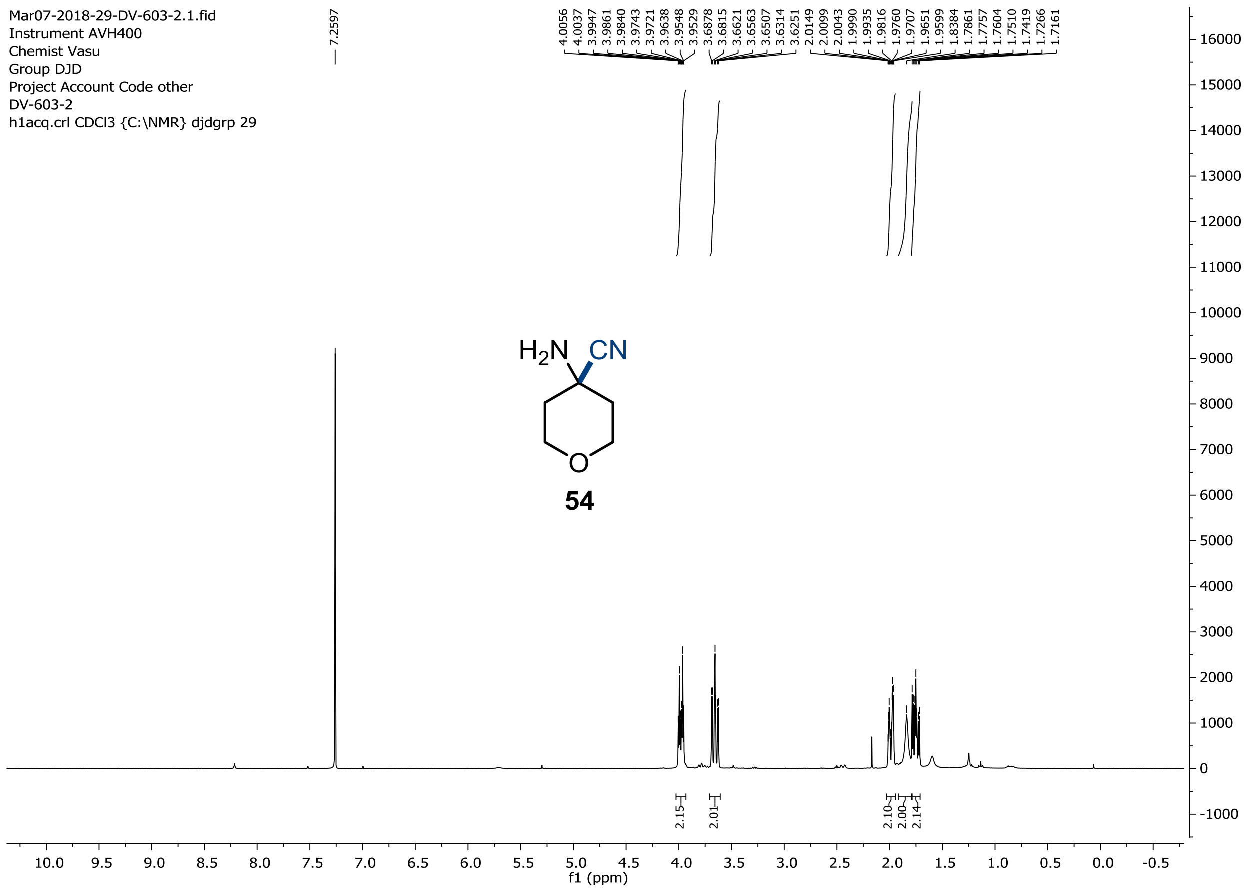
Mar07-2018-29-DV-603-2.1.fid
Instrument AVH400
Chemist Vasu
Group DJD
Project Account Code other
DV-603-2
h1acq.crl CDCl3 {C:\NMR} djdgrp 29
7.2597
4.0056
4.0037
3.9947
3.9861
3.9840
3.9743
3.9721
3.9638
3.9548
3.9529
3.6878
3.6815
3.6621
3.6563
3.6507
3.6314
3.6251
2.0149
2.0099
2.0043
1.9990
1.9935
1.9816
1.9760
1.9707
1.9651
1.9599
1.8384
1.7861
1.7757
1.7604
1.7510
1.7419
1.7266
1.7161
H2N
CN
O
54
2.15
2.01
2.10
2.00
2.14
f1 (ppm)

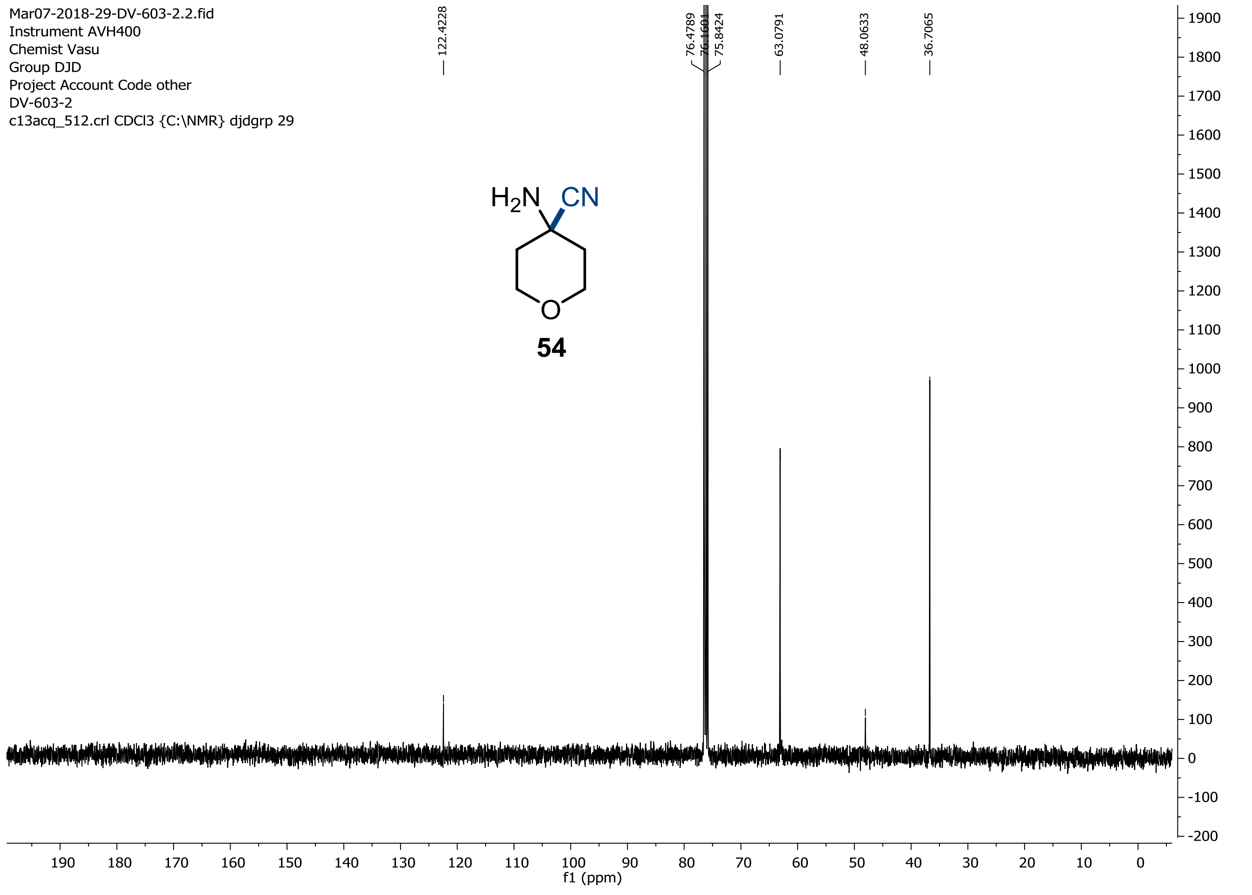
Mar07-2018-29-DV-603-2.2.fid
Instrument AVH400
Chemist Vasu
Group DJD
Project Account Code other
DV-603-2
c13acq_512.crl CDCl3 {C:\NMR} djdgrp 29
122.4228
76.4789
75.8424
63.0791
48.0633
36.7065
H2N
CN
O
54
f1 (ppm)

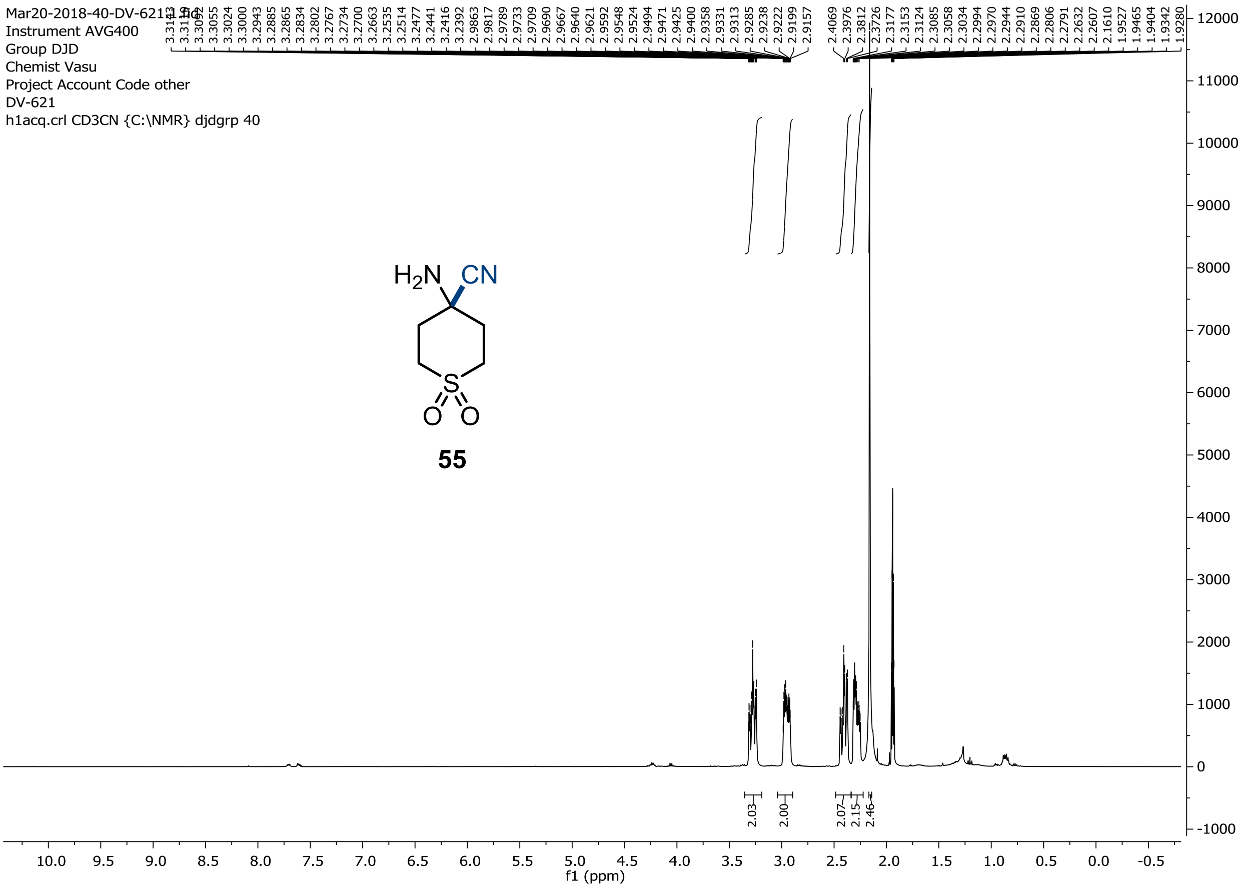
Mar20-2018-40-DV-621.1.fid
Instrument AVG400
Group DJD
Chemist Vasu
Project Account Code other
DV-621
h1acq.crl CD3CN {C:\NMR} djdgrp 40
55
f1 (ppm)

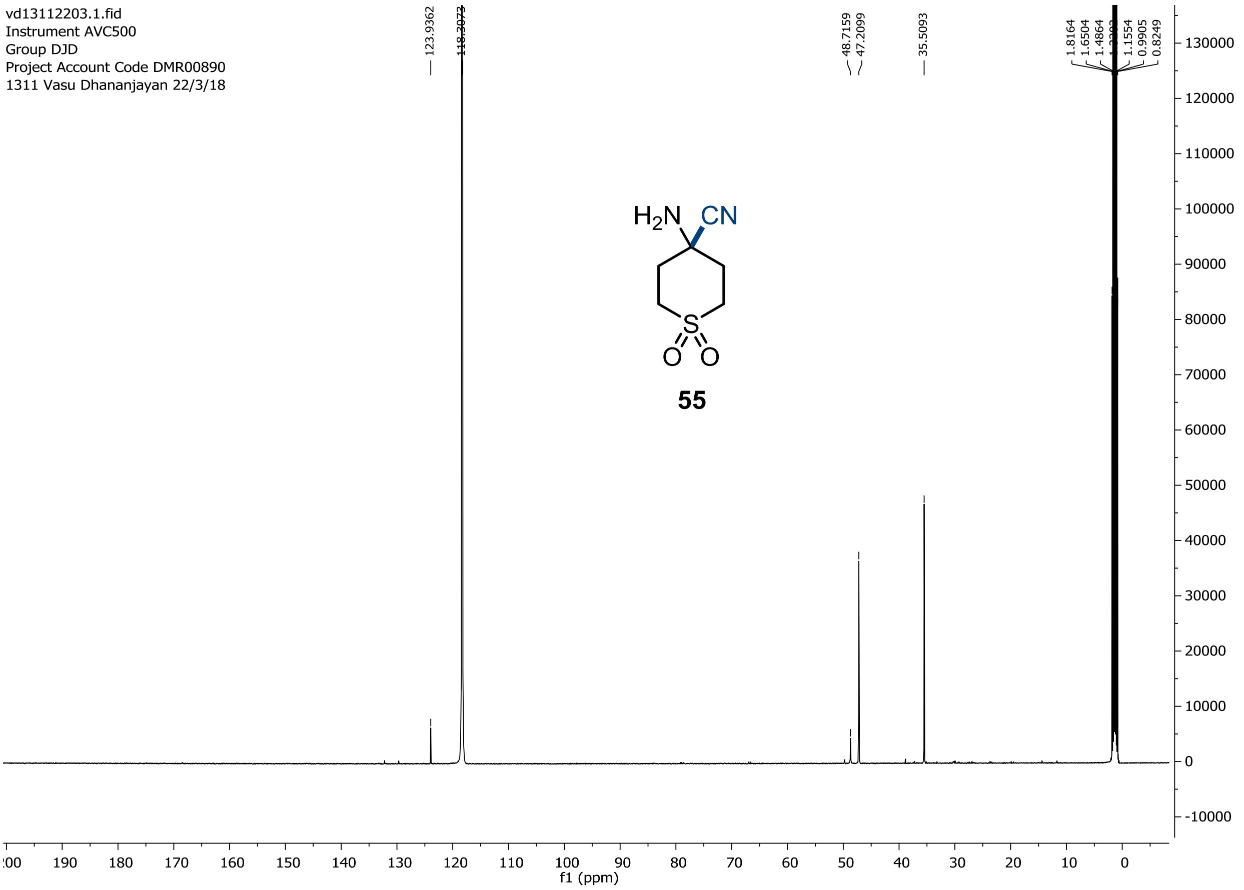
vd13112203.1.fid
Instrument AVC500
Group DJD
Project Account Code DMR00890
1311 Vasu Dhananjayan 22/3/18
123.9362
118.3073
48.7159
47.2099
35.5093
55
f1 (ppm)

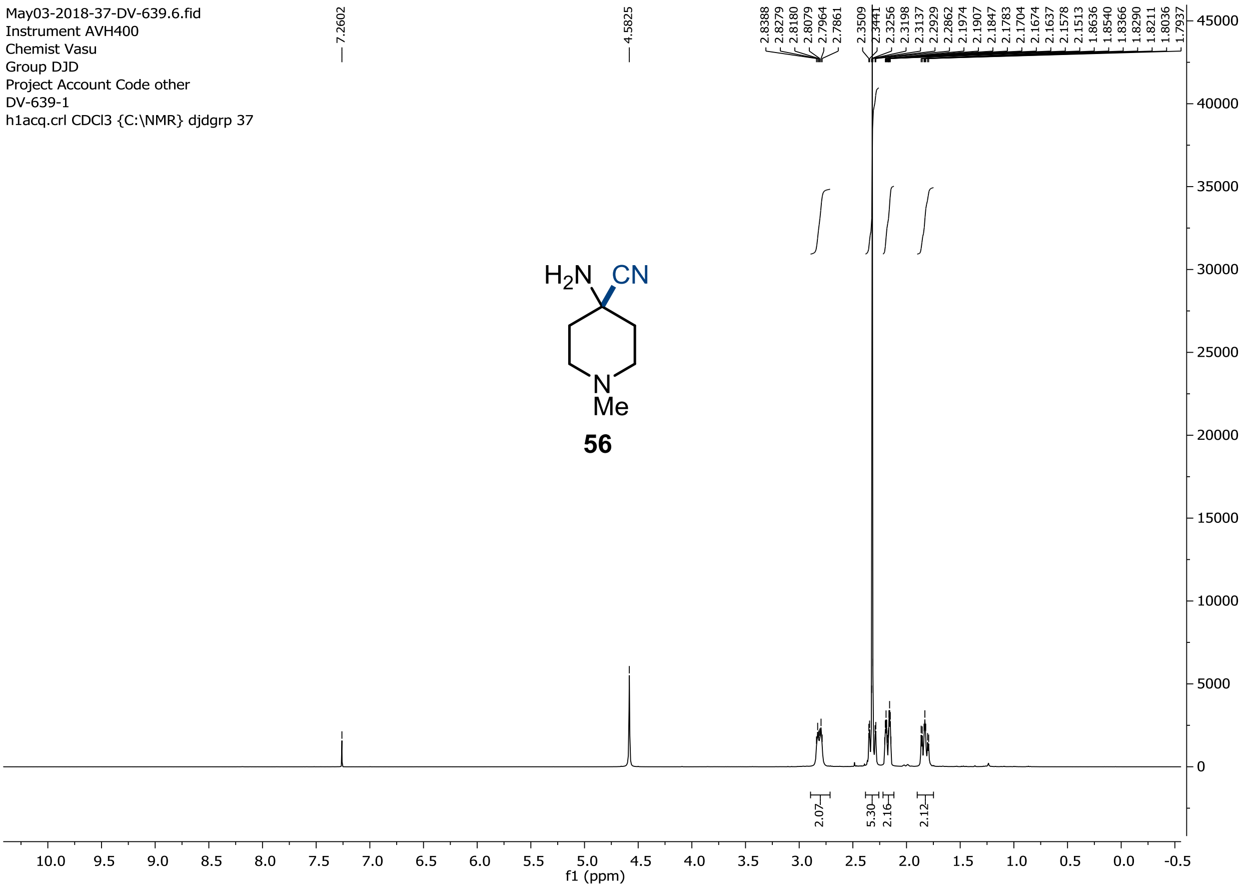
May03-2018-37-DV-639.6.fid
Instrument AVH400
Chemist Vasu
Group DJD
Project Account Code other
DV-639-1
h1acq.crl CDCl3 {C:\NMR} djdgrp 37
7.2602
4.5825
2.8388 2.8279 2.8180 2.8079 2.7964 2.7861
2.3509 2.3441 2.3256 2.3198 2.3137 2.2929 2.2862 2.1974 2.1907 2.1847 2.1783 2.1704 2.1674 2.1637 2.1578 2.1513 1.8636 1.8540 1.8366 1.8290 1.8211 1.8036 1.7937
H_2N CN
N
Me
56
2.07
5.30
2.16
2.12
f1 (ppm)

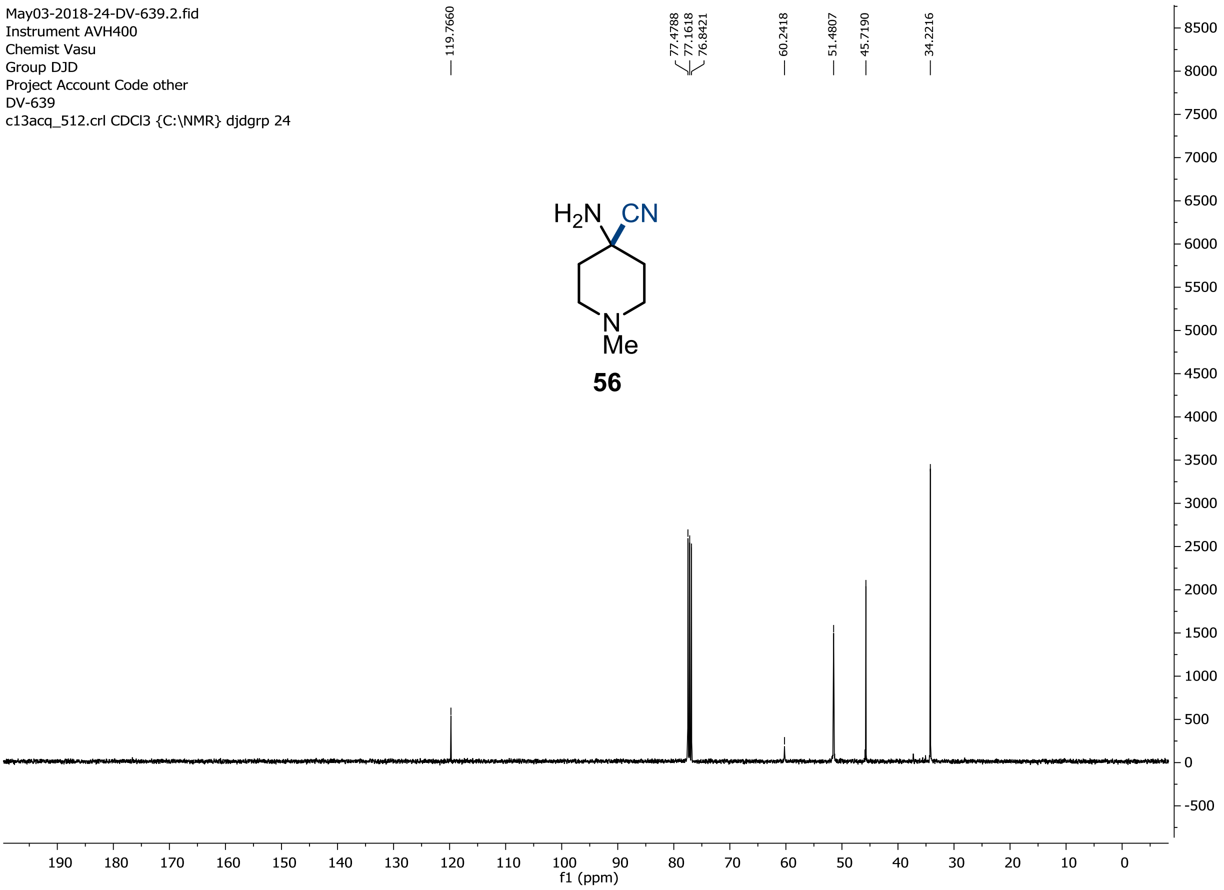
May03-2018-24-DV-639.2.fid
Instrument AVH400
Chemist Vasu
Group DJD
Project Account Code other
DV-639
c13acq_512.crl CDCl3 {C:\NMR} djdgrp 24
119.7660
77.4788 77.1618 76.8421
60.2418
51.4807
45.7190
34.2216
H_2N CN
N
Me
56
f1 (ppm)

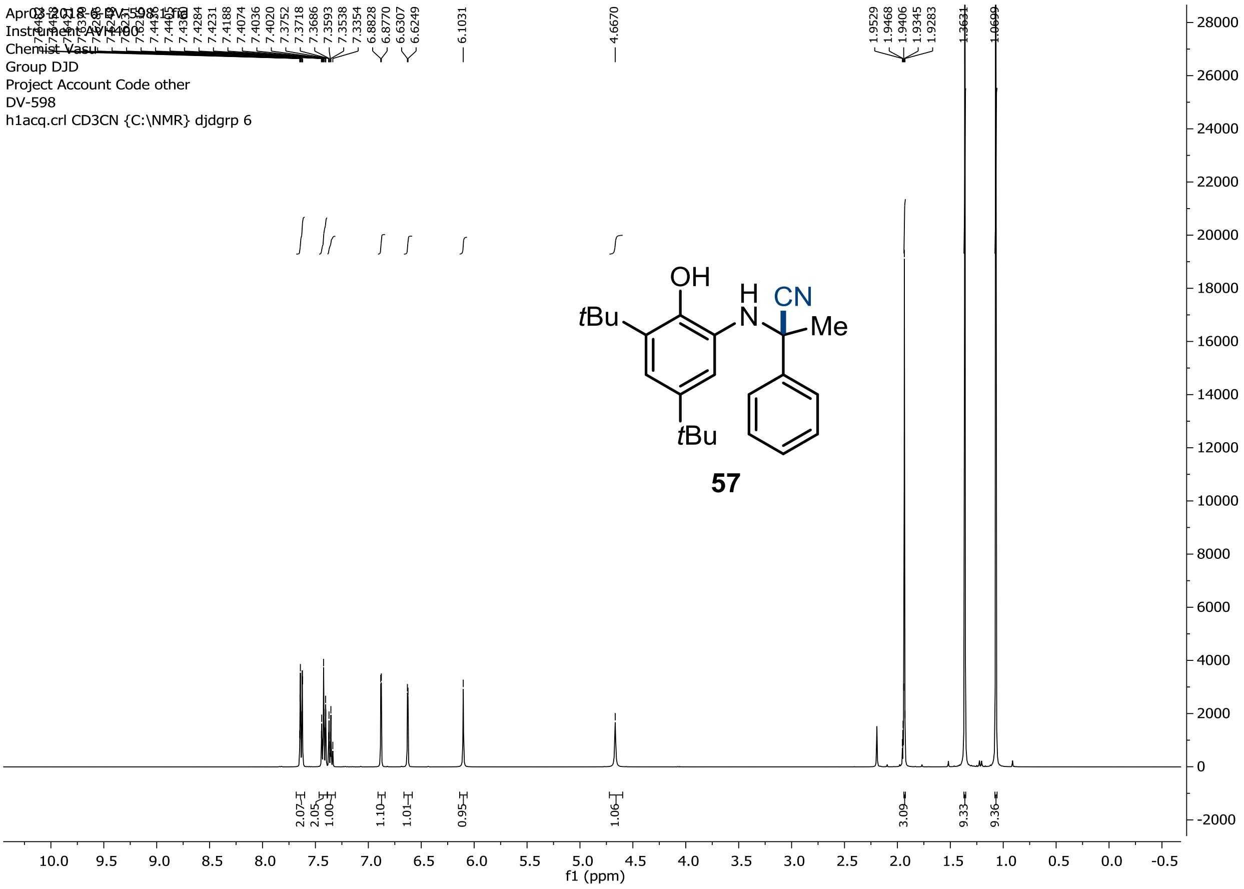
Instrument AVH400
Chemist Vasu
Group DJD
Project Account Code other
DV-598
h1acq.crl CD3CN {C:\NMR} djdgrp 6
6.1031
4.6670
1.9529
1.9468
1.9406
1.9345
1.9283
1.3631
1.0699
OH
H
N
CN
Me
tBu
tBu
57
2.07
2.05
1.00
1.10
1.01
0.95
1.06
3.09
9.33
9.36
f1 (ppm)

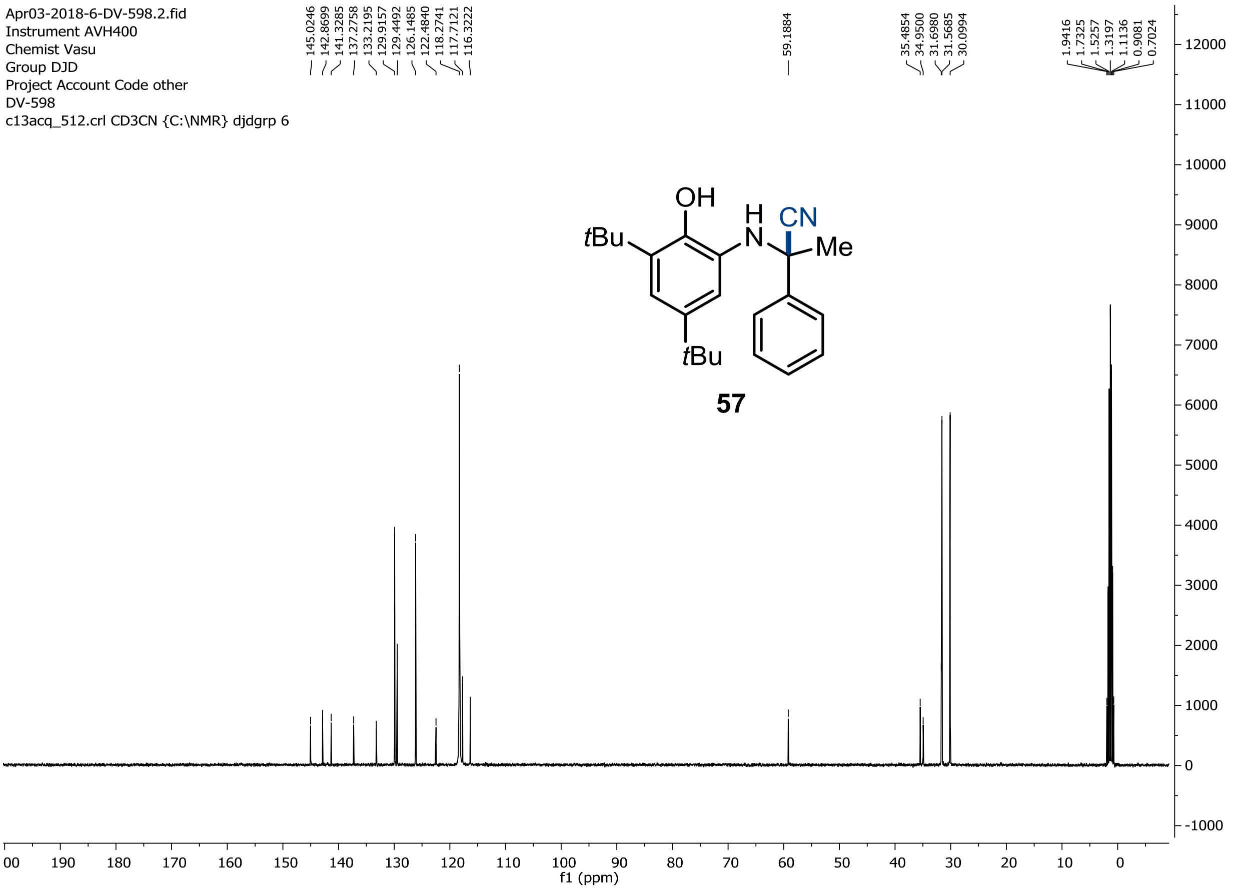
Apr03-2018-6-DV-598.2.fid
Instrument AVH400
Chemist Vasu
Group DJD
Project Account Code other
DV-598
c13acq_512.crl CD3CN {C:\NMR} djdgrp 6
145.0246
142.8699
141.3285
137.2758
133.2195
129.9157
129.4492
126.1485
122.4840
118.2741
117.7121
116.3222
59.1884
35.4854
34.9500
31.6980
31.5685
30.0994
1.9416
1.7325
1.5257
1.3197
1.1136
0.9081
0.7024
OH
H
N
CN
Me
tBu
tBu
57
f1 (ppm)

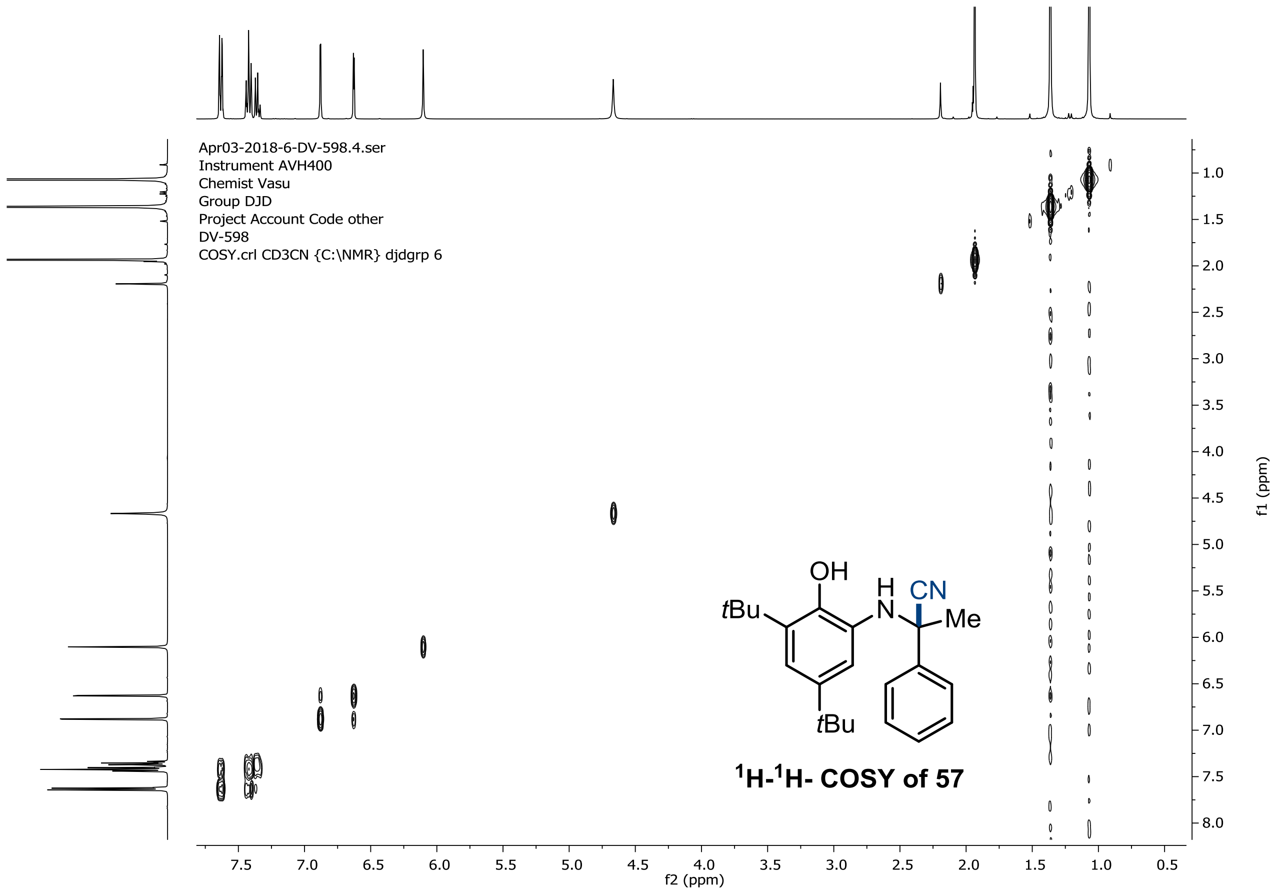
Apr03-2018-6-DV-598.4.ser
Instrument AVH400
Chemist Vasu
Group DJD
Project Account Code other
DV-598
COSY.crl CD3CN {C:\NMR} djdgrp 6
OH
tBu
H
N
CN
Me
tBu
1H-1H- COSY of 57
f2 (ppm)
f1 (ppm)

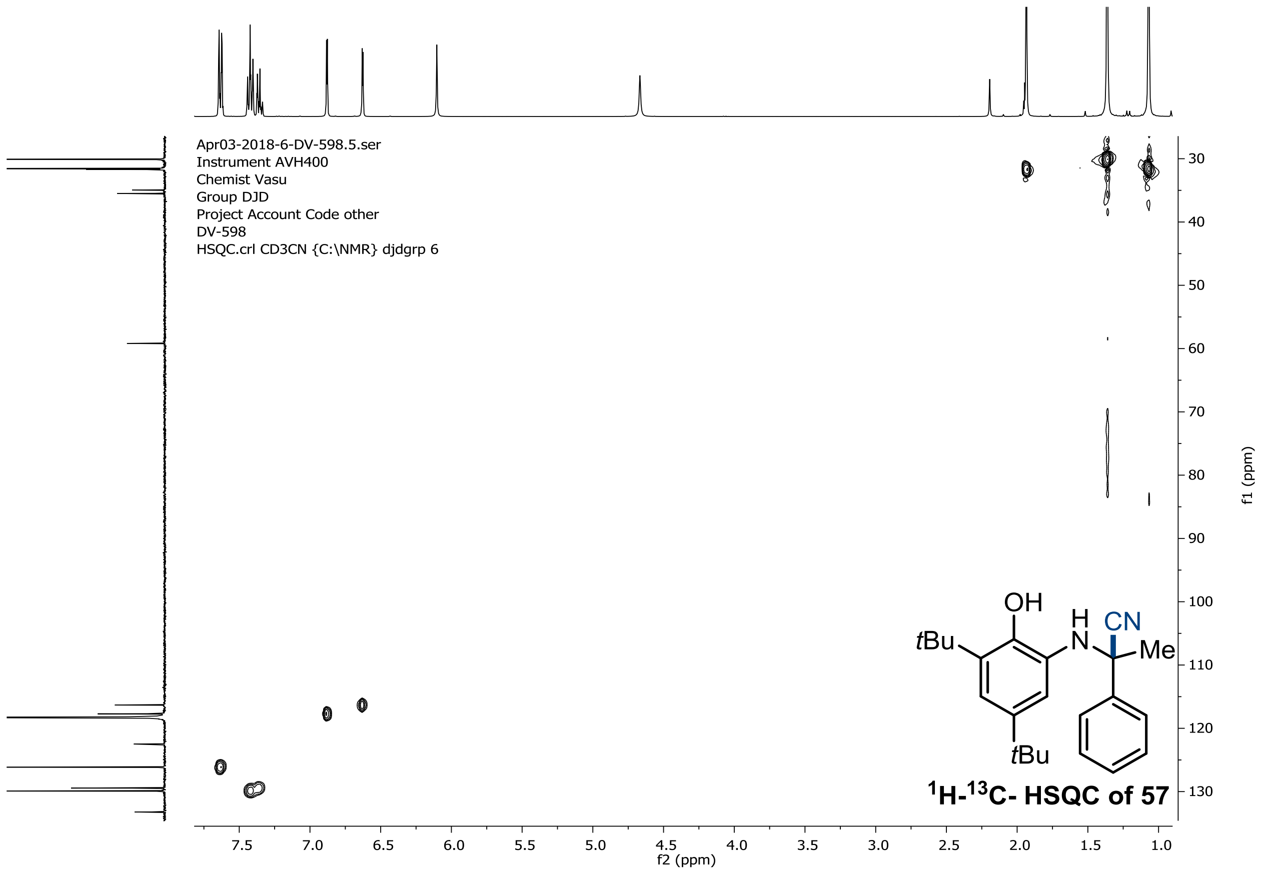
Apr03-2018-6-DV-598.5.ser
Instrument AVH400
Chemist Vasu
Group DJD
Project Account Code other
DV-598
HSQC.crl CD3CN {C:\NMR} djdgrp 6
OH
tBu
H
N
CN
Me
tBu
1H-13C- HSQC of 57
f2 (ppm)
f1 (ppm)

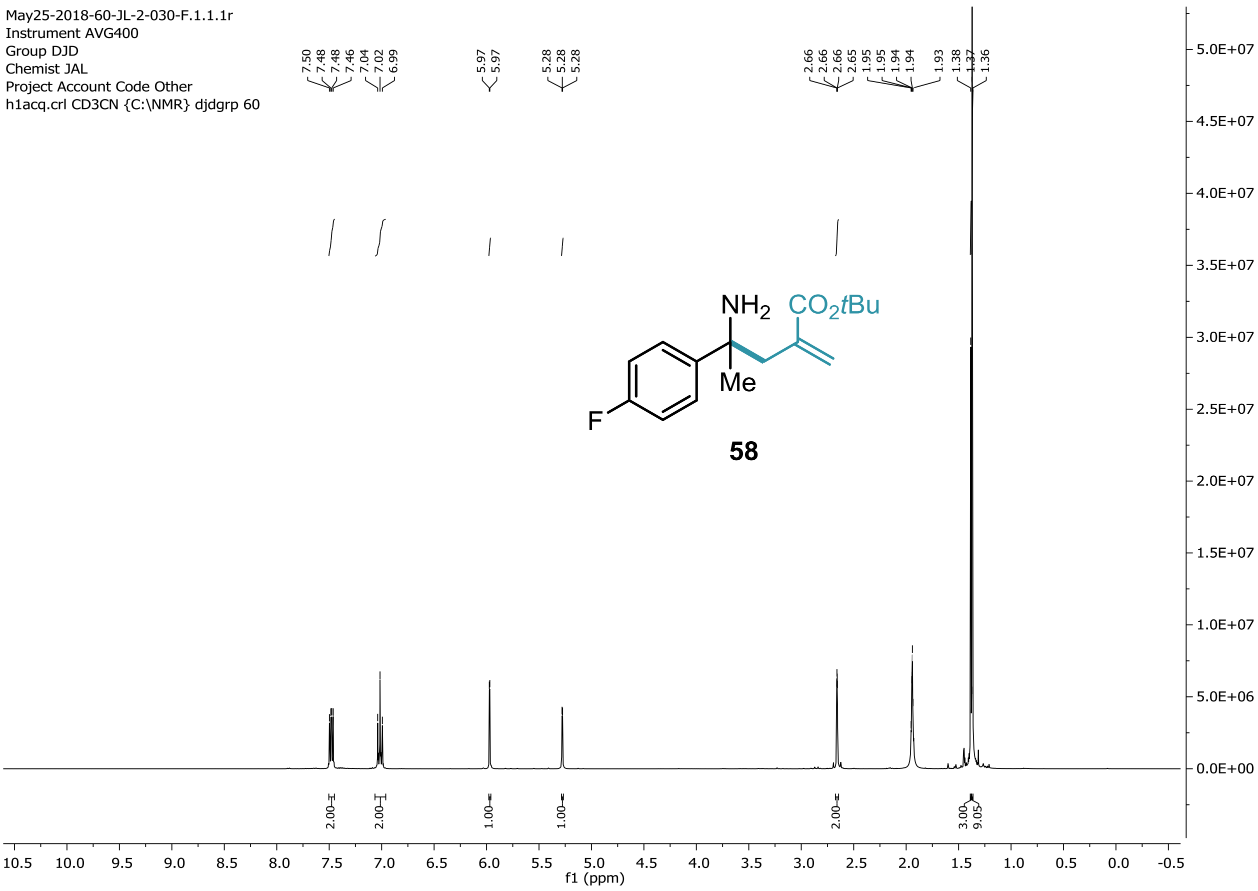
May25-2018-60-JL-2-030-F.1.1.1r
Instrument AVG400
Group DJD
Chemist JAL
Project Account Code Other
h1acq.crl CD3CN {C:\NMR} djdgrp 60
NH2 CO2tBu
F
Me
58
f1 (ppm)

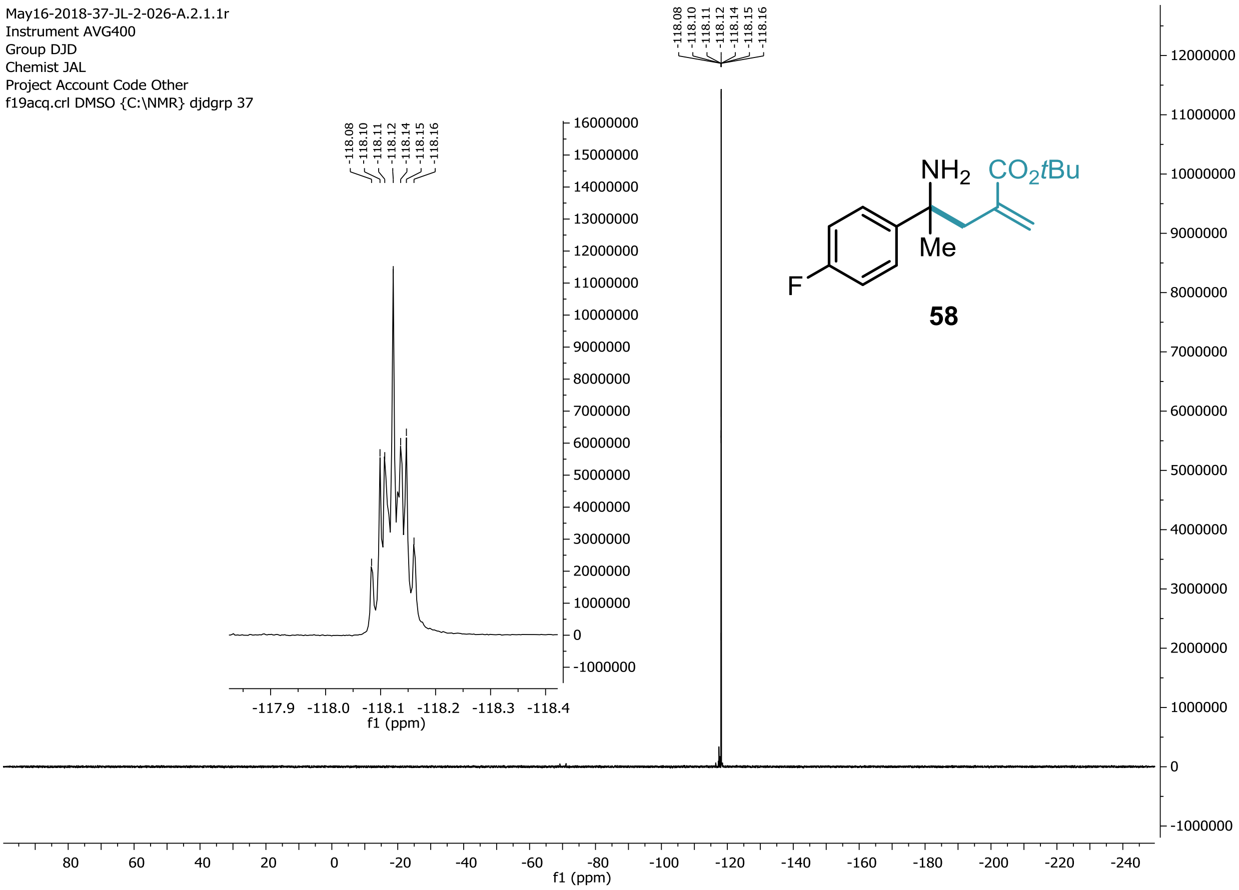
May16-2018-37-JL-2-026-A.2.1.1r
Instrument AVG400
Group DJD
Chemist JAL
Project Account Code Other
f19acq.crl DMSO {C:\NMR} djdgrp 37
NH2 CO2tBu
F
Me
58
f1 (ppm)

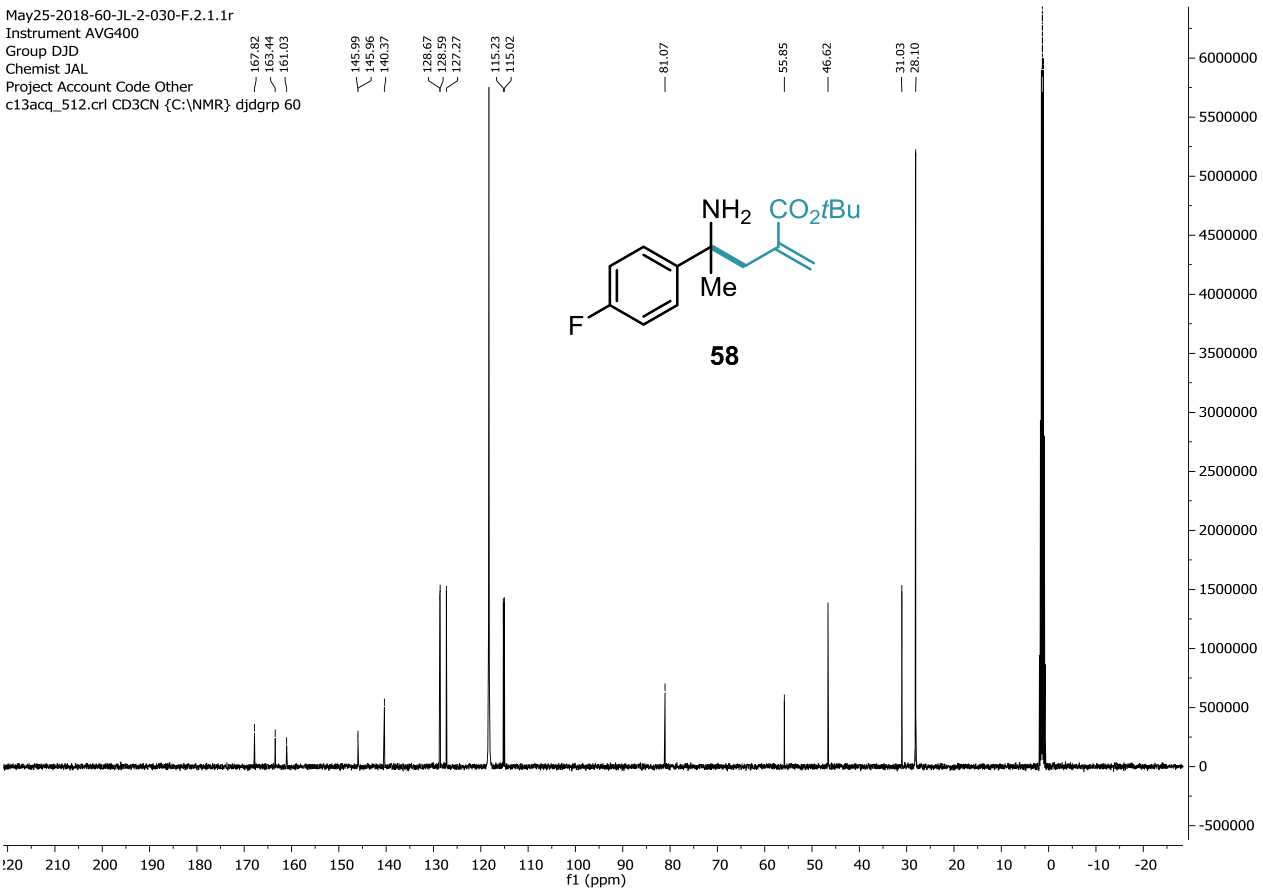
May25-2018-60-JL-2-030-F.2.1.1r
Instrument AVG400
Group DJD
Chemist JAL
Project Account Code Other
c13acq_512.crl CD3CN {C:\NMR} djdgrp 60
167.82
163.44
161.03
145.99
145.96
140.37
128.67
128.59
127.27
115.23
115.02
81.07
55.85
46.62
31.03
28.10
NH2
CO2tBu
Me
F
58
f1 (ppm)

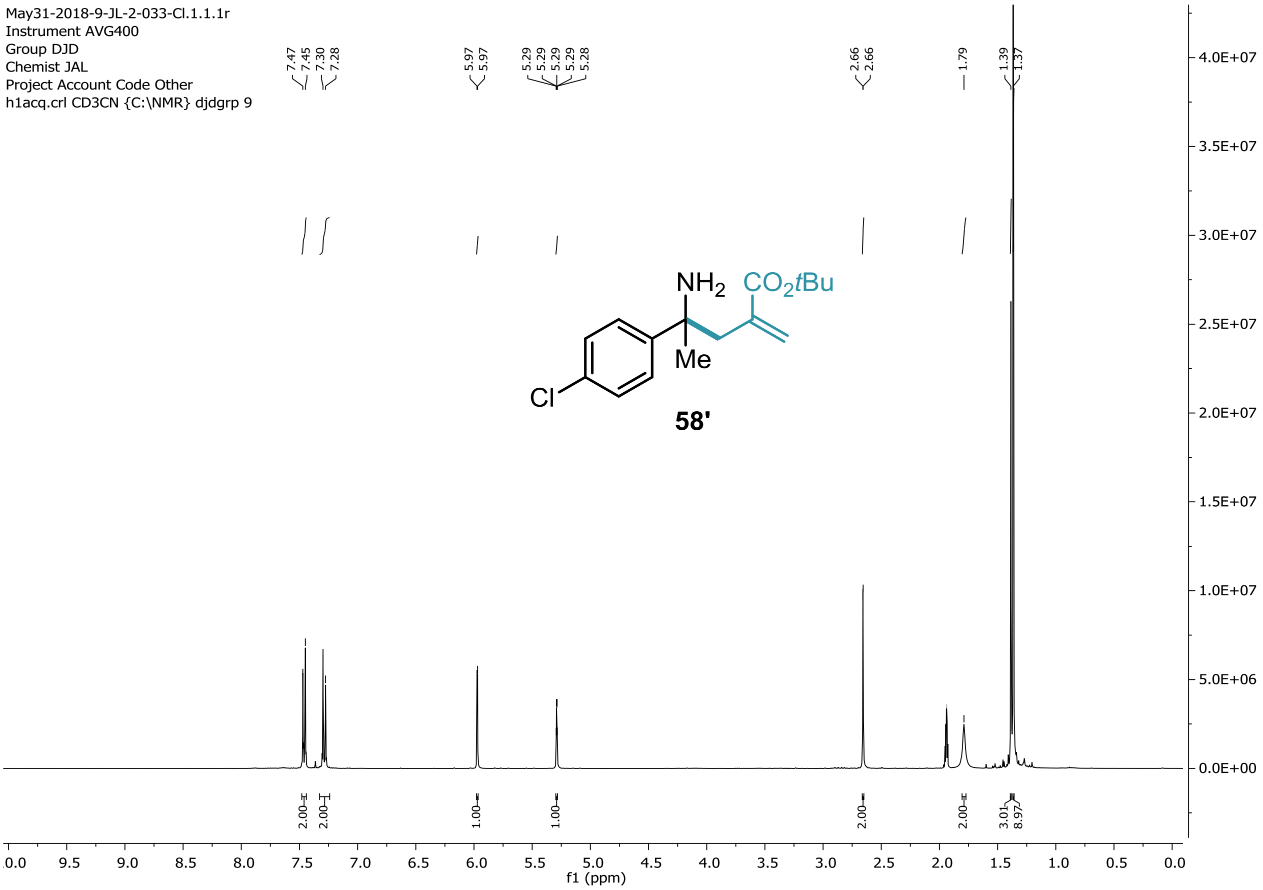
May31-2018-9-JL-2-033-Cl.1.1.1r
Instrument AVG400
Group DJD
Chemist JAL
Project Account Code Other
h1acq.crl CD3CN {C:\NMR} djdgrp 9
7.47 7.45 7.30 7.28
5.97 5.97
5.29 5.29 5.29 5.29 5.28
2.66 2.66
1.79
1.39 1.37
NH2 CO2tBu
Me
Cl
58'
2.00 2.00 1.00 1.00 2.00 2.00 3.01 8.97
f1 (ppm)

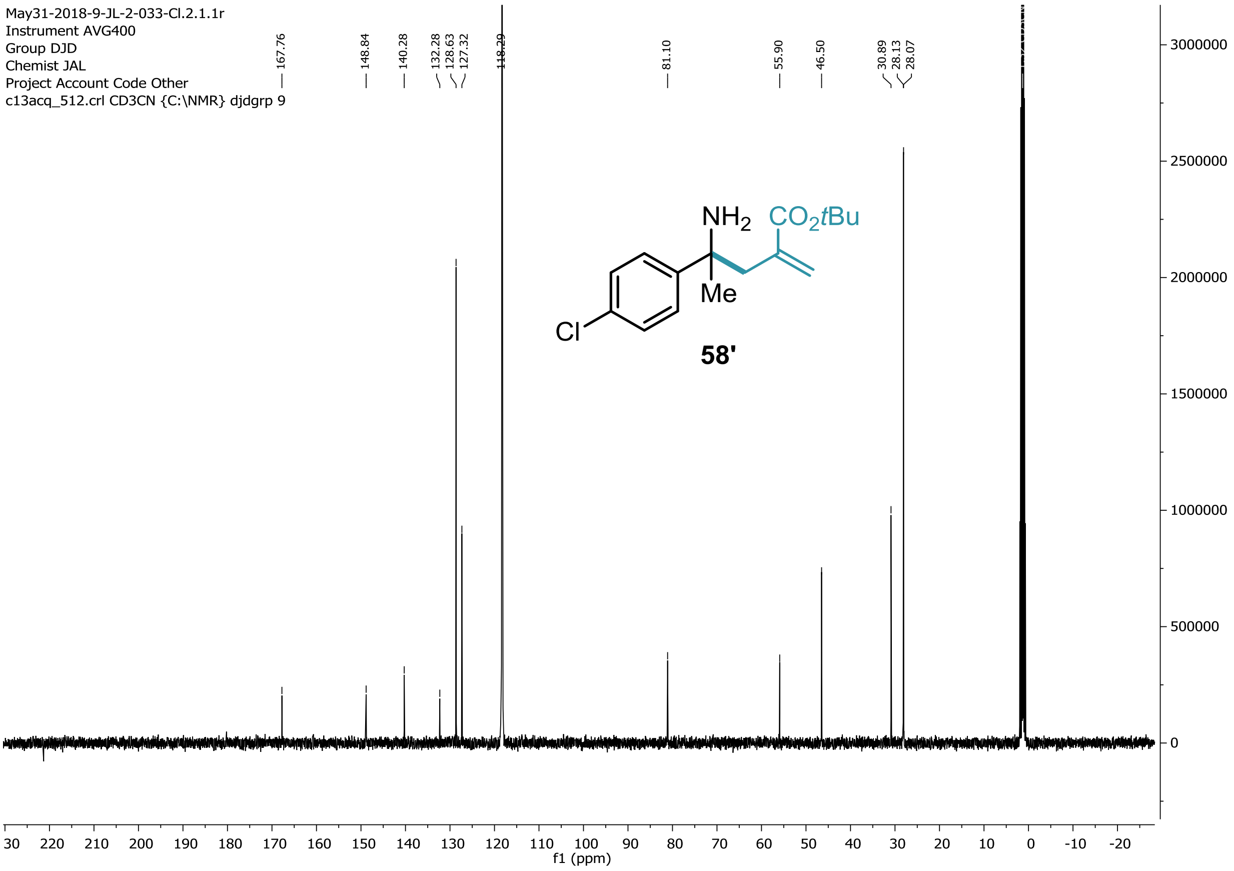
May31-2018-9-JL-2-033-Cl.2.1.1r
Instrument AVG400
Group DJD
Chemist JAL
Project Account Code Other
c13acq_512.crl CD3CN {C:\NMR} djdgrp 9
167.76
148.84
140.28
132.28 128.63 127.32
118.29
81.10
55.90
46.50
30.89 28.13 28.07
NH2 CO2tBu
Me
Cl
58'
f1 (ppm)

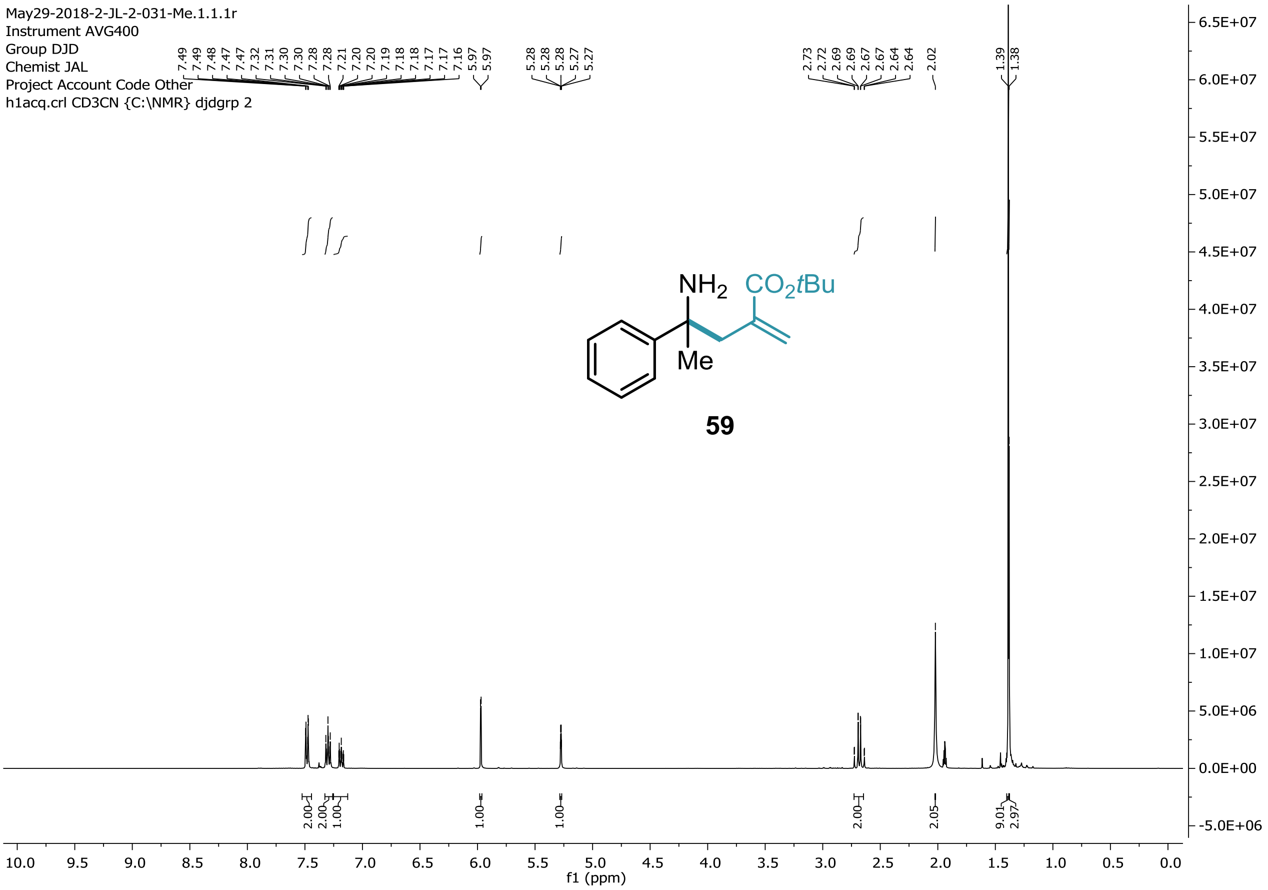
May29-2018-2-JL-2-031-Me.1.1.1r
Instrument AVG400
Group DJD
Chemist JAL
Project Account Code Other
h1acq.crl CD3CN {C:\NMR} djdgrp 2
NH2
CO2tBu
Me
f1 (ppm)

59

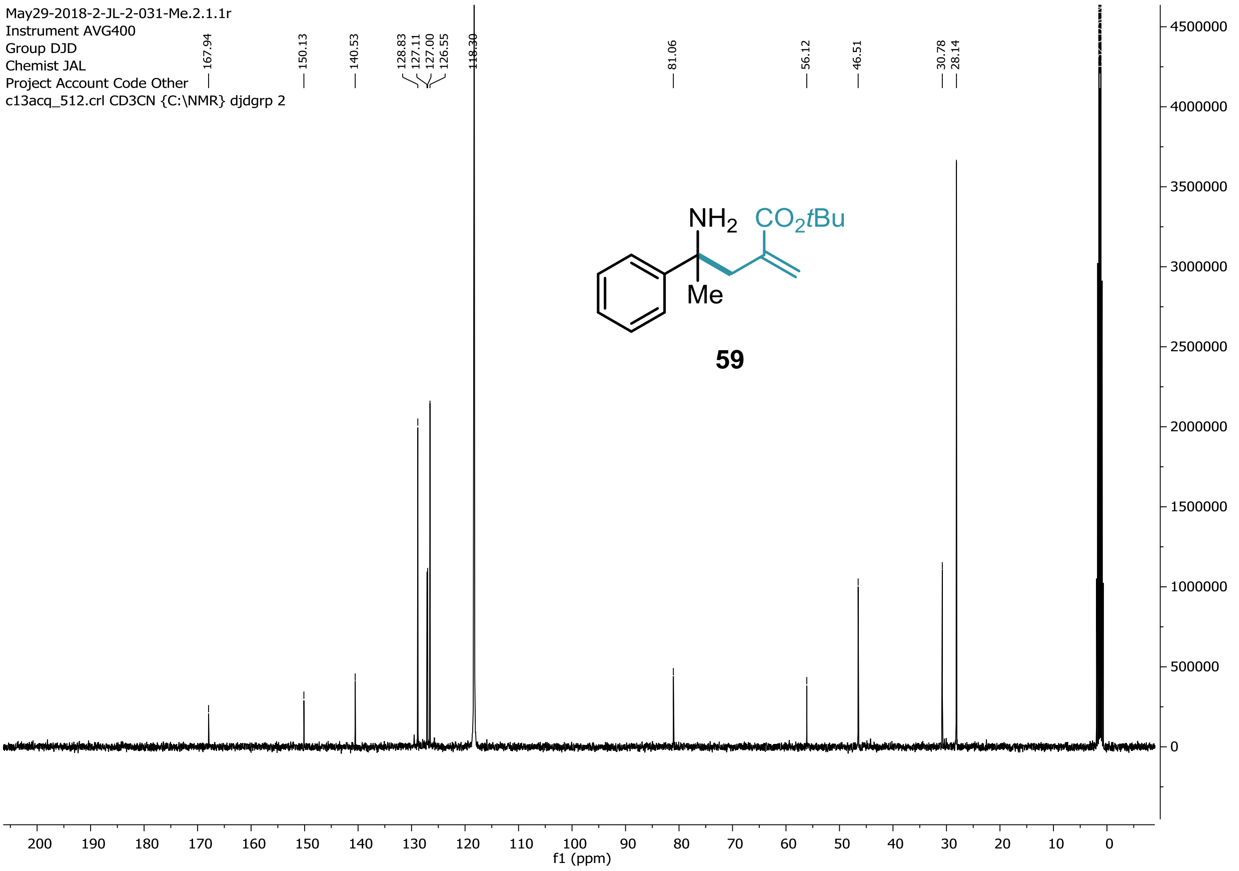
May29-2018-2-JL-2-031-Me.2.1.1r
Instrument AVG400
Group DJD
Chemist JAL
Project Account Code Other
c13acq_512.crl CD3CN {C:\NMR} djdgrp 2
167.94
150.13
140.53
128.83
127.11
127.00
126.55
118.30
81.06
56.12
46.51
30.78
28.14
NH2
CO2tBu
Me
f1 (ppm)

59

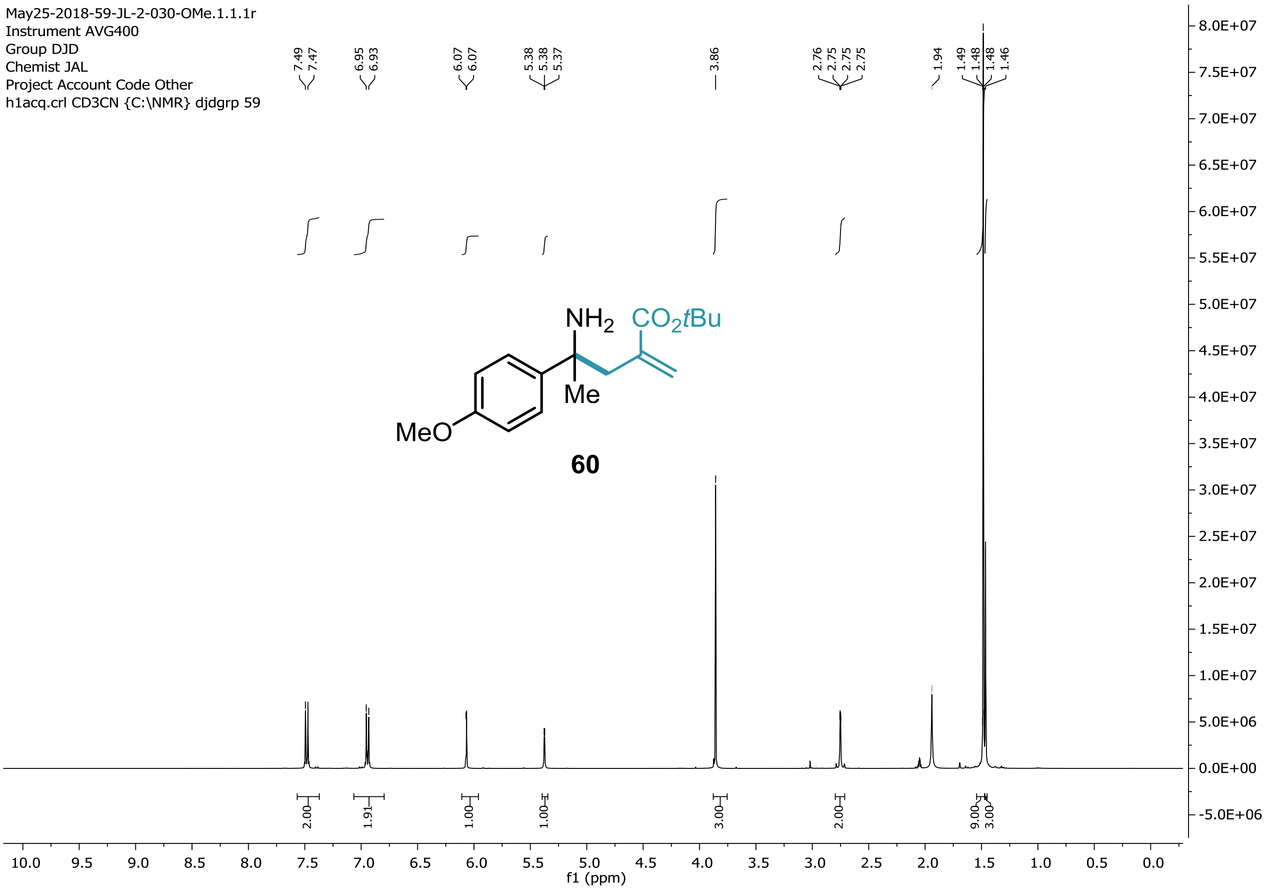

May25-2018-59-JL-2-030-OMe.1.1.1r
Instrument AVG400
Group DJD
Chemist JAL
Project Account Code Other
h1acq.crl CD3CN {C:\NMR} djdgrp 59
7.49
7.47
6.95
6.93
6.07
6.07
5.38
5.38
5.37
3.86
2.76
2.75
2.75
2.75
1.94
1.49
1.48
1.48
1.46
NH2
CO2tBu
Me
MeO
60
f1 (ppm)

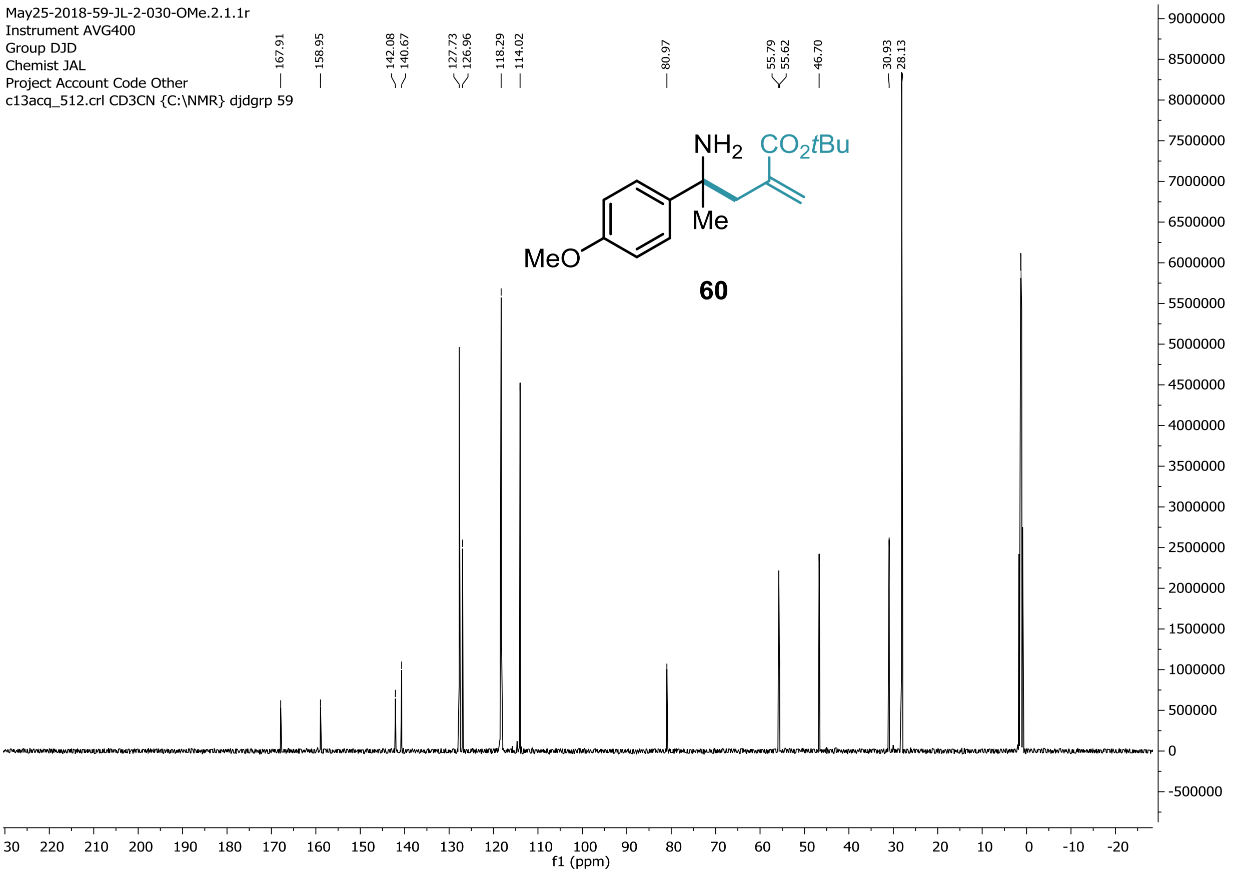

May25-2018-59-JL-2-030-OMe.2.1.1r
Instrument AVG400
Group DJD
Chemist JAL
Project Account Code Other
c13acq_512.crl CD3CN {C:\NMR} djdgrp 59
167.91
158.95
142.08
140.67
127.73
126.96
118.29
114.02
80.97
55.79
55.62
46.70
30.93
28.13
NH2
CO2tBu
Me
MeO
60
f1 (ppm)

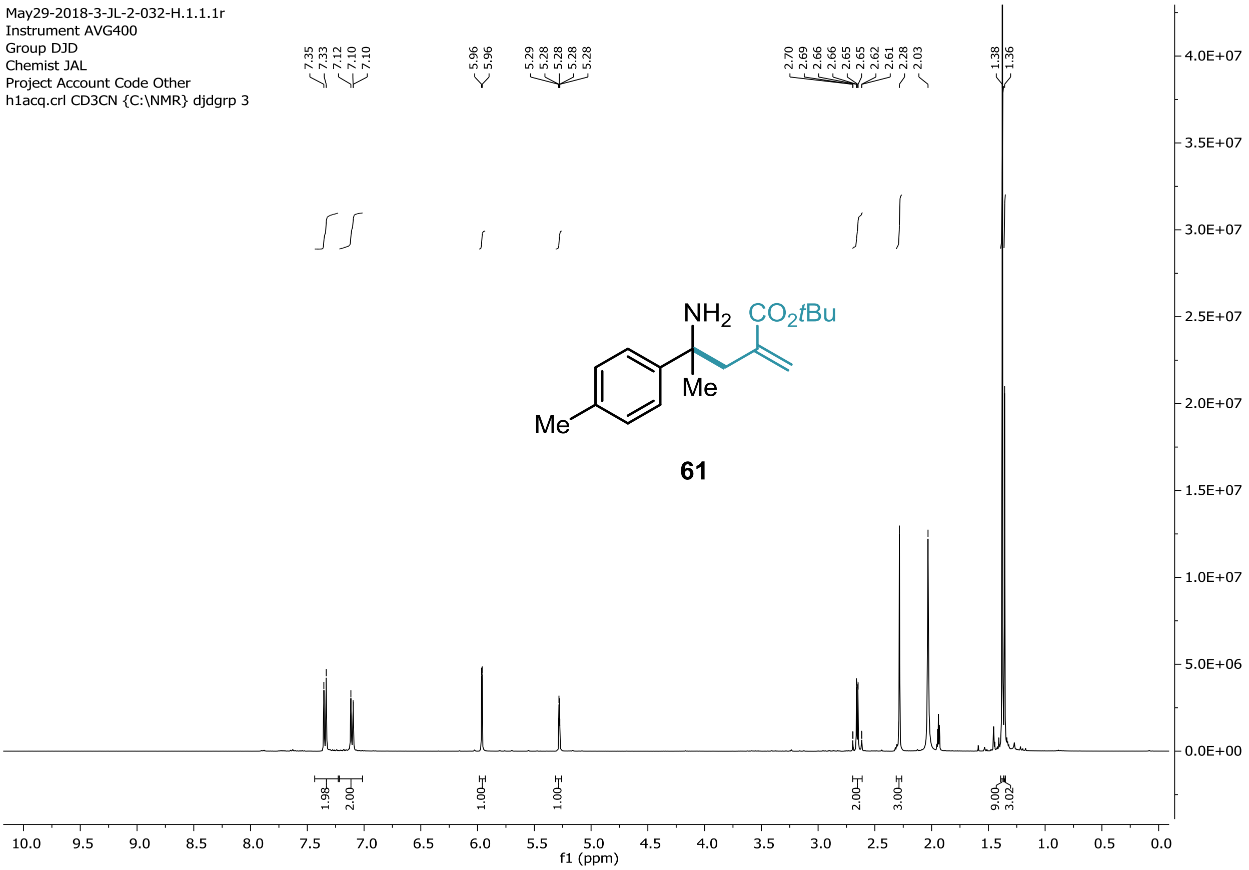
May29-2018-3-JL-2-032-H.1.1.1r
Instrument AVG400
Group DJD
Chemist JAL
Project Account Code Other
h1acq.crl CD3CN {C:\NMR} djdgrp 3
7.35
7.33
7.12
7.10
7.10
5.96
5.96
5.29
5.28
5.28
5.28
5.28
2.70
2.69
2.66
2.66
2.65
2.65
2.62
2.61
2.28
2.03
1.38
1.36
NH2
CO2tBu
Me
Me
61
1.98
2.00
1.00
1.00
2.00
3.00
9.00
3.02
f1 (ppm)

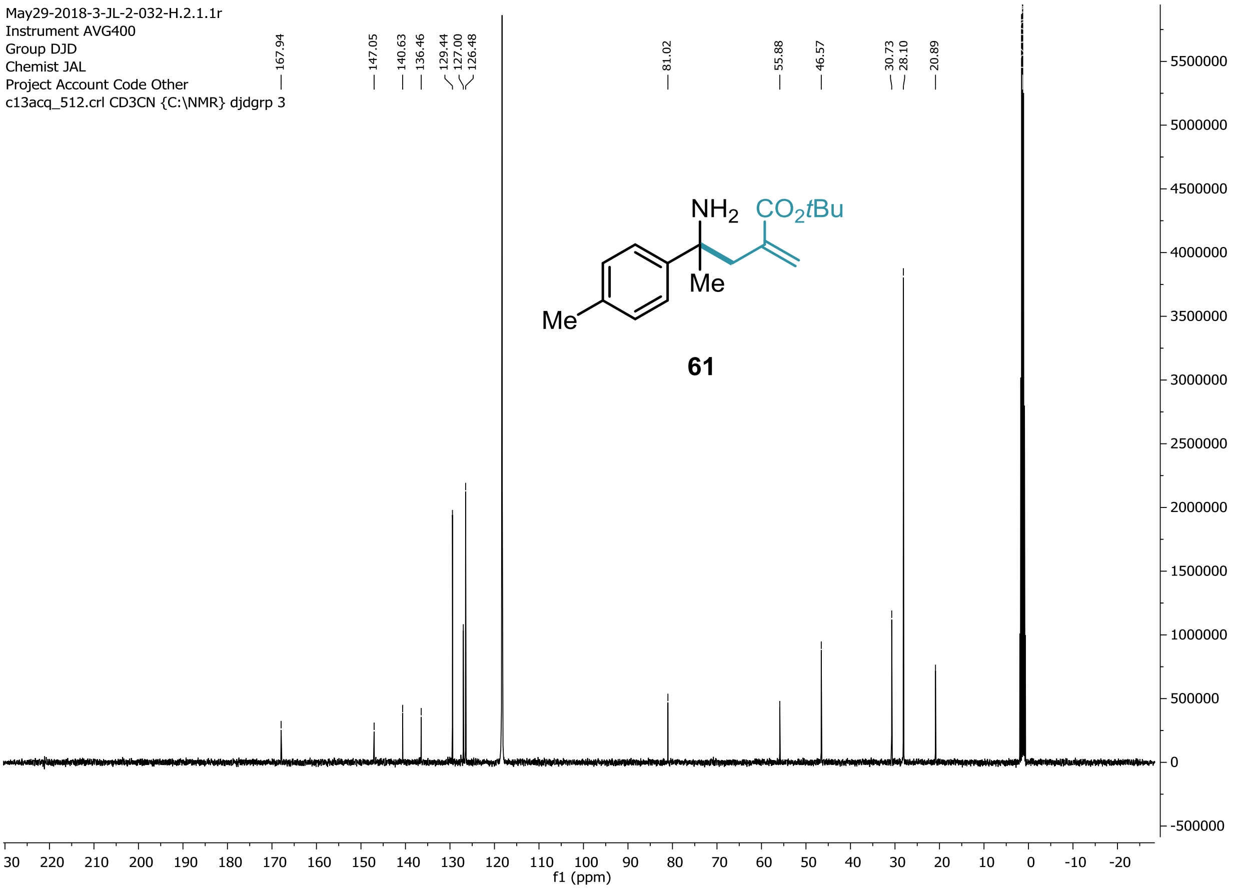
May29-2018-3-JL-2-032-H.2.1.1r
Instrument AVG400
Group DJD
Chemist JAL
Project Account Code Other
c13acq_512.crl CD3CN {C:\NMR} djdgrp 3
167.94
147.05
140.63
136.46
129.44
127.00
126.48
81.02
55.88
46.57
30.73
28.10
20.89
NH2
CO2tBu
Me
Me
61
f1 (ppm)

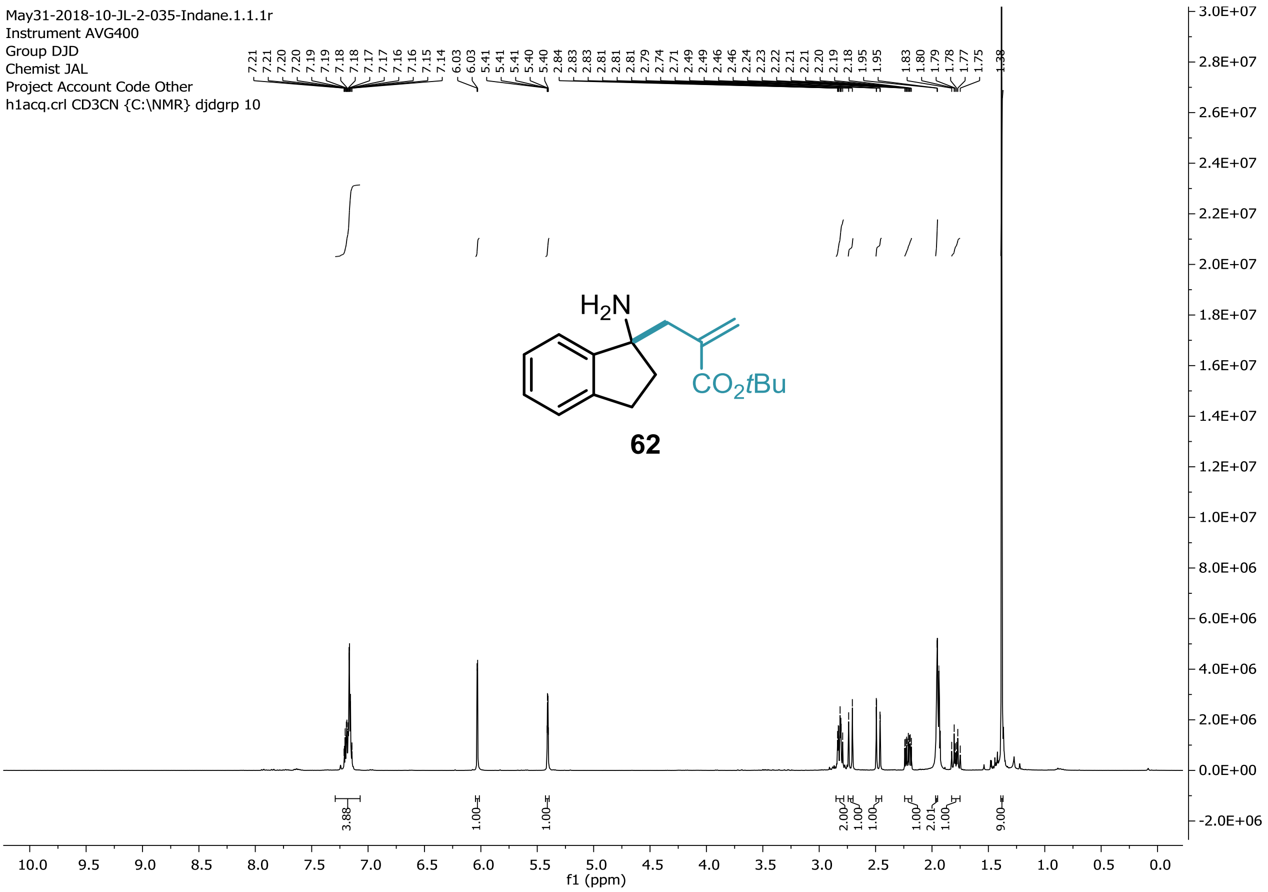
May31-2018-10-JL-2-035-Indane.1.1.1r
Instrument AVG400
Group DJD
Chemist JAL
Project Account Code Other
h1acq.crl CD3CN {C:\NMR} djdgrp 10
7.21
7.21
7.20
7.20
7.19
7.19
7.18
7.18
7.17
7.17
7.16
7.16
7.15
7.14
6.03
6.03
5.41
5.41
5.41
5.40
5.40
2.84
2.83
2.83
2.81
2.81
2.79
2.74
2.71
2.49
2.49
2.46
2.46
2.24
2.23
2.22
2.22
2.21
2.21
2.20
2.19
2.18
1.95
1.95
1.83
1.80
1.79
1.78
1.77
1.75
1.38
H2N
CO2tBu
62
3.88
1.00
1.00
2.00
1.00
1.00
1.00
2.01
1.00
9.00
f1 (ppm)

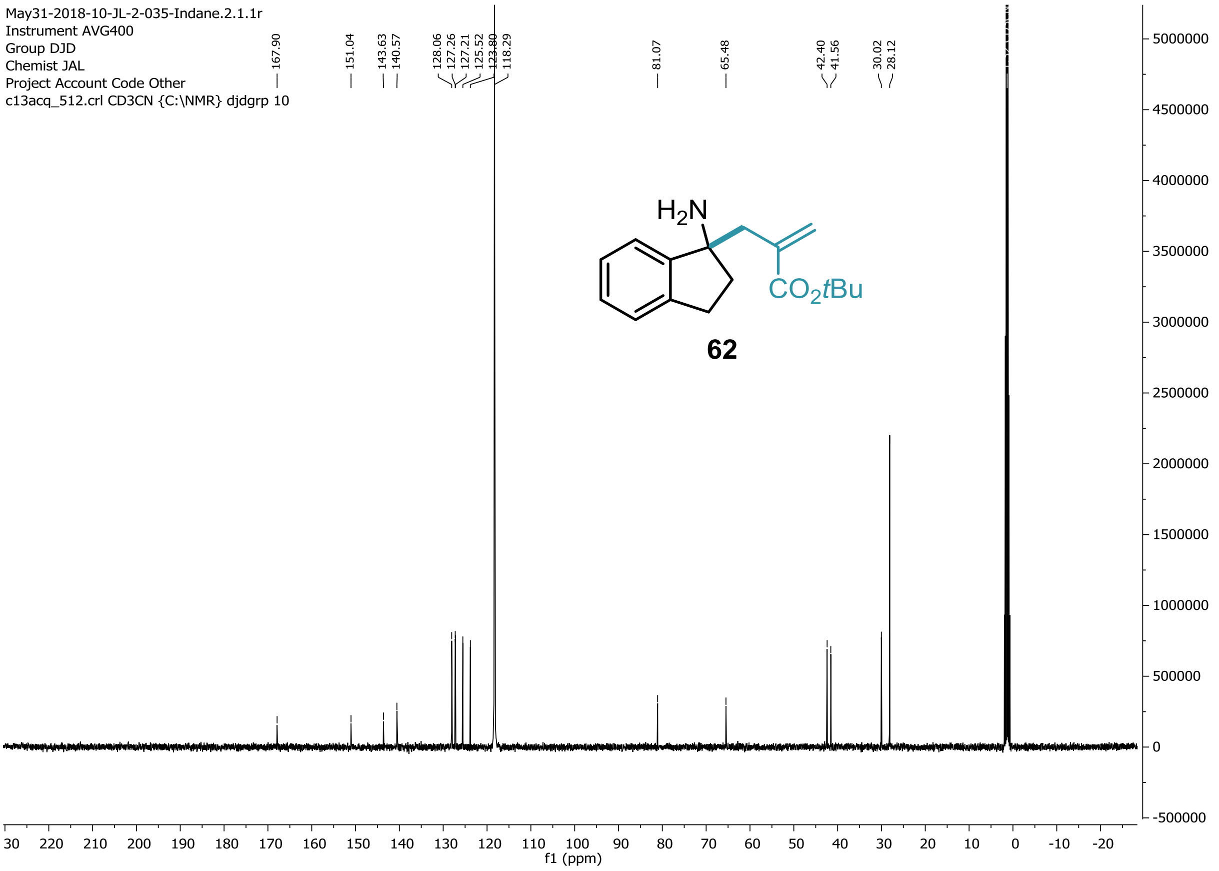
May31-2018-10-JL-2-035-Indane.2.1.1r
Instrument AVG400
Group DJD
Chemist JAL
Project Account Code Other
c13acq_512.crl CD3CN {C:\NMR} djdgrp 10
167.90
151.04
143.63
140.57
128.06
127.26
127.21
125.52
123.80
118.29
81.07
65.48
42.40
41.56
30.02
28.12
H2N
CO2tBu
62
f1 (ppm)

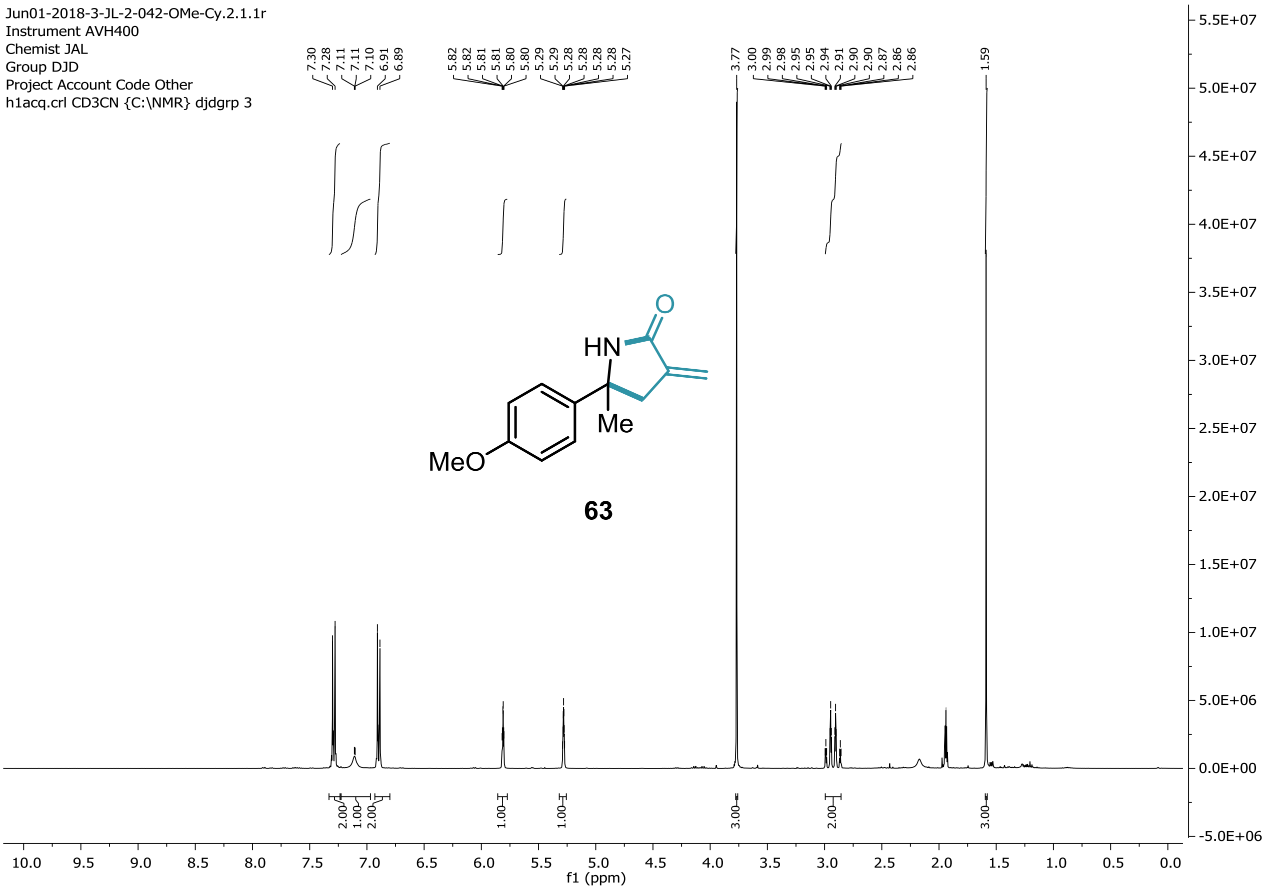
Jun01-2018-3-JL-2-042-OMe-Cy.2.1.1r
Instrument AVH400
Chemist JAL
Group DJD
Project Account Code Other
h1acq.crl CD3CN {C:\NMR} djdgrp 3
7.30
7.28
7.11
7.11
7.10
6.91
6.89
5.82
5.82
5.81
5.81
5.80
5.80
5.29
5.29
5.28
5.28
5.28
5.28
5.27
3.77
3.00
2.99
2.98
2.95
2.95
2.94
2.91
2.90
2.90
2.87
2.86
2.86
1.59
HN
O
Me
MeO
63
2.00
1.00
2.00
1.00
1.00
3.00
2.00
3.00
f1 (ppm)

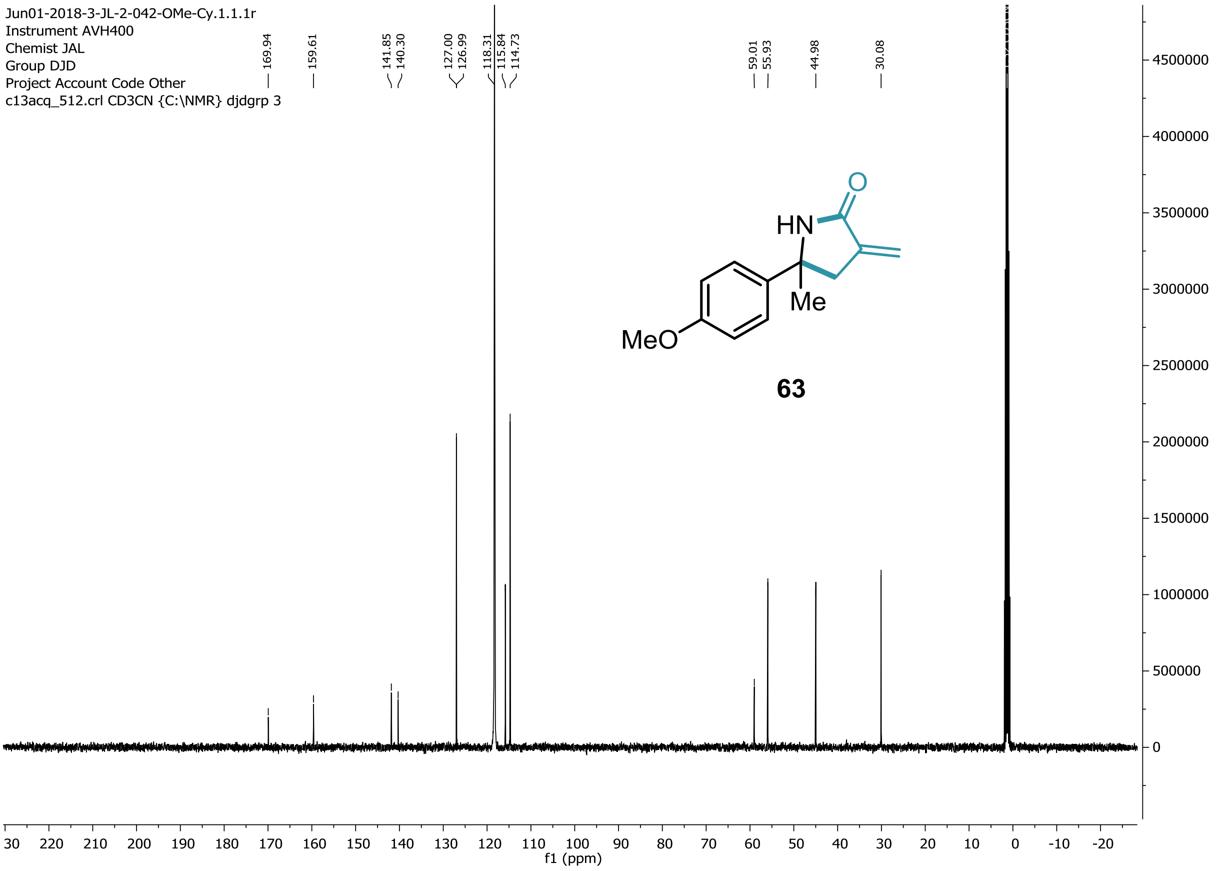
Jun01-2018-3-JL-2-042-OMe-Cy.1.1.1r
Instrument AVH400
Chemist JAL
Group DJD
Project Account Code Other
c13acq_512.crl CD3CN {C:\NMR} djdgrp 3
169.94
159.61
141.85
140.30
127.00
126.99
118.31
115.84
114.73
59.01
55.93
44.98
30.08
HN
O
Me
MeO
63
f1 (ppm)

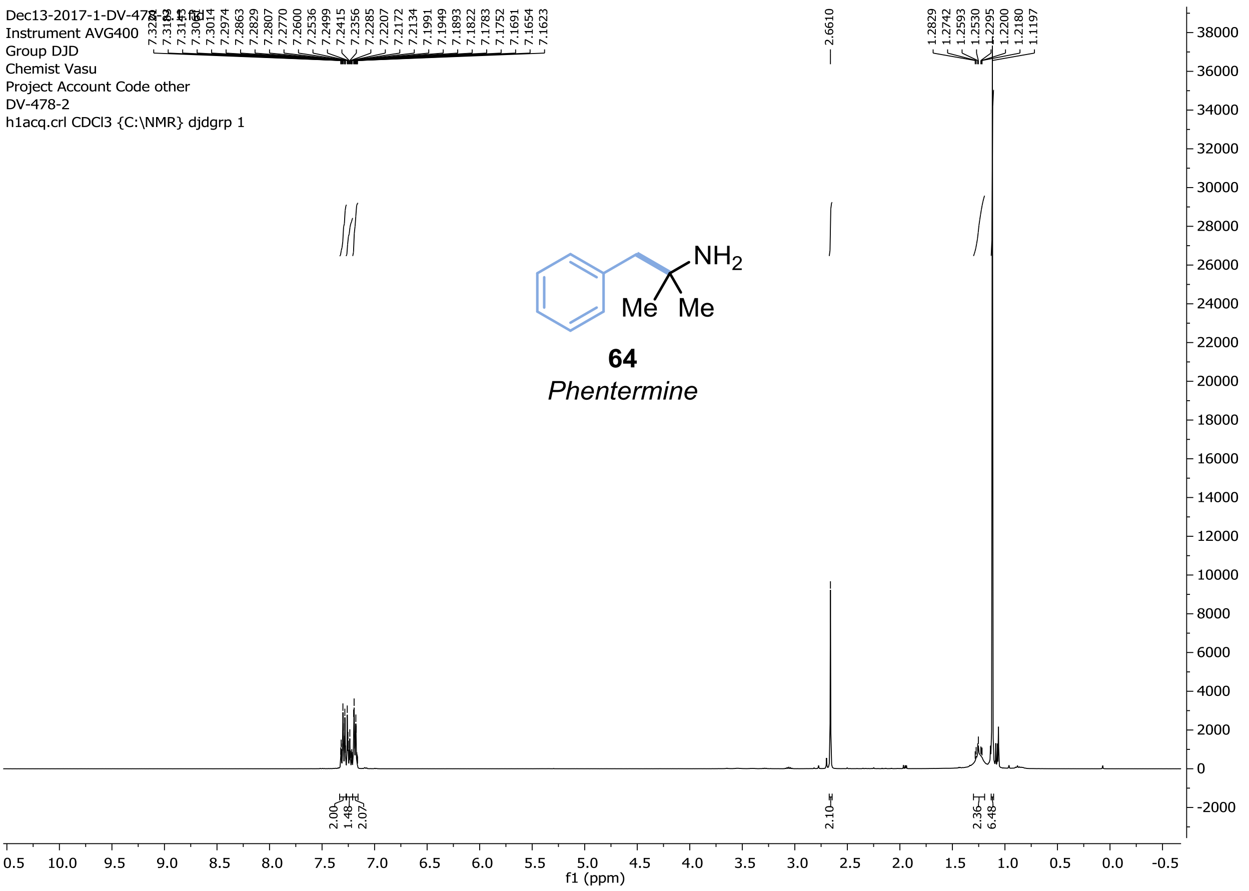
Dec13-2017-1-DV-478-2.1.fid
Instrument AVG400
Group DJD
Chemist Vasu
Project Account Code other
DV-478-2
h1acq.crl CDCl3 {C:\NMR} djdgrp 1
2.6610
64
Phentermine
f1 (ppm)

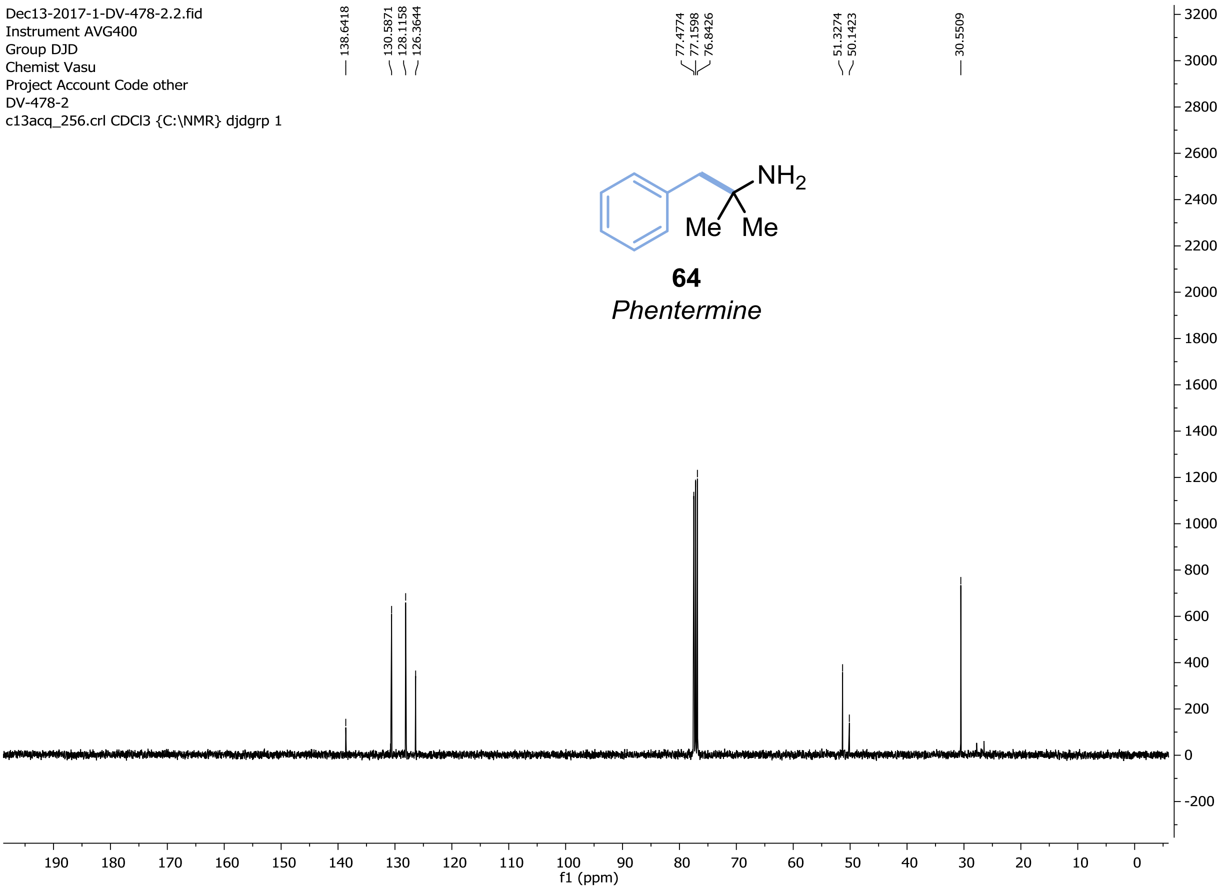
Dec13-2017-1-DV-478-2.2.fid
Instrument AVG400
Group DJD
Chemist Vasu
Project Account Code other
DV-478-2
c13acq_256.crl CDCl3 {C:\NMR} djdgrp 1
138.6418
130.5871
128.1158
126.3644
77.4774
77.1598
76.8426
51.3274
50.1423
30.5509
64
Phentermine
f1 (ppm)

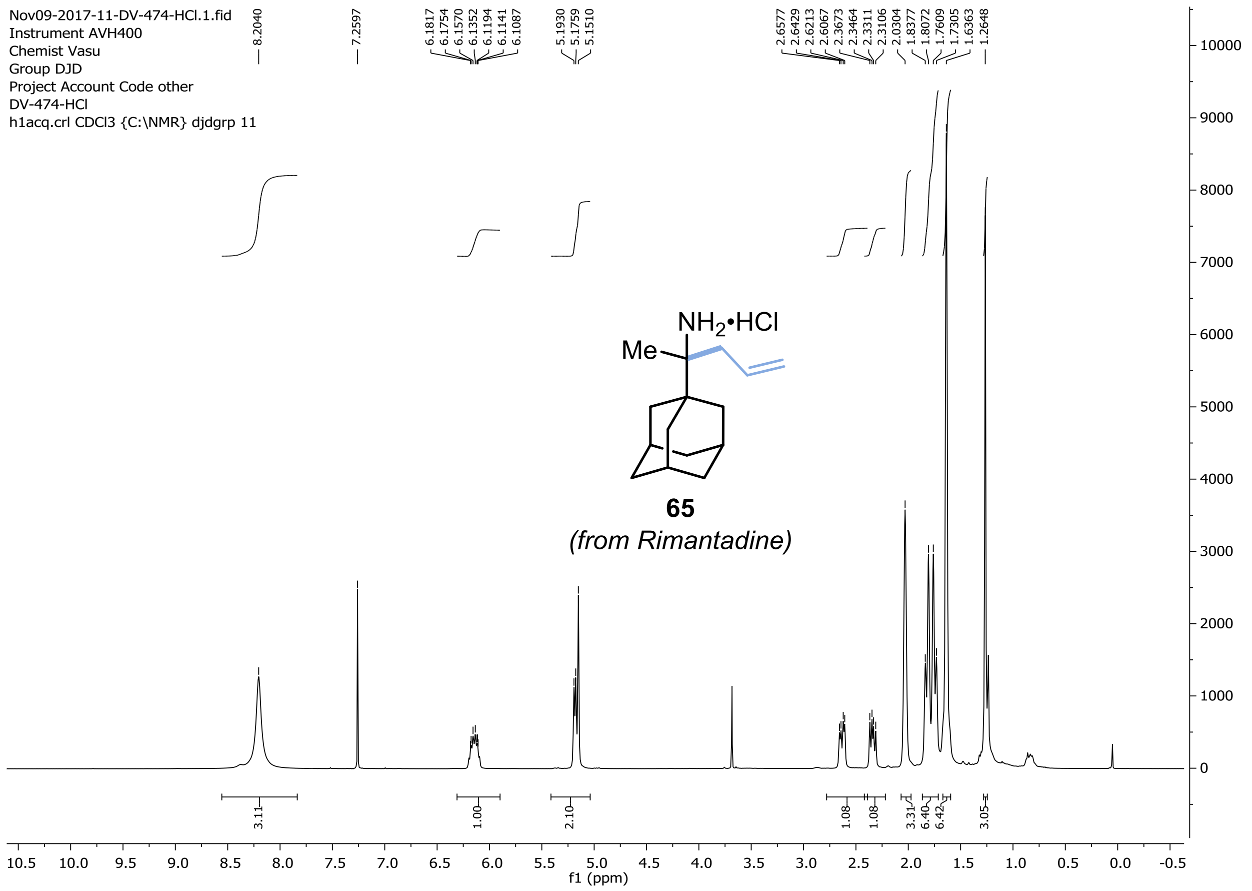
Nov09-2017-11-DV-474-HCl.1.fid
Instrument AVH400
Chemist Vasu
Group DJD
Project Account Code other
DV-474-HCl
h1acq.crl CDCl3 {C:\NMR} djdgrp 11
8.2040
7.2597
6.1817 6.1754 6.1570 6.1352 6.1194 6.1141 6.1087
5.1930 5.1759 5.1510
2.6577 2.6429 2.6213 2.6067 2.3673 2.3464 2.3311 2.3106 2.0304 1.8377 1.8072 1.7609 1.7305 1.6363 1.2648
NH2•HCl
Me
65
(from Rimantadine)
3.11
1.00
2.10
1.08
1.08
3.31
6.40
6.42
3.05
f1 (ppm)

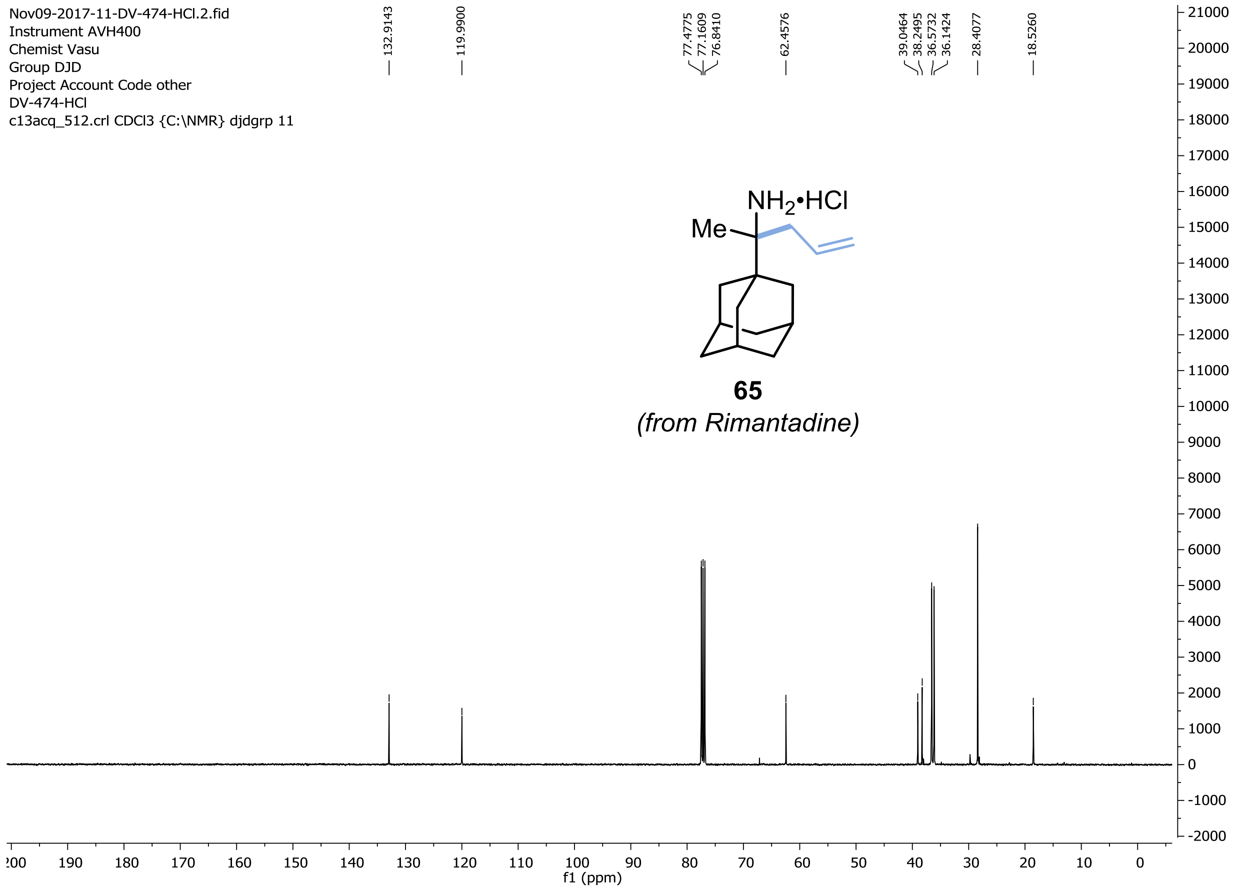
Nov09-2017-11-DV-474-HCl.2.fid
Instrument AVH400
Chemist Vasu
Group DJD
Project Account Code other
DV-474-HCl
c13acq_512.crl CDCl3 {C:\NMR} djdgrp 11
132.9143
119.9900
77.4775 77.1609 76.8410
62.4576
39.0464 38.2495 36.5732 36.1424
28.4077
18.5260
NH2•HCl
Me
65
(from Rimantadine)
f1 (ppm)

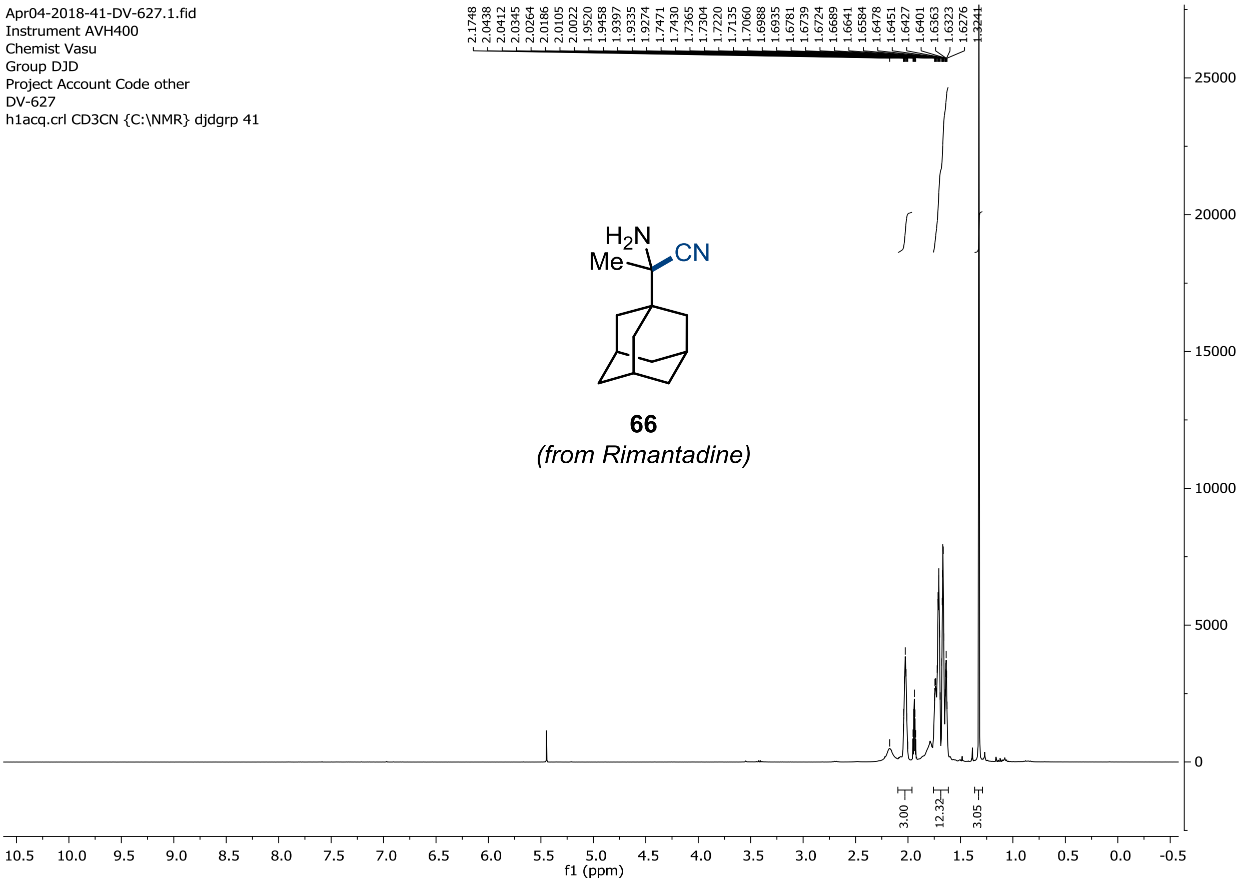
Apr04-2018-41-DV-627.1.fid
Instrument AVH400
Chemist Vasu
Group DJD
Project Account Code other
DV-627
h1acq.crl CD3CN {C:\NMR} djdgrp 41
2.1748
2.0438
2.0412
2.0345
2.0264
2.0186
2.0105
2.0022
1.9520
1.9458
1.9397
1.9335
1.9274
1.7471
1.7430
1.7365
1.7304
1.7220
1.7135
1.7060
1.6988
1.6935
1.6781
1.6739
1.6724
1.6689
1.6641
1.6584
1.6478
1.6451
1.6427
1.6401
1.6363
1.6323
1.6276
1.3241
H2N
Me
CN
66
(from Rimantadine)
3.00
12.32
3.05
f1 (ppm)

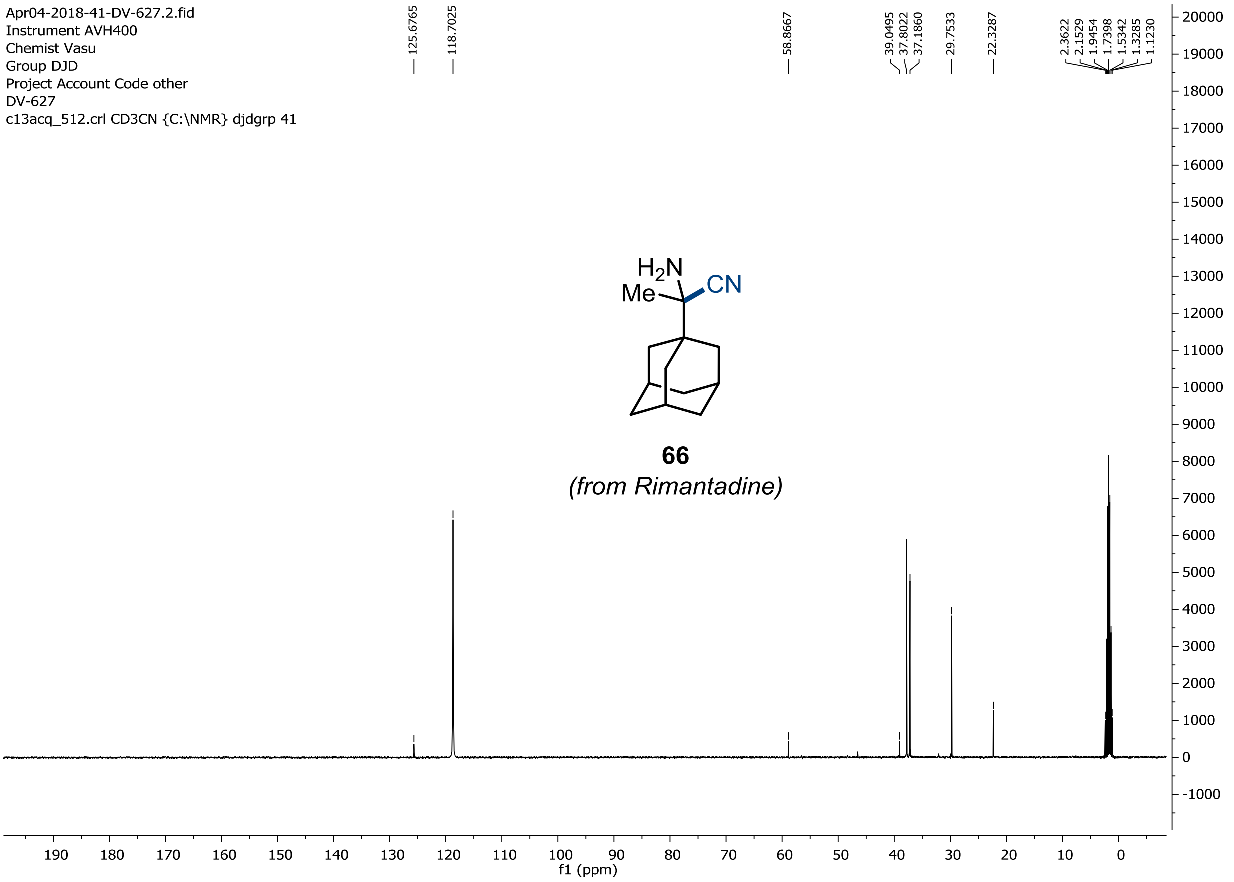
Apr04-2018-41-DV-627.2.fid
Instrument AVH400
Chemist Vasu
Group DJD
Project Account Code other
DV-627
c13acq_512.crl CD3CN {C:\NMR} djdgrp 41
125.6765
118.7025
58.8667
39.0495
37.8022
37.1860
29.7533
22.3287
2.3622
2.1529
1.9454
1.7398
1.5342
1.3285
1.1230
H2N
Me
CN
66
(from Rimantadine)
f1 (ppm)

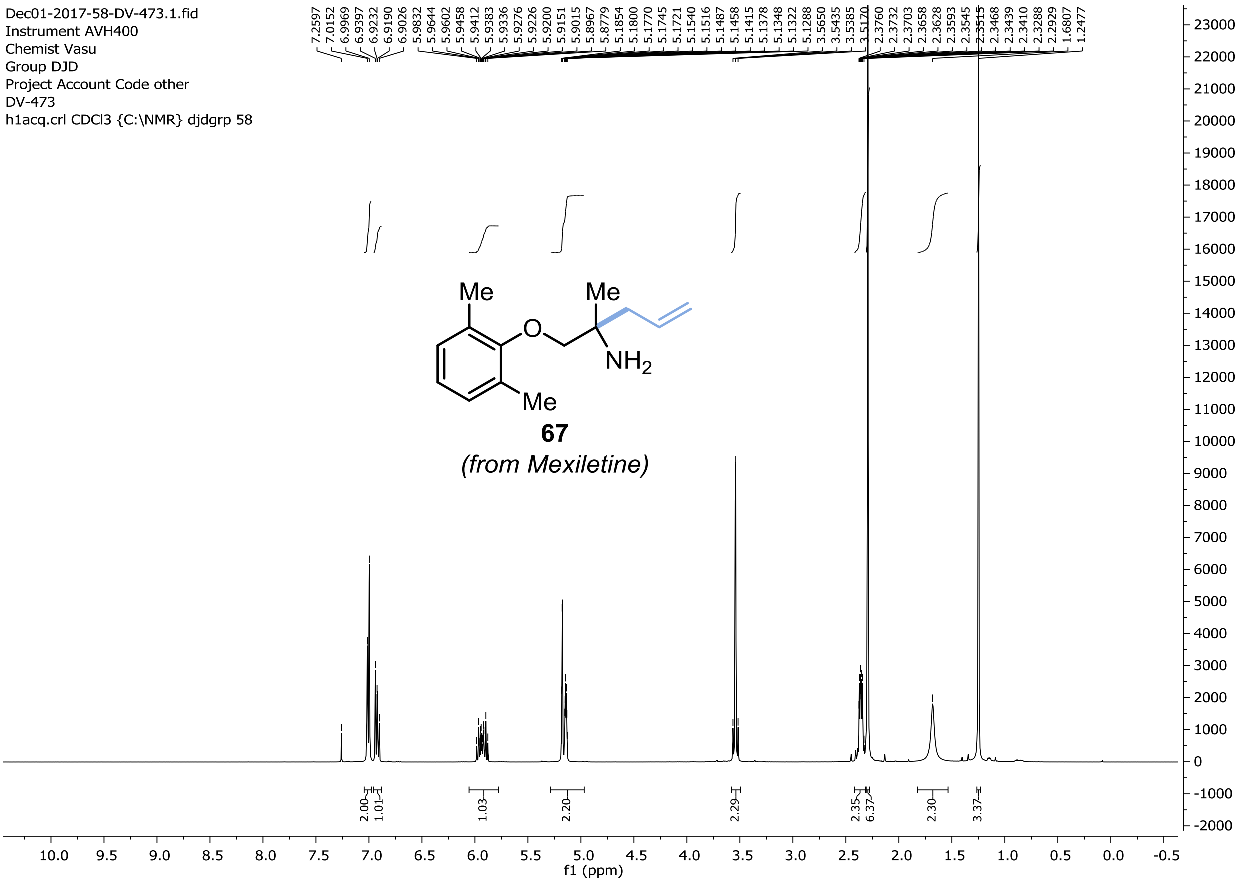
Dec01-2017-58-DV-473.1.fid
Instrument AVH400
Chemist Vasu
Group DJD
Project Account Code other
DV-473
h1acq.crl CDCl3 {C:\NMR} djdgrp 58
7.2597 7.0152 6.9969 6.9397 6.9232 6.9190 6.9026 5.9832 5.9644 5.9602 5.9458 5.9412 5.9383 5.9336 5.9276 5.9226 5.9200 5.9151 5.9015 5.8967 5.8779 5.1854 5.1800 5.1770 5.1745 5.1721 5.1540 5.1516 5.1487 5.1458 5.1415 5.1378 5.1348 5.1322 5.1288 3.5650 3.5435 3.5385 3.5170 2.3760 2.3732 2.3703 2.3658 2.3628 2.3593 2.3545 2.3515 2.3468 2.3439 2.3410 2.3288 2.2929 1.6807 1.2477
Me
Me
O
NH2
Me
67
(from Mexiletine)
2.00 1.01 1.03 2.20 2.29 2.35 6.37 2.30 3.37
f1 (ppm)

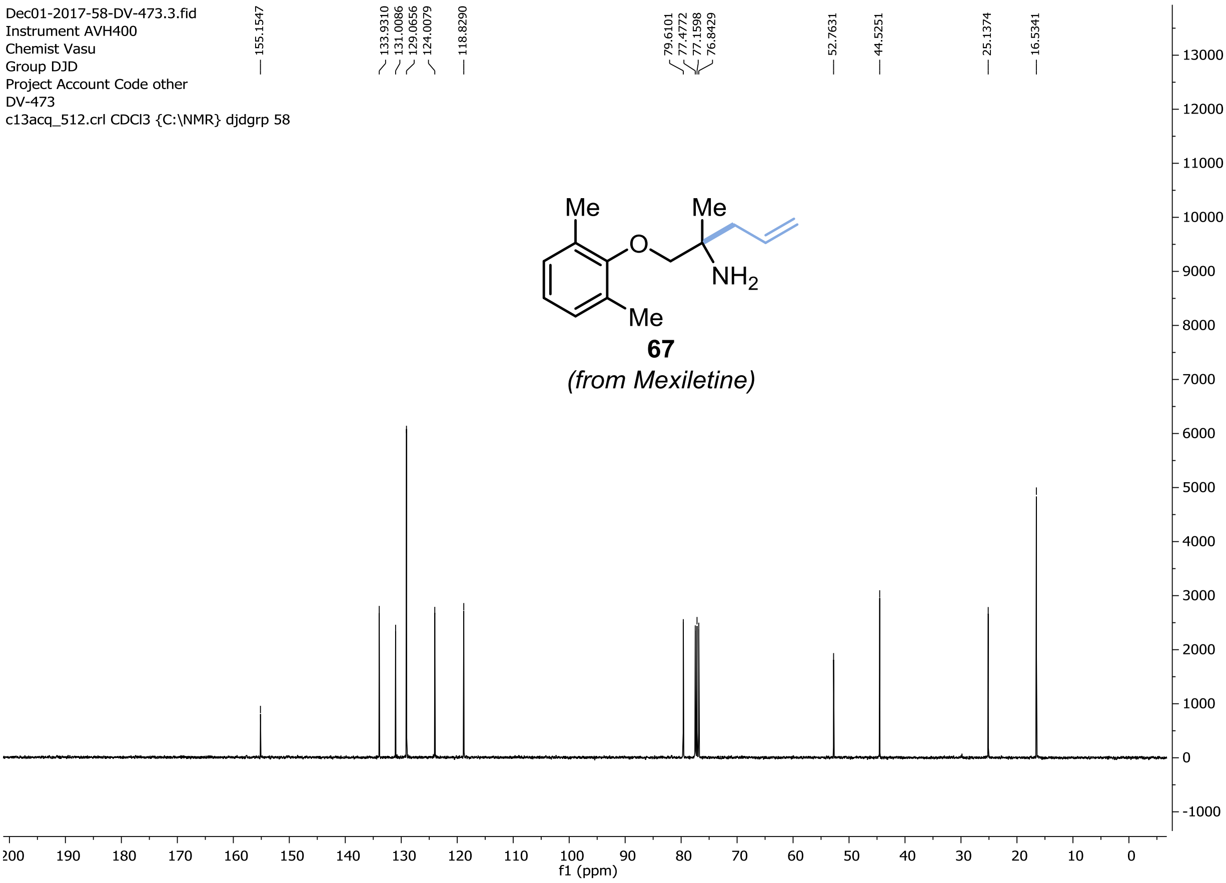
Dec01-2017-58-DV-473.3.fid
Instrument AVH400
Chemist Vasu
Group DJD
Project Account Code other
DV-473
c13acq_512.crl CDCl3 {C:\NMR} djdgrp 58
155.1547 133.9310 131.0086 129.0656 124.0079 118.8290 79.6101 77.4772 77.1598 76.8429 52.7631 44.5251 25.1374 16.5341
Me
Me
O
NH2
Me
67
(from Mexiletine)
f1 (ppm)

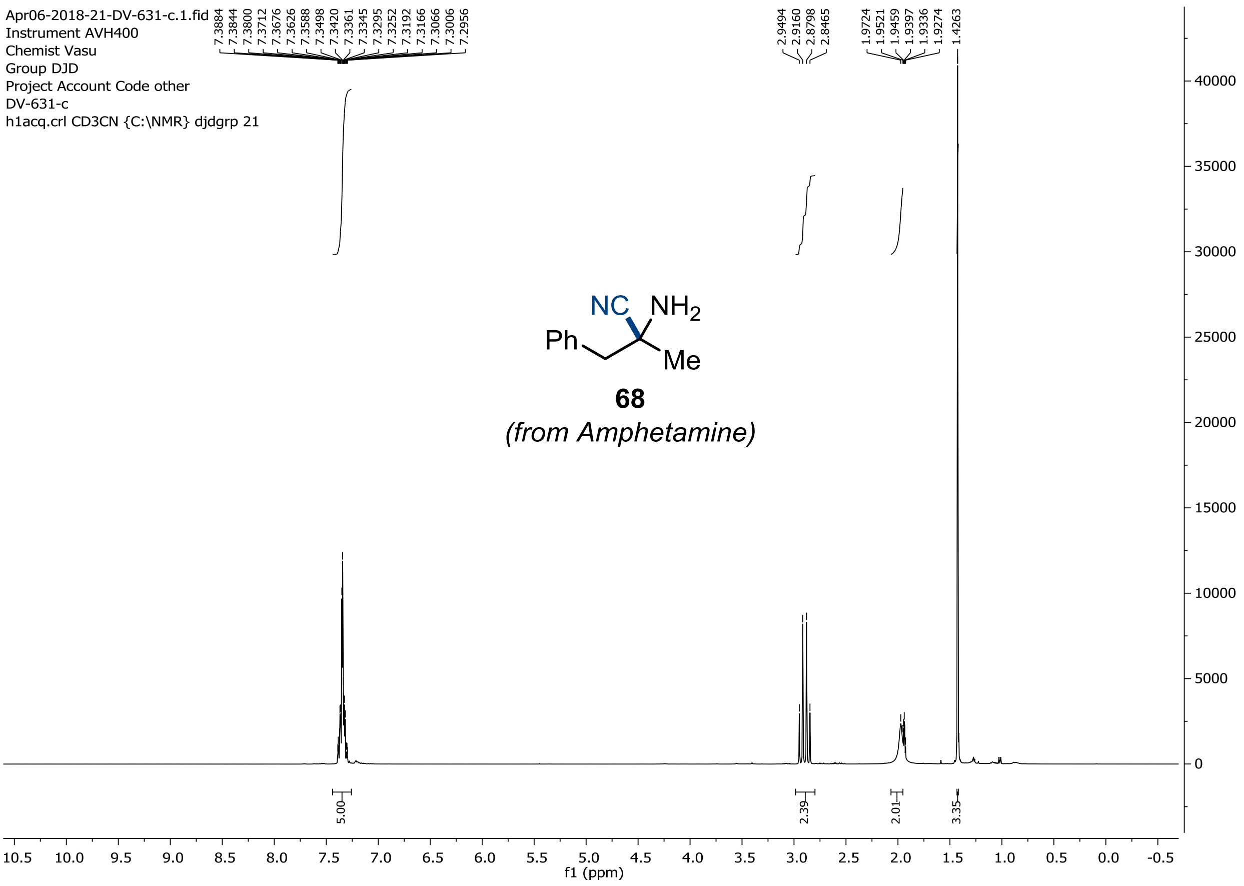
Apr06-2018-21-DV-631-c.1.fid
Instrument AVH400
Chemist Vasu
Group DJD
Project Account Code other
DV-631-c
h1acq.crl CD3CN {C:\NMR} djdgrp 21
NC NH2
Ph
Me
68
(from Amphetamine)
f1 (ppm)

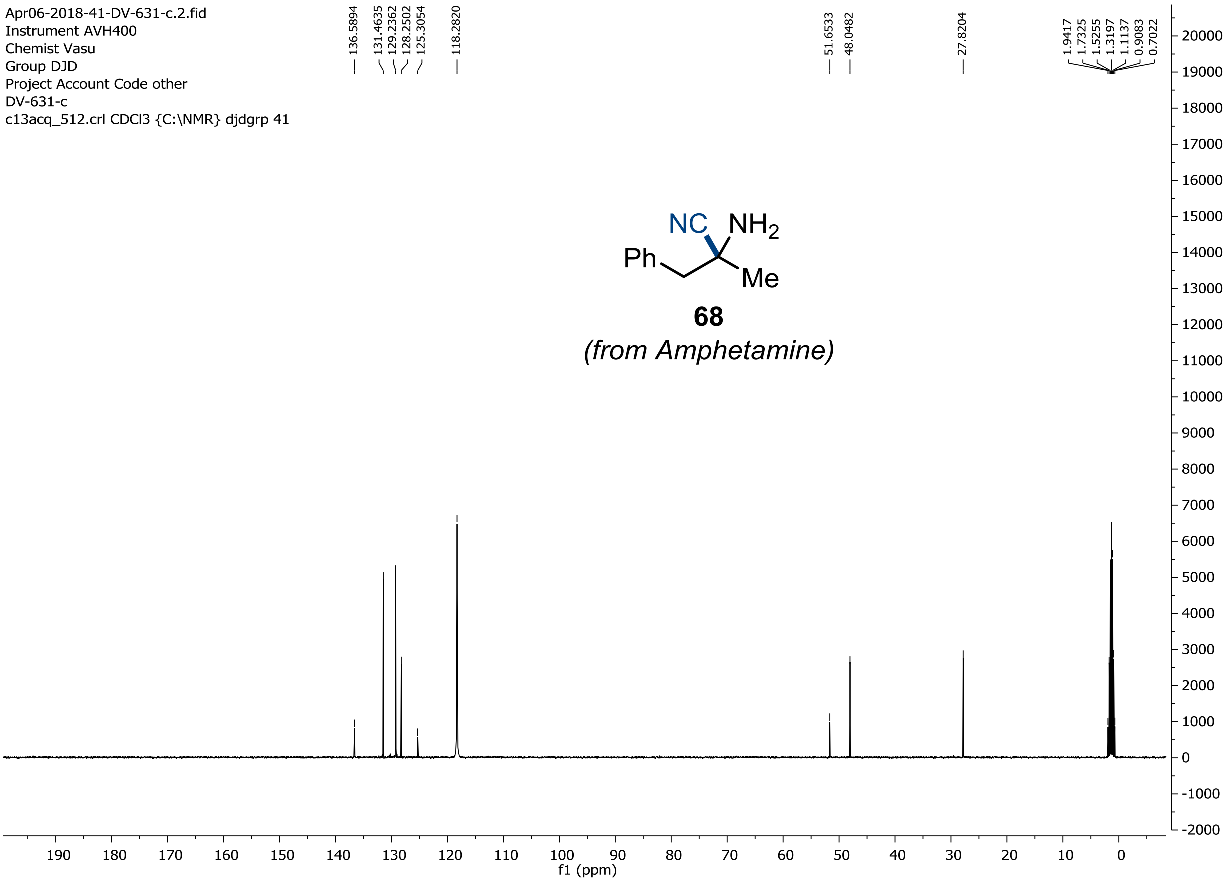
Apr06-2018-41-DV-631-c.2.fid
Instrument AVH400
Chemist Vasu
Group DJD
Project Account Code other
DV-631-c
c13acq_512.crl CDCl3 {C:\NMR} djdgrp 41
NC NH2
Ph
Me
68
(from Amphetamine)
f1 (ppm)

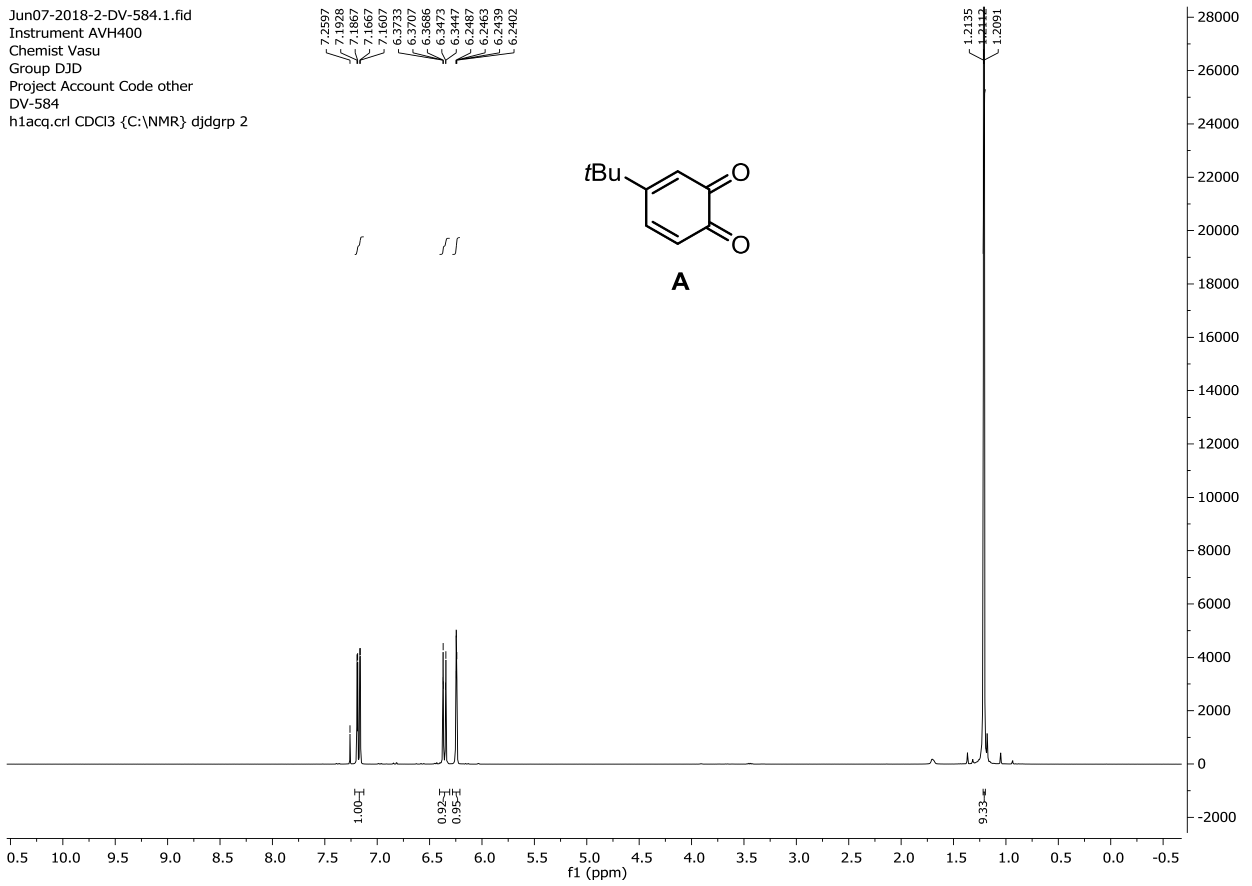
Jun07-2018-2-DV-584.1.fid
Instrument AVH400
Chemist Vasu
Group DJD
Project Account Code other
DV-584
h1acq.crl CDCl3 {C:\NMR} djdgrp 2
7.2597
7.1928
7.1867
7.1667
7.1607
6.3733
6.3707
6.3686
6.3473
6.3447
6.2487
6.2463
6.2439
6.2402
1.2135
1.2112
1.2091
tBu
O
O
A
1.00
0.92
0.95
9.33
f1 (ppm)

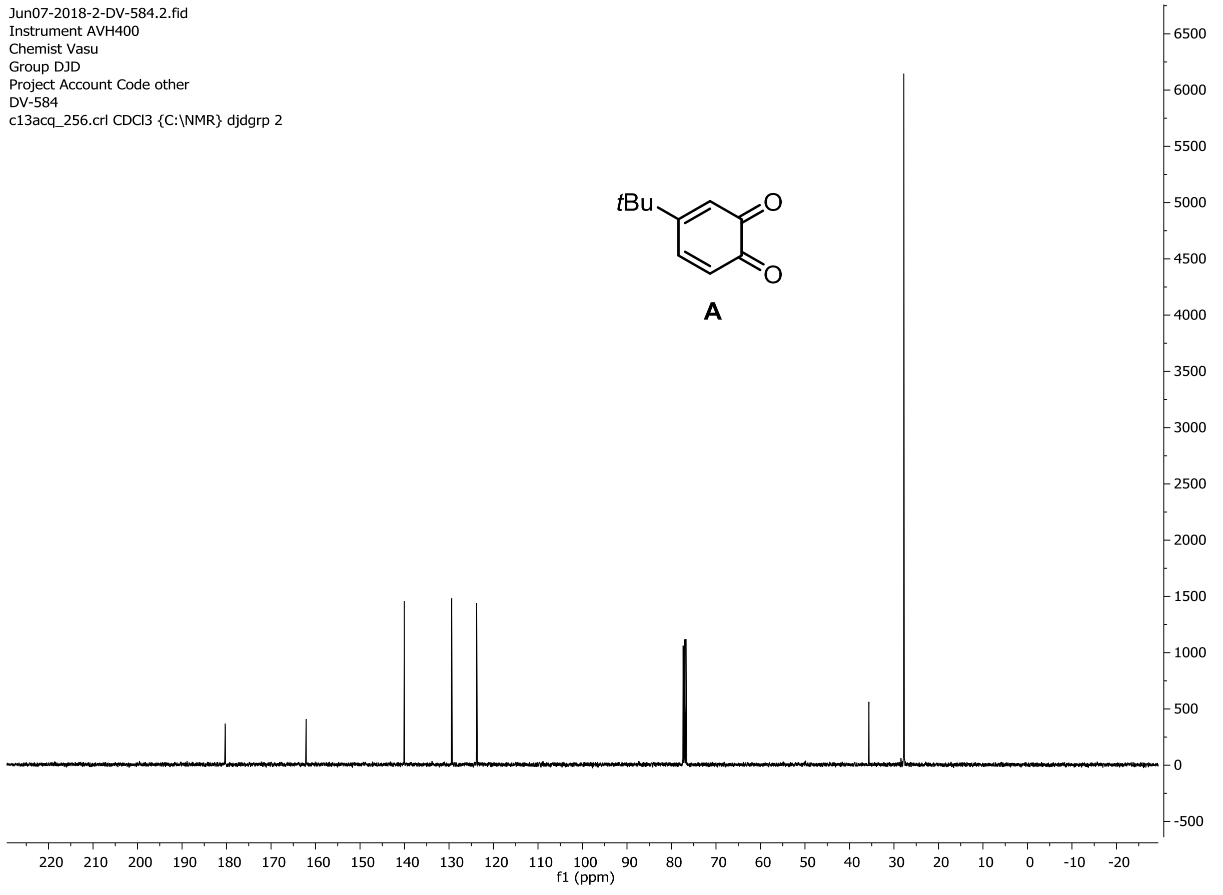
Jun07-2018-2-DV-584.2.fid
Instrument AVH400
Chemist Vasu
Group DJD
Project Account Code other
DV-584
c13acq_256.crl CDCl3 {C:\NMR} djdgrp 2
tBu
O
O
A
f1 (ppm)

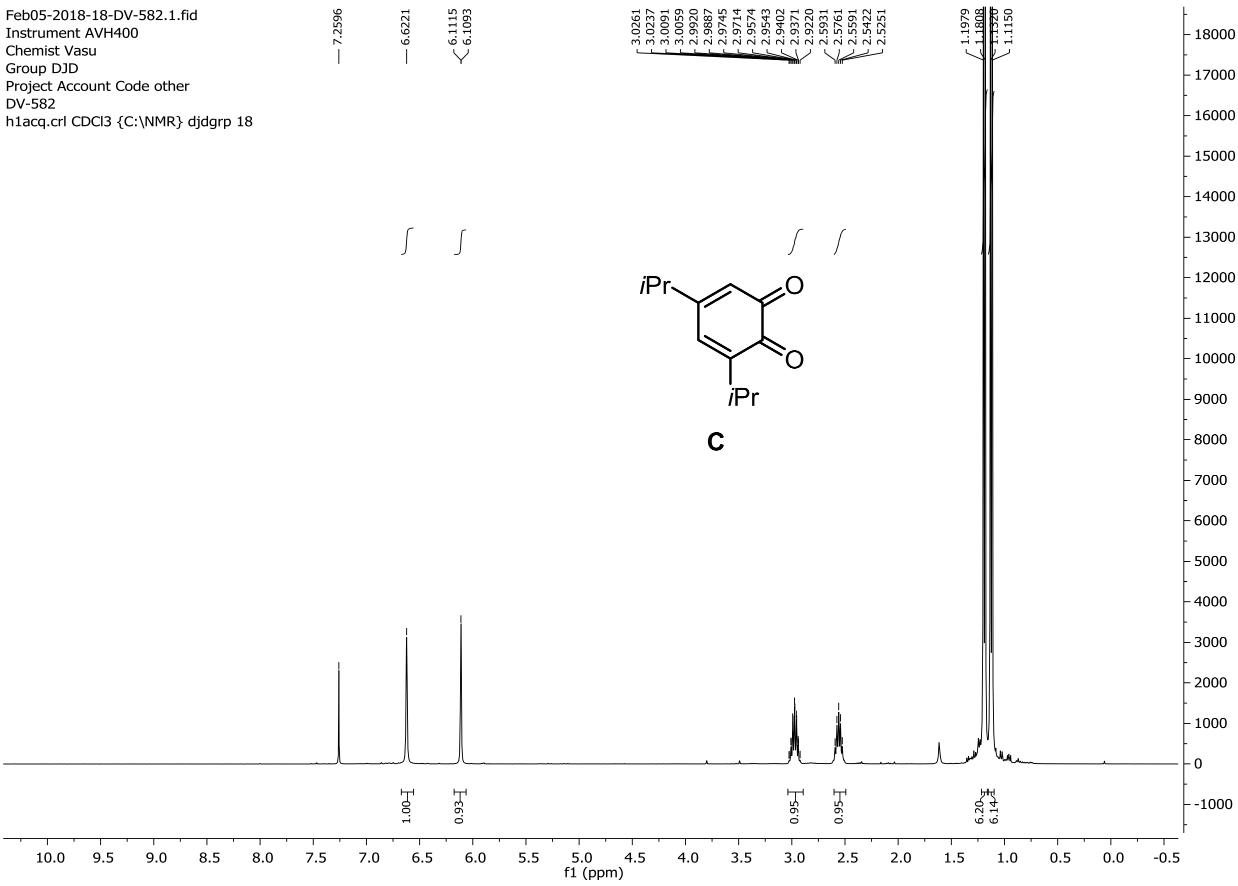
Feb05-2018-18-DV-582.1.fid
Instrument AVH400
Chemist Vasu
Group DJD
Project Account Code other
DV-582
h1acq.crl CDCl3 {C:\NMR} djdgrp 18
7.2596
6.6221
6.1115
6.1093
3.0261
3.0237
3.0091
3.0059
2.9920
2.9887
2.9745
2.9714
2.9574
2.9543
2.9402
2.9371
2.9220
2.5931
2.5761
2.5591
2.5422
2.5251
1.1979
1.1808
1.1320
1.1150
iPr
O
O
iPr
C
1.00
0.93
0.95
0.95
6.20
6.14
f1 (ppm)

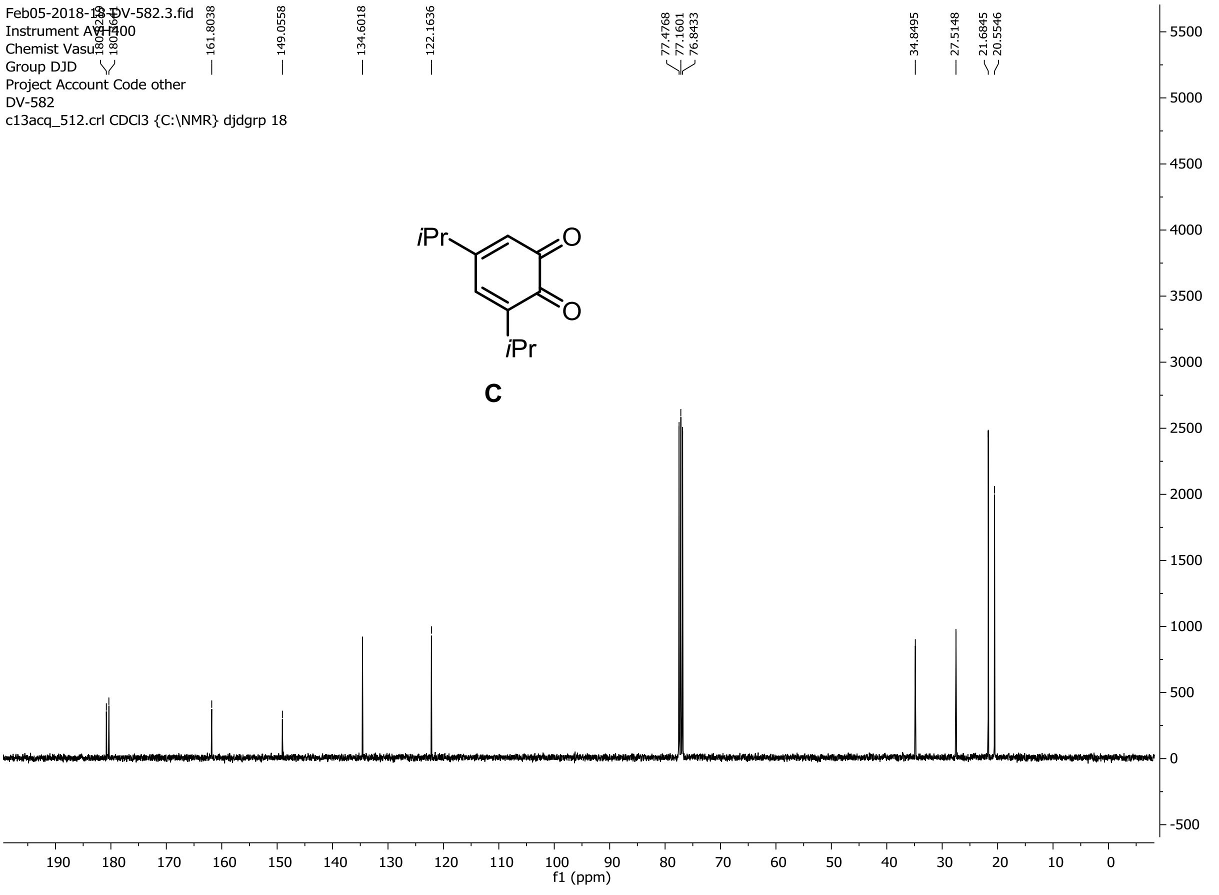
Feb05-2018-18-DV-582.3.fid
Instrument AVH400
Chemist Vasu
Group DJD
Project Account Code other
DV-582
c13acq_512.crl CDCl3 {C:\NMR} djdgrp 18
161.8038
149.0558
134.6018
122.1636
77.4768
77.1601
76.8433
34.8495
27.5148
21.6845
20.5546
iPr
O
O
iPr
C
f1 (ppm)

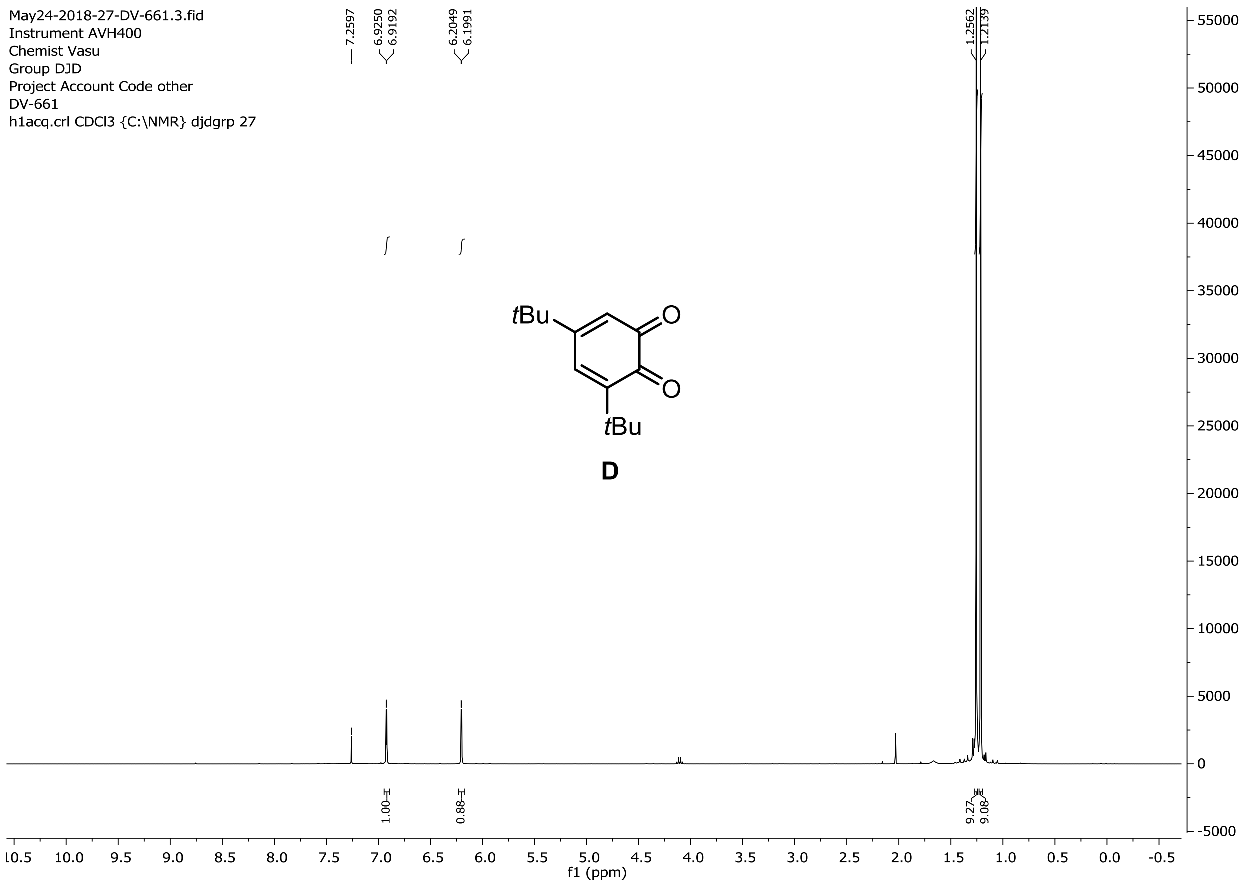
May24-2018-27-DV-661.3.fid
Instrument AVH400
Chemist Vasu
Group DJD
Project Account Code other
DV-661
h1acq.crl CDCl3 {C:\NMR} djdgrp 27
7.2597
6.9250
6.9192
6.2049
6.1991
1.2562
1.2139
tBu
O
O
tBu
D
1.00
0.88
9.27
9.08
f1 (ppm)

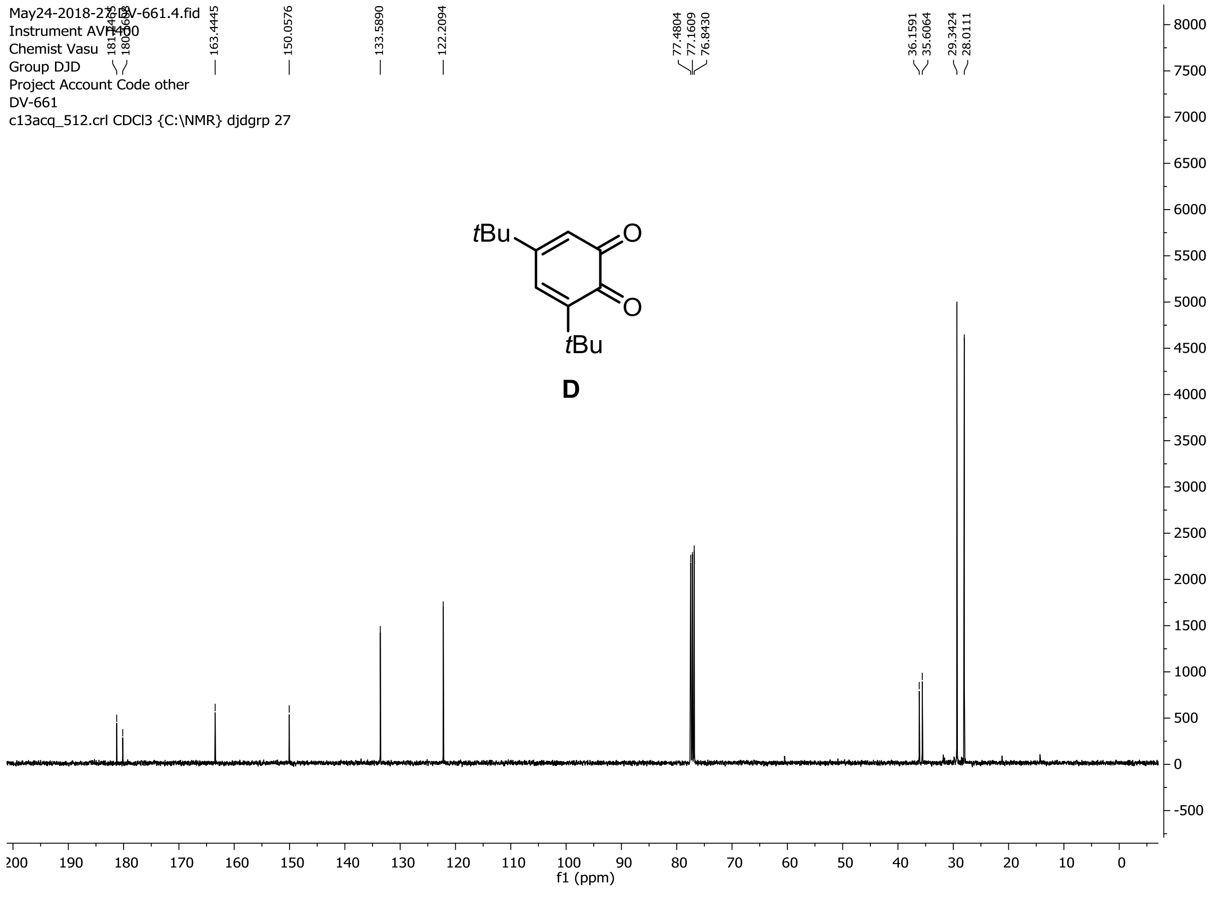
May24-2018-27-DV-661.4.fid
Instrument AVH400
Chemist Vasu
Group DJD
Project Account Code other
DV-661
c13acq_512.crl CDCl3 {C:\NMR} djdgrp 27
163.4445
150.0576
133.5890
122.2094
77.4804
77.1609
76.8430
36.1591
35.6064
29.3424
28.0111
tBu
O
O
tBu
D
f1 (ppm)

7: X- ray structure and data for compound 57:

Table S4: Crystal data and structure refinement for compound 57

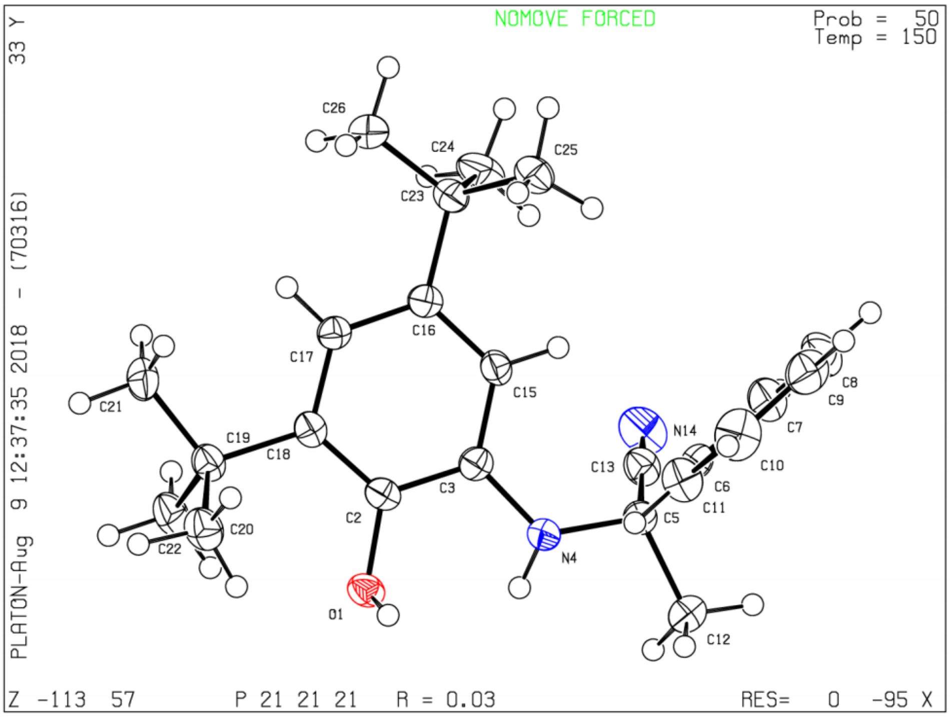

Table 1

Experimental details

Crystal data	
Chemical formula	$C_{23}H_{30}N_2O$
M_r	350.50
Crystal system, space group	Orthorhombic, $P2_12_12_1$
Temperature (K)	150
a, *b*, *c* (Å)	9.8225 (2), 10.4555 (2), 19.9670 (4)
V (Å^3)	2050.59 (4)
Z	4
Radiation type	Cu *K*α
μ (mm^{-1})	0.53
Crystal size (mm)	0.20 × 0.15 × 0.05
Data collection	
Diffractometer	Unknown
Absorption correction	Multi-scan<br>*DENZO*/*SCALEPACK* (Otwinowski & Minor, 1997)
T_{min}, T_{max}	0.86, 0.97
No. of measured, independent and observed [$I > 2.0\sigma(I)$] reflections	14910, 4230, 3970
R_{int}	0.032

$(\sin \theta/\lambda)_{max}$ (Å^{-1})	0.630
Refinement	
$R[F^2 > 2\sigma(F^2)]$, $wR(F^2)$, S	0.034, 0.087, 1.03
No. of reflections	4230
No. of parameters	326
No. of restraints	116
H-atom treatment	Only H-atom coordinates refined
$\Delta\rho_{max}$, $\Delta\rho_{min}$ (e Å^{-3})	0.22, −0.16
Absolute structure	Flack (1983), 1797 Friedel-pairs
Absolute structure parameter	−0.1 (2)

Computer programs: USER DEFINED DATA COLLECTION, USER DEFINED *CELL* REFINEMENT, USER DEFINED DATA REDUCTION, *SUPERFLIP* (Palatinus & Chapuis, 2007), *CRYSTALS* (Betteridge *et al.*, 2003), *CAMERON* (Watkin *et al.*, 1996).

Table 2

Selected geometric parameters (Å, º)

O1—C2	1.3866 (16)	C10—C11	1.392 (2)
C2—C3	1.4077 (18)	C13—N14	1.136 (2)
C2—C18	1.3930 (18)	C15—C16	1.3958 (18)
C3—N4	1.3990 (17)	C16—C17	1.3882 (18)
C3—C15	1.3893 (18)	C16—C23	1.5398 (18)
N4—C5	1.4447 (16)	C17—C18	1.4086 (18)
C5—C6	1.5304 (18)	C18—C19	1.5405 (17)
C5—C12	1.5385 (18)	C19—C20	1.539 (2)
C5—C13	1.5003 (18)	C19—C21	1.533 (2)
C6—C7	1.3841 (19)	C19—C22	1.538 (2)
C6—C11	1.388 (2)	C23—C24	1.534 (2)
C7—C8	1.385 (2)	C23—C25	1.537 (2)
C8—C9	1.384 (3)	C23—C26	1.534 (2)
C9—C10	1.376 (3)		
O1—C2—C3	117.05 (11)	C5—C13—N14	178.10 (15)
O1—C2—C18	121.91 (11)	C3—C15—C16	120.67 (12)
C3—C2—C18	120.99 (11)	C15—C16—C17	118.46 (12)
C2—C3—N4	116.27 (11)	C15—C16—C23	118.42 (11)
C2—C3—C15	119.78 (12)	C17—C16—C23	123.11 (11)
N4—C3—C15	123.93 (12)	C16—C17—C18	122.71 (12)
C3—N4—C5	124.04 (11)	C17—C18—C2	117.39 (11)
N4—C5—C6	112.94 (11)	C17—C18—C19	121.63 (12)
N4—C5—C12	107.65 (11)	C2—C18—C19	120.98 (11)
C6—C5—C12	108.96 (11)	C18—C19—C20	110.78 (11)
N4—C5—C13	110.23 (11)	C18—C19—C21	111.43 (11)

C6—C5—C13	109.61 (11)	C20—C19—C21	107.07 (12)
C12—C5—C13	107.27 (11)	C18—C19—C22	109.77 (11)
C5—C6—C7	121.42 (12)	C20—C19—C22	109.56 (12)
C5—C6—C11	119.01 (12)	C21—C19—C22	108.14 (12)
C7—C6—C11	119.35 (13)	C16—C23—C24	109.40 (11)
C6—C7—C8	120.44 (14)	C16—C23—C25	109.66 (11)
C7—C8—C9	120.28 (15)	C24—C23—C25	108.87 (12)
C8—C9—C10	119.47 (15)	C16—C23—C26	112.19 (11)
C9—C10—C11	120.61 (15)	C24—C23—C26	108.63 (13)
C10—C11—C6	119.85 (14)	C25—C23—C26	108.02 (12)

Table 3

Hydrogen-bond geometry (Å, º)

D—H⋯*A*	*D*—H	H⋯*A*	*D*⋯*A*	*D*—H⋯*A*
C20—H201⋯O1	0.95	2.42	3.016 (2)	120 (1)
C22—H221⋯O1	0.97	2.43	3.051 (2)	122 (1)
O1—H11⋯N14[i]	0.84	1.99	2.822 (2)	175 (2)

checkCIF/PLATON report

Alert level G

```
PLAT002_ALERT_2_G Number of Distance or Angle Restraints on AtSite 53 Note
PLAT152_ALERT_1_G The Supplied and Calc. Volume s.u. Differ by ... 3 Units
PLAT230_ALERT_2_G Hirshfeld Test Diff for C5 --C13 . 6.3 s.u.
PLAT791_ALERT_4_G Model has Chirality at C5 (Chiral SPGR) R Verify
PLAT860_ALERT_3_G Number of Least-Squares Restraints ............. 116 Note
PLAT882_ALERT_1_G No Datum for _diffrn_reflns_av_unetI/netI ...... Please Do !
```

8: References:

1. Y. Qin, L. Zhang, J. Lv, S. Luo, J. P Cheng, *Org. Lett.* **2015**, *17*, 1469.

2. M. Uyanik, N. Sasakura, M. Kuwahata, Y. Ejima, K. Ishihara, *Chem Lett.* **2015** *44*, 381.

3. M. Kischkewitz, K. Okamoto, C. Mück-Lichtenfeld, A. Studer, *Science* **2017**, *355*, 936.

4. T. Hashimoto, Y. Kawamata, K. Maruoka, *Nat. Chem*. **2014**, *6,* 702.

5. V. Cherkasov, N. Druzhkov, T. Kocherova, G. Fukin, A. Shavyrin, *Tetrahedron* **2011**, *67*, 80.

6. B. Thiedemann, P. J. Gliese, J. Hoffmann, P. G. Lawrence, F. D. Sönnichsen, A. Staubitz, *Chem. Commun.* **2017**, *53*, 7258.

7. S. Stotani, C. Lorenz, M. Winkler, F. Medda, E. Picazo, R. O. Martinez, A. Karawajczyk, J. Sanchez-Quesada, F. Giordanetto, *ACS Comb. Sci.* **2016**, *18*, 330.

8. J. Zhang, Y. Li, F. Zhang, C. Hu, Y. Chen, *Angew. Chem. Int. Ed.***2016**, *55*, 1872.

9. B. Giese, T. Linker, *Synthesis*, **1992**, 46-48

10. G. J. Choi, Q. Zhu, D.-C. Miller, C. J. Gu, R. R. Knowles, *Nature* **2016**, *539*, 268.

11. P. Gildner, A. A. S. Gietter, D. Cui, D. A. Watson, *J. Am. Chem. Soc.*, **2012**, *134* 9942.
